# Gains of ubiquitylation sites in highly conserved proteins in the human lineage

**DOI:** 10.1186/1471-2105-13-306

**Published:** 2012-11-17

**Authors:** Dong Seon Kim, Yoonsoo Hahn

**Affiliations:** 1 Department of Life Science, Research Center for Biomolecules and Biosystems, Chung-Ang University, Seoul, 156-756, Korea

**Keywords:** Ubiquitylation, Human evolution, Human genome, Molecular evolution

## Abstract

**Background:**

Post-translational modification of lysine residues of specific proteins by ubiquitin modulates the degradation, localization, and activity of these target proteins. Here, we identified gains of ubiquitylation sites in highly conserved regions of human proteins that occurred during human evolution.

**Results:**

We analyzed human ubiquitylation site data and multiple alignments of orthologous mammalian proteins including those from humans, primates, other placental mammals, opossum, and platypus. In our analysis, we identified 281 ubiquitylation sites in 252 proteins that first appeared along the human lineage during primate evolution: one protein had four novel sites; four proteins had three sites each; 18 proteins had two sites each; and the remaining 229 proteins had one site each. PML, which is involved in neurodevelopment and neurodegeneration, acquired three sites, two of which have been reported to be involved in the degradation of PML. Thirteen human proteins, including ERCC2 (also known as XPD) and NBR1, gained human-specific ubiquitylated lysines after the human-chimpanzee divergence. ERCC2 has a Lys/Gln polymorphism, the derived (major) allele of which confers enhanced DNA repair capacity and reduced cancer risk compared with the ancestral (minor) allele. NBR1 and eight other proteins that are involved in the human autophagy protein interaction network gained a novel ubiquitylation site.

**Conclusions:**

The gain of novel ubiquitylation sites could be involved in the evolution of protein degradation and other regulatory networks. Although gains of ubiquitylation sites do not necessarily equate to adaptive evolution, they are useful candidates for molecular functional analyses to identify novel advantageous genetic modifications and innovative phenotypes acquired during human evolution.

## Background

Ubiquitin is a 76-residue polypeptide that is highly conserved among eukaryotes. Ubiquitylation of the lysine residues of substrate proteins targets the ubiquitylated proteins for degradation by the proteasome [1]. The ubiquitin-proteasome system is required for targeted degradation of key regulatory proteins and misfolded proteins [2]. Ubiquitin and ubiquitin-like proteins, such as SUMO, ISG15, NEDD8, and ATG8, function as critical regulators of many cellular processes including signal transduction, cell-cycle control, and transcription [1]. Ubiquitylation is known to crosstalk with the phosphorylation process to modulate various regulatory networks [3]. For example, protein kinases can be regulated negatively or positively through ubiquitylation with or without degradation [3-5].

A large number of genetic modifications have occurred in the human lineage during primate evolution that might be responsible for the emergence of human phenotypes [6,7]. These genetic modifications include the generation of novel genes and transcript variants [8,9], loss of genes [10,11], and acceleration of substitutions in specific nucleotide and amino acid sequences [12,13]. For example, the FOXP2 protein, which is implicated in speech and language in humans, acquired two amino acid substitutions specific to humans after the divergence of humans and chimpanzees [12]. In contrast to chimpanzee FOXP2, human FOXP2 differentially regulates genes involved in central nervous system development [14]. Introduction of amino acids that are subject to post-translational modification (PTM), such as phosphorylation, during evolution, may be responsible for the reorganization of regulatory circuits [15]. Some novel phosphorylation modification sites in human proteins that originated after the divergence of humans and chimpanzees have been identified [16].

To assess the impact of PTMs on human proteome evolution and to identify candidates for evolutionarily innovative PTM sites, a large amount of PTM data from human cells is needed. Recent progress in high-throughput screening by mass spectrometric analysis has enabled the large-scale characterization of PTM sites in the human proteome, including phosphorylation sites [17,18], O-linked β-*N*-acetylglucosamine modification sites [19], lysine acetylation sites [20], and ubiquitylation sites [21-25].

We hypothesize that appearance of novel ubiquitylation sites in proteins along the human lineage during primate evolution may have modified protein regulatory networks, potentially resulting in the acquisition of novel phenotypic traits. To address this possibility, we developed a bioinformatics method to systematically identify gains of novel ubiquitylation sites in the human lineage during primate evolution. As a pilot study, we used ubiquitylation data for human proteins reported by Kim *et al.*[22] and Wagner *et al.*[24] as input data and then analyzed multiple sequence alignments of orthologous proteins from 37 mammalian species, including humans and 10 other primates. We then determined when the ubiquitylated lysine residues of the human proteins first appeared during primate evolution. Kim *et al.* and Wagner *et al.*’s datasets include lysines modified not only by ubiquitin, but also by ubiquitin-like proteins such as SUMO, ISG15, and NEDD8. In this report, we therefore use the term “ubiquitylation” to indicate both ubiquitin and ubiquitin-like protein modifications.

## Results

### Detection and timing of gains of ubiquitylated lysines during human evolution

**Figure 1:**
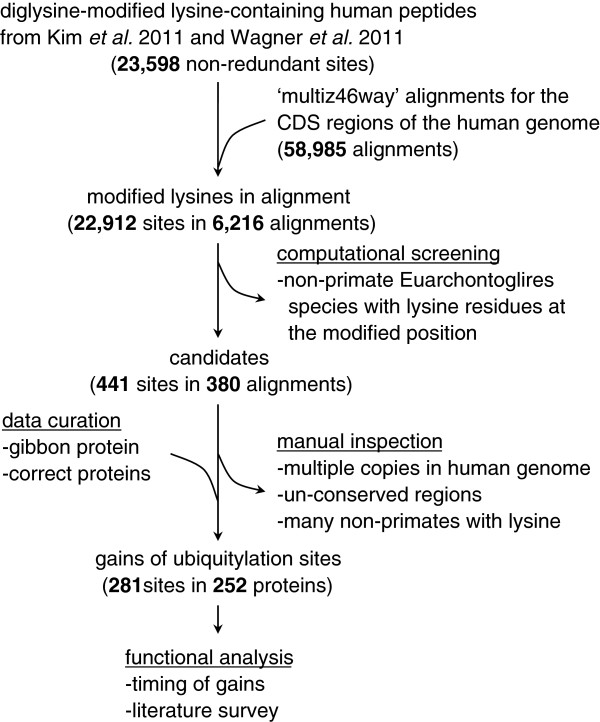
**Procedure for identifying gains of ubiquitylation sites during human evolution.** Computational screening and manual inspection were employed to identify novel gains of ubiquitylation sites in the human lineage since divergence from the common ancestor of Euarchontoglires.

We aimed to identify human ubiquitylated lysines located in highly conserved regions of mammalian proteins that first appeared along the human lineage during primate evolution. To do this, a large amount of ubiquitylation site data and multiple sequence alignments of orthologous mammalian proteins are required. To assess ubiquitylation sites, one can use databases containing PTM data, such as UniProt (http://www.uniprot.org) and PhosphoSitePlus (http://www.phosphosite.org) [26], or large-scale analysis datasets [21-23,25]. In this study, as input data, we used 23,598 non-redundant human ubiquitylation sites collected from the datasets of Kim *et al.*[22] and Wagner *et al.*[24], as well as 58,985 mammalian protein alignments derived from the ‘multiz46way’ alignment data [27]. The overall procedure is illustrated in Figure 1. We filtered out cases where any Euarchontoglires species or many non-Euarchontoglires mammals had the lysine, or those where there were multiple copies of the protein in the human genome or the sequence conservation level was low. Finally, we identified 281 ubiquitylated lysines in highly conserved regions of 252 proteins that appeared in the human lineage during primate evolution. A summary of our results is presented in Additional file 1 and detailed alignments are provided in Additional file 2. Of the 252 proteins, one protein (NUP205) acquired four ubiquitylation sites; four proteins (AKAP12, PML, RAD18, and XRCC5) acquired three sites each; 18 proteins acquired two sites each; and the remaining 229 proteins acquired one site each.

The timing of the gain of a ubiquitylated lysine was determined by finding the branch that enclosed the earliest shared lysine between humans and other primates on the mammalian phylogenetic tree. For example, the human PML residue Lys 394 (No. 182 in Additional file 2) is shared with chimpanzee, gorilla, and orangutan, but not with gibbon and other early-diverged primates. Hence, this lysine was gained in the ancestor of the great apes after they diverged from gibbons. In some cases, the timing could not be determined precisely due to a lack of informative sequences. For example, Lys 448 of the human BIRC2 protein (No. 28 in Additional file 1) is shared with the other great apes (chimpanzee, gorilla, and orangutan) but not with other primates that diverged earlier. Because the gibbon sequence is missing, however, it is not clear whether the gain of Lys 448 occurred in the ape clade (before the divergence of gibbons) or in the great ape clade (after the divergence of gibbons). In such ambiguous cases, we inferred that the novel lysine residue was gained in the smallest clade that included all the species with the novel lysine residue.

**Figure 2:**
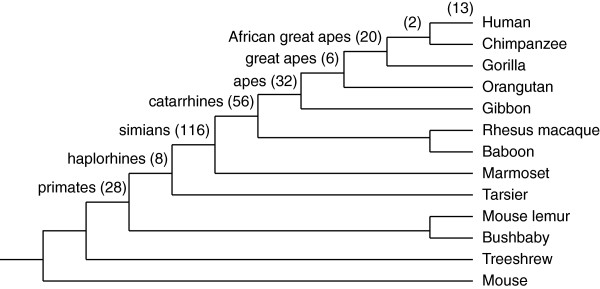
**Timing of the gains of ubiquitylated lysine in the human lineage.** Numbers of gains of ubiquitylated lysine residues in the human lineage of the mammalian phylogenetic tree are shown. The number of gains is shown on each branch where the lysine residue emerged in the ancestor of the corresponding clade.

In Figure 2, the distribution of the 281 ubiquitylated lysines gained in the human lineage is shown in the context of the mammalian phylogenetic tree. The numbers of lysine gains in each clade of the human lineage were as follows: humans, 13; humans and chimpanzees, 2; African great apes, 20; great apes, 6; apes, 32; catarrhines (Old World monkeys and apes), 56; simians (monkeys and apes), 116; haplorhines (tarsiers, monkeys, and apes), 8; and primates, 28. When we surveyed the UniProt database to determine the molecular function of the novel ubiquitylation sites, we found that only two (Lys 400 and Lys 401 of the PML protein) have been functionally characterized (see below for details). The potential functional roles of the remaining 279 sites have yet to be determined.

### Human-specific gains of ubiquitylation sites

**Figure 3:**
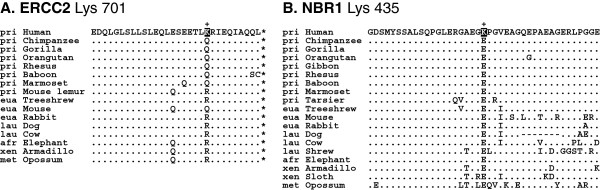
**Multiple sequence alignments of representative human-specific gains of ubiquitylation sites.** Human-specific ubiquitylation sites, which are marked by plus signs (+), and the surrounding regions for ERCC2 (**A**) and NBR1 (**B**) proteins are shown. The gained lysine residues are highlighted on a black background. The residues that are the same as those in the human sequence are marked with dots (.). Dashes (−) and asterisks (*) denote alignment gaps and stop codons, respectively. Unknown amino acids are indicated by ‘X’. Some of the non-primate species were removed to save space (see Additional file 2 for complete data). The three-letter code preceding each species refers to the major mammalian clade to which that species belongs: pri, Primates; eua, Euarchontoglires; lau, Laurasiatheria; afr, Afrotheria; xen, Xenarthra; met, Metatheria; and pro, Prototheria.

Of the 281 ubiquitylation sites, 13 sites were human-specific; that is, these ubiquitylated lysine residues evolved in humans after the divergence of humans and chimpanzees. These proteins are CASC5, CIAPIN1, DSC3, ERCC2, FANCA, KIAA1731, MYO6, NBR1, NCAPD2, SCO2, SDR42E1, SLX4, and TRMT6 (Table 1). In DSC3, ERCC2, and SDR42E1, the novel lysine position was polymorphic in humans, and the derived lysine allele was the major allele while the ancestral (minor) allele was shared with chimpanzees and other apes. Multiple sequence alignments for ERCC2 Lys 701 and NBR1 Lys 435, the two representative human-specific gains, are shown in Figure 3.

**Table 1:** List of proteins with human-specific ubiquitylation sites

**No**^**a**^	**Protein**	**IPI accession**	**Modification site**^**b**^	**Position**^**c**^	**Experiment**^**d**^	**Title**
38	CASC5	IPI00163659.6	QMHVSL**K**EDENNS	262	Kim	cancer susceptibility candidate 5
49	CIAPIN1	IPI00387130	VSVENI**K**QLLQSA	48	Wagner	cytokine induced apoptosis inhibitor 1
67	DSC3	IPI00031549	SGRGVD**K**EPLNLF	180	Wagner	desmocollin 3
75	ERCC2	IPI00442420.2	ESEETL**K**RIEQIA	701	Kim	excision repair cross-complementing rodent repair deficiency, complementation group 2
82	FANCA	IPI00006170.2	GRSLEL**K**GQGNPV	1387	Kim	Fanconi anemia, complementation group A
118	KIAA1731	IPI00400986.6	SGTIAS**K**ERTLSS	435	Kim	KIAA1731
150	MYO6	IPI00844172.1	AQLARQ**K**EEESQQ	993	Kim	myosin VI
155	NBR1	IPI00299920.5	ERGAEG**K**PGVEAG	435	Kim	neighbor of BRCA1 gene 1
156	NCAPD2	IPI00299524.1	RGLDGI**K**ELEIGQ	1301	Kim, Wagner	non-SMC condensin I complex, subunit D2
214	SCO2	IPI00014458	GLTGST**K**QVAQAS	196	Wagner	SCO cytochrome oxidase deficient homolog 2 (yeast)
215	SDR42E1	IPI00163504.4	LNRNLI**K**EVNVRG	96	Kim	short chain dehydrogenase/reductase family 42E, member 1
234	SLX4	IPI00291796.2	SDPLEE**K**KALEIS	1179	Kim	SLX4 structure-specific endonuclease subunit homolog (*S. cerevisiae*)
259	TRMT6	IPI00099311	HGTFSA**K**MLSSEP	273	Wagner	tRNA methyltransferase 6 homolog (*S. cerevisiae*)

^a^The number corresponds to that in Additional file 1 and in Additional file 2.

^b^The ubiquitylated lysine is in bold.

^c^The positions are based on the International Protein Index (IPI) records and may differ from those of the UniProt or NCBI Protein records.

^d^Experimental evidence for modifications: Kim, Kim *et al.*[22]; Wagner, Wagner *et al.*[24].

The ERCC2 (excision repair cross-complementing rodent repair deficiency, complementation group 2) protein, which is also known as XPD, is involved in transcription-coupled nucleotide excision repair and is implicated in cancer-prone xeroderma pigmentosum, trichothiodystrophy, and Cockayne syndrome [28]. In the highly conserved C-terminal region of this protein, there is a human-specific ubiquitylated residue, Lys 701 (equivalent to Lys 751 of UniProt record P18074); other mammals have either a glutamine (Q) or an arginine (R) at this position (Figure 3A and No. 75 in Additional file 2). Interestingly, this position is polymorphic in humans (Lys/Gln; dbSNP accession rs13181). The lysine (codon AAG) is the derived allele while the glutamine (codon CAG) is the ancestral allele that is shared with other apes and monkeys. In the human population, the derived lysine allele is the major allele with a frequency of 73.285%. Humans with the ancestral (minor) glutamine allele have reduced DNA repair capacity, indicating that the derived lysine allele confers enhanced DNA repair capacity [29,30]. Hence, the gain of a lysine at this position is advantageous in humans, although an association between ubiquitylation of the lysine and enhanced DNA repair capacity remains to be demonstrated.

The neighbor of BRCA1 gene 1 (NBR1) protein has been identified as one of the principle cargo receptors for selective autophagy of ubiquitylated targets [31,32]. Abnormalities in NBR1 have been implicated in a type of progressive degenerative myopathy of older persons [33]. In a highly conserved region of NBR1, there is a human-specific ubiquitylated residue, Lys 435, at which position all the other mammals examined have an glutamic acid (E) (Figure 3B and No. 155 in Additional file 2). This novel ubiquitylation site could play a role in the degradation or molecular function of NBR1. However, it is also possible that the ubiquitylation of Lys 435 was simply an indication of NBR1 degradation at the timepoint the experiment was performed.

### Other notable gains of ubiquitylation sites

**Figure 4:**
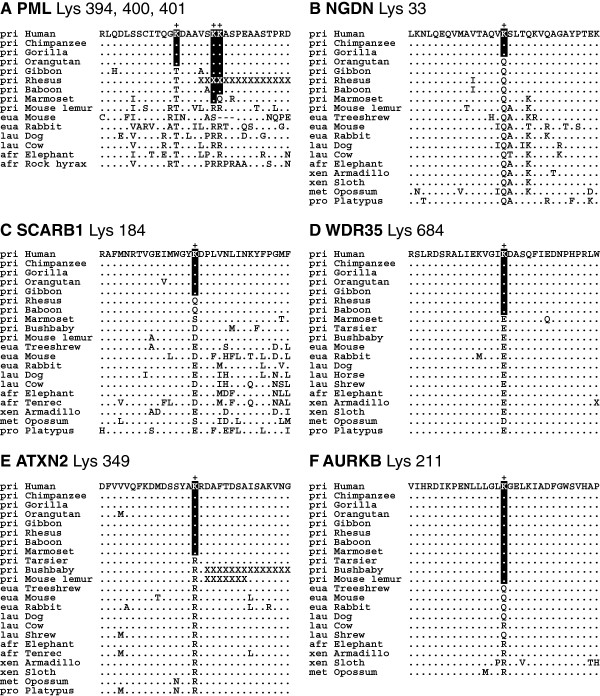
**Multiple sequence alignments of representative gains of ubiquitylation sites in the human lineage during primate evolution.** Novel ubiquitylation sites (+) and the surrounding regions for PML (**A**), NGDN (**B**), SCARB1 (**C**), WDR35 (**D**), ATXN2 (**E**), and AURKB (**F**) proteins are presented. See Figure 3 for manipulations and Additional file 2 for complete data.

Of the 281 ubiquitylation sites, 269 sites in 243 human proteins were acquired along the human lineage during primate evolution, and are shared with chimpanzees and other primates (see Figure 4 for representative cases). The promyelocytic leukemia (PML) protein acquired three novel ubiquitylation sites in the human lineage: Lys 394 in the great apes, Lys 400 in the simians, and Lys 401 in the catarrhines (Figure 4A and Nos. 182–184 in Additional file 2). These three sites are located within an eight amino acid range of one another. Two of these sites, Lys 400 and 401, are modified by RNF4, which is required for arsenic-induced PML degradation [34]. The *PML* gene is often fused with the retinoic acid receptor α (*RARA*) gene, which is associated with acute promyelocytic leukemia [35]. Interestingly, recent studies revealed that PML has roles in neurodevelopment and neurodegeneration [36]. It would be very interesting to investigate if the gain of these three ubiquitylation sites is associated with the evolution of the human nervous system.

Human neuroguidin (NGDN) has a ubiquitylated Lys 33 that is shared with chimpanzees and gorillas, while other early-diverged primates (including orangutans) and all other mammals examined have a glutamine (Q) residue at this position (Figure 4B and No. 159 in Additional file 2). NGDN functions as a translational regulatory protein by interacting with eukaryotic initiation factor 4E (EIF4E) and cytoplasmic polyadenylation element binding (CPEB) protein, and is required for the development of the vertebrate nervous system [37].

The scavenger receptor class B member 1 (SCARB1) protein is a plasma membrane receptor for high-density lipoprotein cholesterol (HDL). It mediates cholesterol transfer to and from HDL [38] and is implicated in hepatitis C virus entry [39]. In this study, SCARB1 Lys 184 was identified as one of 32 ubiquitylation sites that were acquired in the apes (Figure 4C and No. 212 in Additional file 2).

We found that 56 novel ubiquitylation sites in 54 proteins first appeared in the common ancestor of catarrhine primates. One representative case is WD repeat-containing protein 35 (WDR35) Lys 684, at which position most other mammals have a glutamic acid (E) (Figure 4D and No. 273 in Additional file 2). WDR35 has been implicated in spontaneous and tumor necrosis factor α-stimulated apoptosis [40]. WDR35 is required for cilia production; its disruption results in a range of human ectodermal, visceral, and skeletal abnormalities [41,42].

Of the 281 novel human ubiquitylated lysines, 116 in 107 proteins are shared with simians. One example is ataxin 2 (ATXN2) Lys 349, at which position all the other mammals examined have an arginine (R) (Figure 4E and No. 23 in Additional file 2). Expansion of a CAG repeat of the *ATXN2* gene causes spinocerebellar ataxia type 2 [43].

There were 28 human ubiquitylated lysines in 28 proteins that were shared by all primates identified in this study. For example, aurora kinase B (AURKB) Lys 211 first appeared in primates after their divergence from the common ancestor of Euarchontoglires and is shared in all primates examined (Figure 4F and No. 24 in Additional file 2). Non-primate mammals have either a glutamine (Q) or an arginine (R) at this position. Aurora kinase B is a component of the chromosomal passenger complex that functions as a key regulator of mitosis [44] and is ubiquitylated by a Cullin 3-based E3 ubiquitin ligase during mitosis, which coordinates precise mitotic progression and completion of cytokinesis [45,46].

## Discussion

This report presents the results of a pilot study to systematically identify gains of novel ubiquitylation sites in the human lineage since its divergence from the common ancestor of Euarchontoglires. To achieve this goal, we analyzed a human ubiquitylation dataset obtained from large-scale analyses [22,24]. We identified 281 novel ubiquitylation sites in 252 highly conserved proteins that first appeared in the human lineage during primate evolution, 13 of which are human-specific. We anticipate that application of our method to analyze the ubiquitylation data recorded in databases such as UniProt and PhosphoSitePlus [26] or collected by other large-scale analyses [21,23,25] will result in identification of additional instances of gains of novel ubiquitylated lysines along the human lineage. We also expect that additional novel ubiquitylation sites will be discovered when higher quality protein sequences of non-human mammals become available. The total number of novel ubiquitylation sites we collected is likely to be an underestimate because of the draft quality of non-human genomes.

In addition to ubiquitylation, lysine residues can be modified by acetylation, and the cross-talk between these two lysine modifications is an important regulatory mechanism [47]. Wagner *et al.*[24] showed that 1,040 ubiquitylated lysines were also acetylated by comparing their 11,054 ubiquitylation sites with the 3,428 acetylation sites reported by Choudhary *et al.*[20]. To check whether any novel ubiquitylation sites identified in this study are also acetylated, we compared our data with 3,948 non-redundant acetylation sites collected from the UniProt database and Choudhary *et al.* dataset. We found that nine ubiquitylated lysines were also acetylated. These are DLD Lys 320, FASN Lys 436, FDPS Lys 353, GAPDH Lys 84, LDHA Lys 251, LRPPRC Lys 613, MCM5 Lys 696, NUP205 Lys 41, and PARP10 Lys 928 (Nos. 63, 85, 89, 96, 125, 128, 135, 170, and 173, respectively, in Additional files 1 and 2). Thus, these nine newly-gained lysines can be modified not only by ubiquitylation but also by acetylation, suggesting regulatory cross-talk between lysine ubiquitylation and acetylation.

Although gains of novel ubiquitylation sites do not necessarily equate to innovative and adaptive changes, they are useful candidates to evaluate when searching for advantageous genetic modifications during human evolution. It is also possible that the modified peptides could be simply derived from protein molecules destined to be degraded or being degraded in the proteasome at the time of the experiment. Nevertheless, new ubiquitylation sites would provide novel target sites to modulate cellular processes by fine-tuning degradation, intracellular localization, or the regulatory network. Recently, the origins and evolution of mammalian and yeast ubiquitylation sites were evaluated by analyzing their eukaryotic and prokaryotic orthologs [48]. The study revealed that ubiquitylation sites evolved at a similar rate to other protein modification sites such as phosphorylation sites, and that about 70% of 452 mammalian ubiquitylation sites first appeared during early vertebrate evolution. Interestingly, some ubiquitylation sites that appeared during animal evolution have been suggested to be associated with development of novel cross-talk pathways with other modifications such as phosphorylation and hydroxylation. This report supports our notion that gain of novel ubiquitylation sites could result in the evolution of protein regulatory networks.

In the case of ERCC2, the human-specific ubiquitylated lysine site is polymorphic in humans. The derived lysine allele is the major or normal allele, while the ancestral (minor) glutamine allele is designated as the mutant, which shows reduced DNA repair capacity; carriers of this minor allele therefore have an increased cancer risk [28]. The gain of a ubiquitylated lysine in ERCC2 can be regarded as a concrete example of adaptive gains identified in this study. Molecular functional analyses of ubiquitylation sites collected in this study are likely to reveal more instances of advantageous functional outcomes.

Interestingly, among the 252 proteins, nine proteins (DZIP3, FKBP4, KIF23, NBR1, PFKP, PIK3C2A, PRKDC, SNAP23, and ZWINT) have been found in human autophagy protein interaction networks [49]. NBR1 has been proposed to act as one of the principle receptors for selective autophagosomal degradation of ubiquitylated targets [31,32]. Human NBR1 acquired a human-specific ubiquitylated residue, Lys 435, after the divergence of humans and chimpanzees. Eight other human proteins have novel ubiquitylated lysines that are shared with other primates. These nine proteins interact with known autophagy proteins such as N-ethylmaleimide-sensitive factor (NSF) and beclin 1, autophagy related (BECN1) [49]. It is possible that the gain of new ubiquitylation sites could provide novel regulatory interactions for autophagy and/or other programmed protein degradation processes.

**Figure 5:**
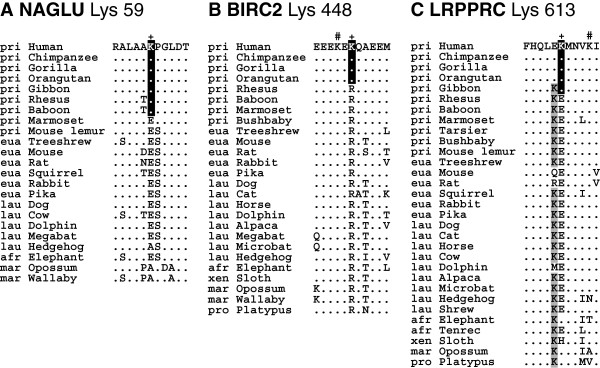
**Representative cases of new, additional, and shifted ubiquitylation sites.** Novel ubiquitylation sites (+) and the surrounding regions for NAGLU (**A**), BIRC2 (**B**), and LRPPRC (**C**) proteins are presented. Ancestrally conserved lysine residues in the LRPPRC protein that disappeared in great apes are highlighted on a gray background. Hash symbols (#) indicate ubiquitylated lysines that were experimentally validated in humans. See Figure 3 for more manipulations.

Hagai *et al.*[48] showed that some non-conserved ubiquitylated lysines are compensated for by nearby lysines, indicating that ubiquitylation sites can move from their original locations during evolution. In these case, the exact position of the ubiquitylation site is not critical for the regulation of the protein and may move over time; this phenomenon has also been observed in studies of phosphorylation sites [50]. To explore this possibility, we determined whether an alternative ancestral lysine residue was found in a small window surrounding the novel ubiquitylated lysine. We analyzed a window of ±5 residues (from −5 to +5) centered on the novel ubiquitylated lysine. A highly conserved lysine residue suggests that the site is a target for ubiquitin/ubiquitin-like protein modification. We found that 160 cases of 281 had no conserved additional lysine within the ±5 residue window, indicating that the sites that we identified are indeed new ubiquitylation sites. For example, the human-specific lysines of ERCC2 (Lys 701) and NBR1 (Lys 435) (see Figure 3) were the only modifiable residues in the window evaluated. Another example is NAGLU Lys 59 (Figure 5A), which is shared by all catarrhine primates. In 91 cases, there are one or more conserved lysines close to the novel ubiquitylated lysine. In these cases, we assumed that the protein acquired additional ubiquitylation site along the human lineage. As shown in Figure 5B, there is a highly conserved lysine in the BIRC2 protein that is ubiquitylated in the human protein at the −2 position from the novel ubiquitylated lysine 448. In the remaining 30 cases, the ancestrally conserved lysine disappeared as the novel lysine appeared along the human lineage, suggesting that the ubiquitylation site may have shifted. For example, there is a novel lysine residue (Lys 613) in the LRPPRC protein (Figure 5C) that first appeared in the common ancestor of apes. At the −1 position from this novel site, there is an ancestrally conserved lysine in mammals, including gibbons, but not in great apes, suggesting that the modified position moved by a single residue during evolution. This analysis indicates that the majority of the novel ubiquitylation sites identified in this study, 251 sites out of 281, are new or additional ubiquitylation targets.

## Conclusions

We developed a bioinformatics method to identify novel ubiquitylation sites that evolved along the human lineage, resulting in the identification of 281 novel ubiquitylation sites. The gain of novel ubiquitylation sites could result in novel ubiquitin-associated protein regulatory interactions. Proteins with a novel ubiquitylation site are useful candidates in the search for genetic modifications implicated in the emergence of novel phenotypes during human evolution.

## Methods

### Datasets and bioinformatics tools

To identify ubiquitylation sites in human proteins, we used the large-scale analysis datasets of Kim *et al.*[22] and Wagner *et al.*[24]. These researchers utilized a monoclonal antibody that recognizes characteristic diglycine-containing isopeptides following trypsin proteolysis [51]. Peptide sequences with the modified lysine residue at the center were mapped to human protein sequences to identify them.

Multiple sequence alignments of the human proteins and orthologous proteins from other mammalian species were obtained from the University of California Santa Cruz (UCSC) Genome Browser Database (http://genome.ucsc.edu). The ‘CDS FASTA alignment from multiple alignment’ data, which are derived from the ‘multiz46way’ alignment data [27], were downloaded using the Table Browser tool of the UCSC Genome Browser. These alignment datasets included 36 mammalian species: humans, nine other primates (chimpanzee, gorilla, orangutan, rhesus macaque, baboon, marmoset, tarsier, bushbaby, and mouse lemur), eight other Euarchontoglires (treeshrew, mouse, rat, kangaroo rat, guinea pig, squirrel, rabbit, and pika), ten Laurasiatheria (dog, cat, horse, cow, dolphin, alpaca, megabat, microbat, hedgehog, and shrew), three Afrotheria (elephant, rock hyrax, and tenrec), two Xenarthra (armadillo and sloth), two Marsupialia (opossum and wallaby), and one Prototheria (platypus) species. The gibbon protein sequences, which were missing from the multiz46way data, were predicted from the genome assembly (nomLeu1) and included in the final alignment, resulting in 37 mammalian species, including 10 non-human primates. The phylogenetic tree of the 37 mammals used in this study is presented in Additional file 3.

The National Center for Biotechnology Information (NCBI) Protein database (http://www.ncbi.nlm.nih.gov/protein) was used to collect protein sequences for some species. The multiple sequence alignments were generated using MUSCLE (http://www.drive5.com/muscle).

### Computational screening for candidate novel ubiquitylation sites

The overall procedure employed in this study is presented in Figure 1. The total number of non-redundant ubiquitylation sites used was 23,598 [22,24]. We compared the peptide sequences containing the ubiquitylation site and the human proteins in the multiz46way (58,985 sets) to collect orthologous protein alignments. We found 22,912 human ubiquitylation sites in 6,216 protein alignments. We analyzed each modification site in the alignment and discarded cases where non-primate Euarchontoglires species (treeshrew, mouse, rat, kangaroo rat, guinea pig, squirrel, rabbit, and pika) had a lysine residue that was aligned with the ubiquitylated lysine of the human proteins. A total of 441 sites in 380 protein alignments were retained after this computational screening step and subjected to manual inspection.

### Manual inspection to select ubiquitylated lysine residues that appeared along the human lineage

As the final step, we manually examined the 441 candidates to identify plausible cases of gains of ubiquitylation sites in the human lineage during primate evolution. First, when multiple copies of the human protein sequence in a dataset were present in the human genome, the set was discarded due to uncertainty about the orthology of the aligned proteins. We also discarded cases showing low sequence conservation and cases where many non-primate proteins had lysine residues that were aligned with the human ubiquitylated lysine.

Next, we curated each protein dataset. Because the original multiz46way data set did not include gibbon sequences, we identified and added the orthologous gibbon proteins to the dataset. Proteins with low quality sequences, with missing amino acids, or derived from older genome assemblies were replaced with curated sequences retrieved from the NCBI Protein database or newly predicted sequences from the most recent assemblies. Some protein sequences with low quality regions or gaps that could not be amended were removed from the dataset. The multiple sequence alignment was rebuilt using MUSCLE.

Finally, 281 sites in 252 proteins were collected. We examined the multiple alignments to estimate the timing of the gain of the ubiquitylated lysine residue. Possible functional consequences of the gain of the ubiquitylation site were assessed by a literature survey. The positions of the residues noted in this manuscript are derived from the datasets of Kim *et al.*[22] and Wagner *et al.*[24], which are, in turn, based on the International Protein Index (IPI) (http://www.ebi.ac.uk/IPI) and may differ from those of the UniProt or NCBI Protein databases.

## Competing interests

The authors declare that they have no competing interests.

## Authors’ contributions

YH conceived of this study, conducted the programming work, and prepared the manuscript. DSK participated in the sequence analysis. Both authors read and approved the final manuscript.

## Supplementary Material

Additional file 1 List of proteins with novel ubiquitylation sites. Click here for file

Additional file 2 Detailed alignments of surrounding regions of novel ubiquitylation sites. Click here for file

Additional file 3 Phylogenetic tree of the 37 mammals used in this study. Click here for file
