# Supplementary material for: Gains of ubiquitylation sites in highly conserved proteins in the human lineage
Source: BMC Bioinformatics. 2012 Nov 17;13:306. doi: 10.1186/1471-2105-13-306 (PMC3561281; doi:10.1186/1471-2105-13-306)
Supplement: Additional file 1 — List of proteins with novel ubiquitylation sites. [file 1471-2105-13-306-S1.pdf]

Supplementary Table S1. List of proteins with novel ubiquitylated sites.

No	Protein	IPI accession	Modification site	Position	Experiment	Clade	Title
1	ABCB1	IPI00027481	YDPLAGKVLLDGK	1093	Wagner	simians	ATP-binding cassette, sub-family B (MDR/TAP), member 1
2	ABCC3	IPI00006674.1	RILEAAKGEIRID	1344	Kim	simians	ATP-binding cassette, sub-family C (CFTR/MRP), member 3
3	ADAM15	IPI00013302.1	DLPISLKKVLQTS	58	Kim	catarrhines	ADAM metallopeptidase domain 15
4	ADAM15	IPI00013302.1	LPISLKKVLQTSL	59	Kim	catarrhines	ADAM metallopeptidase domain 15
5	ADNP2	IPI00402209.3	TEGPIVKDEALQI	1032	Kim	simians	ADNP homeobox 2
6	AEN	IPI00074083.5	RRPAPGKASGPLP	101	Kim	apes	apoptosis enhancing nuclease
7	AHCTF1	IPI00170594.5	GEVWASKEPINST	1078	Kim	simians	AT hook containing transcription factor 1
8	AHR	IPI00021008.1	RHMQNEKFFRNDF	560	Kim	primates	aryl hydrocarbon receptor
9	AKAP12	IPI00237884.3	QEAEPAKELVKLK	461	Kim	simians	A kinase (PRKA) anchor protein 12
10	AKAP12	IPI00237884.3	AATEVSKELSESQ	882	Kim	simians	A kinase (PRKA) anchor protein 12
11	AKAP12	IPI00237884.3	IVSATTKKGLSSD	1493	Kim	African great apes	A kinase (PRKA) anchor protein 12
12	ALDH2	IPI00006663.1	GNPFDSKTEQGPQ	355	Kim	primates	aldehyde dehydrogenase 2 family (mitochondrial)
13	ALG8	IPI00032370	DGSIRWKSFSFVR	231	Wagner	apes	asparagine-linked glycosylation 8, alpha-1,3-glucosyltransferase homolog (S. cerevisiae)
14	ANGEL2	IPI00375317.2	LTQTQLKQTEVLV	261	Kim	primates	angel homolog 2 (Drosophila)
15	ANKIB1	IPI00292914.4	DGSEGVKDVELVL	1012	Kim	simians	ankyrin repeat and IBR domain containing 1
16	ANKRD13A	IPI00217831	LHLLVWKNDYRQL	19	Wagner	catarrhines	ankyrin repeat domain 13A
17	ANXA3	IPI00024095.3	DAQILYKAGENRW	184	Kim	catarrhines	annexin A3
18	AP1M2	IPI00219794.5	GDVAMSKIEHFMP	29	Kim	simians	adaptor-related protein complex 1, mu 2 subunit
19	APOB	IPI00022229.1	EIKTLLKAGHIAW	2915	Kim	humans and chimpanzees	apolipoprotein B (including Ag(x) antigen)
20	ARMC10	IPI00420025.2	LGIRSSKSAEDLT	44	Kim	catarrhines	armadillo repeat containing 10
21	ATAD2	IPI00170548.1	PAATTAKAGDGSS	55	Kim	simians	ATPase family, AAA domain containing 2
22	ATP13A2	IPI00015154.1	DTAQLHKSEEAVS	150	Kim	simians	ATPase type 13A2
23	ATXN2	IPI00180154.4	MDSSYAKRDAFTD	349	Kim	simians	ataxin 2
24	AURKB	IPI00176642.3	NLLLGLKGELKIA	211	Kim, Wagner	primates	aurora kinase B
25	BBS2	IPI00306961	NINTLFKIMRVGT	712	Wagner	apes	Bardet-Biedl syndrome 2
26	BFAR	IPI00009811.1	TEEEFSKTPYTIE	225	Kim, Wagner	simians	bifunctional apoptosis regulator
27	BFAR	IPI00009811.1	TKLLDLKEPTWKQ	322	Kim, Wagner	simians	bifunctional apoptosis regulator
28	BIRC2	IPI00013418.1	REEEKEKQAEEMA	448	Kim	great apes	baculoviral IAP repeat containing 2
29	BSG	IPI00019906.1	ITDSEDKALMNGS	148	Kim	African great apes	basigin (Ok blood group)
30	BSN	IPI00020153.4	SKEAGPKPLGSGP	398	Kim	simians	bassoon (presynaptic cytomatrix protein)
31	BTN2A2	IPI00013576.2	RITFVSKDINRGS	105	Kim	primates	butyrophilin, subfamily 2, member A2
32	C16orf42	IPI00011693.4	VLSPVGKQYASPA	103	Kim, Wagner	simians	chromosome 16 open reading frame 42
33	C1orf124	IPI00552744.1	IKKEQIKSSGNDP	428	Kim	apes	chromosome 1 open reading frame 124
34	C3orf75	IPI00107155.2	VVFQAQKEPHPLQ	99	Kim	apes	chromosome 3 open reading frame 75
35	C7orf44	IPI00414548.1	LNIHYLKLIDREN	74	Kim	simians	chromosome 7 open reading frame 44
36	C9orf114	IPI00418229.1	EEAAAEKEDRGRP	66	Kim	catarrhines	chromosome 9 open reading frame 114
37	CAPG	IPI00027341.1	EDLTADKANAQAA	243	Kim	catarrhines	capping protein (actin filament), gelsolin-like
38	CASC5	IPI00163659.6	QMHVSLKEDENNS	262	Kim	humans	cancer susceptibility candidate 5
39	CCDC138	IPI00065415.2	KVGSSLKYSDESK	66	Kim	simians	coiled-coil domain containing 138
40	CCDC14	IPI00396060.5	ETIEPDKTYENVL	681	Kim	simians	coiled-coil domain containing 14

41	CCDC14	IPI00396060.5	SRASDMKDTQLLK	811	Kim	primates	coiled-coil domain containing 14
42	CDC25B	IPI00029734.1	LEKEEEKDLVMYS	328	Kim	African great apes	cell division cycle 25 homolog B (S. pombe)
43	CDK5RAP1	IPI00328278.1	TFQHFLKSASAPQ	70	Kim, Wagner	catarrhines	CDK5 regulatory subunit associated protein 1
44	CENPF	IPI00855998	KTHLQEKLQSLEK	2132	Wagner	catarrhines	centromere protein F, 350/400kDa (mitosin)
45	CENPN	IPI00305656.3	VDLFDMKQFKNSF	102	Kim	primates	centromere protein N
46	CEP250	IPI00160622	CQQKLIKELEGQR	1577	Wagner	African great apes	centrosomal protein 250kDa
47	CEP290	IPI00784201	DLEQQIKILKHVP	2324	Wagner	simians	centrosomal protein 290kDa
48	CHPF	IPI00465319.5	PGAEREKPGAGEG	73	Kim	simians	chondroitin polymerizing factor
49	CIAPIN1	IPI00387130	VSVENIKQLLQSA	48	Wagner	humans	cytokine induced apoptosis inhibitor 1
50	COL6A2	IPI00304840.4	APNQNLKEQGLRD	197	Kim	haplorhines	collagen, type VI, alpha 2
51	CORO7	IPI00027996.1	DGFCANKLRVAVP	508	Kim	simians	coronin 7
52	CORO7	IPI00027996.1	QEGPGPKGGRGAR	680	Kim	simians	coronin 7
53	CPPED1	IPI00305010.3	EQTEDLKRVLRAV	109	Kim	catarrhines	calcineurin-like phosphoesterase domain containing 1
54	CREB3L4	IPI00152889.3	QGLQGWKSGGDRG	51	Kim	simians	cAMP responsive element binding protein 3-like 4
55	CTSA	IPI00640525	RRPWLVKYGDSGE	447	Wagner	simians	cathepsin A
56	CTSD	IPI00011229	LGGKGYKLSPEDY	348	Wagner	catarrhines	cathepsin D
57	CUL7	IPI00001690.2	WRAVVEKQVNNFL	1153	Kim	simians	cullin 7
58	CUL7	IPI00001690.2	QSLSTSKELQRQF	1312	Kim	simians	cullin 7
59	D2HGDH	IPI00166642	PFSTVSKQDLAAF	64	Wagner	apes	D-2-hydroxyglutarate dehydrogenase
60	DCAF6	IPI00164246	SDKFTAKPLDSNS	652	Wagner	haplorhines	DDB1 and CUL4 associated factor 6
61	DKC1	IPI00221394.8	PESKVAKLDTSQW	46	Kim	simians	dyskeratosis congenita 1, dyskerin
62	DKFZp761E198	IPI00386243.3	GPLPVLKLQPEAL	624	Kim, Wagner	catarrhines	adaptor protein 5
63	DLD	IPI00015911	GRRPFTKNLGLEE	320	Wagner	simians	dihydrolipoamide dehydrogenase
64	DNPEP	IPI00015856.5	EVANKVKVPLQDL	417	Kim	catarrhines	aspartyl aminopeptidase
65	DNTTIP2	IPI00290410.3	PKVTPTKESYTEE	132	Kim	simians	deoxynucleotidyltransferase, terminal, interacting protein 2
66	DPH1	IPI00718991.3	IPPEILKNPQLQA	43	Kim	catarrhines	DPH1 homolog (S. cerevisiae)
67	DSC3	IPI00031549	SGRGVDKEPLNLF	180	Wagner	humans	desmocollin 3
68	DTX3L	IPI00152503	EGRKVLKLLYRAF	675	Wagner	African great apes	deltex 3-like (Drosophila)
69	DZIP3	IPI00452463.1	LQEIGDKNDHWFD	212	Kim	catarrhines	DAZ interacting protein 3, zinc finger
70	ECHDC1	IPI00302688.7	LMTPESKIRFVHK	179	Kim	simians	enoyl CoA hydratase domain containing 1
71	EIF2AK2	IPI00019463.3	LFEQITKGVDYIH	400	Kim	catarrhines	eukaryotic translation initiation factor 2-alpha kinase 2
72	ENDOD1	IPI00001952.5	RMVQSQKSSSPLS	300	Kim	catarrhines	endonuclease domain containing 1
73	EPB41L2	IPI00015973.1	AEEMAQKKQEIKV	135	Kim	simians	erythrocyte membrane protein band 4.1-like 2
74	EPCAM	IPI00296215.2	LRTALQKEITTRY	168	Kim	apes	epithelial cell adhesion molecule
75	ERCC2	IPI00442420.2	ESEETLKRIEQIA	701	Kim	humans	excision repair cross-complementing rodent repair deficiency, complementation group 2
76	FADS2	IPI00183786	SLKKSGKLWLDAY	435	Wagner	catarrhines	fatty acid desaturase 2
77	FAM175A	IPI00030384.4	VQTHSSKFFEEDG	201	Kim, Wagner	simians	family with sequence similarity 175, member A
78	FAM175A	IPI00030384.4	NQDKASKMSSPET	384	Kim	simians	family with sequence similarity 175, member A
79	FAM82B	IPI00329696	PSSTYEKALGYFH	243	Wagner	catarrhines	family with sequence similarity 82, member B
80	FAM83D	IPI00480103.3	EMPAEGKAERKPH	385	Kim	primates	family with sequence similarity 83, member D
81	FANCA	IPI00006170.2	LPRELQKLQEGRQ	1199	Kim, Wagner	catarrhines	Fanconi anemia, complementation group A
82	FANCA	IPI00006170.2	GRSLELKGQGNPV	1387	Kim	humans	Fanconi anemia, complementation group A

83	FANCD2	IPI00075081.1	EYFFENKNSDEIN	165	Kim	simians	Fanconi anemia, complementation group D2
84	FANCE	IPI00030252.1	QPVMAVKTGEDGS	260	Kim	simians	Fanconi anemia, complementation group E
85	FASN	IPI00026781.2	TPEAVQKLLEQGL	436	Kim, Wagner	apes	fatty acid synthase
86	FASN	IPI00026781.2	FVEQLRKEGVFAK	667	Kim	catarrhines	fatty acid synthase
87	FAT1	IPI00031411.3	FTASSYKGRVYES	1568	Kim	simians	FAT tumor suppressor homolog 1 (Drosophila)
88	FBXO40	IPI00008203.7	GEGAPKKKEPQEN	266	Kim	simians	F-box protein 40
89	FDPS	IPI00914566.2	EQYQILKENYGQK	353	Kim, Wagner	simians	farnesyl diphosphate synthase
90	FKBP4	IPI00219005.3	EGYYKDKLFDQRE	181	Kim, Wagner	African great apes	FK506 binding protein 4, 59kDa
91	FTSJ1	IPI00004308	KLLQLDKEFQLFQ	34	Wagner	catarrhines	FtsJ homolog 1 (E. coli)
92	GADD45GIP1	IPI00552587	LQDLEKKERKRLK	185	Wagner	simians	growth arrest and DNA-damage-inducible, gamma interacting protein 1
93	GALE	IPI00553131.2	QLLEIMKAHGVKN	120	Kim	catarrhines	UDP-galactose-4-epimerase
94	GALK1	IPI00019383.2	LVGSPRKDGLVSL	99	Kim, Wagner	catarrhines	galactokinase 1
95	GALT	IPI00013925.3	HPLFQAKSARGVC	120	Kim	simians	galactose-1-phosphate uridylyltransferase
96	GAPDH	IPI00219018.7	QERDPSKIKWGDA	84	Kim, Wagner	primates	glyceraldehyde-3-phosphate dehydrogenase
97	GRHPR	IPI00037448.3	QALASGKIAAAGL	262	Kim	apes	glyoxylate reductase/hydroxypyruvate reductase
98	GSDMD	IPI00028027.3	EVETISKELELLD	299	Kim	simians	gasdermin D
99	GSR	IPI00016862	AFTSDPKPTIEVS	181	Wagner	simians	glutathione reductase
100	GSS	IPI00010706.1	EMVQALKQLKDSE	384	Kim	simians	glutathione synthetase
101	GSTO1	IPI00019755.3	ERLEAMKLNECVD	188	Kim	simians	glutathione S-transferase omega 1
102	HEATR2	IPI00242630.3	TLEEDSKMTRLIS	715	Kim	humans and chimpanzees	HEAT repeat containing 2
103	HEBP1	IPI00148063.1	PPAPSDKSVKIEE	118	Kim	simians	heme binding protein 1
104	HPRT1	IPI00218493.7	PRSVGYKPDFVGF	175	Kim, Wagner	catarrhines	hypoxanthine phosphoribosyltransferase 1
105	HPS6	IPI00015505.7	QLDGNGKLRSQAP	534	Kim	primates	Hermansky-Pudlak syndrome 6
106	HSD17B12	IPI00007676.3	VLISRSKDKLDQV	84	Kim	African great apes	hydroxysteroid (17-beta) dehydrogenase 12
107	HSPBAP1	IPI00298207.5	DLFQDVKWSDFGF	147	Kim	primates	HSPB (heat shock 27kDa) associated protein 1
108	HYLS1	IPI00065180.3	VQFQEDKESSFDV	144	Kim	catarrhines	hydrolethalus syndrome 1
109	IFT122	IPI00066817.7	LRLVETKDSIGDE	1141	Kim	simians	intraflagellar transport 122 homolog (Chlamydomonas)
110	ISYNA1	IPI00549569	EAMRRAKVLDWGL	155	Wagner	primates	inositol-3-phosphate synthase 1
111	ITGA3	IPI00215995.1	VSEQQQKLSRLQY	638	Kim	simians	integrin, alpha 3 (antigen CD49C, alpha 3 subunit of VLA-3 receptor)
112	ITGAL	IPI00219896	WLPSRQKTSLLAS	416	Wagner	simians	integrin, alpha L (antigen CD11A (p180), lymphocyte function-associated antigen 1; alpha polypeptide)
113	ITGB1	IPI00217563.4	GTAEKLKPEDITQ	107	Kim	simians	integrin, beta 1 (fibronectin receptor, beta polypeptide, antigen CD29 includes MDF2, MSK12)
114	JMJD4	IPI00011132.2	AFSPQPKELLQQL	447	Kim, Wagner	primates	jumonji domain containing 4
115	KHNYN	IPI00829596.1	GFAEHGKQQQGRE	615	Kim	simians	KH and NYN domain containing
116	KIAA0753	IPI00006499.8	DGRSDPKVLQTQN	26	Kim, Wagner	African great apes	KIAA0753
117	KIAA0753	IPI00006499.8	SSVNTAKAQPAQE	718	Kim	African great apes	KIAA0753
118	KIAA1731	IPI00400986.6	SGTIASKERTLSS	435	Kim	humans	KIAA1731
119	KIAA1731	IPI00400986.6	LQEQLTKQRDTLQ	1066	Kim	simians	KIAA1731
120	KIF20B	IPI00827503	TQNQRIKELINII	767	Wagner	apes	kinesin family member 20B
121	KIF23	IPI00873577	TYNTPLKVTSIAR	741	Wagner	catarrhines	kinesin family member 23
122	LAMB3	IPI00299404.1	GGTGSPKLVALRL	766	Kim	apes	laminin, beta 3
123	LAMP1	IPI00884105.2	ARDPAFKAANGSL	319	Kim	simians	lysosomal-associated membrane protein 1
124	LAPTM5	IPI00013827.1	MNSVEEKRNSKML	225	Kim	apes	lysosomal protein transmembrane 5

125	LDHA	IPI00217966.8	PDLGTDKDKEQWK	251	Kim	catarrhines	lactate dehydrogenase A
126	LMNB2	IPI00009771.6	EQVRLYKLELEQT	275	Kim, Wagner	primates	lamin B2
127	LPCAT2	IPI00016418	GIEEFAKYLKLPV	386	Wagner	catarrhines	lysophosphatidylcholine acyltransferase 2
128	LRPPRC	IPI00783271	YFHQLEKMNVKIP	613	Wagner	apes	leucine-rich PPR-motif containing
129	LTN1	IPI00783835	KWNSLLKIIEKAC	763	Wagner	simians	listerin E3 ubiquitin protein ligase 1
130	MACF1	IPI00432363	RAWVGNKNLILNS	1941	Wagner	African great apes	microtubule-actin crosslinking factor 1
131	MAP1S	IPI00296485.6	RGPVPAKPTVLFE	380	Kim	simians	microtubule-associated protein 1S
132	MARC2	IPI00329552	CGNEAAKWFTNFL	166	Wagner	apes	mitochondrial amidoxime reducing component 2
133	MARS	IPI00008240.2	IFQQLLKRGFVLQ	375	Kim, Wagner	apes	methionyl-tRNA synthetase
134	MCM3	IPI00013214	IFDQLAKSLAPSI	346	Wagner	African great apes	minichromosome maintenance complex component 3
135	MCM5	IPI00018350	SEHSIIKDFTKQK	696	Wagner	simians	minichromosome maintenance complex component 5
136	MCM7	IPI00299904.3	NARRYAKLFADAV	75	Kim, Wagner	simians	minichromosome maintenance complex component 7
137	MDH2	IPI00291006.2	LSHIETKAAVKGY	74	Kim	simians	malate dehydrogenase 2, NAD (mitochondrial)
138	MET	IPI00029273.1	RRLKETKDGFMFL	223	Kim	haplorhines	met proto-oncogene (hepatocyte growth factor receptor)
139	METTL10	IPI00411752.3	VAARSDKGSPGED	19	Kim	catarrhines	methyltransferase like 10
140	METTL15	IPI00783001	VRQQVMKTSQLGS	352	Wagner	primates	methyltransferase like 15
141	MFN2	IPI00642329.2	HALHQDKQLHAGS	171	Kim	simians	mitofusin 2
142	MRPL37	IPI00162330.3	VGPVGFKPETFRK	407	Kim	simians	mitochondrial ribosomal protein L37
143	MRPL45	IPI00790292	LVIPPEKSDRSIH	94	Wagner	catarrhines	mitochondrial ribosomal protein L45
144	MRPS11	IPI00010244	AAPSHTKFSIYPP	59	Wagner	apes	mitochondrial ribosomal protein S11
145	MRPS9	IPI00641924.2	QGMAFSKSEGKRK	271	Kim	simians	mitochondrial ribosomal protein S9
146	MSTO1	IPI00306049.6	SVLKEPKYQEELE	206	Kim	simians	misato homolog 1 (Drosophila)
147	MTO1	IPI00180380.2	VDEIVLKNLHLNS	200	Kim, Wagner	simians	mitochondrial translation optimization 1 homolog (S. cerevisiae)
148	MUM1	IPI00922531	YQEVGAKVLQRTN	643	Wagner	simians	melanoma associated antigen (mutated) 1
149	MX1	IPI00167949.6	PADIGYKIKTLIK	197	Kim	simians	myxovirus (influenza virus) resistance 1, interferon-inducible protein p78 (mouse)
150	MYO6	IPI00844172.1	AQLARQKEEESQQ	993	Kim	humans	myosin VI
151	N4BP2	IPI00328825.2	EDEKEMKEILMAG	1339	Kim	apes	NEDD4 binding protein 2
152	NACC1	IPI00045207.2	PKVKVLKAEDDAY	483	Kim	primates	nucleus accumbens associated 1, BEN and BTB (POZ) domain containing
153	NAGLU	IPI00008787.3	ERALAAKPGLDTY	59	Kim, Wagner	catarrhines	N-acetylglucosaminidase, alpha
154	NBN	IPI00299463.1	FRSLVIKNSTSRN	665	Kim	primates	nibrin
155	NBR1	IPI00299920.5	ERGAEGKPGVEAG	435	Kim	humans	neighbor of BRCA1 gene 1
156	NCAPD2	IPI00299524.1	RGLDGIKELEIGQ	1301	Kim, Wagner	humans	non-SMC condensin I complex, subunit D2
157	NDUFB6	IPI00219385	NKFLENKSPWRKM	54	Wagner	simians	NADH dehydrogenase (ubiquinone) 1 beta subcomplex, 6, 17kDa
158	NDUFB9	IPI00255052.5	KATQLLKEAEEEF	65	Kim	simians	NADH dehydrogenase (ubiquinone) 1 beta subcomplex, 9, 22kDa
159	NGDN	IPI00000162.5	AVTAQVKSLTQKV	33	Kim	African great apes	neuroguidin, EIF4E binding protein
160	NGLY1	IPI00074605.2	NKSHKVKSSQQPA	123	Kim	great apes	N-glycanase 1
161	NIT1	IPI00023779.1	SEPLGGKLLEEYT	108	Kim	catarrhines	nitrilase 1
162	NOA1	IPI00385928	PQPTREKQLQELQ	89	Wagner	apes	nitric oxide associated 1
163	NOL11	IPI00303813.5	KNQSLVKSLLLKA	247	Kim	African great apes	nucleolar protein 11
164	NRM	IPI00217557	YWEPIPKGPVLWE	121	Wagner	African great apes	nurim (nuclear envelope membrane protein)
165	NSFL1C	IPI00100197.3	AYVAGEKRQHSSQ	172	Kim	catarrhines	NSFL1 (p97) cofactor (p47)
166	NSMCE1	IPI00184884	WPHEIPKVFDPEK	241	Wagner	great apes	non-SMC element 1 homolog (S. cerevisiae)

167	NUDT22	IPI00031645.4	IWETRLKAQPWLF	57	Kim	simians	nudix (nucleoside diphosphate linked moiety X)-type motif 22
168	NUMA1	IPI00872028	ERDAALKQLEALE	610	Wagner	great apes	nuclear mitotic apparatus protein 1
169	NUP205	IPI00783781	YKDIWHKVGNALW	23	Wagner	catarrhines	nucleoporin 205kDa
170	NUP205	IPI00783781	AVHLLDKILKKHK	41	Wagner	simians	nucleoporin 205kDa
171	NUP205	IPI00783781.1	LPLLTEKQYIATI	304	Kim, Wagner	simians	nucleoporin 205kDa
172	NUP205	IPI00783781.1	DNVEGDKVSKKDE	1742	Kim	simians	nucleoporin 205kDa
173	PARP10	IPI00064457	NATVYGKGVYFAR	928	Wagner	simians	poly (ADP-ribose) polymerase family, member 10
174	PCM1	IPI00001654.5	AEKPRNKKLPEEE	1236	Kim	simians	pericentriolar material 1
175	PDCD5	IPI00023640.3	GLIEILKKVSQQT	97	Kim, Wagner	simians	programmed cell death 5
176	PEX1	IPI00411291	SISTREKLVLTTL	806	Wagner	catarrhines	peroxisomal biogenesis factor 1
177	PFKP	IPI00009790.1	RNGQIDKEAVQKY	156	Kim	simians	phosphofructokinase, platelet
178	PGAM5	IPI00063242.5	EEELASKLDHYKA	88	Kim, Wagner	simians	phosphoglycerate mutase family member 5
179	PIK3C2A	IPI00002580.2	VSSLLAKDPWDAV	301	Kim	simians	phosphoinositide-3-kinase, class 2, alpha polypeptide
180	PLD4	IPI00060310	WVLGVPKAVLPKT	278	Wagner	catarrhines	phospholipase D family, member 4
181	PLRG1	IPI00002624.1	ALPLQTKADANRT	135	Kim	simians	pleiotropic regulator 1
182	PML	IPI00022348.2	SCITQGKDAAVSK	394	Kim, Wagner	great apes	promyelocytic leukemia
183	PML	IPI00022348.2	KDAAVSKKASPEA	400	Kim	simians	promyelocytic leukemia
184	PML	IPI00022348.2	DAAVSKKASPEAA	401	Kim	catarrhines	promyelocytic leukemia
185	PMPCB	IPI00927892	AAVGPIKQLPDFK	472	Wagner	African great apes	peptidase (mitochondrial processing) beta
186	PNMA1	IPI00005685.2	SRDAQIKFLNTYQ	249	Kim, Wagner	simians	paraneoplastic antigen MA1
187	POLI	IPI00296840	VLSFFSKKQMQDI	549	Wagner	catarrhines	polymerase (DNA directed) iota
188	PRCP	IPI00001593.1	HLDLRTKNALDPM	462	Kim	simians	prolylcarboxypeptidase (angiotensinase C)
189	PRKDC	IPI00296337.2	LLTFIDKAMHGEL	3067	Kim	simians	protein kinase, DNA-activated, catalytic polypeptide
190	PSAT1	IPI00001734	LAAFMKKFLEMHQ	408	Wagner	apes	phosphoserine aminotransferase 1
191	PTGR1	IPI00292657.3	MGQQVAKVVESKN	70	Kim	apes	prostaglandin reductase 1
192	PTPN1	IPI00297261.3	CPIKEEKGSPLNA	350	Kim	apes	protein tyrosine phosphatase, non-receptor type 1
193	PTRH2	IPI00032903.3	PKSKTSKTHTDTE	47	Kim, Wagner	primates	peptidyl-tRNA hydrolase 2
194	PYGB	IPI00004358.4	DHLPELKQAVDQI	740	Kim	primates	phosphorylase, glycogen; brain
195	RABGGTB	IPI00295849.3	KVVEYVKGLQKED	124	Kim	simians	Rab geranylgeranyltransferase, beta subunit
196	RAD18	IPI00024579.1	ALESPAKSPASSS	102	Kim, Wagner	simians	RAD18 homolog (S. cerevisiae)
197	RAD18	IPI00024579.1	PDPSEAKRPEPPS	186	Kim, Wagner	catarrhines	RAD18 homolog (S. cerevisiae)
198	RAD18	IPI00024579.1	KYRKKHKSEFQLL	347	Kim	primates	RAD18 homolog (S. cerevisiae)
199	RARS2	IPI00873116	LLLSDYKFSWDRV	446	Wagner	simians	arginyl-tRNA synthetase 2, mitochondrial
200	RBCK1	IPI00783058	LLEREIKALLTPE	342	Wagner	simians	RanBP-type and C3HC4-type zinc finger containing 1
201	RDX	IPI00219365.3	ELMERLKQIEEQT	352	Kim	simians	radixin
202	RECQL4	IPI00014925.1	LTLLQGKRFQNLD	695	Kim	simians	RecQ protein-like 4
203	RGL2	IPI00005656.1	TGYAAGKGVGGGS	198	Kim	simians	ral guanine nucleotide dissociation stimulator-like 2
204	RGL3	IPI00643373.5	GSAEAQKAEKLLE	187	Kim	catarrhines	ral guanine nucleotide dissociation stimulator-like 3
205	RHBDD2	IPI00010255.3	PSGLTLKSEALRN	51	Kim, Wagner	primates	rhomboid domain containing 2
206	RHBDD3	IPI00215831.2	TLVTHGKGGPAHS	374	Kim	primates	rhomboid domain containing 3
207	RIOK2	IPI00306406.4	ELFPTFKDIRRED	288	Kim	apes	RIO kinase 2 (yeast)
208	RNF25	IPI00059944	AMLDPPKPSRGPW	341	Wagner	simians	ring finger protein 25

209	RPF1	IPI00292221.3	FASYFNKQTSPKI	138	Kim	simians	ribosome production factor 1 homolog (S. cerevisiae)
210	RUSC1	IPI00425688.2	SLRGTSKEAASDP	711	Kim	simians	RUN and SH3 domain containing 1
211	SCAF11	IPI00746412.2	PQSGWMKQEEETS	1178	Kim	catarrhines	SR-related CTD-associated factor 11
212	SCARB1	IPI00177968.2	EIMWGYKDPLVNL	184	Kim	apes	scavenger receptor class B, member 1
213	SCFD2	IPI00141564.8	ARPDKRKLGSLGD	196	Kim	simians	sec1 family domain containing 2
214	SCO2	IPI00014458	GLTGSTKQVAQAS	196	Wagner	humans	SCO cytochrome oxidase deficient homolog 2 (yeast)
215	SDR42E1	IPI00163504.4	LNRNLIKEVNVRG	96	Kim	humans	short chain dehydrogenase/reductase family 42E, member 1
216	SDR42E1	IPI00163504.4	KKELGYKAQPFDL	337	Kim	haplorhines	short chain dehydrogenase/reductase family 42E, member 1
217	SEC11C	IPI00219436.3	DIFGDLKKMNKRQ	23	Kim	apes	SEC11 homolog C (S. cerevisiae)
218	SFXN4	IPI00412741	SSNLIPKLFRPAA	106	Wagner	catarrhines	sideroflexin 4
219	SFXN4	IPI00412741.1	RSLESIKGIAVMD	223	Kim	simians	sideroflexin 4
220	SHQ1	IPI00465265	NPWWTDKYSKMMA	222	Wagner	haplorhines	SHQ1 homolog (S. cerevisiae)
221	SKA3	IPI00789249.1	TPPEVTKIPEDIL	366	Kim	simians	spindle and kinetochore associated complex subunit 3
222	SKP2	IPI00013294.1	IVNTLAKNSNLVR	228	Kim	African great apes	S-phase kinase-associated protein 2 (p45)
223	SLC16A1	IPI00024650.1	SKASLEKAGKSGV	216	Kim	simians	solute carrier family 16, member 1 (monocarboxylic acid transporter 1)
224	SLC20A1	IPI00023035.2	VGDIENKHPVSEV	295	Kim	African great apes	solute carrier family 20 (phosphate transporter), member 1
225	SLC22A18	IPI00410347.5	ASTKGAKTDAQAP	210	Kim	catarrhines	solute carrier family 22, member 18
226	SLC25A3	IPI00022202.3	SASLVLKRLGFKG	304	Kim, Wagner	simians	solute carrier family 25 (mitochondrial carrier; phosphate carrier), member 3
227	SLC3A2	IPI00554702.1	RDIENLKDASSFL	323	Kim	simians	solute carrier family 3 (activators of dibasic and neutral amino acid transport), member 2
228	SLC43A3	IPI00301100.3	TPGAGQKQELRSF	277	Kim, Wagner	catarrhines	solute carrier family 43, member 3
229	SLC9A1	IPI00020060.2	LSRDPAKVAEEDE	750	Kim	simians	solute carrier family 9 (sodium/hydrogen exchanger), member 1
230	SLC9B2	IPI00386475.3	QEETVMKLKGIDA	36	Kim	apes	solute carrier family 9, subfamily B (cation proton antiporter 2), member 2
231	SLC9B2	IPI00386475.3	EGSILLKSSEKKL	53	Kim	haplorhines	solute carrier family 9, subfamily B (cation proton antiporter 2), member 2
232	SLFN5	IPI00433279.3	KQKLVNKGGYTGR	481	Kim	haplorhines	schlafen family member 5
233	SLIRP	IPI00009922	AASSQLKEHFAQF	36	Wagner	catarrhines	SRA stem-loop interacting RNA binding protein
234	SLX4	IPI00291796.2	SDPLEEKKALEIS	1179	Kim	humans	SLX4 structure-specific endonuclease subunit homolog (S. cerevisiae)
235	SNAP23	IPI00010438.2	TMLDEQKEQLNRI	49	Kim	apes	synaptosomal-associated protein, 23kDa
236	SNAP23	IPI00010438.2	AQNPQIKRITDKA	185	Kim	simians	synaptosomal-associated protein, 23kDa
237	SOD1	IPI00218733	ESNGPVKVWGSIK	31	Wagner	simians	superoxide dismutase 1, soluble
238	SPAG5	IPI00328118	NKLQHLKESHEMG	488	Wagner	simians	sperm associated antigen 5
239	SPC24	IPI00168317.1	TQDGAEKQLREIL	50	Kim, Wagner	apes	SPC24, NDC80 kinetochore complex component, homolog (S. cerevisiae)
240	SPG20	IPI00430622	GISISSKESEHTG	62	Wagner	apes	spastic paraplegia 20 (Troyer syndrome)
241	SPG20	IPI00430622	ASGTDVKQLDQGN	370	Wagner	simians	spastic paraplegia 20 (Troyer syndrome)
242	SSFA2	IPI00400812.5	NSSSFAKGIDIKV	212	Kim, Wagner	simians	sperm specific antigen 2
243	SSFA2	IPI00400812.5	SGIVESKLDSDFN	416	Kim	catarrhines	sperm specific antigen 2
244	ST14	IPI00001922.1	YNSRHEKVNGLEE	28	Kim	catarrhines	suppression of tumorigenicity 14 (colon carcinoma)
245	ST14	IPI00001922.1	AFPTDSKTVQRTQ	204	Kim	catarrhines	suppression of tumorigenicity 14 (colon carcinoma)
246	STOX1	IPI00065183.5	CRLEAQKAAGAAE	23	Kim	primates	storkhead box 1
247	STXBP2	IPI00910670.1	SVQALIKDFQGTP	89	Kim, Wagner	great apes	syntaxin binding protein 2
248	SUGP2	IPI00554436	TIDQLVKRVIEGS	595	Wagner	simians	SURP and G patch domain containing 2
249	TACC3	IPI00002135.1	DLDAVVKATQEEN	649	Kim	primates	transforming, acidic coiled-coil containing protein 3
250	TAF1C	IPI00246842.4	NPQFLGKPGRIQL	258	Kim	African great apes	TATA box binding protein (TBP)-associated factor, RNA polymerase I, C, 110kDa

251	TAP2	IPI00328112.4	LVLQEGKLQKLAQ	679	Kim	African great apes	transporter 2, ATP-binding cassette, sub-family B (MDR/TAP)
252	TBL2	IPI00000948.3	QDPYLLKTGRFEE	322	Kim	apes	transducin (beta)-like 2
253	TBL3	IPI00477971	KLWPLPKALLSKN	453	Wagner	simians	transducin (beta)-like 3
254	TDP2	IPI00009913.7	ETISEPKTYVDLT	112	Kim, Wagner	catarrhines	tyrosyl-DNA phosphodiesterase 2
255	THAP5	IPI00293093.5	ITLLELKEQQTLG	351	Kim	simians	THAP domain containing 5
256	TOM1L1	IPI00023186.5	EQNKNQKEATNTT	301	Kim	simians	target of myb1 (chicken)-like 1
257	TOR3A	IPI00301631.5	TWYCSFKDCCPRG	109	Kim	apes	torsin family 3, member A
258	TRADD	IPI00018744.1	DALRNLKCGSGAR	163	Kim	simians	TNFRSF1A-associated via death domain
259	TRMT6	IPI00099311	HGTFSAKMLSSEP	273	Wagner	humans	tRNA methyltransferase 6 homolog (S. cerevisiae)
260	TSR2	IPI00056314	HSQEKAKWLGGAV	44	Wagner	catarrhines	TSR2, 20S rRNA accumulation, homolog (S. cerevisiae)
261	TSTA3	IPI00014361.1	GSGLVGKAIQKVV	21	Kim	simians	tissue specific transplantation antigen P35B
262	TTC26	IPI00328172.1	LEELLSKRDFTGA	36	Kim	catarrhines	tetratricopeptide repeat domain 26
263	TTC4	IPI00000606	HEDQWEKEFEKVP	37	Wagner	apes	tetratricopeptide repeat domain 4
264	TUBGCP3	IPI00033516.1	KRIGEFKESIPKM	837	Kim	primates	tubulin, gamma complex associated protein 3
265	UACA	IPI00173359	LDNKLLKEQAHNL	730	Wagner	simians	uveal autoantigen with coiled-coil domains and ankyrin repeats
266	UIMC1	IPI00384342.3	DKEVGNKEDAEKE	485	Kim	primates	ubiquitin interaction motif containing 1
267	URB1	IPI00297241	EQKTEQKWVFGVL	1786	Wagner	African great apes	URB1 ribosome biogenesis 1 homolog (S. cerevisiae)
268	URB1	IPI00297241.3	VVADLLKDSAVRS	2141	Kim	catarrhines	URB1 ribosome biogenesis 1 homolog (S. cerevisiae)
269	URGCP	IPI00743955.4	DSDSFVKRIRAIV	386	Kim	primates	upregulator of cell proliferation
270	UTP6	IPI00020128.2	ECPKLYKEYFRME	180	Kim, Wagner	haplorhines	UTP6, small subunit (SSU) processome component, homolog (yeast)
271	VRK2	IPI00329275.7	TSPDIFKKSRSPS	443	Kim	simians	vaccinia related kinase 2
272	WBP5	IPI00008434.1	KPEEEEKLEEEAK	34	Kim	catarrhines	WW domain binding protein 5
273	WDR35	IPI00165984.4	IEKVGIKDASQFI	684	Kim	catarrhines	WD repeat domain 35
274	WDR67	IPI00061009.4	VEDMQAKVDQQRV	715	Kim	apes	WD repeat domain 67
275	WDR73	IPI00180704	LSVKENKGLFPER	66	Wagner	catarrhines	WD repeat domain 73
276	XAB2	IPI00163084.3	GNEDTIKEMLRIR	721	Kim	simians	XPA binding protein 2
277	XRCC5	IPI00220834.8	FSESLRKLCVFKK	233	Kim	simians	X-ray repair complementing defective repair in Chinese hamster cells 5 (double-strand-break rejoining)
278	XRCC5	IPI00220834.8	WTVVDAKTLKKED	282	Kim, Wagner	simians	X-ray repair complementing defective repair in Chinese hamster cells 5 (double-strand-break rejoining)
279	XRCC5	IPI00220834.8	LAKKDEKTDTLED	469	Kim	simians	X-ray repair complementing defective repair in Chinese hamster cells 5 (double-strand-break rejoining)
280	ZNF451	IPI00384690.2	LFGQDVKAVVAED	490	Kim	primates	zinc finger protein 451
281	ZWINT	IPI00294008.4	IKIGLTKALTQME	116	Kim, Wagner	simians	ZW10 interactor
