# Supplementary material for: Gains of ubiquitylation sites in highly conserved proteins in the human lineage
Source: BMC Bioinformatics. 2012 Nov 17;13:306. doi: 10.1186/1471-2105-13-306 (PMC3561281; doi:10.1186/1471-2105-13-306)
Supplement: Additional file 2 — Detailed alignments of surrounding regions of novel ubiquitylation sites. [file 1471-2105-13-306-S2.html]

```
# NO, number
# GN, gene symbol
# IP, IPI accession number
# PE, human peptide sequence with the modified lysine at the center
# MP, modified position
# EX, experiment, [Kim, Kim et al (2011); Wagner, Wagner et al, (2011)]
# CL, clade sharing the novel lysine residue
# DE, description
# SQ, a part of the aligned sequences
#  pri, Primates
#  eua, Euarchontoglires
#  lau, Laurasiatheria
#  afr, Afrotheria
#  xen, Xenarthra
#  mar, Marsupialia
#  pro, Prototheria
#  -, alignment gap
#  X, unidentified amino acid
#  *, stop codon
#  In parentheses are the lysine residues ubiquitinated in human and the aligned amino acids of the other species.

NO 1
GN ABCB1
IP IPI00027481
PE YDPLAGKVLLDGK
MP 1093
EX Wagner
CL simians
DE ATP-binding cassette, sub-family B (MDR/TAP), member 1
SQ
pri Human        GCGKSTVVQLLERFYDPLAG(K)VLLDGKEIKRLNVQWLRAHL
pri Chimpanzee   GCGKSTVVQLLERFYDPLAG(K)VLLDGKEIKRLNVQWLRAHL
pri Gorilla      GCGKSTVVQLLERFYDPLAG(K)VLLDGKEIKRLNVQWLRAHL
pri Orangutan    GCGKSTVVQLLERFYDPLAG(K)VLLDGKEIKRLNVQWLRAHL
pri Gibbon       GCGKSTVVQLLERFYDPLAG(K)VLLDGKEIKQLNVQWLRAHL
pri Rhesus       GCGKSTVVQLLERFYDPLAG(K)VLLDGKEIKQLNVQWLRAHL
pri Baboon       GCGKSTVVQLLERFYDPLAG(K)VLLDGKEIKQLNVQWLRAHL
pri Marmoset     GCGKSTVVQLLERFYDPLAG(K)VLLDGKEIKQLNVQWLRAHL
pri Tarsier      GCGKSTVVQLLERFYDPLAG(T)VLIDGEEIKQLNVQWLRAHL
pri Bushbaby     GCGKSTVVQLLERFYDPLAG(T)VLIDNKEIKQLNVQWVRAHM
pri Mouse_lemur  GCGKSTVVQLLERFYDPLAG(T)VLIDNKEIKQLNVQWLRAHL
eua Treeshrew    GCGKSTVVQLIERFYDPIAG(T)VKIDGEEIKNLNVQWLRAQL
eua Mouse        GCGKSTVVQLLERFYDPMAG(S)VFLDGKEIKQLNVQWLRAHL
eua Rat          GCGKSTVVQLLERFYNPMAG(T)VFLDGKEIKQLNVQWLRAHL
eua Kangaroo_rat GCGKSTVVQLLERFYDPLAG(T)VLLDGKEIKQLNVQWLRAHL
eua Guinea_pig   GCGKSTTVQLLERFYNPISG(T)VFVDGKEIQQLNVQWLRAQL
eua Squirrel     GCGKSTVVQLLERFYDPLAG(T)VLLDGQEAKKLNVQWLRAQL
eua Rabbit       GCGKSTVVQLIERFYDPLAG(T)VLLDGKEVNQLNVQWLRAHL
lau Dog          GCGKSTVVQLLERFYDPLAG(S)VLIDGKEIKHLNVQWLRAHL
lau Cat          GCGKSTVVQLLERFYDPMAG(T)VLIDGKEIKHLNVQWLRAHM
lau Horse        GCGKSTLVQLLERFYDPMAG(T)VLLDGTEIKHLNVQWLRAHL
lau Cow          CCGKGTVIQLLERFYDPLAG(T)VLIDGKEIKQLNVQWLRAHM
lau Dolphin      GCGKSTVVQLLERFYDPLAG(T)VLIDGKEIKHLNVQWLRAHM
lau Microbat     GCGKSTVVQLLERFYDPLAG(T)VRIDSKEIKQLNVQWLRAQL
lau Hedgehog     GCGKSTVVQLLERFYDPLSG(T)VLIDGKEIQHLNVQWLRAHL
lau Shrew        GCGKSTVVQLLERFYDPLSG(T)VLIDGEEIKHLNVQWLRAHL
afr Elephant     GCGKSTVVQLIERFYDPLAG(K)VLIDGQEIKHLNVQWLRAHL
afr Tenrec       GCGKSTVVQLLERFYDPLAG(R)VLIDDKEIKHLNVQWLRAHL
xen Sloth        GCGKSTVVQLLERFYDPMGG(T)VLIDGKDIKQLNVQWLRAHL
mar Opossum      GCGKSTVVQLLERFYDPLGG(K)VIVDDKDVKTLNVKWLRAQL
mar Wallaby      GCGKSTTVQLLERFYDPLAG(Q)VLLDGQETKKLNVQWLRAQL
pro Platypus     GCGKSTVVQLLERFYDPLAG(S)LLLDGQDARQLNVQWLRGQI

NO 2
GN ABCC3
IP IPI00006674.1
PE RILEAAKGEIRID
MP 1344
EX Kim
CL simians
DE ATP-binding cassette, sub-family C (CFTR/MRP), member 3
SQ
pri Human        RTGAGKSSMTLCLFRILEAA(K)GEIRIDGLNVADIGLHDLRS
pri Chimpanzee   XXXXXXXXXXXXXXXILEAA(K)GEIRIDGLNVADIGLHDLRY
pri Gorilla      RTGAGKSSMTLCLFRILEAA(K)GEIRIDGLNVADIGLHDLRS
pri Orangutan    RTGAGKSSMTLCLFRILEAA(K)GEIRIDGLNVADIGLHDLRS
pri Gibbon       RTGAGKSSMTLCLFRILEAA(K)GEIRIDGLNVADIGLHDLRS
pri Rhesus       RTGAGKSSMTLCLFRILEAA(K)GEILIDGLNVADIGLHDLRS
pri Baboon       RTGAGKSSMTLCLFRILEAA(K)GEILIDGLNVADIGLHDLRS
pri Marmoset     RTGAGKSSMTLCLFRILEAA(K)GKILIDGLNVADIGLHDLRS
pri Tarsier      RTGAGKSSMTLCLFRILEAA(E)GEILIDGLNVADIGLHDLRS
pri Bushbaby     RTGAGKSFMTLCLPRILEAA(E)GEIRIDGLNVADIGLHDLRS
pri Mouse_lemur  RTGAGKSSMTLSLFRILEAA(E)GEIRIDGLNVADIGLHDLRS
eua Treeshrew    RTGAGKSSMTLCLFRILEAA(E)GEIRIDGLNVADIGLHDLRS
eua Mouse        RTGAGKSSMTLCLFRILEAA(E)GEIVIDGLNVAHIGLHDLRS
eua Rat          RTGAGKSSMTLCLFRILEAA(E)GEIFIDGLNVAHIGLHDLRS
eua Kangaroo_rat RTGAGKSSMTLSLFRILEAA(E)GEILIDGLNVANIGLHDLRS
eua Guinea_pig   RTGAGKSSMTLCLFRILEAA(E)GEIFIDGLNVANIGLHDLRS
eua Rabbit       RTGAGKSSMTLCLFRILEAA(E)GEIRIDGLNVADIGLHDLRS
eua Pika         RTGAGKSSMTLCLFRILEAA(E)GEIRIDGLNVANMGLHDLRS
lau Dog          RTGAGKSSMTLCLFRILEAA(E)GEIRIDGLNVADIGLHDLRS
lau Cat          RTGAGKSSMTLCPFRILEAA(E)GEIRIDGLNVADMGLHDLRP
lau Horse        RTGAGKSSMTLSLFRILEAA(E)GEIRIDGLNVADIGLHDLRS
lau Cow          RTGAGKSSMTLCLFRILEAA(E)GEIYIDGLNVADIGLHDLRS
lau Dolphin      RTGAGKSSMTLCLFRILEAT(E)GDILIDGLNVADIGLHDLRS
lau Megabat      RTGAGKSSMTLCLFRILEAA(E)GEIRIDDLNVAAIGLHDLRS
lau Microbat     RTGAGKSSMTLSLFRLLEAA(Q)GEIRIDGVNVADMGLHDLRS
lau Hedgehog     RTGAGKSSMTLCLFRILEAA(E)GEIRIDGLNVANLGLHDLRS
afr Elephant     RTGAGKSSMTLCLFRILEAA(E)GEILIDSLNVADIGLHDLRS
afr Rock_hyrax   RTGAGKSSMTLCLFRILEAA(E)GEICIDGLNVADIGLHDLRS
afr Tenrec       RTGAGNSSMTLCLFRILEAA(G)GQIGIDGLNVADMGLHDLRF
mar Opossum      RTGAGKSSMTLCLFRILEAA(K)GEIHIDGVNIATIGLHDLRS
pro Platypus     RTGAGKSSMTLCLFRILEAA(A)GEIRIDGIKISDIGLHDLRS

NO 3
GN ADAM15
IP IPI00013302.1
PE DLPISLKKVLQTS
MP 58
EX Kim
CL catarrhines
DE ADAM metallopeptidase domain 15
SQ
pri Human        KAPREPLEPQVLQDDLPISL(K)KVLQTSLPEPLRIKLELDGD
pri Chimpanzee   KAPREPLEPQVLQDDLPISL(K)KVLQTSLPEPLRIKLELDGD
pri Gorilla      KAPREPLEPQVLQDDLPISL(K)KVLQTSLPEPLRIKLELDGD
pri Orangutan    KAPREPLEPQVLQD-----L(K)KVLQTSLPEPLRIKLELDGD
pri Gibbon       KALREPLEPQVLQDDLPISL(K)KVLQTSLPEPLRIKLELDGD
pri Rhesus       KAPRGPLEPQVLQDDLPISL(K)KVLQTSLPEPLRIKLELDGD
pri Baboon       KAPRGPLEPQVLQDDLPISL(K)KVLXTSLPEPLRIKLELDGD
pri Marmoset     KSPSGPLEPQVLQDDLPNSL(E)EVLQSSLPEPLRIKLELDGD
pri Bushbaby     RALSGTLEPQVLQDNLLISL(P)EMLQTSLPESLRIILELDGE
pri Mouse_lemur  SALNGPLEPQVLQDNLPINL(A)EVLQTSLPESLRIKLELDGE
eua Treeshrew    RALSGSSEPQVLQDDLPVNL(A)EALQTSQPEVLRIKLELDGE
eua Mouse        RTLSGSMESRVVQDSPPMSL(A)DVLQTGLPEALRISLELDSE
eua Rat          RTQSRSLENQVVQDSPPINL(T)EVLQTGLPETLRIGLELDGE
eua Guinea_pig   RTLSGPLEPQVLQDHPLFSL(A)EMLQTSLPEALKVKLELDGE
eua Rabbit       KLWSGSLEPQILQDDRPLSL(A)EVLHSSLPEALRIQLELDGE
lau Dog          RALGGPSEPQILRDHPMLSL(A)EMLQTNLPEAFRIKLELDGD
lau Cow          RAQNGPLEPQILQDNPTLSL(A)EALQTSLPEALRIKLELDGE
lau Dolphin      RALNGSSEPQILQDNLTLSL(A)EVLQTSLPEALRIKLELDGE
lau Megabat      RALSGASEAQALQDGLTLSL(T)EVLQTNVPETLRIKVELDGE
lau Microbat     KTPSGPWEPQILQDNLTLSL(A)EVLQTSLPETLRIKLELDGE
afr Elephant     RVLSGPLEPQILQDYLMLSL(A)EVVQTSLPEALQIKLELDGE
afr Rock_hyrax   KTLSGPLEPQILQDNLTLSL(A)EVLQTGL-EALRIKLELDGE
mar Opossum      ELPRRYPEPQILQGNLTLNL(S)EALQDSVPEKLLIKLELDGK
mar Wallaby      GLPRGYPEPQVFQGNLTLNL(S)EALQDRLPEKLQISLELEGK

NO 4
GN ADAM15
IP IPI00013302.1
PE LPISLKKVLQTSL
MP 59
EX Kim
CL catarrhines
DE ADAM metallopeptidase domain 15
SQ
pri Human        APREPLEPQVLQDDLPISLK(K)VLQTSLPEPLRIKLELDGDS
pri Chimpanzee   APREPLEPQVLQDDLPISLK(K)VLQTSLPEPLRIKLELDGDS
pri Gorilla      APREPLEPQVLQDDLPISLK(K)VLQTSLPEPLRIKLELDGDS
pri Orangutan    APREPLEPQVLQD-----LK(K)VLQTSLPEPLRIKLELDGDS
pri Gibbon       ALREPLEPQVLQDDLPISLK(K)VLQTSLPEPLRIKLELDGDS
pri Rhesus       APRGPLEPQVLQDDLPISLK(K)VLQTSLPEPLRIKLELDGDS
pri Baboon       APRGPLEPQVLQDDLPISLK(K)VLXTSLPEPLRIKLELDGDS
pri Marmoset     SPSGPLEPQVLQDDLPNSLE(E)VLQSSLPEPLRIKLELDGDS
pri Bushbaby     ALSGTLEPQVLQDNLLISLP(E)MLQTSLPESLRIILELDGES
pri Mouse_lemur  ALNGPLEPQVLQDNLPINLA(E)VLQTSLPESLRIKLELDGES
eua Treeshrew    ALSGSSEPQVLQDDLPVNLA(E)ALQTSQPEVLRIKLELDGES
eua Mouse        TLSGSMESRVVQDSPPMSLA(D)VLQTGLPEALRISLELDSES
eua Rat          TQSRSLENQVVQDSPPINLT(E)VLQTGLPETLRIGLELDGEN
eua Guinea_pig   TLSGPLEPQVLQDHPLFSLA(E)MLQTSLPEALKVKLELDGES
eua Rabbit       LWSGSLEPQILQDDRPLSLA(E)VLHSSLPEALRIQLELDGES
lau Dog          ALGGPSEPQILRDHPMLSLA(E)MLQTNLPEAFRIKLELDGDS
lau Cow          AQNGPLEPQILQDNPTLSLA(E)ALQTSLPEALRIKLELDGES
lau Dolphin      ALNGSSEPQILQDNLTLSLA(E)VLQTSLPEALRIKLELDGES
lau Megabat      ALSGASEAQALQDGLTLSLT(E)VLQTNVPETLRIKVELDGES
lau Microbat     TPSGPWEPQILQDNLTLSLA(E)VLQTSLPETLRIKLELDGES
afr Elephant     VLSGPLEPQILQDYLMLSLA(E)VVQTSLPEALQIKLELDGES
afr Rock_hyrax   TLSGPLEPQILQDNLTLSLA(E)VLQTGL-EALRIKLELDGES
mar Opossum      LPRRYPEPQILQGNLTLNLS(E)ALQDSVPEKLLIKLELDGKS
mar Wallaby      LPRGYPEPQVFQGNLTLNLS(E)ALQDRLPEKLQISLELEGKS

NO 5
GN ADNP2
IP IPI00402209.3
PE TEGPIVKDEALQI
MP 1032
EX Kim
CL simians
DE ADNP homeobox 2
SQ
pri Human        TSVVPFKRQRNESRTEGPIV(K)DEALQILALDPKKYEGRSYE
pri Chimpanzee   TSVVPFKRQRNESRTEGPIV(K)DEALQILALDPKKYEGRSYE
pri Gorilla      TSVVPFKRQRNESRTEGPIV(K)DEALQILALDPKKYEGRSYE
pri Orangutan    TSVVPFKRQRNESRTEGPIV(K)DEALQILALDPKKYEGRSYE
pri Gibbon       TSVVPFKRQRNESRTEGPVV(K)DEALQILALDPKKYEGRSYE
pri Rhesus       TSVVPFKRQRNESRTEGPIV(K)DEALQILALDPKKYEGRSYE
pri Baboon       TSVVPFKRQRNESRTEGPIV(K)DEALQILALDPKKYEGRSYE
pri Marmoset     TSVVPFKRQRNESRTEGPIV(K)DDALQILALDPKKYEGRSYE
pri Tarsier      MSAVPFKRQRNESRTEGPIV(N)EDALQILALDPKKYEDRXXX
pri Bushbaby     ASVVPFKRQRNETRTEGLVA(S)DDALQILALDPKKYEDRSYE
pri Mouse_lemur  TSVVPCKRQRNETRTEGPVV(S)EDALQILALDPKKYEDRSYE
eua Mouse        LGAVPLKRQKSEIRTEGSGP(S)EDSLQALALDPSKYEGRSYE
eua Rat          LSAVPLKRQKNESRTEGSGA(S)DDSLQVLALDPSKYGSRSYE
eua Kangaroo_rat VSAVPLKRQRNESRTEGQVA(N)DDALQVLALDPKKYEGRSYE
eua Guinea_pig   TSSVPLKRQRNESRTEGLVA(N)DDALQILVLDPKKYEGRSYE
eua Rabbit       ASVVPSKRQRNESRTEVPAV(G)DDALQILALDPKRYEDRSYE
eua Pika         SSAVPCKRQRNESRTEGQTA(S)EDVLQILALDPKRYEDRSYE
lau Dog          ASVVPFKRQRNEIRTEGPLV(N)DDALQILALNPKKYEDRSYE
lau Horse        TSVVPFKRQRNESRTEGPLV(N)DDALQILALNPKKYEDRSYE
lau Cow          ASAAPVKRQRSEGRTEGPPV(S)DDALQILALNPKKYEDRSYE
lau Dolphin      TSAVPSKRQRSESRTEAPLV(H)DDALQVLALNPKRYEDRSYE
lau Alpaca       TSVVPCKRQRNESRTEGPLV(S)DDALQILALDPKKYEDRSYE
lau Megabat      TSVMPFKRQRSESRTEGPLV(S)DDTLQILALNPKKYEDRSYE
lau Microbat     ISVVPLKRQRNESRTEGPLV(N)DDALQILALNPKKYEDRSYE
lau Shrew        GSAVPCKRQRNESRTEGSLG(S)DDPLQILALNPKKYEDRTYE
afr Elephant     ASAMPFKRQKNEGRTDGLLI(N)DDALQILALNPRKYEDRSYE
xen Armadillo    ATAVPVKRQKNDSRTEGVLI(N)DDALQILALNPKKYEDRSYE
xen Sloth        GNAVPFKRHKNDNRAEGVLA(N)DDTLQILALNPKKLEDRSYE
mar Opossum      VN--PSKKQKNENKTEGPLI(N)DDALHILALNPQKYEDRSYE
mar Wallaby      VN--PSKKQKNEIRTDGPVI(N)DDALHILALNPQKYEDRSYE

NO 6
GN AEN
IP IPI00074083.5
PE RRPAPGKASGPLP
MP 101
EX Kim
CL apes
DE apoptosis enhancing nuclease
SQ
pri Human        QCLRAGSGSAP-CSRRPAPG(K)ASGPLPSKCVAIDCEMVGTG
pri Chimpanzee   QCLRAGSGGAP-CSRRPAPG(K)ASGPLPSKCVAIDCEMVGTG
pri Gorilla      QCLRAGSGSAP-CSRRPAPG(K)ASGPLPSKCVAIDCEMVGTG
pri Orangutan    QCPRAGSGGAP-CSRRPAPG(K)ASGPLPSKCVAIDCEMVGTG
pri Gibbon       QCLRAGSGGVP-CSRRPAPG(K)ASGPLPSKCVAIDCEMVGTG
pri Rhesus       QCLRAGSGSAP-CSRRPAPR(R)ASGPLPSKCVAIDCEMVGTG
pri Baboon       QCLRAGSGGAP-CSRRPAPS(R)ASGPLPSKCVAIDCEMVGTG
pri Tarsier      QRPKAGSGGTS-CSRRPTPR(E)ASGPLPSKCVAIDCEMVGTG
pri Bushbaby     QLPRACSGSSQ-CSRIPAPR(E)SFGHFPSKYVAIGCEMVAQG
pri Mouse_lemur  QHPRAGSGSAP-GSRRPAPT(E)VSGPSPSKYVAIDCEMVGTG
eua Treeshrew    QCPRAEPGSTP-CSRRLPTR(E)ALGPSPSKCVAIDCEMVGTG
eua Mouse        QRPKARSGSNGLCSKKSVPR(E)APRPGPIKCVAIDCEMVGTG
eua Rat          QRTKARSGSKGLCSKRPVPR(E)APSSGPSKYVAIDCEMVGTG
eua Guinea_pig   QHSRAGSGGTGLGSRRPAPG(E)ALRPSPSKCVAIDCEMVGTG
eua Squirrel     QHPRAEFGSGGPYSKRLTSR(E)ASGPLPSKCVAIDCEMVGTG
lau Dog          QRLRNEPGGAS-WSRKPTPR(E)SAGPRPSKCVAIDCEMVGTG
lau Cat          QRPRNESGGAS-CSRKPTPR(E)STAPWPSKCVAIDCEMVGTG
lau Horse        QRPRTESGGVP-CSRKPAPW(E)SAGPLPSKCVAIDCEMVGTG
lau Cow          QRPRAEPGGAG-CSRKPAPR(D)SAGPLPSKCVAIDCEMVGTG
lau Dolphin      QRPRAESGGAQ-GSRKPTPR(E)SAGPLPSKCVAIDCEMVGTG
lau Megabat      QRPRAGPGGAP-CSRKPTPR(V)CTGPLPSKCVAIDCEMVGTG
afr Elephant     PRPRAESGSTS-CSREPAPK(S)VAWPLPSKFVAIDCEMVGTG
afr Rock_hyrax   PRPWSDSGSTL-CSRKPAPT(S)VTWPLPGKVVAIDCEMVGTG

NO 7
GN AHCTF1
IP IPI00170594.5
PE GEVWASKEPINST
MP 1078
EX Kim
CL simians
DE AT hook containing transcription factor 1
SQ
pri Human        LTRSVFINNVLSKIGEVWAS(K)EPINSTTPFNSSKIEEPSPI
pri Chimpanzee   LTRSVFINNVLSKIGEVWAS(K)EPINSTTSFNSSKIEEPSPI
pri Gorilla      LTRSVFINNVLSKIGEVWAS(K)EPINSTTPFNSSKIEEPSPI
pri Orangutan    LTRSVFINNVLSKIGEVWAS(K)EPINSTTSYNSSKIEEPSPI
pri Gibbon       LTRSVFINNVLSKIGEVWAS(K)EPINSTTPYNSSKIEEPSPI
pri Rhesus       LTRSVFINNVLSKIGEVWAS(K)EPISSTTPYNSSKIEEPSPI
pri Baboon       LTRSVFINNVLSKIGEVWAS(K)EPISSTTPYNSSKIEEPSPI
pri Marmoset     LTRSVFINNVLSKIGEVWAS(K)EPINSITPSISSKIEEPSPI
pri Tarsier      LTRSVFINNVLSKIGDVSAS(S)EPKNSDLLYKSPKIEEPS-I
eua Mouse        LTRSTFISNVLSKIGEVWAS(H)EPRNGVSLFNSPKTEQPSPV
eua Rat          ITRSTFISNVLSKIGEVWAS(H)EPRNGISLFNSPKTEQPSPV
eua Kangaroo_rat LTRSTFISNVLSKIGEVWAS(H)EPRNSPLLHSSPKTEQASPG
eua Guinea_pig   ITKSTFISNVLSKIGEVWAN(S)ESTSHVSLYNSSKIEETSPI
eua Rabbit       LTRSSFVNNVLSKIGEVWTS(S)EPKNSILPYNSPKIEEPSPI
eua Pika         LTRSSFINNVLSKIGEVWTS(S)EPKNSILTYISPKTEEPAPI
lau Dog          LTRSAFISNVLSKIGEIWAC(T)GPKGGSSAYKSPRRE-PSP-
lau Cat          LTRSTFISSVLSKIGEVWAN(S)DPKDGTSACSSPKTEGPSP-
lau Horse        LTRSTFINNVLSKIGEVWAS(N)EPKNSISAYSSSKIEDPCPI
lau Cow          LTRSTFINNVLSKIGEVWAS(N)EPENSISVYNSPKVEEPSPV
lau Alpaca       LTRSTFINNVLSKIGEVWAS(S)EHKNSISIYNSPKIEEPSPV
lau Megabat      LTRSTFISNVLSRIGEVWTS(S)EPKNNILTYNSPRVEVPSPI
lau Microbat     LTRSTFIHNVLSKVGEVWKS(D)EPKNSTLAYNSPRIEGPSPV
lau Hedgehog     ISRSTFISNVLSKVGEVWAS(N)EPKPSVSLYNGPQIQEPSPI
afr Elephant     LTRSAFINNVLSKIGEVWAS(S)EPKNSTSPYISPNIEEPSPI
afr Rock_hyrax   LTRSAFINNVLSKIGEVWAN(S)ELKNSISPYVRPNVEIQSPM
xen Armadillo    LTRSAFINNVLSKIGEVWAS(N)EPK----PYNSPNIEEPSPI
xen Sloth        LTRSAFINNVLSKIGEVWAS(N)KPKTSISPYNSPNREEPS-I
mar Opossum      LTRAAFINNVLSKIGEVWVG(N)ELKNNLSPYNSPKIEEISPV
pro Platypus     LTRAAFISNVLSKIGEVWVG(N)EHKNHFSPYRSPKNEASSPI

NO 8
GN AHR
IP IPI00021008.1
PE RHMQNEKFFRNDF
MP 560
EX Kim
CL primates
DE aryl hydrocarbon receptor
SQ
pri Human        SIMKNLGIDFEDIRHM-QNE(K)FFRNDFS--GEVDFRDID--
pri Chimpanzee   SIMKNLGIDFEDIKHM-QNE(K)FFRNDFS--GEVDFRDID--
pri Gorilla      SIMKNLGIDFEDIKHM-QNE(K)FFRNDFS--GEVDFRDID--
pri Orangutan    SIMKNLGIDFEDIKHM-QNE(K)FFRNDFS--GEVDFRDID--
pri Gibbon       SIMKNLGIDFEDIKHM-QNE(K)FFRNDFS--GEVDFRDID--
pri Rhesus       SIMKNLGIDFEDIKHM-QNE(K)FFRNDFS--GEVDFRDID--
pri Baboon       SIMKNLGIDFEDIKHM-QNE(K)FFRNDFS--GEVDFRDID--
pri Marmoset     SIMKNLGIDLEDIKHM-QNE(K)FFRNDFS--GEVDFRDID--
pri Tarsier      SIMKNLGIDFEDIKNM-QNE(K)FFRNDFS--GEVDFRDID--
pri Bushbaby     NIMKNLDIDSEDIKHM-QDD(K)FFRNDFP--GEVDFRDID--
pri Mouse_lemur  SIMKNLGIDFEDIKHM-QNE(K)FFRNDFS--GEVDFRDID--
eua Treeshrew    SIMKNLGIDFEDIKHM-QNE(R)FFRAEFP--GEVDFRDID--
eua Mouse        SIMRNLGIDFEDIRSM-QNE(E)FFRTDSTAAGEVDFKDID--
eua Rat          SIMRNLGIDFEDIRSM-QNE(E)FFRTDSS--GEVDFKDID--
eua Kangaroo_rat HTMKNLGIDFEDIKQM-QNE(E)FFGNDFS--GEVDFRDID--
eua Guinea_pig   SIMKNLGIDFEDIKCM-QNE(E)FFRTDAS--SEVDFRDID--
eua Squirrel     SIMKNLGIDFEDIKCM-QNE(E)IFRTDSS--GEVDFRDID--
eua Rabbit       NIMKNLGVDFEDIKNM-QNE(E)FFGADFS--GEVDFRDID--
eua Pika         DIMRSLGVDFEDFKHM-QNE(E)FFSTDLP--AEVDFRDID--
lau Dog          SIMKHLGIDFEDIKHMQQNE(E)FFRTDFS--GEDDFRDID--
lau Cat          SIMKHLGIDFEDIKDMQQNE(E)FFRTDFS--GEDDFRDID--
lau Horse        SIMKHLGIDFEDIRHMQQNE(E)FFRTDFS--GEEDLRDID--
lau Cow          NIMKHLGIDFEDIEHMQQNE(E)FFRTDFS--GEDDFRDID--
lau Dolphin      SIMKHLGIDFEDIKHM-QNE(E)FFRSDFS--GE-DFRDID--
lau Alpaca       SIMQRLGIDFEDIKHM-QNE(E)FFQTDFS--EENDFGDID--
lau Megabat      GIMKHLDLDFEDINHM-QNE(E)FFRTYFS--GSDDFRDID--
afr Elephant     SMMKNLGIDFEDIRHMQQNE(E)FFRADFS--GEDDFRDID--
afr Rock_hyrax   SMMKNLGIDFEDIKHM-QNE(E)FFQTDFP--SEDDFGDID--
afr Tenrec       SIMKHLGIDAEDIKHM-QNE(E)FFRSDFS--GADDFGDLD--
xen Armadillo    SIMKNLGIDFEDIKHM-QNE(E)FFRNDFS--GEDDFRDMD--
xen Sloth        SIMKNLGIDFEDIKHM-QNE(E)FFRTDFS--GEDDFRDID--
mar Opossum      GIMRSLGLDFEDLRHL-QRE(E)FFRTDLS--GGDDMGDIDPD
mar Wallaby      SIMKSLGIDFEDIRCM-QQE(E)FFRTDFS--GGDDIRDID--
pro Platypus     DIMENLGIDFEDIKHMQQDE(E)FFRAEFA--RGDDLGDID--

NO 9
GN AKAP12
IP IPI00237884.3
PE QEAEPAKELVKLK
MP 461
EX Kim
CL simians
DE A kinase (PRKA) anchor protein 12
SQ
pri Human        VPAEELVEMDA-EPQEAEPA(K)ELVKLKETCVSGEDPTQGAD
pri Chimpanzee   VPAEELVEMDA-EPQEAEPA(K)ELVKLKETCVSGEDPTQGAD
pri Gorilla      VPAEELVEMDA-EPQEAEPA(K)ELVKLKETCVSGEDPTQGAD
pri Orangutan    VPAEELVEMDA-EPQEAEPA(K)ELVKLKETCVSGEDPTQGAD
pri Gibbon       VPAEELVEMNA-EPQEAEPA(K)ELMKD----VSGEDPTQGAD
pri Rhesus       VPAEELVEMNA-EPQEAEPA(K)ELVKLKETCVSGEDPTQGAD
pri Baboon       VPAEELVEMNA-EPQEAEPA(K)ELVKLKETCVSGEDPAQGAD
pri Marmoset     VPAEELVEMNA-EPQEAEPA(K)ELVKLKDTCVSGVDPVQGAD
pri Tarsier      LPPGKSVEASA-EPQEGEPP(T)ELEKTKDACVSGGDQPQGAD
pri Mouse_lemur  LPPEKSVETDV-EPQEAEPA(E)DLGKTKEACAPGEDHTQVPE
eua Treeshrew    LPPEKFVETEA-ELQEAEPA(E)ELGKTREVCAPGGDHLQQAE
eua Mouse        LPPEKLAETQE-VPQEAEPV(E)ELMKTKEVCVSGGDHTQLTD
eua Rat          LPPEKLAEPQE-VPQEAEPA(E)ELMKSREMCVSGGDHTQLTD
eua Guinea_pig   LSSEKLLETNAQEAQEAELA(E)ALEKTKEVPASGGDHARPTE
lau Cat          PPPETAVETPA-ELEKARPA(A)EPAKAAEACAPGGDHSRPAD
lau Horse        LLPEKLVEADV-ELEAAEPA(E)DL-KAEDTCAPGGEQTKPSE
lau Cow          SPPEKSTETSA-DLQEAESA(E)MLKTEEDAGALSGDHSQATE
lau Dolphin      SPPERSLETDA-DLPDAEPP(E)ALLKMQEAGAPSGDHAQP-E
lau Megabat      LPTEKLLETNA-ELQEAEPA(E)ELVKTKAVCAPGGDHTQPAE
afr Elephant     LPPEKSVETNA-ELQEAEPA(E)ELVMTEEVCAPGEDHIQPAE
afr Rock_hyrax   LPPEKTVERKA-ELQEAELA(E)DSVKAEDLSCPGEEHPQPAE
afr Tenrec       LPPEKSAETTA-QVQAAEPA(E)EVVMTREASVPGEDQAQPTE
xen Armadillo    LLTEASFETNA-ELQEAEPV(Q)GLVKTKELCAPEDDHTQFKE
pro Platypus     LPLDQSPEAAA-EQQKIDSV(E)ESSKTKEVCTSEGDHDTQTE

NO 10
GN AKAP12
IP IPI00237884.3
PE AATEVSKELSESQ
MP 882
EX Kim
CL simians
DE A kinase (PRKA) anchor protein 12
SQ
pri Human        QQAQKSAEQPEQK-AATEVS(K)ELSESQVHMMAAAVADGTRA
pri Chimpanzee   QQAQKSAEQPEQK-AAAEVS(K)ELSESQVHMMAAAVVDGTRA
pri Gorilla      QQAQKSAEQPEQK-AAAEVS(K)ELSESQVHMMAAAVVDGTRA
pri Orangutan    QQAQKSAEQPEQK-AAAEVS(K)ELSESQAHMMAAAVIDGMRA
pri Gibbon       QQAHKSTEQPEQK-AAAEVS(K)ELSESQAHMMAAAVIDGTRA
pri Rhesus       QQAQKSAEQPEQK-AAAEVS(K)ELSESQGHTMAAAVVDGIRA
pri Baboon       QQAQKIAEQPEQK-AAAEVS(K)ELSESQGHTMAAAVVDGIRA
pri Marmoset     QQAQKSIEQPEHQ-EAAEVS(K)ELSDSQGHMVAAAVIDGTRA
pri Bushbaby     QRDHISADKPEQM-ADDRVS(E)ELSKTLDHTVAAAVLDGTRA
pri Mouse_lemur  QQAQKSAEKPEQM-AGVCVS(E)ELSKTLVHTVAVAVVDGTRA
eua Treeshrew    QQAQEGVEKPEQT-VAVYVS(E)ELSKTLVHTVAVAVVDGTRA
eua Mouse        QRAQENVELPQLK-GAVYVS(E)ELSKTLVHTVSVAVIDGTRA
eua Rat          --AQGNAELPQLL-GAVYVS(E)ELSKTLVHTVSVAVIDGTRA
eua Kangaroo_rat QLARGGAEPPQPQ-VDLYVS(E)EVSQSLVHTVTVAVIDGARA
eua Squirrel     QQALQSAETSQGK-MPVDVP(E)ELSKNLVHTVTVAVIDGTRA
lau Dog          QQAQKSEEQPEQK-AAVSVS(E)ELSTDLVRGVTVTVLDGARA
lau Cat          QQAQKGEEKPEQK-VAVSVS(E)ELGTTLVHAVAVTVVDGARA
lau Horse        QQAQK-EEAPEPK-AAVHVS(E)ELSKSLVQAVTVAVVDGTRA
lau Cow          QPAPKSQEGPEQKQVDVDVS(E)ELSKSLVHTVSVAVVDGTRA
lau Dolphin      QQAPKSEEGPEQK-VAVDVS(E)ELSKSLVHTVTVAVMDGTRA
lau Megabat      QEARKSEERPERQ-VAVYVS(E)EVSKSLVHRLVVADIDGARA
lau Microbat     QEAQKREEKAEQK-GAVSVS(E)ELSKSLVHTVTVAVVDGARA
afr Elephant     QQAPRSEEKPEQK-VAVCVS(E)ELSKSLVQTVTVTVVDGLRA
afr Tenrec       QQAQKR-EKPALE-APVYVS(E)ELSKSLVHTVAVTVIDGTRA
mar Wallaby      Q--QKNAKQQKRK-VECPGT(E)ETVEGLVHAVSVTVVEGERA

NO 11
GN AKAP12
IP IPI00237884.3
PE IVSATTKKGLSSD
MP 1493
EX Kim
CL African great apes
DE A kinase (PRKA) anchor protein 12
SQ
pri Human        VPTGPDCQAKSTPVIVSATT(K)KGLSSDLEGEKTTSLKWKSD
pri Chimpanzee   VPTGPDCQAKSTPVIVSATT(K)KVLSSDLEGEKTTSLKWKSD
pri Gorilla      VPTGPDCQAKSTPVIVSATT(K)KGLSSDLEGEKTTSLKWKSD
pri Orangutan    VPTGPECQAKSTPVIVSATT(E)KGLSSDLEGEKTTSLKWKSD
pri Gibbon       VPTGPECQAKSTPVIVSATT(E)KGLSSDLEGEKTTSLKWKSD
pri Rhesus       VPAGPECRAKSTPVIVSATT(E)KGLSSDLEGEKTTSLEWKSD
pri Baboon       VPAGPECRAKSTPVIVSATT(E)KGLSSDLEGEKTTSLEWKSD
pri Marmoset     VPTVPEGQAKSIPGIGSAGT(E)ESLRSDLDGERTTSLKWKSD
pri Tarsier      VSTGPESQAESVAVTVSATT(E)KDFCSDLDGEKTKSLRWKLD
pri Bushbaby     EPTGPGSQAESITEITSATS(E)KSLCSDLEGEKTTSLKRKSD
pri Mouse_lemur  EPTGPESQAEATPVTASATH(E)EGFCSALEGEKTPSQKWNSD
eua Mouse        VPQGPESQAESIPIIVTPAP(E)SILHSDLQREVSASQKQRSD
eua Rat          VPLGPESQAESIPIIVTPAP(E)STLHPDLQGEISASQRERSE
eua Guinea_pig   LAVGPEPQAESEPVITLATP(E)KDLLSGLGGEESAFQKQKGD
eua Squirrel     VLEGPEPQAESMPAIVSATP(E)RGLPSDLEGEKSTSQKQRSD
eua Rabbit       VSPVPDAQAESIPVMAGAGT(E)EDVSSEPEGDKTTSQKWTSE
eua Pika         VSTAPEPQTGSMTVTGGAAA(E)DDVSSEPEGDKTASQKWTSE
lau Dog          VPTGTEARPEPGPGTASAEP(D)GGTSAAPEGEKTTPQRQTSE
lau Cat          VPAGTESQPESVPEAVSAAP(E)GGISADLEADKTTPQKRASD
lau Horse        APTGTETQAESTPEVISATP(E)RGIHADLEEDKTTSQKQKLD
lau Cow          ASTGTESQAESIPTIVSITP(G)KGIDADLEGDETTSQKQELG
lau Dolphin      VPTGTESQAKPIPVIVSVMP(E)KGISADLEGDKTTSQKWESD
lau Megabat      VPVGTKSQAESSPVIVSATP(E)KGTSADLEQDKTTSQKEKSE
lau Microbat     APTGPASPAESAPVVIPAAP(A)KGVRADLEGDKTTSQEGKSE
afr Elephant     VPTGXGSQAESIPPIVSAPA(E)EGGSSDLEGDKTTSQKWKPD
afr Rock_hyrax   VPTRPESQAASVPVTVSAPA(E)KEVTSDPEGDKTTAQKGKPD
xen Sloth        VPMGTESQAELIPVIVSATT(E)ECLCSDLKGDQTPSQKWKSD
pro Platypus     GSTESASPAEPISGTVTAVA(E)KLKTSDLEQGCSKLHKLKLD

NO 12
GN ALDH2
IP IPI00006663.1
PE GNPFDSKTEQGPQ
MP 355
EX Kim
CL primates
DE aldehyde dehydrogenase 2 family (mitochondrial)
SQ
pri Human        FVERSVARAKSRVVGNPFDS(K)TEQGPQVDETQFKKILGYIN
pri Chimpanzee   FVERSVARAKSRVVGNPFDS(K)TEQGPQVDETQFKKILGYIN
pri Gorilla      FVERSVARAKSRVVGNPFDS(K)TEQGPQVDETQFKKILGYIN
pri Orangutan    FVERSVARAKSRVVGNPFDS(K)TEQGPQVDETQFKKILGYIN
pri Rhesus       FVERSVARAKSRVVGNPFDS(K)TEQGPQVDETQFKKILGYIN
pri Baboon       FVERSVARAKSRVVGNPFDS(K)TEQGPQVDETQFKKILGYIN
pri Marmoset     FVDRSVARAKSRVVGNPFDS(K)TEQGPQVDETQFKKILGYIN
pri Bushbaby     FVERSVARAKSRVVGNPFDS(R)TEQGPQVDETQFKKILGYIN
pri Mouse_lemur  FVERSVARAKSRVVGNPFDS(K)TEQGPQVDETQFKKILGYIN
eua Mouse        FVERSVARAKSRVVGNPFDS(R)TEQGPQVDETQFKKILGYIK
eua Rat          FVERSVARAKSRVVGNPFDS(R)TEQGPQVDETQFKKILGYIK
eua Guinea_pig   FVERSIARAKARVVGNPFDS(R)TEQGPQVDETQFKKILGYIK
eua Squirrel     FLERSVARAKSRVVGNPFDS(R)TEQGPQVDETQFKKILGYIQ
eua Rabbit       FVERSVARAKARVVGNPFDS(Q)TEQGPQVDETQFKKILGYIN
eua Pika         FVERSVARAKSRVVGNPFDS(R)TEQGPQVDETQFKKILGYIK
lau Dog          FVERSVARAKSRVVGNPFDS(Q)TEQGPQVDETQFKKILGYIK
lau Cat          FVERSVARAKSRVVGNPFDS(Q)TEQGPQVDKTQFQKVLGYIK
lau Horse        FVERSVARARSRVVGNPFDS(Q)TEQGPQVDETQFNKVLGYIK
lau Cow          FVERSVARAKSRVVGNPFDS(R)TEQGPQVDETQFKKVLGYIK
lau Dolphin      FVERSVARARSRVVGNPFDS(R)TEQGPQVDETQFKKVLGYIK
lau Alpaca       FVERSVARARSRVVGNPFDS(Q)TEQGPQVDETQFKKVLGYIK
lau Megabat      FVERSVTRAKSRVVGNPFDS(R)TEQGPQVDETQFKKILGYIK
lau Microbat     FVERSVARAKSRVVGDPFDS(Q)TEQGPQVDETQFKKILGYIN
lau Hedgehog     FVERSVARAKSRVVGNPFDS(Q)TEQGPQVDETQFNKVLGYIK
afr Elephant     FLERSVTRAKARVVGNPFDS(Q)TEQGPQVDETQFKKILGYIQ
xen Armadillo    FVERSVARAKSRVVGNPFDS(R)TEQGPQVXETQFNKIHGYIK
mar Opossum      FVERSVARAKARVVGNPFDS(R)TEQGPQVDEEQFKKILGYIN
mar Wallaby      FVERSVARAKARVVGNPFDS(R)TEQGPQVDKEQYKKILGYID
pro Platypus     FVERSVARAKSRVVGNPFDS(Q)TEQGPQVNEEQFKKILGYIS

NO 13
GN ALG8
IP IPI00032370
PE DGSIRWKSFSFVR
MP 231
EX Wagner
CL apes
DE asparagine-linked glycosylation 8, alpha-1,3-glucosyltransferase homolog (S. cerevisiae)
SQ
pri Human        VYLLRSYCFTANKPDGSIRW(K)SFSFVRVISLGLVVFLVSAL
pri Chimpanzee   VYLLRSYCFTANKPDGSIRW(K)SFSFVRVISLGLVVFLVSAL
pri Gorilla      VYLLRSYCFTANKPDGSIRW(K)SFSFVRVISLGLVVFLVSAL
pri Orangutan    VYLLRSYCFTANKPDGSIRW(K)SFSFVRLISLGLVVFLVSAL
pri Gibbon       VYLLRSYCFTANKPDGSIRW(K)SFSFVRVISLGLVVFVVSAL
pri Rhesus       VYLLRSYCFTANKPDGSIRW(N)SFSFVRVISLGLVVFLVSAL
pri Baboon       VYLLRSYCFTANKPDGSIRW(N)SFSFVRVISLGLVVFLVSAL
pri Marmoset     VYLLRSYCFTANKPDGSIRW(N)SFSSVRVISLGLVVFLVSAL
pri Tarsier      VYLLRSYCFTANKPDGSIRW(N)SFNFIRVISLGLVVFLVSAL
pri Bushbaby     XXXXXXXXXXXXXXXGSIRW(N)SFSFTRVISLGLVVFLVSAL
pri Mouse_lemur  XXXXXXXXXXXXXXXGSIRW(N)SFSFLRVTSLGLVVFLISAF
eua Mouse        VYLLRSYCFTASKPDGSVRW(S)SFSVVRVTSLGLIVFLVSAL
eua Rat          IYLLRSYCFTASKPDGSVRW(D)SFSIVRVTALALIVFLVSAL
eua Kangaroo_rat VYLLRSYCFISSKPDGSVQW(N)SFSFVRVFSLGLIVFLVSAL
eua Guinea_pig   IYLLRSYCFTASKPDGSVRW(N)SFSFTRLISLGLIVFLVSAL
eua Squirrel     IYLLRSYCFAANKPDGSVRW(N)SFSFVRVILLGLIVFLVSAL
eua Rabbit       VYLLRSYCFTAVKPDGSVRW(N)SFSFVRVTSLGLVVFLVTAL
lau Dog          IYLLRSYCFTANKPDGSIRW(N)SFSFIRLISLGLIVFLVSAL
lau Horse        IYLLRSYCFTANKPDGSIRW(N)SFSFVRLISLGLTVFLVSAL
lau Cow          VYLLRSYCFTANKQDGSIRW(N)SFSFVRLISLGLIVFLVSAL
lau Dolphin      VYLLRSYCFTANKPDGSIRW(N)SFSFLRLISLGLIVFLVSAL
lau Alpaca       IYLLRSYCFTANKPDGSIRW(N)SFSFVRLISLGLIVFLVSAL
lau Megabat      IYLLRSYCFTANKPDGSILW(N)SFSVVRFISLGLIVFLVSAL
lau Microbat     IYLLRSYCFTANKPDGSVRW(N)SFNFVRLISLGLIVFLVSAL
lau Shrew        IYLLRSYCFTANKPDGSIRW(T)SFSFIRLTSLGMVVFLVSAL
afr Elephant     VYLLRSYCFTANKPDGSVRW(N)SFSFVRVISLGLIAFLVSAL
afr Rock_hyrax   IYLLRSYCFTANKPDGSIRW(N)SFSFVRVISLGLIVLLVTAL
afr Tenrec       IYLLRSYCFTANKPDGSVRW(D)SFSLARVASLGLIVFIVSAV
xen Armadillo    IYLLRSYCFAANKPDGSIRW(N)SFSFIRVISLGLIVFLVSAL
xen Sloth        VYLLRSYCFTANKPDGSIRW(K)SFSFIRVISLGLIVFLVSAL
mar Opossum      IYLLRSYCFSSDNPDGSIRW(S)SFSFLRLFTLSFIVCLISAL

NO 14
GN ANGEL2
IP IPI00375317.2
PE LTQTQLKQTEVLV
MP 261
EX Kim
CL primates
DE angel homolog 2 (Drosophila)
SQ
pri Human        EVQQVPKVEKTDSDLTQTQL(K)QTEVLVTAEKLSSNLQHHFS
pri Chimpanzee   EVQQVPKVEKTDSDLTQTQL(K)QTEVLVTAEKLSSNLQHHFS
pri Gorilla      EVQQVPKVEKTDSDLTQTQL(K)QTEVLVTAEKLSSNLQHHFS
pri Orangutan    EVQQVPKVEKTDSDLTQTQL(K)QTEVLVTAEKLSSNLQHHFS
pri Gibbon       EVQQVPKVEKTDSDLTQTQL(K)QTEVLVTAEKLSSNLQHHFS
pri Rhesus       EVQQVPKVEKTDSDLTQTQL(K)QTEVLVTAEKLSSNLQHHFS
pri Baboon       EVQQVPKVEKTDSDLTQTQL(K)QTEVLVTAEKLSSNLQHHFS
pri Marmoset     EVQQVPKVEKTDSDLTQTQL(K)QTEVLVTAEKLSSNLQHRFS
pri Tarsier      EVQQVPKVEKTGSDLTQTQL(K)KTEVLVTAEKLSSNLQHHFS
pri Mouse_lemur  EVQQVPKVEKTDSDLTQTEL(K)KTEVVVTAEKXXXXXXXXXX
eua Treeshrew    XXXXXXXXXKTDSDLTQTQL(E)KTEVLVTTEKLSSNLQHHFS
eua Mouse        EAQQVPKVEKTDSDVTQAQQ(E)KAEVPVSADKVSSHLQHGFS
eua Rat          EAQQVPKVEKTDSDVTQAQQ(E)KAEVPVPADKVSSHLQHGFS
eua Kangaroo_rat EVQQVSKVEKTDGDVTQAQL(G)ETDILVTAEKVSSHLQHHFS
eua Guinea_pig   EAQQGPKVAESDGGLTQTQL(E)NTEVLVTAEKLPSSLQHHFS
eua Rabbit       EGQQVPKVEKTDCDLTQTQQ(E)KTEALVTAEKLSSSLQHHFS
eua Pika         EVQQVPKVEKTDSDVTETQQ(E)QTEALVTTEKLSSSLQHHFS
lau Dog          EVQQLPKVEKTDSDLTQTEL(D)KTEVLVTAEKLSSNLQHHFS
lau Cat          EVQQLPKVEKTDGDLTQTEL(D)KTEVLVTAEKLSSNLHHHFS
lau Horse        EVQQVPKVEKTDGDPTQTQL(D)KTEVLVTPEKLSSHLQHHFS
lau Cow          EVQQVPKVEKPDGDLTQPEL(D)KTEVLVTAEKLSSNLQHHFS
lau Dolphin      EVQQVPKVEKTDSDLTQTQL(D)KTEVLVTAEKLSSKLQHHFS
lau Alpaca       EVQQVPKVEKTDSDLTQTEL(D)KTGVLVTAEKVSSNLQHHFS
lau Megabat      EVQQVPKVEKTDSDLTQTQL(D)KTEVLETAEKLSSNLQHHFS
lau Shrew        EGPRAQRTEKAGADPTQTEL(N)KSEVVEPAEKSSSSLQHRFS
afr Elephant     EVQQVPKVEKTDDSLTQTQL(E)KTDVMVTPEKLSSNLQHHFS
xen Sloth        EVQQVPKIEKTDSDLTQTQL(D)KTEVIVTADKLSSNLKHHFS
mar Opossum      EIPQVTRVEKAEDDMTQKQL(E)KTEVLIATEKLSSNLHHHFS
mar Wallaby      ELPQVTGVEKPEDDVTQKQL(E)KTEVLVAAKKLSSNLHHHFS

NO 15
GN ANKIB1
IP IPI00292914.4
PE DGSEGVKDVELVL
MP 1012
EX Kim
CL simians
DE ankyrin repeat and IBR domain containing 1
SQ
pri Human        TTEISADSQLPCIKDGSEGV(K)DVELVLPEDSMFEDASVSEG
pri Chimpanzee   TTEISADSQLPCIKDGSEGV(K)DVELVLPEDSMFEDASVSEG
pri Gorilla      TTEISADSQLPCIKDGSEGV(K)DVELVLPEDSMFEDASVSEG
pri Orangutan    TTEISADSQLPCIKDGSEGV(K)DVELVLPEDSMFEDASVSEG
pri Gibbon       TTEISADSQLPCIKDGSEGV(K)DVELVLPEDSMFEDASVSEG
pri Rhesus       TTEISADSQLPCIKDGSEGV(K)DVELVLPEDSVFEDASVSED
pri Baboon       TTEISADSQLPCIKDGSEGV(K)DVELVLPEDSVFEDASVSED
pri Marmoset     TTEISADSQLPCIKDGSEGV(K)DVELVLPEDSVFEDASGREG
pri Tarsier      TTEIGADSQLSCVKDGSEGV(R)EMELVSPEDSIFEDASVSEG
pri Bushbaby     TTEISEDSQLPCVKDGSEGV(R)DVELVPPEDSVFEDASVSEG
pri Mouse_lemur  ATEISADSQLPCVKDGSEGV(R)DAELVPPEDSVFEDASVSEG
eua Treeshrew    TTEISADSQLPCVKDGSEGV(R)DVELMPPEDSVFEDASVSEG
eua Mouse        TTEISAEPQLPCIRDGSEGV(R)DMELVPPEDSVSKDTGVHEG
eua Rat          TTETSAEPPLPCVRDGSEGV(R)DTELVPPEDSVSKDAGVHEG
lau Dog          TTETSADSQLPCVKDELEGT(R)DVELVPPEDSVFEDAVVNEG
lau Horse        STEISADSQHPYVKDGSEGV(R)GMELVPPEDAMFEDAVISKG
lau Cow          ATEVSADSQLPCVKDESEGV(R)DVELMPPEDSVFGDAAISEG
lau Dolphin      ATEISADSQLPCVKDESEGV(R)DVELMLPEDSVFEDAVVSEG
lau Alpaca       APEISADSQLPCVKDGSEGV(R)DVELMQPEDSVFEDAVVSEG
lau Megabat      AAEISSDSQLPCVRDASEDV(R)DVGPAPPEDSVFEDAVVSEC
lau Microbat     TTETSADSQRPCVGDGSEGV(K)DVELVPPEDSVFEDAVVSEG
lau Hedgehog     TTEVSAESQLPCVKDGSEGM(R)DVELVPPEDSVFEDAIVSEG
lau Shrew        TTDISADSQPPCVKDGSEDV(R)DMELVLPEESVFQDAIVSED
afr Elephant     TTEISADSQLPSVKDGSEGM(R)DVEVVPPEDSIFEDAVISEG
afr Rock_hyrax   PAEISADSQLTCEKDGSEGL(R)DVELVPTEDAVFEDAVVSES
afr Tenrec       VTESSANSQHPCVKDGSEGM(R)DVEMLPPEXSVFEDAVISEG
xen Armadillo    TTEVSADSQLPCVKNESEYM(R)DVEQVQPEDSVFEDAVVSED
xen Sloth        TTELSADSQLPYVKDGSEGG(R)DVELVPPEDSVFEDAVLSEG

NO 16
GN ANKRD13A
IP IPI00217831
PE LHLLVWKNDYRQL
MP 19
EX Wagner
CL catarrhines
DE ankyrin repeat domain 13A
SQ
pri Human        MSSACDAGDHYPLHLLVW(K)NDYRQLEKELQGQNVEAVDP
pri Chimpanzee   MSSACDAGDHYPLHLLVW(K)NDYRQLEKELQGQNVEAVDP
pri Gorilla      MSSACDAGDHYHLHLLVW(K)NDYRQLEKELQGQNVEAVDP
pri Orangutan    MSSACDAGDHYPLHLLVW(K)NDYRQLEKELQGQNVEAVDP
pri Gibbon       MSSACDAGDHYPLHLLVW(K)NDYRQLEKELQGQNVEAVDP
pri Rhesus       MSSACDASDHYPLHLLVW(K)NDYRQLEKELQGQNVEAVDP
pri Baboon       MSSACDASDHYPLHLLVW(K)NDYRQLEKELQGQNVEAVDP
pri Marmoset     MSSACDAGNHYPLHLLVW(N)NDYRQLEKELQGQNVEAVDP
pri Tarsier      MSSACGAGSRFPLHLLVW(N)NDYRQLEKELRGQNVEALDP
pri Mouse_lemur  MSSACDASSHFPLHLLVW(N)NDYRQLEKELRDQNVEALDP
eua Mouse        MSSARDTSSRFPLHLLVW(N)NDYEQLEKELRDQNAEALDP
eua Rat          MSSARDTSSRFPLHLLVW(N)NDYRQLEKELRDQNAEALDP
eua Kangaroo_rat MSSARDARGQFPLHLLVW(N)NDYRQLEKELRDQNVEALDP
eua Guinea_pig   MSSVRDASSHFPLHLLVW(N)NDYRQLEKVLRGQNVEALDP
eua Rabbit       MSSARDVSDHFPLHLLVW(N)NDYRQLEKELRGQNVEALDP
eua Pika         MSSSRDASDHFPLHLLVW(N)NDYRQLEKELRGQNVEALDP
lau Dog          MSSARDSSGHFPLHLLVW(N)NDYRQLEKELRGQNVEALDP
lau Horse        MSSARDAGGHFPLHLLVW(N)NDYRQLERELRGQNVEALDP
lau Cow          MSSPRDASGDFPLHLLVW(N)NDYRQLEKELRDQNVEALDP
lau Dolphin      MSSACDASGHFPLHILVW(N)NDYRQLEKELQGQNVEALDP
lau Alpaca       MSSARDASGHFPLHLLVW(N)NDYRQLEKELRGQNVEALDP
lau Microbat     MSAARDSSGHFPLHLLVW(N)NDYRQLEKELRDQNVEAVDP
afr Elephant     MSSARDASGHFPLHLLVW(K)NDYRQLEKELRDQNVEALDP
afr Tenrec       MSSRRDPGGPFPLHLLVW(N)NDYRQLEKELRDQNVEARDP
xen Armadillo    MSSAHDASSHFPLHLLVW(N)NDYRQLEKELRSQNVEALDP
xen Sloth        MSSAHDASSHFPLHLLVW(N)NDYRQLEKELRGQNVEALDP
mar Opossum      MSCGGDASSQFPLHALVW(N)NDYRQLEKALDGQNVEALDP

NO 17
GN ANXA3
IP IPI00024095.3
PE DAQILYKAGENRW
MP 184
EX Kim
CL catarrhines
DE annexin A3
SQ
pri Human        RDESLKVDEHLAKQDAQILY(K)AGENRWGTDEDKFTEILCLR
pri Chimpanzee   RDESLKVDEHLAKKDAQILY(K)AGENRWGTDEDKFTEILCLR
pri Gorilla      RDESLKVDEHLAKKDAQILY(K)AGENRWGTDEDKFTEILCLR
pri Orangutan    RDESLKVDEHLAKKDAQILY(K)AGENRWGTDEDKFTEILCLR
pri Gibbon       RDESLKVDEHLAKKDAQILY(K)AGENRWGTDEDKFTEILCLR
pri Rhesus       RDESLKVDEHLAKKDAQILY(K)AGENRWGTDEDKFTEILCLR
pri Baboon       RDESLKVDEHLAKKDAQILY(K)AGENRWGTDEDKFTEILCLR
pri Marmoset     RDESLKVDEHLAKKDAQILY(N)AGENRWGTDEDKFTEILCLR
pri Tarsier      XXXXXXXXXXXXXXXXXLLY(N)AGENRWGTDEDRFTEILCLR
pri Bushbaby     RDETLKVDEHLAKKDAQILY(N)AGESRWGTDEDKFTEILCLR
eua Treeshrew    RDESLKVDEHLAKKDAQVLY(Q)AGENKWGTDEDKFTEILCLR
eua Mouse        RDESLKVDEHLAKKDAQILY(N)AGENKWGTDEDKFTEVLCLR
eua Rat          RDESLKVDEHLAKKDAQTLY(D)AGEKKWGTDEDKFTEILCLR
eua Kangaroo_rat RDESLKVDEHLAKKDAQVLY(N)AGENKWGTDEDQFTEILCLR
eua Guinea_pig   RDESLKVDEHLAKKDAQILY(N)AGENKWGTDEDKFTEILCLR
eua Squirrel     XXXXXXXXXXXXXXXXXILY(N)AGENKWGTDEDKFTEILCLR
eua Rabbit       RDESVKVDEHLAKKDAQILY(N)AGENKWGTDEDKFTEILCLR
eua Pika         RDESLKVDEHLAKKDAQILY(N)AGENKWGTDEDKFTEILCLR
lau Dog          RDESLKVDEHLAKKDAQILY(N)AGENRWGTDEDKFTEILCLR
lau Horse        RDESLKVDEHLAKKDAQILY(N)AGENRWGTDEDKFTEILCLR
lau Cow          RDESLKVDEQLARKDAQILY(N)AGEKRWGTDEDAFTDILCLR
lau Dolphin      RDEGLKVDEQLAKEDAQILY(N)AGENKWGTDEDTFTEILCLR
lau Alpaca       REESQKVDEQLAKKDAQILY(N)AGEKRWGTDEDTFTEILCLR
lau Megabat      RDESLKVDEHLAKKDAQILY(N)AGENRWGTDEDKFTEILCLR
lau Microbat     RDESLKVDEQLAKDAXXILY(N)AGEKKWGTDEDKFTEILCLR
lau Hedgehog     RDESLKVDEHLAKKDAQILY(N)AGENKWGTDEDKFTEILCLR
afr Elephant     RDESLKVDEHLAKKDAQILY(N)AGEKKWGTDEDKFTEILCLR
afr Rock_hyrax   RDENLKVDEHLAKKDAQILY(N)AGEKRWGTDEDKFTEILCLR
afr Tenrec       RDESLKVDEHLAKKDAQILY(D)AGEKRWGTDEEKFIEVLCLR
xen Armadillo    XXXXXXXXXXXXXXXXXILY(N)AGEKKWGTDEDKFTEILCLR
xen Sloth        RDESLMVDEHLAKDDAQILY(N)AGEKNWGTDEDKFTEILCLR
mar Opossum      RDESLKVDEYLAKKDAQILY(E)AGEKRWGTDEDKFTEILCLR
mar Wallaby      REEGLKVDEDLAKKEAQILY(D)AGEKKWGTDEDTFTEILCLR

NO 18
GN AP1M2
IP IPI00219794.5
PE GDVAMSKIEHFMP
MP 29
EX Kim
CL simians
DE adaptor-related protein complex 1, mu 2 subunit
SQ
pri Human        LDVKGKPLISRNYKGDVAMS(K)IEHFMPLLVQREEEGALAPL
pri Chimpanzee   LDVKGKPLISRNYKGDVAMS(K)IEHFMPLLVQREEEGALAPL
pri Gorilla      LDVKGKPLISRNYKGDVAMS(K)IEHFMPLLVQREEEGTLAPL
pri Orangutan    LDVKGKPLISRNYKGDVAMS(K)IEHFMPLLVQREEEGALAPL
pri Gibbon       XXXXXXPLISRNYKGDVAMS(K)IEHFMPLLVQREEEGALTPL
pri Rhesus       LDVKGKPLISRNYKGDVAMS(K)IEHFMPLLVQREEEGALAPL
pri Baboon       LDVKGKPLISRNYKGDVAMS(K)IEHFMPLLVQREEEGALAPL
pri Marmoset     XXXXXXPLISRNYKGDVPMS(K)IEHFMPLLVQREEEGALTPL
pri Bushbaby     LDVKGKPLISRNYKGDVAMS(E)IEHFMPLLVQREEEGALAPL
eua Mouse        LDVKGKPLISRNYKGDVPMT(E)IDHFMPLLMQREEEGVLAPL
eua Rat          LDVKGKPLISRNYKGDVPMT(E)IDHFMPLLMQREEEGMLAPL
eua Kangaroo_rat LDVKGKPLISRNYKGDVPMS(E)IERFMPLLMQREEEGALTPL
eua Guinea_pig   XXXXXXPLISRNYKGDVAMS(E)IDHFMPLLMQREEEGALAPL
eua Squirrel     LDVKGKPLISRNYKGDVAMS(E)IDHFMPLLMQREEEGALAPL
eua Pika         LDVKG-PLISCSYKGDVPMS(E)IDYFMPLLMQHEKEGSLAPL
lau Dog          IEVKVKPLISRNYKGDVAMS(E)IEHFMPLLMQREEEGALAPL
lau Horse        LDVKGKPLISRNYKGDVAMS(E)IEHFMPLLMQREEEGVLAPL
lau Cow          LDVKGKPLISRNYKGDVAMS(E)IDHFMPLLMQREEEGALTPL
lau Dolphin      XXXXXXPLISRNYKGDVAMS(E)IEHFMPLLMQREEEGALAPL
lau Megabat      LDVKGKPLISRNYKGDVAMS(E)IEHFMPLLLQREEEGALAPL
lau Microbat     LDVKGKPLISRNYKGDVAMS(E)IEHFMPLLMQREEEGALTPL
afr Elephant     LDVKGKPLISRNYKGDVAMS(E)IEHFMPLLMQREEEGALAPL
afr Rock_hyrax   LDVKGKPLISRNYKGDVAMS(E)IEHFMPLLMQREEEGALAPL
mar Opossum      LDMKGKPLICRNYKGDVSMT(E)IDHFMPLLMQKEEEGALTPL
mar Wallaby      LDMKGKPLICRNYKGDVNMA(E)IDHFMPLLMQKEEEGALTPL

NO 19
GN APOB
IP IPI00022229.1
PE EIKTLLKAGHIAW
MP 2915
EX Kim
CL humans and chimpanzees
DE apolipoprotein B (including Ag(x) antigen)
SQ
pri Human        IPKLDFSSQADLRNEIKTLL(K)AGHIAWTSSGKGSWKWACPR
pri Chimpanzee   IPKLDFSSQADLRNEIKTLL(K)AGHVAWTSSGKGSWKWACPR
pri Gorilla      IPKLDFSSQADLRNEIKTLL(E)AGHIAWTSSGKGSWKWACPR
pri Orangutan    IPKLDFSSQADLRNEIKTLL(E)AGHITWTSSGKGSWKWACPR
pri Gibbon       IPKLDFSSQADLRNEIKTLL(E)ASHIAWTSSGKGSWKWACPR
pri Rhesus       IPKLDFSSQADLRNEIKTLL(E)AGHIAWTSSGKGSWKWACPR
pri Baboon       IPKLDFSSQADLRNEIKTLL(E)AGHIAWTSSGKGSWKWACPR
pri Marmoset     IPKLDFSSQADLRNEVKTLL(E)AGHVAWTSSGKGSWKWACPH
pri Bushbaby     IPKLDFSSQADLRNEIKTLL(K)AGHVAWTSSGTGSWKWAYPK
pri Mouse_lemur  IPKLDFSSQADLRNEIKTLL(K)AGHVAWTSSGLGSWKLACPN
eua Mouse        VPRLDFSSKASLNNEIKTLL(E)AGHVALTSSGTGSWNWACPN
eua Rat          VPRLDFSSKASLNNEIKTLL(E)AGHMAWTSSGTGSWNWACPN
eua Kangaroo_rat IPQFDFSSKAELLNEMKTLM(E)AGDLSWTSSGSGSWKWSCPN
eua Squirrel     IPKLDLSSKVDLNNDIKTLL(E)AGHLTWTSSGIGSWKLACLN
eua Rabbit       VPSLDISSQADLHNEIKTLL(E)AGHVTWTSSGTGTWKWACPK
eua Pika         IPNLDFSSQADLQNEIKTLL(E)AGYMTWTSSGTGTWKWACPK
lau Dog          IPKLDFSSQAELRNEIKTLL(E)SGHITWTSSGLGSWKWACNT
lau Cat          IPKLDFSSQAELRNEIKTLL(E)AGHVAWTSSGIGSWKWAHQT
lau Horse        IPELDFSSQADLHNEIKTLL(E)AGHIAWTSSGTGSWKWACPK
lau Cow          IPQLDFSSQADLHSDLKTLL(E)AGHIAWASSGRASWKLACHE
lau Dolphin      IPKLDFSSQADLRSEIKTLL(E)AGHLAWTSSGTGSWKLACHK
lau Alpaca       IPKLDFSSQADLRNEIKTLL(E)ARHIAWTSSGTGSWKLARHK
lau Microbat     IPKLDFSSQADLHDEIKTLW(D)SEKIAWTSSGRASWKVACPK
lau Hedgehog     IPTFDFSSQADLRNDIKTLL(E)VGHVTLSSSGTGSWKWAHPK
lau Shrew        FPKLEFSSQADLRNEIKTLL(E)VGHIDWTSSGTGSWKWACPR
afr Elephant     IPKLDFSSQADLRNEMKTLL(E)AGRITWTSSGTGSWKWACPK
afr Rock_hyrax   IPKLEFSSQADLHNEMKTLL(E)AGHVTWTSSGTGSWKWACPK
afr Tenrec       IPQLDFSSQADLRNGMKTLW(E)AGRIGWTSSGTGSWKWACPK
xen Sloth        IPTLDFSSQADLRNEIKTLL(E)AGHLSWASSGMGSWKWASPK
mar Opossum      LPQMDFSSQTDLRHELAMLL(E)AGRVAATSTGKGAWKWAFPR
mar Wallaby      LPQGDFSSQMNLHHELRMLL(V)LERVAMTSTGMGSWKWAFPH
pro Platypus     IPQADFSSQADLRNEVKALL(E)AGHVA-SSEGTGSWKWACPH

NO 20
GN ARMC10
IP IPI00420025.2
PE LGIRSSKSAEDLT
MP 44
EX Kim
CL catarrhines
DE armadillo repeat containing 10
SQ
pri Human        IYRLTRGRRRGDRELGIRSS(K)SAEDLTDGSYDDVLNAEQLQ
pri Chimpanzee   IYRLTRGRRRGDRELGIRSS(K)SAEDLTDGSYDDVLNAEQLQ
pri Gorilla      IYRLTRGRRRGDRELGIRSS(K)SAEDLTDGSYDDVLNAEQLQ
pri Orangutan    IYRLTRGRRRGDRELGIRSS(K)SAEDLTDGSYDDVLNAEQLQ
pri Gibbon       IYRLTGGRRRGDRELGIRPS(K)PAEDLTVGSYDDVLNAEQLQ
pri Rhesus       IYRLTRGRRRGDRELGMRPS(K)SAEDLTDGSYDDVLNAAQLQ
pri Baboon       IYRLTRGRRQGDRELGKRPS(K)SAXXXXXXXXXXXXXXXXXX
pri Marmoset     IYRLTRRRRRGDRGLVLRPS(Q)SAEDLTDGSYDDVLNAEQLQ
pri Bushbaby     VYRLTR-RRRGVRGLRLRPS(R)SAXXXXXXXXXXXXXXXXXX
eua Mouse        IYRLTRGPRRGGR--RLRPS(R)SAEDLTDGSYDDILNAEQLK
eua Rat          IYRLTRGPRRGGR--RLRPS(R)SAEDLTEGSYDAILSAEQLE
eua Kangaroo_rat IYRLTRGPRRGGRGRGLRPS(R)SAEDLTNASYDDVLNAEQLK
eua Guinea_pig   LYRLSRGRQRTKR--GVRPS(G)SAEDLTNGSYNDVLNAEQLR
eua Squirrel     IYRLTRGQQRGSGGRWLRPS(R)SAEDLTSDSCDDVLNAEQLQ
eua Rabbit       IYLLTRGRRRGGRGLGPRRS(G)SAEDLTSGSHDDVLNASQLQ
lau Dog          IYKLARGRRRGARGLRLRPS(R)SAEDLTNGSYDDVLNVDQLQ
lau Cat          IYKLTRGQRQGDRGLRLRPS(R)SAEDLTDGSYDDILNVDQLQ
lau Cow          FYKLTRGRRRGGRQLRLRPS(R)SAEDLTSGSYDDTLNAEQVQ
lau Dolphin      FYRLTRGRRRGVPGLRLRPS(R)SAEDLTNGSYDDILNAEQLQ
lau Megabat      IYRLTRGQRRGGRGLRLRPS(R)SAEDLTNGSCDDVVNAEQLQ
afr Elephant     IYRLTRGQRRVGRGPRLRPS(P)SAEDLTDGSYDEVLNAEQLQ
afr Rock_hyrax   IYRLTRGQRRGGRKPRLRPS(R)SAEDLTDGSYDDVLNAEQLK

NO 21
GN ATAD2
IP IPI00170548.1
PE PAATTAKAGDGSS
MP 55
EX Kim
CL simians
DE ATPase family, AAA domain containing 2
SQ
pri Human        GRRRLRSAGAAQKKPAATTA(K)AGDGSSVKEVETYHRTRALR
pri Chimpanzee   GRRRLRSAGAAQKKPAATTA(K)AGDGSSVKEVETYHRTRALR
pri Gorilla      GRRRLRSAGAAQKKPAATTA(K)AGDGSSVKEVETYHRTRALR
pri Orangutan    GRRRLRSAGAAQKKPAATTA(K)AGDGSSVKEVETYHQTRALR
pri Gibbon       GRRRLRSAGAAQKKPAATTA(K)AGDGSSVKEVETYHRTRALR
pri Rhesus       GRRRLRSAGAAQKQPVATTA(K)AGDGSSVKEVGTYHRTRALR
pri Baboon       GRRRLRSAGAAQKQPVATTA(K)AGDGSSVKEVGTYHRTRALR
pri Marmoset     GRRRLRSAGAAQKQPAVTAA(K)AGDGSSVKEVETYHRTRALR
pri Bushbaby     GGRRLRSSSAAA-KPAVTAS(A)AGDGSSVKEVEPYHRTRTLR
eua Mouse        GGRRPRLRRAG--RKSTASK(A)AGDGFTVKGAETYHNTRSLR
eua Rat          GRRRPSLARAAGKSTVPKAE(A)AGDGSSVKGVEICHNTRSLR
eua Guinea_pig   GRRRLRSAGAAE-KAAVTGA(A)KRDGSSVKEVETNHRTRSLR
eua Squirrel     GRRRLRSAGAAX-KGAVTAG(A)PSDGSSVKEVETYHRTRALK
eua Rabbit       GRRRLRSAGAAA-KSAVTAA(A)AGDGSSVKKAESYHRTRALR
lau Dog          GRRRLRSARAAE-QAAVTVA(A)SGDGSSVKEVETYHRTRASR
lau Cow          GRRRLRSAGAAE-QSAVTAA(A)AGDGSSVKEVETYHQTRTLR
afr Rock_hyrax   GRRRLRSAGAAX-KSAVPAA(A)AGDGSSVKEVKTYHQRRTLM
afr Tenrec       GQRRFRSAGAAX-KSAVTAA(A)ASDESSVKGVETYHQKRALR
xen Sloth        GRRRLRSARAAX-KSAVTAA(A)AGVRSSVKEVXXYHQTRALR
mar Opossum      GSRRRQLRSSSAMGKAAATA(S)APTRSSVKEVESF-STHALR

NO 22
GN ATP13A2
IP IPI00015154.1
PE DTAQLHKSEEAVS
MP 150
EX Kim
CL simians
DE ATPase type 13A2
SQ
pri Human        SQAAVGAVPEGAWKDTAQLH(K)SEEAVSVGQKRVLRYYLFQG
pri Chimpanzee   SQAAVGAVPEGAWKDTAQLH(K)SEEAVSVGQKRVLRYYLFQG
pri Gorilla      SQAAVGAVPEGAWKDTAQLH(K)SEEAVSVGQKRVLRYYLFQG
pri Orangutan    SQAAVGAVPEGAWKDTAQLH(K)SEEAVSVGQKRVLRYYLFQG
pri Gibbon       SHVAVGAVPEGAWKDTAQLH(K)SEKAVS---KRVLRYYLFQG
pri Rhesus       SQAAVGAVPEGAWKDTAQLH(K)SEEAVSGR-KRMLRYYLFQG
pri Baboon       SQAAVGAVPEGAWKDTAQLH(K)SEEAVSR--KRVLRYYLFQG
pri Marmoset     SQAAVGVVPEGAWKDTAQLH(K)NEEEVSVGRKRVLRYYLFRG
eua Treeshrew    SQAAVGAVPGGAWKDTAQLH(R)SEEAVSQ--KRVLRYYVFQG
eua Mouse        SQAAVGVTPEGTWQDTSELH(R)QEEAVSQ--KQVLRYYVLQG
eua Rat          SLAAVGVAPESMWQDTTQLH(R)QKEAVSQ--KQVLRYYILQG
eua Guinea_pig   SQVAVGAVPRSVWKDTAQLR(R)REEAVSQ--KRVARYYLFQG
eua Squirrel     SQLAVGATPEGTWKDTAPLH(R)RQEAVSP--TQVLRYYVFQG
eua Pika         SQVAVGAVPEGEWTDTAQLH(T)KTEAVS---TRLLRYYLFQG
lau Dog          SQAAVGAVPEGAWKDTAQFC(K)NEEAVSQ--QRMLRYYLFRG
lau Cat          SQAAVGAVPEGAWKDTTQFC(R)NEEAVSGQ-QRKLRYYLFRG
lau Horse        SQAAVGAVPEGAWKDTAQLH(G)NEEAVSR--QRMLRYYLFQG
lau Cow          SRAAVGAEPEDAWKDTAQLH(R)PEERVRR--QRRLRYYVFRG
lau Dolphin      SQAAVGTLPEGTWKDTARLH(R)TEEAVSQ--QRTLRYYLFRG
lau Megabat      SQAAVGAVPEGTWKDTAQLT(R)NEEAMRQ--QQVLRYYLFQG
lau Microbat     SQAAVGAVPEEAWKDTAQLH(G)SEEVVSQ--QRLLRFYLFRG
afr Elephant     SQAALGAVPEESWKDTTQLH(R)KEEAVSQ--KPVLRYYLFQG
afr Rock_hyrax   SQAAVGAVPEESWKDTVQLH(R)EEETVSQ--EHVLRYYLFRG
mar Opossum      SHMAVGVSQEEAWRDTIQLY(K)KEEV-----QNILRYYVFEG
pro Platypus     NHIAVGVTQEEDWQDTIQLH(R)KEE------KSILRYYVFQG

NO 23
GN ATXN2
IP IPI00180154.4
PE MDSSYAKRDAFTD
MP 349
EX Kim
CL simians
DE ataxin 2
SQ
pri Human        LFKCSDFVVVQFKDMDSSYA(K)RDAFTDSAISAKVNGEHKEK
pri Chimpanzee   LFKCSDFVVVQFKDMDSSYA(K)RDAFTDSAISAKVNGEHKEK
pri Gorilla      LFKCSDFVVVQFKDMDSSYA(K)RDAFTDSAISAKVNGEHKEK
pri Orangutan    LFKCSDFVMVQFKDMDSSYA(K)RDAFTDSAISAKVNGEHKEK
pri Gibbon       LFKCSDFVVVQFKDMDSSYA(K)RDAFTDSAISAKVNGEHKEK
pri Rhesus       LFKCSDFVVVQFKDMDSSYA(K)RDAFTDSAISAKVNGEHKEK
pri Baboon       LFKCSDFVVVQFKDMDSSYA(K)RDAFTDSAISAKVNGEHKEK
pri Marmoset     LFKCSDFVVVQFKDMDSSYA(K)RDAFTDSAISAKVNGEHKEK
pri Tarsier      LFKC-DFVVVQFKDMDSSYA(R)RDAFTDSAISAKVNGEHKEK
pri Bushbaby     LFKCSDFVVVQFKDMDSSYA(R)RXXXXXXXXXXXXXXXXXXX
pri Mouse_lemur  LFKCSDFVVVQFKDMDSSYA(R)RXXXXXXXISAKVNGEHKEK
eua Treeshrew    LFKCSDFVVVQFKDMDSSYA(R)RDAFTDSAISAKVNGEHKEK
eua Mouse        LFKCSDFVVVQFKDTDSSYA(R)RDAFTDSALSAKVNGEHKEK
eua Kangaroo_rat LFKCSDFVVVQFKDMDSSYA(R)RDAFTDSAISAKVNGEHKEK
eua Guinea_pig   LFKCSDFVVVQFKDMDSSYA(R)RDAFTDSAISAKVNGEHKEK
eua Rabbit       LFKCSDFVVAQFKDMDSSYA(R)RDAFTDSALSARVNGEHREK
eua Pika         LFKCSDFVVVQFKDMDSSYA(R)RDAFTDSALSAKVNGEHREK
lau Dog          LFKCSDFVVVQFKDMDSSYA(R)RDAFTDSAISAKVNGEHKEK
lau Horse        LFKCSDFVVVQFKDMDSSYA(R)RDAFTDSAISAKVNGEHKEK
lau Cow          LFKCSDFVVVQFKDMDSSYA(R)RDAFTDSAISAKVNGEHKEK
lau Dolphin      LFKCSDFVVVQFKDMDSSYA(R)RDAFTDSAISAKVNGEHKEK
lau Alpaca       LFKCSDFVVVQFKDMDSSYA(R)RDAFTDSAISAKVNGEHKEK
lau Megabat      LFKCSDFVVVQFKDMDSSYA(R)RDAFTDSAISAKVNGEHKEK
lau Microbat     LFKCSDFVVVQFKDMDSSYA(R)RDAFTDSAISAKVNGEHKEK
lau Hedgehog     LFKCSDFVVVQFKDMDSSYA(R)RDAFTDSAISAKVNGEHKEK
lau Shrew        LFKCSDFVMVQFKDMDSSYA(R)RDAFTDSAISAKVNGEHKEK
afr Elephant     LFKCSDFVVVQFKDMDSSYA(R)RDAFTDSAISAKVNGEHKEK
afr Tenrec       LFKCSDFVMVQFKDMDSSYA(R)RDAFTDSALSAKVNGEHKEK
xen Armadillo    LFKCSDFVVVQFKDMDSSYA(R)RDAFTDSAISAKVNGEHKEK
xen Sloth        LFKCSDFVVVQFKDMDSSYA(R)RDAFTDSAISAKVNGEHKEK
mar Opossum      LFKCSDFVVVQFKDMDSNYA(R)RDAFTDSAISAKVNGEHKEK
pro Platypus     LFKSSDFVMVQFKDMDSNYA(R)RDAFTDSAISAKVNGEHKEK

NO 24
GN AURKB
IP IPI00176642.3
PE NLLLGLKGELKIA
MP 211
EX Kim, Wagner
CL primates
DE aurora kinase B
SQ
pri Human        CHGKKVIHRDIKPENLLLGL(K)GELKIADFGWSVHAPSLRRK
pri Chimpanzee   CHGKKVIHRDIKPENLLLGL(K)GELKIADFGWSVHAPSLRRK
pri Gorilla      CHGKKVIHRDIKPENLLLGL(K)GELKIADFGWSVHAPSLRRK
pri Orangutan    CHGKKVIHRDIKPENLLLGL(K)GELKIADFGWSVHAPSLRRK
pri Gibbon       CHGKKVIHRDIKPENLLLGL(K)GELKIADFGWSVHAPSLXXX
pri Rhesus       CHGKKVIHRDIKPENLLLGL(K)GELKIADFGWSVHAPSLRRK
pri Baboon       CHGKKVIHRDIKPENLLLGL(K)GELKIADFGWSVHAPSLRRK
pri Marmoset     CHGKKVIHRDIKPENLLLGL(K)GELKIADFGWSVHAPSLRRK
pri Tarsier      CHGKKVIHRDIKPENLLLGL(K)GELKIADFGWSVHAPSLRRK
pri Bushbaby     CHGKKVIHRDIKPENLLLGL(K)GELKIADFGWSVHAPSLRRK
pri Mouse_lemur  CHGKKVIHRDIKPENLLLGL(K)GELKIADFGWSVHAPSLRRK
eua Treeshrew    CHGKKVIHRDIKPENLLLGL(Q)GELKIADFGWSVHAPSLRRK
eua Mouse        CHKKKVIHRDIKPENLLLGL(Q)GELKIADFGWSVHAPSLRRK
eua Rat          CHKKKVIHRDIKPENLLLGL(Q)GELKIADFGWSVHAPSLRRK
eua Kangaroo_rat CHGKKVIHRDIKPENLLLGL(Q)GELKIADFGWSVHAPSLRRK
eua Guinea_pig   CHGRKVIHRDIKPENLLLGL(Q)GELKIADFGWSVHAPSLRRK
eua Squirrel     CHGKKVIHRDIKPENLLLGL(Q)GELKIADFGWSVHAPSLRRK
eua Rabbit       CHEKKVIHRDIKPENLLLGL(Q)GELKIADFGWSVHAPSLRRK
eua Pika         CHEKKVIHRDIKPENLLLGL(Q)GELKIADFGWSVHAPSLRRK
lau Dog          CHGKKVIHRDIKPENLLLGL(Q)GELKIADFGWSVHAPSLRRK
lau Horse        CHGKKVIHRDIKPENLLLGL(Q)GELKIADFGWSVHAPSLRRK
lau Cow          CHAKKVIHRDIKPENLLLGL(R)GELKIADFGWSVHAPSLRRK
lau Dolphin      CHGKKVIHRDIKPENLLLGL(Q)GELKIADFGWSVHAPSLRRK
lau Alpaca       CHGKKVIHRDIKPENLLLGL(Q)GELKIADFGWSVHAPSLRRK
lau Megabat      CHGKKVIHRDIKPENLLLGL(Q)GELKIADFGWSVHAPSLRRK
lau Microbat     CHKKKVIHRDIKPENLLLGL(Q)GELKIADFGWSVHAPS-RRK
lau Hedgehog     CHGKKVIHRDIKPENLLLGL(Q)GELKIADFGWSVHAPSLRRK
lau Shrew        CHGKRVIHRDIKPENLLLGL(Q)GELKIADFGWSVHAPSLRRK
afr Elephant     CHGKKVIHRDIKPENLLLGL(R)GELKIADFGWSVHAPSLRRK
afr Rock_hyrax   CHGKKVIHRDIKPENLLLGL(R)GELKIADFGWSVHAPSLRRK
xen Armadillo    CHGRKVIHRDIKPENLLLGL(R)GELKIADFGWSVHAPSLRRK
xen Sloth        CHEKKVIHRDIKPENLLLGP(R)GEVKIADFGWSVHTHSL-RK
mar Opossum      CHKKKVIHRDIKPENLLMGL(R)GELKIADFGWSVHAPSLRRK
mar Wallaby      CHGKKVIHRDIKPENLLMGL(R)GELKIADFGWSVHAPSLRRK

NO 25
GN BBS2
IP IPI00306961
PE NINTLFKIMRVGT
MP 712
EX Wagner
CL apes
DE Bardet-Biedl syndrome 2
SQ
pri Human        NQVITACRDAIRSNNINTLF(K)IMRVGTASS*
pri Chimpanzee   NQVITACRDAIRSNNINTLF(K)IMRVGTASS*
pri Gorilla      NQVITACRDAIRSNNINTLF(K)IMRVGTASS*
pri Orangutan    NQVITACRDAIRSNNINTLF(R)IMRVGTASS*
pri Gibbon       NQVIAACRDAIRSNNINTLF(K)IMRVGTASS*
pri Rhesus       NQVITACRDAIRSNNINTLF(R)IMRVGTASS*
pri Baboon       NQVITACRDAIRSNNINTLF(R)IMRVGTASS*
pri Marmoset     NQVITACRDAIRSNNINTLF(R)IMRGGTASSQERKM*
pri Tarsier      NQVITACRDAIRSNNINTLF(R)IMRVGTASS*
pri Bushbaby     NQVIAACRDAIRSNNINTLF(R)IMRVGTASS*
eua Mouse        NQVISACRDAIRSNNINTLF(R)IMRVGTAPS*
eua Rat          NQVISACRDAIRSNNINTLF(R)IMRVGTAPS*
eua Kangaroo_rat NQVITACRDAIRSNNINTLF(R)IMRVGTAPS*
eua Guinea_pig   NQVITACRDAIRSNNINTLF(R)IMRVGTAPS*
eua Squirrel     NQVISACRDAIRSNNINTLF(R)IMRVGTAPS*
eua Rabbit       NQVITACRDAIRSNNINTLF(R)IMRVGTASS*
eua Pika         NQVITACRDAIRSNNISTLF(R)IMRVGTASS*
lau Dog          NQVITACRDAIRSNNINTLF(R)IMRVGTASS*
lau Cat          NQVITACRDAIRSNNINMLF(R)IMRVGTASS*
lau Horse        NQVITACRDAIRSNNINMLF(R)IMRVGTASS*
lau Cow          NQVITACRDAIRSNNINMLF(R)IMRVGTASS*
lau Dolphin      NQVITACRDAIRSNNINTLF(R)IMRVGTASS*
lau Alpaca       NQVITACRDAIRSNNINTLF(R)IMRVGTASS*
lau Megabat      NQVVTACRDAIRSNNINMLF(R)VMRVGTASS*
lau Hedgehog     NQVISACRDAIRSNNINTLF(R)IMRVGTASS*
lau Shrew        NQVISACRDAIRSNNINTLF(R)IMRVGTASS*
afr Elephant     NQVITACRDAIRSNNINTLF(R)IMRVGTASS*
afr Tenrec       NQVITACRDAIRSNNINTLF(R)VMRAGTASS*
xen Armadillo    NQVITACRDAIRSNNINTLF(R)IMRVGTASS*
xen Sloth        NQVITACRDAIRSNNINTLF(R)IMRVGTASL*
mar Opossum      NQVVSACRDAIRSNNLNMLF(R)IMRVGTASS*
pro Platypus     NQVITACRDAIRNNNLNMLF(R)IMRVGTASS*

NO 26
GN BFAR
IP IPI00009811.1
PE TEEEFSKTPYTIE
MP 225
EX Kim, Wagner
CL simians
DE bifunctional apoptosis regulator
SQ
pri Human        FLSERVNGRLLLTLTEEEFS(K)TPYTIENSSHRRAILMELER
pri Chimpanzee   FLSERVNGRLLLTLTEEEFS(K)TPYTIENSSHRRAILMELER
pri Gorilla      FLSERVNGRLLLTLTEEEFS(K)XPYTIENSSHRRAILMELER
pri Orangutan    FLSERVNGRLLLTLTEEEFS(K)TPYTIENSSHRRAILVELER
pri Gibbon       FLSERVNGRLLLTLTEEEFS(K)TPYTIENSSHRRAILMELER
pri Rhesus       FLSERVNGRLLLTLTEEEFS(K)TPYTIENSSHRRAILMELER
pri Baboon       FLSERVNGRLLLTLTEEEFS(K)TPYTIENSSHRRAILMELER
pri Marmoset     FLSERVNGRLLLTLTEEEFS(K)SPYTIENSSHRRAILMELER
eua Treeshrew    FLSERVNGRLLLTLTEEEFS(R)APYTIENSSHRRAILMELER
eua Mouse        FLSERVNGRLLLTLTEEEFS(R)APYTIENSSHRRVILTELER
eua Kangaroo_rat FLSERVNGRLLLTLTEEEFS(Q)APYTIENSSHRRVILTELER
eua Guinea_pig   FLSERVNGRLLLTLTEEEFS(R)APYAIENSSHRRAILTELER
eua Squirrel     XXXXXXXXXLLLTLTEEEFS(R)APYTIENSSHRRVILMELER
eua Rabbit       FLSERVNGRLLLTLTEEEFA(Q)APYTIENSSHRRAILLELER
eua Pika         FLSERVNGRLLLTLTEEEFA(R)APYAIESSSHRRAILLELER
lau Dog          FLAERVNGRLLLTLTEEEFS(R)APYTIENSSHRRAILLELER
lau Cat          XXXXXXXXXLLLTLTEEEFS(R)APYNIENSSHRRAILLELER
lau Horse        FLSERVNGRLLLTLTEEEFS(R)APYTIESSSHRRAILMELER
lau Cow          FLSERVNGRLLLTLTEEEFS(K)APYTIESSSHRRAILMELER
lau Dolphin      FLSERVNGRLLLTLTXEEFS(R)APYTIENSSHRRAILMELER
lau Alpaca       FLSERVNGRLLLTLTEEEFS(R)APYTIENSSHRRAILMELER
lau Megabat      FLSERVNGRLLLTLTEEEFA(R)APYTIENGSHRRAILMELER
lau Microbat     FLAERVNGRLLLTLTEEEFS(R)APYTIENSSHRRAXLMELER
lau Hedgehog     XXXXXXXXXLLLTLTEEEFS(R)APYTIENNSHRRVILMELER
afr Elephant     FLSERVNGRLLLTLTEEEFS(R)APYTIENSSHRRVILMELER
afr Rock_hyrax   FLSERVNGRLLLTLTEEEFS(R)APYTIENSSHRRVILLELER
afr Tenrec       FLSERVNGRLLLTLTEEEFS(R)APYTIENSSHRRAILMELER
xen Sloth        FLSERVNGRLLLTLTEEEFS(R)VPYTIENSSHRRAIFMELER
mar Opossum      FLSERVNGRLLLTLTDEDFS(K)APYNIENSSHRKAIIMELER
mar Wallaby      FLSERVNXXLLLTLTDEDFS(K)APYNIENSSHRKAIIMELER
pro Platypus     FLSERVNGRLLLTLTDEEFS(K)IPYSIENSSHRKAILMELER

NO 27
GN BFAR
IP IPI00009811.1
PE TKLLDLKEPTWKQ
MP 322
EX Kim, Wagner
CL simians
DE bifunctional apoptosis regulator
SQ
pri Human        ICPLQEDSSGEDIVTKLLDL(K)EPTWKQWREFLVKYSFLPYQ
pri Chimpanzee   ICPLQEDSSGEDIVTKLLDL(K)EPTWKQWREFLVKYSFLPYQ
pri Gorilla      ICPLQEDSSGEDIVTKLLDL(K)EPTWKQWREFLVKYSFLPYQ
pri Orangutan    ICPLQEDSSGEDIVTKLLDL(K)EPTWKQWREFLVKYSFLPYQ
pri Gibbon       ICPLQEDSSGEDIVTKLLDL(K)EPTWKQWREFLVKYSFLPYQ
pri Rhesus       ICPLQEDSSGEDIVTKLLDL(K)EPTWKQWREFLVKYSFLPYQ
pri Baboon       ICPLQEDSSGEDIVTKLLDL(K)EPTWKQWREFLVKYSFLPYQ
pri Marmoset     ICPLQEDSSREDIITKLLDL(K)EPTWKQWREFLVKYSFLPYQ
pri Tarsier      ICPLQEDSPGEDIITKLLDL(R)EPTWKQWREFLVKYSFLPYQ
pri Bushbaby     ICPLQENGSGEDIIAKLLDL(R)EPSWKQWREFLVKYSFLPYQ
pri Mouse_lemur  ICPLQEDSSGEDIITKLLDL(R)EPTWKQWREFLVKYSFLPYQ
eua Treeshrew    ICPLQEDSSGEDILTKLLDL(R)EPTWKQWREFLVKYSFLPYQ
eua Mouse        ICPLQENSSGEDIFTKLLDL(R)EPTWKQWREFLVKYSFLPYQ
eua Kangaroo_rat ICPLQEDSSGEDLLSKLLDL(R)EPTWKQWREFLVKYSFLPYQ
eua Guinea_pig   ICPLQEDSSGDDIITKLLDL(R)EPTWKQWREFLVKYSFLPYQ
eua Squirrel     ICPLQEDSSGEDIVTKLLDL(R)EP-WKQWRE-LVKYSFLPYQ
eua Rabbit       ICPVHEDSAREDVVSRFLDL(R)EPTWKQWREFLVKYSFLPYQ
eua Pika         ICPLQEDGTSEGIITKFLDL(R)DPTWKQWREFLVKYSFLPYQ
lau Dog          ICPLQEDSPGEDILTKLLDL(R)EPTWKQWREFLVKYSFLPYQ
lau Cat          ICPLQEDNSGEDIITKLLDL(R)EPTWKQWREFLVKYSFLPYQ
lau Horse        ICPLPEDGSGEDLITKLLDL(R)EPTWKQWREFLVKYSFLPYQ
lau Cow          ICPLQEDGSGDDIISKLLDL(R)EPTWKQWREFLIKYSFLPYQ
lau Dolphin      ICPLQEDSSGEDIITKLLDL(R)EPTWKQWREFLVKYSFLPYQ
lau Alpaca       ICPLQEDGSGEDILTKLLDL(R)EPTWKQWREFLVKYSFLPYQ
lau Megabat      ICPLQEDSSEEDIIAKLLDL(R)EPTWKQWREFLVKYSFLPYQ
lau Microbat     XXXXXXXXXXXXXXXXXXDL(R)EPTWKQWREFLVKYSFLPYQ
lau Hedgehog     XXXXXXXXXXXXXXXXXXDL(R)EPTWKQWREFLVKYSFLPYQ
afr Elephant     ICPLQEDDSGEDIITKLLDL(R)EPTWKQWREFLVKYSFLPYQ
afr Rock_hyrax   VCPLQEDDSGEDLITRLLDL(R)EPTWKQWREFLVKYSFLPYQ
afr Tenrec       ICXXHEDDLGEDIISKLLDL(R)EPTWKQWREFLVKYSFLPYQ
xen Armadillo    ICPLQEDDAVEDILTKLLDL(R)EPTWKQWREFLVKYSFLPYQ
xen Sloth        ICPLQEDDSGEEIITKLLXL(R)EPTWKQRGEFFVKYNFLPYZ
mar Opossum      ICPVQEXXXXEDLITKFLDF(K)EPTWKQWREFLVKYSFLPYQ
mar Wallaby      ICPIQEXXXXEDIITKFXDF(K)EPTWKQWREFLVKYSFLPYQ
pro Platypus     ICPVQEXXLEEDIITKLLDL(K)DPSWKQWREFLVKYSFLPYQ

NO 28
GN BIRC2
IP IPI00013418.1
PE REEEKEKQAEEMA
MP 448
EX Kim
CL great apes
DE baculoviral IAP repeat containing 2
SQ
pri Human        NDIVSALLNAEDEKREEEKE(K)QAEEMASDDLSLIRKNRMAL
pri Chimpanzee   NDIVSALLNAEDEKREEEKE(K)QAEEMASDDLSLIRKNRMAL
pri Gorilla      NDIVSALLNAEDEKREEEKE(K)QAEEMASDDLSLIRKNRMAL
pri Orangutan    NDIVSALLNAEDEKREEEKE(K)QAEEMASDDLSLIRKNRMAL
pri Rhesus       NDIVSALLNAEDEKREEEKE(R)QAEEMASDDLSLIRKNRMAL
pri Baboon       NDIVSALLNAEDEKREEEKE(R)QAEEMASDDLSLIRKNRMAL
pri Marmoset     NDIVSALLNAEDEKREEEKE(R)QAEEMASDDLSLIRKNRMAL
pri Bushbaby     NDIVSALLNAEDEKREEEKE(R)QAEEMASDDLSLIRKNRMAL
eua Treeshrew    NDIVSALLSAEDEKREEEKE(R)QAEELASDDLSLIRKNRMAL
eua Mouse        NDIVSVLLNAEDERREEEKE(R)QTEEMASGDLSLIRKNRMAL
eua Rat          SDIVSALLNAEDERREEEKE(R)QSEETASGDLSLIRKNRMAL
eua Guinea_pig   NDIVSELLNAEDERREEEKE(R)QAEEMASDDLSLIRKNRMAL
eua Rabbit       NDIVSALLNAEDEKREEEKE(R)QAEEVASDDLSLIRKNRMAL
eua Pika         NDIVSALLNAEDEKREEEKE(R)QAEEMASDDLSLIRKNRMAL
lau Dog          NEIVLALLNAEDETREEEKE(R)QTEEMASDDLSLIRRNRMAL
lau Cat          RDIVSDLLNAEDEIREEEKE(R)ATEEKESDDLSLIRRNRMAL
lau Horse        NDIVSALLNAEDEKREEEKE(R)QTEEMASDDLSLIRRNRMAL
lau Dolphin      NDIVSALLNAEDEKREEEKE(R)QTEETASDDLSLIRRNRMAL
lau Alpaca       NDIVSALLNAEDEKREEEKE(R)QTEEVASGDLSLIRKNRMAL
lau Megabat      NDIVSALLNAEDEKRQEEKE(R)QTEEMASDDLSLIRKNRMAL
lau Microbat     NDIVSALLHAEDENRQEEKE(R)QTEEMASDDLSLIRKNRMAL
lau Hedgehog     NDIVSALLNAEDEKREEEKE(R)QIEEVASDDLSLIRRNRMAL
afr Elephant     NDIVSALLNAEDEKREEEKE(R)QTEELASDDLSLIRKNRMAL
afr Rock_hyrax   NDIVSALLDAEDEKREEEKE(R)QTEEMASDDLALIRKNRMAL
xen Sloth        SDLVFDLLNAEDEKREEEKE(R)QTEEMASDDLSLIRKNRMAL
mar Opossum      NEVVSDLLNAEDEKRKEEKE(R)QTEEMASDDLTLIRKNRMAL
mar Wallaby      NEVVSDLLNAEDEKRKEEKE(R)QTEEMASDDLTLIRKNRMAL
pro Platypus     NDLVSDLLNAEDETREEEKE(R)QNEEMASDDLSLIRKNRMAL

NO 29
GN BSG
IP IPI00019906.1
PE ITDSEDKALMNGS
MP 148
EX Kim
CL African great apes
DE basigin (Ok blood group)
SQ
pri Human        SESVPPVTDWAWYKITDSED(K)ALMNGSESRFFVSSSQGRSE
pri Chimpanzee   SESVPPVTDWAWYKITDSGD(K)ALMNGSESRFFVSSLPGRSK
pri Gorilla      SESVPPVTDWAWYKITDSGD(K)ALTNGSESRFFVSSSQGXSE
pri Orangutan    SESVPPVTDWVWYKITDSGD(Q)VIMNGSESRFFVSSSQGRSE
pri Rhesus       SESLPPVTTWVWYKITDSGD(Q)VIVNGSQGRFFVSSSQGRSE
pri Baboon       SESLPPVTNWVWYKITDSGD(Q)VIVNGSQGRFFVSSSQSRSE
pri Marmoset     SKSAPPVTDWVWYRITDAGD(Q)VIVNGSQSRFFVNSSQGRSE
pri Mouse_lemur  SSSLPPVTDWVWFKAEDSGD(Q)VIVNGSQNKFFVSSSEAKSE
eua Guinea_pig   SDSHPPIDTWVWYKVVDSEV(Q)VITNGSNNKFFVSSSETQTQ
eua Squirrel     SDSYPPVTDWAWYKMVDSHY(Q)VITNGSQSKFYVSSSEAKSE
lau Dog          SDSFPPVTDWVWYKVESSGD(Q)VISNSSQSKFLVVSSETKTE
lau Cat          SDSFPPVSDWVWYKMESSGD(Q)VISNSSQNKFFVVSSETKTE
lau Horse        STSFPPVTDWFWYKISESAD(Q)VITNTSQGKFLVTSSESKTE
lau Cow          SDSFPPVTNWLWYKESESGD(Q)XITNSTQSKFFVVSSESRTE
afr Rock_hyrax   SESYPDVTEWLWFRKSDTTE(Q)DIVNGSQSXFVVSSSESQTE

NO 30
GN BSN
IP IPI00020153.4
PE SKEAGPKPLGSGP
MP 398
EX Kim
CL simians
DE bassoon (presynaptic cytomatrix protein)
SQ
pri Human        APSKGTPKIVFNDASKEAGP(K)PLGSGPGPGPAPGAKTEPGA
pri Chimpanzee   APSKGTPKIVFNDASKEAGP(K)PLGSGPGPGPAPGAKTEPGA
pri Gorilla      APSKGTPKIVFNDASKEAGP(K)PLGSGPGPGPAPGAKTEPGA
pri Orangutan    APSKGTPKIVFNDASKEAGP(K)PLGSGPGPGPAPGAKTEPGA
pri Gibbon       APSKGTPKIVFNDASKEAGP(K)PLGSGPGPGPAPGAKTEPGA
pri Rhesus       APSKGSPKIVFSDASKEAGP(K)PLGLGPGPGPAPGAKTEPGA
pri Baboon       APSKGSPKIVFSDASKEAGP(K)PLGLGPGPGPAPGAKTEPGA
pri Marmoset     APSKGTPKIVFSDASKEAGP(K)PSGSVPGPGPAPGAKTEPGA
pri Mouse_lemur  APSKGPPKIVFSDASKEAGP(R)PPGSGPGPGPAPGAKTEPGT
eua Treeshrew    APSKGPPKIVFSDASKEAGP(R)PLGSGPGPGPAPGAKTEPGA
eua Mouse        SPSKGQPKIVFSDASKEAGP(R)PPGSGPGPGPTPGAKTEPGA
eua Rat          SPSKGPPKIVFSDASKEAGP(R)PPGSGPGPGPTPGAKTEPGP
eua Squirrel     SPNKGPPKIVFSDASKEAGP(R)PPGSWPGPGPAPGAKTEPGA
eua Rabbit       APSKGPPKIVFSDASKEAGP(R)PPGSGPGPGPVSGAKTEPGA
eua Pika         APSKGPPKIVFSDASKEAGP(R)PPGSGPGPGPAPAAKTEPGA
lau Dog          APSKGPPKIVFSDASKEAGP(R)PPGSGPGP--LPGAKTEPGA
lau Cat          APSKGPPKIVFSDASKEAGP(R)PPGSGPGPGPAPGAKTEPGA
lau Horse        APSKGPPKIVFSDASKEAGP(R)PPGSGSGPGSAPGAKTEPGA
lau Cow          APSKGPPKIVFSDASKEAGP(R)PPGSGPGPGPTTGAKTEPG-
lau Dolphin      APSKGPPKIVFSDASKQAGP(R)PPGSGPGSGPAPGAKTEPGA
lau Megabat      APTKGPPKIVFSDASKEAGP(R)TPGSGPGP--ALGAKTELAA
lau Hedgehog     ALSKGPPKIVFSDASKETGP(R)HPSSGPGLGPAPGAKTEPGA
afr Elephant     SPSKGQPKIILSDASKEAGS(R)PPGSGTRPGPVPGAKTEPGA
afr Rock_hyrax   SPSKGQPKIVFSDASKEAGS(R)PPGSGTGPGPAPGAKTEPGA
afr Tenrec       SPSKGQPKLVFSDASKEAGS(R)APGSGPGVGPAPGAKTEPGT

NO 31
GN BTN2A2
IP IPI00013576.2
PE RITFVSKDINRGS
MP 105
EX Kim
CL primates
DE butyrophilin, subfamily 2, member A2
SQ
pri Human        GRERTEEQMEEYRGRITFVS(K)DINRGSVALVIHNVTAQENG
pri Chimpanzee   GRERTEEQMEEYRGRITFVS(N)DINRGSVALVIHNVTAQENG
pri Orangutan    GRERTEEQMEQYRGRITFVS(K)DISRGSVALVIHNVTAQENG
pri Gibbon       GRERTEEQMEEYRGRITFVS(K)DIIKGSVALVIHNVTTQENG
pri Rhesus       RRERTEEQMEEYRGRITFVS(K)DISRGSVALVIHNVTTQENG
pri Marmoset     GRERTEEQMEEYRGRTTFVS(K)GISRGSVALVIHNVTAQENG
pri Mouse_lemur  GRERTEEQMEEYRGRTTLVS(K)DISRGKVALVIHNVTARENG
eua Mouse        HQERPEEQMVAYRGRTTFMR(T)DISKGRVALIIHNVTAYDNG
eua Squirrel     RRARAEEQMEEYRGRTTFLS(R)DLSKGGAALVIRNVTAQENG
eua Rabbit       GRERPEEQMEEYRGRTTFVS(E)DIGRGSVALVIHNLTAREDG
lau Dog          GRERTEEQMEEYQGRTTFVS(E)HINKGSVALIIHNVTAHDNG
lau Horse        GRERTEEQMKEYRGRTTFVK(E)EISKGSVALIIHNVTAHENG
lau Cow          RRERTDEQMEQYRGRTTFVS(E)AISEGSVGLIIHNVTAHENG
lau Dolphin      HRERTEEQMEEYRGRTTFVS(E)AISKGSVGLIIHNVTAHDNG
lau Shrew        GQERNEEQMAAYRGRTTFVS(E)NIQRGRVALVIHNVTAHENG
afr Elephant     GRERAEEQMEAYQGRTTFVS(N)DISKGRVALIIHNVTTHENG
xen Armadillo    GRERPEEQMQQYRGRTSLEG(A)GLGRGHAALVIHNVTAHEDG
mar Opossum      GKNRDEEQMEEYRSRTTFMR(D)NIKDGSVALKIHNVTAFENG
mar Wallaby      GKEREEEQMREYQGRTTFMR(D)NIRDGKVALKIHNVTAFENG
pro Platypus     GQDQAGEQMGEYRGRTELLK(D)AINDGSLTVKIRDVRVSDDG

NO 32
GN C16orf42
IP IPI00011693.4
PE VLSPVGKQYASPA
MP 103
EX Kim, Wagner
CL simians
DE chromosome 16 open reading frame 42
SQ
pri Human        LVRCLRLGHRFGGLVLSPVG(K)QYASPADRQLVAQSGVAVID
pri Chimpanzee   LVRCLRLGHRFGGLVLSPVG(K)QYVSPADRQLVAQSGVAVID
pri Gorilla      LVRCLRLGHRFGGLVLSPVG(K)QYVSPADRQLVAQSGVAVID
pri Orangutan    LVRCLRLGHRFGGLVLSPVG(K)QYVSPADRQLVAQSGVAVID
pri Rhesus       LVRCLRLGHRFGGLVLSPVG(K)QYVSPADRQLVAQSGVAVID
pri Baboon       LVRCLRLGHRFGGLVLSPVG(K)QYVSPADRQLVAQSGVAVID
pri Marmoset     LVRCLRLGRRFGGLVLSPVG(K)QYVSPADRQLVAQSGIAVID
pri Mouse_lemur  LVRCLRLGHRFGGLVLSPVG(S)QYVSPADRQLVAQSGVAVID
eua Mouse        LVRCLRLSQRFGGLVLSPVG(T)EYVSPADRQLVAQSGVAVID
eua Rat          LVRCLRLSQRFGGLVLSPVG(T)QYVSPADRQLVAQSGIAVID
eua Guinea_pig   LVRCLRLGHRFGGLVLSPVG(S)LYVSPADRQLVAQAGVAVID
eua Squirrel     LVRCLRLGQRFGGLVLSPMG(T)QYVSPADRQLVAQSGVAVID
eua Pika         VVRCLRLGHRFGGLVLSPLG(T)QLVSPADRQLVAQAGVAVID
lau Dog          LVRCLRLGQRFGGLVLSPVG(S)QYVSCADRQLVAQCGVAVID
lau Horse        LVRCLRLGHRFGGLVLSPMA(S)QYVSPADRQLVAQSGVAVID
lau Cow          LVRCLRLGHRFGGLVLSPVG(S)QYVSPADRHLVAQSGVAVID
lau Dolphin      LVRCLRLGHRFGGLVLSPVG(S)QYVSPADRQLVAQSGVAVID
lau Alpaca       LGCCLGLGPRSSSLVLSLVG(A)QHMSHTD-QLGTLSGVPVID
lau Megabat      LVRCLRLGHRFSGLVLSPMG(S)QYVSPADRQLVAQSGVAVID
lau Microbat     LVRCLRLGHRFGGLVLSPMG(S)QYVSPAD--LVAQSGVAVID
afr Elephant     LVHCLRLGHRFGGLVLSPVG(A)QYVSPKDRQLVAQSGVAVID
afr Rock_hyrax   LVRCLRLGHRFGGLVLSPVG(V)QYVSPADRQLVARSGVAVID
afr Tenrec       LVRCLLLGHRFGGLVLSPVG(T)QYVSPADRELVAQSGVAVID
xen Armadillo    LVRCLRLGHRFGGLVLSPAG(A)QYVSPADRQLVAQLGVAVID
xen Sloth        LVRCLRLGHRFGGLVLSPAA(A)QYVSPADRQLVAQSGVAVID
mar Opossum      LVRTLRLNQRFNGLILSPMG(T)HYVSPADKQLVAQCGVAVID
mar Wallaby      LVRTLRLNQRFNGLILSPMG(T)HYVSPADKQLVAQWGVAVID

NO 33
GN C1orf124
IP IPI00552744.1
PE IKKEQIKSSGNDP
MP 428
EX Kim
CL apes
DE chromosome 1 open reading frame 124
SQ
pri Human        RPRLEDKTVFDNFFIKKEQI(K)SSGNDPK---YSTTTAQNSS
pri Chimpanzee   RPRLEDKTVFDNFFIKKEQI(K)SSGNDPK---YSTTTAQNSS
pri Gorilla      RPRLEDKTVFDNFFIKKEQI(K)SSGNDPK----YSTTAQNSS
pri Orangutan    RPRLEDKTVFDNFFIKKEQI(K)SSGNDPK---YSTTTAQNSS
pri Gibbon       RPRLEDKTLFNNFFIKREQM(K)SSGNDPK---CSTTTAQNSS
pri Rhesus       RPRLEDKTVFDNFFIKKEQI(E)SSGNDPK---YSTTTTQNSS
pri Baboon       RPRLEDKTVFDNFFIKKEQI(E)SSGNDPK---YSTTTTQNSS
pri Marmoset     RPRLEDKTVFDNFFIKKERI(E)SRGNDPN-----YSTAQNSS
pri Tarsier      RPRLEGKTVSDNFFVKKKQM(Q)SGDNDPK---NSATTVQNFN
pri Bushbaby     RPRLEDKTVFDNFFIKKEQI(Q)SDGNDAK---CSVTPSQNSG
pri Mouse_lemur  RPRLEDKTVFDNFFIKKEQM(Q)SDGNDPK---CSITTTQNSN
eua Mouse        RPRLEDRTALDTI---KEQT(Q)SGGDLRS--SSQPTAASAPQ
eua Rat          RPRLEDRTALNNI---KEQT(Q)SGGDLED--SSRPTAISTPR
eua Kangaroo_rat RPRLEAKTAFDHFFIKKEQA(H)GPGDAER----SGASTPAAS
eua Guinea_pig   RPRLEDKSTFDTFLM-KEQV(P)SDGREPDSTSHSAAAAESSS
eua Squirrel     RPRLEDKTILDNFFIKKEQI(Q)KGGSDVKSSSHPATASQSSS
lau Dog          RPRLEDKTVFDNFFIKKEQV(Q)SGGNDPKWSSHPTAATQDSS
lau Cat          RARLEDQSVSDELFI-KEQV(Q)RGGSDSKWSSCPPAAAQSSS
lau Horse        RPRLEDKTVFDKFLIKKEQI(R)SGGNEPKCSSHPTAAAQNSS
lau Cow          RPRIEDKTFFDLFFIKKEQA(Q)SGGGDVTSSSHPPAAAQSPS
lau Dolphin      RPRLEDRTVFDNLFIAKEQT(Q)SGGNDPK---CGIAATQNAN
lau Microbat     QRRREDKTAFDNFFIKKEKT(R)SGGGDPQCSSRPAAATPSSS
lau Hedgehog     RVKLEDKTVFDSFFIKREQL(K)NAGNHPICSSYPVSTIQNSS
afr Elephant     RPRLEDKTLFDSFFIKKEQL(Q)SGGSDPKCNSHPTTSAQNSS
xen Sloth        RPRLEDKNVFANFFIKKEPT(Q)SAGNDLQ---YSATSAQNSS
mar Opossum      RPKIENKTAFANFFIKKEQI(N)SNGNGTN---CSVTSSQNSS
mar Wallaby      RPKVENKTDFANFFIKKKHV(N)NNGNDSK---CSVTSSQNSS

NO 34
GN C3orf75
IP IPI00107155.2
PE VVFQAQKEPHPLQ
MP 99
EX Kim
CL apes
DE chromosome 3 open reading frame 75
SQ
pri Human        GQLVFLEGLKSAVDVVFQAQ(K)EPHPLQFLREANAGNLKPLF
pri Chimpanzee   GQLVFLEGLKSAVDVVFQAQ(K)EPHPLQFLREANAGNLKPLF
pri Gorilla      GQLVFLEGLKSAVDIVFQAQ(K)EPHPLQFLREANAGNLKPLF
pri Orangutan    GQLVFLEGLKSAVDVVFQAQ(K)EPQPLQFLREANAGNLKPLF
pri Gibbon       GQLVFLEGLKSAVDVVFQAQ(K)EPHPLQFLREANAGNLKPLF
pri Rhesus       GQLVFLEGLKSAVDVFFQTQ(E)EPHPLQFLREANAGNLKPLF
pri Baboon       GQLVFLEGLKSAVDVFFQTQ(E)EPHPLQFLREANAGNLKPLF
pri Marmoset     GQLVFLEGLKSAVDVFFQAQ(E)KPHPLQFLREASAGNLKPLF
pri Tarsier      GQLVFLEGLKSSVDVFFQAQ(K)EPHPLQFLXXXXXXXXXXXX
pri Bushbaby     GQLVFLEGLKSSMDVFFQAH(K)EPHPLQFLREVNAGNLQPLF
pri Mouse_lemur  GQLVFLEGLKSSVDVFFQAN(E)EPHPLQFLREANPGNLQPLF
eua Treeshrew    GQLVFLEGLKSSVDVFFQAQ(E)KTHPLHFLREANAGNLQPLY
eua Mouse        GQLVFLEGLKSSVEVLFHSQ(D)EPHPLQFLREAGTGNLQSLY
eua Rat          GQLVFLEGLKSSVEVLFHSQ(E)EPHPLQFLREAGAGNLQSLY
eua Kangaroo_rat GQLVFLEGLKSSVDIFFHPQ(E)EPHPLRFLREANAGSLQPLY
eua Guinea_pig   GQLVFLEALKTSVDIFFQPP(V)EPHPLQFLREASAGNLQPLF
eua Squirrel     GQLVFLEGLKSSVDVFFQAQ(E)EPHPLQFLREASAGNLEPLY
eua Rabbit       GQLVFLEGLKSSVDVFFQAQ(E)DPHPLQFLREASAGNLQPLY
eua Pika         GQLVFLEGLRSSVDIFFQAQ(E)DPHPLQFLREASAGDLKPLY
lau Dog          GQLVFLEGLKSSVDVFFRAQ(A)EPHPLQFLREADAGNLQPLY
lau Cat          GQLVFLEGLKSSVDVFFRAQ(A)EPHPLQFLREANAENLQPLY
lau Horse        GQLVFLEGLKSSVDVFFRSQ(E)EPHPLQFLREANTGNLQPLF
lau Cow          GQLVVLEGLKSAVDIFFRPQ(E)EPHPLQFLREANAGDLQPLY
lau Dolphin      GQLVFLEGLKSAVDVFFRPQ(E)EPHPLQFLXXXXXXXXXXXX
lau Alpaca       RQLVFLEGLKSAVDIFFRPQ(E)EPHPLQFLREANTRNLQPLY
lau Megabat      GQLVFFEGLKSSVDVFFRVK(G)EPHPLQFLREASAGNLRPLY
lau Microbat     GQLVFLEGLRSAVDVFFRAE(G)EAHPLHFLREAKAASLQPLY
lau Hedgehog     GQLVFLEGLKSSVEVFFQTP(E)EPHPLQFLREAKAGNLQPLY
lau Shrew        GQLVFFEGLKAAVDVIFRTP(E)EPQPLQFLREATAGSLLPLY
afr Elephant     GQLVFLEGLKSSVDVFFRAQ(E)EPHPLQFLREANAETLQPLY
afr Rock_hyrax   GQLVFLEGLKSSVDVFFRAQ(K)EPHPLQFLREASAETLQPLY
afr Tenrec       GQLVVLEGLKSSVDVFFRAQ(E)EPHPLQFLRKASAGDLQPLY
xen Sloth        RQLVFLEGLKSSVELFFRAQ(E)ETHPLQFLREANTGNLQPLY

NO 35
GN C7orf44
IP IPI00414548.1
PE LNIHYLKLIDREN
MP 74
EX Kim
CL simians
DE chromosome 7 open reading frame 44
SQ
pri Human        LQSHPEAQEALGPPLNIHYL(K)LIDRENFVDIVDAKLKIPVS
pri Chimpanzee   LQSHPEAQEALGPPLNIHYL(K)LIDRENFVDIADAKLKIPVS
pri Gorilla      LQSHPEAQEALGPPLNIHYL(K)LIDRENFVDIADAKLKIPVS
pri Orangutan    LQSHPEAQEALGPPLNIHYL(K)LIDRENFVDIADAKLKIPVS
pri Gibbon       LQSHPEAQEALGTPLNIHYL(K)LIDRENFVDIADAQLKIPVS
pri Rhesus       LQSHPEAQEALGPPLNIHYL(K)LTDRENFVDIADAKLKIPVS
pri Baboon       LQSHPEAQEALGPPLNIHYL(K)LTDRENFVDIADAKLKIPVS
pri Marmoset     LQIHPGAQEALGPPVSVHHL(K)LTGKDNFVDIADAKLKIPVS
pri Tarsier      LHGHPEAQEALGAPLNVHHL(R)LTDKHNFVDIADAKLKIPIS
pri Bushbaby     LHGHPEAREALGTPLNVHHL(Q)LMDKYNFVDIADAKLKIPVS
pri Mouse_lemur  LHSHPEALEALGTPVNVHNL(K)LPDKQNFVDIAEAQLKIPVS
eua Treeshrew    LHNHPTALEALGAPLSVHRI(Q)LLDKHNLVDIADAQLKIPVS
eua Guinea_pig   LHGHPEALEALGSPLRVHHL(P)MLSRDHFVDITDARLKIPIS
eua Squirrel     LHSHPEVLEALSIPLHVHYL(Q)PPSKHNFVDITVAWLKIPVF
eua Pika         LRSHPEALEALGRPLNIHHL(Q)LTDRHNFVDIADAKLKIPVS
lau Dog          LHSRPEALEALGPPLHIHYL(H)LTDKYNFVDIADAQLKIPVS
lau Horse        LHSRPEALEALGPPLSVHYL(H)LTDRYNFVDIADAQLKIPVS
lau Cow          LHSHPEALEALGTPLNVHYL(R)LTDKYNFVDIADAKLKIPVS
lau Dolphin      LHSHPEALEALGTPLNVHYL(R)LTDKYNFVDIADAKLKIPVS
lau Microbat     LHSHPEALEALGTPLNTHYL(Q)LMDKYNFVDIADAKLKIPVS
lau Hedgehog     LRNHSGALEVLGTPLNVHYL(Q)LTDRDNFVDIADAKLKIPVS
lau Shrew        LNNNPGALEALGTPLNVHYL(Q)LIDKYNFVDITNAQLKIPVS
xen Armadillo    LQSHPEAREALGPPLNTHYL(H)LTDRENFVDIAYAQLKIPVS
pro Platypus     LKRQPAVLEALGPPLKVHHI(S)LTDGHNRIDVSGAQIKVPVS

NO 36
GN C9orf114
IP IPI00418229.1
PE EEAAAEKEDRGRP
MP 66
EX Kim
CL catarrhines
DE chromosome 9 open reading frame 114
SQ
pri Human        QRAQEEQAKRL-EEEEAAAE(K)EDRGRPYTLSVALPGSILDN
pri Chimpanzee   QRAQEEQAKRQ-EEEEAAAE(K)EDRGRPYTLSVALPGSILDN
pri Gorilla      QRAQEEQAKRL-EEEEAAAE(K)EDRGRPYTLSVALPGSILDN
pri Orangutan    QRAQEEQAKRQ-EEEEAAAE(K)EDRGRPYTLSVALPGSILDN
pri Gibbon       QRAQEEQEKRQ-EEEEAVAE(K)EDRGRPYTLSVALPGSILDN
pri Rhesus       QRAQEEQAKRQ-EDEEAAAE(K)EDRXXXXXXXXXXXXXXXXX
pri Baboon       QRAQEEQAKRQ-EDEEAAAE(K)EDRGRPYTLSVALPGSILDN
pri Marmoset     QRAQEEQAKRQ-EEEEAAAE(R)EEHGRPYTLSVALPGSILDN
pri Bushbaby     QRAQMEQAKRQ-EEEEAATQ(R)DDHGRPYTLSVALPGSILDN
pri Mouse_lemur  QRAQEEQAKRQ-EEEEAXXQ(R)EDRGRPYTLTVALPGSILDN
eua Treeshrew    QRAQEEQAKQQ-EEEAAAAE(R)QDQGRPYTLSVALPGSILDN
eua Mouse        QRAQEEEAKRQEEEEEAAAQ(R)SNQGRPYTLSVALPGSILDN
eua Rat          QRAQEKQAKRQEEEEEAAAQ(R)SNQGRPYTLSVALPGSILDN
eua Guinea_pig   QRAQEEQAKRQKEEEEAAAQ(R)EDLGRPYTLSVALPGSILDN
eua Squirrel     QRAQEEQTKRQ-EEEEAAAQ(R)ENQGRPYTLSVALPGSILDN
eua Rabbit       QRAQEAAAKQ--QEEEAATR(A)EDRGRPYTLSVALPGSILDN
eua Pika         QQTQEAAAKX--QAEEAASQ(E)EDRGRLYTLSVALPGSILDN
lau Dog          QQAQEEQAKLQ-QEEEAAAQ(R)EDQGRPYTLSVALPGSILDN
lau Cat          QRAQEEQAKRQ-QEEEAAAQ(R)EDQGRPYTLSVALPGSILDN
lau Horse        QRAQEEQAKCQ-QEEEAAAQ(G)EDRGRPYTLSVALPGSILDN
lau Cow          QRAQEEQAKRQ-QEEEAAAQ(S)EDRGRHYTLSVALPGSILDN
lau Dolphin      QRVQEEQAKRQ-QEEEAAAQ(R)EDRGRHYTLSVALPGSILDN
lau Megabat      QRMQEEQTKRQ-EEEEAAAQ(R)EDQGRRYTLSVALPGSILDN
lau Microbat     QRAQEEQTKRQ-EEEEAAAQ(R)EDQGRPYTLSVALPGSILDN
afr Elephant     QRAQEELAKRQ-EEEEAALQ(K)EDHGRPYTLSVALPGSILDN
afr Tenrec       QRALEEQALQ--EEQEAALH(R)EDQGRPYTLSVALPGSILDN

NO 37
GN CAPG
IP IPI00027341.1
PE EDLTADKANAQAA
MP 243
EX Kim
CL catarrhines
DE capping protein (actin filament), gelsolin-like
SQ
pri Human        VLGPKPALKEGNPEEDLTAD(K)ANAQAAALYKVSDATGQMNL
pri Chimpanzee   VLGPKPALKEGNPEEDLTAD(K)ANAQAAALYKVSDATGQMNL
pri Gorilla      VLGPKPALKEGNPEEDLTAD(K)ANAQAAALYKVSDATGQMNL
pri Orangutan    VLGPKPALKEGNPEEDLTAD(K)ANAQAAALYKVSDATGQMNL
pri Gibbon       VLGPKPALKEGNPQEDLTAD(K)ANAQAAALYKVSDATGQMNL
pri Rhesus       VLGPKPALKEGNPEEDLTAD(K)ANAQAAALYKVSDATGQMNL
pri Baboon       VLGPKPALKEGNPEEDLTAD(K)ANAQAAALYKVSDATGQMNL
pri Marmoset     VLGPK-ALKEGNPEEDLTAD(Q)TNSQAAALYKVSDATGQMNL
pri Bushbaby     VLGPKPALKEGNPEEDLRAD(Q)TNAQAAALYKVSDATGQMNL
pri Mouse_lemur  VLGPKPALKEGNPEEDLTAD(Q)TNAQAAALYKVSDATGQMNL
eua Mouse        VLGPKPALKEGNPEEDITAD(Q)TNAQAAALYKVSDATGQMNL
eua Rat          VLGPKPALKEGNPEEDITAD(Q)TNAQAAALYKVSDATGQMNL
eua Kangaroo_rat VLGPKPALKEGNPEEDLTAD(Q)TNAQAAALYKVSDATGQMNL
eua Guinea_pig   VLGPKPALKEGNPEEDLTAD(Q)TNAQAAALYKVSDATGQMNL
eua Rabbit       VLGPKPPLKEGNPEEDLTAD(Q)TNAQAAALYKVSDATGQMNL
eua Pika         VLGPKPPLKEGNPEEDLTAD(Q)TNAQASALYKVSDATGQMNL
lau Dog          VLGPKPALKEGNPEEDLTAD(R)TNAQAAALYKVSDATGQMNL
lau Cat          VLGPKPALKEGNPEEDLTAD(R)TNAQAAALYKVSDATGQMNL
lau Horse        VLGPKPALKEGNPEEDLTAD(K)TNAQAAALYKVSDATGQMHL
lau Cow          VLGPKPSLKEGNPEEDLTAD(R)TNAQAAALYKVSDATGQMNL
lau Dolphin      VLGPKPALKEGNPEEDLTAD(Q)TNAQAAALYKVSDATGQMSL
lau Alpaca       VLGPKPALKEGNPEEDLTAD(R)TNAQAAALYKVSDATGQMNL
lau Hedgehog     VLGPKPALKEGNPEEDLTAD(Q)TNAQAAALYKVSDATGQMNL
lau Shrew        VLGPKPVLKEGNPEEDLTAD(Q)TNAQAAALYKVSDATGQMNL
afr Elephant     VLGPKPSLKEGNPEEDLTAD(R)TNAQAAALYKVSDATGQMNL
afr Tenrec       VLGPKPALKEGNPEEDLTAD(Q)TNAQAAALYKISDATEQMNL
xen Armadillo    VLGPKPALKEGDPEEDLTAD(R)TNAQAAALYKVSDATGQMNL
mar Opossum      VLGSKPTLKEGNPEEDLRAD(Q)TNAQAAALYKVSDATGQMHL
mar Wallaby      ILGSKPTLKEGNPEEDLRAD(Q)TNAHAAALYKVSDATGQMHL
pro Platypus     VLGPKPALREGNPEEDLTAD(Q)TNAHAAALYKVSDATGKMDL

NO 38
GN CASC5
IP IPI00163659.6
PE QMHVSLKEDENNS
MP 262
EX Kim
CL humans
DE cancer susceptibility candidate 5
SQ
pri Human        PIYSKEPNSASSTHQMHVSL(K)EDENNSNITRLFREKDDGMN
pri Chimpanzee   PIYSKEPNSASSTHQMHVSL(N)EDENNSNITRIFREKDDGMN
pri Gorilla      PIYSKEPNSASSTHQMHVSL(N)EDENNSNITRIFREKDDGMN
pri Orangutan    PVYSKEPNSASSTHQMHISL(N)EDENNSNITRIFREKDDGMN
pri Gibbon       PIYSNEPNSASSTHQMHVSL(N)EDENNSNITRIFREKDDGMN
pri Rhesus       PVYSNESNSASSIHQMHISL(N)KDENNSNVTRIFREKDDGMN
pri Baboon       PVYSNESNSASSIHQMHISL(N)KDENNSNVTRIFREKDDGMN
pri Marmoset     PVYSDESRNAFSTRHVHVSL(N)EDESNSNITRIFREKDDGMN
pri Tarsier      PVYSKESNSSSSTHKMHVSL(N)IDENNSNMTRIFREQDDGMN
eua Treeshrew    PIYSRESGSASSTQQMNLSL(N)VDENISNRTRIFREQDDGMN
eua Mouse        PVYSKDSNSASSTYQMHASL(G)VDENSSNRTRIFREQDDGMN
eua Rat          PVHSKDSNSASSTCQIHASL(S)VDEHSSNKTRLFTEQDDGMN
eua Kangaroo_rat PIYSKQPSSASSTHQMQASL(H)VNENSSNITRIFREQDDGMN
eua Guinea_pig   PIFSKKSDNTPSTYQICTSF(N)VDEKSSNITRVFREQDDGMN
eua Rabbit       PIYSKEANSASSIYQMYVPV(N)VAENSSNMTRIFREQDDGMN
eua Pika         PIHSKESSSASCIPQTHVPV(N)IDENSNDRTQIFREQDAGMN
lau Dog          SVHPIKTKKASSVHQMHVSL(R)VDENSSNTTRIFGEQNDGMN
lau Cat          SFHPKESNRASSVHQMHVSL(R)VDENSNNMTRIFREQDDEMN
lau Horse        SVHAKESNRASSIHQMHVSV(N)TDENSSNMTRIFREQDDGMN
lau Cow          PIHSKEANKTTSVHQMHVSL(G)VDENTRNMTRLFREQDDGMN
lau Dolphin      PIHYKESNRASSMHQMHVSL(S)VDANSSNMTRLFREQDDGMN
lau Alpaca       PIHSKESNRTSSIHQMHVSV(N)VDESSSNMTRIFREQDDGMN
lau Microbat     PIHSIESNKASSIHQKHVSL(N)VDENNSNMTRIFIEEDDGMN
lau Hedgehog     PVYYKDSNRGSAIHQKHVAF(N)ADENNSNMTKILREQDDVMN
lau Shrew        LTYSKNSNRTSTMYQKHASL(N)VEENSTNMTRIFRDQDDGMN
afr Elephant     FVHAKGSENASSIHQMGVSL(N)VDENSSNRTRIFREQDDGMN
afr Rock_hyrax   YVHVKESKNASSTHQMDVSL(N)VDENRSNRTKIFKEQDDRMN
afr Tenrec       SVHANESNNASSIHQMHVSL(N)VDEDNRNRTRIFREQDDVMN
xen Armadillo    MVHCKESNRASSVHQVHVSL(N)VEEDNSNVTRIFREQDDEMN
xen Sloth        MVHSKESNSASSVHQMHISL(N)VGENNNNITRIFREQDDEMN

NO 39
GN CCDC138
IP IPI00065415.2
PE KVGSSLKYSDESK
MP 66
EX Kim
CL simians
DE coiled-coil domain containing 138
SQ
pri Human        LTSPGDLDIY-SGDKVGSSL(K)-YSDESKHCRTPLGSLFKHV
pri Chimpanzee   LTSPGDLDIY-SGDKVGSSL(K)-YSDESKHCRTPLGGLFKHV
pri Gorilla      LTSXGDLDVY-SGDKVGSXL(K)-YSDESKHCRTPLGSLFKHV
pri Orangutan    LTSPGDLEIY-SGDKIGSSL(K)-YSDESKHCRTPLGSLFKHV
pri Gibbon       LTSPGDLDIY-SGDKVGSSL(K)-YSDESKHYRTPLCSLFKHA
pri Rhesus       LTSPGDLDIY-SGDKVGSSL(K)-YSDESKHCRTPLCSLFKRV
pri Baboon       LTSPGDLDIY-SGDKVGSSL(K)-YSDESKHCRTPLCSLFKRV
pri Marmoset     LTSPDDLDVC-SGDKVGSSL(K)-YSDESKHCRKPFCSSFKHI
pri Tarsier      LAAPVDWDIY-SGDKVGSSL(R)-YFDDGKHCRMPLCSSLKHF
pri Bushbaby     LTSLEDLDIY-SGDKVVSSL(R)-YSDESKHHRTPFCSSFKHL
pri Mouse_lemur  PTSPEDLDIY-SGDKAVSSL(R)-YSAESKRCTTPFGSSFKHL
eua Treeshrew    LTSPDDLDTY-SGDKVGPSL(R)-YSDERKHCTTPFRSSFKHL
eua Mouse        VTSPDGLDTYSSGDKVGSSP(R)YYSDGRNHPTPPFCSSFKHL
eua Rat          LTSPDDLDTYSSGDKVGSSP(R)YYSDERKHPITSLCSSFKHL
eua Guinea_pig   LTYPDDVDIYCSGDKVSPSV(R)YYSKERKHCVMPLCSSFKHL
lau Dog          LISPDDLDIFSSGDKVGSSL(R)CHSDERKHCTTPLCSSFKLL
lau Cow          RELGEDLDIFCCGDKVGPSL(R)CFSDERRRRPTSPRGVXXXL
lau Horse        LTSPDDLAIYSSGDKVGSSL(R)CYSDERKHYTTPLCSSFKYL

NO 40
GN CCDC14
IP IPI00396060.5
PE ETIEPDKTYENVL
MP 681
EX Kim
CL simians
DE coiled-coil domain containing 14
SQ
pri Human        SFTHSEPLSTIKNEETIEPD(K)TYENVLSSRGPQNSNTRGME
pri Chimpanzee   SFTHSEPLSTIKNEETIEPD(K)PYENVLSSGGPQNSNTRGME
pri Gorilla      SFTHSEPLSTIKNEETIEPD(K)PYENVLSSRGPQNSNTRGME
pri Orangutan    SFTPSEPLSTIKNEETIEPD(K)PYENVLSSRGPQNSNTRGME
pri Gibbon       SFTHSEPLSTIKNEETIEPD(K)PY--VLSSRGPQNSNTRGME
pri Rhesus       SFTHSEPQSTIKNEETIEPD(K)PYENVLSSRSPQNSNTRGTE
pri Baboon       SFTHSEPLSTIKNEETIEPD(K)PYENVLSSRSPQNSNTRGME
pri Marmoset     SFIYSESLSTIKNEATIEPD(K)PYENVLPSRSPQHSNTRDME
pri Tarsier      SFIHSEPLSMIKNEEITELD(R)PYENVQPSKGLQHSNTRDME
pri Bushbaby     TFTHSQPLSTIKTEETLEPG(R)LYEDTLPSRSPQPGHTRPME
eua Guinea_pig   TFMYSEPLSAVNREETTEPI(R)SCENVLPSRGSPVGKNRVVE
eua Rabbit       SVAHLEPLSTIKNEETTEPD(R)PYENVLPSKGPQPRNTSGRE
eua Pika         SFAHLEAQSTVQNEEAIGPD(G)PSESVVPSRGPHPNNTRGRG
lau Dog          NFTHSEPLSAVNTEENIEPD(R)PYENALPSKGPQQSNTKSME
lau Cat          AFIHSQPLSTVNNEENIEPG(R)PYENVLPSKSPQHGNTRNKE
lau Horse        TFTRSELLSTIDNEENMVPD(R)PYENVLPPKGPQPRDTKNME
lau Cow          TLTHPEPVSTIDSEENMVPD(R)PYENVLPSKGPQHSNTRSME
lau Dolphin      TITRSEPLSTINNEENTMPD(R)PYENVLPSEGPQHTNARNME
lau Alpaca       SITHSEPLSAINNEKNIVRG(R)PYENVMPSKGSQHSNTRSME
lau Megabat      TFTHSEPLSTVNDEKNIVPD(R)PYESVLPSKGPQHSNTSSTE
lau Microbat     TFTHSEPLSAINNAENIVPD(R)PYENVLPSKSPQHSNTRKME
afr Elephant     IFTHSEPLCAVKNEENMVPG(R)RHETALPSKVPQHGNTENME
afr Rock_hyrax   IFTRSEPLSAVTDKEDVVPD(R)HPENDLPSKTPQHGNTRGME
xen Sloth        IFTHPEPLNADKNVENIVPD(R)PYEHVLPSKDIQHSSSRSME
mar Opossum      IYTQTESPFLKEDGKGRVPA(M)SYENFLNPEVCKHNSWVDHE

NO 41
GN CCDC14
IP IPI00396060.5
PE SRASDMKDTQLLK
MP 811
EX Kim
CL primates
DE coiled-coil domain containing 14
SQ
pri Human        SSSTKEAEDAPEKLSRASDM(K)DTQLLKKIKEAIGKIPAATK
pri Chimpanzee   SSSTKKAEDAPEKLSRASDM(K)DTQLLKKIKEAIGKIPAATK
pri Gorilla      SSSTKEAEDAPEKLSRASDM(K)DTQLLKKIKEAIGKIPAATK
pri Orangutan    SSSTKEAEDAPEKLFRASDM(K)DTQLLKKIKEAIGKIPAATK
pri Rhesus       SSSTKEAEDAPEKLSRASDM(K)DTQLLNKIKEVIGKIPAATK
pri Baboon       SSSTKEAEDAPEKLSRACDM(K)DTQLLNKIKEAIGKIPAATK
pri Marmoset     SSSTKEAEYVAEKLSRTSDI(K)DIQLLKKIKEAIGKIPAATE
pri Tarsier      SSLIKEVEDTPEKLSRAADT(E)DKQLLKKIKEAIGKIPVATE
pri Bushbaby     SSSIKEAENGPEELSRTADM(K)DKQLLKKIKEVICKIPAATK
eua Mouse        SS-RNEAEDAPGDLSSTYDT(E)DVQLLRKIKEAIGKIPAAAE
eua Rat          PS-RDKAEDAPGNLSGTYDT(E)DVQLLRKIREALGKIPAAAG
eua Squirrel     SS-KNETEDAPEKLSRTTDT(E)DKQLLKKIKEAIGKIPVAAE
eua Rabbit       SS-RQEVEDAHEKLSRTADT(E)DKQLLKKIKEAIGKIPVARE
eua Pika         S-LRQEVEDAPEKLSRTADM(E)DKQLLEKIKEAIGKIPVARV
lau Dog          SSSKKEAEDAPEKLPRTADM(K)DKQLLKKIKEAICKIPAAPE
lau Cat          SSSKKEAEDTPEKLSRTPDM(E)DKQLLKKIKEAICKIPAAPE
lau Horse        SSSKKEAEVAPEKLSRTADM(E)DKHLLEKIKEALCKIPAATG
lau Cow          SSCQKE--DAPDKLCRTADL(E)DNQLLKKIKEAICKIPPAPE
lau Dolphin      SSSKEEG--APEKLSRTADM(E)DKQLLRIIKEAIRKIPPATE
lau Alpaca       SSSKKEA--VPEKLSRTADM(E)DKQLLKKIKEAICKIPAATK
lau Microbat     S--KKEAEDAPEKLSRTAEM(E)DKQLLKKIKEAIGKIPAAIE
lau Hedgehog     SFSKKEAEDTPEKLSRAADL(E)DKQLLKKIKEVIGKIPSATE
afr Elephant     SSSKKEAEDAPEELSRTDDI(E)DRQLLKKIKEAIGKIPATTE
afr Rock_hyrax   S--KREADDAPEKLSGTDTV(Q)DRQLLKKIKEAISKIPAATE
afr Tenrec       SSPKADAEDVPEKLSRIADM(E)DRQLLKKIQEAIGKIPAVTE
xen Sloth        SSSKKEAEDALEKLPRQNDT(E)DKQLLEKIKEAIGKIPAVPE
mar Opossum      STLKRETEVISEKM-RAVNV(E)DEQLLMKIKEVISKIPTDFG
mar Wallaby      SSLKSEAEVVSEKI-RAVNV(E)DEQLLVKIKEVISKIPADFV

NO 42
GN CDC25B
IP IPI00029734.1
PE LEKEEEKDLVMYS
MP 328
EX Kim
CL African great apes
DE cell division cycle 25 homolog B (S. pombe)
SQ
pri Human        PGMESLISAPLVKTLEKEEE(K)DLVMYSKCQRLFRSPSMPCS
pri Chimpanzee   PGMESLISAPLVKTLEKEEE(K)DLVMYSKCQRLFRSPSMPCS
pri Gorilla      PGMESLISAPLVKTLEKEEE(K)DLVMYSKCQRLFRSPSMPCS
pri Orangutan    PGMESLISAPLVKTLEKEDE(Q)DLVMYSKCQRLFRSPSMPCS
pri Gibbon       PGMESLISAPLVKTLEKEEE(Q)XXXXXXXXXXXXXXXXXXXX
pri Rhesus       PGMESLISAPLVKTLQKEEE(Q)DLVMYSKCQRLFRSPSMPCS
pri Baboon       PGMESLISAPLVKTLQKEEE(Q)DLVMYSKCQRLFRSPSMPCS
pri Marmoset     PGMESLISAPLVKTLEKEEE(Q)DLIMYSKCQRLFRSPSMPCS
pri Tarsier      PGKVSFISSPLVKTLGKEEE(Q)DLVMYSKCQRLFRSPSMPCS
pri Bushbaby     PGMESLISAPLVKTLEKEEE(Q)DLIIYSKCQRLFRSPSMPCS
eua Treeshrew    PGMESLISAPLVKTLEKEEE(Q)DLIMFSKCQRLFRSPSMPCS
eua Mouse        AGMENLISAPLVKKLDKEEE(Q)DLIMFSKCQRLFRSPSMPCS
eua Rat          AGMENLISAPLVKKLDKEEE(Q)DLIMFSKCQRLFRSPSMPCS
eua Kangaroo_rat PGMESLISAPLVKTLDKEEE(Q)DLMVFNKCQRLFRSPSMPCS
eua Guinea_pig   PGMESLISAPLVKTLEKEEE(Q)DLVMFSKCQRLFRSPSMPCT
eua Pika         PGMESLISAPLVKIVDKQEE(Q)DLVMFSKCQRLFRSPSMPCS
lau Dog          PGMESLISAPLVKTSEKEEE(Q)DLIMYSKCQRLFRSPSMPCS
lau Cat          PGMESLISAPLVKTSEKEEE(Q)DLIIYSKCQRLFRSPSMPCS
lau Horse        PGMESLISAPLVKTSEKEEE(Q)DLIMHSKCQRLFRSPSMPCS
lau Cow          PGMESLISAPLVKTSEKEEE(Q)DLIMYSKCQRLFRSPSMPCG
lau Dolphin      PGMESLISAPLVKTSEKEEE(Q)DLIMYSKCQRLFRSPSMPCS
lau Megabat      PGMESLISAPLVKTSEKEEE(Q)DLVMYSKCHRLFRSPSMPCG
lau Hedgehog     PGMESLISAPLVRTSEKEKE(Q)DLIMYSKCRRLFRSPSMPCS
afr Elephant     PGMESLISAPLVKTSEKEGE(Q)DLIMYSKCQRLFRSPSMPCR
afr Rock_hyrax   PGMENLISAPLVKTSEKEGD(Q)DLVMYSKCQRLFRSPSMPCS
afr Tenrec       PGMESLISAPLVKTLEKELE(Q)XXXXXXXXXXXXXXXXXXXX

NO 43
GN CDK5RAP1
IP IPI00328278.1
PE TFQHFLKSASAPQ
MP 70
EX Kim, Wagner
CL catarrhines
DE CDK5 regulatory subunit associated protein 1
SQ
pri Human        GARKDFSSRLAAGPTFQHFL(K)SASAPQEKLSS-EVEDPPPY
pri Chimpanzee   GARKDFSSRLAAGPTFQHFL(K)SASAPQEKLSS-EVEDPPPY
pri Gorilla      GARKDFSSRLAAGPTFRHFL(K)SASAPQEKLSS-EVEDPPPY
pri Orangutan    GAQKDFSSRLATGPTFQHFL(K)SASAPQEKLST-EVEDPPPY
pri Gibbon       GARKDFSSRLAAGPTFQHFL(K)SASAPQEKLSS-EVEDPPPY
pri Rhesus       GARKDFSSRLAAGPTFQHFL(K)SASAPQEKLSS-EVEDPPPY
pri Baboon       GAPKDFSSRLAAGPTFQHFL(K)SASAPQEKLSS-EVEDPPPY
pri Marmoset     GARKDFSSRLATGPTLQYFL(R)SASAPQEKLSS-EVEDPPPY
pri Bushbaby     GVQKDFSSRLATGPTFQHFL(R)SASAPQEKLSS-EVEDLPPY
eua Treeshrew    GVPKDFSSRLATGPTFQHFL(R)SASAPQEKVSS-EEEDPPPY
eua Mouse        EAQKDFSSRLATGPTFQHFL(R)SASVPQEKPSSPEVEDPPPY
eua Rat          GVQKDFSSRLATGPTFQHFL(R)SASVPQEKPSSPEVEDPPPY
eua Kangaroo_rat GEQKDFSSRLATGPTFQHFL(R)NTSAPQEKPSL-ELEDPPPY
eua Squirrel     GVHKDFSSRLATGPTFQHFL(R)-ASAPQEKPSS-EAEDPPPY
eua Rabbit       GVRKDFSSRLAAGPAFQHFL(R)NASASEEKPSL-EAEDPPPY
eua Pika         RVRKDFSSRLADGPTFQHFL(R)NALPSEEKPPW-EVADSPPY
lau Dog          GVRKDFSSRLATGPTFQHFL(R)SASAPQEKP---DMEDPPPY
lau Cat          GVRKDFSSRLAAGPTFQHFL(R)SALDPQEKP---DVEDPPPY
lau Horse        GVQKDFSSRLATGPTFQHFL(R)NASVPQENLSS-DVEDPPPY
lau Cow          GVWKDFSSRLATGPTFQDFL(R)SASVPPEQPSS-EVEDPPPY
lau Dolphin      GVWKDFSSRLATGPTFQHFL(R)SASVPQEKPST-DVEDPPPY
lau Alpaca       GVWKDFNSRLATGPTFQHFL(R)NASVPQEKP---EVDDPPPY
lau Megabat      GVRKDFSSRLATGPTFQHFL(R)SASVPQEKLLS-EVEDPPPY
lau Microbat     GVRKDFSSRLATGPTFQHFL(R)SASVPQEKLAS-DVVDPPPY
afr Elephant     GVPKDFSSRLATGPTFQDFL(R)SASGPQEEPSS-EAEDPPPY
afr Rock_hyrax   EVPKDFSSRLAAGPTFQHFL(R)NASAPQEKQSS-EAGDPPPY
afr Tenrec       GVLKDFSTRLLTGPTFQHFL(S)SASAPQEKLPS-EAEDPPPY
xen Armadillo    EVQKDFSSRLATGPTFQHFL(R)SVSVPQEKLSS-EAEDPPPY
mar Opossum      RLQRDFSSRLAAGPTFQHFL(R)NASVPQEKSSS-TVEDPPPY
mar Wallaby      GVQRDFSSRLAAGPTFQHFL(R)NASTPQEKSSS-TVEDPPPY

NO 44
GN CENPF
IP IPI00855998
PE KTHLQEKLQSLEK
MP 2132
EX Wagner
CL catarrhines
DE centromere protein F, 350/400kDa (mitosin)
SQ
pri Human        LQTLSSDVSELLKDKTHLQE(K)LQSLEKDSQALSLTKCELEN
pri Chimpanzee   LQTLSSDVSELLKDKTHLQE(K)LQSLEKDSQALSFTKCELEN
pri Gorilla      LQTLSSDVSELLKDKTHLQE(K)LQSLEKDSQALSLTKCELEN
pri Orangutan    LQTLSSDVSELLKDKTHLQE(K)LQSLEKDSQALSLTKCELEN
pri Gibbon       LQTLSSDVSELLKDKTHLQE(K)LQSLEKDSQSLSLTKCELEN
pri Rhesus       LQTLSSDVSELLKDKTHLQE(K)LQSLEKDSQALSLTKCELEN
pri Baboon       LQTLSSDVSELLKDKTHLQE(K)LQSLEKDSQALSLTKCELEN
pri Tarsier      LQNLSSDVNELLKDKTRLQE(Q)LQSLEKDSQALSLVKCELEN
pri Bushbaby     LQTLSSDVSQLLKDKAHLQE(Q)VHSLEKDSQALSLVKCELEI
pri Mouse_lemur  IQTLSSDVSQLLKEKAHLQE(Q)LQSLEKDSQALSLVKCELEI
eua Treeshrew    LQTLSSDVSGLLKDKTHLQE(Q)LQNLEEDSQALSLVKRELEN
eua Mouse        IQTLSFNVGELTKDKAHLQE(Q)LQNLQNDSQELSLAIGELEI
eua Rat          IQTLSFSVDELTKDKAHLQE(Q)LQNLQNDSQGLSLAIGELEI
eua Kangaroo_rat LQTLSSEVSKLSEDKAHLQE(Q)LQSGQKASETLSLAACELES
eua Guinea_pig   LEALSSQVSQLLEDKGDLQE(W)LQRLEGDSQALSSAVQELES
eua Squirrel     LQTLSSDVSELLKDKAHLQE(Q)LQNLEEDSQALSLAKCELEN
eua Rabbit       VQTLSSEVNKLLKDKTHLQE(Q)LQGLEKESQAWSLIKCELEN
eua Pika         IPTSSSDESEWLKDKAHLQE(Q)LQSSERGLQALSLIKCELEH
lau Dog          VQRLSSDVTQLLEDNTRLQE(Q)LQSLEKDSQALSLVKSELEI
lau Cat          LQTLPSNVSELLEENARLQE(Q)LQSLEKDSQALSLVKGELEI
lau Horse        LQTLSSEVSELLEDKGHLQE(Q)LQSLEKDSQTLSLVKSELEN
lau Megabat      LQTLSSDVSELLKDKTHLQE(Q)MQSLEKDSQALSSVKSELES
afr Rock_hyrax   LQASSSEADRLGQEKAQLLG(Q)LRGLEEASLALSLLKAELEN
afr Tenrec       LQTLSSDANGLLKEKADLQV(Q)LHTLEQDSQVLSLVKGDLED
xen Armadillo    LQTLSSDTSELLKDKARLQE(Q)LQSLERDSQALSLVTGDLEN
xen Sloth        LQTLSSDTSELLKDKADLQE(Q)LQHLERDSQELSLVKAELEN
mar Opossum      LQTMSSDVNQLLKDKDYLQE(Q)LQNLEKDAQMLSLIKDGLKN
mar Wallaby      IQTMSSDVNQLLKEKDHLQE(Q)LQSLEKNTQVLSLAKDGLKN
pro Platypus     VQALSSDINHLSRDKDCLQS(Q)LQNLDKTMQAFILEKEELQK

NO 45
GN CENPN
IP IPI00305656.3
PE VDLFDMKQFKNSF
MP 102
EX Kim
CL primates
DE centromere protein N
SQ
pri Human        VWEVFQMSKGPGEDVDLFDM(K)QFKNSFKKILQRALKNVTVS
pri Chimpanzee   VWDVFQMSKGPGEDVDLFDM(K)QFKNSFKKILQRALKNVTVS
pri Gorilla      VWDVFQMSKGPGEDVDLFDM(K)QFKNSFKKILQRALKNVTVS
pri Orangutan    VWDVFQMNKGPGEDVDLFDM(K)QFKNSFKKILQRALKNVTVS
pri Gibbon       VWDVFQMSKGPGEDVDLFDM(K)QFKNSFKKILQRALKNVTVS
pri Rhesus       VWDVFQMSKGPGEDVDLFDM(K)QFKNSFKKILQRALKNVTVS
pri Baboon       VWDVFQMTKGPGEDVDLFDM(K)QFKNSFKKILQRALKNVTVS
pri Marmoset     IWDVFQMSKEPGEDVDLFDM(K)QFKNSFKKILQRALKNVTVS
pri Tarsier      IWDVFRMSKEPGDDVDLFDM(N)QFKSSFKKVLQRALKNVTVS
pri Bushbaby     MWDVFQMSKGPDEDVDLFDM(K)QFKSTFKKILQRALKNVTVK
eua Treeshrew    GWDVFQMSKE-GEDVDLFDM(E)QFKSSFKKILQRALKNVTVS
eua Mouse        LWNVFQMSKEPGEDVDLFDM(E)QFQSSFKRILQRALKNVTVS
eua Rat          LWNVFQMSKEPGGDVDLFDM(E)QFQSSFKRILQRALKNVTVS
eua Kangaroo_rat VWDVFQMSEEPDDDVNLFDM(E)QFKSSFKKTLQRALRNVTVS
eua Guinea_pig   IWNVFHMNRKPDEEVDIFDM(E)EFKRTFKKILHRALKNVTVS
eua Rabbit       IWDVYQMSKGPGEDLDLFDI(E)QFKSSFKKILQRALRNVTVS
eua Pika         IWDVYQMSKEPGEDPDLFDI(E)QFKSSFKKILQRALKNVTVS
lau Dog          VWDVFQMSKGPGEDIDLFDM(E)QFKSSFKKILQRALKNVMVS
lau Cat          VWDVFQMSKEPGEDVDLFDM(E)RFKSSFEKILRRALKNVTVS
lau Horse        VWDVFRMSREPGEDIDLFDM(E)QFKRSFKKILQRALKNVTIS
lau Cow          IWDVFQMSKAPGDDIDLFDM(E)QFKSSFKKILQRALKNVTVS
lau Dolphin      VWDVFQMSKGPGEDTDLFDM(E)QFKSSFKKILKRALKNVTVS
lau Alpaca       VWDVFQMSRRPGEDIDLFDM(E)QFKVHFKKILXXXXXXVTVS
lau Megabat      VWDVFQMTKEPGEDTDLSDM(E)QFKSSFKKILQRALKNVTVT
lau Microbat     VWDVFQMTKEPGEDIDLFDM(K)QFKSSFKRILQRALKNXXXX
lau Hedgehog     IWDVFQMSKEPGEDSDLFDM(E)QFKSSFKRILQRALKNXXXX
lau Shrew        VWDVFQMNRLPGEDSDLFDM(E)QFKQXXXXXXXXXXXXVTVS
afr Elephant     LWDVFKMNKGPGEDIDLFDI(E)QFKSSFTKILRKALKNVTVS
xen Sloth        IWDVFHMSKGQGEDIDLFDM(K)QFKSSFKKILQRALKNXXXX
mar Opossum      IWTVYEMTKFPGEEVDLSDM(E)EFKDSFKKILQRNLRNVHVS
mar Wallaby      IWIVYKMTKAPGEEVDLFDM(E)EFKDSFKKVLQRTLKNVHIS
pro Platypus     IWDVYRMSKAPEEDIDLFDQ(E)EFKRTFKRILQAALKNVTIS

NO 46
GN CEP250
IP IPI00160622
PE CQQKLIKELEGQR
MP 1577
EX Wagner
CL African great apes
DE centrosomal protein 250kDa
SQ
pri Human        CLALELEENHHKMECQQKLI(K)ELEGQRETQRVALTHLTLDL
pri Chimpanzee   CLALELEENHHKMECQQKLI(K)ELEGQRETQRVALTHLTLDL
pri Gorilla      CLALELEENHHKMECQQKLI(K)ELEGQRETQRVALTHLTLDL
pri Orangutan    CLALELEENHHKMECQQKLI(E)ELEGQRETQRVALTHLTLDL
pri Gibbon       CLALELEENHHKMECQQKLI(E)ELEGQRETQRVALTHLTLDL
pri Rhesus       CLALELEENHHKMECQQKLI(K)ELEGQRETQRVALTHLTLDL
pri Baboon       CLALELEENHHKMECQQKLI(K)ELEGQRETQRVALTHLTLDL
pri Marmoset     CLALELEENHHKMECQQKLI(E)ELEGQREAQRVALTHLTLDL
pri Bushbaby     CLALELEENHHKMECQQKLI(E)ELEGQREMQRVALTH-----
pri Mouse_lemur  CLALELEENHRKMECQQKLI(E)ELEGQREMQRVALTHLTLDL
eua Treeshrew    CLALELEENRHKLECQQKVI(E)ELEGQRERQRVALTHLTLDL
eua Mouse        CLALELEESHHKVESQQKMI(T)ELEGQREMQRVALTHLTLDL
eua Rat          CLALELEESHRKVEGQQKVI(T)ELEGQRELQRVALTHLTLDL
eua Kangaroo_rat HLAMEVEESHHKVESLQKMI(E)ELEGQREMQRVALTHLTLDL
eua Guinea_pig   CLALELQESHHRVESQQKVI(E)ELEGQREMQRVALTHLTLDL
eua Rabbit       CLALELEESHHRVECQHKVI(E)ELEGQREMQRAALTQLTLDL
eua Pika         CLALELEESQHKVECQQKVI(G)ELEGQQEMQRVALTHLTLDL
lau Dog          CLALEREENHHKMECQQKAI(E)ELEGQREMQRVALTHLTLDL
lau Cat          CLALELEENHHKMECQQKAI(E)ELEDQREVQRLALTHLTLDL
lau Horse        CLALELEENHHKMECQQKAI(E)ELEGQREMQKVALTHLTLDL
lau Cow          CLALELEEKRHKTESQQKAI(E)ELEGQREAQRVALTHLTLDL
lau Dolphin      RLALDLEENHHKVECQQKAI(E)ELEGQREMQRVALTHLTLDL
lau Megabat      CLALELEENHHKIECQEKAI(E)ELEAQREMQRVALTHLTLDL
lau Microbat     CLALELEENHRKVECQQKAI(E)ELESQREVQRVALTHLTLDL
afr Elephant     CLALELEENHHKMECQQKAI(E)ELEGQREMQRVTLTHMTLDL
afr Rock_hyrax   CLALELEENHHKMECQQKAI(E)ELEGQREIQRVTLTHMTLDL
mar Opossum      HLVLEVKKNHQEIESQKKKI(E)DLENQREMQRVTLTHLTLDL
mar Wallaby      HLGLEVKENHQEIEYQKKKI(E)DLENQREMQRSTLTHLTLDL

NO 47
GN CEP290
IP IPI00784201
PE DLEQQIKILKHVP
MP 2324
EX Wagner
CL simians
DE centrosomal protein 290kDa
SQ
pri Human        EATEREQKVNKYNEDLEQQI(K)ILKHVPEGAETEQGLKRELQ
pri Chimpanzee   EATEREQKVNKYNEDLEQQI(K)ILKHVPEGAETEQGLKRELQ
pri Gorilla      EATEREQKVNKYNEDLEQQI(K)ILKHVPEGAETEQGLKRELQ
pri Orangutan    EATEREQKVNKYNEDLEQQI(K)ILKHVPEGAETEQGLKRELQ
pri Gibbon       EATEREQKVNKYNEDLEQQI(K)ILKHVPEGAETEQGLKRELQ
pri Rhesus       EATEREQKVKKYTEDLEEQI(K)ILKHVPEGAETEQGLKQELQ
pri Baboon       EATEREQKVKKYTEDLEEQI(K)ILKHVPEGAETEQGLKQELQ
pri Marmoset     EATEREQKVKKYTEDLEQQI(K)ILKHVPEGAETEQGLKQELQ
pri Tarsier      DATERELKVKKYTKDLEQQI(E)ILKHVPEGPETEQGVKRELQ
pri Bushbaby     EATEREQKVKKHTEDLEQQI(E)ILKHIPEGAETDQGLKRELQ
eua Treeshrew    EATEREQKVKKYTEDLEQQI(E)ILKHVPEAAETEEGLKRELH
eua Mouse        EATEREQKAKKYTEDLEQQI(E)ILKNVPEGAETEQELIRELQ
eua Rat          EATEREQKAKKYTEDLEQQI(E)ILKNVPEGAETEQELIQQLQ
eua Kangaroo_rat EATEREQKVKKYTEDLEQQI(E)ILKHVPEGAETEQSLKQQLQ
eua Guinea_pig   EAEEKEQKIKKYTEDLEQQI(E)ILKQIPEDAETEEGLKRELQ
eua Squirrel     EATEREQKVKKYNEDLEQQI(E)ILKHVPEGTETEQGLKRELQ
eua Pika         EATEREQKAKKYAEDLERQI(E)VLKCVPDDIKTEEDLKRELQ
lau Dog          QATEREQKAKKYTEDLEQQI(A)ILKHVPEGAETEQSLQRELQ
lau Horse        QAAEREQKVKKYTEDLEQQI(E)ILKHIPEGAETEQGLQRELQ
lau Cow          QATEREQRVKKYTADLEQQI(E)ILKHVPEGDETEQGLQRELR
lau Dolphin      QATEREQKVKKYTEDLEQQI(E)ILKHVPEGDETEQGLQWELQ
lau Alpaca       QATEREQKVKKYNEDLKQQI(E)ILKRVPEGDETEQGLQQELQ
lau Megabat      QATEREQKVKKYTEDLEQQI(E)ILKHIPEGAETEQGLQRELQ
lau Hedgehog     ESREREQKAKKYTEDLQQQI(E)ILKHVPEGVETEQGLQREFQ
afr Elephant     EATEREQKVKKYTEDLEQQI(E)ILKHVPEGAKTEQVLNRELQ
afr Rock_hyrax   EAAEREQKLKRYTEDLEQQV(E)ILKHVPEGDKTEQGLNQELQ
mar Opossum      EATEREEEIKKYNQDLEEQT(E)ILKNFPEGSHQEQSLRRELQ
pro Platypus     EATERERKITECTQDLKEQI(E)LLKQFPEDAKTDQGLHRELQ

NO 48
GN CHPF
IP IPI00465319.5
PE PGAEREKPGAGEG
MP 73
EX Kim
CL simians
DE chondroitin polymerizing factor
SQ
pri Human        RGNTNAARRPNSVQPGAERE(K)PGAGEGAGENWEPRVLPYHP
pri Chimpanzee   RGNTNAARRPNSVQPGAERE(K)PGAGEGAGENWEPRVLPYHP
pri Orangutan    RGNTNAARRPNSVQPGAERE(K)PGAGEGAGENWEPRVLPYHP
pri Gibbon       RGNTNAARRPNSVQPGAERE(K)PGAGEGAGENWEPRVLPYHP
pri Rhesus       RGNTNAARRPNSVQPGAERE(K)PGTGAGAGENWEPRVLPYHP
pri Baboon       RGNTNAARRPNSVQPGAERE(K)PGTGAGAGENWEPRVLPYHP
pri Marmoset     RGNTNAARRPNSVQPGAERE(K)PGAGAGAGENWEPRVLPYHP
pri Bushbaby     RGNSNAARRPNSVHPGAERE(R)PGDGAGAGENWEPRV-PYHP
eua Treeshrew    RGNTNAARRPNSVQPGAQRE(R)PGA---VGENWEPRVLPYHP
eua Mouse        RGNTNAARRPNSVQPGSERE(R)PGAGAGTGESWEPRVLPYHP
eua Rat          RGNTNAARRPNSVQPGAERE(R)PGA----DESWEPRVLPYHP
eua Kangaroo_rat RGNTNAARRPNSVQPGAERE(R)PGAGEGTGENWEPRVLPYHP
eua Pika         RGNTNAARRPNSVQPGAERD(R)PGAAGGAGENWEPRVLPYRP
lau Dog          RGNTNAARRPNSVQPGAERD(R)PAAG--AAESWEPRVLPYHP
lau Cat          RGNTNAARRPNSVQPGAERE(R)PGVGAGAGENWEPRVLPYHP
lau Horse        RVNTNAGRRLNSVQPGAERE(R)PGAGAGAGENWEPRVLPYHP
lau Cow          RGNTNAARRPNSVQPGAERE(R)PGAGAGAGENWEPRVLPYHP
lau Dolphin      RGNTNAARRPNSVQPGAERE(R)PGAGAGAGENWEPRVLPYHP
lau Megabat      RGNTNAARRPNSVQPGAERE(R)PGAGAGAGENWEPRVLPYHP
afr Rock_hyrax   HGNTNAARRPNSVQPGAEQE(R)PGIGAGTGENWEPRVLPYHP
afr Tenrec       RGNTNAARRPNSVQAGAERE(R)PGPG---GENWEPRVLPYHP
xen Armadillo    RSNTNAARRPNSVQPGAERE(R)PGAG---GENWEPRVLPYRP

NO 49
GN CIAPIN1
IP IPI00387130
PE VSVENIKQLLQSA
MP 48
EX Wagner
CL humans
DE cytokine induced apoptosis inhibitor 1
SQ
pri Human        LVDKLQALTGNEGRVSVENI(K)QLLQSAHKESSFDIILSGLV
pri Chimpanzee   LVDKLQALTGNEGRVSVENI(N)QLLQSAHKESSFDIILSGLV
pri Gorilla      LVDKLQALTGNEGRVSVENI(N)QLLQSAHKESSFDIILSGLV
pri Orangutan    LVDKLQVLTGNEGRVSVENI(N)QLLQSAHKESSFDIILSGLV
pri Gibbon       LVDKLQVLTGNEGHVSVENI(N)QLLQSAHKESSFDIILSGLV
pri Rhesus       LVDKLQVLTGNEGHVSVENI(N)QLLQSAHKESSFDIILSGLV
pri Baboon       LVDKLQVLTGNEGHVSVENI(N)QLLQSAHKESSFDIILSGLV
pri Marmoset     LVDKLQVLTGNEGHVSVENI(N)QLLQSAHKESSFDIILSGLV
pri Tarsier      LVDKLQVLTGNEGRVSVENI(N)QLLQSAHKESSFDIILSGII
pri Bushbaby     LVDKLQVLTGSEGRVSVENI(D)QLLQXXXXXXXXXXXXXXXX
pri Mouse_lemur  LVEKLQVLTGSEGRVSVENI(N)QLLQSAHKESSFDIILSGVV
eua Treeshrew    LVGKLQVSTGNEGQVFVENI(N)QLLQXXXXXXXXXXXXXXXX
eua Mouse        LVARLQELTGSEGQVFMENV(T)QLLQSSHKESSFDVILSGVV
eua Rat          LVGRVQGLTGSEGQVFVENI(T)QLLQSAHKESSFDVILSGIV
eua Guinea_pig   LVDKLQGLTGSEDRVSVENI(D)QLLQSAHKESSFDIILSGVV
eua Squirrel     LVDKLQVLTGDKGQVSVENM(S)QLLQSAHKESSFDVILSGVV
eua Rabbit       LVDKLHVLTGSEGQVSVENV(N)QLLQSAHKESSFDVILSGVV
lau Dog          LVDKLQKLTGDEGQVSVENI(N)QLLKSAHKESSFDIILSGVI
lau Cat          LVEKLRTLTGDEGQVSVENI(S)QLLQSAHKESSFDVILSGVI
lau Horse        LVDKLQALTGDEGHVSMENI(N)QLLQSAHKESSFDIVLSGVI
lau Cow          LVDKLQALTGDEGRVSVENI(N)QLLQSAHKESSFDIVLSGII
lau Dolphin      LVDKLQALTGDEGRVSVENI(N)QLLQSAHKESSFDIILSGII
lau Alpaca       LVDKLQELTGIEGRVSVENI(N)QLVQSAHKESSFDIILSGVI
lau Megabat      LVDELQALTGSEGRVSVENI(N)QLLQSAHKESSFDIILSGVI
lau Microbat     LVEKLQALTGSEGRVSVENI(N)QLLQSAHKESSFDVILAGAI
lau Hedgehog     LVDKLQELTGDEGRVSVENI(N)QLLQSAHKESSFDIILSGVV
afr Elephant     LVDKLQALTGSEGHVSVENI(S)QLLQSAHKESSFDFILSGVV
xen Sloth        LVDKLQVLTGHEGRVSVENI(N)QLLQSAHKESSFDFILSGVI
mar Opossum      LVDKLQLLAGDESHVSVENI(N)QLLQSAHKESSFDVVLSGLV
mar Wallaby      LVDKLQKLSGDESHVSVENI(N)QLLQSAHKESSFDVVLSGLV
pro Platypus     LVDQLQATAGSESRVSVENI(D)QLLQSAHKESSFDVVLSGVV

NO 50
GN COL6A2
IP IPI00304840.4
PE APNQNLKEQGLRD
MP 197
EX Kim
CL haplorhines
DE collagen, type VI, alpha 2
SQ
pri Human        AERAREEGIRLFAVAPNQNL(K)EQGLRDIASTPHELYRNDYA
pri Chimpanzee   AERAREEGIRLFAVAPNQNL(K)EQGLRDIASTPHELYRNDYA
pri Gorilla      AERAREEGIRLFAVAPNQNL(K)EQGLRDIASTPHELYRNDYA
pri Orangutan    AERAREEGIRLFAVAPNQNL(K)EQGLRDIASTPHELYRNDYA
pri Gibbon       AERAREEGIRLFAVAPNQNL(K)EQGLRDIASTPHELYRNDYA
pri Rhesus       AERAREEGIRLFAVAPNRNL(K)EQGLRDIASTPHELYRNDYA
pri Baboon       AERAREEGIRLFAVAPNRNL(K)EQGLRDIASTPHELYRNDYA
pri Marmoset     AERAREEGIRLFAVAPNQNL(K)EQGLRDIASTPHELYRSDYA
pri Tarsier      AERAREEGIXLFAVAPNQNL(K)EQGLXXXXXXXXXXXXXXXX
eua Treeshrew    AERAREEGIRLFAVAPNVNL(N)EQGLRDIASTPHELYRSNYA
eua Mouse        AERAREEGIRLFAVAPNRNL(N)EQGLRDIANSPHELYRNNYA
eua Rat          AERAREEGIRLFAVAPNRNL(N)EQGLRDIANTPHELYRNNYA
eua Guinea_pig   AERARDEGIRLFAVSPNQNL(N)EQGLRDIASTPHELYRSNYA
eua Rabbit       AERAREEGIRLFAVAPNRNL(N)EQGLRDIASTPHELYRNNYA
eua Pika         AERAREEGIRLFAVSPNRNL(N)EQGLRDIASTPHELYRSNYA
lau Cat          AERAREEGIRLFAVAPRQNL(H)EQGLRDIASMPLELYRNNYA
lau Horse        AERAREEGIRLFAVAPNRNL(H)EQGLRDIASTPLELYRSNYA
lau Cow          AERAREEGIRLFAVPPNLKL(N)EQGLRDIANTPHELYRNNYA
lau Dolphin      AERAREEGIRLFAVPPYLKL(N)EQGLRDIANTPHELYRNNYA
lau Megabat      AERAREEGIRLFAVAPNRNL(N)EQGLRDIASTPHELYRSSYA
lau Microbat     AERAREEGIRLFAVAPNKNL(N)EQGLREIASTPQELYRSNYA
afr Elephant     AERAREEGIRLFAVAPNPNL(N)EQGLRDIASSPHELYRNNYA
afr Tenrec       AERAREAGIRLFDVVPNLHL(S)EQGLRDIANSPYELYRSNYA
mar Wallaby      AERARDMGIKLFSVALNQNK(N)EHGLREIANLPHELYRNNYL
pro Platypus     AERARDAGIKLFSVAVNENL(Y)ESGLREIANLPYELYRNNYT

NO 51
GN CORO7
IP IPI00027996.1
PE DGFCANKLRVAVP
MP 508
EX Kim
CL simians
DE coronin 7
SQ
pri Human        TNLKGLNLTTPGESDGFCAN(K)LRVAVPLLSSGGQVAVLELR
pri Chimpanzee   TNLKGLNLTTPGESDGFCAN(K)LRVAVPLLSSGGQVAVLELR
pri Gorilla      TNLKGLNLTTPGESDGFCAN(K)LRVAVPLLSSGGQVAVLELR
pri Orangutan    TNLKGLNLTTPGESDGFCAN(K)LRVAVPLLSSGGQVAVLELR
pri Gibbon       TNLKGLNLTTPGESDGFCAN(K)LRVAVPLLSSGGQVAVLEXX
pri Rhesus       TNLKGLNLTTPGESDGFCAN(K)LRVAVPLLSSGGQVAVLELR
pri Baboon       TNLKGLNLTTPGESDGFCAN(K)LRVAVPLLSSGGQVAVLELR
pri Marmoset     TNLKGLNLTTPGECDGFCAN(K)LRVAVPLLSSGGQVAVLELQ
pri Bushbaby     TNLKGLNLTTPGESNGFCAN(R)LRVAVPLLSSGGQVAVLELQ
pri Mouse_lemur  TNLKGLNLTTPGESDGFCAN(Q)LRVAVPLLSSGGQVAVLELR
eua Mouse        TNLKGLNLTTPGESDGFCAN(R)LRVAVPLLSSGGQVAVLELQ
eua Rat          TNLKGLNLTTPGESDGFCAN(R)LRVAVPLLSSGGQVAVLELQ
eua Kangaroo_rat TNLKGLNLTTPGESDGFCAN(R)LRVAVPLLSSGGQVAVLELR
eua Guinea_pig   TNLKGLNLTTPGESDGFCAN(Q)LHVAVPLLSSGGQVAVLELK
eua Squirrel     TNLKGLSLTTPGESDGFCAN(Q)LHVAVPLLSSGGQVAVLEXX
eua Rabbit       TNLKGLNLTTPGESDGFCAN(Q)LRVAVPLLSSGGQVAVLELR
eua Pika         TNLKGLNLTTPGESDGFCAN(R)LRVAVPLLSSGGQVAVLELR
lau Dog          TNLKGLNLTTPGESDGFCAN(R)LRVAVPLLSSGGQVAVLELQ
lau Cat          TNLKGLNLTTPGESDGFCAN(R)LRVAVPLLSSGGQVAVLELR
lau Horse        TNLKGLNLTTPGESDGFCAN(Q)LRVAVPLLSSGGQVAVLELR
lau Cow          TNLKGLNLTTPGESDGFCAN(Q)LRVAVPLLSSGGQVAVLELR
lau Dolphin      TNLKGLNLTTPGESDGFCAN(W)QRVAMPLLSSGGQVAVLELR
lau Megabat      TNLKGLSLTTPGESDGFCAN(R)LRVAVPLLSSGGQVAVLELQ
lau Hedgehog     TNLKGLNLTTPGESDGFCAN(Q)LRVAVPLLSSGGQVAVLELR
afr Rock_hyrax   TNLKGLNLTTPGESDGFCAN(Q)LRVAVPLLSSGGQVAVLELR
afr Tenrec       TNLKGLNLTTPGESDGFCAN(G)QRVAVPLLSSGGQVAVLELQ
mar Opossum      TNLKGLNLTTPGESDGFCAN(R)LHVALPLLSTGGQVAVLELR
mar Wallaby      TNLKGLNLTTPGESDGFCAN(R)LHVAVPLLSTGGQVAVLELR
pro Platypus     TNLKGLNLTTPGESDGFCAN(R)QRVAVPLLGTGGQIAVLELQ

NO 52
GN CORO7
IP IPI00027996.1
PE QEGPGPKGGRGAR
MP 680
EX Kim
CL simians
DE coronin 7
SQ
pri Human        RVRVYRPRSGPEPLQEGPGP(K)GGRGARIVWVCDGRCLLVSG
pri Gorilla      RVRVYRPRSGPEPLQEGPGP(K)GGRGARIVWVCDGRCLLVSG
pri Gibbon       RVRVYRPRIGPEPLQEGPGP(K)GGRGARIVWVCDGRCLLVSG
pri Rhesus       RVRIYRPRSGPEPLQEGPGP(K)GGRGARIVWVCDGRCLLVSG
pri Baboon       RVRIYRPRSGPEPLQEGPGP(K)GGRGARIVWVCDGRCLLVSG
pri Marmoset     RVRVYRPLSSPEPLQEGPGP(K)GGRGARIVWVCGGRCLLVSG
pri Mouse_lemur  RVRVYDPRSGSEPLQEGPGP(E)GGRGARIVWVCDGRCLLVSG
eua Treeshrew    HVRVYEPRGGPEPLQEGPGP(E)GARGARVVWVCGGRCLLVSG
eua Mouse        HVRVYEPRSSPLPLQEGPGP(E)GGRGARIVWVCDGGCLLVSG
eua Rat          RVRVYDPRSSPLPLQDGPGP(E)GGRGARIVWVCNGGCLLVSG
eua Kangaroo_rat RVRVYQPRSSPEPLQEGPGP(E)GARGARIVWVCDGHCLLVSG
eua Guinea_pig   YVRVYEPRSGPEPLQEGPGP(E)GGRGARIVWVCDGCCLLVSG
eua Squirrel     RVRVYEPRSGPEPLQEGPGP(E)GGRGARIVWVCDGRCLLVSG
eua Rabbit       RVRVYEPRNGPEPLQEGPGP(E)GGRGARVVWVCDGRCLLVSG
eua Pika         RVRVYEPLHGPEPLQEGPGP(E)GARGARVVWVCDGRCLLVSG
lau Dog          YLRVYEPRGGPEPLQEGPGP(E)GARGARVVWVCDGQCLLVSG
lau Cat          HLRVYEPRGGPEPLQEGPGP(E)GARGARIVWVCDGHCLLVSG
lau Cow          RLRIYEPRGSPEPLQEGPGP(E)GARGARVVWVCDGHYLLVSG
lau Dolphin      HLRIYEPRGSPEPLQEGPGP(E)GARGARIVWVCDGHCLLVSG
lau Megabat      HLRVYEPRSSPEPLQEGPGP(E)GARGARIVWVCDGYCLLVSG
lau Shrew        RVRVYAPRSSPEPLQEGPGP(E)GARGARIVWVCDGRCLLVSG
afr Elephant     HVRVYEPRSHPEPLQEGPGP(E)GARGARIVWVCDGHCLLVSG
afr Rock_hyrax   RVRVYEPRSGPEPLQEGPGP(E)GSRGARVVWVCDGHRLLVSG
afr Tenrec       HVRVYEPRSSPEPLEEGPGP(E)GVRGARVVWVCGGRCLLVSG
mar Opossum      RVRIYDPRSSPEPIQEGPGP(E)GGRGARVVWVCDGRCLLVSG
mar Wallaby      RVRIFDPRRSADPIQEGPGP(E)GGRGARVVWVCNGRCLLVSG
pro Platypus     KVRVYDPRNGPEPLQEGPGP(E)GGRGARVVWVCSGRCLLVSG

NO 53
GN CPPED1
IP IPI00305010.3
PE EQTEDLKRVLRAV
MP 109
EX Kim
CL catarrhines
DE calcineurin-like phosphoesterase domain containing 1
SQ
pri Human        GDLIHAMPGKPWRTEQTEDL(K)RVLRAVDRAIPLVLVSGNHD
pri Chimpanzee   GDLIHAMPGKPWRTEQTEDL(K)RVLRAVDRAIPLVLVSGNHD
pri Orangutan    GDLIHAMPGKPWRTEQTEDL(K)RVLRTVDRAIPLVLVSGNHD
pri Gibbon       GDLIHAMPGKPWRTEQTEDL(K)RVLRTVDRAIPLVLVSGNHD
pri Rhesus       GDLIHAMPGKPWRTEQTEDL(K)RVLRMVDRAIPLVLVSGNHD
pri Baboon       GDLIHATPGKPWRTEQTEDL(K)RVLRTVDRAIPLVLVSGNHD
pri Marmoset     GDLIHAMPGKPWRKEQTEDL(Q)RVLKTVDRAIPLVLVSGNHD
pri Tarsier      GDLIHAMPGMPWRKEQTEDL(Q)QVLRAVDKEIPLVIVNGNHD
pri Bushbaby     XXXXXXXXXMPWRKEQTKDL(Q)QILRKVDKEIPLVLVSGNHD
pri Mouse_lemur  GDLIHAMPGTPWRKEQTEDL(Q)RVLGAVDREIPLVLVSGNHD
eua Treeshrew    GDLIHAMPGTAWRKEQTEDL(Q)RVLREVDRDIPLVFVSGNHD
eua Mouse        GDLVHAMPGTPWRQEQTRDL(Q)RVLKAVDQDIPLVMVSGNHD
eua Rat          GDLVHAMPGTRWRKEQTRDL(Q)RVLKVVDQDIPLVLVSGNHD
eua Kangaroo_rat GDLIHAMPGTPWRKQQTEDL(Q)RVLGAVDREIPLVLVSGNHD
eua Guinea_pig   GDLIHAMPGTPWRKEQTEDL(Q)RVLKDLDREIPLVLVSGNHD
eua Squirrel     GDLIHAMPGMPFQREQTKDL(Q)RVLGAVDREIPLVFVSGNHD
eua Rabbit       GDLVHAMPGMPWRKEQTRDL(Q)RVLRQLDRDIPLVFVSGNHD
eua Pika         GDLVHAMPGTPWRKEQTRDL(Q)RVLGEVDRDIPLVFVSGNHD
lau Dog          GDLIHAMPGMPWRKEQTADL(Q)RVLTQVDSDIPLVLVSGNHD
lau Horse        GDLVHAMPGMPWRKEQTEDL(Q)RVLRDVDSEIPLVFVSGNHD
lau Cow          GDLVHAMPGRPWRKEQTEDL(Q)RVLRTVDSDIPLVLVSGNHD
lau Dolphin      GDLVHAMPGTPWRKEQTEDL(Q)RVLRTVDSEIPLVLVSGNHD
lau Alpaca       GDLIHAMPGTPWRKEQTEDL(Q)RVLRTVDSEIPLVFVSGNHD
lau Megabat      GDLIHAMPGMPWRKEQTDYL(Q)RVLRHVDSEIPLVLVSGNHD
lau Microbat     GDLIHAMPGMPWRKEQTKDL(Q)RVLRNVDSDIPLVLVSGNHD
lau Hedgehog     XXXXXXXXXXXWRKEQTEDL(Q)RVFAAVDSDIPLVLVSGNHD
afr Elephant     GDLIHAMPGTPWRQEQTEDL(Q)RVLKELDREIPLVFVSGNHD
afr Rock_hyrax   XXXXXXXXXXPWRKEQTEDL(Q)RVLMEVDREIPLVFVSGNHD
afr Tenrec       GDLIHAMPGTPWRKEQTEDL(Q)RVLEDVDSEIPLVFVSGNHD
xen Armadillo    GDLIHAMPGTPWRKEQTDDL(Q)RVLRDVDGDIPLVFVSGNHD
mar Opossum      GDLIHAMPGTQWRSNQIKDL(K)EILKAVDKEIPLVFVSGNHD
mar Wallaby      GDLIHAMPGTQWRENQTKDL(K)KVLKDIDKEVPLVFVSGNHD
pro Platypus     GDLIHGMPGTPWREEQIKDL(K)RVLKNIDRDIPLVFVSGNHD

NO 54
GN CREB3L4
IP IPI00152889.3
PE QGLQGWKSGGDRG
MP 51
EX Kim
CL simians
DE cAMP responsive element binding protein 3-like 4
SQ
pri Human        HCPPPEVPVTRLQEQGLQGW(K)SGGDRGCGLQESEPEDFLKL
pri Chimpanzee   HCPPPEVPVTRLQEQGLQGW(K)SGGDRGCGLQESEPEDFLKL
pri Gorilla      HCPPPEVPVTRLQEQGLQGW(K)SGGDGGCGLQESEPEDFLKL
pri Orangutan    HCPPPEVPVIRLQEQGLQGW(K)SGGDRGCGLQESEPEDFLKL
pri Gibbon       HCSPPEVPVTKLQEQGLQGW(K)SGGDRGCGLQESEPEDFLKL
pri Rhesus       HCPPPEVPVTRLQEQGLQGW(K)SGGDHGCGLQESEPEDFLKL
pri Baboon       HCPPPEVPVTRLQEQGLQGW(K)SGGDHGCGLQESEPEDFLKL
pri Marmoset     HCPAPEVPVTRLQEQGLQGW(K)SSGDHGCGLPESEPEDFLKL
pri Bushbaby     HCPPPEIPGTRLQEPGLQGL(E)SSGGRGYGLQESEPEDFLKF
pri Mouse_lemur  HCPPPEIPVTRLQEQGLQGL(E)SSGGHGCGLQESEPEDFLKL
eua Treeshrew    HCPP-EVPLARLQEQGLQGW(E)CSGGRGCGLQESEPEDLLNL
eua Kangaroo_rat NCSPPQVPVIKLQEQGLQGW(E)ASGGQVCGLQENESEDFLKL
eua Squirrel     NCSPPEVPVTRLQEQALHGW(D)SSGGQGCGLQESEPEDFLKL
eua Rabbit       QCPPPEAPGTRLQEQGPQGW(E)SGGDHGCGLQENEPEDCLRI
eua Pika         HCPP-EAPGTRLQEQGLPGW(E)STGGHGRGLQENEPEDCLKI
lau Horse        HCPPPEVPVTRLQEQGKQGW(E)SSGGLGCGLQESEPEDFLKL
lau Cow          QGLPSEVPVTRLQEQRLQGW(E)CSVGHGYGLQESEPEDFLKL
lau Dolphin      HGPPSEVPVTRLQEQGLQGW(E)SSGGHGCGLQESEPEDFLKL
lau Alpaca       LLP--EGPVIRLQEQGLQGW(E)SSGGHGCGLRESESEDFLKL
lau Megabat      HCPPPEAPVTRMQEQGLQGW(E)STGGRGRGLQESEPEDLPKL
lau Hedgehog     HCPVLEAPATRLQEQGLQAW(E)SSGDHGFGIQESESEDFLKL
afr Elephant     PCPPPEVSVTRQQEQGLQGW(T)SSGGHGCGLQESEPEDFLKL
afr Rock_hyrax   HCPP-EISVTRQQEHGMQSW(T)SSGDHGCGLQENEPEDFLKL
afr Tenrec       HCLPPELPGTRLPEQGLQGW(E)ATGGLGCGLQESEPEDSLKL
xen Armadillo    HCPPPAVPGTRLQEQGLQGW(E)SSGDHVCGLQESEPEDFLKL
mar Opossum      HSSFGEPERTGLQDPGLGGW(K)PSGSSSCGLQESEPDDFLNL

NO 55
GN CTSA
IP IPI00640525
PE RRPWLVKYGDSGE
MP 447
EX Wagner
CL simians
DE cathepsin A
SQ
pri Human        EWFVDSLNQKMEVQRRPWLV(K)YGDSGEQIAGFVKEFSHIAF
pri Chimpanzee   EWFVDSLNQKMEVQRRPWLV(K)YGDSGEQIAGFVKEFSHIAF
pri Gorilla      EWFVDSLNQKMEVQRRPWLV(K)YGDSGEQIAGFVKEFSHIAF
pri Orangutan    EWFVDSLNQKMEVQRRPWLV(K)YGDSGEQIAGFVKEFSHIAF
pri Gibbon       EWFVDSLNQKMEVQRRPWLV(K)YGDSGEQIAGFVKEFSHITF
pri Rhesus       EWFVDSLNQKMEVQRRPWLV(K)YGDSGEQIAGFVKEFSHIAF
pri Baboon       EWFVDSLNQKMEVQRRPWLV(K)YGDSGEQIAGFVKEFSHIAF
pri Marmoset     EWFVDSLNQKMEVQRRPWLV(K)YGDSGEQIAGFVKEFSHIAF
pri Bushbaby     EWFVDSLNQKMEVQRRPWLV(N)YGESGEQIAGFVKEFSHITF
pri Mouse_lemur  EWFVDSLNQKMEVQRRPWLV(N)YGESGEQIAGFVKEFSHIAF
eua Treeshrew    EWFVDSLNQKMEVQRRPWLV(D)YGESGEQIAGYVKDFSHITF
eua Mouse        EWFVDSLNQKMEVQRRPWLV(D)YGESGEQVAGFVKECSHITF
eua Rat          EWFVDSLNQKMEVQRRPWLV(D)YGESGEQVAGFVKECSHITF
eua Kangaroo_rat EWFVDSLNQKMEVQRRPWLV(D)YGESGEQIAGFVKEFSHITF
eua Guinea_pig   EWFVDSLNQKMEVQRRPWLV(D)YGDSGEQVAGFVKEFLNIDF
eua Rabbit       EWFVDSLNQKMEVQRRPWLV(D)YGDSGEQIAGFVKEFSHIAF
eua Pika         EWFVDSLNQKMEVQRRPWLV(D)YGESGEQIAGFVKEFSHIAF
lau Dog          EWFVDSLNQKMEVQRRPWLV(D)YGDSGEQIAGFVKEFSHIAF
lau Cat          EWFVDSLNQKMEVQRRPWLV(D)YGDSGEQIAGFVKEFSHIAF
lau Horse        EWFVDSLNQKMEVQRRPWLV(N)YGDSGEQIAGFVKEFSHIAF
lau Cow          EWFVDSLNQKMEVQRRPWLV(D)YGESGEQIAGFVKEFSHIAF
lau Dolphin      EWFVDSLNQKMEVQRRPWLV(D)YGDSGEQIAGFVKEFSHIAF
lau Megabat      EWFVDSLNQKMEVQRRPWLV(D)YGDSGEQIAGFVKEFSHIAF
lau Microbat     EWFVDSLNQKMEVQRRPWLV(K)YGDSGEQIAGFVKEFSNIAF
lau Hedgehog     EWFVDSLNQKMEVQRRPWLV(D)YGDSGEQIAGFVKEFSHISF
afr Elephant     EWFVDSLNQKMEVQRRPWLV(G)YGESGEQIAGFVKEFSHIAF
afr Rock_hyrax   EWFVDSLNQKIEVQRRPWLV(G)YGESGEQIAGFVKEFAHITF
afr Tenrec       EWFVDSLNLKMEVQRRPWLV(K)LGESGEQIAGFVKESTNIAF

NO 56
GN CTSD
IP IPI00011229
PE LGGKGYKLSPEDY
MP 348
EX Wagner
CL catarrhines
DE cathepsin D
SQ
pri Human        PCEKVSTLPAITLKLGGKGY(K)LSPEDYTLKVSQAGKTLCLS
pri Chimpanzee   PCEKVSTLPAITLKLGGKGY(K)LSPEDYTLKVSQAGKTLCLT
pri Gorilla      PCEKVSTLPAITLKLGGKGY(K)LSPEDYTLKVSQAGKTLCLS
pri Orangutan    PCEKVSTLPAITLKLGGKGY(K)LSPEDYTLKVSQAGKTLCLS
pri Gibbon       PCEKVSTLPAITLKLGGKGY(K)LSPEDYTLKVSQAGKTLCLS
pri Rhesus       PCEKVSTLPTITLKLGGKGY(K)LSPEDYTLKVSQAGKTLCLS
pri Baboon       PCEKVSTLPTITLKLGGKGY(K)LSPEDYTLKVSQAGKTLCLS
pri Marmoset     PCEKVSTLPVIMLKLGGKDY(E)LSPQDYTLKVSQAGKTICLS
pri Mouse_lemur  PCDKVSSLPSVTLKLGGKDY(V)LSGEDYTLXXXXXXXXXXXX
eua Mouse        PCEKVSSLPTVYLKLGGKNY(E)LHPDKYILKVSQGGKTICLS
eua Rat          PCEKVSSLPIITFKLGGQNY(E)LHPEKYILKVSQAGKTICLS
eua Kangaroo_rat PCEKVSSLPSVTLNLGGKDY(T)LTADDYILKVSQAGKSICLS
eua Guinea_pig   PCEKVSSLPSVTLKLGGTDY(T)LASEDYVLKVSQAGKTICLS
eua Rabbit       PCEKVSSLPPVTLKLGGRDY(T)LSSEDYTLKVSQGGKTICLS
eua Pika         PCEKVSGLPAVTVKLGGRDY(T)LSAEDYTLTVSQGGKTICLS
lau Dog          PCEKVSTLPDVTLKLGGKLY(K)LSSEDYTLKVSQGGKTICLS
lau Cat          PCEKVSTLPEVTVKLGGKGY(K)LSSKDYTLKVSQGGRTICLS
lau Cow          PCEKVSSLPQVTVKLGGKDY(A)LSPEDYALKVSQAGTTVCLS
lau Dolphin      PCEKVSSLPKVTVKLGAKDY(T)LSPEDYTLKVSQAGRTMCLS
lau Megabat      PCEKVSSLPEVTLKLGGKGY(K)LGAEDYTLKVSQGGKTICLS
lau Microbat     PCEKVSSLPEVTLKLGGKDY(K)LRAEDYTLKVSQGGKTICLS
afr Elephant     PCEKVSSLPPVSLQLGGRSY(T)LSSEDYVLKVSQAGRSVCLS
afr Rock_hyrax   PCEKISGLPPVSLQLGGKSY(T)LSPEDYVLKVSQAGKTICLS
afr Tenrec       PCEKISSLPDIVLSLGNKPY(T)LSPEAYVLKVSQAGKTICLS

NO 57
GN CUL7
IP IPI00001690.2
PE WRAVVEKQVNNFL
MP 1153
EX Kim
CL simians
DE cullin 7
SQ
pri Human        RGLPSSIMRNLTRCWRAVVE(K)QVNNFLTSSWRDDDFVPRYC
pri Chimpanzee   RGLPSSIMRNLTRCWRAVVE(K)QVNNFLTSSWRDDDFVPRYC
pri Gorilla      RGLPSSIMRNLTRCWRAVVE(K)QVNNFLTSSWRDDDFVPRYC
pri Orangutan    RGLPSSIMRNLTRCWRAVVE(K)QVNNFLTSSWRDDDFVXXXX
pri Gibbon       RGLPSSIMRNLTRCWRAVVE(K)QVNNFLTSSWRDDDFVPRYC
pri Rhesus       QGLPSSIMRNLTRCWQAVVE(K)QVNNFLTSSWRDDDFVPRYC
pri Baboon       RGLPSSIMRNLTRCWQAVVE(K)QVNNFLTSSWRDDDFVPRYC
pri Marmoset     RGFPSSIMRNLTRCWQAVVE(K)QVDNFLTSSWRDDDFVPRYC
pri Tarsier      RGLPSSIMRNLTRCWQAVVE(E)QVNNFLTSSWQDDDFVPRYC
pri Bushbaby     RGLPSSIMRNLTRCWRSVVE(E)QVNNFLTSSWRDDDFVPRYC
eua Treeshrew    RGLPSSIMRNLTRCWRAVVE(E)QVDGFLTSHWQDDDFVPRYC
eua Mouse        RGLPSSIMKNLTRCWRSVVE(E)QMNKFLTASWKDDDFVPRYC
eua Rat          RGLPSSIMRNLTRCWRSVVE(E)QVNKFLTSSWKDDDFVPRYC
eua Kangaroo_rat RGLPSSIMRNLTRCWQAVVE(E)KVNNFLTSHWRDDDFVPRYC
eua Guinea_pig   RGLAGSILRNLTRCWRAVVE(Q)QVNAFLTSHWRDNDFVQHYC
eua Rabbit       RGLPSSIMRNLTRCWRAVVE(E)QVHSFLTSHWRDDDFVPRYC
eua Pika         RGLPSSIMRNLTRCWQAVVE(D)QVHTFLVSHWRDENFVPRYC
lau Dog          RGLPSSIVRNLTRCWQAVVE(E)QVNNFLTSHWRDDDFVPRYC
lau Cat          RGLPSSIVRNLTRCWRAVVE(E)QVNNFLTSRWRDGDFVPRYC
lau Horse        RGLPSSIMRNLTRCWGAVVE(E)QVNNFLTSHWRDDDFVPRYC
lau Cow          RGLPSSIMRNLTRCWRAVVE(E)QVHSFLTSRWRDDDFVPRYC
lau Dolphin      RGLPGSIMRNLTRCWRAVVE(E)QVSSFLTSHWRDDDFVPRYC
lau Megabat      RGLPSSIMRNLTRCWQSAVE(E)QVNSFLTSHWRDDDFVPRYC
lau Hedgehog     RGLPSSIMRNLTRCWRAVVE(E)QVNNFLTSHWRDDDFVPRYC
afr Elephant     RGLPSSIMRNLTRCWRAVVE(E)QVNKFLTSYWQDDDFVPRYC
afr Rock_hyrax   RGLPSSIMRNLTRCWRVVVE(E)QVNKFMTSYWQDDDFVPRYC
afr Tenrec       RGLPSSIMRNLTRCWRAVVE(E)QVNKFLTSYWQDDDFVPRYC
xen Armadillo    RGLPSSITRNLTRCWQAAVE(E)QVNNFLTSSWQDDDLVPRYC
mar Opossum      LGPPGGSLRGITQCWRGVVQ(E)QVSRFLTAAWQAPDLVPRYC
mar Wallaby      RGTPGGSLRGITQCWRGVVQ(E)QVSRFLTAAWQEPDLVPRYC

NO 58
GN CUL7
IP IPI00001690.2
PE QSLSTSKELQRQF
MP 1312
EX Kim
CL simians
DE cullin 7
SQ
pri Human        IGPCFPNRLPQQMLQSLSTS(K)ELQRQFHVYQLQQLDQELLK
pri Chimpanzee   IGPCFPNRLPQQMLQSLSTS(K)ELQRQFHVYQLQQLDQELLK
pri Gorilla      IGPCFPNRLPQQMLQSLSTS(K)ELQRQFHVYQLQQLDQELLK
pri Orangutan    IGPCFPNRLPQQMLQSLSTS(K)ELQRQFHVYQLQQLDQELLK
pri Gibbon       IGPCFPNRLPQQMLQSLSTS(K)ELQRQFHVYQLQQLDQELLK
pri Rhesus       IGPCFPNRLPQQMLQSLSTS(K)ELQRQFHVYQLQRLDQELLK
pri Baboon       IGPCFPNRLPQQMLQSLSTS(K)ELQRQFHVYQLQRLDQELLK
pri Marmoset     IGPCFPNRLPQQMLQNLSTS(K)ELQRQFHVYQLQRLDQELLK
pri Tarsier      IGPCFPNRLPQQMLRSLSAS(E)ELQRQFHVYQLQRLDQELXX
pri Mouse_lemur  IGPCFPNRLPQQMLRSLSTS(E)ELQRQFHVYQLQRLDQELLK
eua Treeshrew    IGPCFPNRLPQQMLQSLSTS(E)ELQRQFHVYQLQRLDQELLK
eua Mouse        IGPCFPSRLPQQMLQSLNVS(E)ELQRQFHVYQLQQLDQELLK
eua Rat          IGPCFPGRLPQQMLQTLNIS(E)ELQRRFHVYQLQQLDQELLK
eua Kangaroo_rat IGLCFPNRLPQQMLQSLSTS(E)ELQRQFDVYQLQQVDQELLK
eua Guinea_pig   IGLCFPNRLPQLMLRSLRTS(E)ELQRQFHVYQLQQLDEELLK
eua Rabbit       IGSCFPNRLPQQMLRSLSTS(E)ELQRQFHVYQLQRLDQELLR
eua Pika         IGLCFPNRLPQQMLRSLSTS(E)ELQRQFHVYQLQRLDQELLK
lau Dog          IGPCFPNRLPQQMLQSLSTS(E)ELQRQFHVYQLQRLDQELLK
lau Cat          IGPCFPNRLPQQMLRSLSMS(E)ELQRQFHVYQLQRLDQELLK
lau Horse        IGLCFPNRLPQQMLRSLSTS(E)ELQRQFHVYQLQRLDQELLK
lau Cow          IGPCFPTRLPQQMLRSLSTS(E)ELQRQFHVYQLQRLDQELLK
lau Dolphin      IGPCFPNRLPQQMLRSLSTS(E)ELQRQFHVYQLQRLDQELLK
lau Megabat      IGPCFPNRLPQQMLRSLSTS(E)ELQRQFHVYQLQRLDQELLK
lau Shrew        IGLCFPNRLPQQMLRSLSAS(E)ELQRQFQVYQLQRLDQELLK
afr Elephant     IGPCFPNRLPQQMLQTLNTS(E)ELQHQFHVYQLQRLDQALLK
afr Rock_hyrax   IGPCFPNRLPQQMLQTLSTS(E)ELQHQFHVYQLQQLDQALLK
afr Tenrec       VGPCFPNRLPQQMLQSLRTS(E)ELQHQFHVYQLQRLDQELLK
xen Armadillo    IGPCFPNRLPQQMLQSLNTS(E)ELQRRFHIYQLQRLDQELLK
mar Opossum      IGLCFPNRLPQQMLSSLSTS(Q)ELQHQFHLFQLEQIDQQLLE
mar Wallaby      IGLCFPNRLPQQMLSSLSTS(E)ELQHQYHLFQLEQIDQQLLE
pro Platypus     IGLCFPNRLPQQMLSGLRAA(A)ELQRHFRLFRLQLFDRRLLE

NO 59
GN D2HGDH
IP IPI00166642
PE PFSTVSKQDLAAF
MP 64
EX Wagner
CL apes
DE D-2-hydroxyglutarate dehydrogenase
SQ
pri Human        EVPLTRERYPVRRLPFSTVS(K)QDLAAFERIVPGGVVTDPEA
pri Chimpanzee   EVPLTRERYPVRRLPFSTVS(K)QDLAAFERIVPGGVITDPEA
pri Gorilla      EVPLTRERYPVRRLPFSTVS(K)QDLAAFERIVPGGVVTDPEA
pri Orangutan    EVPLTRERYPVRRLPFSTVS(K)QDLAAFERIVPGGVVTDPEA
pri Gibbon       EVPLTRERYPVRRLPFSTVS(K)QDLAAFERIVPGGVVTDPEA
pri Rhesus       EVPLTRERYPVRRLPFSTVS(E)QDLAAFERIVPGGVVTDPEA
pri Baboon       EVPLTRERYPVRRLPFSTVS(E)QDLAAFERIVPGGVVTDPEA
pri Marmoset     EVPLTQERYPVRRLPFSTVS(E)QDLAAFERIVPGGVVTDPEA
eua Mouse        EVTLTPERYPVQRLPFSTVS(E)EDLAAFECIIPGRVITDPEQ
eua Rat          EVMLTPERYPVQRLPFSTVS(E)EDLAAFECIIPGRVITDPEQ
eua Guinea_pig   SVMLTSERYSVQRLPFSMVS(E)EDLASFECIIPGRVITDPEE
eua Rabbit       EVTLTPQRYAVQRLPFSAVS(E)QDLAAFERIVPGRVVTDPDE
lau Dog          EVALTQERYPVKRLPFSTPS(E)ADLAAFERIIPGRVVTDTEV
lau Cat          EVMLTRERYPVRRLPFSVVS(G)EDLAAFERIVPGRVITDPEV
lau Horse        EVMLTRERYPVQRLPFSVVS(E)EDLAAFERITPGRVVTDPEE
lau Cow          EVTLTPERYPVQRLPFSVVS(E)DDLAALERVVPGRVITDPEE
lau Dolphin      EVVLTRERYSVRRLPFSVVS(E)DDLAALERIVPGRVITDPEE
lau Megabat      EVVLTHERYPVQRLPFSVVS(E)EDRAAFERMVPGRVIMDPEE
afr Elephant     EVMLTQERYPVRRLPFAVVS(E)EDLATFEGILPGRVVTSPEE
afr Tenrec       GVRLTQERYPVQRLPFSVVS(E)QDLAAFERILPGRVVTSPEE
xen Armadillo    EVALTSERYPVRRLPFSQVS(E)EDMAAFERIVPGGVITDPQA
mar Opossum      EVTRTRDRYPVRRLPFAEVS(E)EDLAFFERVLPGRVCTDARE
mar Wallaby      EVMLTCDRYPVRRLPFAEVS(E)EDLAFFERIIPGRVITDPHE
pro Platypus     EVMLTSDRYAVKRLPFSHVS(N)EDLAFFENIIPGRVVTDPAE

NO 60
GN DCAF6
IP IPI00164246
PE SDKFTAKPLDSNS
MP 652
EX Wagner
CL haplorhines
DE DDB1 and CUL4 associated factor 6
SQ
pri Human        SAENPVENHINITQSDKFTA(K)PLDSNSGERNDLNLDRSCGV
pri Chimpanzee   SAENPVENHINITQSDKFTA(K)PLDSNSGERNDLNLDRSCGV
pri Gorilla      SAENPVENHINITQSDKFTA(K)PLDSNSGEKNDLNLDRSCGV
pri Orangutan    SAENPVENHINITQSDKFTA(K)PLDSNSGERNDLNLDSSCGV
pri Gibbon       SAENPVENHINITQSDKFTA(K)PSDSNSGERNDLNLDSPCGV
pri Rhesus       SAENPVQNHINITQSDKFTA(K)PSDSNSGERNDLNLDSSCGV
pri Baboon       SAENPVQNHINITQSDKFTA(K)PSDSNSGERNDLNLDSSCGV
pri Marmoset     SAENPVQNHINIAQSDKFTA(K)PLDSNSGERNELNLDIPCGV
pri Tarsier      SAENPIQNHIDIPQSDKFTT(K)PLDSNSGERNDFNLDSPCGV
pri Mouse_lemur  SAESPVQNHIDIAQSDEFTT(E)PXXXXXXXXXXXXXXXXXXX
eua Treeshrew    SAESPVQNPVDTAQSDLLTA(E)PLDSRSEEGHDLSLGSPCGV
eua Mouse        LPENLTQNQIDTAQLDNFPA(E)PLDSNSGEKNNPSQDSPCGL
eua Rat          FSENLTQNQIDTAQFDNFPT(E)PLDSNSGERNNPSLDSPCGV
eua Kangaroo_rat SAENLVESHADIAQLGNLAV(E)PLNSNSGEKNDLSLDSPSVV
eua Rabbit       SAENLDENSTDKAQSDTFTA(E)PLESNSGERNNLNLDSPCEV
eua Pika         YAETLGQNPTAKAQSNTFTS(E)P-SSNSGDKSDLNLGSPCEG
lau Dog          SAENTIEKRIDIAQSDKLTS(E)PLDSSSGERNDFNLDSSCEV
lau Cat          XXXXXXXXXXXXXQSDKLTS(E)PLDSCSGERNDLNLDSSCGV
lau Horse        SAENIVQDRVDIAQSDKFTS(E)PLDSSSGERNDFHLDSPCGV
lau Cow          SAESTVQNHIDTAQSDKFTS(E)SLDSGSGERNDFNLDSPCGV
lau Dolphin      SAESTVQNSIDIAQSDKFTF(E)PLDPGSGERNDFNLDSPGGV
lau Alpaca       SAENTAQNHVDRAQSDKFTS(E)PLDSGSGERNDLNLDSPCGV
lau Megabat      SSEDIAQNRVDTAQSDKCTS(E)PSDSSSGERNDFNLDSPCEV
lau Hedgehog     AAEISVQDPIDIAQSDQFTS(E)LLGSSSGERNDFNLDSPCGV
afr Elephant     SAENTVENQIVVAQSDKLTS(E)PLDSSSGERNDLCLDSPCGV
afr Rock_hyrax   SAEDTTENRIDVARSDSVTS(E)PLDSSSGERNDLDHGSPCEV
afr Tenrec       SAEDAVENPVDRAQSDKVTP(A)PLDSSSGERNDPSLESLCAV
xen Sloth        SIENTGQHK---AQSDKFTS(E)PLDSSSGERNDINLDISCGA
mar Opossum      YAEETGQNPTDPAQLDTVFS(E)SLECSSEEKNDPNHDNSGNV
mar Wallaby      YSEETVQNPTDTAQSD-IVS(E)PLGCSSEEKNDPNLDNSCKV

NO 61
GN DKC1
IP IPI00221394.8
PE PESKVAKLDTSQW
MP 46
EX Kim
CL simians
DE dyskeratosis congenita 1, dyskerin
SQ
pri Human        DVAEIQHAEEFLIKPESKVA(K)LDTSQWPLLLKNFDKLNVRT
pri Chimpanzee   DVAEIQHAEEFLIKPESKVA(K)LDTSQWPLLLKNFDKLNVRT
pri Gorilla      DVAEIQHAEEFLIKPESKVA(K)LDTSQWPLLLKNFDKLNVRT
pri Orangutan    DVAEIQHAEEFLIKPESKVA(K)LDTSQWPLLLKNFDKLNVRT
pri Gibbon       DVAEIQHAEEFLIKPESKVA(K)LDTSQWPLLLKNFDKLNVRT
pri Rhesus       DVAEIQHAEEFLIKPESKVA(K)LDTSQWPLLLKNFDKLNVRT
pri Baboon       DVAEIQHAEEFLIKPESKVA(K)LDTSQWPLLLKNFDKLNVRT
pri Marmoset     DVAEIQHVEEFLIKPESKVA(K)LDTSQWPLLLKNFDKLNVRT
pri Tarsier      DVAEIQHAEEFRIKPESKVA(Q)LDTYQWPLLLLNFDRLNVRT
pri Bushbaby     DIAEIQHAEIFLIKPESKVA(Q)LDTSQWPLLLKNFDKLNVRT
pri Mouse_lemur  DVAEIQHAEEFLIKPESKVA(Q)LDTSQWPLLLKNFDKLNVRT
eua Mouse        DVAEIQHAEEFLIKPESKVA(Q)LDTSQWPLLLKNFDKLNVRT
eua Kangaroo_rat DVAEIQYAEEFLIKPESKVA(Q)LDTSQWPLLLKNFDKLNVRT
eua Guinea_pig   DVAEIQHAEEFLIKPESKVA(Q)LDTSQWPLLLKNFDKLNVRT
eua Squirrel     DVAEIQHAEEFLIKPESKVA(Q)LDTSQWPLLLKNFDKLNVRT
eua Rabbit       DIAEIQHAEEFLIKPESKVA(Q)LDTSQWPLLLKNFDKLNVRT
lau Dog          DVAEIQHAEEFLIKPESKVA(Q)LDTSQWPLLLKNFDKLNVRT
lau Horse        DVAEIQHAEEFLIKPESKVA(Q)LDTSQWPLLLKNFDKLNVRT
lau Cow          AVAEIQHAEEFLIKPESRVA(Q)LDTSQWPLLLKNFDKLNVRT
lau Dolphin      DAEEIQHAEEFLIKPESKVA(Q)LDTSQWPLLLKNFDKLNVRT
lau Alpaca       DVAEIQHAEEFLIKPESKVA(Q)LDTSQWPLLLKNFDKLNVRT
lau Megabat      DVAEIQHVEEFLIKPESKVA(Q)LDTSQWPLLLKNFDKLNVRT
lau Microbat     DVAEIQHIGEFLIKPESKVA(Q)LDTSQWPLLLKNFDKLNIRT
lau Shrew        DVAEIQLAEDFLIKPESKVA(Q)LDTSHWPLLLKNFDKLNIRT
afr Elephant     DVAEIQHAEEFLIKPESKVA(Q)LDTSQWPLLLKNFDKLNVRT
afr Tenrec       DVAEIQHAEEFLIKPESKVA(L)LDTSQWPLLLKNFDKLNVRT
xen Armadillo    DIAEIQHIEEFLIKPESKVA(Q)LDTSQWPLLLKNFDKLNVRT
xen Sloth        DVAEIQHIEDFPIKPESKVA(Q)LGTSQWPLLLKNFDKLNVRT
mar Opossum      AVAELQHEGEFFIKPESKVV(Q)LDTSQWPLLLKNFDKLNVRT
mar Wallaby      DVADLQHEGEFFIKPESKVI(Q)LDTSQWPLLLKNFDKLNVRT
pro Platypus     DVAEIQHMGEFFIKPESKVA(Q)LDTSQWPLLLKNFDKLNVRT

NO 62
GN DKFZp761E198
IP IPI00386243.3
PE GPLPVLKLQPEAL
MP 624
EX Kim, Wagner
CL catarrhines
DE adaptor protein 5
SQ
pri Human        PALVRLSLGSHRVKGPLPVL(K)LQPEALE-PIYSLELRFRVE
pri Chimpanzee   PALVRLSVGSHRVKGPLPVL(K)LQPEALE-PIYSLELRFRVE
pri Gorilla      PALVRLSMGSHRVKGPLPVL(K)LQPEALE-PIYSLELRFRVE
pri Orangutan    PALVRLSVGSHRVKGPLPVL(K)LQPEALE-PIYSLELRFRVE
pri Gibbon       PALIQLSMGSHRVKGPLPVL(K)LQPEALE-PIYSVELRFRVE
pri Rhesus       PALVRLSVGSHGVKGPLPVL(K)LQPEALE-PIYSLELRFRVE
pri Baboon       PALVRLSVGSHGVKGPLPVL(K)LQPEALE-PIYSLELRFRVE
pri Marmoset     PALIRLSMGSHEVKGPLPVL(Q)LLPEALE-PIYSLELRFRVE
eua Treeshrew    PAPIRLSLGPSRAEGPLPVL(Q)LQVEVLQ-PIYSLELRFQVE
eua Mouse        SAPIQLSVGPQQAKGPLPVL(C)LQVQALDAPVYSLELRFRVE
eua Rat          SAPIQLSVGPQKAKGPLPVL(H)LQVQALDTPVYSLELRFRVE
eua Kangaroo_rat PAPIQLSLGPQRAKGPFPVL(Q)LQVETLE-PVYSLELRFHVE
eua Guinea_pig   LAPIQLRVGPRIAKGPLPVL(Q)LHIEALE-PIYSLELRFRVE
eua Rabbit       AAPMRLSVGPRVATGPCPVL(Q)LQVEALE-PAYSLELRFRVE
eua Pika         AAPVRLSVGPRVTQGQLPVL(R)LQVEALQ-PVYSLELRFRVE
lau Dog          PAPIRLSVGPRRAEGPVPVL(Q)LQVEVLE-PVYSLELRFRVE
lau Cat          PAPIRLSVGPHRAAGPGPVL(Q)LHVEVLE-PAYSLELRFRVE
lau Horse        PAPIQLSVGPRRAEGPIPVL(Q)LQVEVLE-PVYSLELRFRVE
lau Cow          PAPIRLSVGPRRAEGAVPVL(R)LQVEVLE-PVYSLELRFRVE
lau Dolphin      PAPIRLSMGPHRAKGPVPVL(Q)LQVEVLE-QVYSLELRFRVE
lau Microbat     PAPIRLSVGPRRAEGPVPVL(Q)LQVEVLE-PVYSLELRFRVE
lau Hedgehog     PAPLRLSVGPRRAGGPTPAL(Q)LLVEALE-PAFSLELRFSAP
afr Elephant     PASLRLSVGPRRVEGPSPVL(E)LQVEALQ-PLYSLELRFRVE
afr Tenrec       PAALRLSVGPRRLEGPSPVL(Q)LLLEPLQ-PLYSLELRFRVE
mar Opossum      RAPLRLSRVPQQAKEPPVVL(G)L-VEASE-VLYSLELRFRVL
mar Wallaby      QAPLQLSV-FQQAKGPPVVL(G)LQ-EASE-ILYSLELRFRVL

NO 63
GN DLD
IP IPI00015911
PE GRRPFTKNLGLEE
MP 320
EX Wagner
CL simians
DE dihydrolipoamide dehydrogenase
SQ
pri Human        KAEVITCDVLLVCIGRRPFT(K)NLGLEELGIELDPRGRIPVN
pri Chimpanzee   KAEVITCDVLLVCIGRRPFT(K)NLGLEELGIELDPRGRIPVN
pri Gorilla      KAEVITCDVLLVCIGRRPFT(K)NLGLEELGIELDPRGRIPVN
pri Orangutan    KAEVITCDVLLVCIGRRPFT(K)NLGLEELGIELDPRGRIPVN
pri Gibbon       KAEVITCDVLLVCIGRRPFT(K)NLGLEELGIELDPRGRIPVN
pri Rhesus       KAEVITCDVLLVCIGRRPFT(K)NLGLEELGIELDPRGRIPVN
pri Baboon       KAEVITCDVLLVCIGRRPFT(K)NLGLEELGIELDPRGRIPVN
pri Marmoset     KAEVITCDVLLVCIGRRPFT(K)NLGLEELGIELDPRGRIPVN
pri Tarsier      KAEVITCEVLLVCIGRRPFT(Q)NLGLEELGIELDPRGRIPVN
eua Mouse        KAEVITCDVLLVCIGRRPFT(Q)NLGLEELGIELDPKGRIPVN
eua Rat          KAEVITCDVLLVCIGRRPFT(Q)NLGLEELGIELDPKGRIPVN
eua Guinea_pig   KAEVITCDVLLVCIGRRPFT(Q)NLGLEELGIELDPRGRIPVN
eua Squirrel     KAEVITCDVLLVCIGRRPFT(Q)NLGLEDLGIELDPRGRIPVN
eua Rabbit       KAEVITCDVLLVCIGRRPFT(Q)NLGLEELGIELDPRGRIPVN
eua Pika         KAEVITCDVLLVCIGRRPYT(Q)NLGLEALGIELDPRGRIPVN
lau Dog          KAEVITCDVLLVCIGRRPFT(Q)NLGLEELGIELDPRGRIPVN
lau Cat          KAEVITCDVLLVCIGRRPFT(Q)NLGLEELGIELDPRGRIPVN
lau Horse        KAEVITCDVLLVCIGRRPFT(Q)NLGLEELGIELDPRGRIPIN
lau Cow          KAEVITCDVLLVCIGRRPFT(Q)NLGLEELGIELDTRGRIPVN
lau Dolphin      KAEVITCDVLLVCIGRRPFT(K)NLGLEELGIELDPRGRIPVN
lau Alpaca       KAEVITCDVLLVCIGRRPFT(Q)NLGLEELGIELDPRGRIPVN
lau Megabat      KAEVITCDVLLVCIGRRPFT(Q)NLGLEELGIELDPRGRIPVN
lau Microbat     KAEVITCDVLLVCIGRRPFT(Q)NLGLEELGIELDPRGRIPIN
lau Hedgehog     KNEVITCDVLLVCIGRRPFT(K)NLGLEDIGIELDPRGRIPVN
lau Shrew        KSEVITCDVLLVCIGRRPFT(Q)NLGLEALGIELDPRGRIPVN
afr Elephant     KAEVITCDVLLVCIGRRPFT(Q)NLGLEELGIDLDSRGRIPVN
afr Tenrec       KAEVITCDVLLVCIGRRPFT(Q)NLGLEELGIELDPRGRIPVN
xen Armadillo    KAEVLTCDVLLVCIGRRPFT(Q)NLGLEELGIELDPRGRIPVN
xen Sloth        KAEVLTCDVLLVCIGRRPFT(Q)NLGLEELGIELDSRGRIPVN
mar Opossum      KAEIITCDLLLVCIGRRPFT(K)NLGLEEFGIELDPKGRIPVN
mar Wallaby      KAEVLTCDVLLVCVGRRPFT(K)NLGLEELGIELDPKGRIPVN
pro Platypus     KGEVITCDVLLVCIGRRPFT(Q)NLGLEELGIELDARGRIPIN

NO 64
GN DNPEP
IP IPI00015856.5
PE EVANKVKVPLQDL
MP 417
EX Kim
CL catarrhines
DE aspartyl aminopeptidase
SQ
pri Human        QRYASNAVSEALIREVANKV(K)VPLQDLMVRNDTPCGTTIGP
pri Chimpanzee   QRYASNAVSEALIREVANKV(K)VPLQDLMVRNDTPCGTTIGP
pri Gorilla      QRYASNAVSEALIREVANKV(K)VPLQDLMVRNDTPCGTTIGP
pri Orangutan    QRYASNAVSEALIREVANKV(K)VPLQDLMVRNDTPCGTTIGP
pri Gibbon       QRYASNAVSEALIREVANKV(K)VPLQDLMVRNDTPCGTTIGP
pri Rhesus       QRYASNAVSEALIREVANKV(K)VPLQDLMVRNDSPCGTTIGP
pri Baboon       QRYASNAVSEALIREVANKV(K)VPLQDLMVRNDSPCGTTIGP
pri Marmoset     QRYASNAVSEALIREVANKV(N)VPLQDLMVRNDSPCGTTIGP
pri Bushbaby     QRYASNAVSEALIREVANNV(G)VPLQDLMVRNDSSCGTTIGP
pri Mouse_lemur  QRYASNAVSEALIREVASNV(G)VPLQDLMVRNDSPCGTTIGP
eua Mouse        QRYASNAVSESMIREVAGQV(G)VPLQDLMVRNDSPCGTTIGP
eua Rat          QRYASNAVSESLIREVAGQV(G)VPLQDLMVRNDSPCGTTIGP
eua Kangaroo_rat QRYASNAVSEALIREVANSV(G)VPLQDLMVRNDSPCGTTIGP
eua Guinea_pig   QRYASNAVSEALIRQVASNV(G)VPLQDLMVRNDSPCGSTIGP
eua Squirrel     QRYSSNAVSEALIREVASSV(G)VPLQDFMVRNDVSCGTTIGP
eua Rabbit       QRYASNAISEALIREVASHV(G)VPLQDLMVRNDSPCGTTIGP
eua Pika         QRYASNAISEALIREVASHV(G)VPLQDLMVRNDSPCGTTIGP
lau Dog          QRYASNAVSEALIREVANNV(G)VPLQDLMVRNDSPCGTTIGP
lau Cat          QRYASNAVSEALIREVASNV(G)VPLQDLMVRNDSPCGTTIGP
lau Horse        QRYASNAVSEALIREVASSV(G)VPLQDLMVRNDSPCGSTIGP
lau Cow          QRYASNAVSEALIREVASSV(G)VPLQDLMVRNDSPCGTTIGP
lau Dolphin      QRYASNAVSEALIREVASSV(G)VPLQDLMVRNDSPCGTTIGP
lau Alpaca       QRYASNAVSEALIREVAKNV(G)VPLQDLMVRNDSPCGTTIGP
lau Megabat      QRYASNAVSEALIREVASNV(G)VPLQDLMVRNDSPCGTTIGP
lau Microbat     QRYASNAVSEALIRRVANNV(G)VPLQDLMVRNDSPCGTTIGP
lau Hedgehog     QRYASNAVTEAIIREVAKNV(G)VPLQDLMVRNDSPCGTTIGP
afr Elephant     QRYASNAISEALIREVANNV(G)VPLQDLMVRNDSPCGTTIGP
afr Rock_hyrax   QRYASNAVSEALIREVANKV(G)VPLQDLMVRNDSPCGTTIGP
afr Tenrec       QRYASTAILEALIRELANNV(G)VPLQDLMVRNDSPCGTTIGP
xen Armadillo    QRYASNAVSEALIREVANHV(G)VPLQDLMVRNDSPCGTTIGP
mar Opossum      QRYASNAVSEALIREVASRV(G)VPLQEFMVRNDSPCGSTIGP
mar Wallaby      QRYASNAVSEALIREVAARV(G)VPLQXFMVRNDSRCGTTIGP

NO 65
GN DNTTIP2
IP IPI00290410.3
PE PKVTPTKESYTEE
MP 132
EX Kim
CL simians
DE deoxynucleotidyltransferase, terminal, interacting protein 2
SQ
pri Human        ILIACSPVSSVRKKPKVTPT(K)ESYTEEIVSEAESHVSGISR
pri Chimpanzee   ILIACSPVSSVRKKPKVTPT(K)ESHTEEIVSEAESHVSGISR
pri Gorilla      ILIACSPVSSVRKKPKVTPT(K)ESYTEEIVSEAESHVSGISR
pri Orangutan    ILIACSPVSSVRKKPKVTPT(K)ESYTEEIVSEAESHVSGISR
pri Gibbon       ILIACSPVSSVRKKPKVTPT(K)ESYTEEIVSEAESHVSGISR
pri Baboon       ILIACSPVSSVRKKPKVTPT(K)ESYTEEIVSEAESHVSGISR
pri Marmoset     ILIACPPVSSVRKKLKVTPT(K)ESYTEEVVSEAESHDSGISR
pri Tarsier      VLIACPPVSSVRKRSKITPT(N)VS--EEIVSEADSHASGISR
pri Bushbaby     ILIACPPVSSVKKRPKITIT(N)ESHNEEVVSEAESHVSGISR
pri Mouse_lemur  ILIACPPASSVRKRPKITPT(D)ESHTEEVVSEAESHVSGISR
eua Treeshrew    ILIACTPMSKIRKKTKITPI(N)ESHTEEVVSEAESHVSGISR
eua Mouse        IVIASTSKSTVRKRQKVAPQ(H)ASADEVVVSEAESHVSGVSM
eua Rat          VVIVPTPKSSVRKRQKVTST(H)ESGDEGAVSEAESHVSGVSM
eua Guinea_pig   IIIAPTPASTVRKKQKITAL(N)ESVAEEIVSEVESHTSGVSR
eua Rabbit       ILIAPTPVSSVRKRLKVTPV(N)ESHTEEVVSEAESHASGVSR
eua Pika         IVIAPTPVS--RKRPKVTTV(S)ESQAEEVVSEAESHTSGVTR
lau Dog          ILVACTPVPSVTKRPKITPA(H)QSPTEEDDSEAESHVSGISR
lau Cat          ILVACTPVSSVRKRLKITPI(N)QSHAEEDDSEAESHVSGISR
lau Horse        ILIACTPVSSARKRLKITPI(N)ESHTEE-VSEAESHVSGISR
lau Cow          ILVAGTPVSSVRKRLKITRV(S)ESHTEEVVSEADSHVSGISR
lau Dolphin      ILVAGTPVSSVRKRLKITRV(S)ESHTEEEVSEAESHISGISR
lau Alpaca       ILVAGTPVSSVRKRLKITPV(K)ESHTEE--SEAESHVLGISR
lau Megabat      ILVACTPVSSVRKKLKITPI(N)ESHTEEELSEAESHVSGISR
lau Microbat     ILVPCTPVSSVKKRLKITSI(S)ESHTEEEVSEAESHVSGISR
afr Elephant     ILVSCTPASSVRKRPKITPV(V)ESHTEE--SEAESHVSGISA
afr Rock_hyrax   ILVSCMPVSSVRKKLKITPV(S)ESRIEEEVSEAESHVSGIST
afr Tenrec       IIIPSTPVSSARKKVKISPI(S)ESHPEEEVSEAESHVSGISA
xen Armadillo    ILVPCTPLSSVRKRPKITPI(S)ESHTEEEVSEAESHVSGISR
xen Sloth        ILVPHTPVSSVRKRLKITPI(N)ESHTKE--SEAESHISGISR
mar Opossum      IIIPCQPESKPRSRHKTTRT(I)KSGDEGEVSESESCSSVLSS
mar Wallaby      IIIPCQPEAKARSRPKTART(D)KSGDEGEVSEAESCSSALSS

NO 66
GN DPH1
IP IPI00718991.3
PE IPPEILKNPQLQA
MP 43
EX Kim
CL catarrhines
DE DPH1 homolog (S. cerevisiae)
SQ
pri Human        GRGRAP-RGRVANQIPPEIL(K)NPQLQAAIRVLPSNYNFEIP
pri Chimpanzee   GRGRAP-RGRVANQIPPEIL(K)NPQLQAAMRVLPSNYNFEIP
pri Gorilla      GRGRAP-RGRVANQIPPEIL(K)NPQLQAAIQVLPSNYNFEIP
pri Orangutan    GRGRAP-RGRVANQIPPEIL(K)NPQLQAAIQVLPSNYNFEIP
pri Gibbon       GRGRAP-RGRVANQIPPEIL(K)SPQLQAAVRALPSNYNFEIP
pri Rhesus       XXXRAP-RGRVANQIPPEIL(K)NSQLQAAIQVLPSNYNFEIP
pri Baboon       GRVRAP-RGRVANQIPPEIL(K)NPQLQAAIQVLPSNYNFEIP
pri Marmoset     GRGPVP-RGRVANQIPPEIL(N)NPQLQAAVQVLPSNYNFEIP
pri Tarsier      ARGRAY-RGRLANQVPPEIL(N)NPQLQTAIRVLPSNYNFEIP
pri Bushbaby     GGGRAL-RGRLANQIPPEIL(N)NPQLQAAIQVLPSNYNFEIP
pri Mouse_lemur  GRGRPP-RGRLANQIPPEIL(N)NPQLQAAIQVLPSNYNFEIP
eua Mouse        GRGRIS-RGRLANQIPPEVL(N)NPQLQAAVQVLPSNYNFEIP
eua Rat          GRGRIS-RGRLANQIPPEIL(N)SPQLQAAVHALPSNYNFEIP
eua Kangaroo_rat XXGRAP-RGRLANQIPPEIL(N)NSQLQAAIQALPSNYNFEIP
eua Guinea_pig   GRGRAT-RGRLANQIPLEIL(N)NPQLQAAIQALPSNYNFEIP
eua Squirrel     XXXRTS-RGRLANQIPPEIL(N)NPQLQTAIKVLPSNYNFEIP
eua Rabbit       GRGRVP-RSRLANQIPPDIL(N)NPQLQAAIQVLPSNYNFEIP
eua Pika         GRGRGP-RGRLANQIPADIL(S)DPQLQAAIQVLPSNYNFEIP
lau Dog          SRGRTP-RGRLANQIPPEIL(N)NPQLQAAIQVLPSNYNFEIP
lau Cat          DRGRAP-RGRLANQIPPEIL(N)NPHLQAAIQVLPSNYNFEIP
lau Horse        GRGRVP-RGRLANQIPPEIL(N)NPQLQAAIRVLPSNYNFEIP
lau Cow          GRGRAP-RGRLANQIPAEIL(N)NPQLQAAIQVLPSNYNFEVP
lau Dolphin      XXGRAP-RGRLANQIPAEIL(N)NPQLQAAIQVLPSNYNFEIP
lau Alpaca       XXGRVP-RGRLANQIPPEIL(N)NPQLQAAIQVLPSNYNFEIP
lau Microbat     GRGRVP-RGRLANQIPPEIL(N)NPQLQAAIQVLPSNYNFEIP
lau Shrew        XXGQPL-RGRLANQIPPEIL(N)NPQLQAAIQVLPSNYNFEIP
afr Elephant     GRGRVL-RSRLANQIPPEIL(N)NPQLQAAIQVLPSNYNFEIP
afr Rock_hyrax   XXGRVP-LGRLANQIPADIL(N)NPQLQAAIQVLPSNYNFEIP
xen Armadillo    XXGRAP-RGRLANQIPPEIL(N)NTQLRAAIQVLPSNYNFEIP
mar Opossum      GKGRGPLRGRLANQIPPEIL(G)DPRLKEAVQALPSNYNFEIP
mar Wallaby      XXRGPP-RGRLANQIPTEIL(G)DPQLQEAIQVLPSNYNFEIP
pro Platypus     XXGTS--RGRLANQIPPEIL(G)DAGLREALRALPDNYNFEIP

NO 67
GN DSC3
IP IPI00031549
PE SGRGVDKEPLNLF
MP 180
EX Wagner
CL humans
DE desmocollin 3
SQ
pri Human        ESDAAQNYTVFYSISGRGVD(K)EPLNLFYIERDTGNLFCTRP
pri Chimpanzee   ESDAAQNYTVFYSISGRGVD(Q)EPLNLFYIERDTGNLFCTQP
pri Gorilla      ESDAAQNYTVFYSISGRGVD(Q)EPLNLFYIERDTGNLFCTQP
pri Orangutan    ESDAAQNYTIFYSISGRGVD(Q)EPLNLFYIERDTGNLFCTQP
pri Gibbon       ESDAAQNYTVFYSISGRGVD(Q)EPLNLFYIERDTGNLFCTRP
pri Rhesus       ESDAAQNYTVFYSISGRGVD(Q)EPLNLFYIERDTGNLFCTQP
pri Baboon       ESDAAQNYTVFYSISGRGVD(Q)EPLNLFYIERDTGNLFCTQP
pri Marmoset     ESDAAQNYTVFYSITGRGVD(Q)EPLNLFYIERDTGNLFCTQR
pri Tarsier      QSDAAQNYTIFYSISGHGVD(Q)EPLNLFYIDRNTGNLYCTQP
pri Bushbaby     QSDAAQNYTIFYSISGRGVD(Q)EPLNLFYIERDTGNLYCTKP
pri Mouse_lemur  QSDAAQNYTIFYSISGRGVD(Q)EPLNLFYIERDTGNLFCTKP
eua Treeshrew    QSDAAQNYTVFYSISGRGVD(Q)EPLNLFFIDRNSGNLFCTRP
eua Mouse        QSDAAQNYTVFYSISGRGAD(Q)EPLNWFFIERDTGNLYCTRP
eua Rat          QSDSAQNYNIFYSISGRGVD(Q)EPLNLFFIERDTGNLYCTRP
eua Kangaroo_rat LSDAAQNYTIFYSISGRGVD(Q)EPLNLFYIERDTGNLYCTRP
eua Guinea_pig   QSDAAQNYTVFYSISGRGVD(Q)EPLNLFFIDRDTGNLFCTRP
eua Squirrel     QSDAAQNYTVFYSISGRGVD(Q)EPLNLFFIERDTGNLYCTQP
eua Rabbit       QSDAAQNYTVFYSISGRGVD(Q)EPLNLFYIERDTGNLYCTRP
eua Pika         QSDAAQNYTIYYSISGRGVD(Q)EPLNLFYIERDTGNLYCTRP
lau Dog          QSDAAQNYTIFYSISGRGVD(Q)EPLNLFFIERDTGNLYCTRP
lau Cat          QSDAAQNYTIFYSISGHGVD(Q)EPLNLFFIERDTGNLYCTRP
lau Horse        QSDAAQNYTIFYSISGRGVD(Q)EPLNLFFIDRDTGNLYCTRP
lau Cow          QSDAAQNYTIFYSISGRGVD(K)EPLNLFFIERDTGNLYCTQP
lau Dolphin      QSDAAQNYTVFYSISGRGVD(Q)EPLNLFFIERDTGNLYCTRP
lau Alpaca       QSDAAQHYTIFYSISGPGVD(Q)EPLNLFFIEKDTGNLFCTRA
lau Megabat      QSDAAQNYTVFYSISGRGVD(Q)EPLNLFFIEKDTGNLFCTRP
lau Hedgehog     QSDAAQNYTIFYSISGRGVD(Q)EPLNLFFIERDTGNLYCTRP
afr Elephant     QSDAAQNYTIFYSISGRGVD(K)EPLNLFYIEKDTGNLYCTRP
afr Rock_hyrax   QSDAAQNYTVFYSISGRGVD(Q)EPLNLFYIEKDTGNLYCTQP
afr Tenrec       QSDAAQNYTIFYSIRGRGVD(Q)EPLDLFYIDKHTGNLYCTKP
xen Armadillo    QSDAAQNYTVFYSISGRGVD(Q)EPLNLFYIEKNTGNLYCTQR
xen Sloth        QSDAAQNYTIFYSISGRGVD(Q)DPLNLFFIEKDTGNLYCTRP
mar Opossum      QSDSAQKYTVYYSISGPGVD(Q)NPLNLFYIEKDTGNLFCTGP

NO 68
GN DTX3L
IP IPI00152503
PE EGRKVLKLLYRAF
MP 675
EX Wagner
CL African great apes
DE deltex 3-like (Drosophila)
SQ
pri Human        YPGIQRTAYLPDNKEGRKVL(K)LLYRAFDQKLIFTVGYSRVL
pri Chimpanzee   YPGILRTAYLPDNKEGRKVL(K)LLCRAFDQKLIFTVGYSRVL
pri Gorilla      YPGIQRTAYLPDNEEGRKVL(K)LLCRAFDQKLIFTVGYSRVL
pri Orangutan    YLGIQRTAYLPDNKEGRKVL(E)LLCRAFDQKLIFTVGYSRVS
pri Gibbon       YSGIQRTAYLPDNKEGRKVL(E)LLRRAFDQKLIFTVGYSRVL
pri Rhesus       YSGTQRTAYLPDNKEGRKVL(E)LLHRAFDQKLIFTVGYSCVS
pri Baboon       YSGTQRTAYLPDNKEGKKVL(E)LLHKAFDQKLIFTVGYSRVS
pri Marmoset     YPGVHRTAYLPDNKEGRKVL(E)LLRRAFEQKLIFTVGNSRAL
pri Bushbaby     YSGIQRTAYLPDNEEGKEVL(G)LLRRAFKQKLIFTVGYSRTL
pri Mouse_lemur  YSGIQRTAYLPNNEEGMKVL(R)LLQRAFDQKLIFTVGESRVS
eua Mouse        YHGTRRTAYLPDNTEGRKVL(D)LLHEAFKHRLTFTIGYSRAT
eua Rat          YSGTCRVAYLPDNAEGRKVL(H)LLRKAFDQKLIFTVGESRVL
eua Kangaroo_rat YYGTQRTAYLPNNKEGREVL(D)LLKIAFEQKLIFTVGDSRVL
eua Guinea_pig   YSGTSRTAYLPDNKEGNEVL(E)LLREAFNHRLIFTVGYSRTL
eua Squirrel     YHEIQRTAYLPDNKEGKKVL(E)LLSKAFEQKLIFTVGQSRTS
eua Rabbit       YPGTQRTAYLPDNREGREVL(E)LLRMAFDQKLIFTVGESRAL
eua Pika         YPGTQRIAYLPDNKEGREVL(E)LLRVAFKQKLIFTVGFSRVL
lau Dog          YSGVQRTAYLPNNEEGNKVL(R)LLCRAFEQKLIFTVGESRTL
lau Horse        YLGIHRTAYLPDNKEGNEVL(R)LLRRAFDQKLIFTVGDSRVT
lau Cow          FSGIRRTAYLPDNKEGNEVL(R)LLRRAFDQKLIFTVGESRTL
lau Dolphin      FSGICRTAYLPDNEEGREVL(H)LLRRAFDEKLIFTVGDSRVL
lau Megabat      YKGIQRTAYLSDNEEGKEVL(T)LLRRAFDHKLIFTVGYSRVS
lau Microbat     YSGTRRTAFLPANEEGKEVL(T)LLQKAFNQKLIFTVGDSRVS
lau Hedgehog     YVGVHRTAYLPDNKEGKEVL(K)LLQRAFDQKLIFTVGYSRTT
lau Shrew        YYGVQRRAYLPDNEEGKEVL(K)LLQRAFEQKLIFTVGTSRTS
afr Elephant     YDGTHRTAYLPDNVEGQEVL(R)LLRKAFDQKLIFTVGQSRTS
afr Tenrec       YSATFRNAYLPDNEEGRDVL(R)LLRTAFERKLIFTVGQSQTT
xen Armadillo    YSGIKRTAFLPDNEEGREVL(R)LLRKAFDQKLIFTVGESRMT
xen Sloth        YCGIQRIAYLPNNKEGNEVL(V)LLRRAFDQRLIFTVGESRVT
mar Opossum      YSGTKRVAYLPDNEEGRQVL(Y)LLKRAFDQKLIFTVGHSRTT
mar Wallaby      YEGTRRVAYLPNNEEGRQVL(H)LLRRAFDQRLIFTVGQSQTS

NO 69
GN DZIP3
IP IPI00452463.1
PE LQEIGDKNDHWFD
MP 212
EX Kim
CL catarrhines
DE DAZ interacting protein 3, zinc finger
SQ
pri Human        QKRYNGGLLEFHKSLQEIGD(K)NDHWFDIDPTEDEDLPTTFK
pri Chimpanzee   QKRYNGGLLEFHKSLQEIGD(K)NDHWFDIDPTEDEDLPTTFK
pri Gorilla      QKRYNGGLLEFHKSLQEIGD(K)NDHWFDIDPTEDEDLPTTFK
pri Orangutan    QKRYNGGLLEFHKSLQEIGD(K)NDHWFDIDPTEDEDLPTTFK
pri Gibbon       QKRYNGGLLEFHKSLQEIGD(K)NDHWFDIDPTEDEDLPTTFK
pri Rhesus       XXXYNGGLLEFHKSLQEIGD(K)NDHWFDIDPTEDEDLPTTFK
pri Marmoset     QKRYNGGLLEFHKGLQEIGD(I)NDHWFDIDPTEDEDLPTTFK
pri Tarsier      QKRCNGGLLEFHKSLQEIGD(P)NDHWFDIDPTEDKDLSTTFE
pri Bushbaby     QKRYHGGLLEFHKSLQEIGD(I)NDHWFDVDPTEDEDLPTTFK
eua Treeshrew    QKRYNGGLLEFHKSLQEIGD(A)NDHWFDLDPTEDEDLPTTFK
eua Mouse        QKRYNGALLEFYKSLQEIGD(T)DDNWFEVDPTDDEDLPTTFK
eua Rat          QKRYNGALLEFYKSLQEIGD(T)DDSWFEVDPTDDEDLPTTFK
eua Guinea_pig   QKKYNGGLLEFHKSLQEIGD(I)NDHWFDIDPTEDEDLPTTFK
eua Squirrel     XXXYNGGLLEFHKSLQEIGD(T)SDHWFDIDPTEDGDLPTTFK
eua Rabbit       QKRYNGGLLEFHKSLQEIGD(T)NDHWFDIDPTEDEELPTSFK
lau Dog          QKRYNGGLLEFHKSLQEIGE(T)NDHWFDIDPTEDEDLPTTFK
lau Horse        QKRYNGGLLEFHKSLQEIGD(T)NDHWFDIDPTEDEDLPTTFK
lau Cow          QKRYNGGLLEFHKSLQEIGD(T)DDHWFDIDPTEDEDLPTTFK
lau Dolphin      QKRYNAGLLEFHKSLQETGD(T)DDHWFDIDPMENEDLPTTFK
lau Alpaca       QKRYNGGLLEFHKSLQEIGD(T)DDHWFEIDPTEDEDLPTTFK
lau Megabat      QKRYNGGLLEFHKSLQEIGD(T)NDHWFDIDPTEDKDLPTTFK
lau Hedgehog     QKRYNGGLLEFHKSLHEIGD(T)NDHWFDIDLTEDEDLPSTFK
afr Elephant     QKRYNGGLLEFHKSLQEIED(P)NDYWFDIDPTGDEDLPTTFK
afr Tenrec       QKRYNGGLLEFYKSLHEIED(P)SDYWFDIDPTEDDNLPTTFK
xen Sloth        QKRYNGGLLEFHKSLQEIED(T)NDHWFDIDPAEDEELPTTFK
mar Opossum      QKRYIGGLLEFQKSKQEIQD(N)NENWYDIDHIKNEDLPETFQ
mar Wallaby      QKRYNGGLLEFQKSKQEIKD(N)NENWYGINHIKDEDLPETFQ

NO 70
GN ECHDC1
IP IPI00302688.7
PE LMTPESKIRFVHK
MP 179
EX Kim
CL simians
DE enoyl CoA hydratase domain containing 1
SQ
pri Human        LGGGAEFTTACDFRLMTPES(K)IRFVHKEMGIIPSWGGTTRL
pri Chimpanzee   LGGGAEFTTACDFRLMTPES(K)IRFVHKEMGIIPSWGGTTRL
pri Gorilla      LGGGAEFTTACDFRLMTPES(K)IRFVHKEMGIIPSWGGTTRL
pri Orangutan    LGGGAEFTTACDFRLMTPES(K)IRFVHKEMGIIPSWGGTTRL
pri Gibbon       LGGGAEFTTACDFRLMTPES(K)IRFVHKEMGIIPSWGGTTRL
pri Rhesus       LGGGAEFTTACDFRLMTPES(K)IRFVHKEMGIIPSWGGTTRL
pri Baboon       LGGGAEFTTACDFRLMTPES(K)IRFVHKEMGIIPSWGGTTRL
pri Marmoset     LGGGAEFTTACDFRLMTPES(K)IRFVHKEMGIIPSWGGTTRL
pri Tarsier      LGGGAEFTTACDFRLMTPES(E)IRFVHKEMGIIPSWGGTTRL
pri Bushbaby     LGEGAEFTTACDFRLMTPDS(E)IRFVHKEMGIIPSWGGTSRL
pri Mouse_lemur  LGGGAELTTACDFRLMTPES(E)IRFVHKEMGIIPSWGGTTRL
eua Mouse        MGGGAELTTACDFRLMTEES(V)IRFVHKEMGIVPSWGGTSRL
eua Rat          MGGGAELTTACDFRLMTEES(V)IRFVHKEMGIVPSWGGASRL
eua Guinea_pig   LGGGAELTTACDFRLMTPES(E)IRFVHKEMGIIPSWGGASRL
eua Rabbit       LGGGAEVTTACDFRLMTPES(E)IRFVHKEMGIIPSWGGATRL
lau Dog          LGGGAEVTTACDFRLMTADG(E)IRFVHREMGIVPSWGGGTRL
lau Cat          LGGGAEFTTACDFRLMTPES(E)IRFVHKEMGIIPSWGGATRL
lau Horse        MGGGAEVTTACDFRLMTRES(E)IRFVHKEMGIVPSWGGAARL
lau Cow          LGGGAEVTTACDFRLMTTES(E)IRFVHKEMGIIPSWGGATRL
lau Dolphin      LGGGAEVTTACDFRLMTTES(E)IRFVHKEMGVIPSWGGATRL
lau Alpaca       LGGGAEITTACDFRLMTADS(E)IRFVHKEMGIIPSWGGATRL
lau Microbat     LGGGAEVTTACDFRLMTRES(E)IRFVHKEMGIIPSWGGATRL
lau Hedgehog     LGGGAEVTTACDFRLMTKGS(E)IRFVHKEMGIIPSWGGATRL
lau Shrew        MGGGAEFTTACDFRLMTKES(E)IRFVHKEMGIVPSWGGATRL
afr Elephant     LGGGAEVTTACDFRLMTPGS(E)IRFVHKEMGIIPSWGGTTRL
afr Tenrec       MGGGAEFTTACDFRLMTPES(E)IRFVHKEMGIIPSWGGTTRL
xen Armadillo    LGGGAEFTTACDFRLMTPGS(E)IRFVHKEMGIIPSWGGTARL
xen Sloth        LCGGAEFTTACDFRLMTPDS(E)IRFVHKEMGIVPSWGGTSRL
mar Opossum      MGGGAEFTTACDFRLMTPSS(E)IRFVHKEMGITPSWGGAARL
pro Platypus     MGGGAELTTACDFRLMTPES(E)IRFVHKEMGIVPSWGGAARL

NO 71
GN EIF2AK2
IP IPI00019463.3
PE LFEQITKGVDYIH
MP 400
EX Kim
CL catarrhines
DE eukaryotic translation initiation factor 2-alpha kinase 2
SQ
pri Human        KRRGEKLDKVLALELFEQIT(K)GVDYIHSKKLIHRDLKPSNI
pri Chimpanzee   DRRGKKLDKVLALELFEQIT(K)GVDYIHSKKLIHRDLKPSNI
pri Gorilla      NRRGEKLDKVLALELFEQIT(K)GVDYIHSKKLIHRDLKPSNI
pri Orangutan    NTRGKKLDKVLALELFEQIT(K)GVDYIHSKKLIHRDLKPSNI
pri Gibbon       NRRGKKLDKVLALEFFEQIT(K)GVDYIHSKKLIHRDLKPSNI
pri Baboon       DRRGKKLDKVLALELFEQIT(K)GLDYIHSKNIIHRDLKPSNI
pri Marmoset     DRRGKKLDKVLSLEFFEQIT(T)GVDYIHSKNLIHRDLKPSNI
pri Tarsier      DRMGEKPEKALVLEFFEQIT(T)GVDYIHSKNLIHRDLKPLNI
pri Bushbaby     NRRGKKPDKALALEFFEQIT(K)GVDFIHSKQLIHRDLKPSNI
pri Mouse_lemur  CRKGEKLDKELALKFFEQIT(T)GVDYIHSKQLIHRDLKPSNI
eua Mouse        NRNQSKVDKALILDLYEQIV(T)GVEYIHSKGLIHRDLKPGNI
eua Rat          KRNRSQEDKALVLELFEQIV(T)GVDYIHSKGLIHRDLKPGNI
eua Kangaroo_rat AGRLNKSDKTMALNLFEQIV(T)GVGYIHSKGLIHRDLKXXXX
eua Guinea_pig   QRRGNYSDKALGLELFEQIT(T)GVCYVHSKNIIHRDLKPGNI
eua Rabbit       NRRQQKSEKALALEFFWQIT(T)GLKYIHSKELIHRDLKPNNI
lau Dog          NRRGKEQDKPLALELFEQIV(A)GVNYIHSKQLIHRDLKPGNI
lau Cat          SRRGQTPDKHLALELYEQIT(A)GVDYIHCKQLIHRDLKXXXX
lau Horse        NRRGQETDKQLSLEFFEQIT(T)GVDYIHSKELIHRDLKPSNI
lau Cow          KRRGKKPDKRLALDFFQQIT(T)GVHYIHSEQLIHRDLKPGNI
lau Dolphin      SRRGKEQDKRLALVFFEQIT(E)GVHYIHSKQLIHRDLKPHNI
lau Alpaca       SRRGKKPNKRLALEFFEKIT(A)GVHYIHSNQLIHRDLKPSNI
lau Megabat      NRRDQKTDKHLSLELFEQIA(K)GVNFIHLKGLIHRDLKPSNI
lau Microbat     IRRGEIPNKDLSLGLFKQIV(T)GVDYIHSEGLIHRDLKPSNI
lau Shrew        SRRSQTPSKALALQFFEQIV(E)GVNYIHGRGFIHRDLKPSNI
afr Elephant     SRRGKQSDKDLALDLFEQIT(K)GVAYIHSQRLIHRDLKPSNI
afr Rock_hyrax   KRRGEKPDKDSALNLFEQIT(K)GVDYIHSKELIHRDLKPSNI
xen Armadillo    KRRDKESDKNLALDLFEQIT(K)GVAYIHSKQLIHRDLKXXXX
xen Sloth        NRRHKKSNKDLSLDLLEQIT(T)GVAYRHSKQLIHRDRKPSNI
mar Opossum      ERRSIGSDKILSLKLFQQIT(A)GVEYIHSENLIHRDIKPSNI
pro Platypus     QRRNKESDKALSLNIFQQIT(S)GVEYIHSQKLIHRDLKPANI

NO 72
GN ENDOD1
IP IPI00001952.5
PE RMVQSQKSSSPLS
MP 300
EX Kim
CL catarrhines
DE endonuclease domain containing 1
SQ
pri Human        KKILEVVNQIQDEERMVQSQ(K)SSSPLSSTRSKRSTLLPPEA
pri Chimpanzee   KKILEVVNQIQDEERMVQSQ(K)NSSPLSSTRSKRSTLLPPEA
pri Gorilla      KKILEVVNQIQDEERMVQSQ(K)SSSPLSSTRSKRSALLPPEA
pri Orangutan    KKILEVVNQIQDEERMVQSQ(K)SSSLLSSTRSKRSAPLPPEA
pri Gibbon       KKILEVVNQIQDEERMVHSQ(K)SSSPLSSTRSKRSAPLPPEA
pri Rhesus       KKILEVVNQVQDEERMVQSQ(K)TSSPPSSTRSKRSAPLPPEA
pri Baboon       KKILEVVNQVQDEERMVQSQ(K)TSSPPSSTRSKRSAPLPPEA
pri Marmoset     KKILEVVNQVQDEERMVQSQ(E)SSSPLSSTRSKRSALLPPEA
pri Mouse_lemur  KKILEMVNQVQDEERQVQSQ(E)RAIPLSSTRSKRSALLPPEP
eua Treeshrew    KKILEMVNQVQDEERNVQSQ(E)STSPLSSPRSKRSALLPPEG
eua Mouse        KKILEVVNQVQDEERSLQSQ(E)RMSPLASTQSQRSALLSPEA
eua Rat          KKILEVVNQVQDEERSLQSQ(E)NMSPLASTRSQRSALLSPEA
eua Kangaroo_rat KKILEVVNQVQDEERALQSQ(E)STVSPAGARSQRAALGPPEP
eua Guinea_pig   KKILEMVNQVQDEERKVQSQ(G)SSIPLSSTRSKTDALLAPEP
eua Squirrel     KKILEMVNQVQDEERMVQSQ(E)SSSPLPSTKSKRSALLPPET
eua Rabbit       KKILEMVNQVQDEERMVQSQ(G)NSVPLSSTRSKRSALLPPEA
eua Pika         KKILEMVNQVQDEERMVESQ(G)SSVPLTSTRSKRSAPLPPEA
lau Dog          KKILEVVNQVQDEERTVLSK(E)DTRPLSSMTSEGSALLPPEA
lau Cat          KKILEVVNQVQDEERAVQWE(E)GASARSSAKSERSALLPPEA
lau Horse        KKILEMVNQVQDEERMVQSK(E)GSGPLSSTRSKKSALLPPEA
lau Cow          KKILEVVNQVQDEERRLDSE(G)GSEMLSSARGTRSALPPPET
lau Dolphin      KKILEMVNQVQDEERRVDSE(G)DAGTLSSTRSTRSTLLPPEA
lau Megabat      KKILEMVNQVQDEERTVRSK(E)TSSPLSSTRSKKSALLPPEA
lau Microbat     KKILEVVNQVQDEERMVQSK(E)TTSALPSTRSKRSALQPPEA
afr Elephant     KKILEMVNQVQDEERTVQSK(V)IPVPLSSTKNKRSAVLSPEA
afr Rock_hyrax   KKILEMVNQVQDEERVQQSK(A)SSVPLSSTRNKRSALLSPEA
afr Tenrec       KRILEMVNRVQDEERVLLSQ(E)SAVPLSSTKSKRSALLPPEA
xen Sloth        KKILEMVNQVQDEERMVQSK(E)SSIPLSSTRGKRSALLPPEA
mar Opossum      KKILEMVNHVQDEERMLQAK(S)SSEQLSGTRNKRSAQLLPD-
mar Wallaby      KKILEMVNHVQDEERTVQAK(S)SSSQLSSTRNKRSAQSLPE-
pro Platypus     KKILEMVNQVQDEERRGQTS(P)GTQPLSTMKSLKSTQLLREE

NO 73
GN EPB41L2
IP IPI00015973.1
PE AEEMAQKKQEIKV
MP 135
EX Kim
CL simians
DE erythrocyte membrane protein band 4.1-like 2
SQ
pri Human        EEPLPEEQRQAKGDAEEMAQ(K)KQ-EIKVEVKEEKPSVSKEE
pri Chimpanzee   EEPLPEEQRQAKGDAEEMAQ(K)KQ-EIKVEVKEEKPSVSKEE
pri Gorilla      EEPLPEEQRQAKGDAEEMAQ(K)KQ-EIKVEVKEEKPSVSKEE
pri Orangutan    EEPLPEEQRQAKGDAEEMAQ(K)KQ-EIKVEVKEEKPSVS---
pri Gibbon       EEPLPEEQRQAKGDAEEMAQ(K)KQ-EIKVEVKEEKPSVSK--
pri Rhesus       EEPLPEEQRQDKGDAEEMAQ(K)KQ-EIKVEVKEEKPSVS---
pri Baboon       EEPLPEEQRQDKGDAEEMAQ(K)KQ-EIKVEVKEEKPSVS---
pri Marmoset     EEPLLEEQRQAKGDAEEMVQ(K)KQ-EIKVEVKEEKPSVS---
pri Tarsier      EEPLPEEERQAKGDAEETAQ(R)KQ-EIKLEAKEEKPSVS---
pri Bushbaby     EEPLPEEERQVKGDXXEAAQ(R)KL-EIKVEVKEEKPPVG---
pri Mouse_lemur  EEPLPEEERQAKGDAEEMAQ(R)KL-EIKVESKEEKSSVS---
eua Mouse        EESLPEEESRAKGDAEEMAQ(R)KHLEVQVEVREAKPALK---
eua Rat          EDTLPEEESRAKGDAEEMAQ(R)KHLEVKVEVREEKSALK---
eua Kangaroo_rat EDSLPLEERRAKGDAEETVQ(R)KQXEVKVEVKEEKPSVS---
eua Guinea_pig   EDSLPEEERKATGNTEEVAQ(R)THLEVKVDVREDQPAEG---
lau Dog          EESSPEGERPHQAEAQERAG(S)RQQEVTAGVKDEKPEKP---
lau Horse        EEPFPEEERQAKGSAEVAAQ(R)KQQETKVDVKEEKPAVR---
lau Cow          EESLPEEERRAKGDAEETAQ(R)KQQEIKVDVKEEKPLLV---
lau Dolphin      EESLPEEERQAKGDAEETAQ(R)KQXXIKVDVKEEKPVVS---
lau Alpaca       EESLPEEEQLAKGDADETAQ(R)KQXXMKADVKEEKPSVS---
lau Microbat     EESLPEEERQAKGDAEEAVQ(R)KQQVIKVDIEEEKPSVS---
afr Elephant     EESLPREERQAKGDAEETAQ(R)KQQEIKAQVKEEQPSVS---
afr Rock_hyrax   EESLLKEERQAKGDAEESAQ(R)KQXEIKAEIKEEQPSVS---
afr Tenrec       EESLPEEERQAKGDAEETAQ(R)RLXEVKVEVKEDKPSTS---
xen Armadillo    EESLLEGERQAKGDAEETGA(R)KHXKIMVEVKEEKPSVS---
pro Platypus     EDVLPEEERPAKGDSEETAD(R)KQXDAKVEDKDEKRSVS---

NO 74
GN EPCAM
IP IPI00296215.2
PE LRTALQKEITTRY
MP 168
EX Kim
CL apes
DE epithelial cell adhesion molecule
SQ
pri Human        LKHKAREKPYDSKSLRTALQ(K)EITTRYQLDPKFITSILYEN
pri Chimpanzee   LKHKAREKPYDGKSLRTALQ(K)EITTRYQLDPKFITNILYEN
pri Gorilla      LKHKAREKPYDGKSLRTALQ(K)EITTRYQLDPKFITNILYEN
pri Orangutan    LKHKAREKPYDGKSLRTALQ(K)EITTRYQLDPKFITNILYEN
pri Gibbon       LKHKAREKPYDGKSLRTALQ(K)EITTRYQLDPKFITNILYEN
pri Rhesus       LKHKAREKPYDVQSLRTALE(E)AIKTRYQLDPKFITNILYED
pri Baboon       LKHKAREKPYDVQSLRTALE(E)AIKTRYQLDPKFITNILYED
pri Marmoset     LKHKAREKPYDVQSLRDALQ(E)AITTRYHLDGKFITNVVYEN
pri Tarsier      LKHKAREKPYDLQGLQIALR(N)VITNRYQLDSKYIANILYEN
pri Bushbaby     LKHKTREKPYDVRSLQLALK(D)MLTTRYLLDSNYIESILYEN
pri Mouse_lemur  LKHKAREKPYDVQSLQSALQ(E)TITTRYQLDPKYITNILYEN
eua Mouse        LKHKERESPYDHQSLQTALQ(E)AFTSRYKLNQKFIKNIMYEN
eua Rat          LKHKERAQPYNFESLHTALQ(D)TFASRYMLNPKFIKSIMYEN
eua Guinea_pig   LKHKERKQPFDLQSLQAAIQ(E)VLTKRYLLNPKFITDIVYEN
eua Squirrel     LKHKAREKPYNVQSLQTAIQ(E)MMTTRYLLDPKFXXXXXXXX
eua Rabbit       LKHKSREKPYDTQSLQTALQ(E)LISSRYLLDPKFITNILYEN
eua Pika         LKHKERQKPYDTQSLRKAIE(Q)LITSRYLLDPKFITDILYEN
lau Dog          LKHKTRETPYDTQSLQNALK(E)TLKNRYQLDPKYITNILYEN
lau Cat          LKHKTRETPYDIKSLQTALK(E)VITTRYQLDPKYITNILYEN
lau Horse        LKHKTREKPYDVQSLQNALK(E)TITNRYQLDPKYIADILYEN
lau Cow          LKHKTREKPYDLQSLQSALK(D)VITNRYQLDPKYITNILYEN
lau Dolphin      LKHK-REKPYDVQSLQAALK(E)VITSRYQLDPKYITNILYEN
lau Alpaca       LKHKAREKPYDVQSLQTALK(E)VITSRYQLDPKYITNILYEN
lau Megabat      LKHKTREKPYDIQSLQTALK(K)IITTRYQLDPKYITNILYEN
lau Microbat     LRHKTREKPYNSQSLQTALK(E)IITTRYQLDSKYIGNILYEN
lau Hedgehog     LKHKARETPFEVESLKSALK(Q)VITSRYQLDPKYITNIVYEN
afr Elephant     LKHKTRDKPYDLQSLRTALE(K)AITTRYQLDRKYITNILYEN
afr Rock_hyrax   LKHKTREKPYDLQSLQTALQ(D)VFSTRYLLDRKYITNILXXX
afr Tenrec       LRHKSREKPYNVESLRLALE(E)AFATRYQLNRKYITNILYEN
xen Armadillo    LKHKTREKPYDVESLRYALE(T)AITTRYQLDPKYITNILYEN
xen Sloth        LKHKTREKPYDFESLRTALE(E)AITTRYQLDPKYITNILYEN
mar Opossum      LKHKTREKPFNTTILENALK(E)EFQRRYQLNKKYVQNILYEN

NO 75
GN ERCC2
IP IPI00442420.2
PE ESEETLKRIEQIA
MP 701
EX Kim
CL humans
DE excision repair cross-complementing rodent repair deficiency, complementation group 2
SQ
pri Human        EDQLGLSLLSLEQLESEETL(K)RIEQIAQQL*
pri Chimpanzee   EDQLGLSLLSLEQLESEETL(Q)RIEQIAQQL*
pri Gorilla      EDQLGLSLLSLEQLESEETL(Q)RIEQIAQQL*
pri Orangutan    EDQLGLSLLSLEQLESEETL(Q)RIEQIAQQL*
pri Rhesus       EDQLGLSLLSLEQLESEETL(Q)RIEQIAQQL*
pri Baboon       EDQLGLSLLSLEQLESEETL(Q)RIEQIAQSC*
pri Marmoset     EDQLGLSLLSLEQLESQETL(Q)RIEQIAQQL*
pri Mouse_lemur  EDQLGLSLLSLEQLQSEETL(R)RIEQIAQQL*
eua Treeshrew    EDQLGLSLLSLEQLESEETL(R)RIEQIAQQL*
eua Mouse        EDQLGLSLLSLEQLQSEETL(Q)RIEQIAQQL*
eua Rat          EDQLGLSLLSLEQLQSEETL(Q)RIEQIAQQL*
eua Kangaroo_rat EDQLGLSLLSLEQLQSEETL(R)RVEQIAQQL*
eua Guinea_pig   EDQLGLSLLSLEQLESEEML(R)RIEQIAQQL*
eua Rabbit       EDQLGLSLLSLEQLESEETL(R)RIEQIAQQL*
lau Dog          EDQLGLSLLSLEQLESEETL(R)RIEQIAQQL*
lau Cat          EDQLGLSLLSLEQLESEETL(R)RIEQIAQQL*
lau Horse        EDQLGLSLLSLEQLESEETL(R)RIEQIAQQL*
lau Cow          EDQLGLSLLSLEQLESEETL(R)RIEQIAQQL*
lau Dolphin      EDQLGLSLLSLEQLESEETL(R)RIEQIAQQL*
lau Alpaca       EDQLGLSLLSLEQLESEETL(R)RIEQIAQQL*
lau Megabat      EDQLGLSLLSLEQLQSEETL(R)RIEQIAQQL*
lau Microbat     EDQLGLSLLSLEQLESEETL(R)RIEQIAQQL*
afr Elephant     EDQLGLSLLSLEQLQSEETL(R)RIEQIAQQL*
xen Armadillo    EDQLGLSLLSLEQLQSEETL(R)RIEQIAQQL*
mar Opossum      EDQLGLSLLSLEQLQSEETL(R)RIEQIAQQL*
mar Wallaby      EDQLGLSLLSLEQLQSEETL(R)RIEQIAQQL*

NO 76
GN FADS2
IP IPI00183786
PE SLKKSGKLWLDAY
MP 435
EX Wagner
CL catarrhines
DE fatty acid desaturase 2
SQ
pri Human        QEKPLLRALLDIIRSLKKSG(K)LWLDAYLHK*
pri Chimpanzee   QEKPLLRALLDIIRSLKKSG(K)LWLDAYLHK*
pri Gorilla      QEKPLLRALLDIIRSLKKSG(K)LWLDAYLHK*
pri Orangutan    QEKPLLRALLDIIRSLKKSG(K)LWLDAYLHK*
pri Gibbon       QEKPLLRALLDIIRSLKKSG(K)LWLDAYLHK*
pri Rhesus       QEKPLLRALLDIIRSLRKSG(K)LWLDAYLHK*
pri Baboon       QEKPLLRALLDIIRSLRKSG(K)LWLDAYLHK*
pri Marmoset     QEKPLLRALLDIIRSLKKSG(E)LWLDAYLHK*
pri Mouse_lemur  QEKPLLRALLDIIRSLKKSG(Q)LWLDAYLHK*
eua Treeshrew    QEKPLLRALRDIIRSLKKSG(D)LWLDAYLHK*
eua Mouse        QEKPLLRALIDIVSSLKKSG(E)LWLDAYLHK*
eua Rat          QEKPLLRALLDIVSSLKKSG(E)LWLDAYLHK*
eua Guinea_pig   QEKPLLQALLDIIRSLKKSG(Q)LWLDAYLHK*
eua Rabbit       QEKPLLKALLDIIRSLKKSG(E)LWLDAYLHK*
eua Pika         QEKPLPRALLDIIRSLKKSG(Q)LWLDAYLHK*
lau Dog          QEKPLLRALQDIIRSLKKSG(E)LWLDAYLHK*
lau Horse        EVKSVPTALVDIIRTLKKSG(N)VWLEAYLHQ*
lau Cow          QEKPLLRALQDIIGSLRKSG(Q)LWLDAYLHK*
lau Dolphin      QQKPLLQALQDIIRSLRQSG(Q)LWLDAYLHK*
lau Megabat      QEKRLLRALLDVIRSLKKSG(D)LWLDAYLHK*
lau Hedgehog     QEKPLLRALQDIVRSLKESG(E)LWLDAYLHK*
afr Elephant     QEKPLLRALLDIIGSLKKSG(Q)LWLDAYLHK*
afr Rock_hyrax   QEKPLLRALLDIVGSLKKSG(Q)LWLDAYLHK*

NO 77
GN FAM175A
IP IPI00030384.4
PE VQTHSSKFFEEDG
MP 201
EX Kim, Wagner
CL simians
DE family with sequence similarity 175, member A
SQ
pri Human        TVSGSCMSTGFSRAVQTHSS(K)FFEEDGSLKEVHKINEMYAS
pri Chimpanzee   TVSGSCMSTGFSRAVQTHSS(K)FFEEDGSLKEVHKINEMYAS
pri Gorilla      XXXXXXXXXXXXXXXXXXXS(K)FFEEDGSLKEVHKINEMYAS
pri Orangutan    TVSGSCMSTGFSRAVQTHSS(K)FFEEDGSLKEVHKINEMYAS
pri Gibbon       TVSGSCMSTGFHQAVQTHSS(K)FFEEDGSLKEVHKINEMYAS
pri Baboon       TVSGSCMSTGFSRAVQTHSS(K)FFEEDGSLREVHKINEMYAS
pri Marmoset     TVSASCMSTGFSRAVQTHSS(K)FFEEDGSLKEVHKINEMYAS
pri Mouse_lemur  TASGSCTSAGFNRAVEAHSS(E)FFEEDGSLKEVHKIKEMYAS
eua Mouse        TEPASCTSTVFSRAVRTHSS(Q)FFNEDGSLKEVHKINEMYAA
eua Rat          TESVSCTSTVFSRAVRTYSS(Q)FFNEDGSLKEVRKINEMYAA
eua Guinea_pig   TTSGSCASLGFSKAVRTHSS(E)FFKEDGSLKEVHKINEMYAC
eua Rabbit       TVSGSCMSTGFGRAVKTHRS(E)FFKDDGSLKEVHKINEMYAS
eua Pika         TVSGSCMSAGFSRAVKTHRS(E)FFKDDGSLKEVHRINEMYAS
lau Dog          TISGSCESTGFSRAVKTHSS(E)FFTEDGSLKEVHKINEMYAS
lau Horse        TVSGSCTSAGFSRAVKTHSS(E)FFKEDGSLKEVHKINEMYAS
lau Cow          TTSGSCTSAGFSRAVKTHSS(E)FFKEDGSLKEVQKINEMYTS
lau Alpaca       TVSGSCMSTGFSRAVKTHSS(E)FFKEDGSLKEVHKINEMYAS
lau Megabat      TVSGSRMSAGFSRAVKTHSP(E)FFKEDGSLKEVHKINEMYAS
lau Hedgehog     TVSSSCMSAGFSRAVKTHSS(E)FFKEDGSLKEVQKISEMYAS
lau Shrew        TVSGSCVSAGFHRAVRTHSS(A)FFKEDGSLQEIQKINEMYAS
afr Elephant     TVSGSCMSAGFNRAVKTHSS(E)FFKEDGSLKEVHKINEMYAS
afr Tenrec       TTSGSCTSTGFSRAVKTHSS(E)FFKEDGSLKEIHKINEMYAS
xen Armadillo    TVSGSCMSRGFGRAVKTHSS(E)FFKEDGSLKEVHKINEMYAS
xen Sloth        TVSSSCTSSGFGRAVKTHSS(E)FFKEDGSLKEVHKINEMYAS
mar Opossum      TVSGSCTSTAFGRTVHAHRS(E)FFNEDGTLKEVQKISELYGT
pro Platypus     TVSSSCTSTSFGRAVKTHRS(E)FFNEDGSLKEVHKINEMYAT

NO 78
GN FAM175A
IP IPI00030384.4
PE NQDKASKMSSPET
MP 384
EX Kim
CL simians
DE family with sequence similarity 175, member A
SQ
pri Human        DTQDKRSKADTGSSNQDKAS(K)MSSPETDEEIEKMKGFGEYS
pri Chimpanzee   DTQDKRSKADTGSSNQDKAS(K)MSSPETDEEIEKMKGFGEYS
pri Orangutan    DTQDKRSKTDTDSSNQDKAS(K)MSSPETDEEIEKMKGFGEYS
pri Gibbon       DTQDKRSKTDTDSSNQDKVS(K)MSSPETDEEIEKIKGFGGYS
pri Baboon       DTQDKRSKADTDSSNEDKAS(K)MSSPETDEEIEKMKGFGECS
pri Marmoset     DTQDKPSKTDTDSSNREKAS(K)MSSPETDEDIEKMKGFDEYS
pri Tarsier      ETQNKPSKTDTDSSNQEKTS(P)TSSPETDDEMEKTKGSVEYP
pri Bushbaby     EIENNPSKTDTDSSNQEKAT(T)ISSPETDEEIEKMKGSDEYP
pri Mouse_lemur  ETQNKPSKTDTDSSNQEKTS(T)ISSPETDEDIEKMKGSGEYS
eua Treeshrew    EIQNKPSSTDSDSSYQEKSS(T)LSSPETDEDIEKMKSSGEYP
eua Mouse        ETESRPSVAASRSRHQDKAS(S)-SSLDIDIEMGSPEDDADYP
eua Rat          ESESRPGPA-FRGSHQDKAS(S)-SSLDIDTEVGSPEDDTDYP
eua Kangaroo_rat ETESKLSKTDTDNENQERKF(T)SSSSDTDEDIEKMKGYEEYP
eua Guinea_pig   EPRNKLCETGTESSNGEKAS(A)TSSPETEDERERTQGADKYP
eua Squirrel     KREHKLSKTGTDSSNQEKAS(T)TSGTETDEEIEKMKGSGEYP
eua Rabbit       ERENKLFKTDADSSNQAKAS(A)MSSPETDEEIEKMKDSIAYP
eua Pika         EREEKRFEADSDSSNPDRVS(P)VSSLETDDDIEKMRDSGEYP
lau Dog          EIQNKPSKTDPDSSNQEKAS(T)VSSPETDEDIEKMKGSGEYP
lau Cow          GIQNRPSKTDTNSSNQEQAS(T)VSSPETDEEIERMKGSGEYP
lau Dolphin      EIQNQPSKTDTDSSNQEKAS(T)VSSPETDEEIEKMNGSGEYP
lau Alpaca       EIQNKPWKADTDSSNQEKAS(A)VSSPETDEEIEKMKGSGEYS
lau Hedgehog     ETPSKLSKTDTSSGNQEKAS(T)ISSPETDEEIEKMKGSGEYP
afr Elephant     EIQNNQSKTDTDSSNQEKAS(T)MSSPETDEDTEKTKGSGEYP
afr Tenrec       ETQNNQFKTDTDSSNHDRAS(A)GSSPETDEDMEKMKDSDEYP
xen Armadillo    EVQKRQSKTDIGSSNQEKAS(T)MSSPETDEEIVKMKGSGDYP
xen Sloth        EMQKRPSKTDTESSNQEKAS(T)ISSPETDEEIVKMKGSGDYP

NO 79
GN FAM82B
IP IPI00329696
PE PSSTYEKALGYFH
MP 243
EX Wagner
CL catarrhines
DE family with sequence similarity 82, member B
SQ
pri Human        WYQRRIAKMLFATPPSSTYE(K)ALGYFHRAEQVDPNFYSKNL
pri Chimpanzee   WYQRRIAKMLFATPPSSTYE(K)ALGYFHRAEQVDPNFYSKNL
pri Gorilla      WYQRRIAKMLFAAPPSSTYE(K)ALGYFHRAEQVDPNFYSKNL
pri Orangutan    WYQRRIAKMLFATPPTSTYE(K)ALSYFHRAEQVDPNFYSKNL
pri Gibbon       WYQRRIAKMLFATPPSSTYE(K)ALGYFHRAEQVDPNFYSKNL
pri Rhesus       WYQRRIAEMLFATPPSSTYE(K)ALGYFHRAEQVDPNFYSKNL
pri Baboon       WYQRRIAEMLFATPPSSTYE(K)ALGYFHRAEQVDPNFYSKNL
pri Marmoset     WYQRRIAEMLFASPPSSTYE(E)ALVYFHRAEQVDPNFYSKNL
pri Tarsier      WYQXRIAKMLFATPLTSTYE(E)ALGYFHRAEQVDPNFYSKNL
eua Treeshrew    WYQRRIAKLLFATPPSSTYE(E)ALGYFERAEQVDPNFYSKNL
eua Mouse        WYQRRIAKVLFANPPSSTYE(E)ALRYFHKAEEVDPNFYSKNL
eua Rat          WYQRRIAEVLFANPPSSTYE(E)ALKYFHRAEEVDPNFYSKNL
eua Kangaroo_rat WYQRRIAEMLFATPPSSTYE(E)ALGYFHRAEQVDPNFYSKNL
eua Guinea_pig   WYQRRIAKMLFATPPSSTYE(E)ALGYFHRAEQVDPNFYSKNL
eua Squirrel     WYQRRIAKMLFATPPSSTYE(E)ALGYFHRAEQVDPNFYSKNL
eua Rabbit       WYQRRIAKMLFATPPSSTYE(E)ALGYFHRAEQVDPNFYSKNL
eua Pika         WYQRRIAKMLFATPPSSTYE(E)ALGYFHRAEQVDPNFYSKNL
lau Dog          WYQRRIAKMLFATPPSSTYE(E)ALGYFHRAEQVDPNFYSKNL
lau Horse        WYQRRMANVLFAAPPSSTYE(E)ALGYFHRAEQVDPNFYSKNL
lau Cow          WYQRRIAKVLFATPPGSTYE(E)ALGYFHRAEQVDPNFYSKNL
lau Dolphin      WYQRRIAEMLFATPPSSTYE(E)ALGYFHRAEQVDPNFYSKNL
lau Alpaca       WYQRRIAKMLFATPPSSTYE(E)ALGYFHRAEQVEPNFYSKNL
lau Megabat      WYQRRIARMLFAAPPSSTYE(E)ALGYFQRAEQVDPNFYSKNL
lau Hedgehog     WYQRRIAKVLFATPPSSTYE(E)ALGYFYKAEQVDPNFYSKNL
lau Shrew        WYQRRIAKMLFATPPSSTYE(E)ALGYFQRAEQVDPNFYSKNL
afr Elephant     WYQRKIAKMLFATPPSSTYE(E)ALSYFHRAEQVDPNFYSKNL
afr Rock_hyrax   WYQRKIAEMLFATPPSSTYE(E)ALSYFHRAEQVDPNFYSKNL
afr Tenrec       WYQRNIAKMLFATPPSSTYE(E)ALHYFHKAEQVDPNFYSKNL
mar Opossum      WVQRKIAKTLFATPPTSTFE(E)ALKYFQMAEKANPNFYSKNL
pro Platypus     WYQSRIAAMLFATPPSSTYE(E)ALNYFQKAEQADPNFYSKNL

NO 80
GN FAM83D
IP IPI00480103.3
PE EMPAEGKAERKPH
MP 385
EX Kim
CL primates
DE family with sequence similarity 83, member D
SQ
pri Human        ARLSSTPRKADLDPEMPAEG(K)AERKPHDCESSTVSEEDYFS
pri Chimpanzee   ARLSSTPRKADLDPEMPAEG(K)AERKPHDCESSTVSEEDYFS
pri Gorilla      ARLSSTPRKADLDPEMPAEG(K)AERKPHDCESSTVSEEDYFS
pri Orangutan    ARLSSTPRKADLGPEMPAEG(K)AERKPHDCESSTVSEEDYFS
pri Gibbon       ARLSSTPRKADLDPEMPAEG(K)AERKPHDCESSTVSEEDYFS
pri Rhesus       ARLSSTPRKADLDPEMPAEG(K)AERKPHDCESSTVSEEDYFN
pri Baboon       ARLSSTPRKADLDPEMPAEG(K)AERKPQDCESSTVSEEDYFN
pri Marmoset     ARLSSTPRKTDLDPEMPAEG(K)AEPKPHDCESSTVSEEDYSN
pri Tarsier      ARFSSTPRKTDLDSEMSTEG(K)AETKRHGSESSTISEEDNFN
pri Bushbaby     ARLSSTPKKSDLESEVPPED(K)TETKRHDSGSSTLSEEEHFN
pri Mouse_lemur  ARLSSTPKKSDLDSEAPREG(K)AGPKRRDSESSTMSEEDHFT
eua Treeshrew    ARLSSTPKKTDVDSQAPAEG(R)ATIRRRDSESSTISEEDYLH
eua Mouse        ARLSSTPRKSNLGPEEPPKD(R)AKPKRPDSEASTISDEDYFH
eua Rat          ARLSSTPKKTGLDPEVPPKD(R)DKTKRHDSETSTISDEDYFC
eua Guinea_pig   AKLSSTPKKTTLDLEVPVED(T)AEPKPRDSVSSTISEEDYFH
eua Squirrel     ARLSSSPRKTDLGLGAPLES(E)AETKRQDSKSSTVSEEGHFH
eua Rabbit       ARLSSTPKKAELEPEAPAEG(P)AESGRPASTSSTLGEDDDCR
eua Pika         ARLSSTPKRPGLEADGPEEG(G)AESRRPASPSSTLGEDDHLH
lau Dog          ARLSSTPKKAELGYEAPAEG(K)AEARRHDSESSTVSDED-LN
lau Cat          SRLSSTPKKAELGCEAPAEG(R)AEARHQNCESSTVGDEDDVI
lau Horse        ARLSSTPKKAELECEVPAEG(R)AEARRHDSESSTVSEEDCFN
lau Cow          ARLSSTPRKVELGGE---EG(R)AEAVCGASKTSTISEEDYFS
lau Megabat      ARLSSTPKKAELECGAPAKG(R)EEARRHDSESSTISEEDYFN
lau Microbat     ARLSSTPKKAELLCGAPAKG(R)AEARRHDSESSTISEEDYFN
lau Hedgehog     ARLSSTPKKADVEHEAPAEG(M)AEARRHDSESSTISDKDYLS
afr Elephant     ARLSSTPKKTDLEPEVPTEV(R)AETGRHDSASSTVSEEDCLN
afr Rock_hyrax   ARLSSTPKKTDLEPEVLTEG(K)GENGHRDSESSTIIEDYCLK
afr Tenrec       ARLSSSPKKPGLEPEVPAEG(R)AEAKRHDSESSTISEEDYLN
xen Sloth        ARLSSTPKKTDLESEVGTED(K)AETKLDDPETSTISEEDCVH
mar Wallaby      ARLSSTPNKADVSSEVPVEE(K)METNCCDAETSTISEDDCLS
pro Platypus     AKHTSTPNRVDPESKLVSEN(E)EEVKRRDSESSTISEEDFQS

NO 81
GN FANCA
IP IPI00006170.2
PE LPRELQKLQEGRQ
MP 1199
EX Kim, Wagner
CL catarrhines
DE Fanconi anemia, complementation group A
SQ
pri Human        PVLLCRWRRHCQSPLPRELQ(K)LQEGRQFASDFLSPEAASPA
pri Chimpanzee   PVLLCRWRRHCQSPLPRELQ(K)LQEGRQFASDFLSPEAASLA
pri Gorilla      PMLLCRWRRHCQSPLPWELQ(K)LQEGRQFASDFLSPEAASPA
pri Orangutan    PVLLCRWRRHCQSPLPRELQ(K)MQGGRQFASDFLSPEAASPA
pri Gibbon       PVLLCQWRRHCQSPLPRELQ(K)LQEGRQFASDFLSPDAASPA
pri Rhesus       PVLLCQWRRHCQSPLPWELQ(K)LQEGRQFASDFLSPDAASPA
pri Baboon       PVLLCQWRRHCQSPLPRELQ(K)LQEGRQFASDFLSPDAASPA
pri Marmoset     PVLLCQWRRRCQSPLPRELQ(R)LQEGRQFASDFLSPDATPQA
pri Mouse_lemur  PVLLCQWRRRCRSPLPRELQ(R)LQEAQRFARHFLSPEAASPS
eua Mouse        PVLCGRWMRCYQSPLPRELR(R)LQEAREFASNFAS-ASASPA
eua Rat          PVLCSQWKKCYQSTLPQELQ(R)LQEARKFASNFAS-DSAAPA
eua Kangaroo_rat PVLCSRWRKCVQDELPREFR(R)LKAARDFASNFFS-DSASPA
eua Guinea_pig   PVLCSRWRKFGQGRLPHELQ(R)LQEAHQFAHSFFS-DSTSAA
eua Rabbit       PVLCAQWRRCFQDCLPQELR(R)LQEARRFASDFLSSDSASSA
eua Pika         PVLCDQWRKSLQDPLPQELR(Q)LQDAQQFASNFLSPGPTSSP
lau Dog          PELHTRWRRCFQGPLPQELQ(R)LWEAQLFGKSCLSSDLASPP
lau Cat          PELRSRWRRCLQAPLPQELQ(R)LWEAQRFGRSCLLPDTASPA
lau Horse        PELHCRWRRCFQGPLPPELQ(R)LQEAQQFAQSFLSPDTAAPA
lau Cow          PELHCRWRRWSQSPLPAELR(K)LQEAHLFAESVSSPLTPSPA
lau Dolphin      PELHRRWRRCSQSPLPAELR(R)LQEARHFAGSVLSPLAAPPT
lau Megabat      RELRCRW-RCVPGPLPRELQ(R)LHEAQRFAQSFLSPDAASPA
lau Microbat     PELLCRW-RCFQSPLPRELQ(R)LRDAWQFASSLLSPDTVSPT
afr Elephant     PVLACQWRRHSQSSLPRALQ(Q)LAEARDLARSFLSLDTALPT
afr Rock_hyrax   PVLMCQWKQHSQSLLPGALR(Q)LAEIRDLANSFLSLETTLST
mar Opossum      PVVQCQWKRHFQTALPQELT(N)IATCREFARTFVSSEAVSSN
pro Platypus     PVLTCQWKRNSENPLPQKLQ(N)LVVGQQSAWIFLSSNMVSPI

NO 82
GN FANCA
IP IPI00006170.2
PE GRSLELKGQGNPV
MP 1387
EX Kim
CL humans
DE Fanconi anemia, complementation group A
SQ
pri Human        LFVAGDTSTVSPPAGRSLEL(K)GQGNPVELITKARLFLLQLI
pri Chimpanzee   LFVAGDTSTVSPPAGRSLEL(Q)GQGNPVELITKARLFLLQLI
pri Gorilla      LFVAGDTSTVSPPAGRSLEL(Q)GQGNPVELITKARLFLLQLI
pri Orangutan    LFVAGDTSTVSPPAGRRLEL(Q)GQDNPVELITKARLFLLQLI
pri Gibbon       LFVAGDTSTVSPPAGRSLEL(Q)GQGNPVELITKARLFLLQLI
pri Rhesus       LFVAGDTSTVSPPAGRSLEL(Q)GQGNPVELITKARLFLLQLI
pri Baboon       LFVAGDTSTVSPPASRSLEL(Q)GQGNPVELITKARLFLLQLI
pri Marmoset     LFVAGDTSTVSPPAGRSLEL(K)GQGDPVDLITKARLFLLQLI
pri Bushbaby     LFMAGDTSAVSTLVSRSPEL(Q)GQGNPVELITKARLFLLHLI
pri Mouse_lemur  LFVAGETSAVLLRASRNPEL(Q)GQGNPVELITEARLFLLQLI
eua Mouse        LFVDGETR----LQGHS---(E)SQGSPVQLITKARVFLLQLI
eua Rat          LFVDGETRIVLPQASRSIS-(E)LQDSPVQLITKARSFLLQLI
eua Rabbit       LFVDGETSAVVTPASGSREL(-)-QGSPVELVTKARDFLLQLI
eua Pika         LFVAGDNGAVGTLAHRSLEL(-)-QDHPVQVVTRARMFLLQLI
lau Dog          LFVTGETSAVSALAGRSQEL(Q)GQGDPVGLIIKARHFLLQSI
lau Cat          LFVAGETSALATVASRSRER(R)GQGDPVGLITEARRLLLRSI
lau Horse        LFVAGETSAVSTPANRSQEL(Q)SQGDPVGLIRKARLFLLQSI
lau Cow          LFVAGETGAIWTVAHD--EL(P)TQGDPVSLITNARLFLLQLI
lau Dolphin      LFVAGETGAVWTLAHS--DL(Q)AQGDPVSLITNARLFLLQSI
lau Megabat      LFVAGETSAASTLAARSQEL(Q)GQDDPVGLVTKARLFLLQSI
lau Microbat     LFVAGETAVLSTLASRTQEL(Q)GQGCPVGLITKARLFLLQSI
lau Hedgehog     LFVAGETGAVSVVVGK----(Q)NQDDPVGLMMKARLFLLNTI
lau Shrew        LFLGGETQPVTGPGRH----(Q)NQEEPVGLITKARLFLLRAI
afr Elephant     LFVAGEMSTLPTLVTQSQAY(Q)DQDDPLGLISKARLFLLRSI

NO 83
GN FANCD2
IP IPI00075081.1
PE EYFFENKNSDEIN
MP 165
EX Kim
CL simians
DE Fanconi anemia, complementation group D2
SQ
pri Human        LQPAIIKTLFEKLPEYFFEN(K)NSDEINIPRLIVSQLKWLDR
pri Chimpanzee   LQPAIIKTLFEKLPEYFFEN(K)NSDEINIPRLIVSQLKWLDR
pri Orangutan    LQPAIIKTLFEKLPEYFFEN(K)NSDEINIPRLIVSQLKWLDR
pri Gibbon       XXPAIIKTLFEKLPEYFFEN(K)NSDEINIPRLIVSQLKWLDR
pri Baboon       LQPAIIKTLFEKLPEYFFEN(K)NSDEINIPRLIVSQLKWLDR
pri Marmoset     LQPAIIKTLFEKLPEYFFDS(K)NSDEINIPRLIVSQLKWLDR
pri Tarsier      LQPAIINTLFEKLPEFFFKN(M)SGDGINMPRLIISQLKWLDR
eua Treeshrew    LQPAIIKTLFEKLPEFFFEN(M)NNDGIHMPRLIFSQLKWLDR
eua Mouse        LQPAIIKMLFEKVPQFLFES(E)NRDGINMARLIINQLKWLDR
eua Rat          LQPAIIKMLFEKVPQFLFES(E)SRDGISMPRLIISQLKWLDR
eua Guinea_pig   LQPAIIKTLFEKLPEFLFVK(V)NSDGISMPRLIISQLKWLDR
eua Squirrel     LQPAIIKILFEKLPEFLFKT(A)SSDGINMPRLIFSQLKWLDR
eua Rabbit       LQPAIIKTLFEKLPEFFFEN(V)NGDGISMPRLIISQLKWLDE
lau Dog          LQPAIIKTLFEKLPEFLFEN(V)NSDGLNIPRLIISQLKWLDR
lau Cat          LQPAIIKTLFEKLPEFLFEN(V)NSDGLNMPRLIISQLKWLDK
lau Horse        LQPAIIKTLFEKLPEFLFEK(V)NTDGISMPRLIISQLKWLDR
lau Cow          LQPAIIKVLFEKLPEFLFEK(V)NSDGISMPRLIINQLKWLDR
lau Dolphin      LQPAIINILFEKLPEFIFKN(V)NSDGLSMPRLIINQLKWLDR
lau Alpaca       LQPAIIKTLFEKLPEFLFEK(V)NSDGINMPRLIINQLKWLDR
lau Megabat      LQPAIIKTLFEKLPEFLFER(V)NSDGISMPRLIISQLKWLDR
lau Shrew        LQPAIVKTLLEQLPEFFFQS(R)SSDGISMPRLIISQLKWLDQ
afr Elephant     LQPAVIKTLFEKLPEFLFEN(R)NSDGISVPRLIISQLKWLDR
afr Rock_hyrax   LQPPVIKTLFEKLPEFLFEN(R)DSDGISMPRLMISQLKWLDR
xen Armadillo    XXPAIIKTLFEKLPEFLFEN(K)NSDGINMPRLIISQLKWLDK
xen Sloth        LQPAVIKILFEKLPEFLFEN(E)NSDGLNMPRLIISQLKWLDX
mar Opossum      LQPPIIRILFEKLPEFLYES(V)NNDGLNMPRLIINQLKWLDR
mar Wallaby      LQPAIIRILFEKLPEFLYDS(V)NNDGLYMPRLIINQLKWLDX
pro Platypus     LQPAVIKLLFEKFPQFMLER(S)GSDEMGIPQLIINQFKWIDR

NO 84
GN FANCE
IP IPI00030252.1
PE QPVMAVKTGEDGS
MP 260
EX Kim
CL simians
DE Fanconi anemia, complementation group E
SQ
pri Human        ESLADGGSASPIKDQPVMAV(K)TGEDGSNLDDAKGLAESLEL
pri Chimpanzee   EFLADGGSASPIKDQPVMGV(K)TGEDGSNLDDAKGLAESLEL
pri Gorilla      ESLADGGSASPIKDQPVMEV(K)TGEDGSNLDDAKGLAESLEL
pri Orangutan    ESLAGGGSTSPIKDQPVMGA(K)TGEDGSSLEDAKGLAESLEL
pri Gibbon       ESLADGGSATPIKDQPVMGA(K)TGEDGSSLEDAKGRAESLEL
pri Rhesus       ESLADGGGASPIKDQPVMGA(K)TGQDGSSLEDAKGLAESLEL
pri Baboon       ESLADGGGASPIKDQPVMGA(K)TGQDGSSLEDAKGLAESLEL
pri Marmoset     ESLADGEGTSPIKDQPVMGA(K)TGKDGSSLEDAKGLPESLEL
pri Bushbaby     ESLVGEGAASPIKNHPVLEA(E)ISKASPSLEAAKSVAESFEL
pri Mouse_lemur  ESLAGGEGASPIKSQPVLGA(E)TSKASASLEDAKGPAEGLVL
eua Treeshrew    ESLADGGGTLHVENQLVTAA(E)TSETGQSLEGAEDVVGTLEL
eua Mouse        GSPPDAGGVLPDTD-----A(Q)APETGPGVEGPKGPAESVEL
eua Rat          ESESDAGGVLPDTE-----V(E)APEARPGVAEAESPAKSVEL
eua Guinea_pig   EALEDRG-ASPRRSQPGEGA(A)PRGTLQSPREVQGPAATVEL
eua Rabbit       ESLADGGVVALVESHPVVGA(E)SGEAGRSTEDARGPALSLEL
eua Pika         GSLADEGATVSVEDDHVVGV(E)SNEAVRRLEAAQGPAESLEL
lau Dog          EPLADGGDALPIKNQSV-RA(K)PSEAGQSLEAAKDLPECLEL
lau Cat          ESLAGGGGTLPIKNQPV-QA(E)PSVAGQSLEDARGLAENLEL
lau Horse        EFLAAGEGTSPIEDQPVAGA(E)PNEAGQSLEDAKGLAESLEL
lau Cow          ESLADGGGASSITNQAVMGP(E)PSEAGQSLKDAKGLPESLEL
lau Dolphin      ESLANGGGASSIKNQPVLGA(K)PSEAGQSLEDAKGLPESLEL
lau Megabat      ESLADEGGTSLIKNQLLVGA(E)LSDAGPSLEDANGSAESLEL
lau Microbat     ETLADGGDTSPTKNQPVVGA(E)CMEAAQGLEDTKGPAERVEL
lau Hedgehog     DPLADEGSASPIKKQPIVGT(E)HSEPAQSPVDAKDLAERLEL
afr Elephant     ESRADGGAASPIKNQTVLRA(E)INEAGQNLEDAKGLVENVEL
afr Rock_hyrax   ESPAEGGSASLIKSKTVVRA(E)TTAAGQNLGHAKGLVENVEL
afr Tenrec       ESAACGGDASPVLSQPAVGA(E)PREAPPKPEGSKDLVETLEL

NO 85
GN FASN
IP IPI00026781.2
PE TPEAVQKLLEQGL
MP 436
EX Kim, Wagner
CL apes
DE fatty acid synthase
SQ
pri Human        PHATLPRLLRASGRTPEAVQ(K)LLEQGLRHSQDLAFLSMLND
pri Chimpanzee   PHATLPRLLRASGRTPEAVQ(K)LLEQGLRHSQDLAFLSMLND
pri Orangutan    PHATLPRLLRASGRTPEAVR(K)LLEQGLQHSQDLAFLSMLNG
pri Gibbon       PHATLPRLLRASGRTPEAVQ(K)LLEQGLQHSQDLAFLSMLND
pri Rhesus       PHASLPRLLRASGRTPEAVQ(E)LLEQGLQHSQELAFLSMLND
pri Baboon       PHASLPRLLRASGRTPEAVQ(E)LLEQGLQHSQELAFLSMLND
pri Marmoset     PHATLPHLLRASGRTAEAVQ(E)LLEQGLQHSQDLAFLSMLNN
pri Bushbaby     PSAASPRLLQASGRTPEAVQ(G)LLELGQHHSQDLAFVCMLND
eua Mouse        AHAALPHLLHASGRTLEAVQ(D)LLEQGRQHSQDLAFVSMLND
eua Kangaroo_rat PHAALPRLLQASGRTPEAVQ(S)LLDQGRRHSQDLAFLSMLND
eua Guinea_pig   PHAALPRLLQASGRTLEAVQ(S)LLEQGRQHSQDLAFVSMLND
eua Squirrel     PHAPLPRLLQASGRTAEAVQ(S)LLDQGCQHGQDMAFVSMLNS
eua Pika         PHAALPRLLRASGRTPEAVQ(N)LLEQGQRHSQDLTFVSMLND
lau Dog          PHITLPRLLRASGRTPEAVR(C)LLEKGQQHSRDVAFVTMLND
lau Cat          RHASLPRLLRASGRTPEAVH(C)LLDQGHRHSQDLAFVSMLND
lau Horse        PHVALPCLLRASGRTSEAVL(G)LLEQGRQHSQDLAFVSMLND
lau Cow          PHAALPRLLRASGRTLEGVQ(G)LLELGLQHSQNLAFVSMLND
lau Dolphin      PHSALPRLLRASGRTLEAVQ(G)LLEQGLRHSHDLAFVSMLND
lau Megabat      PHAVLPRLLLASGRTPEAVQ(A)LLEQGHRHSQNLAFVSMLND
lau Hedgehog     PHASLPRLLVSSGRTPKAVQ(G)LLEQGLQHSQDLAFLSMLNS
lau Shrew        PHASLPRLLLASGRTAEAVQ(S)LLAQGQQHAQDLALVSMLNS
afr Elephant     P-PALPRLLLASGRTHEAVQ(S)LLEQGCRHGQDLAFLSMLND
afr Rock_hyrax   P-PALPRLLRASGRTPEAVQ(G)LLEQGRQHSQDLAFLGLLND
mar Opossum      S-PPLPHLLQVSGRTEEAVE(T)LLEQGQLQAQDQTFVSMLND
mar Wallaby      PH--LPHLLQVSGRTEEAVE(T)LLEQGQLQAQDETFVSMLND

NO 86
GN FASN
IP IPI00026781.2
PE FVEQLRKEGVFAK
MP 667
EX Kim
CL catarrhines
DE fatty acid synthase
SQ
pri Human        DTVTISGPQAPVFEFVEQLR(K)EGVFAKEVRTGGMAFHSYFM
pri Chimpanzee   DTVTISGPQAPVFEFVEQLR(K)EGVFAKEVRTGGMAFHSYFM
pri Gorilla      DTVTISGPQAPVFEFVEQLR(K)EGVFAKEVRTGGMAFHSYFM
pri Orangutan    DTVTISGPQAPVFEFVEQLR(K)EGVFAKEVRTGGLAFHSYFM
pri Gibbon       DTVTISGPQAPVFEFVEQLR(K)EGVFAKEVRTGGMAFHSYFM
pri Rhesus       DTVTISGPQASVFEFMEQLR(K)EGVFAKEVRTGGMAFHSYFM
pri Baboon       DTVTISGPQASVLEFMEQLR(K)EGVFAKEVRTGGMAFHSYFM
pri Marmoset     DTVTISGPQGPVHEFVEQLK(Q)EGVFAKEVRTGGMAFHSYFM
pri Mouse_lemur  DTVTISGPQAAVSAFVEQLK(Q)EGVFAKEVRTGGMAFHSYFM
eua Treeshrew    DTVTISGPQASVCEFVDQLK(R)EGVFAKEVRTGGMAFHSYFM
eua Mouse        DTVTISGPQAAVNEFVEQLK(Q)EGVFAKEVRTGGLAFHSYFM
eua Kangaroo_rat DTVTVSGPQASVSKFVEQLK(Q)DGVFAKEVRTGGIAFHSYYM
eua Guinea_pig   DTVTISGPQAAVSKFVEQLK(Q)EGVFAKEVQTGGIAFHSYFM
eua Pika         DTVTISGPQAPMAEFVEQLR(R)EGVFAKEVRTGGLAFHSYFM
lau Dog          DTVTISGPQAEVAAFLEELR(Q)EGVFAKEVRTGGMAFHSYFM
lau Cat          DTVTISGPQAEVAAFVAELK(R)EGVFAKEVRTGGMAFHSYFM
lau Horse        DTVTISGPQAAVSEFVEQLK(Q)EGVFAKEVRTGGLAFHSYFM
lau Cow          DTVTISGPQASMLEFVQQLK(Q)EGVFAKEVRTGGMAFHSYFM
lau Megabat      DTVTVSGPQAAMSKFVAQLK(Q)EGVFAKEVRTGGLAFHSYFM
lau Microbat     DTVTISGPQASVSEFVEQLR(Q)EGVFAKEVRTGGLAFHSYYM
afr Elephant     DTVTISGPQAAVVEFVEQLK(Q)EGVFAKEVRTGGLAFHSYFM
afr Rock_hyrax   DTVTISGPEAAVAEFVQQLK(Q)EGVFAKEVRTGGMAFHSYFM
mar Opossum      DTVTISGPQALMSKFIEELK(A)EGVFAKEVRTGGVAFHSYFM
mar Wallaby      DTVTISGPQXXXXKFIEELK(A)EGVFAKEVRTGGVAFHSYFM
pro Platypus     DTVTISGPQAAVATFIEELK(A)EGVFAKEVRTGGVAFHSYYM

NO 87
GN FAT1
IP IPI00031411.3
PE FTASSYKGRVYES
MP 1568
EX Kim
CL simians
DE FAT tumor suppressor homolog 1 (Drosophila)
SQ
pri Human        IVVNVSDTNDHAPWFTASSY(K)GRVYESAAVGSVVLQVTALD
pri Chimpanzee   IVVNVSDTNDHAPWFTASSY(K)GRVYESAAVGSVVLQVTALD
pri Gorilla      IVVNVSDTNDHAPWFTASSY(K)GRVYESAAVGSVVLQVTALD
pri Orangutan    IVVNVSDTNDHAPWFTASSY(K)GRVYESAAVGSVVLQVTALD
pri Gibbon       IVVNVSDTNDHAPWFTASSY(K)GRVYESAAVGSVVLQVTALD
pri Rhesus       IVVNVSDTNDHAPWFTASSY(K)GRVYESAAVGSVVLQVTALD
pri Baboon       IVVNVSDTNDHAPWFTAASY(K)GRVYESAAVGSVVLQVTALD
pri Marmoset     IVVDVSDTNDHAPWFTSSSY(K)GRVYESAAVGSVVLQVTALD
pri Tarsier      IMINVGDTNDHAPWFTSSSY(E)GRVYESAAIGSVALQVTALD
pri Bushbaby     XXXXXXXXXXXXXXXTSSSY(E)GRVYESAAVGSVVLQITALD
pri Mouse_lemur  IVVNVSDTNDHAPWFTSSSY(E)GRVYESAAVGSVVLQVTALD
eua Treeshrew    IVVNVSDTNDHAPWFTSSSY(E)GRVYESSAVGSVVLQVTALD
eua Mouse        IVVNVSDKNDHAPWFTSPSY(D)GRVYESAAVGSVVLQVTALD
eua Rat          IIVNVSDMNDHSPWFTSSSY(E)GRVYESAAVGSVVLQVTALD
eua Kangaroo_rat IIVNVSDTNDHAPWFTSSSY(E)GRVYESAAVGSVVLQVTALD
eua Guinea_pig   IIVNVSDTNDHAPWFTSSSY(E)GRVYESAAVGSVVLQVTALD
eua Squirrel     IVINVSDTNDHAPWFTSSSY(E)GRVYESAAVGSVVLQVTALD
eua Rabbit       IIVNVSDTNDHAPWFTSSSY(E)GRVYESAAVGSVVLEVTALD
eua Pika         IIVNVSDTNDHAPWFTSSSY(E)GRVYESAAVGSVVLEVTALD
lau Dog          IVVNVSDTNDHAPWFTSSSY(E)GRVYESAAVGSVVLQVTALD
lau Cat          IIVNVSDTNDHAPWFTSSSY(E)GRVYESAAVGSVVLQVTALD
lau Horse        IVVNVSDTNDHAPWFTSSSY(E)GRVYESAAVGSVVLQVTALD
lau Cow          IVVNVSDTNDHAPWFTSSSY(E)GRVYESAAVGSVVLQVTALD
lau Dolphin      IVVNVSDTNDHAPWFTSSSY(K)GRVYESAAVGSVVLQVTALD
lau Alpaca       IVVNVSDTNDHAPWFTSSSY(E)GRVYESAAVGSVVLQVTALD
lau Megabat      IVVNVSDTNDHAPWFTSSSY(E)GRVYESAAVGSVVLQVTALD
lau Microbat     IVVNVSDTNDHAPWFTSSSY(E)GRVYESAAVGSVVLQVTALD
lau Hedgehog     IAVNVSDTNDHAPWFTSSTY(E)GRAYESAAIGSVVLQVTALD
lau Shrew        IVVDVSDTNDHAPWFTSSSY(E)GRVYESAAVGSVVLQVTALD
afr Elephant     IVVNVSDTNDHAPWFTSSSY(E)GRVYESAAIGSVVLQVTALD
afr Tenrec       IVVNISDTNDHAPWFTSSSY(E)GRVYESAAVGSVVLQVTALD
xen Armadillo    VVVNVSDSNDHAPWFTSPAH(E)GRVYESAAVGSVVLQVTALD
xen Sloth        IVVNVSDTNDHAPWFTSPSY(E)GRVYESAAVGSVVLQVTALD
mar Opossum      VIVNVSDTNDHAPWFTSSSY(Q)GRVYESAAVGSVVLQVTALD
mar Wallaby      VIVNVSDMNDHAPWFTSSSY(Q)GRVYESAAVGSVVLQVTALD

NO 88
GN FBXO40
IP IPI00008203.7
PE GEGAPKKKEPQEN
MP 266
EX Kim
CL simians
DE F-box protein 40
SQ
pri Human        EKEQISSGHNMVE-GEGAPK(K)KEPQENQKQQDVRTAMETTG
pri Chimpanzee   EKEQISSGHNMVE-GEGTPK(K)KEPQENQKQQDVRTAMETTG
pri Gorilla      KKEQISSGHNMVE-GEGAPK(K)KEPQENQKQQDICTAMETTG
pri Orangutan    EKEQISSGNNMVE-GEGAPQ(K)KEPQENQKQQDVRTAMETTG
pri Gibbon       EKEQISSGNNMVE-GEGAPQ(K)KEPQENQKQQDVHTAVETTG
pri Rhesus       EKEQISSGNNMVE-GEGAPE(K)KEPQENQKQQNVHTAVETTG
pri Baboon       EKEQISSGNNMVE-GEGAPK(K)KEPQENQKQQDVHTAVETTG
pri Marmoset     EKEQISSGNNTVE-GEGVPK(K)KEPQENEKQQDIHKAVETAG
eua Treeshrew    GKDQIPSGNNMVK-GEGAAK(D)EESQENQRQRDFPATVETTG
eua Mouse        GKEQCSSNVRIGD-AEGSAE(R)RGPQESQKSQELPATMEMTG
eua Rat          GKEQRSGDGSTGD-AECSTE(R)RGPQESPKPQEPPTTIEMTG
eua Guinea_pig   GKEQSSSGDNMAD-TEGATK(G)KEPQEYQMQQNLSSVIEKAG
eua Squirrel     GTEQISDGNNVVE-GEAAAN(G)EEPQQDQKQRDFSAEMEKTG
eua Rabbit       GREQDSSDNNKAD-GEGVAK(G)QEPQANQKQRDFLAAVEKTG
lau Dog          EKEQSPSHSNGVGAGENTLR(E)TEARGEQRQRDVPAALETTG
lau Cat          GKEQVSSDNNMVR--EDDLQ(E)KETQEKQKQQDFNAVVEKTG
lau Horse        GKEQISSGNNMVK--EDTAN(E)KEPQENQKQQDSRGAMETTG
lau Cow          EKEQISNSNRMVE--EGAAK(G)KEPQEDQKQQDFHAAIEKTG
lau Dolphin      EKEQISSSNNMVE--KSAAK(G)KETQEDQKQQEFHEAIEKSG
lau Alpaca       EKEQVSSGSNSGE--EGAAK(G)KELQGNQKQQDFNAAVEKTG
lau Megabat      EKKQISNSNNMVG--EGTDE(G)KEPQENQKQQDFHAAEETTG
lau Microbat     EKEQISITNNMIG--EASAE(E)IESQENQRQQDYHAAMETTG
lau Hedgehog     GEEQISSSNNMID--AGTAK(G)TQPQENQKQRDFHAAVETTG
lau Shrew        GKEQIFSNRNMVA--NGAAS(G)KDSEENQKQEDFHAAIETTG
afr Elephant     GKEQVSRDNNAGE---GVAK(G)KEPQETQKKEDFHTAVETVG
afr Rock_hyrax   GKEEDSRNNNAVE-EEGTAK(E)KEPQENQKQQDLHTTVETTG
xen Armadillo    GREQSSSSQNVVE-GEGAAK(G)KAAQENQQPRDFHTTVETTG
xen Sloth        GREQISSINNVVE-GEGDAK(G)KEPQENQQQQDFHTIMETTG
mar Opossum      GRVQNSSQGNASE--KGNDM(E)KEAQANQEEKDPYRNMETTG
mar Wallaby      GKEQTASQGRASE--KGASV(E)KEGRADQKE-NPYRNMETTG

NO 89
GN FDPS
IP IPI00914566.2
PE EQYQILKENYGQK
MP 353
EX Kim, Wagner
CL simians
DE farnesyl diphosphate synthase
SQ
pri Human        CSWLVVQCLQRATPEQYQIL(K)ENYGQKEAEKVARVKALYEE
pri Chimpanzee   CSWLVVQCLQRATPEQYQIL(K)ENYGQKEAEKVARVKALYEE
pri Gorilla      CSWLVVQCLQRATPEQYQIL(K)ENYGQKEAEKVARVKALYEE
pri Orangutan    CSWLVVQCLQRATPEQYQIL(K)ENYGQKEAEKVARVKALYEE
pri Gibbon       CSWLVVQCLQRATPEQYQIL(K)ENYGQKEAEKVARVKALYEE
pri Rhesus       CSWLVVQCLQRATPEQYQIL(K)ENYGQKEAEKVARVKALYEE
pri Baboon       CSWLVVQCLQRATPEQYQIL(K)ENYGQKEAEKVARVKALYEE
pri Marmoset     CSWLVVQCLQRSTPEQRQIL(K)ENYGQKEAEKVARVKALYEE
pri Bushbaby     CSWLVVQCLQRATPEQRQIL(Q)XXXXXXXXXXXXXXXXXXXX
eua Treeshrew    CSWLVVQCLQRATLEQRQIL(Q)XXXXXXXXXXXXXXXXXXXX
eua Mouse        CSWLVVQCLLRASPQQRQIL(E)ENYGQKDPEKVARVKALYEA
eua Rat          CSWLVVQCLLRATPQQRQIL(E)ENYGQKDPEKVARVKALYEE
eua Kangaroo_rat CSWLVVQCLQRGTPEQRQIL(Q)ENYGQKDAKKVAQVKALYEQ
eua Guinea_pig   CSWLVVQCLQRATLEQRQIL(E)ENYGQKDAEKVAKVKELYKE
eua Pika         CSWLVVQCLQRATPEQRQIL(Q)ENYGQKEAEKVARVKALYEE
lau Dog          CSWLVVQCLQQASPEQRKVL(Q)ENYGQKEAEKVARVKALYEE
lau Horse        CSWLVVQCLQRASPEQRQIL(Q)ENYGQKEAEKVARVKALYEE
lau Cow          CSWLVVQCLQRASPEQRQIL(Q)ENYGQKEAEKVARVKALYEE
lau Dolphin      CSWLVVQCLQRASPEQRQIL(Q)ENYGQKEAEKVARVKALYEE
lau Alpaca       CSWLVVQCLQRASPEQRQIL(Q)ENZGQKEAEKVAQVKSLYEE
lau Hedgehog     CSWLVVQCLQRASPEQRQII(Q)ENYGQKEEEKVARVKMLYEE
lau Shrew        CSWLVVQCLQRASPEQRRIL(Q)ENYGQKDAKKVARVKALYEE
afr Elephant     CSWLVVQCLQRASPEQRQIL(Q)ENYGQKEPEKVARVKALYEE
afr Rock_hyrax   CSWLVVQNLQRASPEQRQIL(Q)ENYGQKDAEKVARVKALYEE

NO 90
GN FKBP4
IP IPI00219005.3
PE EGYYKDKLFDQRE
MP 181
EX Kim, Wagner
CL African great apes
DE FK506 binding protein 4, 59kDa
SQ
pri Human        YAKPNEGAIVEVALEGYYKD(K)LFDQRELRFEIGEGENLDLP
pri Chimpanzee   YAKPNEGAIVEVALEGYYKD(Q)LFDQRELRFEIGEGENLDLP
pri Gorilla      YAKPNEGAIVEVALEGYYKD(K)LFDQRELRFEIGEGENLDLP
pri Orangutan    YAKPNEGAIVEVALEGYYKD(Q)LFDQRELRFEIGEGENLDLP
pri Gibbon       YAKPNEGAIVEVALEGYYKD(Q)LFDQRELRFEIGEGENLDLP
pri Rhesus       YAKPNEGAIVEVALEGYYKD(Q)LFDQRELRFEIGEGENLDLP
pri Baboon       YAKPNEGAIVEVALEGYYKD(Q)LFDQRELRFEIGEGENLDLP
pri Marmoset     YAKPNEGAIVEVALEGYYKD(Q)LFDQRELRFEIGEGENLDLP
pri Mouse_lemur  YARPNEGAVVEVALEGYYKD(Q)LFDQRELHFEVGEGESLDLP
eua Mouse        YARPNDGAMVEVALEGYHKD(R)LFDQRELCFEVGEGESLDLP
eua Rat          YARPNDGAMVEVALEGYYND(R)LFDQRELCFEVGEGESLDLP
eua Guinea_pig   YSRPNDGAIVEVSLEGYYKD(Q)LFDQRELRFEVGAAESLDIP
eua Rabbit       YARPNDGAIVEVALEGYYKD(R)LFDQRELRFEVGEGESLDLP
eua Pika         YARPNDGAIVEVALEGYYKD(R)LFDQRELRFEVGEGDSLDLP
lau Dog          YARPNEGAIVDVTLEGYYKD(Q)MFDQRELRFEVGEGESLDLP
lau Cat          YARPNEGAIVEVTLEGYYKD(Q)MFDQRELRFEVGEGESLDLP
lau Horse        YARPNDGALVEVALEGYYKD(Q)LFDRREVHFEVGEGENLDLP
lau Cow          YAKPNEGALVEVALEGYFKD(Q)VFDRRELRFEVGEGESMDLP
lau Dolphin      HAKPNDGALVEVALEGYYKD(Q)IFDRRELRFEVGEGESVDLP
lau Megabat      FAKPNDGALVEVALEGYYQD(Q)VFDQRELHFEIGEGEILHLP
lau Microbat     YAKPNEGAIVEVALEGYYQD(Q)IFDQRELHFEIGEGESLDLP
lau Hedgehog     YAKPNEGAIVEIALEGYFKD(Q)VFDQRELRFEVGEGESLDLP
afr Elephant     YARPNDGAIVDVILKGYYND(R)VFDERELQFEIGEGENLDLP
afr Tenrec       YARPNEGAIVEVVLEGYYKD(R)MFDQRELQFEIGDGENLDLP
mar Opossum      HSKPNDGAIVEVALEGHHKG(R)IFDQRELSFEIGDGENYDVP
mar Wallaby      YAKPNEGARVEVVLEGRHKG(R)VFDQRELSFEIGEGENYDLP
pro Platypus     YAKPNEGATVDITLEGRHGE(R)VFDRRELCFEIGEGESFDVP

NO 91
GN FTSJ1
IP IPI00004308
PE KLLQLDKEFQLFQ
MP 34
EX Wagner
CL catarrhines
DE FtsJ homolog 1 (E. coli)
SQ
pri Human        RLAKENGWRARSAFKLLQLD(K)EFQLFQGVTRAVDLCAAPGS
pri Chimpanzee   RLAKENGWRARSAFKLLQLD(K)EFQLFQXXXXXXXXXXXXXX
pri Orangutan    RLAKENGWRARSAFKLLQLD(K)EFQLFQGVTRAVDLCAAPGS
pri Gibbon       RLAKENGWRARSAFKLLQLD(K)EFQLFQGVTRAVDLCAAPGS
pri Rhesus       RLAKENGWRARSAFKLLQLD(K)EFQLFQGVTRAVDLCAAPGS
pri Baboon       RLAKENGWRARSAFKLLQLD(K)EFQLFQGVTRAVDLCAAPGS
pri Mouse_lemur  RLAKENGWRARSAFKLLQLD(E)EFQLFKXVTRAVDLCAAPGS
eua Mouse        RLAKENGWRARSAFKLLQLD(E)EFQLFKGVKRAVDLCAAPGS
eua Rat          RLAKEKGWRARSAFKLLQLD(E)EFQLFKGVKRAVDLCAAPGS
eua Kangaroo_rat RLAKENGWRARSAFKLLQLD(E)EFQLFQGVTRAVDLCAAPGS
eua Guinea_pig   RLAKENGWRARSAFKLLQLD(E)EFHLFQGVTRAVDLCAAPGS
eua Rabbit       RLAKENGWRARSAFKLLQLD(E)EFQLFKGVTRAVDLCAAPGS
lau Dog          RLAKENGWRARSAFKLLQLD(E)EFHLFQGVTRAVDLCAAPGS
lau Horse        RLAKENGWRARSAFKLLQLD(E)EFQLFQGVTRAVDLCAAPGS
lau Cow          RLAKENGWRARSAFKLLQLD(E)EFQLFQGVTRAVDLCAAPGS
lau Megabat      RLAKENGWRARSAFKLLQLD(E)EFQLFQGVTRAVDLCAAPGS
lau Microbat     RLAKENGWRARSAFKLLQLN(E)EFQLFQGVTRAVDLCAAPGS
lau Hedgehog     RLAKENGWRARSAFKLLQLD(E)EFQLFQGVKRAVDLCAAPGS
lau Shrew        RLAKENGWRARSAFKLLQLD(E)EFQLFQGVTRAVDLCAAPGS
afr Elephant     RLAKENGWRARSAFKLLQLD(E)EFQLFQGVMRAVDLCAAPGS
afr Tenrec       RLVQENGWHAHSAFKLLQLN(E)KFQLFEGVVQLVDLCAAPGS
pro Platypus     RLAKEEGWRARSAFKLLQLD(E)EFQLFQGVHRAVDLCAAPGS

NO 92
GN GADD45GIP1
IP IPI00552587
PE LQDLEKKERKRLK
MP 185
EX Wagner
CL simians
DE growth arrest and DNA-damage-inducible, gamma interacting protein 1
SQ
pri Human        GYQVDPRSARFQELLQDLEK(K)ERKRLKEEKQKRKKEARAAA
pri Chimpanzee   GYQVDPRSARFQELLQDLEK(K)ERKRLKEEKQKRKKEARAAA
pri Gorilla      GYQVDPRSARFQELLQDLEK(K)ERKRLKEEKQKRKKEARAAA
pri Orangutan    GYQVDPRSARFQELLQDLEK(K)ERKRLKEEKQKRKKEARAAA
pri Gibbon       GYQVDPRSARFQELLQDLEK(K)ERKRLKEEKKKQKKEARAAA
pri Baboon       GYQVDPKSARFQELLQDLEK(K)ERKRLKEEKQRQKQEARAAA
pri Marmoset     GYQVDPRSTRFQELLQDLEK(K)ERKRLKEEKQRQKKEARAAA
pri Tarsier      GYHVTPRSTRFQELLQDLEK(Q)QRKRLKEEKQRQKKEARAAA
pri Mouse_lemur  GYHVDTRSARFQELLQDLEK(Q)QRKRLKEEKQRKKKEARAAA
eua Treeshrew    GYHVDPRSARFQELLQDLEK(Q)QRKRLKEEKQQKKKEARAAA
eua Mouse        GYHVDPRSARFQELLQDLDK(Q)QRKRLKEERQRQKKEARIAA
eua Rat          GYHVDPRSARFQELLQDLDK(Q)QRKRLKEERQRQKKEARIAA
eua Guinea_pig   GYHVDPRSTRFQELLQDLEK(Q)QRKRLKEEKQRQKKEARAAA
eua Squirrel     VYHVNPRSTRFQELLQDLEN(Q)LRKRLWEERQRQIREARAAA
lau Dog          GYHVDPRSARFQELLQDLEK(Q)QRKRLKEEKQRQKKEARAAA
lau Cat          GYHVDPRSARFQELLQDLEK(Q)QR-RLKEEKQRQKKEARAAA
lau Horse        GYHVDPRSARFQELLQDMEK(Q)QRKRLKEERQRQKKEARAAA
lau Cow          GYHVDPRSARFQELLQDLEK(Q)HRKRLKEEKQRKKKEARAAA
lau Dolphin      GYHVDPRSARFQELLQDLEK(Q)HRKRLKEEKQRKKKEARAAA
lau Megabat      GYHVDPRSARFQELLQDLEK(Q)QRKRLKEEKQRQKKEAQAAT
lau Microbat     GYHVDPRSTRFQELLQDLEK(Q)QRKRLKEEKQRQKKEARTAA
lau Shrew        GYHVDPRSTRFQELLQDMEK(Q)QRKRLKEEKQRQKKEARMAA
afr Rock_hyrax   GYDVDPRSTRFQELLQDMEK(Q)QRKRLKEEKQRQKKEARAAA
mar Opossum      GYDVDPRSSRFQELLQELEK(Q)DRKRLKQQKKQQKEAARMAA
mar Wallaby      GYDVDPRSSRFQELLQELEK(Q)DRKRLKQQKKQQKEAARMAA
pro Platypus     GYNVDPRSTKFQELVRDLEK(K)QRKQLKEQKQQQKLEARAAA

NO 93
GN GALE
IP IPI00553131.2
PE QLLEIMKAHGVKN
MP 120
EX Kim
CL catarrhines
DE UDP-galactose-4-epimerase
SQ
pri Human        KPLDYYRVNLTGTIQLLEIM(K)AHGVKNLVFSSSATVYGNPQ
pri Chimpanzee   KPLDYYRVNLTGTIQLLEIM(K)AHGVKNLVFSSSATVYGNPQ
pri Gorilla      KPLDYYRVNLTGTIQLLEIM(K)AHGVKNLVFSSSATVYGNPQ
pri Orangutan    KPLDYYRVNLTGTIQLLEIM(K)AHGVKNLVFSSSATVYGNPQ
pri Gibbon       KPLDYYRVNLTGTIQLLEIM(K)SHGVKNLVFSSSATVYGNPQ
pri Rhesus       KPLDYYRVNLTGTIQLLEIM(K)AHGVKNLVFSSSATVYGNPQ
pri Baboon       KPLDYYRVNLTGTIQLLEIM(K)AHGVKNLVFSSSATVYGNPQ
pri Marmoset     KPLDYYRVNLTGTIQLLEIM(R)AHGVKNLVFSSSATVYGNPQ
pri Mouse_lemur  KPLDYYRVNLTGTIQLLETM(R)ACGVKNLVFSSSATVYGNPQ
eua Mouse        KPLDYYRVNLTGTIQLLEIM(R)AHGVKNLVFSSSATVYGNPQ
eua Rat          KPLDYYRVNLTGTIQLLEIM(R)AHGVKSLVFSSSATVYGNPQ
eua Kangaroo_rat KPLDYYRVNLTGTIQLLEIM(R)AHEVKNLVFSSSATVYGNPQ
eua Guinea_pig   KPLDYYRVNLTGTIQLLEIM(R)AHGVKNLVFSSSATVYGNPQ
eua Squirrel     KPLDYYRVNLTGTIQLLEIM(R)AHGVKNLVFSSSATVYGNPQ
eua Rabbit       KPLDYYRVNLTGTIQLLEIM(R)THGVKNLVFSSSATVYGNPQ
lau Dog          KPLDYYRVNLTGSIQLLEIM(R)AHGVKNLVFSSSATVYGNPQ
lau Horse        KPLDYYRVNLTGTIQLLETM(R)AHGVKNLVFSSSATVYGNPQ
lau Cow          KPLDYYRVNLTGTIQLLEIM(R)AHGVKNLVFSSSATVYGNPQ
lau Dolphin      KPLDYYRVNLTGTIQLLEIM(R)AHGVKNLVFSSSATVYGNPQ
lau Megabat      KPLDYYRVNLTGTIQLLEIM(R)AHGVKNLVFSSSATVYGNPQ
lau Microbat     KPLDYYRVNLTGTIQLLEIM(R)AHGVKNLVFSSSATVYGNPQ
lau Hedgehog     KPLDYYKVNLTGTIQLLEIM(S)THGVKNLVFSSSATVYGNPQ
lau Shrew        KPLDYYRVNLTGTIQLLEIM(R)AHGVKNLVFSSSATVYGNPQ
afr Elephant     KPLDYYRVNLTGTIQLLEIM(R)AHGVKNLVFSSSATVYGNPQ
afr Rock_hyrax   KPLDYYRVNLTGTIQLLEIM(R)AHGVKNLVFSSSATVYGNPQ
xen Armadillo    KPLDYYRVNLTGTIQLLEIM(R)AHGVKNLVFSSSATVYGNPQ
mar Opossum      KPLDYYKVNLTGTIQLLEAM(N)AHGVKNLVFSSSATVYGNPH
mar Wallaby      KPLDYYRVNLTGTIQLLETM(N)THGVKNLVFSSSATVYGNPH

NO 94
GN GALK1
IP IPI00019383.2
PE LVGSPRKDGLVSL
MP 99
EX Kim, Wagner
CL catarrhines
DE galactokinase 1
SQ
pri Human        QGLVLPMALELMTVLVGSPR(K)DGLVSLLTTSEGADEPQRLQ
pri Chimpanzee   QGLVLPMALELMTVLVGSPR(K)DGLVSLLTTSEGADEPQRLQ
pri Gorilla      QGLVLPMALELMTVLVGSPR(K)DGLVSLLTTSEGADEPQRLQ
pri Orangutan    QGLVLPMALELMTVLVGSPR(K)DGLVSLLTTSEGADEPQRLQ
pri Gibbon       QGLVLPMALELVTVLVGSPR(K)DGLVSLLTTSEGADEPQRLQ
pri Rhesus       QGLVLPMALELVTVVVGSPR(K)DGLVSLLTTSEGADEPQRLQ
pri Baboon       QGLVLPMALELVTVVVGSPR(K)DGLVSLLTTSEGADEPQRLQ
pri Marmoset     XXXXXXXALELVTVLVGSPR(E)DGLVSLLTTSADADEPQRLQ
pri Tarsier      XXXXXXXALELVTVLVGSPR(A)DGLVSLLTTSEDADEPHRLQ
pri Bushbaby     XXXXXXXALELVTVLVGSPR(A)DGLVSLLTTSEDADEPQRLQ
pri Mouse_lemur  XXXXXXXALELVTVLVGSPR(A)DGLVSLLTTSEDADEPQRLQ
eua Mouse        QGLVLPMALELVTVMVGSPR(T)DGLVSLLTTSKDADEPQRLQ
eua Rat          QGLVLPMALELVTVMVGSPR(T)DGLVSLLTTSKDADEPQRLQ
eua Kangaroo_rat QGLVLPMALELVTLIVGSPR(T)DGLVSLLTTSKNADEPQRLQ
eua Guinea_pig   QGLVLPMALELVTLLVGSPR(T)DGLVSLLTTSKDADEPQRLQ
eua Squirrel     QGLVLPMALELVTLLVGSPR(T)DGLVSLLTTSKDADEPQRLQ
eua Rabbit       QGLVLPMALELVTVLVGSPR(E)DGLVSLLTTSEDADEPQRLQ
eua Pika         QGLVLPMALELVTVLVGSRR(E)DGLVSLLTTSKDADEPQRLQ
lau Dog          QGLVLPMALELVTVLVGSPR(A)DGLVSLLTTSEDADEPRRLQ
lau Horse        QGLVLPMALELLTVLVGSPR(A)DGLVSLLTTSEDADEPRRLQ
lau Cow          RGLVLPMALELVTVLVGSPR(V)DGLVSLLTTSEDADEPRRLQ
lau Dolphin      QGLVLPMALELGTVLVGSPR(A)DGLISLLTTSEDADEPRRLQ
lau Megabat      QGLVLPMALELVTVLVGSSR(A)DGLVSLLTTSDDADEXXXLR
lau Microbat     QGLVLPMALELVTVVVGSRR(A)DGLVSLLTTSEGADEPRRLQ
lau Hedgehog     QGLVLPMALELVTVLVGSPR(A)DGLVSLLTTSQDADEPRRLQ
afr Rock_hyrax   QGLVLPMALELMTVLVGSPR(A)DRLVSLLTTSPNADEPRRLQ
xen Armadillo    XXXXXXXALELVTVLVGSPR(V)DGLVSLLTTSEEADEPRRLQ
mar Wallaby      DGLVLPMALELVTVLVGVPR(L)DGLISLVTTSEYADEPRRVE
pro Platypus     XXXXXXXALQLVTVLVGSPR(E)DGLVSLRTTSPSADEPRSXQ

NO 95
GN GALT
IP IPI00013925.3
PE HPLFQAKSARGVC
MP 120
EX Kim
CL simians
DE galactose-1-phosphate uridylyltransferase
SQ
pri Human        PALQPDAPSPGPSDHPLFQA(K)SARGVCKVMCFHPWSDVTLP
pri Chimpanzee   PALQPDAPSPGPSDHPLFQA(K)SARGVCKVMCFHPWSDVTLP
pri Gorilla      PALQPDAPSPGPSDHPLFQA(K)SARGVCKVMCFHPWSDVTLP
pri Orangutan    PALQPDAPSPGPSDHPLFQA(K)SARGVCKVMCFHPWSDVTLP
pri Gibbon       PALQPDAPSPGPSDHPLFQA(K)SARGVCKVMCFHPWSDVTLP
pri Rhesus       PALQPDAPSPGPSDHPLFQA(K)AARGVCKVMCFHPWSDVTLP
pri Baboon       PALQPDAPSPGPSDHPLFQA(K)AARGVCKVMCFHPWSDVTLP
pri Marmoset     PALQPDAPSPGPSDHPLFQA(K)AARGVCKVMCFHPWSDVTLP
pri Tarsier      PALQPDAPSPGPSDHPLFQA(E)AARGVCKVMCFHPWSDVTLP
pri Bushbaby     PALQPDAPSPGPSDHPLFQA(E)AARGVCKVMCFHPWSDMTLP
pri Mouse_lemur  PALQPDAPSPGPSDHPLFQA(E)AARGVCKVMCFHPWSDMTLP
eua Treeshrew    PALQPDAPSPGPSDHPLFQA(E)AARGVCKVMCFHPWSDVTLP
eua Mouse        PALQPDAPDPGPSDHPLFRA(E)AARGVCKVMCFHPWSDVTLP
eua Rat          PALQPDAPDPGPSDHPLFRV(E)AARGVCKVMCFHPWSDVTLP
eua Kangaroo_rat PALQPDAPDPGPSDHPLFRT(E)AARGVCKVMCFHPWSDVTLP
eua Guinea_pig   PALQPDAPDPGPSDHPLFRA(E)AARGVCKVMCFHPWSDVTLP
eua Squirrel     PALQPDAPDPGPSDHPLFQA(G)AARGVCKVMCFHPWSDVTLP
eua Rabbit       PALQPDAPNPGPSDHPLFQA(Q)AARGVCKVMCFHPWSDVTLP
eua Pika         PALQPDAPSPGPSDHPLFQA(Q)AARGVCKVMCFHPWSDVTLP
lau Dog          PALQPDAPNPGPSDHPLFQA(E)AARGVCKVMCFHPWSDVTLP
lau Cat          PALQPDAPSPGPSDHPLFKA(E)AARGVCKVMCFHPWSDVTLP
lau Horse        PALQPDAPNPGPSDHPLFQA(E)AARGVCKVMCFHPWSDVTLP
lau Cow          PALQPDAPSPGPSDHPLFQA(E)AAQGVCKVMCFHPWSDVTLP
lau Dolphin      PALQPDAPSPGPSDHPLFQA(E)AARGVCKVMCFHPWSDVTLP
lau Alpaca       PALQPDAPSPGPSDHPLFQA(E)AARGVCKVMCFHPWSDVTLP
lau Megabat      PALQPDAPNPGPSDHPLFQA(E)AARGVCKVICFHPWSDVTLP
lau Microbat     PALQPDAPSPGPSDHPLFQA(K)AARGVCKVLCFHPWSDVTLP
lau Shrew        PALQPDAPSPGPSDHPLFQA(E)AARGVCKVMCFHPWSDVTLP
afr Elephant     PALQPDAPNPGLSDHPLFQA(E)AARGVCKVMCFHPWSDVTLP
mar Opossum      PALQPDAPSPGPSDHPLFQA(E)AARGVCKVLCFHPWSDITLP
pro Platypus     PALRPDAPSPGPSDHPLFRA(E)PARGVCKVLCFHPWSDVTLP

NO 96
GN GAPDH
IP IPI00219018.7
PE QERDPSKIKWGDA
MP 84
EX Kim, Wagner
CL primates
DE glyceraldehyde-3-phosphate dehydrogenase
SQ
pri Human        NGKLVINGNPITIFQERDPS(K)IKWGDAGAEYVVESTGVFTT
pri Chimpanzee   NGKLVINGNPITIFQERDPS(K)IKWGDAGAEYVVESTGVFTT
pri Gorilla      NGKLVINGNPITIFQERDPS(K)IKWGDAGAEYVVESTGVFTT
pri Orangutan    NGKLVINGNPITIFQERDPS(K)IKWGDAGAEYVVESTGVFTT
pri Gibbon       NGKLVINGNPITIFQERDPS(K)IKWGDAGAEYVVESTGVFTT
pri Rhesus       NGKLVINGSPITIFQERDPS(K)IKWGDAGAEYVVESTGVFTT
pri Baboon       NGKLVINGSPITIFQERDPS(K)IKWGDAGAEYVVESTGVFTT
pri Marmoset     NGKLVINGNPITIFQERDPS(K)IKWGDAGAEYVVESTGVFTT
pri Bushbaby     NGKLVINGNPITIFQERDPT(K)IKWGDAGAEYVVESTGVFTT
pri Mouse_lemur  NGKLVINGQPITIFQERDPT(K)IKWGEAGADYVVESTGVFTT
eua Treeshrew    NGKLVINGKSITIFQERDPA(N)IKWGDAGAEYVVESTGVFTT
eua Mouse        NGKLVINGKPITIFQERDPT(N)IKWGEAGAEYVVESTGVFTT
eua Rat          NGKLVINGKPITIFQERDPA(N)IKWGDAGAEYVVESTGVFTT
eua Guinea_pig   NGKLVINGKAITIFQERDPA(N)IKWGDAGAEYVVESTGVFTT
eua Squirrel     NGKLVINGKSISIFQERDPA(N)IKWGDAGAEYVVESTGVFTT
eua Rabbit       NGKLVINGKAITIFQERDPA(N)IKWGDAGAQYVVESTGVFTT
eua Pika         NGKLVINGKAITIFQERDPA(N)IKWGDAGAQYVVESTGVFTT
lau Dog          NGKLVINGKSISIFQERDPA(N)IKWGDAGAEYVVESTGVFTT
lau Cat          NGKLVINGKPITIFQERDPA(N)IKWGDAGAEYVVESTGVFTT
lau Horse        HGKLVINGKAITIFQERDPA(N)IKWGDAGAEYVVESTGVFTT
lau Cow          NGKLVINGKAITIFQERDPA(N)IKWGDAGAEYVVESTGVFTT
lau Dolphin      NGKLVVNGKAITIFQERDPA(N)IKWGDAGAEYVVESTGVFTT
lau Microbat     NGKLVINGKSISIFQERDPA(N)IKWGDAGAEYVVESTGVFTT
lau Hedgehog     NGKLVINGKPISIFQERDPA(N)IKWGDAGAEYVVESTGVFTT
afr Elephant     NGKLVINGKPISIFQERDPA(N)IKWGDAGAEYVVESTGVFTT
afr Rock_hyrax   NGKLVINGKHITIFQERDPA(N)IKWGDAGAEYVVESTGVFTT
afr Tenrec       NGKLNINGKAISIFQERDPA(N)IKWAEAGAEYVVESTGVFTT
mar Opossum      NGKLVINGKAITISQERDPA(N)IKWGDAGAEYVVESTGVFTT
mar Wallaby      NGKLVINGKAITIFQERDPT(N)IKWGDAGAEYVVESTGVFTT

NO 97
GN GRHPR
IP IPI00037448.3
PE QALASGKIAAAGL
MP 262
EX Kim
CL apes
DE glyoxylate reductase/hydroxypyruvate reductase
SQ
pri Human        NISRGDVVNQDDLYQALASG(K)IAAAGLDVTSPEPLPTNHPL
pri Chimpanzee   NISRGDVVNQDDLYQALASG(K)IAAAGLDVTSPEPLPTNHPL
pri Gorilla      NISRGDVVNQDDLYQALASG(K)IAAAGLDVTSPEPLPTNHPL
pri Orangutan    NISRGDVVNQDDLYQALASG(K)IAAAGLDVTSPEPLPTNHPL
pri Gibbon       NISRGDVVNQDDLYQALASG(K)IAAAGLDVTSPEPLPTNHPL
pri Rhesus       NISRGDVVNQDDLYQALASG(Q)IAAAGLDVTTPEPLPTNHPL
pri Baboon       NISRGDVINQDDLYQALASG(Q)IAAAGLDVTTPEPLPTNHPL
pri Marmoset     NISRGEVVNQDDLYQALASG(Q)IAAAGLDVTTPEPLPTNHPL
pri Bushbaby     NISRGDVVNQDDLYQALASG(Q)IAAAGLDVTTPEPLPTNHPL
pri Mouse_lemur  NISRGDVVNQDDLYQALASG(Q)IAAAGLDVTTPEPLPTNHPL
eua Treeshrew    NISRGDVVNQDDLYQALASG(Q)IAAAGLDVTTPEPLPTNHPL
eua Mouse        NISRGDVVNQEDLYQALASG(Q)IAAAGLDVTTPEPLPPSHPL
eua Rat          NISRGDVVNQEDLYQALASG(Q)IAAAGLDVTTPEPLPPSHPL
eua Kangaroo_rat NISRGDVVNQDDLYEALASG(Q)IAAAGLDVTTPEPLPTNHPL
eua Guinea_pig   NISRGDVVNQDDLYQALASG(Q)IAAAGLDVTTPEPLPTDHPL
eua Squirrel     NISRGDVVNQDDLYQALASG(Q)IAAAGLDVTTPEPLPTNHPL
eua Rabbit       NISRGDVVNQDDLYQALASG(Q)IAAAGLDVTTPEPLPTDHPL
eua Pika         NISRGDVVNQDDLYQALASG(Q)IAAAGLDVTTPEPLPTDHPL
lau Dog          NISRGDVVNQDDLYEALASG(Q)IAAAGLDVTTPEPLPTNHPL
lau Cat          NISRGDVVNQDDLYQALAGG(Q)IAAAGMDVTTPEPLPTNHPL
lau Horse        NISRGDVVNQDDLYQALASG(Q)IAAAGLDVTTPEPLPTDHPL
lau Cow          NISRGEVVDQDDLYQALASG(Q)IAAAGLDVTTPEPLPTNHPL
lau Dolphin      NISRGEVVDQDDLYQALASG(Q)IAAAGLDVTTPEPLPTNHPL
lau Megabat      NISRGEVVNQDDLYQALTSS(Q)IAAAGLDVTTPEPLPTNHPL
lau Microbat     NISRGEVVNQDDLXQALGSG(Q)IAAAGLDVTTPEPLRTSHPL
lau Hedgehog     NISRGDVVNQDDLYQALASG(Q)FAGAGLDVTTPEPLPTSHPL
lau Shrew        NISRGDVVNQDDLYQALASG(R)IAGAGLDVTTPEPLPTDHPL
afr Elephant     NISRGDVVNQDDLYQALAGG(H)IAAAGLDVTTPEPLPTNHPL
afr Tenrec       NISRGDVVNQDDLYQALASG(Q)IAAAGLDVTTPEPLPTNHPL
xen Sloth        XXXXGDVVNQDDLYQALASG(Q)IAAAGLDVTTPEPLPTNHPL
mar Opossum      NISRGDVVNQEDLYQALLNN(Q)IGAAGLDVTTPEPLPTSHPL
pro Platypus     NTSRGGVVNQDDLYQALVKG(Q)IAAAGLDVTVPEPLPTNHPL

NO 98
GN GSDMD
IP IPI00028027.3
PE EVETISKELELLD
MP 299
EX Kim
CL simians
DE gasdermin D
SQ
pri Human        EGAF-TEDFQGLRAEVETIS(K)ELELLDRELCQLLLEGLEGV
pri Chimpanzee   EGAF-TEDFQGLRVEVETIS(K)ELERLDRELCQLLLKGLEGV
pri Gorilla      EGAF-TEDFQGLRAEVETIS(K)ELELLDRELCQLLLEGLEGV
pri Gibbon       EGTF-TEDFQGLRAEVETIS(K)ELELLDRDLCQLLLEGLEGV
pri Rhesus       EGTF-TEDFQGLREEVEAIS(K)ELELLDRELCQLLLEGLEGV
pri Baboon       EGTF-TEDFQGLRAEVEAIS(K)ELELLDRELCQLLLEGLEGV
pri Marmoset     NGTV-TEDFQGLRAEVEVVS(K)ELALMDGQLCRLLLEGLERG
pri Bushbaby     EWEV-TDNFQGLKAEVAARS(V)ELEHMERELSRQLLGGLAGV
pri Mouse_lemur  KQVV-TEDFQGLQAEVGARS(T)ELEHMEPGLSRQLLGDLAAV
eua Mouse        EELIEAADFQGLYAEVKACS(S)ELESLEMELRQQILVNIGKI
eua Rat          EEVI-REDFQGLRAEVEAGS(S)ELRSLEMELRQQLLVDIGRI
eua Kangaroo_rat DWVV-SEDLQGLQAEVEAVS(E)ELQHMETELKRQLLGPLGAV
eua Guinea_pig   KWEI-TEDFQGLRTEVEAGS(A)ELQKMERDLRQQLLLNLEKI
eua Rabbit       WPVL-TEDFQGLRAEVEAKL(L)ELECWDRRLSRKLLGGLAPL
lau Cat          EMPV-TEDFQGLKVEVSVHA(D)GLKGLSGELCGQILAGLMKV
lau Horse        ELVP-TQDFQGLQKEVRAQA(E)GLEGLCKDLREQLLGGLRQV
lau Cow          QLAT-TDDYQGLRAEVKAWA(M)GLEGLSKGLCGQLLGGLGQV
lau Dolphin      ELVT-TEGFQGLQAEVEAWA(V)GLEGLSREPCRQLLGALGQV
lau Megabat      NPVP-TEDFQGLQAEVKAWA(S)ALENGSATLCGQLLGGLGQV
lau Hedgehog     NLML-AQDFEDLQAEVEAQA(E)GLCILSPELCQQLLTGLGQV
afr Elephant     EAVV-TKDFAGLRAEVEPQA(Q)ELENLSKELRQEMLGALGRL
afr Rock_hyrax   EAVV-KKDFAGLKAEVAAKG(Q)VLGRLNCDLRQELLGALQQL
afr Tenrec       GALP-TEDFTGLRTEVETQA(E)LLEPLSCGLRRQLLEALGSL
xen Armadillo    EAAF-TEDLQGLRAEMGTWS(Q)GL-VLVGGPPPVLRAALGQL

NO 99
GN GSR
IP IPI00016862
PE AFTSDPKPTIEVS
MP 181
EX Wagner
CL simians
DE glutathione reductase
SQ
pri Human        NLTKSHIEIIRGHAAFTSDP(K)PTIEVSGKKYTAPHILIATG
pri Chimpanzee   NLTKSHIEIIRGHAAFTSDP(K)PTIEVSGKKYTAPHILIATG
pri Gorilla      XXXXSHIEIIRGHAAFTSDP(K)PTIEVSGKKYTAPHILIATG
pri Gibbon       NLTKSHIEIIRGHAAFTSDP(K)PTIEVSGKKYTAPHILIATG
pri Rhesus       NLTKSHIEIIRGHAAFTSDP(K)PTIEVSGKKYTAPHILIATG
pri Baboon       NLTKSHIEIIRGHAAFTSDP(K)PTIEVGGKKYTAPHILIATG
pri Marmoset     NLTKAHIEIIHGHAVFTSDT(K)PTIEVSGRKYTAPHILIATG
pri Tarsier      NLTKSHIEIIHGHAAFTSDP(S)PTFEVSGKRFTAPHILIATG
pri Bushbaby     NLTKSNIEIIYGYASFTSDP(Q)PTVEVNGKKYTAPHILIATG
pri Mouse_lemur  NLTKSNIEFIHGHAAFTSDP(Q)PTVEVNGKKYTAPHILIATG
eua Mouse        NLTKSHIEIIHGYATFADGP(R)PTVEVNGKKFTAPHILIATG
eua Rat          NLTKSHIEVIHGYATFADGP(Q)PTVEVNGKKFTAPHILIATG
eua Guinea_pig   NLTKSNIEIIHGYASFTSDP(Q)PTVEVNGEKYTAPHILIATG
eua Squirrel     NLTKSHIEIIHGHAAFTCDP(Q)PTVEVNGKKYTAPHILIATG
eua Rabbit       NLTKSHIDIIHGYAAFTSDP(E)PTVEVNGNKYTAPHILIATG
eua Pika         NLTKSHIDIIHGYAAFTNDP(E)PTVEVNGKKYTARHILIATG
lau Dog          NLTKSHIEIIHGHAAFTCDS(E)PTIEVNGNKYTAPHILIATG
lau Cat          NLTKSHIEIIHGHAAFTSDP(K)PTVEVSGNKYTAPHILIATG
lau Horse        NLTKSHIEIIRGHAAFTSDP(R)PTVEVSGKKYTAPHILIATG
lau Cow          NLTKSHIDIIHGHAAFTCDP(Q)PTVEVNGKKYTAPHILIATG
lau Dolphin      NLIKSHIEIIRGHAVFTGDA(Q)PTVEVNGEKYTAPHILIATG
lau Alpaca       NLTKSHIEIIRGHAAFTSDP(Q)PTVEVSGKKYTAPHILIATG
lau Microbat     NLTKAQIEIITGYASFTGDP(R)PTVEVSGEKYTAPHILIATG
lau Shrew        NLTKSHIDIIHGHAAFTSDS(R)PTIEVNGVKYTAPHILIATG
afr Elephant     NLTKAHIDIIHGYGTFTCDP(T)PTVEVNGKKYTAPHILIATG
afr Rock_hyrax   NLTKSNIDIIHGYAAFTCDP(K)PTVEVSGKKYTAPHILIATG
afr Tenrec       NLTKSRIDIIHDCATFTCDR(T)PTVQVNPQRYSAPHILIATS
xen Armadillo    NLTKSHIEIIRGRAAFTGDP(E)PTVEVDGRKFTAPHILIATG
xen Sloth        NLTKSHIEIIRGHAAFTGDP(E)PTVEVNGKKYTAPHILIATG
mar Opossum      NLTKSEIEIIRGHAAFTSDP(E)PTVEVNGKKYRAPHILIATG
pro Platypus     NLNKSHIEIIRGHATFTSDP(E)PTVEVNGRKYSAPHILIATG

NO 100
GN GSS
IP IPI00010706.1
PE EMVQALKQLKDSE
MP 384
EX Kim
CL simians
DE glutathione synthetase
SQ
pri Human        KPQREGGGNNLYGEEMVQAL(K)QLKDSEERASYILMEKIEPE
pri Chimpanzee   KPQREGGGNNLYGEEMVQAL(K)QLKDSEERASYILMEKIEPE
pri Gorilla      KPQREGGGNNLYGEEMVQAL(K)QLKDSEERASYILMEKIEPE
pri Orangutan    KPQREGGGNNLYGEEMVQAL(K)QLKDSEERASYILMEKIEPE
pri Rhesus       KPQREGGGNNLYGEEMVQAL(K)QLKDSEERASYILMEKIEPE
pri Baboon       KPQREGGGNNLYGEEMVQAL(K)QLKDSEERASYILMEKIEPE
pri Marmoset     KPQREGGGNNLYGEEMVQAL(K)QLKDSEERASYILMEKIEPE
pri Bushbaby     KPQREGGGNNLYGEKMVQAL(E)QLKDSEERASYILMEKIEPE
eua Treeshrew    KPQREGGGNNLYGEEMVQAL(E)RLKDSEERASYILMEKIEPE
eua Mouse        KPQREGGGNNLYGEEMVQAL(E)QLKDSEERASYILMEKIEPE
eua Rat          KPQREGGGNNFYGEEMVHAL(E)QLKDSEERASYILMEKIEPE
eua Guinea_pig   KPQREGGGNNLYGDEMVQAL(V)RLKDIEERASYILMEKIEPE
eua Rabbit       KPQREGGGNNLYGEEMVQAL(E)RLKAIEERASYILMEKIEPE
eua Pika         KPQREGGGNNLYGEEMVQAL(V)RLKDSEERASYILMEKIEPE
lau Dog          KPQREGGGNNLYGEEMVQAL(E)RLKDSEERASYILMEKIKPE
lau Horse        KPQREGGGNNLYGEEMVQAL(E)QLKDSEERASYILMEKIEPE
lau Cow          KPQREGGGNNLYGEEMVQAL(E)RLKDSEERASYILMEKIEPE
lau Dolphin      KPQREGGGNNLYGEEMVQAL(E)RLKDSEERASYILMEKIEPE
lau Megabat      KPQREGGGNNLYGEEMVQAL(E)QLKDSEERASYILMEKIEPE
lau Hedgehog     KPQREGGGNNLYGEEMVQAL(V)RLKDCEERASYILMEKIEPE
lau Shrew        KPQREGGGNNLYGEEMVQAL(Q)QLKDSEERASYILMEKIEPE
afr Elephant     KPQREGGGNNLYGEEMVQAL(E)RLKDSEERASYILMEKIEPE
afr Tenrec       KPQREGGGNNLYGEEMVQTL(Q)RLKDSEERASYILMEKIEPE
xen Sloth        XXXXXXXXNNLYGEEMVQAL(E)RLKDSEERASYILMEKIEPE
mar Opossum      KPQREGGGNNLYGEEMAQTL(K)QLKASEKRASYILMEKIEPE
mar Wallaby      KPQREGGGNNLYGEEIAQTL(E)QLKDSEKRASYILMEKIEPE
pro Platypus     XXXXXXXXNNLYGEEMKREL(E)RLKGSAERASYILMDKIEPE

NO 101
GN GSTO1
IP IPI00019755.3
PE ERLEAMKLNECVD
MP 188
EX Kim
CL simians
DE glutathione S-transferase omega 1
SQ
pri Human        NSISMIDYLIWPWFERLEAM(K)LNECVDHTPKLKLWMAAMKE
pri Chimpanzee   NSISMIDYLIWPWFERLEAM(K)LNECVDHTPKLKLWMAAMKE
pri Gorilla      NSISMIDYLIWPWFERLEAM(K)LNECVDHTPKLKLWMAAMKE
pri Orangutan    SSISMIDYLIWPWFERLEAM(K)LNECVGHTPKLKLWMAAMKE
pri Gibbon       NSISMIDYLIWPWFERLEAM(K)LNECVDHTPKLKLWMAAMKE
pri Rhesus       NSISMIDYLIWPWFERLEAM(K)LYECVDHTPKLKLWMAAMKE
pri Baboon       NSISMIDYLIWPWFERLEAM(K)LYECVDHTPKLKLWMAAMKE
pri Marmoset     SSISMIDYLIWPWFERLEAM(K)LNECVDHTPKLKLWMAAMKE
pri Tarsier      NSLSMIDYLIWPWFERLEAL(E)LNECVGHTPKLKLWMATMRK
pri Bushbaby     NSLSMIDYLIWPWFERLEAS(E)LNECIDSTSKLGLWMAVMKE
eua Treeshrew    SSPSIGYDLMWPWFERLEVL(E)LNDYVDHTPKLKLWMAAMKK
eua Mouse        DSPSMVDYLTWPWFQRLEAL(E)LKECLAHTPKLKLWMAAMQQ
eua Rat          NSLSMIDYLIWPWFQRLEAL(E)LNECIDHTPKLKLWMATMQE
eua Kangaroo_rat NSISMIDYLIWPWFERLEPL(E)LTECVSHTPKLKLWMTATRK
eua Guinea_pig   NSIGLVDYLIWPWFERMELQ(E)LTEWAAATPKLKLWMAAMKT
eua Rabbit       SSVSMTDYLIWPWFERLEGL(E)LTDCLDHTPKLKLWMAAMKK
eua Pika         NSVSMTDYLIWPWFERLEGL(E)ITDCVDHTPKLKLWIAAMKK
lau Dog          NSLSMIDYLIWPWFERLEAL(E)LNDCVDHTPKLKLWMAAMRE
lau Cat          NSLSMIDYLIWPWFERLEVL(E)LNECVDHTPKLKLWMAAMRE
lau Horse        KNISMVDYLIWPWFERLEAV(E)LIDCVDHTPKLKLWMEAMRK
lau Cow          SSLSMIDYLIWPWFEWLEAL(E)LNECVDHAPTLKLWMAAMKK
lau Dolphin      SSLSMIDYLIWPWFEWLVAL(E)LNEYVNHTPNLKLWMEAMMK
lau Alpaca       NSPSMIDYLIWPWFERLEAM(E)LNECIDHTANLKLWMAAMMK
lau Microbat     NSLSMIDYLMWPWFGWLEAL(E)LNECVDHTPKLKLWMAAMRK
lau Shrew        SSVSMIDYLMWPWFERLEVL(E)LNECLDHSPKLKLWVAAMRE
afr Elephant     NSVSMIDYLIWPWMERLEAM(E)LSECVDHTPKLKLWIAAMLE
afr Rock_hyrax   SSPSMTDYLIWPWFERLESV(E)LSDCIDHSPKLKLWMAAMGE
xen Armadillo    TSLSMIDYLIWPWFERLEVL(E)LNECVTHTPKLKFWMASMRK
mar Opossum      NTISMIDYLIWPWFERLSAY(G)IAHCVDHTSKLKLWIAAMKK
mar Wallaby      NTISMIDYLIWPWFDRLGSY(G)IADCIDHTSKLKLWVAAMKK

NO 102
GN HEATR2
IP IPI00242630.3
PE TLEEDSKMTRLIS
MP 715
EX Kim
CL humans and chimpanzees
DE HEAT repeat containing 2
SQ
pri Human        IRDVQETLMPQVLTTLEEDS(K)MTRLISCRIINTFLKTSGGM
pri Chimpanzee   IRDVQETLMPQVLTTLEEDS(K)MTRLISCRIINTFLKTSGGM
pri Gorilla      IRDVQETLMPQVLTTLEEDS(Q)MTRLISCHIINTFLKTSGGM
pri Orangutan    IRDVQETLMPQVLTTLEEDS(Q)MTRLISCRIINTFLKTSGGM
pri Gibbon       IRDVQETLMPQVLTTLEEDS(Q)MTRLISCRIINTFLKTSGGM
pri Rhesus       IQDVQETLMPQVLTTLEEDS(Q)MTRLISCRIINTFLKTSGSM
pri Baboon       IQDVQETLMPQVLTTLEEDS(Q)MTRLISCRIINTFLKTSGSM
pri Marmoset     IRDVQETLMPQVLTTLEEDS(Q)MTRLISCRIINTFLKTLGSM
pri Bushbaby     IQDMQGTLMSHILTTLEEDS(Q)MTRLISCRIINIFLKTSDDT
pri Mouse_lemur  IRDVQETLMPHILTTLEEDS(Q)MTRLISCRIINIFLKTSDDV
eua Mouse        VQEAQETLMPQVLATLEDDS(Q)TTRLMSCRIINMFLKNSGDT
eua Rat          VQEAQETLMPQVLATLEDDS(Q)TTRLISCRIISRFLKNSGDT
eua Kangaroo_rat MQEAQETLMAHVLGTLEEDS(M)MTRLTSCHIINTFLKTSGDA
eua Guinea_pig   VQDVQDALLPRVLATLEEDL(Q)TARLISCRIINIFLKTSGDV
eua Squirrel     MQEVREAVMPQVLATLEEDT(Q)TTRLISCRVLNTFLKTAGDT
eua Pika         VEAVQEALLPQVLTTLDEDS(Q)RTRLVSCHIVNSFLKTSGAV
lau Dog          IQQVQETLMPHILTTLEEDS(Q)MTRLISCQIINRFLKSSSGI
lau Cat          IRKVQETLMPQILTSLEEDS(Q)MTRLISCQIINTFLKTSGGV
lau Horse        LREVQEALTPQILTTLEEDA(Q)VTRLVSCRIIDAFMKASGAV
lau Dolphin      IQEVQEMLMPQILTTLEEDS(Q)MTRLISCRIINVFLKTSDGV
lau Megabat      ILDVQETLMPQVLSTLEEDS(Q)MTRLTSCRIINVFLKTSGGA
lau Microbat     VEDVRETLLPPVLTALEEDS(Q)MTRLTSCRIVSAFLKSSGGA
lau Hedgehog     VQAMQDRLLPQVLTTLEEDT(Q)TARLLSCHILCVFLDRSSSP
afr Elephant     ILEVQGVLMPQMVTTLEEDS(Q)MTRLISCRIINLFLQTSGDT
afr Rock_hyrax   IRELQGTLMPQMVTTLEEDS(Q)MTRLMSCRIINLFLQTAGDT
afr Tenrec       ILEAQGALMPQVLTVLEEES(P)TARLLSCRVTNLFLETAGDQ
xen Armadillo    VLGVQQTVLPQVVSALEEDS(Q)TARLLACCIVNVFLKACAGG
mar Opossum      IIEIQEVLMPQIINTLEEDS(K)MTRLMSCRIINIFLKACTDI
mar Wallaby      IIEIQEVLMPQIINTLEEDS(K)MTRLMSCRIINIFLKASSDT
pro Platypus     VLEVQELLLPQIITTLEEDS(K)MTRLLSCRIISIFLKSCGDM

NO 103
GN HEBP1
IP IPI00148063.1
PE PPAPSDKSVKIEE
MP 118
EX Kim
CL simians
DE heme binding protein 1
SQ
pri Human        LKVWFRIPNQFQSDPPAPSD(K)SVKIEEREGITVYSMQFGGY
pri Chimpanzee   LKVWFRIPNQFQSDPPAPSD(K)SVKIEEREGITVYSMQFGGY
pri Gorilla      LKVWFRIPNQFQSDPPAPSD(K)SVKIEEREGITVYSTQFGGY
pri Orangutan    LKVWFRIPNQFQSDPPAPSD(K)SVKIEEREGITVYSMQFGGY
pri Gibbon       LKVWFRIPNQFQSDPPAPSD(K)SVKIEEREGITVYSTQFGGY
pri Rhesus       LKVWFRIPNQFQSDPPAPSD(K)SVKIEEREGITVYSMQFGGY
pri Baboon       LKVWFRIPNQFQSDPPAPSD(K)SVKIEEREGITVYSMQFGGY
pri Marmoset     LKVWFRIPNQFQSDPPAPSD(K)SVKIEEREGITVYSMQFGGY
pri Tarsier      LKVWFRIPNQFQSDPPVPSD(E)SIKIEERESITVYSTQFGGY
pri Mouse_lemur  IKVWFRIPNQFQSNPPIPCD(E)SIKIEERDSITVYSTQFGGY
eua Treeshrew    LKVWFRIPNQFQSNPPIPSD(E)SVKIEERESITVYSLQFGGY
eua Mouse        LKVWFRIPNQFQGSPPAPSD(E)SVKIEEREGITVYSTQFGGY
eua Rat          LKVWFRIPNQFQGSPPTPSD(Q)SVKIEEREGITVYSTQFGGY
eua Guinea_pig   LKVWFRIPNQFQSNPPVPSD(E)SIKIEERESITVYSTQFGGY
eua Squirrel     LKVWFRIPNQFQSNPPIPSD(E)SIKIEERESITVYSTQFGGY
eua Rabbit       LKVWFRIPNQFQSNPPTPSD(E)SIKIEEREGITVYSTQFGGY
eua Pika         LKVWFRIPNQFQSNPPSPSD(D)SIRIEERESITVYSTQFGGY
lau Dog          LKVWFRIPNQFQSNPPVPTD(D)SIKIEERESITVYSLQFGGY
lau Cat          LKVWFRIPNQFQSNPPVPTD(D)SVKIEERESITVYSLQFGGY
lau Horse        LKVWFRIPNQFQSSPPVPSD(D)SVKIEEREGITVYSTQFGGY
lau Cow          LKVWFRIPNKFQSDPPAPSD(D)SIKIEDREGITVYSTQFGGY
lau Microbat     IKVWFRIPNQFQSDPPVPSD(N)SIKIEERESITVYSTQFGGY
afr Elephant     LKVWFRIPNQFQSDPPVPSD(D)SIKIEEREGITIYSKQFGGY
xen Armadillo    LKVWFRIPDQFQSNPPVPSD(E)SIKIEEREGITVYSTQFGGY
xen Sloth        LKVWFRIPNQFQSNPPVPSD(E)SIKIEEREGITVYSMQFGGY
mar Opossum      VKVWFRIPSQFQADTPIPSD(N)SIKLEERGSITVYSKQFGGY

NO 104
GN HPRT1
IP IPI00218493.7
PE PRSVGYKPDFVGF
MP 175
EX Kim, Wagner
CL catarrhines
DE hypoxanthine phosphoribosyltransferase 1
SQ
pri Human        PKMVKVASLLVKRTPRSVGY(K)PDFVGFEIPDKFVVGYALDY
pri Chimpanzee   PKMVKVASLLVKRTPRSVGY(K)PDFVGFEIPDKFVVGYALDY
pri Gorilla      PKMVKVASLLVKRTPRSVGY(K)PDFVGFEIPDKFVVGYALDY
pri Orangutan    PKMVKVASLLVKRTPRSVGY(K)PDFVGFEIPDKFVVGYALDY
pri Gibbon       PKMVKVASLLVKRTPRSVGY(K)PDFVGFEIPDKFVVGYALDY
pri Rhesus       PKMVKVASLLVKRTPRSVGY(K)PDFVGFEIPDKFVVGYALDY
pri Baboon       PKMVKVASLLVKRTPRSVGY(K)PDFVGFEIPDKFVGGYALDY
pri Marmoset     PKMVKVASLLVKRTPRSVGY(R)PDXXXXXXXXXXXXXXXXXX
pri Tarsier      PKMVKVASLLVKRTPRSVGY(R)PDFVGFEIPDKFVVGYALDY
pri Bushbaby     XXXXXXXXLLVKRTPRSVGY(R)PDFVGFEIPDKFVVGYALDY
pri Mouse_lemur  PKMVKVASLLVKRTPRSVGY(R)PDFVGFEIPDKFVVGYALDY
eua Treeshrew    PKMVKVASLLVKRTPRSVGY(R)PDFVGFEIPDKFVVGYALDY
eua Mouse        PKMVKVASLLVKRTSRSVGY(R)PDFVGFEIPDKFVVGYALDY
eua Rat          PKMVKVASLLVKRTSRSVGY(R)PDFVGFEIPDKFVVGYALDY
eua Guinea_pig   PKMVKVASLLVKRTPRSVGY(R)PDFVGFEIPDKFVVGYALDY
eua Rabbit       PKMVKVASLLVKRTPRSVGY(R)PDFVGFEIPDKFVVGYALDY
eua Pika         XXXXXXXXLLVKRTPRSVGY(R)PDFVGFEIPDKFVVGYALDY
lau Dog          PKMVKVASLLVKRTPRSVGY(K)PDFVGFEIPDKFVVGYALDY
lau Cat          PKMVKVASLLVKRTPRSVGY(K)PDFVGFEIPDKFVVGYALDY
lau Horse        PKMVKVASLLVKRTPRSVGY(R)PDFVGFEIPDKFVVGYALDY
lau Cow          PKMVKVASLLMKRTPRSVGY(K)PDFVGFEIPDKFVVGYALDY
lau Alpaca       PKMVKVASLLVKRTPRSVGY(R)PDFVGFEIPDKFVVGYALDY
lau Megabat      PKMVKVASLLVKRTPRSVGY(R)PDFVGFEIPDKFVVGYALDY
lau Hedgehog     PKMVKVASLLVKRTPRSVGY(R)PDFVGFEIPDKFVVGYALDY
lau Shrew        PKMVKVAXXLVKRTPRSVGY(R)PDFVGFEIPDKFVVGYALDY
afr Elephant     PQMIKVASLLVKRTPRSVGY(R)PDFVGFEIPDKFVVGYALDY
xen Armadillo    PKMVKVASLLVKRTPRSVGY(R)PDFVGFEIPDKFVVGYALDY
xen Sloth        PKMVKVAXXLVKRTPQSVGY(R)PDFVGFEIPDRFVVGYVLDY
mar Opossum      PKMVKVASLLVKRTPRSVGY(R)PDFVGFEIPDKFVVGYALDY
mar Wallaby      PRMVKVXXVLVKRTPRSVGY(R)PDFVGFEIPDKFVVGYALDY
pro Platypus     PKMVKVASLLVKRTPRSVGY(R)PDFVGFEIPDKFVVGYALDY

NO 105
GN HPS6
IP IPI00015505.7
PE QLDGNGKLRSQAP
MP 534
EX Kim
CL primates
DE Hermansky-Pudlak syndrome 6
SQ
pri Human        PTAAWGATLRALQLQLDGNG(K)LRSQAPPDVWKKVLGGITAG
pri Chimpanzee   PTAAWGATLRALQLQLDGNG(K)LRSQAPPDVWKKVLGGITAG
pri Gorilla      PTAAWGATLRALQLQLDGNG(K)LRSQAPPDVWKKVLGGITAG
pri Orangutan    PTAAWGATLRALQLQPDGNG(K)LRSQAPPDVWKKVLGGITAG
pri Gibbon       PTAAWGATLRALQLQPDGNG(K)LRSQAPPDVWKKVLGGITTG
pri Rhesus       PTAAWGATLRALQLQTDGNG(K)LRSQAPPDVWKKVLGGITAV
pri Baboon       PTAAWGATLRALQLQPDGNG(K)LRSQAPPDVWKKVLGGITAV
pri Marmoset     PTAAWGAALRALQLQPDGNG(K)LRSQAPPDVWKKVLGGITAG
pri Mouse_lemur  PTAAWAATLRTLQLQPDGNG(K)LRSQAPPDVWKKVLWGTTAG
eua Treeshrew    PTAAWGATLRALQLQPDGNG(R)LSSQAPPDVWKKVLRGTSAG
eua Mouse        PTAAWGATLQALQLQPDRSG(R)LRSQAPPDVWKKVLRAPTAG
eua Rat          PTAAWSATLQALQLQPDRSG(R)LRSQAPPDVWKKVLRAPTAG
eua Guinea_pig   PSAAWGATLRALQLQPDGNG(R)LSSQAPTDVWKKVLGATAAI
eua Pika         PTAAWGTILRTLQLQLDGNG(R)LRSQAPPDVWKKVLDGPAAG
lau Dog          PTAAWGAILRALQLQPDGKG(R)LKSHAPPDVWKKVLGGTSAG
lau Cat          SSAAWGAVFRAEQLQPDGNG(R)LRSHAPPDVWKKLLGGSSAG
lau Horse        PTAAWGAILRVLQLQPDGNG(K)LRSQAPPDVWKKVLGGTAAG
lau Cow          PTAAWGAILRVLQLQPDGNG(H)LRSQAPPDVWKKVLGVTAAG
lau Dolphin      PTAAWGAILRALQLQPDGNG(R)LRSQAPPDVWKKVLGVTTGG
lau Alpaca       PTAAWGAILRALQLQPDGNG(R)LRSQAPPDVWKKVLAGTATG
lau Megabat      PTAAWGAILRALQLQPDGNG(R)LRSQAPPDVWKKVLGGTAAG
lau Microbat     PTAAWGAILRALQLQPDGNG(R)LRSQAPPDVWKKVLGGTAVG
lau Hedgehog     PIAAWGAILRALQLQPDGNG(R)LRSQAPPDVWKKVLGSTAAG
lau Shrew        PTAAWGAILRALQLQPDGSG(R)LKSQAPPDTWKKVLAGTAAG
afr Elephant     PTAAWSATHRALQLQPDGDG(R)LRSQAPPDVWKKVLGGTAAG
afr Tenrec       PTAAWGATHRALQLQLDRAG(R)LRSQAPPDVWKKVLRGTAAG
xen Armadillo    PTAAWGAAHRALQLQPDGNG(R)LRSQAPPDVWKKVLGSTAAR
mar Opossum      PAAAWGSTRRALQLQLDKAG(Q)LKSQAPPDLWKKVLADTG--
mar Wallaby      PAAAWCSTRRALQLQLNEGS(Q)LKSQAPPDLWKKVLADTEA-

NO 106
GN HSD17B12
IP IPI00007676.3
PE VLISRSKDKLDQV
MP 84
EX Kim
CL African great apes
DE hydroxysteroid (17-beta) dehydrogenase 12
SQ
pri Human        KSYAEELAKHGMKVVLISRS(K)DKLDQVSSEIKEKFKVETRT
pri Chimpanzee   KSYAEELAKHGMKVVLISRS(K)DKLDQVSSEIKEKFKVETRT
pri Gorilla      KSYAEELAKHGMKVVLISRS(K)DKLDQVSSEIKEXFKVETRT
pri Orangutan    KSYAEELAKHGMKVVLISRS(Q)DKLDQVSSEIKEKFKVETRT
pri Gibbon       KSYAEELAKRGMKVVLISRS(Q)DKLDQLSSEIKEKFKVETRT
pri Rhesus       KSYAEELAKRGMKVVLISRS(Q)DKLDQVSSEIKEKFKVETRT
pri Baboon       KSYAEELAKRGMKVVLISRS(Q)DKLDQVSSEIKEKFKVETRT
pri Marmoset     KSYAEELAKRGMKVVLISRS(Q)DKLDQVSSEIKEKFKVETRT
pri Tarsier      XXXXXXLAKRGMKVVLVSRS(Q)DKLNQVSSEIKEKFKVETRT
pri Bushbaby     KSYAEELAKRGMKVVLISRS(Q)DKLNQVSSDIXXKFKVETZT
eua Treeshrew    KSYAEELARHGMKVVLISRS(Q)DKLNQVSSEIKEKFKVETRT
eua Mouse        KAYAEELAKRGMKIVLISRS(Q)DKLNQVSNNIKEKFNVETRT
eua Rat          KSYAEELAKRGMKIVLISRS(Q)DKLKEVSNNIKEKFNVETRT
eua Kangaroo_rat XXXXXXLAKRGRKIVLISRS(Q)DKVNQVSSDIKEKFKVETRT
eua Guinea_pig   KSYAEQLAKRGMKIVLVSRS(Q)DKLNQVSSEIREKFKVETKT
eua Squirrel     KAYAEELANHGMKVVLISRS(Q)DKLNQVSSEIXXXXXXXXXX
eua Rabbit       KSYAEQLAKRGMNVVLISRS(Q)DKLNQVSNEIKEKFKVETRT
eua Pika         KSYAEQLAKRGMKIVLISRS(Q)DKLSQVASEIKEKFKAETRT
lau Dog          KSYAEELAKHGMKVVLISRS(Q)DKLNQVSSEIREKFKVETRT
lau Cat          XXXXXXLAKHGMKIVLISRS(Q)DKLNQVSSEIREKFKVETMT
lau Horse        KSYAEELAKRGMKIVLISRS(Q)DKLDQVSSEIREKFKVETRT
lau Cow          KSYAEKLAERGMKIVLISRS(Q)DKLNQVSSEIREKFKVETKT
lau Dolphin      KSYAEELAKRGMKIVLISRS(Q)DKLNQVSSEIREKFKVETKT
lau Alpaca       KSYAEELAKRGMKIVLISRS(Q)DKLNQVSSEIREKFKVETKT
lau Megabat      KSYAKELAKRGMKIVLISRS(Q)DKLNQVSSEIREKFKVETRT
lau Hedgehog     KAYAEELAKHGMKIVLISRS(Q)DKLNQISSEIXXXXXXXXXX
afr Elephant     KSYAEELAKRGMKVVLISRS(Q)DKLNQVSSEIREKFNVETRT
xen Sloth        KLYAEELAKRGMKVVLISRS(Q)DKLKQVSSEIXXXXXXXXXX
mar Opossum      RSYAEELAKRGMKIVLISRS(Q)EKLKEVANDIKEKFKVETKT
mar Wallaby      KSYAEELAKRGMKIVLISRS(Q)EKLKEVANAIREKFKVETKT
pro Platypus     KAYAEELAKRGLKIVLISRS(Q)EKLNQVAQEIKENFKVETKT

NO 107
GN HSPBAP1
IP IPI00298207.5
PE DLFQDVKWSDFGF
MP 147
EX Kim
CL primates
DE HSPB (heat shock 27kDa) associated protein 1
SQ
pri Human        ADYKYFVSLFEDKTDLFQDV(K)WSDFGFPGRNGQESTLWIGS
pri Chimpanzee   ADYKYFVSLFEDKTDIFQDV(K)WSDFGFPGRNGQESTLWIGS
pri Gorilla      ADYKYFVSLFEDKTDLFQDV(K)WSDFGFPGRNGQESTLWIGS
pri Orangutan    ADYKYFVSLFEDKTDLFQDV(K)WSDFGFPGRNGQESTLWIGS
pri Gibbon       ADYKYFVSLFEDKTDLFQDV(K)WSDFGFPGRNGQESTLWIGS
pri Rhesus       ADYKYFVSLFEDKTDIFQDV(K)WSDFGFPGRNGQESTLWIGS
pri Baboon       ADYKYFVSLFEDKTDIFQDV(K)WSDFGFPGRNGQESTLWIGS
pri Marmoset     ADYKYFLSLFEDKTDIFQDV(K)WSDFGFPGRNGQESTLWIGS
pri Bushbaby     DDYTXFFSLLEDKTWIFQDV(K)WSDFGFPGRNGRESTLWIGS
pri Mouse_lemur  ADYKYFVSLFEDKTDIFQDV(K)WSDFGFPGRNGQESTLWIGS
eua Mouse        ADYKYFVTLFEDKTDVFQEV(V)WSDFGFPGRNGQESTLWIGS
eua Rat          ADYKYFVTLFEDKTDVFQEV(M)WSDFGFPGRNGQESTLWIGS
eua Kangaroo_rat ADYKYLISLFEDRADVFQDV(I)WSDFGFPGRNGQESTLWIGS
eua Guinea_pig   ADYKYFVNLFEDNSDVFQDV(L)WSDFGFPGRNGQESTLWIGS
eua Rabbit       ADYKYFVNLFEDKTDVFQEV(M)WSDFGFPGRNGQESTLWIGS
eua Pika         ADYKYFVNLFEDKTDVFQEV(M)WSDFGFPGRNGQESTLWIGS
lau Dog          ADYKYFISLFDDKTDIFQDV(I)WSDFGFPGRNGRESTLWIGS
lau Cat          ADYKYFVSLFDDKTDIFEGV(I)WSDFGFPGRNGRESTLWIGS
lau Horse        ADYKYFVNLFEDKTDIFQDV(M)WSDFGFPGRNGRESTLWIGS
lau Cow          ADYKYFVSLFEDKTDIFQDV(I)WSDFGFPGRNGQESTLWIGS
lau Dolphin      ADYKYFVSLFEDKTDIFQDV(M)WSDFGFPGRNGQESTLWIGS
lau Alpaca       ADYKYFINLFEDKTDVFQDV(I)WSDFGFPGRNGRESTLWIGS
lau Megabat      ADYKYFVSLFEDKTDIFQDV(I)WSDFGFPGRSGQESTLWIGS
lau Microbat     ADYKYVVSLFEDKTDIFQDV(I)WSDFGYRGRNGRESTLWIGS
lau Shrew        ADYKYCVSLFEDKTDIFQDV(I)WSDFGFPGRNGRESTLWIGS
afr Elephant     ADYKYFVRLFENKTDVFQDV(L)WSDFGFPGRNGRESTLWIGS
xen Armadillo    ADYKYFVHLFEDKTDIFQDV(I)WSDFGFPGRTGRESTLWIGS
mar Opossum      ADYKYLVNVFEDNTDVLQNV(M)WSDFGFPGRDGRESTLWIGS

NO 108
GN HYLS1
IP IPI00065180.3
PE VQFQEDKESSFDV
MP 144
EX Kim
CL catarrhines
DE hydrolethalus syndrome 1
SQ
pri Human        NDQDLWDLRQRLMNVQFQED(K)ESSFDVSQKFNLPHEYQGIS
pri Chimpanzee   NDQDLWDLRQRLMNVQFQED(K)ESSFDVSQKFNLPHEYQGIS
pri Gorilla      NDQDLWDLRQRLMNVQFQED(K)ESSFDVSQKFNLPHEYQGIS
pri Orangutan    NDQDLWDLRQRLMNVQFQED(K)ESSFDVSQKFNLPHEYQGIA
pri Gibbon       NDQDLWDLRQRLMNIQFQED(K)ESSFDVSQKFNLPHEYQGIS
pri Rhesus       NDQGLWDLRQRLMNVQFQED(K)ESSFDISQKFNPPHEYQGIS
pri Baboon       NDQGLWDLRQRLMNVQFQED(K)ESSFDISQKFNPPHEYQRIS
pri Marmoset     NDQGLWDLRQRLMNVQFEED(R)ESSFDISQKLNPPYEYQGIS
pri Tarsier      NDLDLWDLRQRMMNLQFQED(R)ESPVDISQKFNLPHEYEEIS
pri Bushbaby     SDPDLWDLRQRLMNLQVQED(K)ESTADSTQKFNLPHENQGIS
pri Mouse_lemur  SDPDLWDLRQRLMNMQFQED(K)ESPVDSSQSFNLPHEYQGIS
eua Mouse        SDLGLWDLRHRFMNLQFQEG(T)ESPVVTSQKFNLPCEYQGIS
eua Rat          SDLDLWDLRHRLMNLQFQEG(T)ASPVDTSQKLNLPCEYQGIS
eua Guinea_pig   SDTDLWDLKQRLMNLQFQED(R)ESPVDISQKFNQPPEHQGIS
eua Squirrel     TDLDLWDLRQRLMNLQFQED(R)ESSIDTSQNVNLPHEYQGIS
eua Rabbit       SDLYLCDLRQRLMNLQFQED(R)ESPIDISQKFNLPHEYQGNS
eua Pika         SDLYLCDLRQKLMNLQFQED(R)ESPVDISQKFNPPHEYQGIS
lau Dog          SDMDLWDLRQKLMSLQFQED(M)DSPDDIPQKFTLPNEYQGIS
lau Cat          SDMDLWDLRQKLMSLQFQED(R)ESPADISQKFNLPHEYQGIS
lau Horse        SDVDLWDLRQRLMNLQFQED(R)ESPVDTSQKFNLPHEYQGIS
lau Cow          NDMDVWDLRQRLMNLHFQED(R)ESPVDVSQKFSLPREYQGIS
lau Dolphin      SDMDVWDLRQRLMNLQFQDD(R)ESPVDISQKFSLPREYQGIS
lau Alpaca       SDVDVWDLRQRLMNLQFQED(R)GSPADSSQKFNLPHEYQGMS
lau Megabat      SDMDLWDLRERLMNLQFQED(R)ESPVDTSEKCNPPHEYQGIS
lau Hedgehog     SDMDLWDLRQKLMNLQFQED(R)ESSLAESQKFSLPEGYPGIS
lau Shrew        SGGDLWALRERLMNLQFQED(R)ETSVDESGTFDLSDEYQEIS
afr Elephant     SDMDLWDLRQRLMNMQFQED(R)ESPVEISQKFNIPHEYQTLT
afr Tenrec       SETDLWDLRQRLANLQFQED(R)KSPNERSQKFSLPHEYQRLT
xen Armadillo    SDIDLWDLRQKLMNLQLQED(R)ESPLDISQKFNLPHEYQGIS
xen Sloth        SDIDLWDLRQRLMNLQLQED(K)ESPLGISQKFNLPHEYQGIS
mar Opossum      NEMELWDLRRRLMNLQPQED(N)ESEVDDSGKFYPQHSYHDIS
mar Wallaby      KDIDLWDLRQRLMNLQCQED(N)EAEMDSSKKLYPQHSYHDIS

NO 109
GN IFT122
IP IPI00066817.7
PE LRLVETKDSIGDE
MP 1141
EX Kim
CL simians
DE intraflagellar transport 122 homolog (Chlamydomonas)
SQ
pri Human        DDRQLEIANNSSQILRLVET(K)DSIGDEDPFTAKLSFEQGGS
pri Chimpanzee   DDRQLEIANNSSQILRLVET(K)DSIGDEDPFTAKLSFEQGGS
pri Gorilla      DDRQLEIANNSSQILRLVET(K)DSIGDEDPFTAKLSFEQGGS
pri Orangutan    DDRQLEIANNSSQILRLVET(K)DSMGDEDPFTAKLSFEQGGS
pri Gibbon       DDRQQEIA-NSSQILRLVET(K)DSMGDEDPFTAKLSFEXXXX
pri Rhesus       DNRQQEIANSSSQILRLVET(K)DSMGDEDPFTAKLSFEQGGS
pri Baboon       DNRQQEIANSSSQILRLVET(K)DSMGDEDPFTAKLSFEQGGS
pri Marmoset     EDRQRETINNSSQILQLVET(K)DSMGDEDPFTAKLSFEQGGS
pri Tarsier      ESKWQEITSHDSQTLRLDET(I)DSMGDGDPFTAKLSFEXXXX
pri Bushbaby     ENKWQEIRNNDSQTLRLDET(M)DSLGD-DPFTAKLSFEQGGS
pri Mouse_lemur  ENKWQEIASNNSQTLRLDET(V)DSMGDDDPFTAKLSFEQGGS
eua Mouse        EGKWRETSSNNSQTLKLDET(M)DSIGEDDPFTAKLSFEQGSS
eua Rat          EGKWQETSSNNSQTLRLDET(M)DSIGEDDPFTAKLSFEQGGS
eua Kangaroo_rat GSKWQEISSNNSQALRLDET(V)DPVGEDDPFTAKLSFEQGGS
eua Guinea_pig   EDRWQEVASGDSQTLRLDET(M)NPXXSDDPFTAKLSFEQGGS
eua Rabbit       ENKWQEVTSSNAQTLRLEET(Q)DSMGDDDPFTAKLSFEQGGS
eua Pika         ESKWQEITGSNSQTLRLEET(Q)ESMGDDDPFTAKLSFEQGGS
lau Dog          ENKWQEIASNNSQTLRLDET(M)DSMGDDDPFTAKLSFEQGGS
lau Cat          ENKWQEITSSNSQTLRLDET(T)DSMRDDDPFTAKLSFEQGGS
lau Horse        ENKWQEITSNNSQTLRLDET(M)DSMGDDDPFTAKLSFEQGGS
lau Cow          ENKWQEITSNNAQTLRLDES(M)DSVGDDDPFTAKLSFEQGGS
lau Dolphin      ENKWQEIASNTSQTLRLDET(L)DSMGDDDPFTAKLSFEQGGL
lau Megabat      ENKWQEITSNNSQTLRLDET(M)DSLGDDDPFTAKLSFEQGGS
lau Microbat     ENKWQEITGNNSQTLRLDET(M)NSMGDDDPFTAKLSFEQGGS
lau Hedgehog     DSKWQEVTSNNSQTLRLDET(T)DAGGDGDPFTARLSFEQGGS
lau Shrew        XXXXXXXXXXXSQTLRLDES(M)DTMGDSDPFTAKLSFEQGGS
afr Elephant     ESKWQEITSNNSQTLRLDET(V)DSMGDDDPFTAKLSFEQGGS
afr Rock_hyrax   XXXXXXXXXXXSQTLRLDET(M)DSVSDDDPFTAKLSFEQGGS
afr Tenrec       XXXXXXXXXXXSQTLRLDET(V)DPMGDDDPFPAKLSFEQGGS
xen Armadillo    ESKWQEITSSNSQILRLDET(V)DSPGDDDPFTAKLSFEQGGS
mar Opossum      ERKWQEIANNNSQTLRLDET(V)DRIEEDDPFTAKLSFEQGGS
mar Wallaby      ERKWQEITNPNSQTLRLDET(V)DHIEEDDPFTAKLSFEQGGS
pro Platypus     ENQWKETESSNSQSLQLSEK(V)GNIKQEDPFTAKLSFEQGNS

NO 110
GN ISYNA1
IP IPI00549569
PE EAMRRAKVLDWGL
MP 155
EX Wagner
CL primates
DE inositol-3-phosphate synthase 1
SQ
pri Human        LVFDGWDISSLNLAEAMRRA(K)VLDWGLQEQLWPHMEALRPR
pri Gorilla      LVFDGWDISSLNLAEAMRRA(K)VLDWGLQEQLWPHMEALRPR
pri Gibbon       LVFDGWDISSLNLAEAMRRA(K)VLDWGLQEQLWPHMEALRPR
pri Rhesus       LVFDGWDISSLNLAEAMRRA(K)VLDWGLQEQLWPHMEALRPR
pri Baboon       LVFDGWDISSLNLAEAMRRA(K)VLDWGLQEQLWPHMEALRPR
pri Marmoset     LVFDGWDISSMNLAEAMRRA(K)VLDWELQEQLWPHMEALRPR
pri Bushbaby     LVFDGWDISSLNLAEAMRRA(K)VLDWGLQEQLWPHMEGLRPR
pri Mouse_lemur  LVFDGWDISSLNLAEAMRRA(Q)VLDWGLQEQLWPHMEGLRPR
eua Treeshrew    LVFDGWDISSLNLAQAMRRA(Q)VLDWGLQEQLWSHMETMRPR
eua Mouse        LVFDGWDISSLNLAEAMRRA(Q)VLDCGLQEQLWPHMESLRPR
eua Rat          LVFDGWDISSLNLAEAMRRA(Q)VLDCGLQEQLWPHMESLRPR
eua Kangaroo_rat LVFDGWDISSLNLAEAMRRA(Q)VLDCHLQEQLWPHMERLRPR
eua Guinea_pig   LVFDGWDISSLNLAEAMRRA(Q)VLDCGLQEQLWPHMETLRPR
lau Dog          LVFDGWDISSLNLAEAMRRA(Q)VLDWGLQEQLWPHLEALRPR
lau Cat          LVFDGWDISSVNLAEAMRRA(Q)VLDWGLQEQLWPHMESLRPR
lau Cow          LVFDGWDISSLNLAEAMRRA(Q)VLDWGLQEQLWPHMEAMRPR
lau Dolphin      IVFDGWDISSLNLAEAMRRA(Q)VLDWGLQEQLWPHMEALRPR
lau Megabat      LVFDGWDISSLNLAEAMRRA(Q)VLDWGLQEQLWPHMEALRPR
lau Microbat     LVFDGWDISSLNLAEAMRRA(K)VLDWGLQEQLWPHMETLRPR
afr Elephant     LVFDGWDISSLNLAQAMRRA(Q)VLDWGLQEQLWPHMEVLHPR
afr Tenrec       LEFDGWDISSLNLAQAMRRA(Q)VLDWGLQEQLWSHMESLRPR
mar Wallaby      IVFDGWDISSLNLAEAMKRA(Q)VLDWALQEQLWPYMEELQPR

NO 111
GN ITGA3
IP IPI00215995.1
PE VSEQQQKLSRLQY
MP 638
EX Kim
CL simians
DE integrin, alpha 3 (antigen CD49C, alpha 3 subunit of VLA-3 receptor)
SQ
pri Human        DNKCESNLQMRAAFVSEQQQ(K)LSRLQYSRDVRKLLLSINVT
pri Chimpanzee   DNKCESNLQMRAAFVSEQQQ(K)LSRLQYSRDVRKLLLSINVT
pri Gorilla      DNKCESNLQMRAAFVSEQQQ(K)LSRLQYSRDVRKLLLSINVT
pri Orangutan    DNKCESNLQMRAAFVSEQQQ(K)LSRLQYSRDVRKLLLSINVT
pri Gibbon       DNKCESNLQMRAAFVSEQQQ(K)LSRLQYSRDVRKLLLSINVT
pri Rhesus       DNKCESNLQMRAAFVSEQQQ(K)LSRLQYSRDVRKLLLSINVT
pri Baboon       DNKCESNLQMRAAFVSEQQQ(K)LSRLQYSRDVRKLLLSINVT
pri Marmoset     DNKCESNLQMRAAFVSEQLQ(K)LSRLQYSRDVRKLLLSINVT
pri Tarsier      DNQCHSNLQMRAAFVSEHLQ(R)LTRLQYSRDVRKLLLSINVT
pri Bushbaby     DNKCESNLQMRAAFLSEQLQ(R)LSRLPYSRDVRKLYLGTNVT
pri Mouse_lemur  DNKCESNLQMRAAFVSEQLQ(R)LSRLQYSRDVRKLLLSINVT
eua Treeshrew    DNKCESNLQMRAAFLSDQLQ(R)LSRLHYNRDIRKLHLSINVT
eua Mouse        DNKCDSNLQMRAAFLSEQLQ(P)LSRLQYSRDTKKLFLSINVT
eua Rat          DNKCDSNLQMRAAFVSEQLQ(P)LSRLQYSRDTKKLFLSINVT
eua Kangaroo_rat DNKCDSNLQMRAAFVSEQLQ(R)LSRLQYSRDVRKLLLSINVT
eua Guinea_pig   DNRCDSNLQMRAAFVSDQLQ(P)LSRFQYSRDVRKLLLSINVT
eua Rabbit       DNKCDSNLQMRAAFVSEQGM(P)MSRLQYSRDVRRLLLSINVT
eua Pika         DNKCDSNLQMRAAFVSEQGQ(Q)LSRLQYSRDVRRLLLSINVT
lau Dog          DNKCDSNLQMQAAFVSELGQ(P)LSRLQYSRDGRKLFLSIDVT
lau Cat          DNICDSNLQMQAAFVSELGQ(P)LSRLHYSRDVRKLHLSINVT
lau Horse        DNKCDSNLQMRAAFVSEQGQ(R)LSRLQYSRDVRKLLLSINVT
lau Cow          DNRCDSNLQMRAAFVSELGQ(R)LSRLQYRRDLRKLLLSINVT
lau Megabat      DNKCDSNLEMQAAFVSEQGQ(P)MSRLQYSRDFGKLLLSINVT
lau Microbat     DNKCDSNLQMQAAFVSEQGQ(R)LSRLQYSKDLRKLFLSINVT
lau Hedgehog     DNKCDSNLQMKAAFMSELGQ(R)LSRLHYSKDTRKLLLSINVT
lau Shrew        DNRCDSNLQMQAAFMSEQGQ(Q)VSRXXYSGEVRKLLLSVNVT
afr Elephant     DNKCESNLGMQADFVTEQMQ(R)LRRLQYGRDTRKLLLSINVT
afr Rock_hyrax   DNKCESNLVMQANFVTEQMQ(Q)LRRLQYGRDTRKLLLSINVT
afr Tenrec       DNKCESNLEMRAAFVTEQMQ(Q)LRRLQYGRDARKLFLSINVT
xen Armadillo    DNKCDSNLQMRAAFVSEQMQ(P)QSRLQYSRDVRKLLLSINVT
mar Opossum      DNKCDSNLQLQAAFLSEQLQ(P)MNRLQYSRDVRKLFLNINVT

NO 112
GN ITGAL
IP IPI00219896
PE WLPSRQKTSLLAS
MP 416
EX Wagner
CL simians
DE integrin, alpha L (antigen CD11A (p180), lymphocyte function-associated antigen 1; alpha polypeptide)
SQ
pri Human        TPEVRAGYLGYTVTWLPSRQ(K)TSLLASGAPRYQHMGRVLLF
pri Chimpanzee   TPEVRAGYLGYTVTRLPSRE(K)TSLLASGAPRYQHVGRVLLF
pri Gorilla      TPEVRAGYLGYTVTWLPSRE(K)TSLLASGAPRYQHVGRVLLF
pri Orangutan    TPEVRAGYLGYTVTWLPSRE(K)TSLLASGAPRYQHVGRVLLF
pri Gibbon       TPEVRAGYLGYTVTWLPSQE(K)TLLLASGAPRYQHVGRVLLF
pri Rhesus       TPEVRAGYLGYSVTWLPSGE(K)TSLLASGAPRYQHVGRVLLF
pri Baboon       TPEVRAGYLGYTVTWLPSGE(K)TSLLASGAPRYQHVGRVLLF
pri Marmoset     TPEVRAGYLGYTMTWLPSRE(K)TWLLASGAPRYQHVGRVLLF
pri Tarsier      TPEVKAGYLGYTVTWLPSPG(H)TSLLAAGAPRYQHVGRVLLF
pri Mouse_lemur  TPEVRAGYLGYTVTWLPSQG(N)TRLLAAGAPRYQHVGRVLLF
eua Mouse        TSDVRGGYLGYTVAWMTSRS(S)RPLLAAGAPRYQHVGQVLLF
eua Rat          TSDERGGYLGYTVAWLTSRS(S)RPLLAAGAPRYQHVGQVLLF
eua Kangaroo_rat TQEAKEGYLGYTVTWLPSRG(P)TSLLAAGAPRYQHVGQVLVF
eua Guinea_pig   TPEVRAGYLGYTVTWLPFQG(S)VSLVAAGAPRYQHVGQVLLF
eua Squirrel     TPEVRAGYLGYTVTWLSSRG(P)TSLLAAGAPRYQHVGRVLLF
eua Rabbit       TPEAKSGYLGYTVTWLPSQG(H)TSLLAAGAPRYQHVGQVLLF
eua Pika         TSDARSGYLGYTVTWLPSLR(N)APLLATGAPRYQHVGQVLLF
lau Dog          TPEARAGYLGYTVTWLYSRG(L)TSLLAAGAPRYQHVGRVVLF
lau Horse        TKEVKEGYLGYTVAWLPSRG(L)PSLLAAGAPRYQHVGRVLLF
lau Megabat      TTEVRAGYLGYTVTWLPSLG(P)TSMLAAGAPRYQHVGRVLLF
lau Microbat     TPEARDGYLGYTVTWLPSRG(L)TALLATGAPRYQHVGRVLLF
lau Hedgehog     TPEVKDSYLGYTVTWLPSRG(S)TPLLASGAPRYQHVGQVLLF
lau Shrew        TPEVKEGYLGYTVTWLFFRG(L)PSMLAAGAPRHQHVGQVLLF
xen Sloth        TQEARAGYLGYTVTWLPSQG(R)MSLLAAGAPRYQHVGRVLLF
mar Opossum      NQEAEDGYLGYTVAWLLSKG(Q)RKLLAAGAPRYQHVGRVLLF
pro Platypus     NSSMEDGYLGYTVAWLPQGT(R)RMLLAAGAPRYQHVGQVQLF

NO 113
GN ITGB1
IP IPI00217563.4
PE GTAEKLKPEDITQ
MP 107
EX Kim
CL simians
DE integrin, beta 1 (fibronectin receptor, beta polypeptide, antigen CD29 includes MDF2, MSK12)
SQ
pri Human        KDIKKNKNVTNRSKGTAEKL(K)PEDITQIQPQQLVLRLRSGE
pri Chimpanzee   KDIKKNKNVTNRSKGTAEKL(K)PEDITQIQPQQLVLRLRSGE
pri Gorilla      KDIKKNKNVTNRSKGTAEKL(K)PEDITQIQPQQLVLRLRSGE
pri Orangutan    KDIKKNKNVTNRSKGTAEKL(K)PEDITQIQPQQLVLRLRSGE
pri Rhesus       KDIKKNKNVTNRSKGTAEKL(K)PEDITQIQPQQLVLRLRSGE
pri Baboon       KDIKKNKNVTNRSKGTAEKL(K)PEDITQIQPQQLVLRLRSGE
pri Marmoset     KDIKKNKNVTNRSKGTAEKL(K)PEDITQIQPQQLVLRLRSGE
pri Tarsier      NDIKKNKNVTNRSKGTAEKL(Q)PEDITQIQPQQLVLQLRSGE
pri Bushbaby     KDIKKNKNVTNRSKGTAEKL(Q)PEDITQIQPQQLLLQLRSGE
eua Treeshrew    KHIKKNKNVTNRSKGTAEKL(Q)PEDITQIQPQQLVLQLRSGE
eua Mouse        QTIKKNKNVTNRSKGMAEKL(R)PEDITQIQPQQLLLKLRSGE
eua Rat          QTIKKNKNVTNRSKGMAEKL(R)PEDITQIQPQQLLLKLRSGE
eua Guinea_pig   KNVKKNKNVTDRKKGAAGKL(N)PEDITQIQPQQLVLQLRSGA
eua Squirrel     KTIKKNKNVTNRSKGTAEKL(Q)PEDITQIQPQQLVLQLRSGE
eua Rabbit       RDIKKNKNVTNRSKGTAEKL(Q)PEDITQIQPQQLVLQLRSGE
eua Pika         TAVKKNKNVTNRSKGTAEKL(Q)PEDITQIQPQQLVLQLRSGE
lau Dog          KDIKKNKNVTNRSKGTAEKL(Q)PEDITQIQPQQLVLQLRSGE
lau Cat          KDVKKNKNVTNRSKGTAEKL(Q)PEDITQIQPQQLVLQLRSGE
lau Horse        KDIKKNKNVTNRSKGTAEKL(Q)PEDITQIQPQQLVLQLRSGE
lau Cow          KDIKKNKNVTNRSKGTAEKL(Q)PEDITQIQPQQLVLQLRSGE
lau Dolphin      KDIKKNKNVTNRSKGTAEKL(Q)PEDITQIQPQQLVLQLRSGE
lau Alpaca       KDIKKNKNVTNRSKGTAEKL(Q)PEDITQIQPQQLVLQLRSGE
lau Megabat      KDIKKNKNVTTRSKGTAEKL(Q)PEDIPQIQPQQLVLQLRSGE
lau Microbat     KDIKKNKNVTNRSKGTAEKL(K)PEDITQIQPQQLVLQLRSGE
lau Hedgehog     KAIKKNKNVTNRSKGTAEKL(Q)PEDITQIQPQQLVLQLRSGE
afr Elephant     KNIKKNKNVTNRSKGTAEKL(Q)PEDITQIQPQQLVLQLRSGE
afr Rock_hyrax   KDIKKNKNVTNRSKGTAEKL(Q)PEDITQIQPQQLVLQLRSGE
afr Tenrec       KAIKKNKNVTNRSKGTAETL(Q)PEDITQIQPQQLVLQLRSGE
xen Sloth        KDIKKNKNVTNRSKGTAEKL(Q)PEDITQIQPQQLVLQLRSGE
mar Opossum      KDIRKNKNVTNRSKDTAEKL(Q)PEDITQIQPQQLTLKLRSGE
mar Wallaby      KYIQKNKNVTNRSKGTAEKL(Q)PEDITQIQPQQLTLKLRSGE
pro Platypus     KNIRKNKNVTNRSKGTAEKL(Q)PEDITQIQPQQLVLKLRSGE

NO 114
GN JMJD4
IP IPI00011132.2
PE AFSPQPKELLQQL
MP 447
EX Kim, Wagner
CL primates
DE jumonji domain containing 4
SQ
pri Human        SLVAHPDFQRVDTSAFSPQP(K)ELLQQLREAVDAAAAP*
pri Chimpanzee   SLVAHPDFQRVDTSAFSPQP(K)ELLQQLREAVDAAAAP*
pri Gorilla      SLVAHXDFQRVDTSAFSPRP(K)ELLQQLREAVDAAAAP*
pri Orangutan    SLVVHPDFQRVDTSAFSPQP(K)ELLQRLREAVYAAAAP*
pri Gibbon       SLVAHPDFQRVDTSAFSPQP(K)ELLQQLRAAVDVAADP*
pri Rhesus       SLVAHPSFQRVDTSAFSLRP(K)ELLQQLREAVDAAVAP*
pri Baboon       SLVAHPNFQRVDTSAFSLRP(K)ELLQQLREAVDAAVAP*
pri Marmoset     SLVAHPDFQRVDTSMFSPQP(K)ELLQQLRKAVAATSAP*
pri Mouse_lemur  SVVEHPDFQRVDTSMFSPQP(K)ELLQQLKEAIAVTSVL*
eua Treeshrew    SVIEHPDFQRVDTSAFSPQP(E)ELLRQLEEVVTATTAL*
eua Mouse        SVVVNPDFQRVDTSAFSPQP(E)ELLQQLEDAVAAAEAL*
eua Rat          SVVVNPDFQRVDTSVFSPQP(E)ELLQQLEDSVTAAEAL*
eua Kangaroo_rat SLITHPDFQRIDTSTFSLQP(E)ELLAQLKDAMSSTAAL*
eua Squirrel     SVVIHPDFQRVDTSTFSPGP(E)ELLCQLQDAVAATTTL*
eua Rabbit       SVIAHPDFQRVDTGAFSPQP(E)ELLRRLEEAIAAAADL*
eua Pika         SVIAHPDFQRVDTGAFSPQP(L)ELLRRLEEAMAAIAEL*
lau Dog          SVVAHPDFQRLDTSMFSLQP(E)ELLQQLEEVMVTTASL*
lau Cat          SVVAHPDFQKVDTSMFSPQP(E)ELLRQLEEVVVTTTTL*
lau Horse        SVVAHPDFQRVDTSTFSPRP(E)ELLQQLEEVIAATVSL*
lau Cow          SVVAHPDFQRVDPSTFSPRP(E)VLLRQLEEAVAATTSL*
lau Dolphin      SVVAHPDFQRVDTSAFSPRP(E)ELLQQKEEAVATTTSL*
lau Alpaca       CVVAHRDFQRVDTSAFSPQP(E)ELLQQLEEVVAATVSL*
lau Megabat      SVVVHPDFQRLDTSEFSPQP(E)DLLQQLEEVVAATTSL*
lau Hedgehog     SVVVHPDFQRVDTSACSPRP(E)ELLQQLEEAVAIATSL*
lau Shrew        SLVTHPDFQRVDTSAFSPQP(E)QLLQQLEQAVASTASL*
afr Elephant     SLVANRDFQRVDTSTFSLQP(E)ELLQQLEEVVTATAAL*
afr Rock_hyrax   SLIASPDFQRIDTSTFSLQP(Q)ELLEQLKEAVTATVSL*
afr Tenrec       SLIANPDFQRVDTSTFSLQP(E)ELLRQLEEAITAVASL*

NO 115
GN KHNYN
IP IPI00829596.1
PE GFAEHGKQQQGRE
MP 615
EX Kim
CL simians
DE KH and NYN domain containing
SQ
pri Human        ARTQGSSKAQHPSRGFAEHG(K)QQQGREEEKGSGGIRKTRET
pri Chimpanzee   ARTQGSSKAQHPSRGFAEHG(K)QQQGREEEKGSGGIRKTRET
pri Gorilla      ARTQGSSKAQHPSRGFAEHG(K)QQQGREEEKGSGGIRKTRET
pri Orangutan    ARTQGSSKAQHPSRGFAEHG(K)QQQGREEEKGSGGIRKTRET
pri Gibbon       ARTQGSSKAQHPSRGFADHG(K)QQQGREEEKGSGGIRKTRET
pri Rhesus       ARTQGSSKAQHPSRGFAEHG(K)QQHGREEEKGSGGIRKTRET
pri Baboon       ARTQGSSKAQHPSRGFAEHG(K)QQHGREEEKGSGGIRKTRET
pri Marmoset     ARTQGPSKTQHPSRGFAEHG(K)QQQGREEEKGSGGIRKTRET
pri Tarsier      XXTPGFSKAQPPSRGFTEHD(N)KQQGREEEKGSGGIRKTRET
pri Mouse_lemur  ARTQESSKAHSPSRGFTEHG(N)QQQGREEEKGSGGIRKNRET
eua Treeshrew    ARAKGSSKAQHPSKVFKEHG(N)QQQGREEEKGSVGIRKTRET
eua Mouse        VRKQGSSKTQQPSKGSTEQA(N)QQQGKDADRSNGGIRKTRET
eua Rat          VRKQGSSKSQQPSKGPTEHG(H)QQQGKEVEKGSGGIRKTRET
eua Guinea_pig   ARKQVSSKAQPTSKGFSGHS(N)QQQGKEEEKGSGGIRKTRET
eua Rabbit       ARTQGSSKAQHPPKGVAGHN(N)KQQGKEEEQGSSGIRKTRET
eua Pika         ARAQEPSEAQHPSRGFAGHG(S)KQQEKEEEKGSSCIRKSRET
lau Dog          VRAQGASKAQHPSRGFTDHG(Q)QQQGREEEKGSGGIRKTRET
lau Cat          VRAQGSSKAQHPSRAFTEHG(H)QQQGREEEKGGGGIRKTRET
lau Horse        ARAQGSSKAQHPSRGFAEHG(N)QQQGRKEEKGSSGIRKTRET
lau Cow          VRAPGSSKPQQSARGVTEHS(N)QQQGRKEEKGNGGIRKTRET
lau Dolphin      VRAQGSSKAQHSAKGFTEHS(N)QQQGRKEEKGSGGIRKTRET
lau Megabat      VRAQASSKAQHPSRGLVEHG(N)QQQGREEEKGSSGIRKTRET
lau Microbat     VRAQESSKAQHPSRGLTEHS(Q)QQQGREEEKGSGGIRKTRET
lau Hedgehog     VRSEGPSKAQPLTRALTEHG(N)QPQG--EEKGSGGIRKTRET
lau Shrew        VRTPQSSKAQNPARGLPEPG(P)QRPGEEK----VGIRKNRET
afr Elephant     VRTQGSSEAQHPSRGITKHN(H)QQQGREEEKGSSGIRKTRET
afr Tenrec       ARPQEPPEAQPPPRGIANPG(S)LPQGKEEEKGGGGIRKTRET
xen Armadillo    XXTQESSEAQHLPRGFAEHG(K)EQQEREEEKGSGGIRKTRET
xen Sloth        XXTQGSSEAQHPSRGFAEHG(K)DQQEREEEKGSGGIRKTRET

NO 116
GN KIAA0753
IP IPI00006499.8
PE DGRSDPKVLQTQN
MP 26
EX Kim, Wagner
CL African great apes
DE KIAA0753
SQ
pri Human        PASTCVHLAPRTQLDGRSDP(K)VLQTQNQLQFNRNVPTHSSN
pri Chimpanzee   PASTCVHLAPRTQLDGRSDP(K)VLQTQNQLQFNRNVPTHSSN
pri Gorilla      PASTCVHLAPRTQLDGRSDP(K)VLQTQNQLQFNRNVPTHSSN
pri Orangutan    PASTCVHLAPRTQLDGRSDP(T)VLQTQNQLQFNRNVPTHSSN
pri Gibbon       PASTCVHLAPRTQLDGKSDP(Q)VLQTQNQLQFNRNVATHSSN
pri Rhesus       PASTCVHLAPRTQLDGRSDP(T)VLQTQNQLQFNRNVPTHSSN
pri Baboon       PASTCVHLAPRTQLDGRSDP(T)VLQTQNQLQFNRNVPTHSSN
pri Marmoset     PASTCVHLASRAQLDGRSDP(T)VLQTQNQLQFNRNVPTHSSN
pri Mouse_lemur  PASTCVHLASSAQLDGRSDL(M)KLQTQNQLQFNRNVPTHSSN
eua Mouse        PASTCVHLAPSLQLDAMSDP(R)NLQPQNQLQFNRNVPTNPSN
eua Rat          PASTCVHLASSLQLDGMTDP(R)NLQPQNQLQFNRNVPTDPSN
eua Guinea_pig   PASTCVHVASCTQEGGTNDP(R)KLQTQNQLQFNRSVPAHPSN
eua Rabbit       PASAYVHLASGTQLDGSGDP(R)KPPTQTQLQFNRDVPTHSSN
lau Dog          PASACAHLVPSIQVDGSTEP(H)MRQTQNQLQFNRSVPTHPSN
lau Horse        PASTCVHPASGDQLDERSDP(R)KLQTQNQLQFNRNVPTHPSN
lau Cow          PASTCVHLASGVQLDGRSDP(R)KLHTQNQLQFNRNVPTHSSN
lau Dolphin      PASTCVHLASSVQLDGRSDP(R)KLQTQNQLQFNRNVPTHSSN
lau Megabat      PASTCVHLASTVQLHGRSDP(M)RLQTQNQLQFNKNIPTHSSN
lau Microbat     PASTCVNHASSVQLDGRNDP(R)RLQTQNQLQFNKNVPTHSSN
afr Elephant     PVPACVRLAPDVHLGGRCDP(R)EFQTQNQLQFNRNVPTHSSS
afr Rock_hyrax   PAPSCVHLASDVQPDGRYDP(R)KLQIQNQLQFNRNVPTHSSN
afr Tenrec       PVPPCVHLTSDAQLDGSCDS(R)KYHTQHQLQFNRDVPTHPSN
xen Armadillo    AASTCVHLATDAQPDGRIYP(R)ELQTQNQLQFNRNVPTHSSN

NO 117
GN KIAA0753
IP IPI00006499.8
PE SSVNTAKAQPAQE
MP 718
EX Kim
CL African great apes
DE KIAA0753
SQ
pri Human        VNSTTEANIHLKDGSSVNTA(K)AQPAQEVAAVDFESNNIRQL
pri Chimpanzee   VNSTTEANIHLKDGSSVNTA(K)AQPAQEVAAVDFESNNIRQL
pri Gorilla      VNSTTEANIHLKDGSSVNTA(K)AQPAQEVAAVDFESNNIRQL
pri Orangutan    INSTTEANIHLKDASSVNTV(T)AQPAQEVAAVDFESNNIRQL
pri Gibbon       VNSTAEANIHLKDGSSVNTA(T)AQPAQEVAAVDFESNNIRQL
pri Rhesus       VNSATEAHTHLKDGSSVNTA(T)AQPAQEVAAVDFESNNIRRL
pri Baboon       VNSATEAHTHLKDGSSVNTA(T)AQPAQEVAAVDFESNNIRRL
pri Tarsier      VNSSTEANAHLKDGLSVNTT(A)AQPAYEATAVDSESNNIRQL
pri Mouse_lemur  VNSSTEANNHLKDGPSVNTA(T)AQPAWEATAVDSKSNNVHQL
eua Treeshrew    ASSSAEANTHLKDCPSASTV(P)ALPTEEATAVASESSNNHQL
eua Mouse        VNSSVEANSHLKDRPSRHAA(A)AQPAEQASDVRFESRNIPQL
eua Rat          VNSSVEVNGHLKDLPSRSRA(A)AQPAVQASDVHFESRNICQL
eua Kangaroo_rat VNSSTEANTHLKDRPSLNEA(A)AQPAEQVTAVNSQSSNIHQL
eua Squirrel     VNSSTEANIHLKDRPSFNTA(A)AQPAEQATAVNSESINIHQL
eua Rabbit       DSSSVKANTQLEDYPSADPT(T)AQPAEEATAVDSGANNIHQL
eua Pika         AGSSEEATAQLKDYLAGDTV(T)AQPAEEATALDSEGNNVHQL
lau Cat          VNSSLEAEVP-KDRPPADTA(T)ARPTDEAVAADGGPGSLRQQ
lau Horse        VNSSLEADTHLKDQPSVDAA(T)AQPAEEAAAVDSEPSSNHLL
lau Cow          VNSSLEVDTHLKDQPSEIAT(T)AQPAEEATAVDGESSNIHQL
lau Dolphin      VNSSVEADTRLKDRLSVIAA(T)AQPAEKATAVDCESSSIQQL
lau Alpaca       VNSSLEADTRLKDRPSGSTV(P)AQSAEEVTDADGGSNNLCQL
lau Megabat      VNSSLEADTHLKGRPSVNTE(T)AQPAEEATAVNCEFNNIHQL
lau Microbat     VNSSVGADTHLKDHPSVNTG(T)AQPAEEATALDCKSNNNPQL
afr Elephant     VNSSLHANSRLKDRPSVNTA(A)SQPTEETAAVDSESSNIRLP
afr Rock_hyrax   VSSSQNANNHLKDCPSVNTV(A)AQPARETAAVDSESSNVCLL
afr Tenrec       VRSSLDTNTHFENRPSGSTA(A)GQPAEEPLPLGSDSSHVRLL

NO 118
GN KIAA1731
IP IPI00400986.6
PE SGTIASKERTLSS
MP 435
EX Kim
CL humans
DE KIAA1731
SQ
pri Human        TV-SEIESKAPTVESGTIAS(K)ERTL-SSGQEQVVESDTLTI
pri Chimpanzee   TV-SEIESKAPTVESGTIAS(E)ERTL-SSGQEQVVESDTLTI
pri Gorilla      TV-SEIESKAPTVESGTIAS(E)ERTL-SSGQEQVVESDTLTI
pri Orangutan    TV-SEIESKAPTVESGAIAS(E)ERTL-SSGQEQVVESDTLTI
pri Gibbon       TI-SEIESKVPTVESGTIAS(E)ERTL-SSGQEQVVESDTLTI
pri Rhesus       TV-SEIESKAPTVESGTIAS(E)ERTL-SSGQEQVVESDTLTI
pri Baboon       TV-SEIESKAPTVESGTIAS(E)ERTL-SSGQEQVVESDTLTI
pri Marmoset     TV-SEIESKAPTIESGTLAS(E)ERML-SSGQEQVVESDTLTI
pri Tarsier      TT-GDIESKAPTVESGTITS(E)ERTS-SSGQEQVAESDTLTI
eua Mouse        SI-IEIESKVPSVDSGAIIT(E)ERTAASFEQEQVTDSDRLTI
eua Rat          SIISEIERKVPSTDSGTITT(G)ETAV-SFEQEQVMGSDRLMI
eua Guinea_pig   NV-SEAESKVPTVESGTIAS(E)ERTL-STEQEHVMESDLLTI
lau Dog          TV-SETESKAPTVESGATFG(E)ERTL-SSVQEQVAESDTLTV
lau Horse        TV-SETESKAPMVESGATAA(E)EGAL-SSGQEQVVESDSLTV
lau Cow          TV-SEAESKALTVESGATVS(E)DRTL-SSGQEQVVESDMLTI
lau Dolphin      TV-SETESKALTVESGAAVS(E)DRPL-SSGQKQDVESDTLTV
lau Alpaca       TV-SETESKAVTLESGATVS(E)DRTL-SSEQEQVVESDTLTV
lau Megabat      TV-SGTESEAPTAKSGTTAV(E)ERTL-SSGQEQXXESDTITV
afr Elephant     TV-SEAESKALTMESGTIAS(E)EQAL-SAGQEQVLESDAQTV
afr Rock_hyrax   AV-SEAESKAPTMESGTIAS(E)EQAA-PPGQEQVLESDAQTI
afr Tenrec       AT-SEARSTAPTVESGTVTS(E)ELAL-PPGQQPXXESDTLTI
mar Opossum      IF-ADGESETLTVETGTIAS(E)EKPL-SHKQEQDGESETLTV

NO 119
GN KIAA1731
IP IPI00400986.6
PE LQEQLTKQRDTLQ
MP 1066
EX Kim
CL simians
DE KIAA1731
SQ
pri Human        FLQQFLPLHDSLKLLQEQLT(K)QRDTLQARHEAQVELLLHRQ
pri Chimpanzee   FLQQFLPLHDSLKLLQEQLT(K)QRDTLQARHEAQVELLLHRQ
pri Gorilla      FLQQFLPLHDSLKLLQEQLT(K)QRDTLQARHEAQVELLLHRQ
pri Orangutan    FLQQFLPLHDSLKLLQEQLT(K)QRDTLQARHEAQVELLLHRQ
pri Gibbon       FLQQFLPLHDSLKLLQEQLT(K)QRDTLQARHEAQVE--LHRQ
pri Rhesus       FLQQFLPLHDSLKLLQEQLT(K)QRDTLQARHEAQVELLLHRQ
pri Baboon       FLQQFLPLHDSLKLLQEQLT(K)QRDTLQARHEAQVELLLHRQ
pri Marmoset     FLQQFLPLHDSLKLLQEQLT(K)QRDTLQAQPEAQVALLLHRQ
pri Tarsier      FLQQFLPLHDSLKLLREQLT(T)QRDALQARHEAQMQLLLHRQ
pri Bushbaby     FLQQFLPPHGSLKLLQEQLT(T)QRDTLQARHGTQAELLLHRQ
pri Mouse_lemur  FLQQFLPLHESLKSLQEQMT(T)QRDTLQARPGAQPELLLHRQ
eua Treeshrew    FLEQFLPLRDSPKLLQEQLI(A)QRDTLQARHEAQVASLLYRQ
eua Mouse        FLQQTLPLQNTLKLLQEQLT(R)QRSMIPPRRDGQETLLLYKE
eua Rat          FLQQTLPLQNTLKLLQEQLT(I)QRGMIQPRLNAQETLLLHKE
eua Guinea_pig   FLQQFLPXHDNLKLLQGQVT(P)ETNTLPARCDTQVELLSHGQ
eua Squirrel     FLQQFRPLHDSLKLLQEQLT(T)QRDTLQARHDAQMDLLFHRQ
eua Rabbit       FLQQFLPLHDSLKLLKEQLT(A)QRDALQARHEAQMELLLHRQ
eua Pika         LPRQFLPLHDSLKLLKEQLV(T)QRDALQARHEAHMKLLAGRQ
lau Dog          FLQHFLPLHDSLKLLQEQLT(T)QRDALQASHEPKTELLLHRQ
lau Horse        FLQQFLPLHNSLKLLQEQLT(T)HRDALQARHEAQAELLLRRQ
lau Cow          FPQQFQPLHDCLKLLQEQLI(T)QRDALQARHEAQVELLSRKQ
lau Dolphin      FLQQFHPLHDSLKLLQEQLT(A)QRDALQARHEAQAELLLHRQ
lau Alpaca       FLQQFLPLHDSLKLLQEQLT(T)QRDALQARHGAQAELLLRRQ
lau Microbat     FLQQFLPLHNSLKLLQEQLT(A)QREALQARHEAQVELLSHRQ
lau Hedgehog     FLQQFLPLHNSLKLLQEQLA(T)QRDALQAHHEAQVQLLSRGR
lau Shrew        ILNQFLPLHNCLKLLQAQLA(T)QWEALAARHDAQAQLLARRP
afr Elephant     FLQHFLPLHDSLKLLQEQLT(V)QRDALQARHDAQAALLLHRQ
afr Rock_hyrax   FLQQFLPLHDSLKLLQEQLT(T)QRAALQARHEAQAALLLRRQ
afr Tenrec       FLRGFLPQQDCFKLLQGQLT(V)QRDALQARHETQTDVLVHRE
xen Armadillo    FHQQFLPLHESLHLLQEPLI(A)LQDTLQARHEAQVELLLYGQ

NO 120
GN KIF20B
IP IPI00827503
PE TQNQRIKELINII
MP 767
EX Wagner
CL apes
DE kinesin family member 20B
SQ
pri Human        SLIQELETSNKKIITQNQRI(K)ELINIIDQKEDTINEFQNLK
pri Chimpanzee   SLIQELETSNKKIITQNQRI(K)ELINIIDQKEDTINEFQNLK
pri Gorilla      SLIQELETSNKKIITQNQRI(K)ELINIIDQKEDTINKFQNLK
pri Orangutan    SLIQELETSNKKIITQNQRI(K)ELINIIDQKEDTVNEFQNLK
pri Gibbon       SLIQELETSNKKIITQNQRI(K)ELINIIDQKEDTINEFQNLK
pri Rhesus       SLIQELETSNKKIITQNQRI(Q)ELINTIDQKEGIINEFQNLK
pri Baboon       SLIQELETSNKKIITQNQRI(Q)ELINTIDQKEGIINEFQNLK
pri Marmoset     SLIQELETSNKKIITQNQRI(Q)ELINIIDQKEDTINKFQNLK
pri Bushbaby     XXXXXXXXXXXKIIMQNQRI(Q)ELIDIINQKEDTISKFQNLK
pri Mouse_lemur  XXXXXXXXXXXKIIMQNQRI(Q)ELIDIIDQKEDTINKFQNLK
eua Treeshrew    XXXXXXXXXXXKIIMQNQRI(Q)ELADIIDQKGDAINTFQNLN
lau Cat          SLVQELEKSNKTILMQNQKI(Q)ELGDMIAQKEDTINKFQNLK
lau Horse        SLVQELEKSKKKIIMQNQRI(Q)DLIDITDQKENTINKFQNLK
lau Cow          SLVQELEKSNKKIIMQNQRI(Q)ELMDKIDQKEDTINKFQNLK
lau Alpaca       SLVQELEKSNKKIVVQNQRI(Q)ELMDKIDQKEDTINKFQNPK
lau Megabat      SLVEELKKSNEKIIMHNQRI(Q)ELKDMIDQKEDTIN-FQNLK
xen Armadillo    SLVQELEQSNKKIIMQNERM(Q)ELIDIIDQKEDTINKYQNLK
mar Opossum      ISVHELENANKKIVAQNQRI(Q)ELMNIIDQKEETINKLQNLV
mar Wallaby      ISVHELENANKKIVAQDQRI(Q)ELMDIIDQKEDTISKLQNLV

NO 121
GN KIF23
IP IPI00873577
PE TYNTPLKVTSIAR
MP 741
EX Wagner
CL catarrhines
DE kinesin family member 23
SQ
pri Human        SVASCISEWEQKIPTYNTPL(K)VTSIARRRQQEPGQSKTCIV
pri Chimpanzee   SVASCISEWEQKIPTYNTPL(K)VTSIARRRQQEPGQSKTCIV
pri Gorilla      SVASCISEWEQKIPTYNTPL(K)VTSIARRRQQEPGQSKTCIV
pri Orangutan    SVASCISEWEQKIPTYNTPL(K)VTSIARRRQQEPGQSKTCIV
pri Gibbon       SVASCISEWEQKMPTYNTPL(K)VTSIARRRQQEPGQSKTCIV
pri Rhesus       SVASCISEWEQKIPTYNTPL(K)VTSIARRRQQEPGQSKTCIV
pri Baboon       SVASCISEWEQKIPTYNTPL(K)VTSIAKRRQQEPGQSKTCIV
pri Marmoset     SVASCISEWEQKIPTCNTPL(R)DTSVARRRQQEPGQSKACIV
pri Tarsier      SVASCISXWEQKLPTYNTPI(N)VTSLARCRQQEPGQSKTCMV
pri Bushbaby     SVASCISEWEQKIPSYNTPL(N)VTSIARRRQQEPEQSKTCIV
pri Mouse_lemur  SVASCISEWEQKIPSYNTPL(N)VTSIARRRQQEPGQSKTCIV
eua Treeshrew    SVASCVSEWEQKMPSYNTPL(N)VTSLARRRQQEPGQSKTCVV
eua Mouse        SVASCISEWEQKLSPFSTPV(N)VTSLARHRQQEPGQSKTCIV
eua Rat          SVASCISEWEQKLSPFSTPV(N)VTSLARHRQQEPGQSKACMV
eua Kangaroo_rat SVASCISEWEQKVPSYNTPG(N)VTSIARRRQQEPGQSKSCIV
eua Guinea_pig   SVASCVSEWEQKIPTFGTPG(N)VTSIVRRRQQEPEQSKTCLM
eua Rabbit       SVASCISEWEQKIPPYNTPL(N)VTSVARRRQREPGQSQTCIV
eua Pika         SVASCISEWEQKIPPYNTPV(N)VTSIARRRQREQGQSKTCIV
lau Dog          SVASCISEWEQKIPPYNTPV(N)VTSIARRRQQEPGQSKTCVV
lau Cat          SVASCISEWEQKIPPYNTPL(N)VTSVARRRQQEPGQSKTCVV
lau Horse        SVASCVSEWEQKIPPYNTPL(T)VTSIARRRQQEPGQSKTCIV
lau Cow          SVASCISEWEQKIPPYNTPL(N)VTSIARRRQQEPGQSKTCIV
lau Dolphin      SVASCISEWEQKIPSYNTPL(N)VTSIARRRQQEPGQSKTCIV
lau Alpaca       SVASCVSEWEQKMPPYSSPL(G)ATSIARRRQQEPGQSKACVV
lau Megabat      SVASCISEWEQKIPPYNTPL(N)VTSIARRRQQEPGQSKTCIV
lau Microbat     SVASCISEWEQKIPSYNTPP(N)VTSIARRRQQEPGQSKTCIV
lau Shrew        SVASCISEWEQKIPSYNTPV(S)VTSIARRRQQEPGQSKTCMV
afr Elephant     SVASCISEWEQKIPPYNSPL(N)VTSIARRRQQEPGQSKTCIV
afr Tenrec       SVASCVSEWEQKIPPYNTPV(S)VTSIARRRQQEPEQSKTCVV
xen Armadillo    SVASCISDWEQKISPYNTPL(N)VTSIARRRQQEPGQSKTCIV
xen Sloth        SVASCISEWEQKIPPYNTPL(N)VTSIARRRQQEPGQSKTCVV
mar Opossum      SVASCVSEWEQKIPPYNTPQ(N)VTSLARRRQQEPGQNKNCIV
pro Platypus     SVASSVSEWEQKIPPFRTPS(H)APSFPRHRPQEPEPKKSCVV

NO 122
GN LAMB3
IP IPI00299404.1
PE GGTGSPKLVALRL
MP 766
EX Kim
CL apes
DE laminin, beta 3
SQ
pri Human        RREAERLVRQAGGGGGTGSP(K)LVALRLEMSSLPDLTPTFNK
pri Chimpanzee   RREAERLVRQAGGGGGTSSP(K)LVALRLEMSSLPDLTPTFNK
pri Gorilla      RREAERLVRQAGGGGGTGSP(K)LVALRLEMSSLPDLTPTFNK
pri Orangutan    RREAERLVQQAGGGGGTGSP(K)LVALRLEMSSLPDLTPTFNK
pri Gibbon       RREAERLVRQAGGGGGAGSP(K)LVALRLEMSSLPDLTPTFNK
pri Baboon       RREAERLAQQAGGGGGTGSP(Q)LVALRLEMSSLPDLTPTFNK
pri Marmoset     RREAERLQQQAGEGGGAGGP(Q)LEALRLEMSSLPDLTPTFNK
pri Mouse_lemur  RREAERLELQ-GGGGGAGGP(Q)LAALRLEMASLPDLTPTINK
eua Treeshrew    RREAERLEGQMTGGGGASSP(Q)LAALRLEMATWPDLTPTINK
eua Mouse        RREAEGLERQ-AGGGGTGGA(Q)LMALRLEMASLPDLTPTINK
eua Rat          RREVEGLERQ-TGEGGAGGA(Q)LMALRLEMASLPDLTPTINK
eua Kangaroo_rat RREAEDPERQVGAGGGAGGP(Q)LEALRLEMASLPDLTPTINK
eua Guinea_pig   RKEAEGLMRQMQGGGAAGAP(E)LAALRAEMASLPDLTSTINK
eua Squirrel     RRRAEGLDRLVGGEG--DGS(H)LASLQLEMASLPDLTPTINK
eua Rabbit       RREAEGLEWQAGTGGGASSP(Q)LAALRLEMAALPDLTPTINK
eua Pika         RRETERLEWQAGSGGAASSP(Q)LEALRLQVAALPDLTPTINK
lau Dog          RREAERLEGQLGGGAGVGGP(Q)LVALRLEMASLPDLTPTINK
lau Cat          RREAERLEGQVGGGTGAGGP(Q)LAALSLGMASLPDLMPTINK
lau Horse        WREAEGLEQQIGAGGGASGP(Q)LAALRLEMASLPDLTPTINK
lau Cow          RREAERLEQQVAGGPGASGP(Q)LAALRLEMASVPDLTPTINQ
lau Dolphin      RREAERLEQQLGGAGGAGGP(Q)LAALRLQMASLPDLTPAANK
lau Megabat      RREAEGLEQQI--GGAASGP(H)LAALKLGLASLPDLTPTINK
lau Microbat     RREAEKLEQQAGGGGGAGGP(Q)LAALKLEMASLPDLTPTINK
lau Shrew        HREAEALQRQMGGAAGTGGP(Q)LASLQLQIATLPNLTPTINQ
afr Elephant     RREAERLERQLA-GGGASGP(Q)LTALRLQMASFPDLTPTINK
afr Rock_hyrax   WREAEQLERQVG--RGASSP(Q)LAALRLEMVSFPNLTPTINK
afr Tenrec       RREAERLERQVAGGGGASSP(Q)LAALRLEMASFPDLTPTANK
xen Armadillo    RKEAERLEMQGVGERGASGP(Q)LTALKKEMAFFPDLTPTFNK
mar Opossum      RREAERLERQVEGSGSAGGP(Q)LQTLRQQMASFPNLTPTINK
pro Platypus     RREAERLEGQLPSGGGIGDL(G)LEALKREMGSLPDLTPTSNK

NO 123
GN LAMP1
IP IPI00884105.2
PE ARDPAFKAANGSL
MP 319
EX Kim
CL simians
DE lysosomal-associated membrane protein 1
SQ
pri Human        FFLQGIQLNTILPDARDPAF(K)AANGSLRALQATVGNSYKCN
pri Chimpanzee   FFLQGIQLNTTLPDARDPAF(K)AANGSLRALQATVGNSYKCN
pri Gorilla      FFLQGIQLNTILPDARDPAF(K)AANGSLRALQATVGNSYKCN
pri Gibbon       FFLQGIQLNTTLPDARDPAF(K)AANGSLRALQATVGNSYKCN
pri Rhesus       FFLQGIQLNTTLPDARDPAF(K)AANSSLRALQATVGNSYKCN
pri Baboon       FFLQGIQLNTTLPDARDPAF(K)AANSSLRALQATVGNSYKCN
pri Marmoset     FFLQGIQLNTTLPDARDPTF(K)ASNNSLRALQATVGNSYRCN
pri Bushbaby     FFLQDIQLSMILADARDPHF(T)ASNGSLMALQATLGNSYKCN
pri Mouse_lemur  FFLKGIQVNMTLPDARDPAF(R)AANESLRALQATVGNSYKCN
eua Mouse        FFLQGVRLNMTLPDALVPTF(S)ISNHSLKALQATVGNSYKCN
eua Rat          FFLQGVQLNMTLPDAIEPTF(S)TSNYSLKALQASVGNSYKCN
eua Guinea_pig   FFLHEVQLNMTVPDARGPSF(S)AANSSLRALQATVGNSYKCS
eua Squirrel     FFLQGIQLNATFPDAKEPTF(R)ATNTSLRALQATVGHSYKCN
eua Rabbit       YFLQGVLLNTTLPDAREPAF(S)ASNSSLRALQATLGNSYKCN
eua Pika         YFLQGVKLNTVLPDAREPAF(A)ATNNSLRALQATLGNSYKCN
lau Dog          FFLQGIQLNMTLPDARDPTF(K)AGNNSLRALQATIGNSYKCN
lau Cat          FFLQGIQLNMTVPDARDPTF(Q)ADNSSLRALQATIGNSYKCN
lau Horse        FFLQEIQLDMTLPDARDPTF(K)AANSSLRALQATIGNSYRCN
lau Cow          VFLQGVQLNLTLPDAKEGSF(T)ATNSSLRALQATAGNSYKCN
lau Dolphin      VFLQGVQLNMTLPDAREPTF(W)ATNSSLRALQAAAGNSYKCN
lau Megabat      FFLQEIQLNTTLPDARDPNF(R)AANSSLRALQATMGNSYKCN
lau Microbat     FFLKEIQLNMTLPDAKVPTF(Q)ASNSSLRALQATMGNSYKCN
lau Shrew        FFLQGVQLNMTLPQAKEPTF(R)AANTSLRALEASVGNSYKCN
afr Elephant     FFLQEIQLNTTFPDAKDPSF(K)AINDSLRALQATVGNSYKCN
afr Rock_hyrax   FFLQEIQLNTTLPDAREPTF(R)AANDSLRALEATIGNSYKCN
xen Armadillo    FFLQGIQLNTMLPDAREPTF(K)AGNDSLRALQATIGNSYKCN
xen Sloth        FFLQGIQLNTTLPDARDPTF(K)ASNDSLRALQATIGNSYKCN
mar Opossum      FFLREIRFHKFFPDAKDPAF(G)AVNSSLKELQATVGNSYKCN
mar Wallaby      FFLQGIYLNKTFPDAKKPTF(E)AFNNSLKELQASVGNSYKCN
pro Platypus     FFLEGIHLRTAFPDAKEMWF(D)AANTSLKALQATVGNSYRCN

NO 124
GN LAPTM5
IP IPI00013827.1
PE MNSVEEKRNSKML
MP 225
EX Kim
CL apes
DE lysosomal protein transmembrane 5
SQ
pri Human        MFKCVWRCYRLIKCMNSVEE(K)RNSKMLQKVVLPSYEEALSL
pri Chimpanzee   MFKCVWRCYKFIKCMNSVEE(K)RNSKMLQKVVLPSYEEALSL
pri Gorilla      MFKCVWRCYKFIKCMNSVEE(K)RNSKMLQKVVLPSYEEALSL
pri Orangutan    MFKCVWRCYRFIKCMNSVEE(K)RNSKMLQKVVLPSYEEALSL
pri Gibbon       MFKCVWRCYRFIKCMNSVEE(K)RNSKMLQKVVLPSYEEAVSL
pri Rhesus       MFKCVWRCYRFIKCLNSIEE(R)SNSKMFQKVVLPSYEEALSL
pri Baboon       MFKCVWRCYRFIKCLNSIEE(R)SNSKMFQKVVLPSYEEALSL
pri Marmoset     MFKCVWRCYRFIKFLNSAEE(R)SNSKMLRKVVLPSYEEALSL
pri Tarsier      MFKCVWRCYRLVKDMNSAEG(R)ASSKELPKXXXXXXXXXXXX
pri Bushbaby     MLKCVWRCYRFIKYMNSVEE(R)SDSKMLQKVVLPSYEEALAL
pri Mouse_lemur  MFKCVWRCYRFIKHMNSVED(K)SDSKMLDKVVLPSYEEALSL
eua Mouse        MFKCVYTCYKFLKHMNSAME(D)SSSKMFLKVALPSYEEALSL
eua Rat          MFKCVWTCYRFMKHMNSAVE(D)SSSKLFLKVALPSYEEALSL
eua Guinea_pig   MFKCVWRCYQFIKYMNSASE(R)SDSKSPPKVVLPSYEEAISL
eua Squirrel     MLKCVWRCYRLLKSANSAEE(R)GASKTLPKVVLPTYEEALSL
eua Rabbit       MFKCVWRCYRLMKCVNLAER(R)SDSKVPPKVALPTYEEALSL
lau Dog          MFKCVWRCYKLMKYMNSAEE(N)SSSKMLPKVVLPSYEEALSL
lau Cat          MFKCVWRCYKFMKYLNSAEE(R)SGSKMLQKAVLPSYEEAVSL
lau Horse        MFKCVWRCYRFIKCTNSTEE(R)SSSKTPQKVVLPSYEEAVAL
lau Cow          MFKCVWRCYRLMKCTNSAEE(R)SGSKMLQKVVLPSYEEAVSL
lau Dolphin      MFKCVWRCYRFMKYMNSAEE(R)SSSKMLQKVVLPSYEEAVSL
lau Alpaca       MFKCVWKCYRFIRYMNSAEG(R)SGSKMLSKVVLPSYEEAVSL
lau Microbat     MFKCVWRCYKLIKCMNSAEE(K)SSSKMLPKVVLPSYEEAVSL
lau Hedgehog     MFKCVWRCYKLIKYMNLAEE(K)DRTKMLPKVVLPSYEEALSL
afr Elephant     MFKCVWRCYKFVKYMNSAEK(R)NSSKMLQKVVLPSYEEALSL
afr Rock_hyrax   MFKCVWRCYRLIKCMNAAEE(R)SSSKVLPKVVLPSYEEALSL

NO 125
GN LDHA
IP IPI00217966.8
PE PDLGTDKDKEQWK
MP 251
EX Kim
CL catarrhines
DE lactate dehydrogenase A
SQ
pri Human        SGMNVAGVSLKTLHPDLGTD(K)DKEQWKEVHKQVVESAYEVI
pri Chimpanzee   SGMNVAGVSLKTLHPDLGTD(K)DKEQWKEVHKQVVESAYEVI
pri Gorilla      SGMNVAGVSLKTLHPDLGTD(K)DKEQWKEVHKQVVESAYEVI
pri Gibbon       SGMNVAGVSLKTLHPDLGTD(K)DKEQWKEVHKQVVESAYEVI
pri Orangutan    SGMNVAGVSLKTLHPDLGTD(K)DKEQWKEVHKQVVESAYEVI
pri Rhesus       SGMNVAGVSLKTLHPDLGTD(K)DKEQWKEVHKQVVESAYEVI
pri Baboon       SGMNVAGVSLKTLHPDLGTD(K)DKEQWKEVHKQVVESAYEVI
pri Marmoset     SGVNVAGVSLKTLHPELGTD(T)DKEQWKEVHKQVVESAYEVI
pri Tarsier      SGVNVAGVSLKNLHPDLGTD(T)DKEQWKEVHKQVVEXXXXXX
pri Bushbaby     SGVNVAGVSLKNLHPDLGTD(T)DKEQWKEVHKQVVEXXXXXX
eua Mouse        SGVNVAGVSLKSLNPELGTD(A)DKEQWKEVHKQVVDSAYEVI
eua Rat          SGVNVAGVSLKSLNPQLGTD(A)DKEQWKDVHKQVVDSAYEVI
eua Kangaroo_rat SGVNVAGVSFKNLNPDLGTD(A)DKEQWKTVHKQVVDSAYEVI
eua Guinea_pig   SGVNVAGVALKNLHPELGTD(G)DKEHWKEVHKQVVDSAYEVI
eua Rabbit       SGMNVAGVSLKTLHPELGTD(A)DKEQWKQVHKQVVDSAYEVI
eua Pika         SGVNVAGVSLKTLHPEMGTD(T)DKEQWKQVHKQVVDSAYEVI
lau Dog          SGVNVAGVSLKNLHPDLGTD(A)DKEQWKQVHKQVVDSAYEVI
lau Horse        SGVNVAGVSLKNLHPELGTD(A)DKEHWKEVHKQVVDSAYEVI
lau Cow          SGVNVAGVSLKNLHPELGTD(A)DKEQWKAVHKQVVDSAYEVI
lau Dolphin      SGVNVAGVSLKNLHPELGTD(A)DKEHWKAIHKQVVDSAYEVI
lau Alpaca       SGINVAGVSLKNLHPELGTD(A)DKEQWKAVHKQVVDSAYEVI
lau Megabat      SGVNVAGVSLKNLHPDLGTD(T)DKEQWKEVHKQVVASAYEVI
lau Microbat     SGVNVAGVSLKNLHPDLGTD(A)DKEQWKQVHKQVVDSAYEVI
lau Hedgehog     SGVNVAGVSLKNLHPELGTD(S)DKEQWKEVHKQVVDXXXXXX
lau Shrew        SGVNVAGVALKNLHPDLGTD(A)DKEQWKQVHKQVVDSAYEVI
afr Elephant     SGVNVAGVSLKHLHPELGTD(A)DKEQWKEVHKQVVESAYEVI
afr Rock_hyrax   SGVNVAGVSLKNLHPELGTD(A)DKEHWKEVHKQVVDSAYEVI
afr Tenrec       SGVNVAGVSLKNLHPALGTD(G)DKEQWKEVHKQVVESAYEVI
xen Armadillo    SGMNVAGVSLKNLHPELGTD(A)DKEQWKEVHKQVVDXXXXXX
xen Sloth        SGVNVAGVSLKNLHPELGTD(A)DKEHWKEVHKQVVDSAYEVI
mar Opossum      SGVNVAGVSLKSLHPALGTD(S)DSEQWKDVHKQVVESAYEVI
pro Platypus     SGVNVAGVSLKNLHPDLGTD(A)DKEQWKDVHKQVVDSAYEVI

NO 126
GN LMNB2
IP IPI00009771.6
PE EQVRLYKLELEQT
MP 275
EX Kim, Wagner
CL primates
DE lamin B2
SQ
pri Human        KMAQALEELRSQHDEQVRLY(K)LELEQTYQAKLDSAKLSSDQ
pri Chimpanzee   KMAQALEELRSQHDEQVRLY(K)LELEQTYQAKLDSAKLSSDQ
pri Gorilla      KMAQALEELRSQHDEQVRLY(K)LELEQTYQAKLDSAKLSSDQ
pri Orangutan    KMAQALEELRSQHDEQVRLY(K)LELEQTYQAKLDSAKLSSDQ
pri Rhesus       KMAQALEELRSQHDEQVRLY(K)LELEQTYQXXXXXXKLSSDQ
pri Baboon       KMAQALEELRSQHDEQVRLY(K)LELEQTYQAKLDSAKLSSDQ
pri Marmoset     KMAQALEELRSQHDEQVRLY(K)LELEQTYQAKLDSAKLSSDQ
pri Bushbaby     KMAQALEELRSQHDEQVRLY(K)QELEQTYQAKLDNAKLSSDQ
eua Mouse        KMAQALEDLRSQHDEQVRLY(R)VELEQTYQAKLDNAKLLSDQ
eua Rat          KIAQALEELRSQHDEQVRLY(R)LELEQTYQAKLDNTKLISDQ
eua Kangaroo_rat KLARALEELRGQHDQQVQLY(R)QELEQTYQAKLDNAKLSSDQ
eua Guinea_pig   KMAQALEELRSQHDQQVQLY(R)QELEQTYQAKLENARLSSDQ
eua Squirrel     RLAQALEELRSQQDEHVRLY(R)LELEQTYQTKXXXXXXXXXX
eua Pika         KMAKALEELRSQHDEQVRLY(R)LELEQSYQAKLDHAKLSSDQ
lau Dog          RMAQALEELRSQHDEQVRLY(K)LELEQTYQAKLDSAKLSSDQ
lau Horse        KMAQALEELRAQHDEQVRLY(K)LELEQTYQAKLDNAKLISDQ
lau Cow          KMAQALEELRAQHDEQVRLY(R)LELEQTYQAKLDNAKLSSDQ
lau Dolphin      KMAQALEELRAQHDEQVRLY(R)LELEQTYQAKLDNAKLSSDQ
lau Megabat      RMAQALEELRSQHDEQVRLY(R)LELEQTYQAKLDHAKLSSDQ
lau Microbat     KMAQALEELRSQHDEQVRLY(K)LELEQAYQAKLDNAKLCSDQ
afr Elephant     KMAQALEELRSQHDEQVRLY(R)LELEQTYQAKLENAKLSSDQ
afr Rock_hyrax   KMAQALEELRSQHEEQVRLY(R)LELEQTYQAKXXXXXXXXXX
afr Tenrec       KMAQALEELRGQHDEQVRLY(R)LELEQTYQAKLDNAKQSAER
mar Opossum      KMAQALEDLRNQHDEQVRLY(K)LELEQTYQAKLENAKLCSDQ
mar Wallaby      KMAQALEDLRNQHDEQVRLY(K)MELEQTYQAKLENAKLCSDQ
pro Platypus     KMSQALEDLRNQHDEQVKIY(K)IELEQTYQAKLENAKLASDQ

NO 127
GN LPCAT2
IP IPI00016418
PE GIEEFAKYLKLPV
MP 386
EX Wagner
CL catarrhines
DE lysophosphatidylcholine acyltransferase 2
SQ
pri Human        EYASIASSSKGGRIGIEEFA(K)YLKLPVSDVLRQLFALFDRN
pri Chimpanzee   EYASIASSSKGGRIGIEEFA(K)YLKLPVSDVLRQLFALFDRN
pri Gorilla      EYASIASSSKGGRIGIEEFA(K)YLKLPVSDVLRQLFALFDRN
pri Orangutan    EYASIASSSKGGRIGIEEFA(K)YLKLPVSDVLRQLFALFDRN
pri Gibbon       EYASIASSSKGGRIGIEEFA(T)YLKLPVSDVLRQLFALFDRN
pri Rhesus       EYASIASSSKGGRIGIDEFA(K)YLKLPVSDVLRQLFALFDRN
pri Baboon       EYASIASSSKGGRIGIDEFA(K)YLKLPVSDVLRQLFALFDRN
pri Marmoset     EYASIASSSKGGRIGIEEFA(E)YLKLPVSDVLRQLFALFDRN
pri Tarsier      EYASIASSSKGGRIGIEEFA(E)YLKFPVSDVLRQLFALFDRN
pri Bushbaby     EYASIASSSKGGRIGIEEFA(E)YLKLPVSDVLRQLFALFDRN
pri Mouse_lemur  EYASIASSSKGGRIGIEEFA(E)YLKLPVSDVLRQLFALFDRN
eua Mouse        EYASIASSSKGGRIGIEEFA(E)YLKLPVSDVLRQLFALFDRN
eua Rat          EYASIASSAKGGRIGIEEFA(E)YLKLPVSDVLRQLFALFDRN
eua Kangaroo_rat EYASIASSSKGGRIGIEEFA(E)YLKLPVSDVLRQLFALFDRN
eua Guinea_pig   EYASIASASKGGRIGIEEFA(E)YLKLPVSDVLRQLFALFDRN
eua Squirrel     EYASIARSSKGGRIGIEEFA(E)YLKLPVSDVLRQLFALFDRX
eua Rabbit       EYASIASSSKGGRIGIEEFA(E)YLKLPVSDVLRQLFALFDRN
eua Pika         EYASIASSSKGGRIGIEEFA(E)YLKLPISDVLRQLFALFDRN
lau Dog          EYATIASSSKGGRIGIEEFA(E)YLKLPVSDVLRQLFALFDRN
lau Cat          EYATIACCSKGGRIGIDEFA(E)YLKLPVSDVLRQLFTLFDRN
lau Horse        EYATIASSSKGGRIGIEEFA(E)YLKLPVSDVLRQLFALFDRN
lau Cow          EYAAIASSSKGGRIGIEEFA(E)YLKLPVSDVLRQLFALFDRN
lau Dolphin      EYAAIASSSKGGRIGIEEFA(E)YLKLPVSDVLRQLFALFDRN
lau Alpaca       EYATIASSSKGGRIGIEEFA(E)YLKLPVSDVLRQLFALFDRN
lau Megabat      EYAAIASSTKGGRIGIEEFA(E)YLKLPVSDVLRQLFALFDRN
lau Microbat     EYATIASSSKGGRIGIEEFA(E)YLKLPVSDVLRQLFALFDRN
lau Hedgehog     EYAAIASSSKGGRIGIEEFA(E)YLKLPVSDVLKQLFALFDRN
lau Shrew        EYAAIASSSKGGRIGIEEFA(E)YLKLPVSDVLRQLFALFDRN
afr Elephant     EYASIARSSKGGRIGIEEFA(E)YLKLPVSDVLRQLFALFDRN
afr Rock_hyrax   EYASIASSSKGGRIGIEEFA(E)YLKLPVSDVLRQLFALFDRN
afr Tenrec       EYAAIASASKGGRIGIEEFA(E)YLKLPVSDVLKQLFALFDRX
xen Armadillo    EYAAIASSSKGGRIGIEEFA(E)YLKLPVSDVLRQLFALFDRX
xen Sloth        EYAAIASSSKGGRIGIEEFA(E)YLKLPVSDVLRQLFALFDRX
mar Opossum      EYAAIATASKGGRIGIEEFA(E)YLKLPISDVLRQLFALFDRN
pro Platypus     AYAAIASSSKGGRIGIEEFA(D)YLKLPVSDVLKDLFALFDRN

NO 128
GN LRPPRC
IP IPI00783271
PE YFHQLEKMNVKIP
MP 613
EX Wagner
CL apes
DE leucine-rich PPR-motif containing
SQ
pri Human        SDSEVQAKEEHLRQYFHQLE(K)MNVKIPENIYRGIRNLLESY
pri Chimpanzee   SDSEVQAKEEHLRQYFHQLE(K)MNVKIPENIYRGIRNLLESY
pri Gorilla      -DSEVQAKEEHLRQYFHQLE(K)MNVKIPENIYRGIRNLLESY
pri Orangutan    SDSEVQAKEEHFRQYFHQLE(K)MNVKIPENIYRGIRNLLDSY
pri Gibbon       SDSEVQAKEEHLRQYFHQLK(K)MNVKIPENIYRGIRNLLGSY
pri Rhesus       SDSEVQAKEERLRQYFHQLK(E)MNVKIPENICRGIRNLLDSY
pri Baboon       SDSEVQAKEERLRQYFHQLK(E)MNVKIPENICRGIRNLLDSY
pri Marmoset     SDSEVQAKEERLRQYFHQLK(E)MNLKIPENTYKGIRNLLDSY
pri Tarsier      SDSEVQAKEERLRQYFHQLK(E)MNVKIPENIYRGIRNLLDSY
pri Bushbaby     SDSEVQAKEEHLRQYFHQLK(E)MNVKIPENIYRGIRNLLDSY
pri Mouse_lemur  SDSEVQAKEERLRQYFHQLK(E)MNVKIPENIYRGIRNLLDSY
eua Treeshrew    SDSEVQAKEERLRQYFHQLK(E)MNVKIPENIYRGIRNLLDSY
eua Mouse        SDSEVQAKEERLRQYFHQLQ(E)MNVKVPENIYKGICNLLNTY
eua Rat          SDSEVQAKEERLRQYFHQLR(E)MNVKVSENIYKGICNLLDNY
eua Kangaroo_rat SDSEVQAKEEHLRQYFHQLK(E)MNIKISENIYRGIRNLLDSY
eua Guinea_pig   SDSEVQAKEERLRQYFHQLK(E)MNIKISENIFRGIRNLLDSY
eua Squirrel     SDSEVQAKEERLRQYFHQLK(E)MNIKIPESIYRGIRNLLDSY
eua Rabbit       SDSEVQAKEELLRQYFHQLK(E)MNVKIPDNIYRGIRNLLDSY
eua Pika         SDSEVQAKEERLRQYFHQLK(E)MNVKIPENIYKGVRNLLDSY
lau Dog          SDSEVQAKEERLRQYFHQLK(E)MNVKITENLYRGIRNLLDSY
lau Cat          SDSEVQAKEERLRQYFHQLK(E)MNVKIPENIYRGIRNLLDSY
lau Horse        SDSEVQAKEEHLRQYFHQLK(E)MNVKIPENIYRGIRNLLDNY
lau Cow          SDSEVQAKEEHLRQYFHQLK(E)MNVKIPENIYRGIRNLLDSY
lau Dolphin      SDSEVQAKEEHLRQYFHQLM(E)MNVKIPENIYRGIRNLLDSY
lau Alpaca       SDSEVQAKEERLRQYFHQLK(E)MNVKIPENIYRGIRNLLDSY
lau Microbat     SDSEVQAKEERLRQYFHQLK(E)MNVKISENIYRGIRNLLDSY
lau Hedgehog     SDSEVQAKEEHLRQYFHQLK(E)MNINISENIYRGIRNLLDSH
lau Shrew        SDSEVQAEEARLRQYFHQLK(E)MNVKIPENIYRGIRNVLDSR
afr Elephant     SDSEVQAKEECLRQYFHQLK(K)MNITISQNIYRGIRNILDSY
afr Tenrec       SDSEVQAKEEHLRQYFHQLK(E)MNLKIPENIYRGIRNLMDSY
xen Sloth        SDLEVQAKEEHLRQYFHQLK(H)MNIKIPENIYRGIRNLLDSY
mar Opossum      SDSEVQAKEERLRQYFHQLK(K)MNIAIPERIFKGIRNRLNSH
pro Platypus     SDSEVQAKEERLRQYFHQLK(K)MNMVIPENLFKGIRNLLDAH

NO 129
GN LTN1
IP IPI00783835
PE KWNSLLKIIEKAC
MP 763
EX Wagner
CL simians
DE listerin E3 ubiquitin protein ligase 1
SQ
pri Human        ERKKVLDDLTKVDLKWNSLL(K)IIEKACPSSDKHALVTPWLK
pri Chimpanzee   ERKKVLDDLTKVDLKWNSLL(K)IIEKACPSSDKHALVTPWLK
pri Gorilla      ERKKVLDDLTKVDLKWNSLL(K)IIEKACPSSDKHALVTPWLK
pri Orangutan    ERKKVLDDLTKVDLKWNSLL(K)IIEKACSSSDKHALVTPWLK
pri Gibbon       ERKKVLDDLTKVDLKWNSLL(K)IIEKACSSSDKHALVTPWLK
pri Rhesus       ERKKVLDDLTKVDLKWNSLL(K)IIEKACSSSDKHALVTPWLK
pri Baboon       ERKKVLDDLTKVDLKWNSLL(K)IIEKACSSSDKHALVTPWLK
pri Marmoset     ERKKVLDDLTKVDLKWNSLL(K)IIEKACSSSDKHALVTPWLK
pri Mouse_lemur  ERKDVLDDLTEVDLKWKFLL(Q)VIEKXXXXXXXXXXXXXXXX
eua Mouse        ERKEVLDDLTKEDLKWSSLL(Q)VIEKACSSSDKHALVTPWLK
eua Rat          ERKEVLDDLTQEDLKWSSLL(R)VIEKACSSSDKHALVTPWLK
eua Guinea_pig   ERRDVLDDLTKEDLKWDSLL(Q)VIEQACSSSDKHALVALWLK
eua Rabbit       ERKDVLDNLTEEHLQWNSLL(Q)VIEKACSSSNKHALVTPWLK
lau Dog          ERKYVLDDLIEGDLKWSSIL(Q)VIEKACSSSDKHALVTPWLK
lau Cat          ERKYVLDDLIEGNLKWNSIL(Q)VIEKACSSSDKHALVTPWLK
lau Horse        ERKDVLDDLTKVDLNWNSVL(Q)VIEKACSSSDKHALVTPWLK
lau Cow          ERKAILDDLIEVELKWNSIL(Q)IIEKACSSSDNHALVTPWLK
lau Dolphin      ERKAVLDDLIEVDLKWNSIL(Q)VIEKACSSSDKHALVTPWLK
lau Alpaca       ERKAVLDDLTEVDLKWNSIL(Q)VIEKACSSSDKYALVTPWLK
lau Megabat      ERKVVLDDLTKVDLKWNSIL(Q)VIEKACSSSDKHSLVVPWLK
lau Microbat     ERKDILDDLTEVDLKWNSVL(Q)VIEKACSSSDKHALVISWLK
lau Hedgehog     ERKDVLDDLTEVELKWKSLF(Q)VIEKACSSSEKHALVTPWLK
afr Elephant     ERKDVLDDLTEVELKWDSLL(Q)VIEKACSSSDKHALVTLWLK
afr Rock_hyrax   DRKDVLDDLTEVELKWDSLL(Q)VIEKACSSSDKHALVTLWLK
afr Tenrec       ERKDILDDLTQVELKWSSLL(Q)VIEKACSSSERRALVTLWLK
xen Armadillo    ERKDVLDHLTEVDLKWNSLL(Q)IIEKACYSPEKHALVTLWLK
mar Opossum      ERKTILDDLTKLDLKWNIIL(R)IIQKACSGSDKHLLVSTWLK

NO 130
GN MACF1
IP IPI00432363
PE RAWVGNKNLILNS
MP 1941
EX Wagner
CL African great apes
DE microtubule-actin crosslinking factor 1
SQ
pri Human        LNQHTQLEGRLQDLRAWVGN(K)NLILNSKGSNSEIDVDSLNL
pri Chimpanzee   LNQHTQLESRLQDLRAWVGN(K)NLILNSKGSNSEIDVDSLNH
pri Gorilla      LNQHTQLEGRLQDLRAWVGN(K)NLILNSKGSNSEIDVDSLNR
pri Orangutan    LNQHTQLEGRLQDLRAWVGN(T)NLILNSKGSNSETDVDSLNR
pri Gibbon       LNQHTQLEGRLQDLRAWVGN(T)NLILNSKGSNSETDVDSLNR
pri Rhesus       LNQHTQLEGRLQDLRAWVGN(T)ILILNSKGSNSETDVDSLNR
pri Marmoset     LNQHTQLEGRLQDLRAWVGN(T)NLILNSKGSNSETDVDSLNC
pri Tarsier      LNQHIQLEGKLQDWRSWVVN(T)NLILNSKEYHSETDADSLKH
eua Treeshrew    LNQHTLLEGELQDLRAWAGS(V)SLALSSPGFGSETDVASLDR
eua Mouse        LNRHKELQGKLQDLRAWVGR(A)SLTLNSKGCDTETDADSLSH
eua Rat          LNWHKELEGKLQDLKAWVDR(T)SLTLHSKGCDTQTDADSLSP
eua Guinea_pig   LNQHTQLESELQDLRAWVGD(T)SVILNNKECNSEADVISLSH
eua Squirrel     LNQHMQLEGKLRGLRAWVGN(T)SLILNSKRYDSETDIDSLNH
eua Rabbit       LSQHAQLEDKLQDLRAWVGS(T)SLTLSSKGCSNKTDPDSLNH
eua Pika         LNQHMELEDKLKDLRAWVGD(T)SATLSSKSCSSKTDVNSLKH
lau Dog          LSQHEQLEGRLQDLRIWVGD(T)NLLLNSKEYNDETDTDSLRH
lau Horse        LTQHEQLEGKLQDLRAWVGN(T)NLILNSKEYNSEIDAESLSH
lau Cow          LNQYEQLEGRLQDLRAWVGD(T)HVILNSKEYDSETDAGSLNH
lau Dolphin      LNQYEQLEGKLQDLRAWVGN(T)HLILNNKEHVDETDADSLSH
lau Alpaca       LNQYEQLESKLQDLRAWVGN(T)HLLLNSKDYDNETDADSLNH
lau Microbat     LNQHKQLQDKLQNLRTWVGD(T)ILILNSKECSSETDADSLNQ
lau Shrew        LNQYEQLEGRLEVLRAWLSN(T)YITLKNKDYNSETNAENLSL
afr Elephant     LNQQVQLEDKLQELKAWVGN(T)NLTLNSKEYNNDTDADSLNH
afr Rock_hyrax   LNQQVQLEGRLEDLKAWIGN(T)SLNLSSKHYSNETDADSLNH
xen Armadillo    LNQHIQLKDRIQDLMSWIAN(T)NFILNSKEFNSETDAANLNH
xen Sloth        LNQHTQLKDRLEDLGAWIAN(S)NLILNSKACNSETDVANLNH
mar Opossum      LNQYEVLQGRLQELGTWINE(T)RHSLYNMEHVSKSDATNLNN
mar Wallaby      LNQHEVLQDKLQELGTWINE(T)SHFLYHRENVNESDAADLNN
pro Platypus     TDQHEKLQEKLHTLAAWVSD(T)HSSLDGRAPVTGLDTTSLNR

NO 131
GN MAP1S
IP IPI00296485.6
PE RGPVPAKPTVLFE
MP 380
EX Kim
CL simians
DE microtubule-associated protein 1S
SQ
pri Human        SLLAQLGITPLPLSRGPVPA(K)PTVLFEKMGVGRLDMYVLHP
pri Chimpanzee   SLLAQLGITPLPLSRGPVPA(K)PTVLFEKMGVGRLDMYVLHP
pri Gorilla      SLLAQLGITPLPLSRGPVPA(K)PTVLFEKMGVGRLDMYVLHP
pri Orangutan    SLLAQLGITPLPLSRGPVPA(K)PTVLFEKMGVGRLDMYVLHP
pri Gibbon       SLLAQLGITPLPLSRGPVPA(K)PTVLFEKMGVGRLDMYVLHP
pri Rhesus       SLLAQLGITPLPLSRGPVPA(K)PTVLFEKMGVGRLDMYVLHP
pri Baboon       SLLAQLGITPLPLSRGPVPA(K)PTVLFEKMGVGRLDMYVLHP
pri Marmoset     SLLAQLGITPLPLSRGPVPA(K)PTVLFEKMGVGRLDMYVLHP
pri Mouse_lemur  SLLARLGIAPLSLSRGAPPR(S)TTVLFQKMGVSRLDMYALQP
eua Mouse        SLLRSLGIAPLPLQRGPQPS(C)PTVLFEKLGVGRLELFVLHP
eua Rat          SLLRSLGIVPLPLQRGPQPS(C)PTVLFEKLGVGRLELFVLHP
lau Cat          SLLARLGIAPLTLNRGPLPA(E)PTVLFQKMGVGRLDMYVLHP
lau Horse        SLLARLGITPLPLTRGPLPA(E)PTVLFQKMGVGRLDMYVLHP
lau Cow          SLLSQLGITPVPLNRGPLPA(E)PTVLFQKMGVGRLDMYVLHP
lau Dolphin      SLLARLGITPLPLNRGPLPA(E)PTVLFQKMGVGRLDMYVLHP
afr Elephant     GLLARLGIPPLPLSRGPLPT(Q)PTVLFQKMGVGRLDMYVLHP
afr Rock_hyrax   GLLARLGISPLPLSRGPLPT(Q)PTVLFQKMGVGRLDMYVLHP
afr Tenrec       GLLARLGIAPLPLSRGPLPT(E)PTVLFQKMGVGRLDMYVLHP
mar Opossum      GYLERLGIPPTPLCRGQVAS(E)PMVLFLKMGVGRLDMYILHP
mar Wallaby      GYLERLGITPTPLCRGPVPS(E)PMVLFLKMGVGRLDMYVLHP
pro Platypus     GYLERLGLEAVPLCRGPSPA(E)PTVLFQKMGVGQLDLYVLHP

NO 132
GN MARC2
IP IPI00329552
PE CGNEAAKWFTNFL
MP 166
EX Wagner
CL apes
DE mitochondrial amidoxime reducing component 2
SQ
pri Human        HNCRIFGLDIKGRDCGNEAA(K)WFTNFLKTEAYRLVQFETNM
pri Chimpanzee   HKCRIFGLDIKGRDCGNEAA(K)WFTNFLKTEAYRLVQFETNM
pri Gorilla      HNCRIFGLDTKGRDCGNEAA(K)WFTDFLKTEAYRLVQFETNM
pri Orangutan    HNCRIFGLDIKGRDCGNEAA(K)WFTNFLKTEAYRLVQFETNM
pri Gibbon       HNCRIFGLDIKGRDCGNEAA(K)WFTNFLKTEAYRLVQFETNM
pri Rhesus       HNCRIFGLDIKGRDCGNEAA(Q)WFTNFLKTEVYRLVQFETNM
pri Baboon       HNCRIFGLDIKGRDCGNEAA(Q)WFTNFLKTEVYRLVQFETNM
pri Marmoset     YDCRIFGLDIKGRDCGNEAA(Q)WFTNFLKTEAYRLVQFETNM
pri Tarsier      HDCRVFGLDIKGRDFSDEAA(Q)WFPKFLTKETFRLAQFEMII
pri Bushbaby     XXXXVFGVDIKGRDCGDEAA(Q)WFSNFLKTEAFRLVQFETNM
pri Mouse_lemur  HDCRVFGLDIKGRDCGDEAA(Q)WFTNFLKTEAFRLVQFEMSM
eua Treeshrew    HDCRLFGLDIXGRDCGDEAA(Q)WFTSFLKTEPFRLVQFETNM
eua Mouse        HNCRLFGLDIKGRDCGDEVA(Q)WFTNYLKTQAYRLVQFDTSM
eua Kangaroo_rat XXXXIFGIDIQGRDCGDEIA(Q)WFTKFLKTEAFRLVQFETNM
eua Guinea_pig   LDCRLFGLDIKGRDCGDEVA(R)WFTSFLKTEAYRLVQYETSM
eua Squirrel     HDCRLFGLDIKGRDCGDEVA(Q)WFTNYLKTESYRLVQFETRM
eua Rabbit       HDCRIFGVDIQGRDCGDEVA(Q)WFTNFLKTEALRLVQFDTKL
eua Pika         HDCRIFGMDIQGRDCGDEVA(Q)WFTSFLKTEPYRLVQFDMKM
lau Dog          XXXXLFGMDIKGRDCGDEAA(Q)WFTSFLKTEAFRLVQFEKHM
lau Cat          HDCRLFGMDIKGRDCGDQAA(K)WFTSFLKTEAFRLVQFEDHM
lau Horse        HDCRLFVLDIKGRDCGDEAA(Q)WFTNFLKTEAFRLGQFEKNM
lau Cow          HDCRVFGLDIQGRDCGDEAA(Q)WFTSFLKTDAFRLVQFEKNM
lau Dolphin      HDCRVFGLDIQGRDCGDEAA(Q)WFTNFLKTEACRLVQFEKNM
lau Alpaca       RDCRVFGLDIKGRDCGDEAA(Q)WFTNFLKTEAFRLVQFESNM
lau Megabat      HDCRLSGLDIKGRDCGDEAA(Q)WFTNFLKTEALRLVQFEKNM
lau Hedgehog     HNCRLFGLDIQGRDCGNEAA(R)WFTDYFKTEAYRLVQFEENM
afr Elephant     XXXXVFGLDIKGRDCGDEAA(Q)WFTNFLKTEPFRLVQFETNM
afr Rock_hyrax   QDCRIFGLDIKGRDCGEEAA(Q)WFTNFLKTAPFRLVQFETSM
afr Tenrec       XXXXLFGLDIQGRDCGDEAA(Q)WFSNFLKTEPYRLVQFETKM
xen Armadillo    QDCRLFGLDIKGRDCGDEAA(R)WFTTFLKTEAFRLVQFETNM
xen Sloth        XXXXLFGLDVKGRDCGNEAA(Q)WFTTFLKTEAFRLVQFETNM
mar Opossum      QNCRVFGVDIQGRDCGDEIA(Q)WITSFLKSETFRLVQFETQM
mar Wallaby      RDCRVFGVDVQGRDCGDEVA(Q)WISSFLKTETFRLVQFETHM
pro Platypus     LDCRVFGSDIQGRDCGPEVA(Q)WITGFLANDGYRLVQFEPQM

NO 133
GN MARS
IP IPI00008240.2
PE IFQQLLKRGFVLQ
MP 375
EX Kim, Wagner
CL apes
DE methionyl-tRNA synthetase
SQ
pri Human        GRTTTPQQTKITQDIFQQLL(K)RGFVLQDTVEQLRCEHCARF
pri Chimpanzee   GRTTTPQQTKITQDIFQQLL(K)RGFVLQDTVEQLRCEHCARF
pri Gorilla      GRTTTPQQTKITQDIFQRLL(K)RGFVLQDTVEQLRCEHCARF
pri Orangutan    GRTTTPQQTKITQDIFQRLL(K)RGFVLQDTVEQLRCEHCARF
pri Gibbon       GRTTTPQQTKITQDIFQRLL(K)RGFVLQDTVEQLRCEHCARF
pri Rhesus       GRTTTPQQTKITQDIFQRLL(T)RGFVLQDTVEQLRCEHCARF
pri Baboon       GRTTTPQQTKITQDIFQRLL(T)RGFVLQDTVEQLRCEHCARF
pri Marmoset     GRTTTPQQTKITQDIFQRLL(A)RGFVLQDTVEQLRCERCARF
pri Tarsier      XXXXXXXXXXITQDIFQRLL(A)RGFVLQDTVEQLRCERCARF
pri Mouse_lemur  GRTTTPQQTKITQDIFQRLL(T)RGFVLRDTVEQLRCEHCARF
eua Treeshrew    GRTTTPQQTKITQDIFQRLL(A)RGFVLQDSVEQLRCERCARF
eua Mouse        GRTTTPQQTKITQDIFQRLL(T)RGFVLRDTVEQLRCERCARF
eua Rat          GRTTTPLQTKITQDIFQRLL(T)RGFVLQDTVEQLRCEQCARF
eua Kangaroo_rat GRTTTPQQTKITQDIFQRLL(T)RGFVVQDTVEQLRCEHCARF
eua Guinea_pig   GRTTTPQQTKITQDIFHRLL(T)RGFILEDTVEQLRCEHCARF
eua Rabbit       GRTTTPQQTKITQDIFQRLL(S)RGFVLQDTVEQLRCERCARF
eua Pika         GRTTTPQQTKITQDIFQRLL(S)RGFVLQDTVEQLRCENCARF
lau Dog          GRTTTPQQTKITQDIFQRLL(T)RGFVLQDTVEQLRCERCARF
lau Cat          XXXXXXXXXXITQDIFQRLL(T)RGFVFQDTVEQLRCEHCARF
lau Horse        GRTTTPQQTKITQDIFQRLF(S)RGFVLQDTVEQLRCEQCARF
lau Cow          GRTTTPQQTKITQDIFQRLL(A)RGFVLQDTVEQLRCEHCARF
lau Dolphin      GRTTTPQQTKITQDIFQRLV(A)RGFVLQDTVEQLRCEHCARF
lau Megabat      GRTTTPQQTEITQDIFQRLW(N)RGFVLQDTVEQLRCESCARF
lau Microbat     GRTTTSQQTEITQDIFQRLW(N)RGFVLQDTVEQLRCERCARF
lau Hedgehog     GRTTTPQQTKITQDIFQRLY(E)RGFVLQDTVEQLRCERCARF
afr Elephant     GRTTTQQQTEITQDIFQRLL(A)RGFVLQDTVEQLRCEHCARF
afr Rock_hyrax   GRTTTPQQTKITQDIFQRLL(E)RGFVLQDAVEQLRCEQCARF
mar Wallaby      GRTTTPLQTKITQDIFQRLL(A)RDFLLTDTVEQLRCERCSRF
pro Platypus     GRTTTPQQTTIAQDIFQRLL(A)RGFLHTATVAQLRCERCARF

NO 134
GN MCM3
IP IPI00013214
PE IFDQLAKSLAPSI
MP 346
EX Wagner
CL African great apes
DE minichromosome maintenance complex component 3
SQ
pri Human        IAKIKKFSKTRSKDIFDQLA(K)SLAPSIHGHDYVKKAILCLL
pri Chimpanzee   IAKIKKFSKTRSKDIFDQLA(K)SLAPSIHGHDYVKKAILCLL
pri Gorilla      IAKIKKFSKTRSKDIFDQLA(K)SLAPSIHGHDYVKKAILCLL
pri Orangutan    IAKIKKFSKTRSKDIFDQLA(R)SLAPSIHGHDYVKKAILCLL
pri Gibbon       IAKIKKFSKTRSKDIFDQLA(R)SLAPSIHGHDYVKKAILCLL
pri Rhesus       IAKIKKFSKTRSKDIFDQLA(R)SLAPSIHGHDYVKKAILCLL
pri Baboon       IAKIKKFSKTRSKDIFDQLA(R)SLAPSIHGHDYVKKAILCLL
pri Marmoset     IAKIKKFSKTRSKDIFDQLA(R)SLAPSIHGHDYVKKAILCLL
pri Tarsier      IAKIKKFSKTRSKDIFDQLA(K)SLAPSIHGHDYVKKAILCLL
pri Bushbaby     IAKIKKFSKTRSKDIFEQLA(R)SLAPSIHGHDYVKKAILCLL
pri Mouse_lemur  IAKIKKFSKTRSKDIFDQLA(R)SLAPSIHGHDYVKKAILCLL
eua Treeshrew    IAKIKKFSKTRSKDIFDQLA(R)SLAPSIHGHDYVKKAILCLL
eua Mouse        IAKIKKFSKTRSKDVFEQLA(R)SLAPSIHGHDYVKKAILCLL
eua Rat          IAKIKKFSKTRSKDVFEQLA(R)SLAPSIHGHDYVKKAILCLL
eua Kangaroo_rat IAKIKKFSKTRSKDIFDQLA(R)SLAPSIHGHDYVKKAILCLL
eua Guinea_pig   IAKIKKFSKTRSKDIFDQLA(R)SLAPSIHGHDYVKKAILCLL
lau Dog          IAKIKKFSKTRSKDIFDQLA(R)SLAPSIHGHDYVKKAILCLL
lau Horse        IAKIKKFSKTRSKDIFDQLA(R)SLAPSIHGHDYVKKAILCLL
lau Cow          IAKIKKFSKTRSKDIFDQLA(R)SLAPSIHGHDYVKKAILCLL
lau Microbat     IAKIKKFSKTRSKDIFDQLA(K)SLAPSIHGHDYVKKAILCLL
lau Hedgehog     IAKIKKFSKTRSKDIFDQLA(R)SLAPSIHGNDYVKKAILCLL
lau Shrew        IAKIKKFSKTRSKDIFDQLA(R)SLAPSIHGHEYVKKAILCLL
afr Elephant     IPKIKKFSKTRSKDIFDQLA(R)SLAPSIHGHDYVKKAILCLL
afr Rock_hyrax   IPKIKKFSKTRSKDIFDQLA(R)SLAPSIHGHEYVKKAILCLL
afr Tenrec       IAKIKKFSKTRSKDIFDQLA(R)SLAPSIHGHDYVKKAILCLL
xen Armadillo    IAKIKKFSKTRSKDIFDQLA(R)SLAPSIHGHDYVKKAILCLL
xen Sloth        IAKIKKFSKTRSKDIFDQLA(R)SLAPSIHGHDYVKKAILCLL
mar Opossum      VAKIKKFSKTRSKDIFDQLS(R)SLAPSIHGHEYIKKAILCML

NO 135
GN MCM5
IP IPI00018350
PE SEHSIIKDFTKQK
MP 696
EX Wagner
CL simians
DE minichromosome maintenance complex component 5
SQ
pri Human        EKQLKRRFAIGSQVSEHSII(K)DFTKQKYPEHAIHKVLQLML
pri Chimpanzee   EKQLKRRFAIGSQVSEHSII(K)DFTKQKYPEHAIHKVLQLML
pri Gorilla      EKQLKRRFAIGSQVSEHSII(K)DFTKQKYPEHAIRKVLQLML
pri Orangutan    EKQLKRRFAIGSQVSEHSII(K)DFTKQKYPEHAIHKVLQLML
pri Gibbon       EKQLKRRFAIGSQVSEHSII(K)DFTKQKYPEHAIHKVLQLML
pri Rhesus       EKQLKRRFAIGSQVSEHSII(K)DFTKQKYPEHAIHKVLQLML
pri Baboon       EKQLKRRFAIGSQVSEHSII(K)DFTKQKYPEHAIHKVLQLML
pri Marmoset     EKQLKRRFAIGSQVSEHSII(K)DFTKQKYPEHAILKVLQLML
pri Bushbaby     EKQLKGYLASDSQVSQHSIV(Q)DFTKQKYQEHAMHKVLQLMI
pri Mouse_lemur  EKQLKRRFAIGSQVSEHSII(Q)DFTKQKYPEHAIHKVLQLML
eua Mouse        EKQLKRRFAIGSQVSEHSIV(Q)DFTKQKYPEHAIRKVLQLML
eua Rat          EKQLKRRFAIGSQVSEHSIV(Q)DFTKQKYPEHAIRKVLQLML
eua Guinea_pig   EKQLKRRFAIGSQVSEHSIV(Q)DFIKQKYPEHAIYKVLQLML
eua Squirrel     ERQLKRRFAIGSQVSEHSII(Q)DFTKQKYPEHAIHKVLQLML
eua Rabbit       EKQLKRRFAIGSQVSEHSIV(Q)DFIKQKYPEHAVHKVLQLML
eua Pika         QKQLKRRFAIGSQVSEHSII(Q)DFIKQKYPAHAIHKVLQLVL
lau Dog          EKQLKRRFAIGSQVSEHSII(Q)DFTKQKYPEHAIHKVLQLML
lau Cat          EKQLKRRFAIGSQVSEHSII(Q)DFTKQKYTEHAIHKALQLMF
lau Horse        EKQLKRRFAIGSQVSEHSII(Q)DFTKQKYPEHAVHKVLQLML
lau Cow          EKQLKRRFAIGSQVSEHSII(Q)DFTKQKYPEHAIHKVLQLML
lau Dolphin      EKQLKRRFAIGSQVSEHSII(Q)DFTKQKYPEHAIHKVLQLML
lau Megabat      EKQLKRRFAIGSQVSEHSII(Q)DFTKQKYPEHAIHKVLQLML
lau Microbat     EKQLKRRFAIGSQVSEHSII(Q)DFTKQKYPEHAIHKVLQLML
afr Elephant     EKQLKRRFAIGSQVSEHSII(Q)DFTKQKYPEHAIHKVLQLML
afr Tenrec       EKQLKRRFAIGSQVSEHSII(Q)DFTKQKYPEHTVYKVLQLML
mar Wallaby      EKQLKRRFAIGSQVSEHSIV(Q)DFTKQKYPEHAIYKVLQLMM

NO 136
GN MCM7
IP IPI00299904.3
PE NARRYAKLFADAV
MP 75
EX Kim, Wagner
CL simians
DE minichromosome maintenance complex component 7
SQ
pri Human        VAEDDPELVDSICENARRYA(K)LFADAVQELLPQYKEREVVN
pri Chimpanzee   VAEDDPELVDSICENARRYA(K)LFADAVQELLPQYKEREVVN
pri Gorilla      VAEDDPELVDSICENARRYA(K)LFADAVQELLPQYKEREVVN
pri Orangutan    VAEDDPELVDSICENARRYA(K)LFADAVQELLPQYKEREVVN
pri Gibbon       VAEDDPELVDSICENARRYA(K)LFADAIQELLPQYKEREVVN
pri Rhesus       VAEDDPELVDSICENARRYA(K)LFADSVQELLPQYKEREVVN
pri Baboon       VAEDDPELVDSICENARRYA(K)LFADSVQELLPQYKEREVVN
pri Marmoset     VAEDDPELVDSICENARRYA(K)LFADAVQELLPQYKEREVVN
pri Tarsier      VAEDDPELVDSICENAKRYA(R)LFADAVQELLPQYKEREVVN
pri Mouse_lemur  VAEDDPELVDSICENAKRYA(R)LFADAVQELLPQYKEREVVN
eua Mouse        IAEDDPELVDSICENAKRYS(R)LFGDVVQELLPEYKEKEVVN
eua Rat          VAEDDPELVDSICENAKRYS(R)LFADVVQELLPEYKEKEVVN
eua Guinea_pig   VAEEDPELVDSICENAKRYM(R)LFADTIQELLPQYKDREVVN
eua Squirrel     VAEDDPELVDSICENAKRYA(R)LFADAVQELLPQYKEREVVN
eua Rabbit       VAEDDPELVDSICENAKRYA(R)LFADAVQELLPQYKDREVVN
eua Pika         VAEDDPELVDSICENAKRYS(R)LFADAVQELLPQYKTREVVN
lau Dog          VAEDDPELVDSICENARRYT(R)LFADAVQELLPQYKEREVVN
lau Cat          VAEDDPELVDSICENARRYA(R)LFADAVQELLPQYKEREVTN
lau Horse        IAEDDPELVDSICENAKRYV(R)LFADAVQELLPQYKEREVVN
lau Cow          IAEDDPELVDSICENTKRYA(R)LFADAVQELLPQYKEREVVN
lau Dolphin      VAEDDPELVDSICENTKRYA(K)LFADAIQELLPQYKEREVVN
lau Alpaca       VARDDPELVDSICESTKRYV(R)LFADAVQELLPQYKAREVVN
lau Hedgehog     VAEDDPELVDSICENAKRYT(R)LFADAVHELLPQYKDREVVN
afr Elephant     IAEDDPELVDSICENAKRYA(R)LFADAVQELLPQYKEREVVN
xen Armadillo    VAEEDPELVDSICENAKRYA(R)IFADAVQELLPQYKEREVVN
xen Sloth        VAEEDPELVDSVCENAKRYV(R)LFADAVQELLPQYKEREVVS
mar Opossum      IAEEDPELVDSISENTKRYT(Q)LFTEAVQELLPQYKEKEVVN
mar Wallaby      VAEEDPELVDSISENTKRYT(Q)LFTDAVQELLPQYKEKEVVN

NO 137
GN MDH2
IP IPI00291006.2
PE LSHIETKAAVKGY
MP 74
EX Kim
CL simians
DE malate dehydrogenase 2, NAD (mitochondrial)
SQ
pri Human        TLYDIAHTPGVAADLSHIET(K)AAVKGYLGPEQLPDCLKGCD
pri Chimpanzee   TLYDIAHTPGVAADLSHIET(K)AAVKGYLGPEQLPDCLKGCD
pri Gorilla      TLYDIAHTPGVAADLSHIET(K)AAVKGYLGPEQLPDCLKGCD
pri Orangutan    TLYDIAHTPGVAADLSHIET(K)AAVKGYLGPEQLPDCLKGCD
pri Gibbon       TLYDIAHTPGVAADLSHIET(K)AAVKGYLGPEQLPDCLKGCD
pri Rhesus       TLYDIAHTPGVAADLSHIET(K)AVVKGYLGPEQLPDCLKGCD
pri Baboon       TLYDIAHTPGVAADLSHIET(K)AVVKGYLGPEQLPDCLKGCD
pri Marmoset     TLYDIAHTPGVAADLSHIET(K)ATVKGYLGPEQLPDCLKGCD
pri Tarsier      TLYDIAHTPGVAADLSHIET(R)ATVKGYLGPEQLPDCXKGCD
pri Bushbaby     TLYDIAHTPGVAADLSHIET(R)ATVKGYLGPEQLPDCLKGCD
pri Mouse_lemur  TLYDIAHTPGVAADLSHIET(R)ATVKGYLGPEQLPDCLKGCD
eua Treeshrew    TLYDIAHTPGVAADLSHIET(R)ANVKGYLGPEQLPDCLKGCD
eua Mouse        TLYDIAHTPGVAADLSHIET(R)ANVKGYLGPEQLPDCLKGCD
eua Rat          TLYDIAHTPGVAADLSHIET(R)ANVKGYLGPEQLPDCLKGCD
eua Kangaroo_rat TLYDIAHTPGVAADLSHIET(R)AKVKGFLGPEQLPDCXEGCD
eua Guinea_pig   TLYDIAHTPGVAADLSHIET(R)ATVKGYLGPEQLPDCLKGSD
eua Rabbit       TLYDIAHTPGVAADLSHIET(R)ATVKGYLGPEQLPDCLKGCD
eua Pika         TLYDIAHTPGVAADLSHIET(R)ATVKGYLGPEQLPDCLKGCD
lau Dog          TLYDIAHTPGVAADLSHIET(R)ATVKGYLGPEQLPDCLKGCD
lau Cat          TLYDIAHTPGVAADLSHIET(R)AAVKGYLGPEQLPDCLKGCD
lau Horse        TLYDIAHTPGVAADLSHIET(R)ATVKGYLGPEQLPDCLKGCD
lau Cow          TLYDIAHTPGVAADLSHIET(R)ATVKGYLGPEQLPDCLKGCD
lau Dolphin      TLYDIAHTPGVAADLSHIET(R)ATVKGHLGAGQLPDCPEGCD
lau Microbat     TLYDIAHTPGVAADLSHIET(R)ASVKGYLGPEQLPDCLKGCD
lau Hedgehog     TLYDIAHTPGVAADLSHIET(R)ANVKGYLGPEQLPDCLKGCD
lau Shrew        TLYDIAHTPGVGADLSHIET(R)ANVKGYLGPEQLPDCLKGCE
afr Rock_hyrax   TLYDIAHTPGVAADLSHIET(R)AEVKGYLGPEQLPDCLKGCN
xen Sloth        TLYDIAHTPGVAANLSHIET(R)ATVKGYLGPEQLPDCLKGCD
mar Opossum      TLYDIAHTPGVGADLSHIET(R)AKVKGYMGPEQLPDCLKGCD
mar Wallaby      TLYDIAHTPGVAADLSHIET(R)ATVKXYLGPEQLPDCLKGCD
pro Platypus     TLYDIAHTPGVAADLSHIET(R)ANAKGYLGPEQLPDCLKGCD

NO 138
GN MET
IP IPI00029273.1
PE RRLKETKDGFMFL
MP 223
EX Kim
CL haplorhines
DE met proto-oncogene (hepatocyte growth factor receptor)
SQ
pri Human        SSYFPDHPLHSISVRRLKET(K)DGFMFLTDQSYIDVLPEFRD
pri Chimpanzee   SSYFPDHPLHSISVRRLKET(K)DGFMFLTDQSYIDVLPEFRD
pri Gorilla      SSYFPDHPLHSISVRRLKET(K)DGFMFLTDQSYIDVLPEFRD
pri Orangutan    SSYFPDHPLHSISVRRLKET(K)DGFMFLTDQSYIDVLPEFRD
pri Gibbon       SSYFPDHPLHSISVRRLKET(K)DGFMFLTDQSYIDVLPEFRD
pri Rhesus       SSYFPHHPLHSISVRRLKET(K)DGFMFLTDQSYIDVLPEFRD
pri Baboon       SSYFPHHPLHSISVRRLKET(K)DGFMFLTDQSYIDVLPEFRD
pri Marmoset     SSYFPDHSLHSISVRRLKET(K)DGFMFLTDQSYVDVLPEFRD
pri Tarsier      SSYFPDHSLHSISVRRLKET(K)DGFKFLTDQSYIDVLPEFQD
pri Bushbaby     SSYFPNHPLHSISVRRLKET(Q)DGFKFLTDQSYIDVLPEFRD
pri Mouse_lemur  SSYFPDHPLHSISVRRLKET(Q)DGFKFLTDQSYIDVLPEFRD
eua Treeshrew    NSYLPDHSLHSISVRRLKET(Q)DGFKFLTDQSYIDVLPEFRD
eua Mouse        SSYPPGYSLHSISVRRLKET(Q)DGFKFLTDQSYIDVLPEFQD
eua Rat          SSYPPDYSLHSISVRRLKET(Q)DGFKFLTDQSYIDVLPEFRD
eua Kangaroo_rat SSYLPDHSLHSISVRRLKET(Q)DGFKFLTDQSYIDVLPEFRD
eua Guinea_pig   SSYLPDHSLHSISVRRLKET(L)DGFKFLTDQSYIDVLPEFRD
eua Squirrel     SSYLPDHSLHSISVRRLKET(Q)DGFKFLTDQSYIDVLPEFRD
eua Rabbit       SSYLPDHSLHSISVRRLKET(Q)DGFKFLTDQSYIDVLPEFRD
eua Pika         SSYLPDHSLHSISVRRLKET(Q)DGFKFLTDQSYIDVLPEFRD
lau Dog          SSDHPDHSLHSISVRRLKET(Q)DGFKFLTDQSYIDVLPEFRD
lau Cat          SSYLTDHSLHSISVRRLKET(Q)DGFKFLTDQSYIDVLPE--R
lau Horse        SSYLPDHSLHSISVRRLKET(Q)DGFKFLTDQSYIDVLPEFRD
lau Cow          SSYLPDYILHSISVRRLKET(Q)DGFKFLTDQSYIDVLPELRD
lau Dolphin      SSYLPDHSLHSISVRRLKET(Q)DGFKFLTDQSYIDVLPEFRD
lau Alpaca       SSYLPDHSLHSISVRRLKET(Q)DGFKFLTDQSYIDVLPEFQD
lau Megabat      SSYLPDHSLHSISVRRLKET(Q)DGFKFLTDQSYIDVLPEFRD
lau Microbat     SSYPPDYSLHSISVRRLKET(Q)DGFKFLTDQSYIDVLPEFRD
lau Hedgehog     SSYLPDHSLHSMSVRRLKET(Q)DGFKFLTDQSYIDVLPEFQD
lau Shrew        SSYPPNHSLHSISVRRLKET(Q)DGFKFLTDQSYIDVLPEFRD
afr Elephant     SSYLPDHSLHSISVRRLKET(Q)DGFKFLTDQSYIDVLPEFRD
afr Rock_hyrax   SSYLPAHSLHSISVRRLKET(Q)DGFKFLTDQSYIDVLPEFRD
afr Tenrec       GSSLPGHALHSISVRRIKET(Q)DGFKFLTDKSYIDVLPEFQA
xen Armadillo    SSYLPDHSLHSISVRRLKET(Q)DGFKFLTDQSYIDVLPEFRD
xen Sloth        SSYFPDHSLHSISVRRLKET(Q)DGFKFLTDQSYIDVLPEFRD
mar Opossum      SSD-PDNSLHSISVRRLKET(Q)DGFKFLTDQSYIDVLPEFQD
mar Wallaby      SSY-SDHSLHSISVRRLKET(Q)DGFKFLTDQSYIDVLPEFRD
pro Platypus     SSSHPFSSLHSISVRRLKET(L)DGFKFLTDQSYIDVLPQFRD

NO 139
GN METTL10
IP IPI00411752.3
PE VAARSDKGSPGED
MP 19
EX Kim
CL catarrhines
DE methyltransferase like 10
SQ
pri Human        MSSGADGGGGAAVAARSD(K)GSPGEDGFVPSALGTREHWD
pri Chimpanzee   MSSGADGGGGAAVAARSD(K)GSPGEDGFVPSALGTREHWD
pri Orangutan    MSSGSDGGGGAAVAARSD(K)GSRGEDGFVPSALGTRKHWD
pri Gibbon       MSSVADGGGSAAVAARSD(K)GSAGEDGFVPSALGTREHWD
pri Baboon       MSSDADGGSGAEVAALSD(K)DSPGEDGFVPSALGTREHWD
pri Marmoset     MSAGADGSGGPEVAARSG(E)GSPGEDGFVPSALGTREHWD
pri Mouse_lemur  MSAGADGGGGAAVAVGSS(E)GFPLEDGFVPSALGTREHWD
eua Treeshrew    MSAGAGGGGGAVAAARSR(E)GSLDEDGFVPSALGTREHWG
eua Mouse        MNADAEGHSGAVVPAQSP(E)GSSAADDFVPSALGTREHWD
eua Rat          MNADAEGNSGAVVSAQSR(E)GSPVADDFVPSALGTREHWD
eua Kangaroo_rat MSAG--DGGGVVAAARSR(R)ASPVEGGFVPSALGTREHWD
eua Guinea_pig   MNAGADDHSCATVAARSR(E)DSLEEDSFVPSALGTREHWN
eua Rabbit       MSSGADGGGGGVAAAPSR(G)GSPAEDGFVPSALGTLEHWD
eua Pika         MSRGADGNGGREAAGRAG(A)RSPAEDGFAPSALGTREHWD
lau Cow          MNEGADYGGGAGGAARSP(G)CGPEGGGFVPSALGTREHWD
lau Dolphin      MSEGADDDGGAGAAARSR(G)GSPRGDGFAPSALGTREHWD
lau Megabat      MSADASGGDAAGVAARSG(A)VSPGGDGFAPSALGTQEHWD
afr Elephant     MSLGSDGGGGAAAAARSR(E)GSPGKDGFAPSALGTREHWD
xen Armadillo    MSTGADNGAGA--AAPSR(E)GSPEEVGFAPSALGTQEHWD
xen Sloth        MNTGADHGTGP--AVQSH(E)GSPREVGFAPSALGTREHWD

NO 140
GN METTL15
IP IPI00783001
PE VRQQVMKTSQLGS
MP 352
EX Wagner
CL primates
DE methyltransferase like 15
SQ
pri Human        FLLGISMTERFNLSVRQQVM(K)TSQLGSDHENTEEVSMRRAP
pri Chimpanzee   FLLGISMTERFNLSVRQQVM(K)TSQLGSDHENTEEVSMRRAP
pri Gorilla      FLLRISTTERFNLSVRQQVM(K)TLQLGSDHENMEEVSMRRAP
pri Orangutan    FLLGISMTERFNLSVRQQVM(K)TSQLGSDHENTEEVSKRRAP
pri Gibbon       FLLGISMTERFNLSVRQQVM(K)TSQLGSDYENTEEVSVRRAP
pri Rhesus       FLLGISMTERFNLSVRQQVM(K)TSQLGSGHENTEEVSMRRAP
pri Baboon       FLLGISMTERFNLSVRQQVM(K)TSQLGSGHENTEEVSMRRTP
pri Marmoset     FLLGISMTERFNLSVRQQVM(K)ASRLDSDHENVE-VSMRRAP
pri Tarsier      FLLGISMTERFNLSVRQKVI(K)TSQLSSGHENNEGVSMRRAS
pri Mouse_lemur  FLLGISMTEKFNLSVRQKVI(K)TSQLGSGHENQEGVSIKKAP
eua Mouse        FLLGISMTERFNLSIRQKVK(Q)TSQLDSDQETEERHS--RAP
eua Rat          FLLGISMTERFNLSIRQKVR(Q)TSQMDSDHEIEERRC--RAP
eua Squirrel     FLLGISMTEKFNLSVRQKVI(Q)TSQLSSYHANKEGSS--RAP
eua Rabbit       FLLGISMTERFNLSVRQKVV(Q)ASQLSSGPENKEEVSAGRAP
lau Dog          FLLGISMTERFNLSARQKVI(Q)ALKLGSGRENMEGASISKAP
lau Horse        FLLGISMAERFNLSARQKVI(Q)SSQLGSGHKNKEGGSMGRAP
lau Cow          FLLGISMTERFNLSARQKVI(Q)KSQLDSDQENKEGVSTGKAP
lau Dolphin      FLLGISMTQRFNLSARQKVI(Q)KSQLGSDHENTEGVSIGRAP
lau Alpaca       FLLGISMTERFNLSARQKVI(Q)KSQLDSDLGNKDGVSIGKAP
lau Megabat      FLLGISMTEKFNLSARQKVI(Q)TPQLDSDHENKEGVSVGRAP
afr Elephant     FLLGINMTERFNLSVRQKVL(K)SSQLGSDQENKEGVS--RAP
afr Tenrec       FLLGINMTERFNLSVRQKVS(R)ISRLDSAQKSEERVS--RDP
xen Sloth        FLLGISMTERFNLSARQKVI(Q)TSRLGLGQENKEGIPIERDP
pro Platypus     FLFKIDITEKFNLSRRKKIT(Q)VQRGNVDRENEEESSSDMSH

NO 141
GN MFN2
IP IPI00642329.2
PE HALHQDKQLHAGS
MP 171
EX Kim
CL simians
DE mitofusin 2
SQ
pri Human        SEEKRSAKTVNQLAHALHQD(K)QLHAGSLVSVMWPNSKCPLL
pri Chimpanzee   SEEKRSAKTVNQLAHALHQD(K)QLHAGSLVSVMWPNSKCPLL
pri Gorilla      SEEKRSAKTVNQLAHALHQD(K)QLHAGSLVSVMWPNSKCPLL
pri Orangutan    SEEKRSAKTVNQLAHALHQD(K)QLHAGSLVSVMWPNSKCPLL
pri Gibbon       SEEKRSAKTVNQLAHALHQD(E)QLHAGSLVSVMWPNSKCPLL
pri Rhesus       SEEKRSAKTVNQLAHALHQD(K)QLHAGSLVSVMWPNSKCPLL
pri Baboon       SEEKRSAKTVNQLAHALHQD(K)QLHAGSLVSVMWPNSKCPLL
pri Marmoset     SEEKRSAKTVNQLAHALHQD(K)QLHAGSLVSVMWPNSKCPLL
pri Tarsier      SEEKRSVKTVNQLAHALHQD(E)QLHAGSLVSVMWPNSKCPLL
pri Bushbaby     SEEKRSVKTVNQLAHALHQD(E)QLHAGSLVSVMWPNSKCPLL
pri Mouse_lemur  SEEKRSVKTVNQLAHALHQD(E)QLHAGSLVSVMWPNSKCPLL
eua Treeshrew    SEEKRSVKTVNQLAHALHQD(E)QLHAGSLVSVMWPNSKCPLL
eua Mouse        SEEKKSVKTVNQLAHALHQD(E)QLHAGSMVSVMWPNSKCPLL
eua Rat          SEEKKSVKTVNQLAHALHQD(E)QLHAGSLVSVMWPNSKCPLL
eua Guinea_pig   SEEKRSVKTVNQLAHALHQD(E)QLHAGSLVSVMWPNSKCPLL
eua Squirrel     SEEKRSVKTVNQLAHALHQD(E)QLNAGSLVSVMWPNSKCPLL
eua Rabbit       SEEKRSVKTVNQLAHALHQD(E)QLHAGSLVSVMWPNSKCPLL
lau Dog          SEEKRSVKTVNQLAHALHQD(E)QLHAGSLVSVMWPNSKCPLL
lau Cat          SEEKRSVKTVNQLAHALHQD(E)QLHAGSLVSVMWPNSKCPLL
lau Horse        SEEKKSVKTVNQLAHALHQD(E)QLHAGSLVSVMWPNSKCPLL
lau Cow          SEEKRSVKTVNQLAHALHQD(E)QLHAGSLVSVMWPNSKCPLL
lau Dolphin      SEEKRSVKTVNQLAHALHQD(E)QLHAGSLVSVMWPNSKCPLL
lau Alpaca       SEEKRSVKTVNQLAHALHQD(E)QLHAGSLVSVMWPNSKCPLL
lau Megabat      SEEKRSIKTVNQLAHALHQD(E)QLHAGSLVSVMWPNSKCPLL
lau Microbat     SEEKRSIKTVNQLAHALHQD(E)QLHAGSLVSVMWPNSKCPLL
afr Elephant     SEEKKSVKTVNQLAHALHQD(E)QLHAGSLVSVMWPNSKCPLL
afr Rock_hyrax   SEEKKNVKTVNQLAHALHQD(E)QLHAGSLVSVMWPNSKCPLL
afr Tenrec       WEEKRSVKTVNQLAHALHQD(E)QLHAGSVVSVMWPNSKCPLL
xen Armadillo    SEEKRSVKTVNQLAHALHQD(E)QLHAGSLVSVMWPNSKCSLL
mar Opossum      SEEKKSVKTVNQLAHALHQD(E)LLNAGSLVSVMWPNSKCSLL
pro Platypus     SEEKKSVKTVNQLAHALHQN(E)LLNAGSLVSVMWPNSKCSLL

NO 142
GN MRPL37
IP IPI00162330.3
PE VGPVGFKPETFRK
MP 407
EX Kim
CL simians
DE mitochondrial ribosomal protein L37
SQ
pri Human        WCLPVIKKRVVVEPVGPVGF(K)PETFRKFLALYLHGAA*
pri Chimpanzee   WCLPVIKKRVVVEPVGPVGF(K)PETFRKFLALYLHGAV*
pri Gorilla      WCLPVIKKRVVVEPVGPVGF(K)PETFRKFLALYLHGAV*
pri Orangutan    WCLPVIKKRVVVEPVGPVGF(K)PETFRKFLALYLHGAV*
pri Gibbon       WCLPVIKKRVVVEPVGPVGF(K)PETFRKFLALYLHGAV*
pri Rhesus       WCLPVIKKRVVVEPVGPVDF(K)PETFRKFLALYLHGAV*
pri Baboon       WCLPVIKKRVVVEPVGPVDF(K)PETFRKFLALYLHGAV*
pri Marmoset     WCLPVIKKRVVVEPVGPVGF(K)PETFRKFLALYLHGAV*
pri Tarsier      XXXXXXXXXXXXEPVGPIGF(Q)PETFRKFLALYLHGAM*
pri Bushbaby     XXXXXXXXXXXXEPVGPVDF(Q)PETFKKFLALYLHGAV*
eua Treeshrew    WCRPVIKKKVVVEPVGPTGF(Q)PETFRKFLALYLHGAV*
eua Mouse        WCRPVIKKKVVVEPVGPVDF(Q)PETFRKFLALYLHGVV*
eua Rat          WCRPVIKKKVVVEPVGPVDF(Q)PETFKKFLALYLHGAV*
eua Kangaroo_rat WCRPVIKKKVVVEPVGPIGF(Q)PETFRKFLALYLHGAL*
eua Guinea_pig   WCRPVIKKKVVVEPVGPVDF(Q)PETFRKFLALYLHGCPEP*
eua Rabbit       WCLPVIKKKVVVEPVGPTGF(Q)PETFRKFLALYLHGAV*
eua Pika         WCLPVIKKKVVVEPVGPVAF(Q)PETFRKFLALYLHGAV*
lau Dog          WCRPVIKKKVVVEPVGPTGF(Q)PETFRKFLALYLHGAV*
lau Cat          WCRPVIKXXXXXEPVGPTGF(Q)PETFRKFLALYLHGAV*
lau Horse        WCLPVVKKKVVVEPVGPTGF(Q)PETFRKFLALYLHGAV*
lau Cow          WCLPVIKKKVVVEPVGPTGF(Q)PETFRKFLALYLHGAV*
lau Dolphin      WCLPVVRRKAVVEPVGPTGF(Q)PETFRKFLALYLHGAV*
lau Megabat      WCLPVIKKKVVVEPVGPTGF(Q)PETFKKFLALYLHGAV*
lau Hedgehog     WCRPVIKKKVVVEPVGPIGF(Q)PETFRKFLALYLHGAV*
afr Elephant     WCRPVIKKKVVVEPVGPIGF(Q)PETFKKFLALYLHGVV*
afr Rock_hyrax   WCRPVIKKKVVVEPVGPTGF(Q)PETFRKFLALYLHGAV*
afr Tenrec       WCQPVVKKKVVVEPVGPTSF(H)PETFRKFLALYLHGAV*
mar Opossum      RCLPVIKKKVVVEPAGIIDY(Q)PETFRKFFALYLHGAA*
mar Wallaby      RCLPVIKKKVIVEPVGIIDY(Q)PGTFKKFLAMYLHGAA*

NO 143
GN MRPL45
IP IPI00790292
PE LVIPPEKSDRSIH
MP 94
EX Wagner
CL catarrhines
DE mitochondrial ribosomal protein L45
SQ
pri Human        KTEKEFMQHARKAGLVIPPE(K)SDRSIHLACTAGIFDAYVPP
pri Chimpanzee   KTEKEFMQHARKAGLVIPPE(K)SDRSIHLACTAGIFDAYVPP
pri Gorilla      KTEKEFMQHARKAGLVIPPE(K)SDRSIHLACTAGIFDAYVPP
pri Orangutan    KTEKEFMQHARKAGLVIPPE(K)SDRSIHLACTAGIFDAYVPP
pri Gibbon       KTEKEFMQHARKAGLVIPPE(K)PDRSIHLACTAGIFDAYVPP
pri Rhesus       KTEKEFMEHARKAGLVIPPE(K)LDHPIHLACTAGIFDAYIPP
pri Baboon       KTEKEFMEHARKAGLVIPPE(K)LDHPIHLACTAGIFDAYIPP
pri Marmoset     KTEKEFLEHARKAGVVIPPE(R)LERPIHLACSAGIFDAYVPP
pri Mouse_lemur  KTEREFMEHARKAGLVIPPE(R)LERPIHLACTAGIFDEYVPP
eua Treeshrew    KTEQEFREHARKAGLVIPPE(R)LERPIHLACTAGIFDAYVPP
eua Mouse        KTEKEFLEYARKAGLVIPQE(R)LERPIHLACTAGIFDPYVPP
eua Rat          KTEKEFLEYARKAGLVIPQE(R)LERPIHLACTAGIFDPYVPP
eua Kangaroo_rat RTEKEFLEHARKAGLVIPPE(T)LERPIHIACTAGIFDAYIPP
eua Guinea_pig   QTQKEFLEHARKAGLVIPPE(R)LERPIHLACTAGIFDAYVPP
eua Rabbit       RTEKEFMEHARKAGLVIPPE(H)LERPIHLACTAGIFDAYVPP
lau Dog          KSEKEFMEYARKAGLVIPPE(R)LERPLHLACTAGIFDAYVPP
lau Horse        KSEKEFVEHARKAGLVIPPE(R)LERPIHLACTAGIFDAYVPP
lau Cow          KTEKEFTEYARKAGLVIPQE(S)LERPIHLACTASIFDAYVPP
lau Dolphin      KSEKEFVEYARKAGLVIPPE(R)LERPIHLACTAGIFDAYVPP
lau Alpaca       KSEKEFIEYARKAGLVIPPE(R)LERPIHLACTAGIFDAYVPP
lau Megabat      KSEKEFIEQSRKAGVVIPPE(R)LERPIHLACTAGIFDAYVPP
lau Microbat     KSEKEFVEHFRKAGVVIPPE(R)LERPIHLACTGGIFDPYVPP
afr Elephant     ESEKEFIERARKAGLVIPPE(R)LERAIHLACTGGIFDSYVPP
afr Tenrec       KSQKEFLEHARKAGLVIPPE(R)LERLITVACTGGIFDAYVPP
xen Armadillo    KTEKEFIEYARKAGLVIPPE(R)MERPIHLACTVGIFDTYIPP
xen Sloth        KTEKEFIEYTRKAGLVVPQE(R)MERPIHLACTASILDAYVPP
mar Opossum      KTEKEFQDRARAAGIVIPKE(R)LERPIHLACTAGIFDAYVPP
mar Wallaby      KTEKEFQEHARAAGIVIPKE(R)LERPIHLACTAGIFDAYIPP

NO 144
GN MRPS11
IP IPI00010244
PE AAPSHTKFSIYPP
MP 59
EX Wagner
CL apes
DE mitochondrial ribosomal protein S11
SQ
pri Human        QLQDAAAKQKVEQNAAPSHT(K)FSIYPPIPGEESSLRWAGKK
pri Chimpanzee   QLQDAAAKQKVEQNAAPSHT(K)FSIYPPIPGEESSLRWAGKK
pri Gorilla      QLQDAAAKQKVEQNAAPSHT(K)FSIYPPIPGEESSLRWAGKK
pri Orangutan    QLQDAAAKQKVEVKAAPSHT(K)FSIYPPIPGEENSLRWAGKK
pri Gibbon       QLRDAAAKQKVEEKAAPSHT(K)FSIYPPIPGEESSLRWAGKK
pri Rhesus       QLQDAAAKQEVEEKAAPSHT(R)FSIYPPIPGEESSLRWAGKK
pri Baboon       QLQDAAAKQEDEEKAAPSHT(R)FSIYPPIPGEESSLRWAGKK
pri Marmoset     QLQDAAAKQEVEEKAAPRHT(S)FSIYPPIPGEESSLRWAGKK
pri Tarsier      ELQDAAAKQEVEENAAPSRS(A)FSIYPPMPGEESPLRWAEKK
pri Bushbaby     XXXXXXXKQEVEVTEAPSHS(S)FSIYPPILGEESPLRWAGKK
pri Mouse_lemur  ELQDAVAKQEVEENAAPSHS(P)FSIYPPVPGEESSLRWAGKK
lau Dog          QLQNQAAKQEAGEKATPSRS(G)FSIYPPVPGQESPLRWAGKK
lau Cat          QLQDAAAKQEVEEKAAPSRS(S)FSIYPPVPGQESPLRWAGKK
lau Horse        QLQDAAAKPEVE-KATSGGS(S)FSIYPPIPGQESSLRWAGKR
lau Cow          QMQDAAAKEEVEADPAPSRS(S)FSIYPPIPGQESSLRWAGKK
lau Dolphin      QMQDAAAKPEVEANPAPRRS(S)FSIYPPIPGQESSLRWAGKK
lau Megabat      LLQDGAAKRETEGKAAPSRS(S)FSIYPPIPGQESSLRWAGKK
afr Rock_hyrax   QRKEEATKNEVEVDTAPSRS(S)FSIYPPIPGQESSLRWAGKK
afr Tenrec       QLQGAAAKVEAEENGAPSRG(S)FSIYPPFPGEESSLRWAGKK
xen Sloth        QLHDAAAKQEVEEKAAPDRS(N)FSIYPPVPGQESSLRWAGKK
mar Opossum      RSLEVVEKKKLKXKDAQKRS(T)FSIFPPVPDQESSLTWGGKK

NO 145
GN MRPS9
IP IPI00641924.2
PE QGMAFSKSEGKRK
MP 271
EX Kim
CL simians
DE mitochondrial ribosomal protein S9
SQ
pri Human        ESKKQLIEPVQYDEQGMAFS(K)SEGKRKTAKAEAIVYKHGSG
pri Chimpanzee   ESKKQLIEPVQYDEQGMAFS(K)SEGKRKTAKAEAIVYKHGSG
pri Gorilla      ESKKQLIEPVQYDEQGMAFS(K)SEGKRKTAKAEAIVYKHGSG
pri Orangutan    ESKKQLIEPVQYDEQGMAFS(K)SEGKRKTAKAEAIVYKHGSG
pri Gibbon       ESKKQLIEPVQYDEQGMAFS(K)SEGKRKTAKAEAIVYKHGSG
pri Rhesus       ASKTQLIEPVQYDEQGMAFS(K)SEGKRKTAKAEAIVYEHGSG
pri Baboon       GSRKQLIEPVQYDEQGMAFS(K)SEGKRKTAKAEAIVYEHGSG
pri Marmoset     QSKKQLIEPVQYDEQGMAFS(K)SEGKRKTAKAEAIVYEHGSG
pri Tarsier      QSKKQPIEPVQYDEQGMAFS(T)SEGKRKSAKAEVVLYEHGSG
pri Bushbaby     QSKKQLIEPVRYDEQGMAFS(T)SEGKRKSAKAEATVYERGSG
pri Mouse_lemur  QSKKQLIEPVQYDEQGMAFS(R)SEGKRKXXXXXXXXXXXXXX
eua Treeshrew    QSKKQLIEPVQYDERGMAFS(R)SEXXXXXXXXXXXXXXXXXX
eua Mouse        QSKKQLIEPVQYDEQGMAFS(T)SEGRRKSATAQAVVYEHGSG
eua Rat          QSKKQLIEPVQYDEQGMAFS(T)SEGRRKSATARVVVYQHGSG
eua Kangaroo_rat QSKKQLIEPVQYDEQGMAFS(R)SEGKRKSAKAEATVYKHGSG
eua Guinea_pig   RSQQQQIPPVQHDAQGAPFS(I)GQGRRKTAQAEAVVTAQGSG
eua Rabbit       QSQQQLREPVQYDERGVAFS(A)CEGKRKSARAEAVVYEQGSG
eua Pika         QSQKQLSVPLQYDEHGVAFS(S)GEGKRKTAKAEATVYEQGSG
lau Dog          QSKKQLIEPVQYDEQGMAFS(T)SEGKRKSAKAEAIVYGHGSG
lau Horse        QSKKQLIEPVQYDEQGMAFS(R)SEGRRKSAEAEAVVYAHGSG
lau Cow          QSKKHLIEPLQYDEQGMAFS(T)GQGKRKTANAEAVVYGHGSG
lau Dolphin      QSKKQLIEPLQYDEKGMAFS(T)GQGNRKTAKAKVTVYDHGSG
lau Megabat      QSKKQLIEPLQYDEQGMAFS(T)SEGKRKSAKAEAVVYEHGSG
afr Elephant     QSKKQLTERLQYDEQGRAFS(T)SEGKRKSAKAEVVVYEHGNG
afr Rock_hyrax   QSQKQLIEPLQYDDQGMAFS(T)SEGKRKSAKAEVVVYEHGSG
xen Armadillo    QSKKHLIEPLQYDEQGMAFS(T)CEGKRKSAKAEAVVYERGSG
xen Sloth        QSKKHLIEPLQYDEQGMAFS(T)SEGKRKSAKAEAVVYEHGSG
mar Opossum      QSKKKLIEPLQYDEHGMAFS(S)GEGKRKTAKANVMVYEHGSG
pro Platypus     QSKKHLIEPLQYDEQGRAFS(I)GEGKRKTAVAKAILYENGSG

NO 146
GN MSTO1
IP IPI00306049.6
PE SVLKEPKYQEELE
MP 206
EX Kim
CL simians
DE misato homolog 1 (Drosophila)
SQ
pri Human        DGEAGRLEAFGQGESVLKEP(K)YQEELEDRLHFYVEECDYLQ
pri Chimpanzee   DGEAGRLEAFGQGESVLKEP(K)YQEELEDRLHFYVEECDYLQ
pri Gorilla      DGEAGRLEAFGQGESVLKEP(K)YQEELEDRLHFXVEECDYLQ
pri Orangutan    DGEAGRLEAFGQGESVLKEP(K)YQEELEDRLHFYVEECDYLQ
pri Gibbon       DGEAGRLEAFGQGESVLKEP(K)YQEELEDRLHFYVEECDYLQ
pri Rhesus       DGEAGRLEAFGQGESVLKEP(K)YQEELEDRLHFYVEECDYLQ
pri Baboon       DGEAGRLEAFGQGESVLKEP(K)YQEELEDRLHFYVEECDYLQ
pri Marmoset     DGEAGRLEAFGQGESVLKEP(K)YQEELEDRLHFYVEECDYLQ
pri Tarsier      DGEEGRLEAFGQGESVLKEP(R)YLEELEDRLHFYVEECDYLQ
pri Bushbaby     DGEAGRLEAFGQGESVLKEP(R)YLEELEDRLHFYVEECDFLQ
eua Treeshrew    DGETGRLEAFGQGESVLKEP(R)YLEELEDRLHFYVEECDYLQ
eua Mouse        DGETGRLEAFGQGESVLKEP(R)YLEELEDRLHFYVEECDYLQ
eua Rat          DGETGRLEAFGQGESVLKEP(R)YLEELEDRLHFYVEECDYLQ
eua Guinea_pig   EGETGRLEAFGQGESILKEP(R)YLEELEDRLHFYVEECDYLQ
eua Squirrel     DGETGRLEAFGQGESVLKEP(R)YLEELEDRLHFYVEECDYLQ
eua Rabbit       EGEAGRLEAFGQGESVLKEP(G)YLEMLEDRLHFYVEECDYLQ
lau Dog          DGEAGRLEAFGQGESILKEP(R)YLEELEDRLHFYLEECDYLQ
lau Cat          DGEAGRLEAFGQGESILKEP(R)CLEDVEDRLHFYVEECDHLQ
lau Horse        DGEAGRLEAFGQGESILKEP(R)YLEELEDRLHFYVEECDYLQ
lau Cow          DGEAGRLEAFGQGESILKEP(K)YLEELEDRLHFYVEECDYLQ
afr Elephant     DGETGRLEAFGQGESILKEP(G)YLEELEDRLHFYVEECDYLQ
mar Opossum      DGEAGRLEGFGQGESLLREP(T)YLEELEDRLHFYVEECDYLQ

NO 147
GN MTO1
IP IPI00180380.2
PE VDEIVLKNLHLNS
MP 200
EX Kim, Wagner
CL simians
DE mitochondrial translation optimization 1 homolog (S. cerevisiae)
SQ
pri Human        EDQLPCYLTHTNPRVDEIVL(K)NLHLNSHVKETTRGPRYCPS
pri Chimpanzee   EDQLPCYLTHTNPRVDEIVL(K)NLHLNSHVKETTRGPRYCPS
pri Gorilla      EDQLPCYLTHTNPRVDEIVL(K)NLHLNSHVKETTRGPRYCPS
pri Orangutan    EDQLPCYLTHTNPRVDEIVL(K)NLHLNSHVKETTRGPRYCPS
pri Gibbon       EDQLPCYLTHTNPKVDEIVL(K)NLHLNSHVKETTRGPRYCPS
pri Rhesus       EDQLPCYLTYTNPRVDEIVL(N)NLHLNSHVKETTRGPRYCPS
pri Baboon       EDQLPCYLTHTNPRVDEIVL(K)NLHLNSHVKETTRGPRYCPS
pri Marmoset     EDQLPCYLTHTNPRVDEIVL(K)NLHLNSHVKETIRGPRYCPS
pri Tarsier      EDQLPCHLTHTNPQVDEIVL(E)NLHLNSHVKETTRGPRYCPS
eua Treeshrew    EDQLPCYLTHTNPQVDEIIL(G)NLHLNSHVKETTRGPRYCPS
eua Mouse        EDQLPCYLTHTNPRVDAIVL(E)NLHLNSHVQETTKGPRYCPS
eua Rat          EDQLPCYLTHTNPRVDELVL(E)NLHLNSHVKETTRGPRYCPS
eua Kangaroo_rat EDQLPCYLTHTNPRVDGIVL(E)NLHLNSHIKETTKGPRYCPS
eua Guinea_pig   EDQLPCHLTHTNPRVDAIIL(E)NLHLNSHVKETTKGPRYCPS
eua Rabbit       EDQLPCYMTHTNPKVDEIVL(E)NLHLNSHVKETTRGPRYCPS
eua Pika         EDQLPCYLTHTNPQVDKIVL(G)NLHLNSHVQETTRGPRYCPS
lau Dog          EDQLPCYLTHTNPRVDEIVL(E)NLHLNSHIKETTRGPRYCPS
lau Horse        EDQLPCYLTHTNPRVDEIVL(Q)NLHLNSHVKETTRGPRYCPS
lau Cow          EDQLPCYLTHTNPRVDEIVL(E)NLHLNCHVKETTRGPRYCPS
lau Alpaca       EDQLPCYLTHTDPRVDEIVL(E)NLHLNSHIKETTRGPRYCPS
lau Megabat      EDQLPCYLTHTNPRVDEIVL(E)NLHLNSHIKETTRGPRYCPS
lau Microbat     EDQLLCYLTHTNPRVDEIIL(E)NLHLNSHIKETTKGPRYCPS
lau Hedgehog     EDQLSCYLTHTNPRVDEIVL(E)NLHLNHHIKETTRGPRYCPS
lau Shrew        EDQLPCYLTHTNPKVDEIVL(E)NLHLNSHIKETTRGPRYCPS
afr Elephant     EDQLPCYLTHTSPRVDEIVL(E)NLHLNSHVKETTRGPRYCPS
afr Rock_hyrax   EDQLPCYLTHTNSRVDEIVL(E)NLHLNSHVKETTRGPXXXXX
xen Armadillo    EDQLPCYLTHTNPTVDKIVL(E)NLHLNSHVKETTRGPRYCPS
xen Sloth        EDQLPCYLTHTNPTVDKIVL(E)NLHLNSHVKETTRGPRYCPS
mar Opossum      EDQLPCYLTHTNSRVDQIVK(E)NIHLNTHIKETTKGPRYCPS
mar Wallaby      EEQLPCYLTYTNSRVEQIVK(E)NIHLNPHVKETTKGPXXXXX
pro Platypus     EDQLPCYLTFTNSEVEQIVR(E)NIHLNNHVKETTRGPRYCPS

NO 148
GN MUM1
IP IPI00922531
PE YQEVGAKVLQRTN
MP 643
EX Wagner
CL simians
DE melanoma associated antigen (mutated) 1
SQ
pri Human        EGQLDLVVKYLQGVYQEVGA(K)VLQRTNGDRIRFILDVLLPE
pri Chimpanzee   EGQLDVVVKYLQGVYQEVGA(K)VLQRTNGDRIRFILDVLLPE
pri Gorilla      EGQLDLVVKYLQGVYQEVGA(K)VLQRTNGDRIRFILDVLLPE
pri Orangutan    EGQLDLVVKYLQGVYQEVGA(K)VLQRTNGDRIRFILDVLLPE
pri Gibbon       EGQLDLVVKYLQGVYQEVGA(K)VLRRTNGDRIRFILDVLLPE
pri Rhesus       EGQLDLVVKYLQGVYQEVGA(K)VLRRTNGDRIRFILDVLLPE
pri Baboon       EGQLDLVVKYLQGVYQEVGA(K)VLRRTNGDRIRFILDVLLPE
pri Marmoset     EGQLDLVVKYLQGVYQEVGA(K)VLRRSNGDRIRFILDVLLPE
pri Mouse_lemur  EEQLDLVVKYLQGVCQEMGS(T)VLTRTHGDRIRFVLDVLLPE
eua Mouse        EAQLDEVVEYLQGVCRDMDG(Q)VPERGSGDRIRFILDVLLPE
eua Rat          EAQLDEVVEYLQGVCRDMDG(E)MPARGSGDRIRFILDVLLPE
eua Kangaroo_rat EEQLDRVVKYLQGVYQEIDS(E)MLTRINGDRIRFILDVLLPE
eua Guinea_pig   EEQLERVVKYLQGVYQDMDG(D)KLARARGDRIRFILDVLLPE
eua Squirrel     EEQLDLVVKYLQGLYQQMDG(E)VLAGVNGDSIRFILDVLLPE
eua Pika         EGQLDCVVAYLQGVCQEAGS(E)ALARTNGDRVRFILDVLLPE
lau Dog          EEQLDLVVKYLQGVYQETGS(R)TLARVNGDRIRFILDVLLPE
lau Cat          EEQLDLVVKYLQGVYQETGS(R)ALARINADRIRFILDVLLPE
lau Horse        EEQLDLVVKYLQGLFQETGS(R)MLARINGDRIRFILDVLLPE
lau Cow          EEQLDLVVKYLQGVYKQAGC(Q)LLARGHGDGIRFILDVLLPE
lau Dolphin      EEQLDLVVKYLQGVYQEAGS(R)LLARRHADGIRFVLDVLLPE
lau Megabat      EEQLDRVVKYLQGLCQDADG(R)ALARSSGDRIRFILDVLLPE
lau Microbat     EEQLDLVVKYLQGVYRETGS(R)MLTQINRDRIRFILDVLLPE
lau Hedgehog     EAQLDLVAAYLQGLGRRLGG(R)LLAHGSGDGVRLVLEVLLPE
afr Elephant     EEQLDLVVKYLQGVYQETGS(T)VLTRVNGDRIRFILDVLLPE
afr Rock_hyrax   EEQLELVVRYLQGVYQEMGS(T)VLARAHGDRIRFILDVLLPE
afr Tenrec       EEQLDLVVKYLQRVCQETGS(T)ALTRAHGDCVRLILHVLLPE
mar Opossum      DEQLDLVVKYLQGVYQEIDS(K)MLTLINGDRIKFILDVLLPE

NO 149
GN MX1
IP IPI00167949.6
PE PADIGYKIKTLIK
MP 197
EX Kim
CL simians
DE myxovirus (influenza virus) resistance 1, interferon-inducible protein p78 (mouse)
SQ
pri Human        IDLPGITRVAVGNQPADIGY(K)IKTLIKKYIQRQETISLVVV
pri Chimpanzee   IDLPGITRVAVGNQPADIGY(K)IKTLIKKYIQRQETISLVVV
pri Gorilla      IDLPGITRVAVGNQPADIGY(K)IKTLIKKYIQRQETISLVVV
pri Orangutan    IDLPGITRVAVGNQPADIGY(K)IKTLIKKYIQRQETISLVVV
pri Gibbon       IDLPGITRVAVGNQPADIGY(K)IKTLIKKYIQRQETISLVVV
pri Rhesus       IDLPGITRVAVGNQPPDIGY(K)IKTLIRKYIQRQETINLVVV
pri Baboon       IDLPGITRVAVGNQPPDIGY(K)IKTLIRKYIQRQETINLVVV
pri Marmoset     IDLPGITRVAVGNQPADIGR(K)IKALIRKYIQRQETISLVVV
pri Tarsier      IDLPGITRVAVGNQPADIGR(Q)IKALIRKYIYRQQTINLVVV
pri Bushbaby     IDLPGITRVAVGNQPADIGR(Q)IKKLIKKYITKQETINLVVV
pri Mouse_lemur  IDLPGITRVAVGNQPADIGR(Q)IKVLIKKYINKQETINLVVV
eua Treeshrew    IDLPGITRVAVGNQPADIGR(Q)IKTLIKKYIHKQETINLVVV
eua Mouse        IDLPGITRVAVGNQPADIGR(Q)IKRLIKTYIQKQETINLVVV
eua Rat          IDLPGITRVAVGNQPADIGR(Q)IKRLITNYIQKQETINLVVV
eua Kangaroo_rat IDLPGITRVAVGDQPADIGY(Q)IKRLILKYIQKQETINLVVV
eua Guinea_pig   IDLPGITRVPVGNQPADIGR(Q)IKRLIRKYIQKEETINLVVV
eua Rabbit       IDLPGITRVAVGGQPADIGH(Q)IKALIRKYIRRQETINLVVV
lau Dog          IDLPGITRVAVGNQPADIGR(Q)TKQLIRKYILKQETINLVVV
lau Cat          IDLPGITRVAVGNQPADIGR(Q)TKQLIRKYIVKQETINLVVV
lau Horse        IDLPGITRVAVGNQPADIGR(Q)IKTLIRKYIQRQETINLVVV
lau Cow          IDLPGITRVAVGNQPPDIEY(Q)IKSLIRKYILRQETINLVVV
lau Dolphin      IDLPGITRIAVGNQPADIEY(Q)IKSLIRNYILKQETINLVVV
lau Alpaca       IDLPGITRVAVGNQPHDIEY(Q)IKSLIRKYIQRQETINLVVV
lau Megabat      IDLPGITRVALGNQPADIGF(Q)IKRLIKKYILKQQTINLVVV
afr Elephant     IDLPGITRVAVGNQPADIGQ(Q)IKALIRKYIHKQETISLVVV
afr Rock_hyrax   IDLPGITRVAVGNQPADIGR(Q)IKSLIRKYINKQETINLVVV
xen Sloth        IDLPGITRVAVGNQPADIGW(Q)IKSLIRKYINKQETINLVVV
pro Platypus     IDLPGITRVAVGNQPHDIGK(K)IKSLIQKYIKKQETINLVVV

NO 150
GN MYO6
IP IPI00844172.1
PE AQLARQKEEESQQ
MP 993
EX Kim
CL humans
DE myosin VI
SQ
pri Human        KREDDEKRIQAEVEAQLARQ(K)EEESQQQAVLEQERRDRELA
pri Chimpanzee   KREDDEKRIQAEVEAQLARQ(R)EEESQQQAVLEQERRDRELA
pri Gorilla      KREDDEKRIQAEVEAQLARQ(R)EEESQQQAVLEQERRDRELA
pri Orangutan    KREDDEKRIQAEVEAQLARQ(R)EEESQQQAVLEQERRDRELA
pri Gibbon       KREDEEKRIQAEVEAQLARQ(R)EEESQQQAVLEQERRDRELA
pri Rhesus       KREDDEKRIQAEVEAQLARQ(K)EEESQQQAVLEQERRDRELA
pri Baboon       KREDDEKRIQAEVEAQLARQ(K)EEESQQQAVLEQERRDRELA
pri Marmoset     KREDDEKRIQAEVEAQLARQ(R)EEESQQQAVLEQERRDRELA
pri Bushbaby     KRQDDEKRIQAEVEAQLARQ(R)EEESQQQAVLEQERRDRELA
eua Treeshrew    XXXXXXXXXXAEVEEQLARQ(R)EEESQQQAVLEQERRDRELA
eua Mouse        KREDDEKRIQAEVEAQLARQ(R)EEESQQQAVLAQECRDRELA
eua Rat          KREDDEKRIQAEVEAQLARQ(R)EEESQQQAVLAQECRDRELA
eua Kangaroo_rat RREDDEKRIQAEVELQLARQ(R)EEEAQQQAVLEQERRDRELA
eua Guinea_pig   KREDDERRIQAEVEEQLARQ(R)EEESQQQAVLEQERRDRELA
eua Squirrel     XXXXXXXXXXXDEEEQLARQ(R)EEESQQQAVLEQERKDRELA
eua Rabbit       KREDDEKRIQAEVEEQLARQ(R)EEESQQQAVLEQERRDRELA
lau Dog          KREDDEKRIQAEVEEQLARQ(R)EEESQQQAVLEQERRDRELA
lau Cat          KREDDEKRIQAEVEEQLARQ(R)EEESQQQAVLEQERRDRELA
lau Horse        KREDDEKRIQAEVEEQLAQQ(R)EEESQQQAVLEQERRDRELA
lau Cow          KREDDEKRIQAEVEAQLARQ(R)EEESQQQAVLEQERRDRELA
lau Dolphin      KREDDEKRIQAEVEEQLARQ(R)EEESQQQAVLEQERRDRELA
lau Alpaca       KREDDEKRIQAEVEEQLARQ(R)EEESQQQAVLEQERRDRELA
lau Megabat      KREDDEKRIQAEVEKQLARQ(R)EEESQQQAVLEQERRDRELA
lau Microbat     KREDDEKRIQAEVEEQLARQ(R)EEESQQQAVLEQERRDRELA
lau Hedgehog     KREDDEKRIQAEVEEQLARQ(R)EEESQQQAVLEQERRDRELA
lau Shrew        KREDDEKRIQVEVEAQLARQ(R)EEESQQQAVLEQERRDRELA
afr Elephant     KREDDEKRIQAEVEAQLARQ(R)EEESQQQAVLEQERRDRELA
afr Rock_hyrax   KREDDEKRIQAEVEAQLARQ(R)EEESQQQAVLEQERRDRELA
afr Tenrec       KREDDEKRIQAEVEEQLARQ(R)EEESQQQAVLEQERRDRELA
xen Armadillo    KREDDEKRIQAEVEEQLARQ(R)EEESQQQAVLEQERRDRELA
mar Opossum      KREDDERRIQAEVEEQLARQ(R)EEETQQQAVLEQERRDRELA
mar Wallaby      KREDDEKRIQAEVEEQLARQ(R)EEETQHQAVLEQERRDRELA
pro Platypus     KREDDEKRMQAEVEEQLAKQ(R)EEENQQQAVREQERRDCELA

NO 151
GN N4BP2
IP IPI00328825.2
PE EDEKEMKEILMAG
MP 1339
EX Kim
CL apes
DE NEDD4 binding protein 2
SQ
pri Human        KDYVKFSDEEEFMNEDEKEM(K)EILMAGSSLSAGVSGEDKTE
pri Chimpanzee   KDYVKFSDEEEFMNEDEKEM(K)EILMAGSSLSAGVSGEDKTE
pri Gorilla      KDYVKFSDEEEFMNEDEKEM(K)EILMAGSSLSAGVSGEDKTE
pri Orangutan    KDYVKFSDEEEFMNEDEKEM(K)EILMAGSSLSAGVSGEDKTE
pri Gibbon       KDYVKFSDEEEFMNEDEKEM(K)EILMAGSSLSAGVSGEDKTE
pri Rhesus       KDYVKFSDAEEFMNEDEKEM(E)EILMAGSSLSAGVSEEDNTE
pri Baboon       KDYVKFPDAEEFMNEDEKEM(E)EILMAGSSLSAGVSEEDNTE
pri Marmoset     KDYVKFLDTEEFMNEDEQEM(E)EILMAGSGLSTGVSEEDKTE
pri Bushbaby     KDFMKFPNMEEFMKEDEQEM(E)KILMAGSSLSDRVSEEDKAE
eua Treeshrew    KDDVQVPNTEEYTKEDEQEM(E)NILMAGXXXXXGVGEEDKTE
eua Mouse        KDYVKFANMEEFINEDKQEM(E)KNLMPGSGWSAGVSEEGKAE
eua Rat          KDYVKFANMEEFINEDKQEM(E)KSLMPGTGWSAGVSEESKPE
eua Guinea_pig   RDFEKFVDTEEFMNEDEQEM(E)KILMEGS-LSAGIGEEDKIE
eua Squirrel     KDYVKFVNSEEFMNVDEQEM(E)KILMAGSSLSVGVNEEDKTE
eua Pika         KDYVKFANMEEFMNEEEQEM(H)KILMAGSNLSTGVSEEDKIE
lau Dog          KDYMRFPSTEEFMNEDEQEM(E)KILMAESTFSAEVSEEDKTE
lau Cat          KTYVKFPNTEEFMNEDEQEM(E)KILMAESTLSAGVSEEDKTE
lau Horse        KDYLKFPNTEEFMNEDEQEM(E)KILMAGSTLSAGVGEEDKTE
lau Cow          KDYVKFPNTEEFMIEEEQEM(E)TILMAGSALSAGVTGEDKAE
lau Dolphin      RDYVKFPNTEEFMNEDEQEV(E)KILMAGSALSAEVTEEDKTE
lau Alpaca       KDYVKFPNTEEFMNEDEREM(E)KILMAGNTLSAGVTEEGKTE
lau Megabat      KNYVKFPKTEEFMNEDEKKM(E)KVLMAGSTMSAGVSEEGKTE
lau Microbat     KDYMKFPNTEEYMNEDEQEM(E)KILMAGSTLPAGVSEEDKTE
lau Hedgehog     KGYVNFPTTEEFINEGEQEM(E)KILLSGNTLSARVSEEDKTE
lau Shrew        KDHGKFTNTEEFMSEVEQEM(E)KLLMTGSTLSAGGGEEGKTE
afr Elephant     KDYVEF-DKEEFMDEDVQEM(E)KILMAGNSWSAGVSEEDKTE
afr Rock_hyrax   KDYVDF-DKEEFIDEDVQEI(E)NILREGNSWSAGVSEEDKTE
afr Tenrec       KDHVGF-DKEECVDKDVREM(E)KILMSGDSLSAGVSENGKTE
xen Armadillo    KDYVEF-FKEEFMNEDEQEM(E)KILMAGSSLSAEVSEEFKTE
mar Opossum      KTDKKF-SAEGFVKEDKPEM(E)NVV-PKANLAGGVIEEDRAE

NO 152
GN NACC1
IP IPI00045207.2
PE PKVKVLKAEDDAY
MP 483
EX Kim
CL primates
DE nucleus accumbens associated 1, BEN and BTB (POZ) domain containing
SQ
pri Human        MCTNARRVVRKSWMPKVKVL(K)AEDDAYTTFISETGKIEPDM
pri Chimpanzee   MCTNARRVVRKSWMPKVKVL(K)AEDDAYTTFISETGKIEPDM
pri Gorilla      MCTNARRVVRKSWMPKVKVL(K)AEDDAYTTFISETGKIEPDM
pri Orangutan    MCTNARRVVRKSWMPKVKVL(K)AEDDAYTTFISETGKIEPDM
pri Gibbon       MCTNARRVVRKSWMPKVKVL(K)AEDDAYTTFISETGKIEPDM
pri Baboon       MCTNARRVVRKSWMPKVKVL(K)AEDDAYTTFISETGKIEPDM
pri Marmoset     MCTNARRVVRKSWMPKVKVL(K)AEDDAYTTFISETGKIEQDM
pri Bushbaby     MCTNARRVVRKSWMPKLKVL(K)TEGDTYTTFISETGKIEPDM
pri Mouse_lemur  MCTNARRAMRKSWMPKLKVL(K)AEGDAYTAFISETGKIEPDM
eua Mouse        MCTNARRVVRKSWLPKTKPL(L)VEGDNYSSFISDTCKIEPDM
eua Rat          MCTNARRVVRKSWLPKTKPL(L)VEGDNYSSFISDTGKIEPDM
eua Guinea_pig   MCTNARRVVRKSWMPKAKPP(L)AEGDSYTSFIGDTCKMEPDM
eua Squirrel     MCTNARRVVRKSWMPKAKPM(L)AEGDAYTTFISDTGKMEPDM
eua Pika         MCTNSRRVVRKSWIPKLKLL(T)AEGDAYTTFISDTGKIEPDV
lau Dog          MCTNARRVVRKSWIPKLKVL(M)AEGDAYTTFISDTGKIEPDM
lau Horse        MCTNARRVVRKSWMPKLKLL(T)AEGDAYTTFISDTGKIEPDM
lau Cow          MCTNARRVVRKSWIPKVKPL(M)AEGDAYTTFISYTGKIEPDM
lau Dolphin      MCTNARRVVRKSWIPKVKPL(M)AEGDAYTTFISYTGKIEPDM
lau Alpaca       MCTNARRVMRKRWISKVKPL(M)AEGDSSTTSFSYTGKTEPDM
lau Shrew        MCTNARRVVRKSWMPKLKVL(K)TEGDTYTTFISDTVKMELDM
afr Elephant     MCTNARRVVRKSWIPKVKPL(T)AEGDVYTTFISDTGKIEPDM
mar Opossum      MCTNARRVVRKSWIPKLKLL(M)AEGDTYTTFINDTGKMEPDM
mar Wallaby      MCTNARRVVRKSWIPKPKLL(M)VEGDTYTAFINDSGKIEPDM
pro Platypus     MCTNACRVVRKSWIPKLKLM(M)AKGDTYTTFINDTGEMEPDI

NO 153
GN NAGLU
IP IPI00008787.3
PE ERALAAKPGLDTY
MP 59
EX Kim, Wagner
CL catarrhines
DE N-acetylglucosaminidase, alpha
SQ
pri Human        LLGPGPAADFSVSVERALAA(K)PGLDTYSLGGGGAARVRVRG
pri Chimpanzee   LLGPGPAADFSVSVERALAA(K)PGLDTYSLGGGGAARVRVRG
pri Gorilla      LLGPGPAADFSVSVERALAA(K)PGLDTYSLGGGGAARVRVRG
pri Orangutan    LLGPGPAADFSVSVERALAA(K)PGLDTYSLGGGGAARVRVRG
pri Gibbon       LLGPGPAADFSVSVERALAA(K)PGLDTYSLGGGGAARVRVRG
pri Rhesus       LLGPGPAADFSVSVERALAT(K)PGLDTYSLGGGGAARVRVRG
pri Baboon       LLGPGPAADFSVSVERALAT(K)PGLDTYSLGGGGAARVRVRG
pri Marmoset     LLGPGPAANFSVSVERALAA(E)PGLDTYSLSGGGAARVRVRG
pri Mouse_lemur  LLGPGRAADFSVSVERALAA(E)SGLDTYSLSGGGGARVRVRG
eua Treeshrew    LLGPGPATNFSVSVERSLAA(E)SGLDTYSLSGGGGAQVRLRG
eua Mouse        LLGPGPAANFLVSVERALAD(E)SGLDTYSLSGGGGVPVLVRG
eua Rat          LLGPGPAADFLVSVERALAN(E)SGLDTYSLSGGGGVPVLVRG
eua Kangaroo_rat LLGPGPAANFLVSVERSLAA(E)SGSDTYSLSGGGEAPVRVRG
eua Guinea_pig   LLGPGPAAAFELSVERTLAA(G)PGADVFELRGGAAAGGRVRG
eua Squirrel     LLGPGPAADFSVSVERALAT(E)SGLDTYRLSGGGGARVQVRG
eua Rabbit       LLGPRRAADFAVSVERALAA(E)SGLDTYSLSGGGAARVRVRG
eua Pika         LLGPERAAEFVVSVERALAA(E)SGLDTYRLSGGGVTPVRVCG
lau Dog          LLGPGPAAAFSVSVKRALAA(E)SGLDTYRLSGGGATRVLVLG
lau Cow          LLGPGPAAAFSVSVERSLAT(E)SGLDTYRLSGGGATRVQVLG
lau Dolphin      LLGPGPAASFSVSVERALAA(E)SGLDTYRLSGGGVTRVRVLG
lau Megabat      LLGPGPAAAFSVSVERALAA(E)SGLDTYRLSGGGATRVQVLG
lau Hedgehog     LLGPELAASFSVWVKRALAA(A)SGLDTYRLSGGGAARVQVLG
afr Elephant     LLGPRPAADFSVSVERSLAA(E)SGLDTYRLSGGGAARVRVAG
mar Opossum      LLGPRAAAGFSVSVERALAP(A)PDADTYCLSGGGGTPVQVTG
mar Wallaby      LLGPRAAAGFSVAVXRSLAP(A)PGADTFRLSGGGGAPVRVAG

NO 154
GN NBN
IP IPI00299463.1
PE FRSLVIKNSTSRN
MP 665
EX Kim
CL primates
DE nibrin
SQ
pri Human        DDSEMLPKKLLLTEFRSLVI(K)NSTSRNPSGINDDYGQLKNF
pri Chimpanzee   DDSEMLPKKLLLTEFRSLVI(K)NSTSRNPSGINDDYGQLKNF
pri Gorilla      DDSEMLPKKLLLTEFRSLVI(K)NSTSRNPSGINDDYGQLKNF
pri Orangutan    DHSEMLPKKLLLTEFRSLVI(K)NSTSRNPSGINGDYGLLKNF
pri Gibbon       DDSEMLPKKLLLTEFRSLVI(K)NSTSRNPSGINDDYGQLKNF
pri Rhesus       DDSEMLPRKLLLTEFRSLVI(E)NSTSRNPSGIN-DYGQLNNF
pri Baboon       DDSEMLPRKLLLTEFRSLVI(E)NSTSRNPSGIN-DYGQLNNF
pri Marmoset     DESEMLPRKLLLTEFKSLVI(K)NSTSRNLSGISDDYGQLKNF
pri Tarsier      NDSEMLPRKVLLTEFRSLVV(K)NSTSRNLSGVNNDYGHLKNF
pri Bushbaby     DSSEMLPRNVLLTEFRSLVV(K)DYPCSDLSGINNDYGQLKNF
pri Mouse_lemur  DNSEMLPSKVLLTEFRSLVV(K)N--SRNPSIVNNDYGQLKNF
eua Treeshrew    VDIEVFPRKVLLTEFR-LYW(W)LITTKSKFGINNDYGQLKNF
eua Mouse        DSSEELPRKLLLTEFRSLVV(S)NHNSTSRNLCVNECGPLKNF
eua Rat          DSSEELPRKLLLTEFRSLVV(H)NNSSRNLCPLNGR-GELKNF
eua Guinea_pig   DSSEMLPTKVLMTEFRSLVV(N)NPSHRNLYGVSNDHGQLKNF
eua Squirrel     DNSEMLPRKVLLTEFRSLVV(N)NSTCRDPCVVNYDYGQIKNF
lau Dog          DDGDMLPRKVLLTEFRSLVV(N)NSTPRNASSVNSDYDQPKNF
lau Cat          GDGDMFPRKVLLTEFRSLVV(S)NFTSRNTSCVNSDYGQLKNF
lau Horse        DHGEMLPRKVLLIEFRSLVV(S)NSTSRDASRVNDDYGQLKNF
lau Cow          DGDEMLPKKVLLTEFRSLVV(C)NSTSRNASNVNNDYGELKNF
lau Dolphin      GDGEMLPKTVLLTEFRSLVV(R)NSTSRNASSVNNDYGELKNF
lau Alpaca       DDGEVLPRKVLLTEFRSLVV(S)NSASRNAASVNNDYGQLKNF
lau Megabat      KDGELLPARVLLTEFRSLVV(G)DSASRSAAGVSGDYRQTKNF
lau Microbat     GDGEMPLRKVLLTEFRSLVV(S)GSASRNAPSVRGDQGPLKNF
lau Hedgehog     EVGEMLPRKLLLTEFRSLVI(D)NSNSRSISNVNNDYGQLKNF
lau Shrew        EDGEMLPRNVLLTEFRSLVV(N)KPDSRKAPGVNHDYGQLKNF
afr Elephant     DDGEKLPRKVILTEFRSLVV(T)NSASRNPSLVDNDYGQLKNF
afr Rock_hyrax   DDGEKLLTKLIVTEFRSLVV(S)NSTSRNASIENNDYGQLKNF
afr Tenrec       CGGEGIPRKMILIEFRSLVT(Y)KIMSRNPSIVNNKYAQLKKF
xen Armadillo    SDTEMLPRKVLQTEFRLLVI(G)NSTSRNPLIVNDDYGQLKNF
xen Sloth        GDGEMLPRKVLLTEFRSLVV(N)NSTSRNPAIINDDYGQLKNF
mar Opossum      DSCERLPKNLLLTEFRSLVV(S)HSGSRNASIIRNDYGQLNNF
pro Platypus     DDSEVLPKKLLLTEFRSLVV(G)HTRARNSSTVRSDYGHRNNF

NO 155
GN NBR1
IP IPI00299920.5
PE ERGAEGKPGVEAG
MP 435
EX Kim
CL humans
DE neighbor of BRCA1 gene 1
SQ
pri Human        GDSMYSSALSQPGLERGAEG(K)PGVEAGQEPAEAGERLPGGE
pri Chimpanzee   GDSMYSSALSQPGLERGAEG(E)PGVEAGQEPAEAGERLPGGE
pri Gorilla      GDSMYSSALSQPGLERGAEG(E)PGVEAGQEPAEAGERLPGGE
pri Orangutan    GDSMYSSALSQPGLERGAEG(E)PGVEAGQGPAEAGERLPGGE
pri Gibbon       GDSMYSSALSQPGLERGAEG(E)PGVEAGQEPAEAGERLPGGE
pri Rhesus       GDSMYSSALSQPGLERGAEG(E)PGVEAGQEPAEAGERLPGGE
pri Baboon       GDSMYSSALSQPGLERGAEG(E)PGVEAGQEPAEAGERLPGGE
pri Marmoset     GDSMYSSALSQPGLERGAEG(E)PGVEAGQEPAEAGERLPGGE
pri Tarsier      GDSMYSSALSQPGLEQVAEG(E)PRVEAGQEPAEAGERLPGGE
eua Treeshrew    GDSMYSSALSQPGLERVAEG(E)PGIEAGQEPAEAGERLPGGE
eua Mouse        GDSMYSSALSQPGLERGAEG(E)PGIESGLEPTEARERLPERE
eua Rat          GDSMYSSALSQPGLERGAEG(E)PGIESGQEPAEARERLPERE
eua Squirrel     GDSMYSSALSQPGLDRGAEG(E)PGVEAGQEPAEAGERLPEEK
eua Rabbit       GDSMYSSALSQPGLERGAEG(E)PGIEAGQEPAEAGERLPAGE
lau Dog          GDSMYSSALSQPGLERGAEG(E)PGIEAG-------ERLAEGE
lau Cat          GDSMYSSALSQPGLERGAEG(E)PGVEAG-------ERVAGGE
lau Horse        GDSMYSSALSQPGLEQGAEG(E)PGVEAGQEPVKAGERRPGEE
lau Cow          GDSMYSSALSQPGLERGAEG(E)PGIEAGQEPVEAGERPLGGD
lau Dolphin      GDSMYSSALSQPGLERGAEG(E)PGVEAGQEPVEAGERPPGGE
lau Alpaca       GDSMYSSALSQPGLERGAEG(E)PGVEAGQEPAEAGESTPGGE
lau Megabat      GDSMYSSALSQQGLERGAEG(E)PGVEAGQEPVEAGERPPGGE
lau Microbat     GDSMYSSALSQPGLERGAEG(E)PGIEAGQEPVEAGETPPGGE
lau Shrew        GDSMYSSALSQPGLERGTEG(E)LGVEAGQEPIEDGGGSTGRE
afr Elephant     GDSMYSSALSQPGLERGAEG(E)PGVEAGQEPAEAGERLPGGE
afr Rock_hyrax   GDSMYSSALSQPGLERGAEG(E)PGVEAGQEPAEAGEGLPGGE
xen Armadillo    GDSMYSSALSQPGLERGTEG(E)PGVEAGQEPAEDGERLPGGK
xen Sloth        GDSMYSSALSQPGLERGTER(E)PGIEAGQEPAKDGERLPGGE
mar Opossum      GESMYSSALSQPGLERLTEL(E)QVVKAEQEPAEAYERLARGE
mar Wallaby      GESMYSSALSQPGLERLMEI(E)QVVKMEQEPAEAYEGLPRGD

NO 156
GN NCAPD2
IP IPI00299524.1
PE RGLDGIKELEIGQ
MP 1301
EX Kim, Wagner
CL humans
DE non-SMC condensin I complex, subunit D2
SQ
pri Human        IIDEFEQKLRACHTRGLDGI(K)ELEIGQAGSQRAPSAKKPST
pri Chimpanzee   IIDEFEQKLRACHTRGLDGI(E)ELEIGQAGSQRAPSAKKPST
pri Gorilla      IIDEFEQKLRACHTRGLDGI(E)ELEIGQAGSQRAPSAKKPST
pri Orangutan    VIDEFEQKLRACHTRGLDGI(E)ELEIGQAGSQRAPSAKKPST
pri Gibbon       IIDEFEQKLRACHTRGLDGI(E)ELEIGQAGSQRAPSAKKPST
pri Rhesus       VIDEFEQKLRACHTRGLDGI(D)ELEIGQAGSQRAPAAKRPST
pri Baboon       VIDEFEQKLRACHTRGLDGI(D)ELEIGQAGSQRAPAAKRPST
pri Marmoset     IIDEFEQKLRACHTRGLDGI(E)ELEISQTGSQRAPSAKKSST
pri Bushbaby     IIDEFEQKLRACHTRGLDGI(E)ELENGQGLSQRAPSARKQPT
pri Mouse_lemur  IIDEFEQKLRAFHTRGLDGR(E)ELEIGQGGSQRAPSARKQST
eua Treeshrew    IIDEFEKKLRACHTRGLDGG(E)EVETAQGAGLRAPSAKKQPT
eua Mouse        IIDEFEQKLRACHTRGMDGI(E)EFETGQGGSQRALSAKKPSA
eua Rat          VIDEFEQKLRACHTRGMDGI(E)ELETGQGGSQRALSAKKPSA
eua Squirrel     IIDEFEQKLRAYHTRGSDGI(E)ELEIGQGGSQRAPSARKQSS
eua Rabbit       MIDEFEQKLRACHTRGLDGV(E)ELEMGQGGSQRAPSAKKPAP
eua Pika         TIDEFEQKLRACHTRGLDAV(E)ELELGQVGSQRALSAKKHGP
lau Dog          IIDEFEQKLRVCHTRGLDAV(E)DLEVGQRGSQRAPSTKKPST
lau Cat          VIDEFEQKLRVCHTRGLDAV(E)DLEVGQGGSQRAPSAKKPST
lau Horse        IIDEFEQRLRACHTRGLDAM(E)ELEIGQGGSQRAPSAKKQST
lau Cow          VIDEFEQKLRTCHTRGLDTV(E)ELEVGQGSNQRAPSARKQPA
lau Dolphin      VIDEFEQKLRACHTRGLDAI(E)ELEIGQAGSQRAPSARKQPA
lau Alpaca       IIDEFEQKLRACHTRGLDAM(E)ELENGPGGSHRAPSAKKQPT
lau Hedgehog     MIDEFEQKLRTCHTRGLDAV(E)ELEAGQGDSQKALSTKKQPA
afr Elephant     VIDEFEQKLRACHTRGVDAL(E)EIEIGQAGSQSAPSAKKQSA
afr Rock_hyrax   IIDEFEQKLRACHTRGMDAL(E)ELELGQGGSQRAPSAKKQSA
afr Tenrec       LIDEFEQKLRACHTRGLEAV(E)ELEIGQGGSQSAPSAKKHSV

NO 157
GN NDUFB6
IP IPI00219385
PE NKFLENKSPWRKM
MP 54
EX Wagner
CL simians
DE NADH dehydrogenase (ubiquinone) 1 beta subcomplex, 6, 17kDa
SQ
pri Human        VLPPQKMGPMEKFWNKFLEN(K)SPWRKMVHGVYKKSIFVFTH
pri Chimpanzee   VLPPQKMGPMEKFWNKFLEN(K)SPWRKMVHGVYKKSIFVFTH
pri Gorilla      VLPPQKMGPMEKFWNKFLEN(K)SPWRKMVHGVYQKSIFVFTH
pri Orangutan    VLPPQKMGPMEKFWNTFLEN(K)SPWRKMVHGVYQKSIFVFTH
pri Gibbon       VLPPQRMGPMEKFWNKFLEN(K)SPWRKMVHGVYQKSIFVFTH
pri Rhesus       VLPPQKMWPMEKFWNKFLEN(K)SPWRRTVHGVYQKGIFIFTH
pri Baboon       VLPPQKMWPMEKFWNKFLEN(K)SPWRRTVHGVYQKGIFIFTH
pri Marmoset     VLPPEATWPMDRFWDKFLAN(K)SPWRNMVYKVYRKSMFIFTC
pri Tarsier      VLPPQRMWPMEKFWNKFLQN(P)SPWRNMMYKVYRSSIFAFTH
pri Mouse_lemur  VLPPRKPWPVEAFWDKFLQN(G)GPWRNTVYKVYQRSLFAFTH
eua Treeshrew    VLPPQRLWPLEEFWNKFLQN(R)TPWRNMVYKAYGHSIFAFTH
eua Mouse        VLPPRRMWPLERFWDNFLRD(G)AVWKNMVFKAYRSSLFAVSH
eua Rat          VLPPRRMWPLERFWNNFLRD(R)ALWKYMIFKAYRTSLFTVSH
eua Kangaroo_rat VLPPQKGWPMEEFWKNFVRN(G)SLWRKLVYSTYHNTVFAVTR
eua Guinea_pig   VLPPRRMWPLEQFWNTFLQD(R)APWKQMIYKVYWRSIFAVTH
eua Rabbit       VLPPQRTWPLESFWNKFLQN(E)APWRNVIYKVYRHSIFAFTH
eua Pika         VQPPQRTWPLERFWNKFLQN(E)APWRKMVYKTYRHSIFVFTH
lau Cat          VLPPQRVWPMERFWNKFLQD(Q)APWKKVIYKAYGHSIFAFTH
lau Cow          VLPPQRVSPVERFWNKFLQD(G)ALWKNVIYKTYRHSIFAFTH
lau Dolphin      VLPPRRMWPMEQFWNKFLQD(G)ASWKNVIYKTYRHSIFAFTH
lau Alpaca       VLPSRRVWPVEQFWNNFLQD(R)APWKNVIYKAYRHSVFAFTH
lau Hedgehog     VLPPQKLRPMEAFWNRFLRD(Q)APWKNMIYKVYRNSIFAFTN
lau Shrew        VLPPQKVGPVESFWNKFLHD(Q)APWKNLVYKTYRHSVFAFTH
afr Rock_hyrax   VLPPQRLGPVEEFWNKFLQN(P)APWRNALFKVYRRSVFVFTH
afr Tenrec       VLPPQRLGPLETFWNKFLLH(P)TPWRKALHKVYRHSVLAFTH
xen Armadillo    VLPPRKVWPLEEFWNKFLQN(K)SPWRKTLYKVYGHSVFAFTH

NO 158
GN NDUFB9
IP IPI00255052.5
PE KATQLLKEAEEEF
MP 65
EX Kim
CL simians
DE NADH dehydrogenase (ubiquinone) 1 beta subcomplex, 9, 22kDa
SQ
pri Human        RARFEEHKNEKDMAKATQLL(K)EAEEEFWYRQHPQPYIFPDS
pri Chimpanzee   RARFEEHKNEKDMAKATQLL(K)EAEEEFWYRQHPQPYIFPDS
pri Gorilla      RARFEEHKNEKDMAKATQLL(K)EAEEEFWYRQHPQPYIFPDS
pri Orangutan    RARFEEHKNEKDMARATQLL(K)EAEEEFWYRQHPQPYIFPDS
pri Gibbon       RARFEEHKNEKDMAKATQLL(K)EAEEEFWYRQHPQPYIFPDS
pri Rhesus       RARFEEHKNEKDMMKATQLL(K)EAEEEFWFCQHPQPYIFPDS
pri Baboon       RARFEEHKNEKDMMKATQLL(K)EAEEEFWYFQHPQPYIFPDS
pri Marmoset     RARFEEHKNEKDMVKATLLL(K)EAEEEFWYRQHPQPYIFPDS
pri Tarsier      RARFEEHRNEKDMMKATQLL(R)EAEEEFWYRQHPQPYIFPDS
pri Bushbaby     RARFEEHRHEKDMRKATQLL(R)EAEEEFWYRQHPQPYIFPDS
pri Mouse_lemur  RARFEEHKNEKDMRKATQLL(R)EAEEEFWFRQHPQPYIFPDS
eua Treeshrew    RARFDEHKDEKDMMKATQLL(R)EAEKEFWYNQHPQPYIFPDS
eua Mouse        RARFEEHKNEKDMMRATQLL(R)EAEEEFWQNQHPQPYIFPDS
eua Rat          RARFEEHKNEKDMMKATQLL(R)QAEEEFWQNQHPQPYIFPDS
eua Kangaroo_rat RARFEEHRNEKDMMRATQLL(R)EAEEEFWQNQHPQPYIFPDS
eua Guinea_pig   RARFEEHKDEKDMIKATRLL(R)EGEEEFWRCQHPQPYIFPDS
eua Rabbit       RARFDEHKNEKDMMKATRLL(R)DAEEEFWCNQHPQPYIFPDS
lau Dog          RARFEEHKNEKDMVKATQLL(R)AAEEEFWHNQHPQPYIFPES
lau Cat          RARFEEHKNEKDMVKATQLL(R)EAEEEFWHNQHPQPYVFPES
lau Horse        RARFEEHKNEKDMVKATQLL(R)EAEEEFWYNQHPQPYIFPES
lau Cow          RARFDEHKNEKDMVKATQLL(R)EAEEEFWHGQHPQPYIFPES
lau Dolphin      RARFDEHKDEKDMVKATQLL(R)EAEEEFWHCQHPQPYIFPES
lau Alpaca       RARFDEHKNEKDMVKATRLL(R)EAEEEFWHSQHPQPYIFPES
lau Megabat      RARFEEHKNEKDMVKATQLL(R)EAEEEFWHNQHPQPYIFPES
lau Microbat     RARFDEHKNEKDMVKATRLL(R)EAEEEFWHNQHPQPYIFPDS
lau Hedgehog     RARFDEHKNEKDMVKATRLL(R)EAEEEFWQCQHPQPYIFPDS
afr Elephant     RARFDEHKNEKNMVKATQLL(K)EAEEEFWQCQHPQPYIFPDS
afr Rock_hyrax   RARFDEHKNEKDMVKATQLL(R)EAEEEFWYRQHPQPYIFPDS
afr Tenrec       RARFEEHKDEKDMIKATQLL(R)AAEEEFWHYQHPQPYIFPDS
xen Armadillo    RARFEEHKNEKDMVKATQLL(K)EAEMEFWHNQHPQPYIFPDS
mar Opossum      RDRFERNRNENDMMKATKLL(W)EGEKEFWANQHPQPYIFPDS
mar Wallaby      RARFEEHKNEKDMVKATKLL(M)KGEEEFWACQHPQPYTFPDS
pro Platypus     RARFEEHKNEKDMIKATKIL(K)AAEEEFWQRQHPQPYIFPDS

NO 159
GN NGDN
IP IPI00000162.5
PE AVTAQVKSLTQKV
MP 33
EX Kim
CL African great apes
DE neuroguidin, EIF4E binding protein
SQ
pri Human        SAVTLLKNLQEQVMAVTAQV(K)SLTQKVQAGAYPTEKGLSFL
pri Chimpanzee   SAVTLLKNLQEQVMAVTAQV(K)SLTQKVQAGAYPTEKGLSFL
pri Gorilla      SAVTLLKNLQEQVMAVTAQV(K)SLTQKVQAGAYPTEKGLSFL
pri Orangutan    SAVTLLKNLQEQVMAVTAQV(Q)SLTQKVQAGAYPTEKGLSFL
pri Gibbon       SAVTLLKNLQEQVMAVTAQV(Q)SLTQKVQAGAYPTEKGLSFL
pri Rhesus       SAVTLLKNLQEQVMAITAQV(Q)SLTQKVQAGAYPTEKGLSFL
pri Baboon       SAVTLLKNLQEQVMAITAQV(Q)SLTQKVQAGAYPTEKGLSFL
pri Marmoset     SAVTLLKNLQEQVMAVTAQV(Q)SLTKKVQAGAYPTEKGLSFL
pri Mouse_lemur  NAVTLLKNLQEQVMTVTAQV(Q)ALTKKVQAGAYPTEKGLSFL
eua Treeshrew    SAVTLLKNLQEQVMAVTAHV(Q)ALTKRVQAGAYPTEKXXXXX
eua Mouse        SSITLLKNLQEQVMAVTAQI(Q)ALTTKVRAGTYSTEKGLSFL
eua Rat          SSITLLKNLQEQVMAVTAQV(Q)ALTAKVRAGAYSTEKGLSLL
eua Kangaroo_rat GAVTLLKSLQEQVMAVTAQV(Q)TLTKKVQGGAYPTEKGLSFL
eua Guinea_pig   NAVTLLKNLQEQVMAVTAQV(Q)ALTKKVQAGAYPTEKGLSFL
eua Rabbit       AAVTLLKNLQEQVMAVTAQV(Q)ALTKKVKAGAYPTEKGLSFL
eua Pika         SAVTLLKTLQEQVMAVTAQV(Q)ALTKKVRAGTYPTEXGLSFL
lau Dog          SAVTLLKNLQEQVMAVTAQI(Q)ALIKKVQARAYPTEKGLSLL
lau Cat          SAVTLLKNLQDQVMAVTAQV(Q)ALTKKVQARAYPTEKGLSLL
lau Horse        SAVTLLKNLQEQVTAVTAQV(Q)ALTKKVQARAFPTEKGLSLL
lau Cow          NAVALLKNLQEQVMAVTAQV(Q)TLTKKVQAKAYPTEKGLSLL
lau Dolphin      NAVALLKNLQEQVMAVTAQV(Q)ALTKKVQAKAYPTEKGLSLL
lau Alpaca       NAVALLKNLQEQVMAVTTQV(Q)ALTKKVQAKAYPTEKGLSLL
lau Megabat      SAVALLKNLQEQVMAVTAQV(Q)ALTKKVQARAYPTEKGLSLL
lau Microbat     RAVALLKNLQEQVMAVTAQI(Q)ALTKKVQARTYPTEKGLSLL
lau Hedgehog     RAVALLKSLQEQVMAVTVQV(Q)ALTKKVQARTYPTEKGLSLL
afr Elephant     SALTLLKNLQEQVMAVTAQV(Q)ALTKKVQAGAYPTEKGLSFL
xen Armadillo    SAVTLLKNLQEQVMAVTAQV(Q)ALTKKVQTGAYPTEKGLSFL
xen Sloth        SAVTLLKNLQEQVMAVTAQV(Q)ALTKKVQAGAYPTEKGLSFL
mar Opossum      LALSLLNNLQEQVVAVTAQI(Q)ALTKKVQAGAYPTDKGLSFL
mar Wallaby      LALSLLKSLQEQVVAVTSQV(Q)ALTKKVRAGAYPTDKGLSLL
pro Platypus     ASLALLKTLQEQVMAVTAQV(Q)ALTQKVRAGAFPTKKGLSFM

NO 160
GN NGLY1
IP IPI00074605.2
PE NKSHKVKSSQQPA
MP 123
EX Kim
CL great apes
DE N-glycanase 1
SQ
pri Human        DLIAIERSSRLDGSNKSHKV(K)SSQQPAASTQLPTTPSSNPS
pri Chimpanzee   DLIAIERSSRLDGSNKSHKV(K)SSQQPAASTQLPTTPSSNPS
pri Gorilla      DLIAIERSSRLDGSNKSHKV(K)SSQQPAASTQLPTTPSSNPS
pri Orangutan    DLIAIERSSRLDGSNKSHKV(K)SSQQPAASTQLPTTPSSNPS
pri Gibbon       DLIAIERSSRLDGSNKSHKI(E)SSQQPAASTQLPTTPSSNPS
pri Rhesus       DLIAIERSSRLDGSNKSHKL(E)SSQQPAASTQLPTTPSSNPS
pri Baboon       DLIAIERSSRLDGSNKSHKV(E)SSQQPAASTQLPTTLSSNPS
pri Marmoset     DLIAIERSSRLDGSNKTLKI(E)SSPQPAASSQLPTTPSSNPS
pri Tarsier      DLIAIERSSRLDGSNKSHKA(E)SSQQPTANTQLSTAQSSDPD
pri Bushbaby     DLIAIERSSRLDGSTKSHKV(E)SSQPPATGTQVSTTESPNPN
pri Mouse_lemur  DLIAIERSSILDVSNKSHKV(E)SSQPPATSTQLPTTQSSNPN
eua Treeshrew    DLIAMGRRSRVDGSNKSHKA(E)ASEQPASSTQPATPQSPDLN
eua Mouse        DLIAIERSSRLDGSSK--KV(Q)FSQHPAA-AKLPLEQSEDPA
eua Rat          DLIAVERRSRLDGS--SQKV(E)FSQHPAA-VRLPAEQPEDPT
eua Kangaroo_rat DLIAIERSSRLDGSDKSFKV(G)SSQQQASGTQILTTQSSNLN
eua Guinea_pig   DLIAVERSSRLGESSKSHKA(E)SSQQPISSTQLPTAQSSNPS
eua Squirrel     DLIAIERSSRLDGSIKSHKV(E)SSKQPSTTTPLPTTQSSNAN
eua Rabbit       DLIAIERSSRLDGSSNSHKT(E)SSQQPATSTPLPATLSSGPT
lau Dog          DLIAIERSSRLDGSNKSYKV(E)LSQQPASSTQLPITQSSNPN
lau Horse        DLIAIERSSRLDGSNKSHKV(E)LPQQPAASIQLPTTQSSNPN
lau Cow          DLIAIERSSRLDGSEKSHKT(E)LSQHLAASSQLPTAPSSHPD
lau Dolphin      DLIAIERSSRLDGSDKSHKA(E)LSQQLAASTQLPTAQPSSPN
lau Alpaca       DLIAIERSSRLDGSNKSHKV(E)LPQQLAASTQLPTTQSSNLN
lau Megabat      DLIAIERSSRLNGSNKSHKV(E)LSQQPATSTQLPTTQTSDPN
lau Microbat     DLIATERSSRLDESYKSHKA(E)LSQQPATSIQLPTAQSSDPN
lau Hedgehog     DLIATERSSRLDGSNKSQKV(E)LPQQAPVSTQLPTTQSSNPS
afr Elephant     DLIAIERSSRLDGSNRNHKV(E)VSQQPAASAQLPTAQPSDAV
afr Rock_hyrax   DLIAIERSSRLDGSDKSRKV(E)LPQQPAASPQLPTGQSSGPV
afr Tenrec       DLIALERSSRLDGSNNNPKV(E)LSQQPASSTQLPAAPSSDPV
xen Armadillo    DLIAMERSSRLDGSKKSHKV(E)LSEQPTSNIQHPTVHSSNPV
xen Sloth        DLIAMERSNRLVGSNKNHKV(E)LSQQPASSIQLPTTQSSNPS
mar Wallaby      DLIAIERSSRLNDSTKTHKV(D)SSQRPTRDAQPVTPQLSRPS
pro Platypus     DLIAEERSSRLNGSSTGPRG(E)SSQQLTSPNQPILSWPAEPA

NO 161
GN NIT1
IP IPI00023779.1
PE SEPLGGKLLEEYT
MP 108
EX Kim
CL catarrhines
DE nitrilase 1
SQ
pri Human        FDFIARDPAETLHLSEPLGG(K)LLEEYTQLARECGLWLSLGG
pri Chimpanzee   FDFIARDPAETLHLSEPLGG(K)LLEEYTQLARECGLWLSLGG
pri Gorilla      FDFIARDPAETLHLSEPLGG(K)LLEEYTQLARECGLWLSLGG
pri Orangutan    FDFIARDPAETLRLSEPLGG(K)LLEEYSQLARECGLWLSLGG
pri Gibbon       FDFIARDPAETLRLSEPLGG(K)LLEEYTQLARECGLWLSLGG
pri Rhesus       FDFIARDPAETLRLSEPLGG(R)LLEEYTRLARECGLWLSLGG
pri Baboon       FDFIARDPAETLRLSEPLGG(R)LLEEYTQLARECGLWLSLGG
pri Marmoset     FDFIARDPAETLHLSEPLGG(K)LLEEYTQLARECGLWLSLGG
pri Tarsier      FDFIARDPAETLRLSEPLGG(K)LLGDYTQLARECGLWLSLGG
pri Bushbaby     FDFIARDPAETLHLSEPLGG(T)LLGEYTQLAXXXXXXXXXXX
pri Mouse_lemur  FDFIARDPAETLHLSE-LGG(N)LLGEYTQLAXXXXXXXXXXX
eua Treeshrew    FDFIARDPAETLRLSEPLGG(N)LLGEYTKLAXXXXXXXXXXX
eua Mouse        FDFIARNPAETLLLSEPLNG(D)LLGQYSQLARECGIWLSLGG
eua Rat          FDFIARNPAETLLLSEPLDG(D)LLGQYSQLARECGIWLSLGG
eua Kangaroo_rat FDFIARDPAETLQLSEPLDG(N)HLSQYTQLARECGLWLSLGG
eua Guinea_pig   FDFIARDPAETLRLSEPLGG(N)LLEDYIQLARECGLWLSLGG
eua Rabbit       FDFVARDPAETLRLSEPLGG(D)LLGAYTQLARECGLWLSLGG
eua Pika         FDFIAQDPAETLRLSEPLGG(N)LLGEYTQLARECGLWLSLGG
lau Dog          FDFIARDPAETLRLSEPLGG(N)LLGEYTQLARECGLWLSLGG
lau Cat          FDFVARDPAETLRLSEPLGG(N)LLGEYTQLARECGLWLSLGG
lau Horse        FDFIARDPAETLRLSEPLDG(N)LLGEYTQLARECGLWLSLGG
lau Cow          FDFIARDPEETRRLSEPLSG(N)LLEEYTQLARECGLWLSLGG
lau Dolphin      FDFIARNPEETLHLSEPLGG(N)LLGEYTQLARECGLWLSLGG
lau Alpaca       FDFIARDPEETLRLSEPLSG(N)LLGEYTQLARECGLWLSLGG
lau Megabat      FDFIARDPAETLHLSEPLGG(K)LLGEYTQLARECGLWLSLGG
lau Microbat     FDFIARDPAETLRLSEPLGG(N)LLGEYAQLARECGLWLSLGG
lau Hedgehog     FDFIARDPDETLRLSEPLGG(N)LLGEYTQLARECGLWLSLGG
afr Elephant     FDFIARDPAETLHLSEPLGG(K)LLGEYTELARECGLWLSLGG
afr Tenrec       FDFIARDTTGTLHLSEPLSG(N)LLEKYTQLARECGLWLSLGG
xen Sloth        FDFIARDPAETLRLSEPLGG(S)LVGEYTQLARECGLWLSLGG
mar Opossum      FDFIARDPAETLQLSEPLGG(D)LVSRYAQLARECGLWISLGG
mar Wallaby      FDFIGRDSAETLRLAEPLGG(D)LVNRYAQLARECGIWLSLGG

NO 162
GN NOA1
IP IPI00385928
PE PQPTREKQLQELQ
MP 89
EX Wagner
CL apes
DE nitric oxide associated 1
SQ
pri Human        QERFLFPEYILDPEPQPTRE(K)QLQELQQQQEEEERQRQQRR
pri Chimpanzee   QERFLFPEYILEPEPEPTRE(K)QLQELQQQQEEEERQRQQRR
pri Orangutan    QERFLFPEYILEPEPEPTRE(K)QLQELQQQQKEEERQRQQRR
pri Gibbon       QERFLFPEYILEPEPEPTRE(K)QLQELQQQQEE-ERQRQQRR
pri Rhesus       QERFLFPEYIPEPELEPTRE(E)QLQELQQQQEEEERQRQQRR
pri Baboon       QERFLFPEYIPEPEPEPTRE(E)QLQELQQQQEEEERQRQQRR
pri Marmoset     QEHFLFPEYLLEPEPEPTPK(E)QLRELQQQQEEEEQQRQQRQ
eua Mouse        EERFLFPEYV----PERTPE(E)ELRELQQLQQEKERERLQQR
eua Rat          EERFLFPEYV----PERTPE(E)ELRELQQLQQEKERQRLQRR
eua Kangaroo_rat EERFLFPEYVVEPASAPTPE(E)ELRELQQRLEEEERQRLRRR
eua Rabbit       TERFLFPEYTPEPEPPPTPE(E)LLRALREREQEQERRRAERR
eua Pika         EEQFLFPEYTPEPEPEPSPA(E)LLLELQRQEQEKERRRLQRQ
lau Dog          EEHFLFPEYVSEPEPAPTVE(E)QLRELQQRQEEEERRKR---
lau Horse        EERFLFPEYVPEPEPEPTPE(E)QLRALQQRQEEEERQQQQRR
lau Cow          EESFLFPEYVLDSESEATLQ(D)QLRELQQRQE--ERRIEQQR
lau Dolphin      EESFVFPEYVPDSEPAVTLQ(D)QLRELQQRQE--ERRVLQRQ
lau Alpaca       ED-FLFPEYVPEPQPALSLQ(D)QLRELKRRGQ---RQKPQRR
lau Megabat      EERFLFPEYVPEPEPALTLE(E)QLQELQQQQEEDERRKQQQR
lau Hedgehog     QEHFFFPEYVLEPEPEPNLE(D)QVRELLQRQEEEERQQQQRR
afr Elephant     EEQFLFPEYVAEPEPAPTPE(E)ELQQQLQRQEEEEREKQQRR
afr Rock_hyrax   EESFLFPEYVEEPEPAPTPE(E)LLHRQQQRQEEEQRQKQQRQ
afr Tenrec       EERFVFPEYVPEPEPWPTVE(S)ELQQARQRQEDEERRKQQLR
xen Sloth        EERFLFPEYIPEPKPEPTPG(E)LLQELQQPQEEEERQRQRRG

NO 163
GN NOL11
IP IPI00303813.5
PE KNQSLVKSLLLKA
MP 247
EX Kim
CL African great apes
DE nucleolar protein 11
SQ
pri Human        IYETLIPIRPADPEKNQSLV(K)SLLLKAVVSGNARNGVALTA
pri Chimpanzee   IYETLIPIRPADPGKNQSLV(K)SLLLKAVVSGNARNGVALTA
pri Gorilla      IYETLIPIRPADPEKNQSLV(K)SLLLKAVVSGNARNGVALTA
pri Orangutan    IYETLIPIRPTDPEKNQSLV(R)SLLLKAVVSDNTQNGVALTA
pri Gibbon       IYETLIPIRPTDPEKNQSLV(R)SLLLKAVVSGNARNGVALTA
pri Rhesus       IYETLIPIRPTDPEKNQTLV(R)SLLLKAVVSGNTRNGVALTA
pri Baboon       IYETLIPIRPTDPEKNQTLV(R)SQLLKAVVSGNTRNGVALTA
pri Marmoset     IYETLIPIRPTDPEKNQSLV(R)SLLFKAVVSGDARHGVALTV
pri Tarsier      VYETLIPVYPTDPEENQRVV(S)SLLLRAVVSGSAPSGVALTV
pri Bushbaby     IYETLIPIHPTDAEQNQRVV(R)SLLLNAVVSGNARKGIALTV
pri Mouse_lemur  VYETLIPIRPTDPEKNQRVV(R)SLLLKAVVSGNARNAVALTV
eua Treeshrew    VYESLIPVHPSDAEENQRVV(R)SVLLKTVVSGSARNGVALTV
eua Mouse        IYETLIPIYSSDTEQNQRLV(R)ALMLKSVVSGGVRNGVALTI
eua Rat          VYETLVPIYSSDTENNQRLV(R)ALMLKTVVSGSARNGSALTI
eua Guinea_pig   IYETLIPIYPSDTEKNQRLV(R)SLLLKAVVSGIPPNGVTLTI
eua Rabbit       IYESLIPVYPSDGEKNQKVV(R)SLLLKTVVSGSAQKSVALTI
eua Pika         VYESVIPVYPSDTDKNQKVV(R)SLLLKTVVSGSSQKEVALTV
lau Dog          IYKSLIPIHPNDPEKNQRVV(R)SLLLKTVVSGNVRNGIALTV
lau Horse        IYETLIPIRPNDPEKNQRVV(R)SLLLKAVVSGNARNGVTLTV
lau Cow          VYETLIPIHPSEPEKNQRLV(Q)SQLLKSVVSGSARNGVALAI
lau Dolphin      VYETLIPVHPSDPEKNQRVV(P)SLLLKSVVSGNARSGVALTI
lau Alpaca       VYETLIPIHPSDPEKNQRVV(G)SLLLKSAVSGNARSGVGLTV
lau Megabat      IYETLIPIHPSDPDKNQRVV(R)SLLLKTVVAGNARNGVALTI
lau Microbat     IYETLIPIHPSDPEKNQRVV(R)SLLLKTVVSGNARXXXALSV
lau Hedgehog     VYEALIPTHPSDPEKTQRVV(R)SLLLKGIVSGNARNGAAVAT
lau Shrew        MYEALIPLHPSDPEKSQQVV(R)SLVLKDVMLCSSRSGAALAI
afr Elephant     IYETLIPTHPSDPEKNQVVV(R)SLLLKAVVSGNAPNGVALSV
afr Rock_hyrax   IYETLIPALPNDPEKKQEVV(R)SLLLNAIVSXXXRNGVALSV
afr Tenrec       IYETLLPTHPSDPGKDQVAV(R)SLVIKGIVSXEAQSGVALSI
mar Opossum      IYETLIPLNHGKPEEKKVLV(T)SLLLKTYVSGDVLKGVSFAI
mar Wallaby      IYETLIPLNQSKLEEKQVLV(T)SLLLKTYVSGDVLKGVSFAI

NO 164
GN NRM
IP IPI00217557
PE YWEPIPKGPVLWE
MP 121
EX Wagner
CL African great apes
DE nurim (nuclear envelope membrane protein)
SQ
pri Human        YVACTALALQLVMRYWEPIP(K)GPVLWEARAEPWATWVPLLC
pri Chimpanzee   YVACTALALQLVMRYWEPIP(K)GPVLWEARAEPWATWVPLLC
pri Gorilla      YVACTALALQLVMRYWEPIP(K)GPVLWEARAEPWATWVPLLC
pri Orangutan    YVACTALALQLVMRYWEPVP(R)GPVLWEARAEPWATWVPLLC
pri Gibbon       YVACTALALQLVMRYWEPIP(R)GPVLWEARAEPWATWVPLLC
pri Rhesus       YVACTALALQLVMRYWEPVP(R)GPVLWEAQAEPWATWVPLLC
pri Baboon       YVACTALALQLVMRYWEPVP(R)GPVLWEAQAEPWATWVPLLC
pri Marmoset     YVACTALALQLVMRYWEPIP(R)GPVLWEARAEPWATWVPLLC
pri Tarsier      YVACTALALQLVMRYWEPIP(R)GPVLWEARTEPWATWVPLLC
pri Mouse_lemur  YVACTALALQLVMRCWEPVP(R)GPVLWEARAEPWATWVPLLC
eua Treeshrew    YVAFTALALQLVMRCWEPVP(R)GPVLWEARAEPWATWVPLLC
eua Mouse        YVACTALALQLVMRYWETTP(R)GPVLWEARAEPWATWVPLLC
eua Rat          YVACTALALQLVMRYWEATP(R)GPVLWEARAEPWATWVPLLC
eua Pika         YVACTALALQVVMRYWEPVP(R)GPVLWEARVEPWATWVPLLC
lau Dog          YVACTALALQLVMRYWEPVP(R)GPVLWEARAEPWATWVPLLC
lau Cat          YVACTALALQLVMRYWEPVP(R)GPVLWEARAEPWATWVPLLC
lau Horse        YVACTALALQLVMRYWEPVP(R)GPVLWEARAEPWATWVPLLC
lau Cow          YVACTALALQLVMRYWEPVP(R)GPVLWETRTEPWATWVPLLC
lau Dolphin      YVACTALALQLVMRYWEPVP(R)GPVLWEARAEPWATWVPLLC
lau Alpaca       YVACTALALQL-MRYWEPVP(R)GPVLWEARAEPWATWVPLLC
lau Megabat      YVACTALALQLVMRYWESVP(R)GPVLWAARTEPWATWVPLLC
afr Elephant     YVACTALALQLVMRYWEPVP(R)GPVLWEARAEPWATWVPLLC
afr Rock_hyrax   YVACTALALQLVMRYWEPVP(R)GPVLWEARTEPWATWVPLLC
mar Wallaby      YVACTALALQVIMRYWEPVP(E)GPVLWEAQSEPWVTWVPLLC

NO 165
GN NSFL1C
IP IPI00100197.3
PE AYVAGEKRQHSSQ
MP 172
EX Kim
CL catarrhines
DE NSFL1 (p97) cofactor (p47)
SQ
pri Human        GGGYRLGAAPEEESAYVAGE(K)RQHSSQDVHVVLKLWKSGFS
pri Chimpanzee   GGGYRLGAAPEEESAYVAGE(K)RQHSSQDVHVVLKLWKSGFS
pri Gorilla      GGGYRLGAAPEEESAYVAGE(K)RQHSSQDVHVVLKLWKSGFS
pri Orangutan    GGGYRLGAAPEEESAYVAGE(K)RQHSSQDVHVVLKLWKSGFS
pri Gibbon       GGGYRLGAAPEEESAYVAGE(K)RQHSSQDVHVVLKLWKSGFS
pri Rhesus       GGGYRLGAAPEEESAYVAGE(K)RQHSSQDVHVVLKLWKSGFS
pri Baboon       GGGYRLGAAPEEESAYVAGE(K)RQHSSQDVHVVLKLWKSGFS
pri Marmoset     GGGYRLGAAPEEESAYVAGE(R)RQHSSQDVHVVLKLWKSGFS
pri Tarsier      GGGYRLGAAPEEESAYVAGE(R)RQHSSQDVHVVLKLWKSGFS
pri Mouse_lemur  GGGYRLGAAPEEESAYVAGE(R)RQHSGQDVHVVLKLWKTGFS
eua Treeshrew    GGGYRLGAAPEEESAYVAGE(R)RRHSSQDVHVVLKLWKSGFS
eua Mouse        GGGYRLGAAPEEESAYVAGE(R)RRHSGQDVHVVLKLWKTGFS
eua Rat          GGGYRLGAAPEEESAYVAGE(R)RRHSGQDVHVVLKLWKTGFS
eua Kangaroo_rat GGGYRLGAAPEEESAYVAGE(R)RRHSSQDVHIVLKLWKSGFS
eua Guinea_pig   GGGYRLGAAPEEESAYVAGE(R)RQHSSQDVHVVLKLWKSGFS
eua Squirrel     GGGYRLGAAPEEESAYVAGE(R)RRHSSQDVHVVLKLWKSGFS
eua Rabbit       GGGYRLGATPEEESAYVAGE(R)RRHSSQDVHVVLKLWKSGFS
eua Pika         GGGYRLGAAPEEESAYVAGE(R)RRHSGQDVHVVLKLWKSGFS
lau Dog          GGGYRLGAAPEEESAYVAGE(R)RRHSGQDVHVVLKLWKSGFS
lau Cat          GGGYRLGAAPEEESAYVAGE(R)RRHSGQDVHVVLKLWKSGFS
lau Horse        GGGYRLGAAPEEESAYVAGE(R)RRHSSQDVHVVLKLWKSGFS
lau Cow          GGGYRLGAAPEEESAYVAGE(R)RRHSGQDVHVVLKLWKTGFS
lau Dolphin      GGGYRLGAAPEEESXYVAGE(R)RRHSGQDVHVVLKLWKSGFS
lau Alpaca       GGGYRLGAAPEEESAYVAGE(R)RRHSGQDVHVVLKLWKSGFS
lau Megabat      GGGYRLGAAPEEESAYVAGE(R)RRHSGQDVHVVLKLWKSGFS
lau Microbat     GGGYRLGAAPEEESAYVAGE(R)RRHSSQDVHIVLKLWKSGFS
lau Shrew        GGGYRLGAAPEEESAYVAGE(R)RRHSGQDVHVVLKLWKSGFS
afr Elephant     GGGYRLGAAPEEESAYVAGE(R)RRHSGQDVHVVLKLWKSGFS
afr Rock_hyrax   GGGYRLGAAPEEESAYVAGE(R)RRHSGQDVHVVLKLWKSGFS
afr Tenrec       GGGYRLGAAPEEESAYVAGE(R)RRHSGQDVHVVLKLWKSGFS
xen Armadillo    GGGYRLGAAPEEESAYVAGE(R)RRHSAQDVHVVLKLWKTGFS
xen Sloth        GGGYRLGAAPEEESAYVAGE(R)RRHSAQDXXXXXXXXXXXXX
mar Opossum      GGGYRLGAAPEEESAYVAGE(R)RSYSGQDVHIVLKLWKSGFS

NO 166
GN NSMCE1
IP IPI00184884
PE WPHEIPKVFDPEK
MP 241
EX Wagner
CL great apes
DE non-SMC element 1 homolog (S. cerevisiae)
SQ
pri Human        QSNAEPRCPHCNDYWPHEIP(K)VFDPEKERESGVLKSNKKSL
pri Chimpanzee   QSNAEPRCPHCNDYWPHEIP(K)VFDPEKERESGVLKSNKKSL
pri Gorilla      QSNAEPRCPHCNDYWPHEIP(K)VFDPEKERESGVLKSNKKAL
pri Orangutan    QSNAEPRCPHCNDYWPHEIP(K)VFDPEKERESGVSKSNKKSL
pri Gibbon       QSNAEPHCPHCNDYWPHEIP(E)VFDPEKERESGISKSNKKSL
pri Rhesus       QSNTEPRCPHCNDYWPHEIP(E)VFDPEKQREAGISKSNKKSL
pri Baboon       QSNAEPRCPHCNDYWPHEIP(E)VFDPEKERESGISKSNKKSL
pri Marmoset     RSNAEPRCPHCNDYWPHEIP(E)VFDPEKEREPGISKSNRKSL
pri Bushbaby     QSNSEPRCPHCNDYWPHDIP(E)VFNPEKEREAGTSKSNKKSL
pri Mouse_lemur  QSNSEPHCPHCNDYWPHEIP(E)VFDPEKEREAGISKSNRRSL
eua Treeshrew    RSNSEPRCPHCNDYWPHEIP(E)VFDPEKEREANVSKSSKKSS
eua Mouse        QSIPEPHCPHCNDYWPHDIP(E)VYNPEKEREAGISKSSRKSL
eua Rat          QSTAEPRCPHCNDYWPHDIP(E)VFDPEKEREAGISKSSRKSL
eua Kangaroo_rat QSTAEPRCPHCNDYWPHEIP(E)VFDPEKERDAGVSRTSRKSL
eua Guinea_pig   QSNAEPHCPHCNDYWPHEIP(E)VFDPEKQREAAASKSSQKSM
eua Rabbit       QSNPEPRCPHCNDYWPHEVP(D)VFDPEKEREAGVSKPTKKSS
eua Pika         QSNPEPRCPHCNDYWPHEIP(E)VFDPEKERESSISRPAKRPL
lau Dog          QSNSEPRCPHCNDYWPHEIP(E)VFDPEKEREASTSKANRKSL
lau Horse        QSNSEPRCPHCNDYWPHEIP(E)VFDPEKEREAGISKSNKKPL
lau Cow          QSSSEPHCPHCNDYWPHEVP(E)VFDPEKERETGMSRSNKRPS
lau Dolphin      QSHSEPRCPHCNDYWPHEIP(E)VFDPEKEREASISKSNKRSL
lau Megabat      QSNSEPHCPHCNDYWPHEIP(E)IFDPEKDREASVSKPNKKSS
lau Microbat     QSNSEPRCPNCNDYWPHEIP(E)VFDPEKERGAGLSKPSRKSL
lau Hedgehog     QANPEPHCPHCNDYWPHEIP(E)VFDPEKEGEPTISRPHRKSL
afr Elephant     RSSSEPRCPHCNDCWPHEIP(E)IFNPEKEREAGTSSSNKKSL
afr Tenrec       QSSPEPHCPHCNDYWPHEIP(E)VFNQEKEEEAGFSKSSKKSL
xen Armadillo    KSNSEPRCPHCNDYWPHEIP(E)VFDPEKDREAGISRSNKKSL

NO 167
GN NUDT22
IP IPI00031645.4
PE IWETRLKAQPWLF
MP 57
EX Kim
CL simians
DE nudix (nucleoside diphosphate linked moiety X)-type motif 22
SQ
pri Human        DRRPLPGGDEAITAIWETRL(K)AQPWLFDAPKFRLHSATLAP
pri Chimpanzee   DRRPLPGGDEAITAIWETRL(K)AQPWLFDAPKFRLHSATLAP
pri Gorilla      DRRPLPGGDEAITAIWETRL(K)AQPWLFDAPKFRLHSATLAP
pri Orangutan    DRRPLPCGEEAITAIWETRL(K)AQPWLFDAPKFRLHSATLAP
pri Gibbon       DRRPLPGGDEAITAIWETRL(K)AQPWLFDAPKFRLHSATLAP
pri Rhesus       DRRPLPGGDEAITAIWETRL(K)AQPWLFNAPKFRLHSATLAP
pri Baboon       DRRPLPGGDEAITAIWETRL(K)AQPWLFNAPKFRLHSATLAP
pri Marmoset     DRRPLPGGDEAITAIWETRL(K)AQPWLFDAPKFRLHSATLAP
pri Bushbaby     NRRPLPGGDKAITAIWETRL(Q)AQPWLFDAPKFRLHSASLVP
eua Treeshrew    DRRPLPGGDKAITAIWETRR(Q)AQPWLFDAPKFRLQSATLAP
eua Mouse        DRRPLPGGDKAITAIWETRQ(Q)AQPWIFDAPKFRLHSATLVS
eua Rat          DRRPLPEGDKTITAIWETRL(Q)AQPWIFDAPKFRLHSATLAS
eua Kangaroo_rat DRRFLPGDDKTITAIWETRL(Q)SQPWLFDAPKFRLHSATLAP
eua Guinea_pig   DRHPLPGEDKTITAIWESRV(Q)TQPWLFNAPKFRLHSATLES
eua Rabbit       DRRPLPGGDKAITAIWESRL(Q)TQPWLFDAPKFRLHTATLAP
lau Dog          DRRPLPGGDEAIAAIWESRL(Q)AQPWLFDAPKFRLHSATLAP
lau Cat          DRRPLPGGDQAIASIWESRL(Q)AQPWLFDAPKFRLHSAILAP
lau Horse        DRRPLPGGDNAISAAWESRL(Q)AQPWLFDAPKFRLHSATLAP
lau Cow          DRRPLPGGDKAIIAIWESRL(Q)AQPWLFNAPKFRLHSATLAP
lau Dolphin      DRRPLPGGDKAITAVWESRL(Q)AQPWLFDAPKFRLHSATLVP
lau Megabat      DRRPLPEEDKAIAAIWESRL(Q)AQPWLFDASKFRLHSVTLAP
lau Hedgehog     DLHQLPZGHDAIAAAWETRL(P)DQPWLYDTPEFRLHAATLAN
lau Shrew        DRRPLPGGDEAISAIWESRL(Q)TQPWLFDAPKFRLHSAVLEP
afr Elephant     DRRPLPGGDKAIATVWENRL(Q)AQPWLFDAPKFRLHSATLAP
afr Rock_hyrax   DRRPLPGGDEAIAAVWENRL(Q)AQPWLFDAPKFRLHSATLEP
afr Tenrec       DRRPLPGGDEAIATVWENRL(Q)AQPWLFDAPKFRLHSATLAP
xen Armadillo    DRHPLPGGDKDIAAIWETRL(Q)AQPWLFDAPKFRLHSAALAP
mar Wallaby      DRRPLPGGEDSITAIWDARR(Q)AQPWLFNAPKFRLHSAVLGP

NO 168
GN NUMA1
IP IPI00872028
PE ERDAALKQLEALE
MP 610
EX Wagner
CL great apes
DE nuclear mitotic apparatus protein 1
SQ
pri Human        QLATAAEEREASLRERDAAL(K)QLEALEKEKAAKLEILQQQL
pri Chimpanzee   QLATAAEEREASLRERDTAL(K)QLEALEKEKAAKLEILQQQL
pri Gorilla      QLATAAEEREASLRERDAAL(K)QLEALEKEKAAKLEILQQQL
pri Orangutan    QLATAAEEREASLRERDAAL(K)QLEALEKEKAAKLETLQQQL
pri Gibbon       QLATAAEEREASLRERDAAL(Q)QLEALEKEKAAKLEILQQQL
pri Rhesus       QLATAAEEREASLRERDAAL(Q)QLEALEKEKAAKLEILQQQL
pri Baboon       QLATAAEEREASLRERDAAL(Q)RLEALEKEKAAKLEILQQQL
pri Marmoset     TLATAAEEREAFLRERDAAL(Q)QLEALEKEKATKLEILQQQL
pri Tarsier      QLATAAQEREASLRERDAAR(Q)QLEALEKETAAKLEALQQQL
pri Bushbaby     QLATAAAELKASLRERDAMR(Q)QLEVWEKEKAAKLEILQQQL
pri Mouse_lemur  QLAAAAAELEAALKERDAAH(Q)KLEALETEKASKLEILKQQL
eua Treeshrew    QLATATEEREASRKERDGAL(Q)QLAALEKEKAARLEVLQQQL
eua Mouse        QLAIVAEAREASLRERDTAR(Q)QLETVEKEKDAKLESLQQQL
eua Rat          QLATIVEAREASVRERDAAR(Q)QLETLEKEKDAKLESLQQQL
eua Kangaroo_rat QLAKAADVREAALRERDATR(Q)QLETLEKEKAAKLEILQQQL
eua Guinea_pig   QLATAAKAQEVSLRERDAAC(L)QLETLKKEKAAKLEILQQQL
eua Squirrel     QLATVAEAREAFLRERDTAR(Q)QLQALEKEKAANLEILQQQL
eua Rabbit       QLARAAEEREAHLRERDSVL(Q)QLEALGQEKAAALETLQQQL
eua Pika         QLARAAEEREASARERDTAL(Q)QLEALGKEKAAELEALQQQL
lau Dog          RLATAAEERQAALKERDSAL(Q)QLEALEKEKTAKLEVLQQQL
lau Cat          RLAAAAEERQASLRERDSAL(Q)QLEVLEKEKTAKLEVLQQQL
lau Horse        QLATAAEEQEVSLRERDSAL(Q)QLEALEKEKATKLEVLQQQL
lau Cow          QLAAAAEEREAALRERDTAL(Q)QLGAVEKEKAAELDVLQEQL
lau Dolphin      QLAAAAEEREASLRERDAAL(Q)QLEALGKEKAAELEVLQQQL
lau Megabat      QLATATEEREASLRERDSAL(Q)RLEALEKEKAAKLEVLQQQL
lau Microbat     QLAIAAEEREASLREKDSVL(Q)QVEALEKEKAAKLEVLQQQL
lau Hedgehog     QLAAAALKREASIREKDSAL(Q)QLQVLEKEKAVKLEILQQQL
lau Shrew        QLAAATAKREAAFRERDSAL(Q)QLKALE-EKAAKLESLQQQL
afr Elephant     QLATVSKKQEASLRQRDTAL(Q)QLEALEKEKAAQLEILQQQL
afr Rock_hyrax   QLATVAKKQEASLRERDTAL(Q)QLEALEKEKAVKLEILQQQL
afr Tenrec       QLATAAREREASLRERDTAL(R)QLEAEDKEKAAQLEVLQQQL
xen Armadillo    QLTSAAEEREASLRERDAAL(Q)KLQALEEEKTTKLESLQQQL
xen Sloth        HLATAAKEGEAFLRERDTAL(Q)KLEALEKEKNAKLESLQQQL
mar Opossum      QLAAAMEKYQEAQQERDAAL(K)QLKELEKTKAVELGALQQQL
mar Wallaby      QLAAATEECKEARQEKDVAL(K)QLEDLQKEKAIELGALQQQL

NO 169
GN NUP205
IP IPI00783781
PE YKDIWHKVGNALW
MP 23
EX Wagner
CL catarrhines
DE nucleoporin 205kDa
SQ
pri Human        TPLAVNSAASLWGPYKDIWH(K)VGNALWRRQPEAVHLLDKIL
pri Chimpanzee   TPLAVNSAASLWGPYKDIWH(K)VGNALWRRQPEAVHLLDKIL
pri Gorilla      TPLAVNSAASLWGPYKDIWH(K)VGNALWRRQPEAVHLLDKIL
pri Orangutan    XXXXXXXXASLWGPYKDIWH(K)VGNALWRRQPEAVHLLDKIL
pri Gibbon       TPLAVNSAASLWGPYKDIWH(K)VGNALWRRQPEAVHLLDKIL
pri Rhesus       TPLAVNSAASLWGPYKDIWH(K)VGNALWRRQPEAVHLLDKIL
pri Baboon       TPLAVNSAASLWGPYKDIWH(K)VGNALWRRQPEAVHLLDKIL
pri Marmoset     TPLAVNSAASLWGPYKAIWH(A)VGNAVYRRQPEAVHLLDKIL
pri Tarsier      TPLAVNSAASLWGPYKDIWQ(T)VGNALWRRQPEAVHLLDMIL
pri Bushbaby     TLLAVNSAASLWGPYKDIWQ(T)VGNVLWRRQPEAVHLLDMIL
eua Mouse        ALLAVNSAASLWGPYKDIWQ(T)VGSALWRRQPEAVHLLDMIL
eua Rat          ALLAVNSAASLWGPYKDIWQ(T)VGNALWRRQPEAVHLLDMIL
eua Kangaroo_rat TLLAVNSAASLWGPYKDIWQ(T)VGNALWRRQPEAVHLLDMIL
eua Guinea_pig   TPLAVNSAASLWGPYKDVWQ(T)VGNALWRRQPEAVHLLDVIL
eua Squirrel     TLLAVNSAASLWGPYKDIWQ(T)VGNALWRRQPEAIHLLDMIL
eua Rabbit       TLLAVNSAASLWGPYKDIWQ(T)VGNALWRRQPEAVHLLDMIL
lau Cat          TLLAVNSAASLWGPYKDIWQ(T)VGNALWRRQREAVHLLDMIL
lau Horse        TLLAVNSAASLWGPYKDIWQ(T)VGNALWRRQPEAVHLLDMIL
lau Cow          TLLAVNSAASLWGPYKDIWQ(T)VGNALWRRQPEAVHLLDMIL
lau Dolphin      TLLAVNSAASLWGPYKDIWQ(T)VGNALWRRQPEAVHLLDMIL
lau Alpaca       TLLAVNSAASLWGPYKDIWQ(T)VGNALWRRQPEAVHLLDMIL
lau Megabat      TLLAVNSAASLWGPYKDIWQ(M)VGNALWRRQPEAVHLLDMIL
lau Microbat     TLLAVNSAASLWGPYKDIWQ(T)VGNVLWRRQPEAVHLLDMIL
lau Hedgehog     TLLAVNSAASLWGPYKDIWQ(T)VGNALWRRQPEAVHVLDMIL
afr Elephant     TPLAVNSAASLWGPYKDIWQ(T)VGNALWKRQPEAVHLLDMIL
xen Armadillo    XXXXXXXXASLWGPYKDIWQ(T)VGNALWRRQPEAVHLLDMIL
mar Opossum      ALLAVNSAASLWGPYKDIWQ(T)VGNAIWRRQPEAVHLVDLIL
mar Wallaby      XXXXXXXXASLWGPYKDIWQ(T)LGNAIWRRQPEAVHLLDLIL
pro Platypus     XXXXXXXXVSMWGPYKDIWQ(S)AGNAIWKRQPEAVHVVDLIL

NO 170
GN NUP205
IP IPI00783781
PE AVHLLDKILKKHK
MP 41
EX Wagner
CL simians
DE nucleoporin 205kDa
SQ
pri Human        WHKVGNALWRRQPEAVHLLD(K)ILKKHKPDFISLFKNPPKNV
pri Chimpanzee   WHKVGNALWRRQPEAVHLLD(K)ILKKHKPDFISLFKNPPKNV
pri Gorilla      WHKVGNALWRRQPEAVHLLD(K)ILKKHKPDFISLFKNPPKNV
pri Orangutan    WHKVGNALWRRQPEAVHLLD(K)ILKKHKPDFISLFKNPPKNV
pri Gibbon       WHKVGNALWRRQPEAVHLLD(K)ILKKHKPDFISLFKNPPKNV
pri Rhesus       WHKVGNALWRRQPEAVHLLD(K)ILKKHKPDFISLFKNPPKNV
pri Baboon       WHKVGNALWRRQPEAVHLLD(K)ILKKHKPDFISLFKNPPKNV
pri Marmoset     WHAVGNAVYRRQPEAVHLLD(K)ILKKHKPDFISLFKNPPKNV
pri Tarsier      WQTVGNALWRRQPEAVHLLD(M)ILKKHKPDFISLFRNPXXXX
pri Bushbaby     WQTVGNVLWRRQPEAVHLLD(M)ILKKHKPDFISLFRNPXKNV
eua Mouse        WQTVGSALWRRQPEAVHLLD(M)ILKKHKPDFISLFKNPPKNV
eua Rat          WQTVGNALWRRQPEAVHLLD(M)ILKKHKPDFISLFKNPPKNV
eua Kangaroo_rat WQTVGNALWRRQPEAVHLLD(M)ILKKHKPDFISLFRNPXXXX
eua Guinea_pig   WQTVGNALWRRQPEAVHLLD(V)ILKKHRPDFISLFKNPPKNV
eua Squirrel     WQTVGNALWRRQPEAIHLLD(M)ILKKHKPDFISLFKNPXXXX
eua Rabbit       WQTVGNALWRRQPEAVHLLD(M)ILKKHKPDFISLFRNPPKNV
lau Dog          WQTVGNALWRRQPEAVHLLD(M)ILKKHKPDFISLFRNPPKNV
lau Cat          WQTVGNALWRRQREAVHLLD(M)ILKKHKPDFISLFRNPPKNV
lau Horse        WQTVGNALWRRQPEAVHLLD(M)ILKKHKPDFISLFKNPXXXX
lau Cow          WQTVGNALWRRQPEAVHLLD(M)ILKKHKPDFISLFRNPPKNV
lau Dolphin      WQTVGNALWRRQPEAVHLLD(M)ILKKHKPDFISLFRNPPKNV
lau Alpaca       WQTVGNALWRRQPEAVHLLD(M)ILKKHKPDFISLFRNPPKNV
lau Microbat     WQTVGNVLWRRQPEAVHLLD(M)ILKKHKPDFISLFRNPPKNV
lau Hedgehog     WQTVGNALWRRQPEAVHVLD(M)ILKKHKPDFISLFRNPPKNV
lau Shrew        WQTVGNALWRRQPEADHLLD(M)ILKKHKPDFISLFRNQPKNV
afr Elephant     WQTVGNALWKRQPEAVHLLD(M)ILKKHKPDFISLFKNPPKNV
mar Opossum      WQTVGNAIWRRQPEAVHLVD(L)ILKKHKPDFISLFKNPAKNV
mar Wallaby      WQTLGNAIWRRQPEAVHLLD(L)ILKKHKPDFISLFRNPAKNV

NO 171
GN NUP205
IP IPI00783781.1
PE LPLLTEKQYIATI
MP 304
EX Kim, Wagner
CL simians
DE nucleoporin 205kDa
SQ
pri Human        IEQSTEERDDMIHQLPLLTE(K)QYIATIHSRLQDSQLWKLPG
pri Chimpanzee   IEQSTEERDDMIHQLPLLTE(K)QYIATIHSRLQDSQLWKLPG
pri Gorilla      IEQSTEERDDMIHQLPLLTE(K)QYIATIHSRLQDSQLWKLPG
pri Orangutan    IEQSTEERDDMIHQLPLLTE(K)QYIATIHSRLQDSQLWKLPG
pri Gibbon       IEQSTEERDDMIHQLPLLTE(K)QYIATIHSRLQDSQLWKLPG
pri Rhesus       IEQSTEERDDMIHQLPLLTE(K)QYIATIHSRLQDSQLWKLPG
pri Baboon       IEQSTEERDDMIHQLPLLTE(K)QYIATIHSRLQDSQLWKLPG
pri Marmoset     IEQITEERDDMIHQSPLLTE(K)QYIATIHSRLQDSQLWKLPG
pri Tarsier      IEQSTEERDDIINQLPLLTE(R)QYIATIHSRLQDSQPWKLPG
pri Mouse_lemur  IEQSTEERDDMIHQLPLLTE(R)QYIATIHSRLQDSQPWKLPG
eua Treeshrew    IEQTTEERDDMIHQLPLLTE(R)QYIAXXXXXXXXXXXXXXXX
eua Mouse        IDQSTEERDDMIHHLPLLTE(R)QYVSTIHSRLQDSQPWKLPG
eua Rat          IEQSTEERDDMIHHLPLLTE(R)QYIATIHSRLQDSQPWKLPG
eua Kangaroo_rat IEQSTEERDDVIHQLPLLTE(R)QYIATIHSRLQDSQPWKLPG
eua Guinea_pig   IEQSTEERDDVIHQLPLLTE(R)QYIATIHSRLQDSQPWKLPG
eua Rabbit       IEQSTEDRDDVIHQLPLLTE(R)QYIATIHSRLQDSQPWKLPG
eua Pika         IEQSAEDREDVIHQLPLLTE(R)QYIGTIHSRLQDCQAWKLPG
lau Dog          LEQSTEERDDMIHQLPLLTE(R)QYIATIHSRLQDSQLWKLPG
lau Horse        IEQSTEERDDMIHQLPLLTE(K)QYIATIHSRLQDSQPWKLPG
lau Cow          IEESTEERDDMIHQLPLLTE(R)QYIATIHSRLQDSQPWKLPG
lau Dolphin      IEQSTEERDDMIHQLPLLTE(R)QYIATIHSRLQDSQPWKLPG
lau Alpaca       IEQSTEERDDMIHQLPLLTE(R)QYIATIHSRLQDSQPWKLPG
lau Megabat      LEQSTEERDDMIHQLPLLTE(R)QYIATVHSRLQDSQPWKLPG
lau Hedgehog     XXXXXXXXXXMIHQLPLLTE(R)QYIATIHSRLQDSQPWKLPG
lau Shrew        LEQSSEDRDDIIHQFPLLTE(R)QYIATIHSRLQDSQPWKLPG
afr Elephant     IEQSTEERDDMLHQLPLLTE(R)QYIATIHSRLQDSQLWKLPG
afr Rock_hyrax   LEQSTEERDDMLQQLPLLTE(R)HYIATVHARLQDSQPWKLPG
xen Armadillo    XXXXXXXXXXMIHQLPLLTE(R)QYIATIHSRLQDSQLWKLPG
mar Opossum      IEQGAEDREDIIQQLPLLTE(K)QYISSIHSRLQDSQPWKLPG
pro Platypus     IEQGTEDREDLIHQLPLLTE(R)QYIGSIHARLQDSQPWKLPG

NO 172
GN NUP205
IP IPI00783781.1
PE DNVEGDKVSKKDE
MP 1742
EX Kim
CL simians
DE nucleoporin 205kDa
SQ
pri Human        FGGSDRLRQFKFQDDNVEGD(K)VSKKDEIELAMQQICANVME
pri Chimpanzee   FGGSDRLRQFKFQDDNVEGD(K)VSKKDEIELAMQQICANVME
pri Gorilla      FGGSDRLRQFKFQDDNVEGD(K)VSKKDEIELAMQQICANVME
pri Orangutan    FGGSDRLRQFKFQDDNVEGD(K)VSKKDEIELAMQQICANVME
pri Gibbon       FGGSDRLRQFKFQDDNVEGD(K)VSKKDEIELAMQQICANVME
pri Rhesus       FGGSDRLRQFKFQDDNVEGD(K)VSKKDEIELAMQQICANVME
pri Baboon       FGGSDRLRQFKFQDDNVEGD(K)VSKKDEIELAMQQICANVME
pri Marmoset     FGGSDRLRQFKFQDDNVEGD(K)VSKKDEIELAMQQICANVME
pri Tarsier      FGGSDRLRQFKFQDDNVEGD(R)VSKKDEIELAMQQICANVME
eua Mouse        FGGADRLRQFKFQDNNAEGD(R)VSKKDEIELAMQQICANVME
eua Rat          FGGSDRLRQFKLQEDNVEGD(R)LSKKDEIELAMQQICANVME
eua Kangaroo_rat FGGADRLRQFKFQDDNVDRD(R)VNKKDEMELAMQQICANVME
eua Guinea_pig   FGGSDRLRQFKFQDDNVDGD(R)MSKKDEIELAMQQICANVME
eua Rabbit       FGGSDRLRQFKFQDDNVEGD(R)VSRKDEIELAMQQICANVME
lau Dog          FGGSDRLRQFKFQDENVEGD(R)VSKKDELELAMQQICANVME
lau Cat          FGGSDRLRQFKFQDDNVEGD(R)VSKKDELELAMQQICANVME
lau Horse        FGGSDRLRQFKFQDDNVEGD(K)VSKKDEIELAMQQICANVME
lau Cow          FGGSDKLRQFKCQDDNVEGD(R)VSRKDEIELAVQQICANVME
lau Dolphin      FGGSDRLRQFKFQDDNVEGD(R)VSKKDEIELAMQQICANVME
lau Megabat      FGGSDRLRQFKFQDDNVEGD(R)VSKKDEIELAMQQICANVME
lau Microbat     FGGSDRLRQFKFQDDNMEGD(R)VNKKDEIELAMQQICANVME
lau Hedgehog     FGGSDRLRQFKFQDDNVEGD(R)MNKKDEIELAMQQXXXXXXX
lau Shrew        FGGSDRLRQFKFQDDNVEGD(R)VNKKDEIELAMQQICANVME
afr Elephant     FGGSDRLRQFKFQDDNVEGD(R)VSKKDEIELAMQQICANVME
afr Rock_hyrax   FGGSDRLRQFKFQDDSVEGD(R)VGKKDEIELAMQQICANVME
afr Tenrec       FGGSDRLRQFKFQEDNVEGD(K)VSKKDEIELAMQQICANVME
xen Armadillo    FGGSDRLRQFKFQDDNVEGD(R)VSKKDEIELAMQQICANVME
xen Sloth        FGGSDRLRQFKFQDDNVEGD(R)VSKKDEIELAMQQXXXXXXX
mar Opossum      FGGSDRLRQFKFQ-DGVEGD(R)VNKKDEIELAMQQICANVME
pro Platypus     FGGSDRLRQFKLQDDNVEGD(R)VSKRDEIELAMQQICANVME

NO 173
GN PARP10
IP IPI00064457
PE NATVYGKGVYFAR
MP 928
EX Wagner
CL simians
DE poly (ADP-ribose) polymerase family, member 10
SQ
pri Human        DICAHGFNRSFCGRNATVYG(K)GVYFARRASLSVQDRYSPPN
pri Chimpanzee   DICAHGFNRSFCGRNATVYG(K)GVYFARRASLSVQDRYSPPN
pri Gorilla      DICAHGFNRSFCGRNATVYG(K)GVYFARRASLSVQDRYSPPN
pri Orangutan    DICAHGFNRSFCGRNATVYG(K)GVYFARRASLSVQDRYSPPN
pri Gibbon       DICAHGFNRSFCGRNATVYG(K)GVYFARRASLSVQDRYSPPN
pri Baboon       DICAHGFNRSFCGRNATVYG(K)GVYFAKRASLSVQDRYSPPN
pri Marmoset     DICAHGFNRSFCGRNATVYG(K)GVYFATRASMSVQDRFSPPN
pri Tarsier      DICAHGFNRSFCGRNGTLYG(Q)GVYFAKRASLSVQDRYSPPD
pri Mouse_lemur  DICAHGFNRSFCGRNGTLYG(Q)GVYFAKRASLSVQDQYSPPN
eua Mouse        DICAHGFNRSFCGRNGTLYG(Q)GVYFAKRASLSVLDRYSPPN
eua Rat          DICAHGFNRSFCGRNGTLYG(Q)GVYFAKRASLSVLDRYSPPN
eua Guinea_pig   EICAHGFNRSFCGRNGTLYG(Q)GVYFAKRASLSVQDRYSPPN
eua Squirrel     DICAFGFNRSFCGRNGTLYG(Q)GVYFAKRASLSVQDRYSPPN
lau Horse        DICARGFNRSFCGRNGTLYG(Q)GVYFAMRASLSVQDRYSPPN
lau Dolphin      DICAHGFNRSFCGRNGTLYG(Q)GVYFAKRASLSVLDRYSPPD
lau Megabat      DICTYGFNRSFCGRNGTLYG(Q)GVYFAKRASLSVQDRYSPPN
lau Microbat     EICAYGFNRSFCGRNGTLYG(Q)GVYFAKRASLSVQDRYSPPD
lau Hedgehog     DICLHGFNRSFCGRNGTLYG(Q)GVYFALRASLSVQDRYSPPD
afr Elephant     DICAHGFNRSFCGRNGTLFG(Q)GVYFAKRASLSVQDRYSPPN
afr Rock_hyrax   DICAHGFNRSFCGRNGTLYG(Q)GVYFAKRASLSVQDRYSPPD
afr Tenrec       DICTHGFNRSYCGRNGTLYG(Q)RVYFAKHASLSVQDRYSPPN
xen Sloth        DICAHGFNRSFCGRNGTLYG(Q)GVYFARRASLSVQDRYSPPD
mar Wallaby      DICRHGFNRSFCGRNATLYG(Q)GVYFAVKAEISIKDRYSPPD
pro Platypus     EICLHGFSRNFCGLNAALYG(Q)GVYFAVQAGLSVQDRYSPCD

NO 174
GN PCM1
IP IPI00001654.5
PE AEKPRNKKLPEEE
MP 1236
EX Kim
CL simians
DE pericentriolar material 1
SQ
pri Human        MAFPKPFESSSSIGAEKPRN(K)KLPEEEVESSRTPWLYEQEG
pri Chimpanzee   MAFPKPFESSSSIGAEKPRN(K)KLPEEEVESSRTPWLYEQEG
pri Gorilla      MAFPKPFESSSSIGAEKPRN(K)KLPEEEVESXRTPWLYEQEG
pri Orangutan    MAFPKPFESSSSIGAEKPRN(K)KLPEEEVESSRTPWLYEQEG
pri Gibbon       MAFPKPFESSSSVGAEKPRN(K)KLPEEEVESSR-PWLYEQEV
pri Rhesus       MAFPKPFESSSSIGAEKARN(K)KLPEEEVESSRTPWLYDQEG
pri Baboon       MAFPKPFESSSSIGAEKPRN(K)KLPEEEVESSRTPWLYDQEG
pri Marmoset     MAFPKPFESSSSIGAEKPRN(K)KLPEEEVDSSRTPWLYDQEG
pri Bushbaby     MAFPKPFESSSSVGAEKPRN(Q)KSPEEDVDSSRTPWLYDQEG
eua Treeshrew    MAFPKPFESSSSIGAEKQRS(Q)KQPEEEVENSRTQWLYDQEG
eua Mouse        MAFPKPFESSSSLGAEKQRN(Q)KQPEEEAENTKTPWLYDQEG
eua Rat          MAFPKPFESSSSLGAEKQRN(Q)KQPEEETENTKTPWLYDQEG
eua Guinea_pig   MAFPKPFESSSSIGAEKQRN(Q)KQPEEEAQNSKAPWLYEQEG
eua Rabbit       MAFPKPFESSSSIGAEKQRN(Q)KQSEEEMENSRTPWLYDQEG
lau Dog          MAFPKPFESSSSIGAEKQRN(Q)KQPGEEVENSRTAWLYDQEG
lau Horse        MAFPKPFESSSSIGAEKQRN(Q)KQPEEEVENSRTPWLHDQEG
lau Cow          MAFPKPFESSSSIGAEKQRN(Q)KQPEEEVENSRTPWFYDQEG
lau Dolphin      MAFPKPFESSSSVGAEKQRN(Q)KQPEEEVENSRTPWLYDQEG
lau Alpaca       MAFPKPFESSSSVGAEKQRN(Q)KQPEEEVENSRTPWLYDQEG
lau Megabat      MAFPKPFESSSSIGPEKQRN(Q)KQSEEEVENSRTPWLYAQEG
lau Microbat     MAFPKPFESNSSIGAEKQRN(Q)KQSTEEVENSRTPWLYDQEG
lau Hedgehog     MAFPKPFESSSSIGAEKQRS(Q)KHPEEEMENSRTPWLYDHEG
lau Shrew        MAFPKPFESSSSIGAEKPRN(Q)KQSEEEVENSRTRWLYDQEG
afr Elephant     MAFPKPFESSSSIGAEKQRN(Q)KQPEEEVENSRPPWLYDQEG
afr Rock_hyrax   MAFPKPFESSSSVGAEKQRN(Q)NQPEEEIESSRQPWLYDQEG
afr Tenrec       MAFPKPFESSSSVGAEKQRN(Q)KQPEEEVENSRPPWLYGQEG
xen Sloth        MAFPKPFESSSSIGAEKQRN(Q)KQPEEEVENSRSPWLYDQEG
pro Platypus     VAFPKPFESNSSLGAEKQRN(T)KPPEEEADPPRPAGLADQEG

NO 175
GN PDCD5
IP IPI00023640.3
PE GLIEILKKVSQQT
MP 97
EX Kim, Wagner
CL simians
DE programmed cell death 5
SQ
pri Human        MARYGQLSEKVSEQGLIEIL(K)KVSQQTEKTTTVKFNRRKVM
pri Chimpanzee   MARYGQLSEKVSEQGLIEIL(K)KVSQQTEKTTTVKFNRRKVM
pri Gorilla      MARYGQLSEKVSEQGLIEIL(K)KVSQQTEKTTTVKFNRRKVM
pri Orangutan    MARYGQLSEKVSEQGLIEIL(K)KVSQQTDKTTTVKFNRRKVM
pri Gibbon       MARYGQLSEKVSEQGLIEIL(K)KVSQQTEKTTTVKFNRRKVM
pri Rhesus       MARYGQLSEKVSEQGLIEIL(K)KVSQQTEKTTTVKFNRRKVM
pri Baboon       MARYGQLSEKVSEQGLIEIL(K)KVSQQTEKTTTVKFNRRKVM
pri Marmoset     MARYGQISEKVSEQGLIEIL(K)KVSQQTEKTTTVKFNRRKVM
pri Mouse_lemur  MARYGQLSGKVSEQGLIEIL(E)KVSQQTEKKTTVKXXXXXXX
eua Treeshrew    MARYGQLSGXVSEQGLIEIL(E)KVSQPTEKKTTVKFNRRKVM
eua Mouse        MARYGQLSGKVSEQGLIEIL(E)KVSQQTEKKTTVKFNRRKVM
eua Rat          MARYGQLSGKVSEQGLIEIL(E)KVSQQTEKKTTVKFNRRKVM
eua Kangaroo_rat MARYGQLSGKVSEQGLVEIL(E)KVSQQTEKKTTVKFNRRKVM
eua Guinea_pig   MARYGQLSGKVSEQGLIEIL(E)KVSQQTEKKTTVKFNRRKVM
eua Rabbit       MARYGQLSGKVSEQGLIEIL(E)KVSQQTEKKTTVKFNRRKVM
eua Pika         MARYGQLSGKVSEQGLIEIL(E)KVSQQTEKKTTVKXXXXXXX
lau Dog          MARYGQLSGKVSEQGLIEIL(E)KVSQQTEKKTTVKFNRRKVM
lau Cat          MARYGQLSGKVSEQGLIEIL(E)KVSQQTEKKTTVKFNRRKVM
lau Horse        MARYGQLSGKVSEQGLIEIL(E)KVSQQTEKKTTVKFNRRKVM
lau Cow          MARYGQLSGKVSEQGLIEIL(E)KVSQQTEKKTTVKFNRRKVM
lau Dolphin      MARYGQLSGKVSEQGLIEIL(E)KVSQQTEKKTTVKFSRRKVM
lau Alpaca       MARYGQLSGKVSEQGLIEIL(E)KVSQQTEKKTTVKXXXXXXX
lau Megabat      XXXXXXXXXXVTEQGLIEIL(E)KVSQQTEKKTTVKFNRRKVM
lau Microbat     XXXXXXXXXXVTEQGLIEIL(E)KVSQQTEKKTTVKFNRRKVM
lau Hedgehog     MARYGQLSGKVSEQGLIEIL(E)KVSQQTEKKTTVKFNRRKVM
afr Elephant     MARYGQLSGKVSEQGLIEIL(E)KVSQQTEKKTTVKFSRRRVM
afr Rock_hyrax   XXXXXXXXXXVSEQGLIEIL(E)KVSQQTEKKTTVKFNRRKVM
afr Tenrec       MARYGQLSGKVSEQGLIEIL(E)KVSQQTEKKTTVKFNRRKVM
xen Armadillo    MARYGQLSGKVSEQGLIEIL(E)KVSQQTEKKTTVKFNRRKVM
xen Sloth        MARYGQLSGKVSEQGLIEIL(E)KVSQQTEKKTTVKFNRRKVM
mar Opossum      MARYGQLSGKVTEQGLIEIL(E)KVSQQTEKKITVKFNRRKVM
mar Wallaby      MARYGQLSGKVSEQGLIEIL(E)KVSQQTEKKITVKXXXXXXX
pro Platypus     MARYGKLSGKVSEQGLIEIL(E)KVSQQTEKKMTVKFNRRKVM

NO 176
GN PEX1
IP IPI00411291
PE SISTREKLVLTTL
MP 806
EX Wagner
CL catarrhines
DE peroxisomal biogenesis factor 1
SQ
pri Human        VLVDRAIHSRLSRQSISTRE(K)LVLTTLDFQKALRGFLPASL
pri Chimpanzee   VLVDRAIHSRLSRQSISTRE(K)LVLTTLDFQKALRGFLPASL
pri Gorilla      VLVDRAIHSRLSRQSISTRE(K)LVLTTLDFQKALHGFLPASL
pri Orangutan    VLVDRAIHSRLSHQSISTRE(K)LVLTTLDFQKALHGFIPASL
pri Gibbon       VLVDRAIHSRLSRQSISTRE(K)LVLTTLDFQKALHGFIPASL
pri Rhesus       VLVDRAIHSRLSRQSISTRE(K)LVLTTLDFQKALHGFIPASL
pri Baboon       VLVDRAIHSRLSRQSISTRE(K)LVLTTSDFQKALHGFIPASL
pri Marmoset     VLVERAIHSRLSQQTISTRE(E)LVLTTLDFQKALQGFIPVSL
pri Mouse_lemur  VLVDRAIHSCLSHQNICSRE(E)LVLTTLDFQKARQGFIPASL
eua Treeshrew    VLVDRAIHSCLSHQSISTKE(E)LILTTLDFEKALQGFIPGSL
eua Mouse        VLVDRAIHSSLSRQHSSSRE(D)LTLTTSDFQKALRGFLPASL
eua Rat          VLVDRAIHSSLSRQQNPTRE(G)LTLTTADFQKALRGFLPASL
eua Kangaroo_rat VLVDRAIHSHLSHQQISTKE(E)LLLSTLDFQKALQRFIPASL
eua Guinea_pig   VLVDRAVHSCLSHQHISAKE(E)LVFTTSDFQKALGGFIPASL
eua Squirrel     MLVDRAIHSCLCHQHISTRE(E)LVLTTSDFEKALKGFTPASL
eua Rabbit       VLVDRAMHARLSHQTVSTEK(E)LVLTTLDFQKALQGFVPASL
eua Pika         LLVDRAVHAHLSHQCVSTKE(E)LVLTTWDFQKALQGFVPAAL
lau Dog          VLVDRAIHSHLSHQRITTRE(E)LVLTTLDFQKALQGFIPASL
lau Horse        VLVDRAIHSRLSHQSISTRE(E)LVLTTLDFQKALQGFVPASL
lau Cow          MLVDRAIHSHLSHQNVYTRE(E)LVLTTLDFQKALRGFTPVSL
lau Dolphin      VLMDRAIHSCLSHQSISTRE(E)LVLTTLDLRKALQGFIPVSL
lau Alpaca       VLVDRAIHSCLSHQSISTRE(E)LVLTTLDFHKALQGFIPASL
lau Megabat      VLVDRAIHSRLSHQNISTRE(E)LVLTTLDFQKALQGFIPASL
lau Microbat     VLVDRAIHSHLSHQSISTRE(E)LLLTTLDFQKALQGFVPASL
lau Hedgehog     MLMDRAIHFCLSDHSISTGE(E)LVLTTLDFQNALQGFVPVSL
lau Shrew        VLVDRAIHSCLSHHSISTKE(E)LVLTTLDFQKALQGFTPASL
afr Elephant     VLVDRAIHSRLSLQSISTKE(E)LVLTTLDFQKALHGFTPASL
afr Rock_hyrax   VLVDRAIHCRLSLQSVATKE(E)LVLRTLDFQKALCGFTPASL
afr Tenrec       VLMDRAIHSRLSLQSLSCKE(E)LVLTTLDFQKALHGFTPASL
mar Opossum      MLVDRAVHACISSRNVCTEE(G)LHLKTLDFQKALKGFTPTSL
mar Wallaby      MLVDRAVHACISSRNVCTKE(G)LLLKTLDFQKGLKGFTPTSL

NO 177
GN PFKP
IP IPI00009790.1
PE RNGQIDKEAVQKY
MP 156
EX Kim
CL simians
DE phosphofructokinase, platelet
SQ
pri Human        LFRKEWSGLLEELARNGQID(K)EAVQKYAYLNVVGMVGSIDN
pri Chimpanzee   IFRKEWSGLLEELARNGQID(K)EAVQKYAYLNVVGMVGSIDN
pri Gorilla      LFRKEWSGLLEELARNGQID(K)EAVQKYAYLNVVGMVGSIDN
pri Orangutan    LFRKEWSGLLEELARNGQID(K)EAVQKYAYLNVVGMVGSIDN
pri Rhesus       LFRKEWSGLLEELARNGQID(K)EAVQKYAYLNVVGMVGSIDN
pri Baboon       LFRKEWSGLLEELAKNGQID(K)EAVQKYAYLNVVGMVGSIDN
pri Marmoset     LFRKEWSGLLEELAKNGQIE(K)EAVQRYAHLNVVGMVGSIDN
pri Bushbaby     IFRKEWMELLEELVRNGQIE(E)KAMQKYAHLNVVGMVGSIDN
eua Mouse        LFRKEWSGLLEELARNGDID(N)DTVQKYSYLNVVGMVGSIDN
eua Rat          LFRKEWSGLLEELAKNGEID(S)DTVKKHAYLNVVGMVGSIDN
eua Kangaroo_rat LFRKEWSGLLEELAENGEID(N)EAVQKYAHLNVVGMVGSIDN
eua Guinea_pig   IFREEWSGLLEELAQNGKIS(E)EAMKKHGYLNVVGMVGSIDN
eua Squirrel     IFRKEWSELLVELAKNGKIS(E)EAVTKYSYLNVVGMVGSIDN
eua Rabbit       IFRMEWSGLLEELAQDGKID(N)EAVQKYAYLNVVGMVGSIDN
lau Dog          IFRKEWSGLLEELAQHGEID(K)EQVQKHAYLNVVGMVGSIDN
lau Cat          IFRKEWSGLLKELAENGDID(R)EEVQKHAYLNVVGMVGSIDN
lau Horse        IFRKEWSGLLEELAQNGQIN(K)EEVEKYSYLNVVGMVGSIDN
lau Cow          IFQTEWSGLLEELARDGKIS(T)EQVQKHGHLNVVGMVGSIDN
lau Dolphin      IFRKEWSGLLEELAREGKIN(T)EEVQKHGYLNVVGMVGSIDN
lau Megabat      IFREEWSGLLEELARNGKID(K)GEVEKYAHLNVVGMVGSIDN
afr Elephant     IFRKEWSGLLEELAQSGKID(S)DAMKKHAYLNIVGMVGSIDN
xen Sloth        IFRKEWSGLLEELAENGKID(A)DTMKEYSYLNVVGMVGSIDN
mar Opossum      LFREEWSGLLDELAKNGKIS(K)DDVKNYSHLNIVGLVGSIDN
pro Platypus     LFREEWSGLLEELAREGKIE(K)EAVKKYAYLNIVGMVGSIDN

NO 178
GN PGAM5
IP IPI00063242.5
PE EEELASKLDHYKA
MP 88
EX Kim, Wagner
CL simians
DE phosphoglycerate mutase family member 5
SQ
pri Human        LSLINVRKRNVESGEEELAS(K)LDHYKAKATRHIFLIRHSQY
pri Chimpanzee   LSLINVRKRNVESGEEELAS(K)LDHYKAKATRHIFLIRHSQY
pri Gorilla      LSLINVRKRNVESGEEELAS(K)LDHYKAKATRHIFLIRHSQY
pri Orangutan    LSLINVRKKNVESGEEELAS(K)LDHYKAKATRHIFLIRHSQY
pri Gibbon       LSLINVRKRNVESGEEELAS(K)LDHYKAKATRHIFLIRHSQY
pri Rhesus       LSLINLRKRNVESGEEELAS(K)LDHYKAKATRHIFLIRHSQY
pri Baboon       LSLINLRKRNVESGEEELAS(K)LDHYKAKATRHIFLIRHSQY
pri Marmoset     LSLINLRKRNVESGEEELAS(K)LDHYKAKATRHIFLIRHSQY
pri Mouse_lemur  LSMINLRKRNVESGEEELAS(R)LDHYKAKATRHIFLIRHSQY
eua Mouse        LSLINLKKRNVESGEDELTS(R)LDHYKAKATRHIFLIRHSQY
eua Rat          LSLINLKKRNVESGEDELAS(R)LDHYKAKATRHIFLIRHSQY
eua Guinea_pig   LSLINLRKRNVESGEEELAS(R)LDHYKAKATRHIFLIRHSQY
eua Squirrel     LSLVNLRKRNVESGEEEQAS(R)LDHYRAKATRHIFLIRHSQY
eua Rabbit       LSLINLRKRNVESGEEELAS(R)LDHYKAKATRHIFLIRHSQY
eua Pika         LSLINLRKRNVESGEEELAS(R)LDHYKAKATRHIFLIRHSQY
lau Dog          LSLVNLRKRNLDSGEEELAS(R)LDHCKAKATRHIFLIRHSQY
lau Cat          LSLVNLRKRNLESGEEELAS(R)LDHYKAKATRHIFLIRHSQY
lau Horse        LSLVNLRKRNLESGEEELAS(R)LDHYKAKATRHIFLIRHSQY
lau Cow          LSLVNLRKRNLETGEEELTS(R)LDHCKAKATRHIFLIRHSQY
lau Dolphin      LSLVNLRKRNLEPGEEELAS(R)LDHYKAKATRHIFLIRHSQY
lau Megabat      LSLVNVRKRSLESGDDDLPT(R)MDHYKAKATRHIFLIRHSQY
lau Microbat     LSLVNLRKRHSGSGDEDLAS(R)IDNYKAKATRHIFLIRHSQY
lau Hedgehog     LSLVNLRKRNVESGEEEMPS(R)LDHFKAKATRHIFLIRHSQY
afr Elephant     LSLVNLRKRNLESGEEELTS(K)LDQCKAKATRHIFLIRHSQY
afr Rock_hyrax   SSLVNLRKRNLESGEEELAS(K)LDQCKAKATRHIFLIRHSQY
mar Opossum      RSLINLRKRSVETGEEEIAT(K)LDNFKARATRHIFLIRHSQY
mar Wallaby      RSLINLRKRSTETGEEEITS(K)LDNFRARATRHIFLIRHSQY
pro Platypus     RSLINLRKRNTETGEEELAS(K)LEHHKAKATRHIFLIRHSQY

NO 179
GN PIK3C2A
IP IPI00002580.2
PE VSSLLAKDPWDAV
MP 301
EX Kim
CL simians
DE phosphoinositide-3-kinase, class 2, alpha polypeptide
SQ
pri Human        VDNVEVLDHEEEKNVSSLLA(K)DPWDAVLLEERSTANCHLER
pri Chimpanzee   VDNVEVLDHEEEKNVSSLLA(K)DPWDAVLLEERSTANCHLER
pri Gorilla      VDNVEVLDHEEEKNVSSLLA(K)DPWDAVLLEERSTANCHLER
pri Orangutan    VDNVEVLDHEEEKNVSSLLA(K)DPWDAVLLEERSTANCHLER
pri Gibbon       VDNVEVLDHEEEKNVSSLLA(K)DPWDAVLLEERSPANCHLER
pri Rhesus       VDNVEVLDHEEEKNISSLLA(K)DPWDAVLLEERLPANCHLER
pri Baboon       VDNVEVLDHEEEKNVSSLLA(K)DPWDAVLLEERLPANCHLER
pri Marmoset     VDNVEVLDHEEEKNVSSLLE(K)DPWDAVLLEERSPANCHLER
pri Tarsier      VDNVEALVHEEEKNIPSLLA(E)DPWDAVLLEERSPASCHLER
eua Treeshrew    VDNVEILDH-EEKNVPSLLA(E)DPWDAVLLEERSLASCHLER
eua Mouse        VDYVEVLEHEEEKKDPVLLA(E)DPWDAVLLEERSP-SCHLER
eua Rat          IDCVEVLEHEEEKKNPVLLA(E)DPWDAVLLEERSPTSRHLER
eua Kangaroo_rat VDNVEVVDHDEEKNFPRLLA(E)DPWDAVLLEERSPSSCHLER
eua Guinea_pig   VDNVEVLDHEEDKHVPSLLA(E)DPWDAVLLGVRSPASCHLER
eua Rabbit       VDNVEVLGHEEEKNIPNLLA(E)DPWDAVLLEERSPANCHFER
eua Pika         VDNVEVLDHEEEKSIPNLLA(E)DPWEAVLLEERSPASCHFER
lau Dog          VDNVEVLNHEEEKNIPGLLA(E)DPWDAVLLEERSPASCHLER
lau Cat          VDNVEVVDHEEEKNIPGLLA(E)DPWDAVLLEERSPASCHLER
lau Horse        VDSVEVLDHEEEKNAPGLLA(E)DPWDAVLLEERSLASCHLER
lau Cow          VDNVEVEDHEEEKNVPDLLA(D)DPWDAVLLEERSPASCHLDR
lau Dolphin      VDSVEVLDHEEEKNIPGLLA(D)DPWDAVLLEERSPASCHLER
lau Alpaca       VDNVEVLDHEEEKNVPGLLA(D)DPWDAVLLEERSPASCHLER
lau Megabat      VDNVEVLDHEEEKNVPGLLA(E)DPWDAVLLEERSPASCHLER
lau Hedgehog     VDNVEGLGWEEEEAVPSLLA(E)DPWDAVLLEERSLASCHHER
lau Shrew        VDNVEVLEHEEEKIVSGLLA(E)DPWDAVLLEERSLANSNHER
afr Elephant     VDNVEVLHHEEEKNVPGLLA(E)DPWDAVLLEERSLASCHLER
afr Tenrec       VDNVEVLDHVEEKDVPGLLA(E)DPWDAVLLEGRSLATCH-ER
xen Sloth        VDNVDVLDHEEKKNVPGLLA(E)DPWDAVLLEERSLASCHLER
mar Opossum      VDNVEVLDHGEEKNTPILTA(E)DPWDAVLLEKRSSTGCNPEK
mar Wallaby      VDNMEVLDHVEEKNTPVLTA(E)DPWEAVLLEKRPPVGCHPEK
pro Platypus     VDSVEALDDEEEKITPVVTA(K)DPWDAVLLEERSPASCHPER

NO 180
GN PLD4
IP IPI00060310
PE WVLGVPKAVLPKT
MP 278
EX Wagner
CL catarrhines
DE phospholipase D family, member 4
SQ
pri Human        SHLAQDLEKTFQTYWVLGVP(K)AVLPKTWPQNFSSHFNRFQP
pri Chimpanzee   SHLAQDLEKTFQTYWVLGVP(K)AVLPKTWPQNFSSHINRFQP
pri Gorilla      SHLAQDLEKTFQTYWVLGVP(K)AVLPKTWPQNFSSHINRFQP
pri Orangutan    SHLAQDLEKTFQTYWVLGVP(K)AVLPKTWPQNFSSHINRFQP
pri Gibbon       SHLAQDLEKTFQTYWVLGVP(K)AVLPKTWPQNFSSHINRFQP
pri Rhesus       SHLAQDLEKTFQTYWVLGVP(K)AVLPKTWPQNFSSHINRFQP
pri Baboon       SHLAQDLEKTFQTYWVLGVP(K)AVLPKTWPQNFSSHINRFQP
pri Marmoset     SHLAQDLEKTFQTYWVLGAP(E)AVLPKTWPQNFSSDINRFQP
pri Tarsier      SHLAQDLEKTFQTYWVLGAP(Q)AVLPKTWPQNVSSHINRFRP
pri Bushbaby     SHLAQDLEKTFQTYWVLGVP(H)AVLPKTWPRNFSSHINRFQP
pri Mouse_lemur  SHLARDLEKTFQTYWVLGAP(Q)AVLPKTWPQNFSSHINLAQP
eua Mouse        SNLAQDLEKTFQTYWVLGTP(Q)AVLPKTWPRNFSSHINRFHP
eua Rat          SHLAEDLEKTFQTYWVLGTP(Q)AVLPKPWPRNFSTHINRFHP
eua Kangaroo_rat SHLAQDLEKIFQTYWALGAP(H)AVLPQTWPRNLSTHINHAQP
eua Guinea_pig   SSLAQDLEKTFQTYWVLGAP(H)AVLPKTWPQNFSSHINRFLP
lau Dog          SHLALDLEKTFQTYWVLGAP(K)AVLPKAWPHNFWSHINRFQP
lau Cat          SRLARDLEKTFQTYWVLGAP(K)AVLPRAWPRNLSSHINRFQP
lau Horse        SRLARDLEKTFQTYWVLGAP(K)AVLPKAWPRNFSSHINRFQP
lau Cow          SRLAQDLEKTFQTYWVLGTP(R)AVLPKRWPQNFSSHINRFQP
lau Dolphin      SCLAQDLEKTFRTYWVLGVP(K)AVLPRPWPRNFSSHINRFQP
lau Alpaca       SRLAQDLEKTFQTYWVLGAP(E)AVLPKPWPRNFSSHINRFQP
lau Megabat      SRLARDLDKTFQTYWVLGAP(Q)AVLPRHWPWNLSAHINQLQP
lau Hedgehog     SQLARDLEKTFQTYWVLGAP(H)ATLPKHWPGNYSTHINRFHP
afr Elephant     SRLAQDLEKTFRIYWALGTP(Q)ATLPKTWPRNFSSHINRFQP
afr Rock_hyrax   SRLARDLERTFQTYWVLGAP(R)ATLPKSWPRNFSSHINHFQP
afr Tenrec       SRLAQDLDKTFQTYWALGAP(G)ATLPLVWPQNFSSHINRIQP
mar Opossum      SSLAHDLEKTFQTYWILGAP(G)ATIPKHWPQNYSTNINWHQP
pro Platypus     SRLARDLWKIFQTYWDLGTP(G)AAIPPSWPQNYSTSINSHRP

NO 181
GN PLRG1
IP IPI00002624.1
PE ALPLQTKADANRT
MP 135
EX Kim
CL simians
DE pleiotropic regulator 1
SQ
pri Human        RMPSESAAQSLAVAL-PLQT(K)ADANRTAPS-GSEYRHPGAS
pri Chimpanzee   RMPSESAAQSLAVAL-PSQT(K)ADANRTAPS-GSEYRHPGAS
pri Gorilla      RMPSESAAQSLAVAL-PSQT(K)ADANRTAPS-GSEYRHPGAS
pri Orangutan    RMPSESAAQSLAVAL-PSQT(K)ADANRTAPS-GSEYRHPGAS
pri Gibbon       RMPSESAAQSLAVAL-PSQT(K)ADANRTAPS-GSEYRHPGAS
pri Rhesus       RMPSESAAQSLAVAL-PSQT(K)TDVNRTAPS-GSEYRHPGAS
pri Baboon       RMPSESAAQSLAVAL-PSQT(K)TDVNRTAPS-GSEYRHPGAS
pri Marmoset     RMPSESAAQSLAVAL-PSQA(K)ADANRTAPS-GSEYRHPGAA
pri Tarsier      RMPSESAAQSLAVAL-PSQA(R)VDANRTAPG-GSEYRHPGAS
pri Bushbaby     RMPSESAAQSLAVAL-PSQA(R)TDANRTAAG-GSEYRHPGAP
pri Mouse_lemur  RMPSESAAQSLAVAL-PSQA(R)ADANRTAPG-GNEYRHPGAP
eua Treeshrew    RMPSESAAQSLAVTL-PSQA(R)VDANRTAPG-GSEYRHPGAA
eua Mouse        RMPSESAAQSLAVAL-PSQT(R)VDANRTGPA-GSEYRHPGAS
eua Rat          RMPSESAAQSLAVAL-PSQT(R)VDANRTAPA-GSEYRHPGAS
eua Guinea_pig   RMPSESAAQSLAVAS-PSQA(R)TDTNRTTSG-GSEYRHPGAS
eua Rabbit       RMPSESAAQALAVTL-PSQT(R)VDANRTAPG-GGEYRLPGAS
lau Dog          RMPSESAAQSLAVAL-PSQT(R)VDANRSAPA-GGEYRHPGTS
lau Horse        RMPSESAAQSLAVALPPSQA(R)VDANRSAPA-GGEFRHSGAS
lau Cow          RMPSESAAQSLAVALPASQA(R)ADANRPVPA-GGEYRHPGAP
lau Dolphin      RMPSESAAQSLAVAL-PTQA(R)VDANRSAPA-GGEYRHPGAS
lau Alpaca       RMPSESAAQSLAVAL-PTQT(R)GDANRSAPA-GGEYRHPGAS
lau Megabat      RMPSESAAQSLAVAL-PSQA(R)VDANRSAPA-GGEYRHPGAS
afr Elephant     RMPSESAAQSLAVAL-PSQA(R)VDANRTAPG-GSEYRHPGAS
afr Rock_hyrax   RMPSESAAQSLTVAL-PSQT(R)VDANRTAPG-GSEYRHPGSS
afr Tenrec       RMPSESAAQSVAVAL-PSQS(R)VDANRTTPG-GNEYXHPGAS
xen Armadillo    RMPSESAAQSLAVAL-PSQA(R)VDANRTAPG-GNEYRHPGAS
xen Sloth        RMPSESAAQSLAVAL-PSQA(R)VDANRTVPG-GNEYRHPGAS
mar Opossum      RMPSESAAQSLAVALPPSQS(R)IDANRTSATVGDIYRHAGLP
pro Platypus     RMPSESAAQSLAVALPPSQS(R)IDANRTAASVGDIYRHAGIA

NO 182
GN PML
IP IPI00022348.2
PE SCITQGKDAAVSK
MP 394
EX Kim, Wagner
CL great apes
DE promyelocytic leukemia
SQ
pri Human        DGFDEFKVRLQDLSSCITQG(K)DAAVSKKASPEAASTPRDPI
pri Chimpanzee   DGFDEFKVRLQDLSSCITQG(K)DAAVSKKASPEAASTPRDPI
pri Gorilla      DGFDEFKVRLQDLSSCITQG(K)DAAVSKKASPEAASTPRDPI
pri Orangutan    DGFDEFKVRLQDLSSCITQG(K)DAAVSKKASPEAASTPRDPI
pri Gibbon       DGFDEFKVRLHDLSSCITQG(T)DAAASKKASPEAASTPRDPT
pri Rhesus       DGFDEFKVRLQDLSSCITQG(T)DAAXXXXXXXXXXXXXXXXX
pri Baboon       DGFDEFKVRLQDLSSCITQG(T)DAAVAKKASPEAASTPRDPT
pri Marmoset     DGFDEFKVRLQDLISCITQG(T)DAAVSKQARPEAASTPRDPI
pri Mouse_lemur  DGFDEFRARLQDLISSITQR(T)DAVLSRRASPEATSTLRDSS
eua Mouse        RGFEEFKLCLQDFISCITQR(I)NAAVAS---PEAASNQPEAA
eua Rat          SGFEEFKLCLQDLVSFITQR(T)DVAVAGRASPEAASTHPEAA
eua Guinea_pig   DSFDEIKVRLQDLISCIPQG(T)DTSLSRRVISEASGTPSDSS
eua Squirrel     DGFEEFKIRLQDFISCITQG(T)DAAVCRRASPETAGTPREPF
eua Rabbit       EGFDEFKVRLQDLVARVTQA(T)DAILSRRTSPQSASTPGDLL
lau Dog          DGFEEFKVRLQELVSCITRG(T)DLAVPRRASPDAAGTPRDSL
lau Cat          DGFDEFKVRLQGLVSCITQA(T)DAALHRRASPEASSTPRDSS
lau Horse        DRFDEFKVRLQDLVSCITQG(T)DAAPPRRASPEAPSTPTDAS
lau Cow          DSFDEFRVRLQDLVSCITRG(T)DAALSRRASPEAASTPRDTC
lau Microbat     DGFEEFKERLQDLVSCITKG(T)DAALPRRANPEETSTPREPS
afr Elephant     DGFNEFKVRLQDLISCTTEG(T)DAALPKRASPEAARTPRNSF
afr Rock_hyrax   DSFDEFKVRLQDLVSCITQR(T)DAAVPRRPRAAAASSPRNSS

NO 183
GN PML
IP IPI00022348.2
PE KDAAVSKKASPEA
MP 400
EX Kim
CL simians
DE promyelocytic leukemia
SQ
pri Human        KVRLQDLSSCITQGKDAAVS(K)KASPEAASTPRDPIDVDLPE
pri Chimpanzee   KVRLQDLSSCITQGKDAAVS(K)KASPEAASTPRDPIDVDLPE
pri Gorilla      KVRLQDLSSCITQGKDAAVS(K)KASPEAASTPRDPIDVDLPE
pri Orangutan    KVRLQDLSSCITQGKDAAVS(K)KASPEAASTPRDPIDVDLPE
pri Gibbon       KVRLHDLSSCITQGTDAAAS(K)KASPEAASTPRDPTDVDLPE
pri Baboon       KVRLQDLSSCITQGTDAAVA(K)KASPEAASTPRDPTDVDLPE
pri Marmoset     KVRLQDLISCITQGTDAAVS(K)QARPEAASTPRDPIDVDLPE
pri Mouse_lemur  RARLQDLISSITQRTDAVLS(R)RASPEATSTLRDSSDIDSPE
eua Guinea_pig   KVRLQDLISCIPQGTDTSLS(R)RVISEASGTPSDXXXXXXXX
eua Squirrel     KIRLQDFISCITQGTDAAVC(R)RASPETAGTPREPFDVDLXX
eua Rabbit       KVRLQDLVARVTQATDAILS(R)RTSPQSASTPGDXXXXXXXX
lau Dog          KVRLQELVSCITRGTDLAVP(R)RASPDAAGTPRDSLDVDQPE
lau Cat          KVRLQGLVSCITQATDAALH(R)RASPEASSTPRDSSDIDLPE
lau Horse        KVRLQDLVSCITQGTDAAPP(R)RASPEAPSTPTDASDVDLPE
lau Cow          RVRLQDLVSCITRGTDAALS(R)RASPEAASTPRDTCGTDPPE
lau Microbat     KERLQDLVSCITKGTDAALP(R)RANPEETSTPREPSDVDLPA
afr Elephant     KVRLQDLISCTTEGTDAALP(K)RASPEAARTPRNSFDVDLPE
afr Rock_hyrax   KVRLQDLVSCITQRTDAAVP(R)RPRAAAASSPRNSSDTDLPV

NO 184
GN PML
IP IPI00022348.2
PE DAAVSKKASPEAA
MP 401
EX Kim
CL catarrhines
DE promyelocytic leukemia
SQ
pri Human        VRLQDLSSCITQGKDAAVSK(K)ASPEAASTPRDPIDVDLPEE
pri Chimpanzee   VRLQDLSSCITQGKDAAVSK(K)ASPEAASTPRDPIDVDLPEE
pri Gorilla      VRLQDLSSCITQGKDAAVSK(K)ASPEAASTPRDPIDVDLPEE
pri Orangutan    VRLQDLSSCITQGKDAAVSK(K)ASPEAASTPRDPIDVDLPEE
pri Gibbon       VRLHDLSSCITQGTDAAASK(K)ASPEAASTPRDPTDVDLPEE
pri Baboon       VRLQDLSSCITQGTDAAVAK(K)ASPEAASTPRDPTDVDLPEE
pri Marmoset     VRLQDLISCITQGTDAAVSK(Q)ARPEAASTPRDPIDVDLPEE
pri Mouse_lemur  ARLQDLISSITQRTDAVLSR(R)ASPEATSTLRDSSDIDSPEE
eua Guinea_pig   VRLQDLISCIPQGTDTSLSR(R)VISEASGTPSDXXXXXXXXX
eua Squirrel     IRLQDFISCITQGTDAAVCR(R)ASPETAGTPREPFDVDLXXX
eua Rabbit       VRLQDLVARVTQATDAILSR(R)TSPQSASTPGDXXXXXXXXX
lau Dog          VRLQELVSCITRGTDLAVPR(R)ASPDAAGTPRDSLDVDQPEE
lau Cat          VRLQGLVSCITQATDAALHR(R)ASPEASSTPRDSSDIDLPEE
lau Horse        VRLQDLVSCITQGTDAAPPR(R)ASPEAPSTPTDASDVDLPEE
lau Cow          VRLQDLVSCITRGTDAALSR(R)ASPEAASTPRDTCGTDPPEE
lau Microbat     ERLQDLVSCITKGTDAALPR(R)ANPEETSTPREPSDVDLPAG
afr Elephant     VRLQDLISCTTEGTDAALPK(R)ASPEAARTPRNSFDVDLPEE
afr Rock_hyrax   VRLQDLVSCITQRTDAAVPR(R)PRAAAASSPRNSSDTDLPVE

NO 185
GN PMPCB
IP IPI00927892
PE AAVGPIKQLPDFK
MP 472
EX Wagner
CL African great apes
DE peptidase (mitochondrial processing) beta
SQ
pri Human        EVCTKYIYNRSPAIAAVGPI(K)QLPDFKQIRSNMCWLRD*
pri Chimpanzee   EVCTKYIYNRSPAIAAVGPI(K)QLPDFKQIHSNMCWLRD*
pri Gorilla      EVCTKYIYNRSPAIAAVGPI(K)QLPDFKQIRSNMCWLRD*
pri Orangutan    EVCTKYIYNRSPAIAAVGPI(E)QLPDFKQICSNMCWLRD*
pri Gibbon       EVCTKYIYNRSPAIAAVGPI(E)QLPDFKQICSNMCWLHD*
pri Rhesus       EVCTKYIYNRSPAIAAVGPI(E)QLPDFKQICSNMCWLRG*
pri Baboon       EVCTKYIYNRSPAIAAVGPI(E)QLPDFKQICSNMCWLRG*
pri Marmoset     EVCTKYIYNKSPAIAAVGPI(E)QLPDFNQICSNMCWLRD*
pri Tarsier      EVCTKYIYDKSPAIAAVGPI(E)QLPDFNQIHSNMRWLRD*
eua Treeshrew    EVCTKYIYDKSPAIAAVGPI(E)LLPDFSQIRSNMSWLHD*
eua Mouse        RVCTKYIHDKSPAIAALGPI(E)RLPDFNQICSNMRWIRD*
eua Rat          EVCTKYIYGKSPAIAALGPI(E)RLPDFNQICSNMRWTRD*
eua Kangaroo_rat EICTKYIYGKSPAIAAVGPI(E)QLPDFNQICSNMCWPQN*
eua Guinea_pig   EVCTKYIYDKSPAVAAVGPI(E)QLPDFNQIRSNMCWIRG*
eua Squirrel     EVCTKYIYDKSPAIAAVGPI(E)QLPDFNQICSNMRWLRD*
eua Rabbit       EVCTRYIYDKSPAIAAVGPI(E)QLPDFNQICHNMRWLHD*
eua Pika         AVCTRYIYDKSPALAAVGPI(E)QLPDFNQICSDMCWPHH*
lau Dog          EVCTKYIYDKSPALAAVGPI(E)QLPDFNQIRRNMCWLRD*
lau Cat          EVCTKYIYDKSPALAAVGPI(E)QLPDFNQIRSNMCWLHD*
lau Horse        EVCTKYIYEKSPALAAVGPI(E)QLPEFNQICSNMRWLRD*
lau Cow          EVCTKYIYDKSPAVAAVGPI(E)QLPDFNQICSNMRWLHD*
lau Dolphin      EVCTKYIYDKSPAVAAVGPI(E)QLPDFKQICSNMCWLHD*
lau Alpaca       EVCTKYIYDKSPAVAAVGPI(E)QLPDFNQIRSNMCWLRD*
lau Megabat      EVCTKYIYDKSPAIAAVGPI(E)QLPDFNRIRSNMCWLRD*
lau Microbat     EVCSKYIYDKSPAIAAVGPI(E)QLPDFNRIRSNMCWLRN*
lau Hedgehog     EVCTKYIYDKSPAVAAVGPI(E)QLPEFNQIRSNMCWLRD*
lau Shrew        EVCMKYIYDKSPAVAAVGPI(E)HLPDFNQIRSNMCWLRD*
afr Elephant     EVCTKYIYDKSPAIAAVGPI(E)QLPHFNKIRSNMCWLHD*
afr Tenrec       EVCTKYIYDKSPAVAAVGPI(E)QLPDFDRIRSNMCWARG*
mar Opossum      DVCTKYIYDKHPAVAAVGPI(E)QLPDYNRICSGMHWLRE*
pro Platypus     DVCTRYIYDKSPAIAAVGPI(E)QLPDYDRIRSGLVWLRD*

NO 186
GN PNMA1
IP IPI00005685.2
PE SRDAQIKFLNTYQ
MP 249
EX Kim, Wagner
CL simians
DE paraneoplastic antigen MA1
SQ
pri Human        CLKALEQVFGSVESSRDAQI(K)FLNTYQNPGEKLSAYVIRLE
pri Chimpanzee   CLKALEQVFGSVESSRDAQI(K)FLNTYQNPGEKLSAYVIRLE
pri Gorilla      CLKALEQVFGSVESSRDAQI(K)FLNTYQNPGEKLXAYVIRLE
pri Orangutan    CLKALEQVFGSVESSRDAQI(K)FLNTYQNPGEKLSAYVIRLE
pri Rhesus       CLKALEQVFGSVESSRDAQI(K)FLNTYQNPGEKLSAYVIRLE
pri Baboon       CLKALEQVFGSVESSRDAQI(K)FLNTYQNPGEKLSAYVIRLE
pri Marmoset     CLQALEQVFGSVESSRDAQV(K)FLNTYQNPGEKLSAYVIRLE
pri Tarsier      CLKALKQVFGSIESSRDALV(R)FLNTYQNPGEKLSAYVIRLE
pri Bushbaby     CLKALEQVFGSVESSRDAQV(K)FLNTYQNPGEKLSAYVIRLE
pri Mouse_lemur  CLKALEQVFGSVESSRDAQV(R)FLNTYQNPGERLSAYVIRLE
eua Treeshrew    CLKALEQVFGSVESSRDAQV(R)FLNTYQNPGEKLSAYVIRLE
eua Mouse        CLKALEQVFGSVESSRDAQV(R)FLNTYQNPGEKLSSYVIRLE
eua Rat          CLKALEQVFGSVESSRDAQV(R)FLNTYQNPGEKLSSYVIRLE
eua Guinea_pig   CLKALEQVFGSIESSRDAQV(R)FLNTYQSPGEKLSAYVIRLE
eua Squirrel     CLKALEQVFGSVESSRDAQV(R)FLNTYQNPGEKLSAYVIRLE
eua Rabbit       CLKALEQVFGSVESSRDAQV(R)FLNTYQNPGEKLSAYVIRLE
lau Dog          CLKALEQVFGSVESSRDAQV(R)FLNTYQNPGEKLSAYVIRLE
lau Cat          CLKALEQVFGSVESSRDAQV(R)FLNTYQNPGEKLSAYVIRLE
lau Horse        CLKALEQVFGSVESSRDAQV(R)FLNTYQNPGEKLSAYVIRLE
lau Cow          CLKALEQVFGSVESSRDVQV(R)FLNTYQNPGEKLSAYVIRLE
lau Dolphin      CLKALEQAFGSVESCRDAQV(R)FLNTYQNPGEKLSAYVIRLE
lau Megabat      CLKALEQVFGSVESSRDAQV(R)FLNTYQNPGEKLSAYVIRLE
lau Microbat     CLKALEQVFGSVESSRDAQV(R)FLNTYQNPGEKLSAYVIRLE
lau Shrew        CLKALEQVFGSVESSRDAQV(R)FLNTYQNPGEKLSAYVIRLE
afr Elephant     CLKALEQVFGSVESSRDAQV(R)FLNTYQNPGEKLSAYVIRLE
afr Rock_hyrax   CLKALEQVFGSVESSRDAQV(R)FLNTYQNPGEKLSAYVIRLE
afr Tenrec       CLKALEQVFGSIESSRDAQV(R)FLNTYQNPGEKLSAYVIRLE
xen Armadillo    CLKALEQVFGSVESSRDAQV(R)FLNTYQNPGEKLSAYVIRLE

NO 187
GN POLI
IP IPI00296840
PE VLSFFSKKQMQDI
MP 549
EX Wagner
CL catarrhines
DE polymerase (DNA directed) iota
SQ
pri Human        GKGSVSCPLHASRGVLSFFS(K)KQMQDIPINPRDHLSSSKQV
pri Chimpanzee   GKGSMSCPLHASRGVLSFFS(K)KQMQDIPINPRDHLSSSKQV
pri Gorilla      GKGSVSCPLHASRGVLSFFS(K)KQMQDIPINPRDHLSSSKQV
pri Orangutan    GKGSVSCPLHASRGVLSFFS(K)KQMQDIPINPGDHLSSSKQV
pri Gibbon       GKGSVSRPLHASRGVLSFFS(K)KQMQDIPINPRDHLSSSKQV
pri Rhesus       GKGSVSCPLHASRGVLSFFS(K)KQMQDIPINPRDHLSSRKQV
pri Baboon       GKGSVNCPLHASRGVLSFFS(K)KQMQDIPINPRDHLSSHKQV
pri Marmoset     GKESVSCPLHASKGVLSFFS(T)KQMQNTPINPKNHSSSSKQV
pri Tarsier      GKGSLSCPLHASRGVLSFFS(T)KQMQDHPIGPRDRLCNSKQV
pri Bushbaby     GKENLSCPLHASRGVLSFFS(P)KRTQDSPINPRNPLFSSKQA
pri Mouse_lemur  GKENLNCPLHASRGVLSFFS(T)KQVQDNPTNPRDHLFNSKQV
eua Treeshrew    GKGSFSCPSHSSRGILSFFS(P)KRMQNTPLNSRDNFSSSKQE
eua Mouse        GKGSLSCPLHASRGVLSFFS(T)KQMQASRLSPRDTALPSKRV
eua Rat          GKGSLSCPLHASRGVLSFFS(T)KQTQAGCLSPRDTLLTGKQA
eua Kangaroo_rat EKGSLSCPLHASRGVLSFFS(T)KQMKDIPLTSRDNLSSSKQV
eua Guinea_pig   VNEKLSCSRQASRGLLSFFS(T)KQMQDSPLNSRGNLSSCKQV
eua Squirrel     GKGSLSCPLHASRGVLSFFS(T)KQMQDRSLNSRDNLSSNNQV
eua Rabbit       GKESLSCPLHASRGVLSFFS(T)KQMRDTPLNTRDHLSSSKEV
eua Pika         GKESLSSPLRKPRGILSYFS(T)KQMQDGPLKSEDHLSRSRGV
lau Dog          GKGSLNCPLHASRGVLSFFS(T)KQMQDSPLNPKDHLSNSKQI
lau Cat          GKESLSCPLHASRGVLSFFS(T)KQMKDSPLNPKDHLSSSKQI
lau Horse        GKGSLSCPLRASRGILSFFS(A)KQMQDSPLNPRDHLSNSKQL
lau Cow          GKGSLSCPLRASRGVLSLFS(T)KQKQDSSLNPKDHVPKSKQV
lau Dolphin      GKGSLSCPLRASRGVLSFFS(T)KQMQDGSLNPRDHLSNTKQI
lau Alpaca       GKGSSSCPLHASRGILSFFS(T)KQMQDGSLNPRERLSNSKQI
lau Megabat      GKGSLSCPLHASRGVLSFFS(T)KQMQNNPFNSRDHLSNSKQI
lau Shrew        SKASPRCSLPASRGILSFFS(T)KKVQESPSNCRDHLSSTQQM
afr Elephant     EKGSLSCPLRASRGILSFFS(T)KQMQDTPSNPRGHLSSSKQV
afr Rock_hyrax   EKGSLRCPLRASRGVLSFFS(T)KQVKDTASNTRGHLSSSGQA
afr Tenrec       GSGSSSYPLYPSRGILSFFP(T)KQMQGIPSNPNNHLSRSKQG
xen Armadillo    GKGSLSCPLHASRGILSFFS(T)KQMQDSPSNSRDHLPNSKQV
xen Sloth        GKGSLSCPLHASRGVLSFFS(T)KQMQDSPSNPRDHLPNSKQV
mar Opossum      GKGSLSCPLHASKGVLSFFS(S)KQMHDIVSNPRDHLSSSKQL
pro Platypus     GKRTPSYSLRASKGVLSFFP(Q)KDRQDSDSHCSEHAASSKHS

NO 188
GN PRCP
IP IPI00001593.1
PE HLDLRTKNALDPM
MP 462
EX Kim
CL simians
DE prolylcarboxypeptidase (angiotensinase C)
SQ
pri Human        TDTLVAVTISEGAHHLDLRT(K)NALDPMSVLLARSLEVRHMK
pri Chimpanzee   TDTLVAVTISEGAHHLDLRT(K)NALDPTSVLLARSLEVRHMK
pri Gorilla      TDTLVAVTISEGAHHLDLRT(K)NALDPTSVLLARSLEVRHMK
pri Orangutan    TDTLVAVTISEGAHHLDLRT(K)NALDPTSVLLARSLEVRHMK
pri Gibbon       TDTLVAVTISEGAHHLDLRT(K)NALDPTSVLLARSLEVRHMK
pri Rhesus       TDTLVAVTISEGAHHLDLRA(K)NALDPTSVLLARALEVRHMK
pri Baboon       TDTLVAVTISEGAHHLDLRA(K)NALDPTSVLLARALEVRHMK
pri Marmoset     TDTLVAVTISEGAHHLDLRA(K)NALDPTSVLLARVLEVRHMK
pri Tarsier      TDTLVAVTIPEGAHHLDLRA(R)NALDPKSVLLARSLEVKHMK
eua Treeshrew    TDTLVAVTIAEGAHHLDLRA(N)NAFDPESVLVARSLEVKHMK
eua Mouse        TDTLVAINIHDGAHHLDLRA(H)NAFDPSSVLLSRLLEVKHMK
eua Rat          TDTLVAINIPEGAHHLDLRA(H)NAFDPSSVLLSRLLEVKHMK
eua Kangaroo_rat SDTLVAIFIKDAAHHLDLRA(N)HAQDPESVLQARLWEVSYMK
eua Guinea_pig   SDTLVAINIAEGAHHLDLRA(Y)SAYDPASVLLARSLEVEYMK
eua Squirrel     TDTLVAVTIKEGAHHLDLRA(N)NPLDPTSVLLARALEIEHMK
eua Rabbit       TDTLVAITISEGAHHLDLRA(N)NEYDPASVLLARTLEVRYMK
eua Pika         TDTLIAITILNGAHHLDLRA(N)NAFDPKSVLLARTSEVKYMK
lau Dog          TDTLVAITIPEGAHHLDLRA(R)NAFDPTAVLLARSLEVRHMK
lau Cat          TDTLVAITIPEGAHHLDLRA(R)NAFDPTTVLLARSLEVRHMK
lau Horse        TDTLVAITIPEGAHHLDLRA(N)NAFDPTSVLLARSLEVRYMK
lau Cow          TDTLLAIVIPNGAHHLDLRA(S)NALDPVSVQLTRSLEVKYMK
lau Megabat      TDTLVAITIPEGAHHLDLRA(N)NAFDPTTVVLARSMEVRYMK
lau Hedgehog     SDTLVAIVIPEGAHHLDLRS(N)NAFDPLTVLLARSLEVKYMQ
lau Shrew        TDTLVAIIIPEGAHHLDLRA(S)NPFDPESVLLARSLEVKYMK
afr Elephant     TDTLVAINIPDGAHHLDLRA(S)NALDPKTVLLARSLEVRYMK
afr Rock_hyrax   TDTLVAINIPDGAHHLDFRA(S)NPLDPQTVLLARSLEVRHME
xen Armadillo    SDTLIAIKIPEGAHHLDLRA(N)NPLDPQSVLLARSLEVKHMK
xen Sloth        TDTLIAINIPQGAHHLDLRA(N)NALDPQSVLLARSLEVKHMK
mar Opossum      TDNLVSIVIPDGAHHLDLRA(R)NIDDPQSVLFARAKEVEYMK
mar Wallaby      TDTLISIVIPDGAHHLDLRA(R)NADDPESVLFARTMEVYYMK
pro Platypus     TDTLVAVVIPEGAHHLDLRA(N)NPYDPKSVLQARAAEVHLIK

NO 189
GN PRKDC
IP IPI00296337.2
PE LLTFIDKAMHGEL
MP 3067
EX Kim
CL simians
DE protein kinase, DNA-activated, catalytic polypeptide
SQ
pri Human        SKLKLLLQGEADQSLLTFID(K)AMHGELQKAILELHYSQELS
pri Chimpanzee   SKLKLLLQGEADQSLLTFID(K)AMHGELQKAILELHYSQELS
pri Gorilla      SKLKLLLQGEADQSLLTFID(R)AMHGELQKAILELHYSQELS
pri Orangutan    SKLKLLLQGEADQSLLTFID(K)AMHGELQKAILELHYSQELS
pri Gibbon       SKLKLLLQGEADQSLLTFID(K)AMHGELQKAILELHYSQELS
pri Rhesus       SKLKLLLQGEADQSLLTFID(K)AMHGELQKAILELHYSQELS
pri Baboon       SKLKLLLQGEADQSLLTFID(K)AMHGELQKAILELHYSQELS
pri Marmoset     SKLKLLLQGEADQSLLTFID(K)AMHGELQKVILELHYSQEMS
pri Tarsier      SKLKLLLQGEADQSLLTFVD(E)AMHEELQKALLELHYNQELS
pri Bushbaby     SKLKLLLRGEGDQTLLTFLD(E)AMKVELRKALLELQYSQELS
pri Mouse_lemur  SKLKLLLRGEGDQSLLTFVD(E)AMRSELRKALVELQYSQELS
eua Mouse        SKLKLLLQGEGNQSLLTFVD(E)AMNKELQKTVLELQYSQELS
eua Rat          SKLKLLLQGEDNQSLLTFVD(E)AMHKELQQMVLELQYSQELS
eua Kangaroo_rat SKLKLLLRGEGDQSLLTFMD(Q)AMRKELQQLLLEVQYSQELS
eua Guinea_pig   SKLKLLLRGEGEQSLLTFVD(E)AMRGELQQALMELQYSQELS
eua Rabbit       SKLKLLLQGEADQSLLTFVD(E)AVHQELQKTVLELQYSQELS
eua Pika         SKLKLLLQGEAEQSLLTFVD(E)AMQQDVRKVLLELQYSQELS
lau Dog          SKLKLLLQGEADQSLLTFID(E)AVNKDLQKALIELHYSQELS
lau Cat          SKLKLLLQGEADQSLLTFID(E)AVNKEVQKALIELHYSQELS
lau Horse        SKLKLLLQGEGDQSLLTFID(E)AVSKELQKVLVELHYSQELS
lau Cow          SKLKLLLRGEGDQALLTFVD(E)ALTSELRKALLELRYSQELS
lau Dolphin      SRLKALLRGEGDQALLTFVD(E)AVTSELRRALLELRYSQELS
lau Microbat     SKLKLLLQGENDQSLLTFID(E)AVNKELQKSIIELNYSQELS
lau Hedgehog     SKLKLLLQGEADQSLLTFID(D)AVSKELQKLLIELHYSQELS
afr Elephant     SKLKLLLHGEDDQSLLTFVD(N)SVSKELQKVLLELHYSQELS
afr Rock_hyrax   SKLKLLLRGEHDQSLLTFID(N)SVSKELQKVLLELHYSQELS
afr Tenrec       SKLKLLLHGDNDQSLLTFVD(N)SVNTELQKVLLELHYSQELS
xen Armadillo    SKLKLLLQGGDDQSLLTFID(D)AVNKELQKTLIELHYSQELS
xen Sloth        SKLKLLLQGGDDQSLLTFID(A)AVNKELQKTLIELHYSQELS
mar Opossum      SKLKLLLQGASDQSLLKFID(E)AMKKEPQKTLIEVHYSQELS
pro Platypus     GKLKLLLQGGSDQSLLTFMD(E)AMKKEKQKTLIETHYSQELS

NO 190
GN PSAT1
IP IPI00001734
PE LAAFMKKFLEMHQ
MP 408
EX Wagner
CL apes
DE phosphoserine aminotransferase 1
SQ
pri Human        ASLYNAVTIEDVQKLAAFMK(K)FLEMHQL*
pri Chimpanzee   ASLYNAVTIEDVQKLAAFMK(K)FLEMHQL*
pri Gorilla      ASLYNAVTIEDVQKLAAFMK(K)FLEMHQL*
pri Orangutan    ASLYNAVTIEDVQKLAAFMK(K)FLEMHQL*
pri Gibbon       ASLYNAVTIEDVQKLAAFMK(K)FLEMHQL*
pri Rhesus       ASLYNAVTIEDVQKLAAFMK(N)FLEMHQL*
pri Baboon       ASLYNAVTIEDVQKLAAFMK(N)FLEMHQL*
pri Marmoset     ASLYNAVTIEDVQKLATFMK(N)FLEMHQL*
pri Tarsier      VSLYNAITIEDVQKLAAFMK(N)FLEMHQL*
pri Bushbaby     ASLYNAVTIEDVQKLAAFMK(N)FLEMHQL*
pri Mouse_lemur  ASLYNAVTIEDVQKLAAFMK(N)FLEMHQL*
eua Treeshrew    ASLYNAVTIEDVQKLAAFMK(N)FLEMHQL*
eua Mouse        ASLYNAVTTEDVEKLAAFMK(N)FLEMHQL*
eua Rat          ASLYNAVTTEDVEKLAAFMK(N)FLEMHQL*
eua Kangaroo_rat ASLYNAVTVEDVEKLAAFMK(N)FLEMHQL*
eua Guinea_pig   ASLYNAVTVEDVQKLAAFME(N)FLEMHQL*
eua Squirrel     VSLYNAVTTEDVETLATFMK(N)FLEMHQL*
eua Rabbit       VSLYNAVTIEDVQKLASFMK(N)FLEMHQL*
eua Pika         VSLYNAVTVEDVQKLAAFMR(N)FLEMHQL*
lau Dog          ASLYNAVTIEDVQKLAAFMK(N)FLEMHQL*
lau Cat          ASLYNAVTIEDVQKLAAFMK(N)FLEMHQL*
lau Horse        ASLYNAVTIEDVQKLAAFMK(N)FLEMHQL*
lau Cow          VSLYNAVTVEDVQKLAAFMK(N)FLEMHQL*
lau Dolphin      ASLYNAVTIEDVQKLAAFMK(N)FLEMHQL*
lau Megabat      ASLYNAVTIEDVQELAAFMK(K)FLEMHQL*
lau Microbat     ASLYNAVTIEDAQELAAFMK(K)FLEMHQL*
lau Hedgehog     ASLYNAVTIEDVQKLAAYMK(N)FLEMHQL*
lau Shrew        ASLYNAVTLEDVEKLAAFMK(N)FLEMHQL*
afr Elephant     ASLYNAVTIEDVQKLAAFMK(N)FLEMHQL*
afr Rock_hyrax   ASLYNAVTIEDVQKLAAFMK(N)FLEMHQL*
afr Tenrec       ASLYNAVTTEDVQKLAAFMT(N)FLEMHQL*
xen Sloth        VSLYNAVTIDDVQKLAAFMK(N)FLEMHQL*
mar Opossum      ASLYNAVTTEDVQKLAAFMK(T)FMETHQQ*
pro Platypus     VSLYNAITVEDVQLLATFME(S)FMETHRF*

NO 191
GN PTGR1
IP IPI00292657.3
PE MGQQVAKVVESKN
MP 70
EX Kim
CL apes
DE prostaglandin reductase 1
SQ
pri Human        MRVAAKRLKEGDTMMGQQVA(K)VVESKNVALPKGTIVLASPG
pri Chimpanzee   MRLAAKRLKEGDTMMGQQVA(K)VVESKNAALPKGTIVLASPG
pri Orangutan    MRFAARSLKEGDKMMGQQVA(R)VVESKNAALPKGTIVLASPG
pri Gibbon       MRLAAKRLKEGDTMMGQQVA(K)VVESKNAALPKGTIVLASSG
pri Rhesus       MRFLAKTLKEGDRMMGQQVA(R)VVESKNADLPKGTIVLTSPG
pri Baboon       MRFLAKTLKEGDRMMGQQVA(R)VVESKNADLPKGTIVLTSPG
pri Marmoset     MRVTAKRLKEGDTMMGQQVA(R)IVESKNADLPTGTVVLASSG
pri Bushbaby     XXLASKKLNEGDTMMGQQVA(R)VVESKNSTLPKGTIVVAFLG
pri Mouse_lemur  MRIASKRLNEGDTMMGQQVA(R)VVESKNSAFPKGTIVAAFLG
eua Treeshrew    MRVAGKRLKEGDMMMGQQVA(R)VVESKNSAWPTGTVVLASSG
eua Mouse        MRVAAKKLKEGDRMMGEQVA(R)VVESKNSAFPKGTIVAALLG
eua Rat          MRVAAKKLKEGDSMMGEQVA(R)VVESKNSAFPTGTIVVALLG
eua Kangaroo_rat MRVAAKRLKEGDMMMGEQVA(R)VVESKNSAFPTGTIVLGFLG
eua Rabbit       MRLGSKRLKEGDTMMGQQVA(R)VVESKNPAWPVGTLVLAHSG
lau Dog          MRVAAKRLKEGDMMMGQQVA(R)VVESKNSAFPAGTVVVASSG
lau Cat          XXVAAKRLKEGDTMMGQQVA(R)VVESKNSAFPTGTMVVASSG
lau Horse        MRIAATKLKEGDVMMGQQVA(R)VVESQNSAFPTGTIVLAHSG
lau Cow          MRIMAKSLKEGDMMMGEQVA(R)VVESKNSAFPTGTIVLAPSG
lau Alpaca       MRLASKRLKEGDKMIGQQVA(R)VVESKNSAFPVGTIVLVQSG
lau Microbat     MRLAAKRLKEGDTLMGQQVA(R)VVESKNSAFPTGTVVLASSG
lau Shrew        MRVASKNLKEGDPMMGQQVA(R)VLESKNSTFPTGTTVVAFSG
afr Elephant     MRIAAKRLKEGDMMMGQQVA(R)VVESKNSAFPTGTIVLASSG
afr Rock_hyrax   MRIVAKRLKEGDTMIGQQVA(R)VVESKNSAFPKGTIVLAQSG
afr Tenrec       MRVAAKKMKEGEKMMGQQVA(R)VVESKNSAFPVGTTVVASSG
xen Armadillo    MRVASKKLKEGETMMGEQVA(R)VVESKNSDFPKGTVVLAFSG
pro Platypus     MRVAAMTLQEGDTMSGGQVA(R)VVESKNAAFPVGSIVVASSG

NO 192
GN PTPN1
IP IPI00297261.3
PE CPIKEEKGSPLNA
MP 350
EX Kim
CL apes
DE protein tyrosine phosphatase, non-receptor type 1
SQ
pri Human        NHQWVKEETQEDKDCPIKEE(K)GSPLNAAPYGIESMSQDTEV
pri Chimpanzee   NHQWVKEETQEDKDCPIKEE(K)GSPLNAAPYGIESMSQDTEV
pri Gorilla      NHQWVKEETQKDKDCPIKEE(K)GSPLNAAPYGIESMSQDTEV
pri Orangutan    NHQWVKEETQEDKDCPIKEE(K)GSPLNAVPYGMESMSQDTEV
pri Gibbon       NHQWVKDETQEDKDCPIKEE(K)GSPLNATPYSMESMSQDTEV
pri Rhesus       NHQWVKDETQEDKDCPIKEE(T)GSPLNAAPYSMESMSQDTEV
pri Baboon       NHQWVKDETQEDKDCPIKEE(A)GSPLNAAPYSMESMSQDTEV
pri Marmoset     NHQWVKDETGEDKACPVKEE(T)GSPLNATPYSMESMSQDTEV
pri Tarsier      NHQWVREDPQDDTDGPIREE(A)RSPLHV-PCSLESMSQDTEV
pri Bushbaby     NHQWVKDETEEDKDCPIEEE(I)RSPLNV-PHSVESTSQDTEV
pri Mouse_lemur  NHQWVKDETEEDKDHSIKEE(T)RSPLNV-PYSTESTSQDTEV
eua Treeshrew    NHQWVKDETEEDKDDPIEEE(T)RSPLNV-PYSPESTSQDTEV
eua Mouse        SHQWVSEETCGDEDSLAREE(G)RAQSSA-MHSVSSMSPDTEV
eua Rat          NHQWVSEESCEDEDILAREE(S)RAPSIA-VHSMSSMSQDTEV
eua Guinea_pig   NHQWVKDEKEDGKDGPVKEE(T)RTLLSV-PYSVDSRSRDTEI
eua Pika         NHQWVKDAAEESTDCPTKED(S)RASLST-PYSLESMSQDTEV
lau Dog          NHQWVKDEPGEDKEASIKEE(T)RTPLNV-PCSVESTSPDTEV
lau Cat          NHQWVKDETGEDKEDPIKEE(T)KTPLNV-PCSVESTSPDTEV
lau Horse        NHQWVKEATEEDKDDPIQEE(T)RTPLSV-PCSMESNRQDTEV
lau Cow          NHQWVKDDTEEDKEDPVQEA(T)RAPLNA-PCSLESASQDTEV
lau Dolphin      NHQWVTAETEEVKEDPVKEE(T)RTLLTA-PCSLESASQDTEV
lau Alpaca       NHQWVKDEPEEDKEDPIKEE(T)RTPLNV-PCSVESMSQDTEV
lau Microbat     NHQWVKDESEEDKEDPIKEE(T)RTPLGV-PCNMESASQDTEV
lau Hedgehog     NHQWLKDEPKEDNIDPSKEG(I)RAPLTA-PRSLESTSPDTEV
lau Shrew        NHQWAKEESEADKVDPIKEE(T)QTLLSV-PPSMESTSQDTEV
afr Elephant     NHQWVKDETEDGGEGAMEAE(T)RTPLNA-SHGVESSSQDTEV
afr Tenrec       NHQWVKEEAGDSREAPIETK(I)RAPLTV-SHSTESSSQDTEV
xen Armadillo    NHQWVKDEMEEDKEGPIEEE(T)RIPLNA-SRNMESTSPDTEA
mar Opossum      NHQWEINEMEEEQDGPIIEE(S)IPSLTV-PLTTESTSQDTGV

NO 193
GN PTRH2
IP IPI00032903.3
PE PKSKTSKTHTDTE
MP 47
EX Kim, Wagner
CL primates
DE peptidyl-tRNA hydrolase 2
SQ
pri Human        MCLGWSLRVCFGMLPKSKTS(K)T--HTDTESEASILGDSGEY
pri Chimpanzee   MCLGWSLRVCFGMLPKSKTS(K)T--HTDTESEASILGDSGEY
pri Gorilla      MCLGWSLRVCFGMLPKSKTS(K)T--HTDTESEASILGDSGEY
pri Orangutan    MCLGWSLRVRFGMLPKSKTS(K)T--HTDTESEASILGESGEY
pri Gibbon       MCLGWSLGVRFGMLPKSKTS(K)T--HTDTESEASILGESGEY
pri Rhesus       VCLGWSLRVRFGMLPKSKTS(K)T--HTDTESEASILGESGEY
pri Baboon       VCLGWSLRVRFGMLPKSKTS(K)T--HTDTESEASILGESGEY
pri Marmoset     ICLGWSLRVRFGMFPKSRMS(K)I--HTETETEASILGESGEY
pri Bushbaby     MCLGWGLRVRFGMLSKGLTS(K)T--DTETGTEASISGENGEY
pri Mouse_lemur  MCLGWGLRVRFGMLSKGLTS(K)T--DTETGTEASVLGENGEY
eua Treeshrew    MCLGWGLRVRFGMFPKSSAS(G)T--NAETGTEASILGESGEY
eua Mouse        MCLGWGLRSHLGMFPQNSTS(E)ANRDTETGTEASILGESGEY
eua Rat          MCLGWGLRSHLGIFPQNSTS(E)TNRDTEMGTEASILGESGEY
eua Guinea_pig   VCLGWSLRVRFRMAPKNMTS(E)M--DTDAGTEASILGESGEY
eua Squirrel     ICLGWGLRVRFGMLSENSTS(Q)T--HTDTGTEASILGETGEY
eua Rabbit       MCLGWGLRVRFGMLPKSLTS(E)T--DTESETEESILGESEEY
eua Pika         MCLGWGLRVRFGMLPKRLTN(E)A--DTESDTEETTLGESEEY
lau Dog          MCLGWGLRVRFGMIPRSSVS(E)T--DTETGSEASILGESGEY
lau Horse        MFVGWGLRTHFGLTPESSVS(K)T--DTETGTEASILGESGEY
lau Cow          VCLGWGLRMRFGMLPKSSVR(E)T--NPDTETEASILGESGEY
lau Dolphin      MCLGWRLRERFRMIPKSSVS(E)T--DTDTRTEASILGEGGEY
lau Alpaca       MCLGWGLRARFGMTPRSSVG(E)T--DTDTGTEASILGESGEY
lau Megabat      MCLGWGLRVRFGMIPKSSVS(E)K--ERETGTEASILGESGEY
lau Microbat     MCLGWGLRVRFGMIPKSSVN(E)T--NTETRTEASILGESGEY
lau Hedgehog     MCLGWSLRVRFGMIPKTSVI(E)K--DKETGPEASILGESGEY
lau Shrew        MFLGWGLRVRFGMIPKTPVN(E)T--ERQTGTEASVLGEGGEY
afr Elephant     MCLGWGLRVRFGMIPKSSVN(E)T--DRETGTEASILGESGEY
afr Rock_hyrax   MCLGWGLRVRFGMIPRSSVN(E)T--DRQTGAEASILGETGEY
xen Sloth        MCLGWGLHIRFGMIPKSSVK(K)A--DTDTGDEASILGESGEY

NO 194
GN PYGB
IP IPI00004358.4
PE DHLPELKQAVDQI
MP 740
EX Kim
CL primates
DE phosphorylase, glycogen; brain
SQ
pri Human        EALDRKGYNAREYYDHLPEL(K)QAVDQISSGFFSPKEPDCFK
pri Chimpanzee   EALDRKGYNAREYYDHLPEL(K)QAVDQISSGFFSPKEPDCFK
pri Gorilla      EALDRKGYNAREYYDHLPEL(K)QAVDQISSGFFSPKEPDCFK
pri Orangutan    EALDRKGYNAREYYDHLPEL(K)QAVDQISSGFFSPKEPNCFK
pri Gibbon       EALDWKGYNAREYYDHLPEL(K)QAVDQISSGFFSPKEPDCFK
pri Rhesus       EALDRKGYNAREYYDRLPEL(K)QAVDQISSGFFSPKEPDCFK
pri Baboon       EALDRKGYNAREYYDRLPEL(K)QAVDQISSGFFSPKEPDCFK
pri Marmoset     EALDQKGYNAREYYDRLPEL(K)QAVDQISSGFFSPKEPDCFK
pri Mouse_lemur  EALDRKGYNAREFYDRLPEL(K)QAVDQISSGFFSPREPDCFK
eua Treeshrew    XXXXXXXYNAREYYERLPEL(R)QAVDQISSGFFSPKDPDCFR
eua Mouse        EALDQKGYNAREFYERLPEL(R)QAVDQISSGFFSPKDPDCFK
eua Rat          EALDQKGYNAREFYERLPEL(R)QAVDQISSGFFSPKDPDCFK
eua Kangaroo_rat EALDQKGYNAREFYERLPEL(R)QAVDQIGSGFFSPKDPDCFK
eua Guinea_pig   EALDQKGYNAREYYERLPEL(R)QAVDQISSGFFSPKNPECFK
eua Rabbit       EALDRKGYVAKEYYERLPEL(R)QAVDQIRGGFFSPKEPDCFK
lau Dog          EALDQKGYNAREYYERLPEL(R)QALDQISSGFFSPKDPHCFR
lau Cat          EALDRKGYNAREYYDRLPEL(K)QAMDQISSGFFSPKDPDCFR
lau Horse        EALDQKGYHAREYYDRLPEL(R)QAVDQISSGFFSPKEPDCFK
lau Cow          EALDRKGYNAHEYYDRLPEL(R)QAVDQINGGFFSPREPDCFK
lau Dolphin      EALDRKGYNAREYYDSLPEL(R)QAVDQISSGFFSPREPDCFK
lau Megabat      EALDQKGYHAQEYYDRLPEL(R)QAVDQISSGFFSPKEPDCFK
lau Microbat     QALDQKGYNAQEYYDRLHEL(R)QAVDQIRDGFFSPQEPDCFK
lau Hedgehog     EALDRKGHTPLEYYERLPEL(S)PGLDQIGSGFFSPQEPDCVQ
afr Elephant     EALDQKGYNAREYYDRLPEL(R)QAVDQIGSGFFSPKEPDCFR
afr Rock_hyrax   EALDQKGYNAKEYYDRLPEL(R)QAVDQISSGFFSPKEPDCFR
afr Tenrec       EALDRKGYNAREFYDRLPEL(R)QAVDQISNGFFLPKEPDCFK
xen Armadillo    EALDRKGYDACHYYERLPEL(R)QAVDQIGSGFFSPKEPGCFQ
mar Opossum      EALDKKGYNAKEYYDRIPEL(K)QVMDQISSGYFSPKDPDCFK

NO 195
GN RABGGTB
IP IPI00295849.3
PE KVVEYVKGLQKED
MP 124
EX Kim
CL simians
DE Rab geranylgeranyltransferase, beta subunit
SQ
pri Human        ILTLYDSINVIDVNKVVEYV(K)GLQKEDGSFAGDIWGEIDTR
pri Chimpanzee   ILTLYDSINVIDVNKVVEYV(K)GLQKEDGSFAGDIWGEIDTR
pri Gorilla      ILTLYDSINVIDVNKVVEYV(K)GLQKEDGSFAGDIWGEIDTR
pri Orangutan    ILTLYDSINVIDVNKVVEYV(K)GLQKEDGSFAGDIWGEIDTR
pri Gibbon       ILTLYDSINVIDVNKVVEYV(K)GLQKEDGSFAGDIWGEIDTR
pri Rhesus       ILTLYDSINVIDVNKVVEYV(K)GLQKEDGSFAGDIWGEIDTR
pri Baboon       ILTLYDSINVIDVNKVVEYV(K)GLQKEDGSFAGDIWGEIDTR
pri Marmoset     ILTLYDSINVIDVNKVVEYV(K)SLQKEDGSFAGDIWGEIDTR
pri Tarsier      XXXXXXXXXXXDVNKVVEYV(Q)SLQREDGSFAGDIWGEIDTR
pri Bushbaby     ILTLYDSINVIDVNKVVEYV(Q)SLQKEDGSFAGDIWGEIDTR
pri Mouse_lemur  ILTLYDSINVIDVNKVVEYV(Q)SLQKEDGSFAGDIWXXXXXX
eua Mouse        ILTLYDSVHVINVDKVVAYV(Q)SLQKEDGSFAGDIWGEIDTR
eua Rat          ILTLYDSIHVINVDKVVAYV(Q)SLQKEDGSFAGDIWGEIDTR
eua Kangaroo_rat XXTLYDSINVIDIDKVVEYV(Q)SLQKEDGSFAGDVWGEIDTR
eua Guinea_pig   ILTLYDSINVIDVNKVVEYV(Q)SLQKEDGSFAGDIWGEIDTR
eua Rabbit       ILTLYDSINVIDVNKVVEYV(Q)SLQKEDGSFAGDIWGEIDTR
lau Dog          ILTLYDSINVIDVNKVVEYV(Q)SLQKEDGSFAGDIWGEIDTR
lau Horse        ILTLYDSINVIDVNKVVEYV(Q)SLQKEDGSFAGDTWGEIDTR
lau Cow          ILTLYDSINVIDINKVVEYV(Q)SLQKEDGSFAGDIWGEIDTR
lau Dolphin      ILTLYDSINVIDVNKVVEYV(Q)SLQKEDGSFAGDIWGEIDTR
lau Alpaca       ILTLYDSINVIDVNKVVEYV(Q)SLQKEDGSFAGDIWGEIDTR
lau Megabat      ILTLYDSINVIDVNKVVEYV(Q)SLQKEDGSFAGDIWGEIDTR
lau Microbat     ILTLYDSINVIDVNKVVEYV(Q)SLQKEDGSFAGDIWGEIDTR
lau Hedgehog     ILTLYDSVNAIDVDKVVEYV(Q)SLQKEDGSFAGDTWXXXXXX
lau Shrew        ILTLYDSINVIDVSKVVEYV(Q)SLQKEDGSFAGDIWXXXXXX
afr Elephant     ILTLYDSINAIDVNKVVEYV(Q)SLQKEDGSFAGDTWGEIDTR
afr Rock_hyrax   ILTLYDSINTIDVNKVVEYV(Q)SLQKEDGSFAGDIWXXXXXX
xen Sloth        ILTLYDSINVIDVNKVVEYV(Q)SLQKEDGSFAGDIWGEIDTR
mar Opossum      ILTLYDSLNVIDVNKVVEYV(Q)SLQKEDGSFAGDIWGEIDTR
mar Wallaby      ILTLYDSLNVIDVNKVVEYV(Q)SLQKEDGSFAGDIWGEIDTR
pro Platypus     ILTLYDSLHVIDVDKVVEYV(Q)SLQKEDGSFAGDIWGEIDTR

NO 196
GN RAD18
IP IPI00024579.1
PE ALESPAKSPASSS
MP 102
EX Kim, Wagner
CL simians
DE RAD18 homolog (S. cerevisiae)
SQ
pri Human        VKSLNFARNHLLQFALESPA(K)SPASSSSKNLAVKVYTPVAS
pri Chimpanzee   VKSLNFARNHLLQFALESPP(K)SPASSSSKNLAVKVYTPVAS
pri Gorilla      VKSLNFARNHLLQFALESPP(K)SPASSSSKNLAVKVYTPVAS
pri Orangutan    VKSLNFARNHLLQFALESPP(K)SPASSSSKNLAVKVYTPVAS
pri Gibbon       VKSLNFARNHLLQFALESPP(K)SPASSSSKNLAVKVYTPVAS
pri Rhesus       VKSLNFARNHLLQLALESPP(K)SPASSSSKNLAVKVCTPVAS
pri Baboon       VKSLNFARNHLLQLTLESPP(K)SPASSSSKNLAVKVYTPVAS
pri Marmoset     VKSLNFARNHLLQFALESPP(K)FPASSSSKNLAVKVDTPIAS
pri Tarsier      XXXXXXXXNHLLQFALESPP(I)SPASSSSKNLTVKVHTPISF
pri Bushbaby     VKSLNFARNRLLQFALESPP(I)SPASSSLKNLAVKAHTPVVY
pri Mouse_lemur  VKSLNFARNHLSQFALEAPP(I)SPASSSSKHLAAKAHTPVAF
eua Treeshrew    VKSLNFARNHLWQFALESPP(V)SPXSSSSKNLTVKVHTPAAF
eua Mouse        VKSMNFARTHLLQFALESPP(I)SPVSSTSKKVVVKVHNADAA
eua Rat          VKSMSFARTRLLQFALESPP(I)SPVSSTSKKVVVKVHNVEAT
eua Kangaroo_rat VKSMSFARNRLLRFTLESPP(L)SPASSSSKKLAIKVSTPGVF
eua Guinea_pig   VKSLKLARNHLLQFALESLP(I)SPASSS-KKLAVKVQSPGAF
eua Squirrel     VKSLNFARNHLLQFALESPP(I)SPASSSSKKLAVKVHTPGGY
eua Rabbit       VKSLHFARSHLLQSALESPP(I)SPVSSSAKKLTGRALTPAAA
eua Pika         VKSLNFARDRLLQLALESPP(V)SPVSSSAKRLAVRAPTSVAS
lau Dog          VKSLNFARNHLLQFALESPP(I)SPASSSSKNLAAKVNTPVAF
lau Horse        VKSLNFARNHLLQFALESPP(I)SPASSSSKNLAAKVHTPVAF
lau Cow          VKNLNFARNHLLQFALESPP(I)SAASSSSKNLATKADTAVAF
lau Dolphin      VKSLNFARNHLLQFALESPR(I)SSASSSSKNLAAKVHTSVAF
lau Alpaca       VKSLNFARNHLLQFALESPP(I)SPASSSSKNLAAKVHTPVAF
lau Megabat      VKSLNFARNHL-QFALESSP(I)SRASSSSKNLAAKVHSPVAL
afr Elephant     VKSVNFARNHLLQFALESPP(T)SPVSTSSKNLAVKVHIPVGS
afr Rock_hyrax   VKS-NFARNHLLQFALESPP(T)SPASCSSKNLAAKVRVPVAS
afr Tenrec       VKSLNFARSHLLQFALESPP(T)SPAAASSKNLAIKASTPVTS
xen Armadillo    VKGLHFARNHLLQLALESPP(I)SPASSSSKNLVVNVHNPIAF
xen Sloth        VKGLNFARNHLLQFALESPP(M)SPASSSSKNHAVKVHNPLAS
mar Opossum      VKSFSAARNHLLQYTLDSPP(I)SPLSSSIKNISGKIHIPKVH
mar Wallaby      VKSFSSARNHLFQFTLDSPP(I)SPLSSCTKNISGKIQVPKVH

NO 197
GN RAD18
IP IPI00024579.1
PE PDPSEAKRPEPPS
MP 186
EX Kim, Wagner
CL catarrhines
DE RAD18 homolog (S. cerevisiae)
SQ
pri Human        AAKTKETRSVEEIAPDPSEA(K)RPEPPSTSTLKQVTKVDCPV
pri Chimpanzee   AAKTKETRSVEEIAPDPSEA(K)RPEPPSTSTLKQVTKVDCPV
pri Gorilla      AAKTKETRSVEEIAPDPSEA(K)RPEPPSTSTLKQVTKVDCPV
pri Orangutan    AAKTKETRSVEEIAPDRSEA(K)RPETPSTSTLKQVTKVDCPV
pri Gibbon       AAKTKETRSVEEIAPDPSEA(K)RPETPSTSTLKQVTKVDCPV
pri Rhesus       AAKTKETRSVEEMAPDPSEV(K)RPETPSTSTLKHITKVDCPV
pri Baboon       AAKTKETRSVEEMAPDPSEV(K)RPETPSTSTLKHITKVDCPV
pri Marmoset     AAKTKETRSVEEMAPGSSEA(Q)RPETPSTSTLKQVTKVDCPV
pri Tarsier      ATETKETHSVERMAPGSSDA(S)GSETPSTSTLKQVVKVDCPV
pri Bushbaby     TAESRETHSVEKMTPGSSEA(N)GPETPSTSTLKQVTKVDCPV
pri Mouse_lemur  TAETVETHSVGKMAPGSSDA(R)GPESPSTSTLKLVTKVDCPV
eua Treeshrew    SVETAEPCSVPRMAPGPSGA(H)DPETPSTSTLKYVTRVDCPV
eua Mouse        SAEIKETSLLGKPVLGLSDA(N)GPVTPSTSTMKLDTKVSCPV
eua Rat          SADIKETSLLGKTALGLSDA(N)GPVTPSTSTLKLDTKVSCPV
eua Kangaroo_rat STETKKTGFVEEVAPGSSDV(N)GPVTPSTSALKQDTKVDCPV
eua Guinea_pig   SIETKDTCFVENMTPGYSDT(N)RPEIPSTSFLKQVVKVDCPV
eua Squirrel     SLETKAACFMGKTALGFSDT(N)CHEIPSTSTLKQVSKVDCPV
eua Rabbit       SAEPKETCFPEKVTPGSMEA(N)GPETPSTSTLKQVAKVDCPV
eua Pika         SAESTGTCFSEPMTPSSSEA(R)RPETPSTSTLKQVTKVDCPV
lau Dog          SSKTKETPSIEKTAPRSLDA(H)VPETPSTSTLKQVTRVDCPV
lau Cat          SSKTKEAPSVEKTAPHSLVA(N)VPETPSTSTLKQAAKVDCPV
lau Horse        SAKTKETPSVEKTTLGSLDA(N)VPETPSTSTLKQVTKVDCPV
lau Cow          PAKTRGTPSAVKSAPGSSED(S)VLETPSTSALKQVTKVDCPV
lau Dolphin      SAKTRGTPSVEKSAPGFSEA(S)VPETPSTSALKEVTKVDCPV
lau Megabat      SVKTKETPSVLKTAPGIANA(K)VPEAPSTSTLKQVAKVDCPV
lau Hedgehog     PAKVRATASVETAAPGSSDA(P)GPETPSTSTLKQVTKVDCPV
afr Tenrec       FARNKETPSVEKTPPGPSGV(S)VPETPSTSTAKQATKVDCPV
xen Sloth        YAKTRETASLEKMVSGPSGA(N)VPETPSTSTSKQVTKVDCPV
mar Opossum      SPEIKGASREAREAQRSTSL(N)GPEKPSTSAFKEVTKVECPV
mar Wallaby      SAELKGASGEEREAQRSISV(N)GPEKPSTSAFKEVPKVECPV

NO 198
GN RAD18
IP IPI00024579.1
PE KYRKKHKSEFQLL
MP 347
EX Kim
CL primates
DE RAD18 homolog (S. cerevisiae)
SQ
pri Human        TKDQTEKEIDEIHSKYRKKH(K)SEFQLLVDQARKGYKKIAGM
pri Chimpanzee   TKDQTEKEIDEIHSKYRKKH(K)SEFQLLVDQARKGYKKIAGM
pri Gorilla      TKDQTEKEIDEIHSKYRKKH(K)SEFQLLVDQARKGYKKIAGM
pri Orangutan    TKDQTEKEIDEIHSKYRKKH(K)SEFQLLVDQARKGYKKIAGM
pri Gibbon       TKDQTEKEIDEIHSKYRKKH(K)SEFQLLVDQARRGYKKISGM
pri Rhesus       TKDQTEKEIDEIHSKYRKKH(K)CEFQLLVDQARKGYKKIVGM
pri Baboon       TKDQTEKEIDEIHSKYRKKH(K)CEFQLLVDQARKGYKKIVGM
pri Marmoset     TKDQTEKEIDEIHSKYRKKH(K)SEFQFLVDQARKEYKKTVGM
pri Tarsier      TKDQTEDQIDEIHSEYRKKY(K)NELQLLVDQAKKGYKKTVGI
pri Mouse_lemur  TKDQTEKEIEEIHSKYKKXX(K)NEFQLLVDQATKECKKTVGM
eua Treeshrew    TKDQTEEEIDEIHSKYRKKH(Q)NEFQLLVDQAKRGYKKTAGT
eua Mouse        TKNQTEKEIEEVHSEYRKKH(Q)NAFQLLVDQAKKGYKKTGRV
eua Rat          TKNQTEKEIEEIHSKYRKKH(Q)NEFQLLVDQAKKGYKKTGRM
eua Guinea_pig   TKDQTEEEIDEIHRKYRKKH(R)NEFQLLVDQAKKGYKKTGGV
eua Squirrel     TKDQTEKEIDEIHSKYRKTH(Q)NEFRLLVDQAKKGYKKTDGM
eua Rabbit       TKDQTEEEIDEIHSKYRKKH(H)NEFQLLVDQAKKGYKKAAGM
eua Pika         TKDQTEEEIDEIHSKYRKKH(H)SEFQRLVDQAKKGYKKAVGM
lau Dog          TKDQTEKEIDEIHSRYRKKH(Q)NEFRLLVDQAKKGYKKTVEM
lau Horse        TKGQTEKEIDEIHSKYRKKH(Q)NEFQLLVDQAKKGYKQAVGM
lau Cow          TKNQTEKEIDEIHSKYRKKH(Q)DEFHLLVDQAKKGYKKTVGM
lau Dolphin      TKDQTEKEIDEIHSNYRIKH(Q)NEFQRLVDQAKKGYKKTVGM
lau Alpaca       TKDQTEKEIDEIHSKYRKKH(Q)NEFQLLVDQAKQGYKKTVGT
lau Megabat      TKDQTEKEIDEIHSKYRKKH(Q)NEFQLLVDQAKKGYKKTVGM
lau Microbat     TKDQTEKEIDEIHSKYRKKH(Q)NEFQLLVDQAKKGYKKTVEM
afr Elephant     TKDQTEKEIDEIHSTYRKKH(Q)NEFQLLVDQARKGYKKTVEM
afr Rock_hyrax   TKDQTEKEIDEIHSIYRKKY(H)NEFQRLVDQAKKGYKKTVEM
afr Tenrec       TKDQTEKEIDEIHRTYRKKH(Q)NEFHLLVDQAKKGYKKTIGV

NO 199
GN RARS2
IP IPI00873116
PE LLLSDYKFSWDRV
MP 446
EX Wagner
CL simians
DE arginyl-tRNA synthetase 2, mitochondrial
SQ
pri Human        RVGLAALIIQDFKGLLLSDY(K)FSWDRVFQSRGDTGVFLQYT
pri Chimpanzee   RVGLAALIIQDFKGLLLSDY(K)FSWDRVFQSRGDTGVFLQYT
pri Gorilla      RVGLAALIIQDFKGLLLSDY(K)FSWDRVFQSRGDTGVFLQYT
pri Orangutan    RVGLAALIIQDFKGLLLTDY(K)FSWDRVFQSRGDTGVFLQYT
pri Gibbon       RVGLAALIIQDFKGLLLSDY(K)FSWDRVFQSRGDTGVFLQYT
pri Rhesus       RVGLAALIIQDFKGLLLSDY(K)FSWDRVFQSRGDTGVFLQYT
pri Baboon       RVGLAALIIQDFKGLLLSDY(K)FSWDRVFQSRGDTGVFLQYT
pri Marmoset     RVGLAALIIQDFKGLLLSDY(K)FNWDRVFQSRGDTGVFLQYT
pri Tarsier      RVGLAALIIQDFKGLLLSDY(Q)FSWDRVFQSRGDTGVFLQYT
pri Bushbaby     KVGLAALIIQDFKGLLLSDY(Q)FSWDRVFQSRGDTGVFLQYT
pri Mouse_lemur  RVGLAALIIQDFRGLLLSDY(Q)FSWDRVFQSRGDTGVFLQYT
eua Mouse        RVGLAAVIIQDFRGTLLSDY(Q)FSWDRVFQSRGDTGVFLQYT
eua Rat          KVGLAALIIQDFRGLLLSDY(Q)FSWDRVFQSRGDTGVFLQYT
eua Guinea_pig   RVGLAALIIQDFRGLLLSDY(H)FSWDRVFQSRGDTGVFLQYT
eua Squirrel     RVGLAALIIQDFRGLLLSDY(Q)FSWDRVFQSRGDTGVFLQYT
eua Rabbit       KVGLAALIIQDFRGALLSDY(Q)FSWDRIFQSRGDTGVFLQYT
lau Dog          RVGLAALIIQDFKGLLLSDY(Q)FSWDRVFQSRGDTGVFLQYT
lau Cat          RVGLAALIIQDFKGVLLSDY(Q)FSWDRIFQSRGDTGVFLQYT
lau Horse        RVGLAALIIQDFKGLLLSDY(Q)FSWDRVFQSRGDTGVFLQYT
lau Cow          QVGLAALIIQDFRGFLLSDY(Q)FSWDRVFQSRGDTGVFLQYT
lau Dolphin      RVGLAALIIQDFRGFLLSDY(Q)FSWDRVFQSRGDTGVFLQYT
lau Alpaca       RVGLAALIIQDFRGLLLSDY(Q)FSWDRVFQSRGDTGVFLQYT
lau Megabat      RVGLAALIIQDFRGLLLSDY(Q)FSWDRVFQSRGDTGVFLQYT
lau Microbat     RVGLAALIIQDFRGSLLSDY(Q)FSWDRVFQSRGDTGVFLQYT
lau Hedgehog     RVGLPALFIQDFKGILLSDY(Q)FSWDRVFQSRGDTGVFLQYT
lau Shrew        KVGLAALIIQDFKGSLSSDY(N)FSWDRVFQSRGDTGVFLQYT
afr Elephant     RVGLAALIIQDFRGLLLSDY(Q)FSWDRVFQSRGDTGVFLQYT
afr Rock_hyrax   KVGLAALIIQDFRGLLLSDY(Q)FSWDRVFQSRGDTGVFLQYT
afr Tenrec       RVGLAALIVQDFKGLLLSDY(Q)FNWDRVFQSRGDTGVFLQYT
xen Sloth        RVGLAALIIQDFRGSLLSDY(Q)FSWDRVFQSRGDTGVFLQYT
mar Opossum      RVGLAALIIQDFKGHLLSDY(K)FNWDRVFQSRGDTGVFLQYT
mar Wallaby      RVGLAALIIQDFKGYLLSDY(K)FSWDRVFQSRGDTGVFLQYT
pro Platypus     RVGLAALIVHDLRGLLLSDY(Q)FSWDRVLQTRGDTGVFLQYT

NO 200
GN RBCK1
IP IPI00783058
PE LLEREIKALLTPE
MP 342
EX Wagner
CL simians
DE RanBP-type and C3HC4-type zinc finger containing 1
SQ
pri Human        SCPFIDNTYSCSGKLLEREI(K)ALLTPEDYQRFLDLGISIAE
pri Chimpanzee   SCPFIDNTYSCSGKLLEREI(K)ALLTPEDYQRFLDLGISIAE
pri Gorilla      SCPFIDNTYSCSGKLLEREI(K)ALLTPEDYQRFLDLGISIAE
pri Orangutan    SCPFIDNTYSCSGKLLEREI(K)ALLTPEDYQRFLDLGISIAE
pri Gibbon       SCPFIDNTYSCSGKLLEREI(K)ALLTPEDYQRFLDLGISIAE
pri Rhesus       SCPFIDNTYSCSGKLLEREI(K)ALLTPEDYQRFLDLGISIAE
pri Baboon       SCPFIDNTYSCSGKLLEREI(K)ALLTPEDYQRFLDLGISIAE
pri Marmoset     SCPFIDNTYSCSGKLLEREI(K)ALLTAEDYQRFLDLGISIAE
pri Mouse_lemur  SCPFIDNTYSCSGKLLEREI(R)ALLTPEDYQRFLDLSIAIAE
eua Treeshrew    SCPFIDNTYSCSGKLLEREI(R)ALLPPEDYQRFLDLSVSIAE
eua Mouse        ACPFIDSTYSCPGKLLEREI(R)ALLSPEDYQRFLDLGVSIAE
eua Rat          SCPFIDNTYSCPGKLLEREI(R)ALLSPEDYQRFLDLGVSIAE
eua Kangaroo_rat SCPFIDNTYSCPGKLLEREI(R)ALLSPEDYQRFLDLSVSLAE
eua Guinea_pig   SCPFIDDTYSCPGKLLEREI(R)ALLPPEDYQRFLDLSVSIAE
eua Rabbit       SCPFIDNTYSCPGKLLEREI(R)ALLSPEDYQRFLDLSVSIAE
eua Pika         SCPFIDNTYSCQGKLLEREI(R)ALLSPEEYQRFLDLSVSIAE
lau Dog          SCPFIDNTYSCSGKLLEREI(R)ALLPPEDYQRFLDLGVSIAE
lau Cat          SCPFIDNTYSCSGKLLEREI(R)ALLPPEDYQRFLDLGVSIAE
lau Horse        ACPFIDNTYSCSGKLLEREI(R)ALLSPEDYQRFLDLGVSIAE
lau Cow          SCPFIDNTYSCSGKLLEREI(R)ALLSPEEYQRFLDLGISIAE
lau Dolphin      ACPFIDNTYSCSGKLLEREI(R)ALLSPEDYQRFLDLGISIAE
lau Alpaca       ACPFIDNTYSCSGKLLEREI(R)ALLSPEDYQRFLDLGVSIAE
lau Megabat      ACPFIDNTYSCSGKLLEREI(R)ALLTPEDYQRFLDLGVSLAE
lau Microbat     ACPFIDNTYSCSGKLLEREI(R)ALLTPEDYQRFLDLGVSIAE
lau Shrew        CCPFIDNTYSCSGKLLDREI(R)ALLSPEDYQRFLDLSVSIAE
afr Elephant     SCPFIDNTYSCSGKLLEREI(R)ALLTPEEYQRFLDLGISIAE
afr Rock_hyrax   SCPFIDNTYSCSGKLLEREI(R)ALLTPEDYQRFLDLGVSIAE
afr Tenrec       SCPFIDNTYSCSGKLLEREI(R)ALLSPEDYQRFLDLSISIAE
xen Armadillo    TCPFMDNTYSCSGKLLEREI(R)ALLTPEDYQRFLDLGISIAE
mar Opossum      SCPYIDDTYSCPGKLQEREI(R)ALLSPEDYQRFLDLGISIAE

NO 201
GN RDX
IP IPI00219365.3
PE ELMERLKQIEEQT
MP 352
EX Kim
CL simians
DE radixin
SQ
pri Human        IAEKEKERIEREKEELMERL(K)QIEEQTIKAQKELEEQTRKA
pri Chimpanzee   IAEKEKERIEREKEELMERL(K)QIEEQTIKAQKELEEQTRKA
pri Gorilla      IAEKEKERIEREKEELMERL(K)QIEEQTIKAQKELEEQTRKA
pri Orangutan    IAEKEKERIEREKEELMERL(K)QIEEQTIKAQKELEEQTRKA
pri Gibbon       IAEKEKERIEREKEELMERL(K)QIEEQTVKAQKELEEQTRKA
pri Rhesus       IAEKEKERIEREKEELMERL(K)QIEEQTVKAQKELEEQTRKA
pri Baboon       IAEKEKERIEREKEELMERL(K)QIEEQTIKAQKELEEQTRKA
pri Marmoset     IAEKEKERIEREKEELMERL(K)QIEEQTIKAQKELEEQTRKA
pri Tarsier      IAEKEKERIEREKEELIERL(R)QIEEQTLKAQKELEEQTRKA
pri Bushbaby     IAEKEKERIEREKEELMERL(R)QIEEQTLKAQKELEEQTRKA
pri Mouse_lemur  IAEKEKERIEREKEELMERL(R)QIEEQTLKAQKELEEQTRKA
eua Treeshrew    IAEKEKERIEREKEELMERL(R)QIEEQTMKAQKXXXXXXXXX
eua Mouse        IAEKEKERIEREKEELMERL(R)QIEEQTVKAQKELEEQTRKA
eua Rat          IAEKEKERIEREKEELMERL(R)QIEEQTMKAQKELEEQTRKA
eua Kangaroo_rat IAEKEKERIEREKDELMERL(R)QIEEQTMKAQKELEEQTRKA
eua Guinea_pig   IAEKEKERIEREKEELMERL(R)QIEEQTMKAQKELEEQTRKA
eua Rabbit       IAEKEKERIEREKEELMERL(R)QIEEQTMKAQKELEEQTRKA
eua Pika         IAEKEKERIEREKEELMERL(R)QIEEQTLKAQKXXXXXXXXX
lau Dog          IAEKEKERIEREKEELMERL(R)QIEEQTVKAQKELEEQTRKA
lau Horse        IAEKEKERIEREKEELMERL(R)QIEEQTMKAQKELEEQTRKA
lau Cow          IAEKEKERIEREKEELMERL(R)QIEEQTMKAQKELEEQTRKA
lau Dolphin      IAEKEKERIEREKEELMERL(R)QIEEQTLKAQKELEEQTRKA
lau Alpaca       IAEKEKERIEREKEELMERL(R)QIEEQTMKAQKELEEQTRKA
lau Megabat      IAEKEKERIEREKEELMERL(R)QIEEQTMKAQKELEEQTRKA
afr Elephant     IAEKEKERIEREKEELMERL(R)QIEEQTMKAQKELEEQTRRA
afr Rock_hyrax   IAEKEKERIEREKEELMERL(K)QIEEQTMKAQKELEEQTRRA
afr Tenrec       IAEKEKERIEREKEELVERL(R)QIEEQTMKAQKELEEQTRRA
xen Armadillo    IAEKEKERIEREKEELMERL(R)QIEEQTMKAQKELEEQTRKA
xen Sloth        IAEKEKERIEREKEELMERL(R)QIEEQTMKAQKELEEQTRKA
mar Opossum      IAEKEKERIEREKEELMERL(R)QIEEQTMKAQKELEEQTRRA
mar Wallaby      IAEKEKERIEREKEELMERL(R)QIEEQTMKAQKXXXXXXXXX
pro Platypus     IAEKEKERIEREKEELMERL(R)QIEEQTMKAQKELEEQTRKA

NO 202
GN RECQL4
IP IPI00014925.1
PE LTLLQGKRFQNLD
MP 695
EX Kim
CL simians
DE RecQ protein-like 4
SQ
pri Human        HLSVSMDRDTDQALLTLLQG(K)RFQNLDSIIIYCNRREDTER
pri Chimpanzee   HLSVSMDRDTDQALLTLLQG(K)RFRNLDSIIIYCNRREDTER
pri Gorilla      HLSVSMDRDTDQALLTLLQG(K)RFRNLDSIIIYCNRREDTER
pri Orangutan    HLSVSMDRDTDQALLTLLRG(E)RFRDLDSIIIYCNRREDTER
pri Gibbon       HLSVSMDRDTDQALLTLLQG(E)RFRNLDSIIIYCNRREDTER
pri Rhesus       HLSVSMDRDTDQALLTLLQG(K)RFRNLDSIIIYCNRREDTER
pri Baboon       HLSVSMDRDTDQALLTLLQG(K)RFRNLDSIIIYCNRREDTER
pri Marmoset     HLSVSMDRDTDQALLTLLQG(K)RFRNLDSIIIYCNRREDTER
pri Tarsier      HLSVSMDRDTDQALVTLLQG(D)RFRTLDSVIVYCNRRVDTER
pri Bushbaby     NLSVSIDKDPDKALVTLLQG(D)RFRTLDSIIVYCNRREDTER
pri Mouse_lemur  HLSVSMDRDPEQALVTLLQG(D)RFRALDSIIVYCNRREDTER
eua Mouse        HLSVSMDRDSDQALVTLLQG(D)RFRTLDSVIIYCTRRKDTER
eua Rat          HLSVSMDRDSDQALVTLLQG(D)RFRTLDSIIIYCARRKDTER
eua Kangaroo_rat HLSVSMDRDPEQALVTLLQS(D)RFRSLDSVIVYCNRRETTER
eua Guinea_pig   HLSVSMDRNPDQALVTLLQG(D)RFRSLDSVIVYCNRREDTLR
eua Squirrel     HLSVSVDRNPHQALVTLLQG(D)RFRALDSIIIYCNRREDTER
eua Pika         HLSVSMDREPEQALVTLLQG(D)RFRVLDSIIVYCPRREDTER
lau Dog          HLSVSRDRDPDQALVTLLQS(D)RFRALSSVIVYCHRRGDTER
lau Cat          YLSVSTDRNPDQALVTLLQS(D)RFRDLNSVIVYCNRREDTER
lau Horse        HLSVSMDRDPDQALVTLLQS(E)RFRALDSVIVYCNRREDTER
lau Cow          HLSVSSDRDPDQALVTLLRS(D)RFRALGSVIIYCHRREDTER
lau Dolphin      HLSVSSDRDPEQALVTLLQS(D)RFCALDSVIVYCNRREDTER
lau Alpaca       HLSVSTDRDPDQALVTLLQS(D)RFRALGSVIIYCNRREDTER
lau Megabat      YLSVSMDRDPDQALVTLLQS(D)RFRSLDSVIIYCNRREDTER
lau Microbat     HLSVSMDRDPDQALVTLLRG(D)RFRTLDSVIVYCNRREDTER
lau Hedgehog     HLSVSMDRDPDQALVTLLQS(D)RFRTLNSVIVYCNRREDTER
lau Shrew        QLSVSMDRDPDQALVTLLQS(D)RFRALDSVIVYCNRREDTER
afr Elephant     HLSVSMDRDPEQALVTLLQG(E)RFRSLDSAIVYCNRREDTER
afr Rock_hyrax   RLSVSTDRNPEQALVTLLQS(R)RFRSLNSVIVYCHRREDTER
mar Wallaby      YLSVSLDRDKDQALVTLLKG(E)RFGSLGSIIVYCNRRDETVR
pro Platypus     HLSVSLDRDKDQALVTLLKG(E)RFRSLGSIIVYCNRREETVR

NO 203
GN RGL2
IP IPI00005656.1
PE TGYAAGKGVGGGS
MP 198
EX Kim
CL simians
DE ral guanine nucleotide dissociation stimulator-like 2
SQ
pri Human        AKGQLDRLESFLLQTGYAAG(K)GVGGGSADLIRNLRSRVDPQ
pri Chimpanzee   AKGQLDRLESFLLQTGYAAG(K)GVGGGSADLIRNLRSRVDPQ
pri Gorilla      AKGQLDRLESFLLQTGYAAG(K)GVGGGSADLIRNLRSRVDPQ
pri Orangutan    AKGQLDRLESFLLQTGYAAG(K)GVGGGSADLIRNLRSRVDPQ
pri Gibbon       AKGQLDRLESFLLQTGYAAG(K)GVGGGSADLIRNLRSRVDPQ
pri Rhesus       AKGQLDRLESFLLQTGYAAG(K)GVGGGSADLIRNLRSRVDPQ
pri Baboon       AKGQLDRLESFLLQTGYAAG(K)GVGGGSADLIRNLRSRVDPQ
pri Marmoset     AKGQLDRLESFLLQTGYAAG(K)GVGGGSADLIRNLRSRVDPQ
pri Tarsier      VKGQLDRLESCLLRTGYAAG(E)GVGGGSADLIRNLRSRVDPQ
pri Bushbaby     VKVQLDRLESFLLRTGYAAG(E)GVGGGSADLIHNLRSRVDSQ
pri Mouse_lemur  VKVQLDRLESFLLQTGYAAG(E)GVGGGSADLICNLRSRVDPQ
eua Treeshrew    VKGQLDRLESFLLRTGYAAG(E)GVGGGSADLIRNLRSRVDPQ
eua Mouse        VKGQLDRLESFLLRTGYAAR(E)GVVGGSADLIRNLRARVDPR
eua Rat          VKGQLDRLESFLLRTGYAAR(E)GVVGGSADLIRNLRARVDPR
eua Kangaroo_rat VKGQLDRLESFLLRTGYAAG(Q)GVGEGSADLIRNLRSRVDPH
eua Guinea_pig   VKGQLDRLERFLLRTGYAAG(E)GVGGCGADLIRNLRSRVDPQ
eua Rabbit       VKGQLDRLESFLLRTGYAAG(E)GVGGGTADLIRNLRSRVDPQ
eua Pika         VKGQLDRLESFLLRTGYAAG(E)GVGGGTADLLRNLRSRVDSQ
lau Dog          VKGQLDRLESFLLRTGYAAG(E)GVGGGSADLIRNLRFRVDPQ
lau Horse        VKGQLDRLEGFLLRTGYAAG(E)GVGGGSADLIRNLRSRVALQ
lau Cow          VKGQLDRLESFFLRTGYAAG(E)GIVGGGADLIRNLRSRVDPQ
lau Dolphin      VKGQIDRLESFLLRTGYAAG(E)GVGGGGADLIRNLRSRVDPQ
lau Alpaca       VKGQLDRLESFLLRTGYAAG(E)GVGGDSADLIRNLRSRVDSQ
lau Megabat      VKGQLDRLESFLLRTGYAGG(E)GVGGGSADLIRNLRSRVDPQ
afr Elephant     VKAQLDRLESFLLRTGYAAG(E)GVGGGSAELIHNLRFRVDPQ
afr Rock_hyrax   VKGQLDRLESFLLRTGYAAG(E)GVGGGSAELVRNLRSRVDPQ
afr Tenrec       VKGQLDRLESFFLRTGYAVG(E)GAGGGSAELIRKLRSRVDPQ
xen Armadillo    VKGQLDRLESFLLRTGYAAG(A)GVRGGGADLVRNLRSRVDPQ
mar Opossum      VRGQLDRLESFLLRTGDAAG(E)IVGGGIDDLIRDLRSRVDTP
mar Wallaby      VRGQLDRLESFLLRTGDAAG(E)VVGGDIVDLIRDLRSRVALP

NO 204
GN RGL3
IP IPI00643373.5
PE GSAEAQKAEKLLE
MP 187
EX Kim
CL catarrhines
DE ral guanine nucleotide dissociation stimulator-like 3
SQ
pri Human        DLGSVRTFLGWAAPGSAEAQ(K)AEKLLEDFLEEAEREQ-EEE
pri Chimpanzee   DLGSVRTFLGWAAPGSAEAQ(K)AEKLLEDFLEEAEREQ-EEE
pri Gorilla      DLGSVRTFLGWAAPGSAEAQ(K)AEKLLEDFLEEAEREQ-EEE
pri Orangutan    DLGSVRTFLGWAAPGGAEAQ(K)AEKLLEEFLEEAEREQ-EEE
pri Gibbon       DLGSVRTFLGWAAPGSAEAR(K)AEKLLEDFLEEAEREQ-EEE
pri Rhesus       DLGSVRTFLGWAAPGSAEAR(K)AEKLLEDFLEEAKREQ-EEE
pri Baboon       DLGSVRTFLGWAAPGSAEAR(K)AEKLLEDFLEEAKREQ-EEE
pri Bushbaby     DLSSVRTFLGWAAPGGAEAQ(E)AEKLLEDFLEEAEQEQ-EDE
pri Mouse_lemur  ALSSVRTFLGWAAPGGAEAQ(E)AEKLLEDFLEEAEQEQ-EEE
eua Mouse        NLGNVRIFLGWAAPGGAEAR(E)AEKLLEDFLKEAKGEQTEEE
eua Rat          NLGDVRIFLGWAAPGGAEVR(E)AEKLLEDFLKEAKEEQTEGE
eua Kangaroo_rat DLGTVRIFLGWAAPGGVEAQ(E)AEKLLEEFLKETEREQ-EEE
eua Guinea_pig   DLGRVRAFLGWAAPGGAEAR(E)AEKLLEDFSEETDGEQ-EEE
eua Pika         NLARVQVFLSWAAPGSPEAQ(D)AEKLLEGFLKEVEQEQ-EEE
lau Dog          DLGSVCTFLGWAAPAGPEAQ(E)AEKLLGDFLEEAERKQ-EEE
lau Horse        DLSSVRTFLGWAAPLGAEAR(E)AEKLLGQFLEEAEHAQ-EEA
lau Cow          DLGSVCNFLGWAAPSGAEAQ(E)AEKLLGDFLKEAKTEQ-EEE
lau Dolphin      DLGSVCTFLGWAAPSGAEAR(E)AEKLLGDFLKNAEPQQ-EEE
lau Alpaca       DLGSIRTFLGWAAPLGAEAQ(E)AEKLLGGFLEEADQEQ-EEE
lau Megabat      DLSSVRTFLGWAAPLGAEAL(E)AEKLLGDFLEEAYREQ-EEE
lau Hedgehog     DLQKVQKFLGWASPAGPEAQ(E)AEKLLREFLAEAEQGG-EED
afr Elephant     DLGNVCTFLGWAAPGGAEAR(E)AKRLLGDFLEEAEQEL-EEE
xen Sloth        GLGSIRAFLSWAAPGGAEAQ(E)AEKLLGDFLLEAEQEP-EEK
mar Opossum      NLVQVCTFLRWAAPGGPESQ(E)AERLLRHFQEEEEEEQ-EEE
mar Wallaby      NLVQVCTFLSWAAPGGAESQ(E)AERLLKHFQEEEEQEQ-KE-
pro Platypus     ALHRVCTFLSCLAPGGPEER(E)AKQLLSRFLAEEVLQETEEE

NO 205
GN RHBDD2
IP IPI00010255.3
PE PSGLTLKSEALRN
MP 51
EX Kim, Wagner
CL primates
DE rhomboid domain containing 2
SQ
pri Human        VSGPRLFLLQQPLAPSGLTL(K)SEALRNWQVYRLVTYIFVYE
pri Chimpanzee   VSGPRLFLLQQPLAPSGLTL(K)SEALRNWQVYRLVTYIFVYE
pri Gorilla      VSGPRLFLLQQPLAPSGLTL(K)SEALRNWQVYRLVTYIFVYE
pri Orangutan    VSGPRLFLLQPPLAPSGLTL(K)SEALRNWQVYRLVTYIFVYE
pri Gibbon       VSGPRLFLLQPPLAPSGLTL(K)SEALRNWQVYRLVTYIFVYE
pri Rhesus       VSGPRLFLLQPPLAPSGLTL(K)SEALRNWQVYRLVTYIFVYE
pri Baboon       VSGPRLFLLQPPLAPSGLTL(K)SEALRNWQVYRLVTYIFVYE
pri Marmoset     VSGPRLFLLQPPLAPSSLTL(K)SEALRNWQVYRLVTYIFVYE
pri Tarsier      VYGPCLFLLQPPXAPWGLTL(K)SEAMYNWS--RLVTYIFVXE
pri Bushbaby     VSGPRLFLLQPPLAPSGLSL(K)SEALRNWQXXXXXXXXXXXX
pri Mouse_lemur  VSGPRLFLLQPPLAPSGLSL(K)SEALRNWQVYRLVTYIFVYE
eua Mouse        VSGPRLFLLQPPLAPSGLSL(R)SEALRNWQVYRLVTYIFVYE
eua Rat          VSGPRLFLLQPPLAPSGLSL(R)SEALRNWQVYRLVTYIFVYE
eua Kangaroo_rat VSGPRLFLLPPPLAPSGLSL(R)SEALRNWQXXXXXXXXXXXX
eua Guinea_pig   VSGPRLFLRQPPQASSDLTL(R)SEALRDWQVYRLVTYIFVYE
eua Squirrel     VSGPRLFLLQPPLAPSGLSL(R)SEALRNWQXXXXXXXXXXXX
eua Rabbit       VSGPRLFLLQPPLAPSGLSL(R)SEALRNWQIYRLVTYIFVYE
eua Pika         VSGPRVFLLQPPLAPSGLSL(R)SEALRNWQVNRLVTYLFAYQ
lau Dog          VSGPRLFLLQPPLAPSGLSL(R)SEALRNWQVYRLVTYIFVYE
lau Cat          VSGPRLFLLQPPLAPSGLSL(R)SEALRNWQVYRLVTYIFVYE
lau Cow          VSGPRLFLLQQPLAPSGLSL(R)SEALRNWQVYRLVTYIFVYE
lau Dolphin      VSGPRLFLLQPPLAPSGLSL(R)SEALRNWQVYRLVTYIFVYE
lau Megabat      VSGPRLFLLQPPLPPSGLSL(R)SDCLRNWQVYRLVTYIFVYE
lau Microbat     VSGPRLFLLQPPLPPSGLSL(R)SDCLRNWQVYRLVTYIFVYE
lau Hedgehog     VSGPRLFLLPQPAAPSGLSL(K)SEALRDWQVYRLVTYIFVYE
lau Shrew        VSGPRLLLMSRPLAPSNLSL(K)NEALRNWQVYRLVTYIFVYE
afr Rock_hyrax   VSGPRLFLLQPPLAPSGLSL(Q)SEALRNWQVYRLVTYIFVYE
afr Tenrec       VSGPRLFLLQPPLAPSGLSL(R)SEALRNWQVYRLVTYIFVYE
xen Armadillo    VSGPRLFLLQPPLAPSGLSL(R)SEALRNWQVYRLVTYIFVYE
mar Opossum      VSAPRLLLPPQPPPPSGLAL(R)PAALRDWEVYRLVTYILVYE

NO 206
GN RHBDD3
IP IPI00215831.2
PE TLVTHGKGGPAHS
MP 374
EX Kim
CL primates
DE rhomboid domain containing 3
SQ
pri Human        GAVSLLVGGQVGTETLVTHG(K)GGPAHSEGPGPP*
pri Chimpanzee   GAVSLLVGGQVGTETLVTHG(K)GRPAHSEGPGPP*
pri Gorilla      GAVSLLVGGQVGTETLVTHG(K)GGPAHSKGPGPP*
pri Rhesus       GAVSLLVGGQVGTETLVTQG(K)GGPAHSEGPGPP*
pri Baboon       GAVSLLVGGQVGTETLVTQG(K)GGPAHSEGPGPP*
pri Marmoset     GAVSLLVGGQVGTEALVTQG(K)GGPAYSEGPGPP*
pri Bushbaby     GAVSLLVGGQVGTEALVTEG(K)CGPAHPEGPGPP*
eua Mouse        GAVSLLVEGLVDTEALVTEG(R)SSPAHCTGTGAS*
eua Rat          GAVSLLVEGLVDTEALVTEE(R)SGPAHCKDTGVS*
eua Guinea_pig   GAVSLLVGGQVDTGALVTEG(R)GLPANPKGPGPP*
eua Rabbit       GAVSLLVGGQVDTEVLVTEG(R)AGSAHPKGPGPP*
eua Pika         GAVSLLVGGQVDTEALMTEG(R)TGSAYSMGIKPS*
lau Dog          GAVSLLVGGEVGPEALVTQG(R)GGPAHPEGPGPP*
lau Horse        GAVSLLVGGQVGAEALVTEG(R)DRPAHSEGPGPP*
lau Cow          GAVALLVGGEVGTEALVTQG(R)EGPTHPEGPGPP*
lau Dolphin      GAVSLLVGGDVGTEALVTQG(R)GGPTHPEGPGPP*
lau Megabat      GAVSLLVGGEVGTEALVTQG(R)GGPAHPEGPGPP*
afr Elephant     GAVSLLVDGQVGAEALVTEG(K)GQPAHPEGPXPP*
xen Armadillo    GAVSLLVGGQVGAEALVTEG(R)VQPAYPEGPGPP*

NO 207
GN RIOK2
IP IPI00306406.4
PE ELFPTFKDIRRED
MP 288
EX Kim
CL apes
DE RIO kinase 2 (yeast)
SQ
pri Human        CIKDFFMKRFSYESELFPTF(K)DIRREDTLDVEVSASGYTKE
pri Chimpanzee   CIKDFFMKRFSYESELFPTF(K)DIRREDTLDVEVSASGYTKE
pri Gorilla      CIKDFFMKRFSYESELFPTF(K)DIRREDTLDVEVSASGYTKE
pri Orangutan    CIKDFFMKRFSYESELFPAF(K)DIKREDTLDVEVSASGYTKE
pri Gibbon       CIRDFFMKRFSYESELFPTF(K)DIRREDTLDVEVSASGYTKE
pri Rhesus       CIRDFFMKRFSYESELFPTF(R)DIRREDTLDVEVSASGYTKE
pri Baboon       CIRDFFMKRFSYESELFPTF(R)DIRREDTLDVEVSASGYTKE
pri Marmoset     CIRDFFLKRFSYESELFPTL(R)DIRREDTLDVEVSASGYTKE
pri Tarsier      CIRDFFMKRFGYESELYPTF(N)DIRREDSLDVEVSASGYTKE
pri Bushbaby     CIHDFFMKRFGYESELYPAF(S)DIXXXXXXXXXXXXXXXXXX
pri Mouse_lemur  CIRDFFMKRFGYESELYPTF(S)DIXXXXXXXXXXXXXXXXXX
eua Treeshrew    CIRDLFLKRFGYKSELYPTF(S)DIXREDSLDVEVSASGYTKE
eua Mouse        CIREFFMKRFSYESELYPTF(S)DIRKEDSLDVEVSASGYTKE
eua Rat          CIREFFLKRFNYESELYPTF(S)DIRREDSLDVEVSASGYTKE
eua Guinea_pig   CIRDFFMRRFGYESELYPAF(S)DIRREGSLDVEVSASGYTKE
eua Rabbit       CVRDFFIKRFNYESELYPTF(S)DIRREDSLDVEVSASGYTKE
eua Pika         CVREFFIKRFNYESELYPTF(S)DIRREDSLDVEVAASDYTKA
lau Dog          CIRDFFMKRFSYESELYPTF(S)DIRREHSLDVEVSASGYTKE
lau Horse        CIRDFFMKRFSYESELYPSF(S)DVRREDCLDVEVCASGCAEE
lau Cow          CIRDFFMKRFNYESELFPTF(S)DIRREDSLDVEVSASGYTKE
lau Dolphin      CIRDFFMKRFSYESELFPTF(S)DIRREDSLDVEVSASGYTKE
lau Alpaca       CIRDFFMKRFSYESELFPTF(S)DIRREDSLDVEVSASGYTKE
lau Megabat      CIRDFFIKRFSYESELYPTF(S)DIRREDSLDVEVSASGYTKE
lau Microbat     CIRDFFMKRFSYESELYPTF(S)DIXXXXXXXXXXXXXXXXXX
lau Hedgehog     CIRDFFIKRFSYESELYPTF(S)DIRREESLDVEVSASGYTKE
lau Shrew        CIRDFFMKRFNYESELYPTF(S)DIKREDSLDIEVSASGYTKE
afr Elephant     CIRNFFIKRFSYESELYPAF(S)DIRREDSLDVEVSASGYTKE
afr Rock_hyrax   CIRDFFIKRFSYESELYPTF(N)DIRREDSLDVEVSASGYTKE
afr Tenrec       CIQDFFMKRFTYESELYPTF(N)DIRREDSLDVEVSASGYTKE
xen Armadillo    CIRDFFMKRFSYESELYPTF(S)DIXXXXXXXXXXXXXXXXXX
xen Sloth        CIRDFFMKRFSYESELYPTF(S)DIRRTNSLEVEVSASGYTKE
mar Opossum      CIRDFFIKRFSYESELYPTF(G)DISSPXSLDVEISASGYTKE
mar Wallaby      GIRDFFIRCFNNESEFYPTF(G)DIKREGCVEAEISASEYTKE
pro Platypus     CIRDFFIKRFNYESELYPTF(G)DIRRENSLDVEIAASGFTKE

NO 208
GN RNF25
IP IPI00059944
PE AMLDPPKPSRGPW
MP 341
EX Wagner
CL simians
DE ring finger protein 25
SQ
pri Human        GTRSNQQRLGETQKAMLDPP(K)PSRGPWRQPERRHPKGGECH
pri Chimpanzee   GARSNQQRLGETQKAMLDPP(K)PSRGPWRQPERRHPKGGECH
pri Gorilla      GARSNQQRLGETQKAMLDPP(K)PSRGPWRQPERRHPKGGECH
pri Orangutan    GARSNQQRLGETQKAMLDPP(K)PSRGPWRQPERRHPKGGECH
pri Gibbon       GARSNQQRLGETQKAMLDPP(K)PSRGPWRQPERRHPKGGECH
pri Rhesus       GAGSNQQRLGETQRAMLDPP(K)PSRGPWRQPERRHPKGGECH
pri Baboon       GAGSNQQRLGETQKAMLDPP(K)PSRGPWRQPERRHPKGGECH
pri Marmoset     GAGPNQQRLGETQKAVLDPP(K)PSRGPWRQPERRYLKGGECH
pri Tarsier      GAGPNQQELGETQKAMLGPP(Q)PNRGPWRQPERRHLKGGECR
pri Bushbaby     GAGPHQQRLGETQKSMLDIP(Q)PSRGPWRQPERRHLKGGECY
pri Mouse_lemur  GAGP-PQRLGETQKAMLDTP(Q)PTRGPWRQPERRHLKGGQCC
eua Mouse        GAGPNQQRPGETQKSVLDPP(R)HGRGSWRQYDRRHPKGGECC
eua Rat          GAGPNQQRLGETQKSMLDPP(R)PGRGSWRQYDRRHPKGGECC
eua Kangaroo_rat GAGLNQQRLGETQKAMLDPP(R)SGRGPWRQSNRRHLKGGECC
eua Guinea_pig   GTGPNQQKLGENKKAMLDPP(R)ASQGPWRQPSQRHLKGGEFS
eua Squirrel     GTGPNQQRLGETQKATLDPP(R)SSQSPWRQPSRRHLKGGECC
lau Dog          GAGPNQQRLGETQKAMIDPP(R)ASRGPWKQPERRHLKGGECH
lau Cat          GAGPNQQRLGETPKTMLDPP(R)AGRGPWRQPERRHLKGGECN
lau Horse        GTGPNQQRLGETQKAMLDAP(R)ATRGPWRQPERRHLKGRECN
lau Cow          GAGPHLPKLGETQKAVLDPR(R)ASRGPWRQPERRHLKGGECN
lau Dolphin      GAGPNQQKLGETQKAMLDPP(R)ASRGPWRQAERRHLKGGECN
lau Alpaca       GAGPNQQKLGETQKAMLDPP(R)ASRGPWRQPERKHLKGGECN
lau Megabat      RAEPNQQKLGETQKAMLDPP(R)ASRGPWRQPERKHLKGGEYN
lau Microbat     GTGPNQQRLSETQKTILDTP(R)ANRGPWRQPERRHLKGGEYN
afr Elephant     GARPNQQRLGKTQKAMPDPP(R)ANRDSWQPPERRYLKGGECY
afr Rock_hyrax   GARPSQQRLGKTQKAMPDPS(R)ASRDTWQPPEQRHLKGGKCC
afr Tenrec       RVGPPRPRLGETPKAPPGPP(R)ASRNPWHPPERRPLKGGQCC
xen Armadillo    EAGLNPQRLGESQKAVPDLP(R)ASRGPWRQLERRRLKGGECY

NO 209
GN RPF1
IP IPI00292221.3
PE FASYFNKQTSPKI
MP 138
EX Kim
CL simians
DE ribosome production factor 1 homolog (S. cerevisiae)
SQ
pri Human        PNDEEVAYDEATDEFASYFN(K)QTSPKILITTSDRPHGRTVR
pri Chimpanzee   PNDEEVAYDEATDEFASYFN(K)QTSPKILITTSDRPHGRTVR
pri Gorilla      PNDEEVAYDEATDEFASYFN(K)QTSPKILITTSDRPHGRTVR
pri Orangutan    PNDEEVAYDEATDEFASYFN(K)QTSPKILITTSDRPHGRTVR
pri Gibbon       PNDEEVAYDEATDEFASYFN(K)QTSPKILITTSDRPHGRTVR
pri Rhesus       PNDEEVAYDESTDEFASYFN(K)QTSPKILITTSDRPHGRTVR
pri Baboon       PNDEEVAYDESTDEFASYFN(K)QTSPKILITTSDRPHGRTVR
pri Marmoset     PNDEEVAYDEATDEFASYFN(K)QTSPKILITTSDRPHGRTVR
pri Tarsier      PND-KVAYDEATDEFASYFN(R)QTSPKILITTSDRPHGRTVR
pri Bushbaby     PNDEEVAYDEATDEFASYFN(R)QTSPKILITTSDRPHGRTVR
pri Mouse_lemur  PNDEEVAYDEATDEFASYFN(R)QTSPKILITTSDRPH-RTVR
eua Treeshrew    PNDEEVAYDEATDEFASYFN(R)QTSPKILITTSDRPH-RTVR
eua Mouse        PNDEEVAYDEATDEFASYFN(R)QTSPKILITTSDRPHGRTVR
eua Rat          PNDEEVAYDEATDEFASYFN(R)QTSPKILITTSDRPHGRTVR
eua Guinea_pig   PNDEEVAYDEATDEFAPYFN(R)ETSPKILITTSDRPHGRTVK
eua Rabbit       PNDEEVAYDEATDEFASYFN(R)QTSPKILITTSDRPHGRTVK
lau Dog          PNDEEVAYDEATDEFASYFN(R)QTSPKILITTSDRPHGRTVR
lau Horse        PNDEEVAYDEATDEFASYFN(R)QTSPKILITTSDRPHGRTVR
lau Cow          PNDEEVAYDEATDEFASYFN(R)QTSPKILITTSDRPHGRTVR
lau Dolphin      PNDEEVAYDEATDEFASYFN(R)QTSPKILITTSDRPHGRTVR
lau Alpaca       PNDEEVAYDEATDEFASYFN(R)QTSPKILITTSDRPHGRTVR
lau Megabat      PNDEEVAFDEATDEFASYFN(R)QTSPKILITTSDRPHGRTVR
lau Microbat     PNDEEVAYDEATDEFASYFN(R)QTSPKILITTSDRPHGRTVR
lau Hedgehog     PNDEEVAYDEATDEFASYFN(R)ETSPKILITTSDRPHGRTVR
lau Shrew        PNDEEVAFDEATDEFASYFN(R)QTSPKILITTSDRPHGRTVR
afr Elephant     PNDEEVAYDEATDEFASYFN(R)QTSPKILITTSDRPHGRTVR
afr Rock_hyrax   PNDEEIAYDEATDEFASYFN(R)QTSPKILITTSDRPHGRTVR
afr Tenrec       PNDEEVAYNEAIDEFASYFN(R)QTYADILITISERPH-RTV-
xen Armadillo    PNDE-VAYDEATDEFASYFN(R)QTSPKILITTSDRPHGRTVR
xen Sloth        PNDE-VAYDEATDEFASZFN(R)QTSPKILITTSDRPH-RTVR
mar Opossum      PNDEEVAYDEATDEFASHFN(R)QTSPKILITTSDRPRGRTVR
mar Wallaby      PNDEEVDYDEATNKFASHFN(R)QISPKILITIPDRIHG-RVZ
pro Platypus     PDDEEVTYDEATDEFASYFN(R)QTSPKILITTSDRPRGRTVR

NO 210
GN RUSC1
IP IPI00425688.2
PE SLRGTSKEAASDP
MP 711
EX Kim
CL simians
DE RUN and SH3 domain containing 1
SQ
pri Human        TMQAMLHFGGRLAQSLRGTS(K)EAASDPSDSPNLPTPGSWWE
pri Chimpanzee   TMQAMLHFGGRLAQSLRGTS(K)EAASDPSDSPNLPTPGSWWE
pri Gorilla      TMQAMLHFGGRLAQSLRGTS(K)EAASDPSDSPNLPTPGSWWE
pri Orangutan    TMQAMLHFGGRLAQSLRGTS(K)EAASDPSDSPNLPTPGSWWE
pri Gibbon       TMQAMLHFGGRLAQSLRGTS(K)EAASDPSDSPNLPTPGSWWE
pri Rhesus       TMQAMLHLGGRLAQSLRGTS(K)EAAPDPSDSPNLPTPGSWWE
pri Baboon       TMQAMLHLGGRLAQSLRGTS(K)EAAPDPSDSPNLPTPGSWWE
pri Marmoset     TMQAVLHLGGRLAQSLRGTS(K)EAAPDPSDSPNLPTPGSWWE
eua Mouse        TMQAVLHWGERLAQSLRGTS(G)ESTTDSSTPSARPPAGSWWD
eua Rat          TMQAVLHWGGRLAQTLRGTS(G)EATTDSSTPSTRPPPGSWWD
eua Kangaroo_rat TVQAMLHWGGRLAQSFRGSA(G)EAAPDPSAASNAPTSGSWWE
eua Guinea_pig   TMQAVLHWGGRLAQSLRGPS(E)EAIPNPSAPSSPPTPGSWWE
eua Squirrel     TVQAMLHWGGRLAQSLRGTS(G)EGAPNPSPPPSPPTPVSWWE
eua Rabbit       TVQAVLHWGGRLAQTLRGAS(G)EAAADASAPPSSSAAGSWWE
eua Pika         TVQAVLHWGGRLAQTLRGAS(E)EAATATSAPPKGPAPGGWWE
lau Dog          TVHAVLHWGGRLAQSLRGAP(G)DTPSGPSALESTPAPGSWWE
lau Cat          TVRAVLHWGGRLAQSLRGAS(G)ETPPGPSAPASPPAPGGWWE
lau Horse        TMQAMLHWGGRLAQSLRGAS(G)ETPPGPSASSSPPTQGSWWK
lau Cow          TVQAVLHWGGRLAQSLRGAS(G)EAPPGPSAPSSSPSPCSWWE
lau Dolphin      TVQAMLHWGGRLAQSLRGAS(G)ETPPGPSAPSSPPTPGSWWE
lau Megabat      TVQALLHWGGRLAQSLRGAS(G)ENPPSPSAPSGPPAAGSWWE
lau Microbat     TVQAMLHWGGRLAQSLRGAS(E)ETPPGPSAPSSPPKPGSWWE
afr Elephant     TVQALLHWGGRLAQSLRGAS(G)EAPPGPAAPTSPPTPGSWWE
afr Tenrec       TVQALLHWGGRLAQSLRGAS(G)EALPDPEAPTSPPTPGSWWE
xen Armadillo    TVQAVLHZGSRLAQSLQRAS(R)ETPSGPTAPPSPPTPASWWE
xen Sloth        TVQAMLHWGGRLAQSLRGTS(G)EAPSGPPAPPSPPTSGSWWE
mar Opossum      TVQAVLGWGGRLAQSLRGGP(G)EAPRGPSPS------SGWWE

NO 211
GN SCAF11
IP IPI00746412.2
PE PQSGWMKQEEETS
MP 1178
EX Kim
CL catarrhines
DE SR-related CTD-associated factor 11
SQ
pri Human        VQNYYSRRGRNSSGPQSGWM(K)QEEETSGQDSSLKDQTNQQV
pri Chimpanzee   VQNYYSRRGRNSSGPQSGWM(K)QEEETSGQDSSLKDQTNQQV
pri Gorilla      VQNYYSRRGRNSSGPQSGWM(K)QEEETSGQDSSLKDQTNQQV
pri Orangutan    VQNYYSRRGRNSSGPQSGWM(K)QEEETSGQDSSLKDQTNQQV
pri Gibbon       VQNYYSRRGRNSSGPQSGWM(K)QEEETSGQDSSLKDQTNQQV
pri Rhesus       VQNYYSRRGRNSTGPQSGWM(K)QEEETSGQDSSLKDQTNQQV
pri Baboon       VQNYYSRRGRNSTGPQSGWM(K)QEEETSGQDSSLKDQTNQQV
pri Marmoset     VQNYYSRRGRNSSGPQSGWM(R)QEEETSGQDSSLKDQTNQQV
pri Tarsier      VQNYYSRRGRNSSGPQSGWM(R)QEEETSEQDPNLKDQTNQQV
pri Mouse_lemur  VQNYYSRRGRNSSGPQSGWM(R)QEEETTEQXXXXXXXXXXXX
eua Treeshrew    VQNYYSRRGRNSSGPQSGWM(R)QEEETTEQDSNLKDQTNQQV
eua Mouse        VQSYYSRRGRNSSGPQSGWM(R)QEEETPEQDSNLKDQTNQ-V
eua Rat          VQNYYSRRGRSSSGPQSAWM(R)QEEETPEQDSNLKDQTNQ-I
eua Kangaroo_rat VQNYYSRRGRNSSGSQSGWM(R)QEEETTEQDSNLKDQTNQQV
eua Squirrel     VQNYYSRRGRNSSVPQSGWM(R)QEEETTEQDSNLKDQTNQEV
eua Rabbit       VQNYYSRRGRNSSGPQSAWI(R)QEEEAPDQDSNLKDQTGQQV
eua Pika         VQNYYSRRGRSASGSQSTWI(R)QEEDTADQDCNLKDQTSQQV
lau Dog          VQNYYSRRGRNSSGPQSGWM(R)QEEETTEQDSNLKDQTNQHG
lau Cat          VQNYYSRRGRNSSGPQSGWM(R)QEEEATEQDSNLKDQTNQHG
lau Cow          VQNYYSRRGRSSSGPQSGWM(R)QDEETAEQESNLKDQTNQQG
lau Dolphin      VQNYYSRRGRNSAGPQSAWM(R)QEEETTEQDSNLKDQTNQQG
lau Megabat      VQNYYSRRGRNSSGSQSGWM(R)QEEETAEQDSNLKDQTNQQG
lau Hedgehog     VQNYYSRRGRNSSGSQSGWT(R)QEEETAEQDSNQKDQTNQQG
lau Shrew        VQSYYSRRGRHSSGPQAGWM(R)QEEETAEQDSNIKDQTNQQS
afr Elephant     VQNYYSRRGRNSSGPQSGWM(R)QEEETAEQDSNLKDQTNQQG
xen Sloth        VQNYYSRRGRNSSGPQSGWM(R)QEEETAEQDPNLKDQANQQS
mar Opossum      VQNYYSRRGRNSSGPQSGWM(R)QEEETPEQDQNLKDQTNQQG
pro Platypus     VQNYYSRRGRNSSGPQSGWM(R)PEEETTEQDPNLKDQTNQQG

NO 212
GN SCARB1
IP IPI00177968.2
PE EIMWGYKDPLVNL
MP 184
EX Kim
CL apes
DE scavenger receptor class B, member 1
SQ
pri Human        TTLGERAFMNRTVGEIMWGY(K)DPLVNLINKYFPGMFPFKDK
pri Chimpanzee   TTLGERAFMNRTVGEIMWGY(K)DPLVNLINKYFPGMFPFKDK
pri Gorilla      TTLGERAFMNRTVGEIMWGY(K)DPLVNLINKYFPGMFPFKDK
pri Orangutan    TTLGERAFMNRTVGEVMWGY(K)DPLVNLINKYFPGMFPFKDK
pri Gibbon       TTLGERAFMNRTVGEIMWGY(K)DPLVNLINKYFPGMFPFKDE
pri Rhesus       TTLGERAFMNRTVGEIMWGY(Q)DPLVNLINKYFPGMFPFKDK
pri Baboon       TTLGERAFMNRTVGEIMWGY(Q)DPLVNLINKYFPGMFPFKDK
pri Marmoset     STLGERAFMNRTVGEIMWGY(S)DPLVNLINKYFPGTFPFKDK
pri Bushbaby     STLGQRAFMNRTVGEIMWGY(D)DPLVNMINKFFPGMFPLKGK
pri Mouse_lemur  STLGQRAFMNRTVAEIMWGY(D)DPLVNLINKYFPGMFPFKGK
eua Treeshrew    STLGERAFMNRTVAEIMWGY(E)DPLVSLINKYFPDMLPFKGK
eua Mouse        VTMGQRAFMNRTVGEILWGY(D)DPFVHFLNTYLPDMLPIKGK
eua Rat          VTMGQRAFMNRTVGEILWGY(E)DPFVNFLSKYFPDMFPIKGK
eua Kangaroo_rat STLGQRAFMNRTVGEVMWGY(E)DPLVNLLNKYFPDMLPVKGK
eua Guinea_pig   TTMGQRAFMNRTVGEVLWGY(D)DPLLDLMDKYFPGALPFKGK
eua Rabbit       SALGQRAFMNRTVGEIMWGY(E)DPLMNLINKYLPGVFPFKDK
lau Dog          STLGERAFMNRTIGEIMWGY(E)DPLIHLINKYLPNMLPFKGK
lau Cat          STLGERAFMNRTVGEIMWGY(E)DPLVHLVNKYLPNMFPFKGK
lau Horse        TTLGERAFMNRTVGEIMWGY(E)DPLMNLINKYFPNMFPFKGK
lau Cow          STLGQRAFMNRTVGEIMWGY(D)DPLIHLINQYFPNSLPFKGK
lau Dolphin      STLGERAFMNRTVSEIMWGY(E)DPLIHIVNKYFPNMFPFKGK
lau Alpaca       STLGERAFMNRTVSEIMWGY(E)DPLLHLINKYFPSTFPFKGK
lau Megabat      TTFNERAFMNRTVGEIMWGY(E)DPLVQLINKYFPNMFPFKGK
lau Microbat     SSLRQRAFMNRTVGEVMWGY(D)DPLVSLVNKYLPGVFPIKGK
afr Elephant     STLGERAFMNRTVGEIMWGY(E)DPLMDFINKYFPNLLPFNGK
afr Rock_hyrax   STLGERAFMNRTVGEIMWGY(E)DPILNLVNKYFPGMLPFSGK
afr Tenrec       STLGQRAFVNRTVGEFLWGY(D)DPLMNFINQYFPNALPVKGK
xen Armadillo    STLGERAFMNRTVADIMWGY(E)DPLVDLINKYFPDMIPFKGK
mar Opossum      SMFGQRAFLNRTVGEIMWGY(S)DPLIDLLNKYFPGLMPFKDK
mar Wallaby      TMFGQRAFMNRTVGEIMWGY(Q)DPLIDLLNKYFPNILPFKDK
pro Platypus     NSFNEHAFMNRTVSEIMWGY(E)DPFVEFLNKYLPGMIPFKGK

NO 213
GN SCFD2
IP IPI00141564.8
PE ARPDKRKLGSLGD
MP 196
EX Kim
CL simians
DE sec1 family domain containing 2
SQ
pri Human        LFPLLPQDVHLLNSARPDKR(K)LGSLGDVDSTTLTPELLLQI
pri Chimpanzee   LFPLLPQDVHLLNSARPDKR(K)LGSLGDVDSTALTPELLLQI
pri Gorilla      LFPLLPQDVHLLNSARPDKR(K)LGSLGDVDATALTPELLLQI
pri Orangutan    LFPLLPQDVHLLNSARPDKR(K)LGSLGDVDATALTPELLLQI
pri Gibbon       LFPLLPQDVHLLNSTRPDKR(K)LGSLGDVDATALTPELLLQI
pri Rhesus       LFPLLPQDVHLLNSTRPDKR(K)LGSLGDVDATALTPELLLQI
pri Baboon       LFPLLPQDVHLLNSTRPDKR(K)LGSLGDVDATALTPELLLQI
pri Marmoset     LFPLLPQDVHLLNSARPDKR(K)LGSLGDVDATALTPELLLQI
pri Bushbaby     LFPLLPQDVHLLNSVRPDKR(R)LASLGEVXAAALTPELLLQI
pri Mouse_lemur  LFPLLPQDVHLLNSARPDKR(R)LGSLGEVDAAALTPELLLQI
eua Treeshrew    LFPLLLQDLHLLNSARPDKR(R)LASLGEVDSTALTPELLLQI
eua Mouse        LFPLLPQDVHALNSARPDKR(R)LSSLGEVDATALTPELLLYI
eua Rat          LFPLLPQDVHVLNNARPDKR(R)LNSLGEVDATALTPELLLYI
eua Guinea_pig   VFPLLPQDLHLLNSARSDKR(R)LGNLGEVDATALTPELMLQM
eua Squirrel     LFPLLPQDVHLLNSARPDKR(R)LGSLGEVDASTLTPKLLLQI
eua Rabbit       LFPLLPGDVRVLNSARPDKR(R)LGSLAEVDAAALTPELQLQI
eua Pika         LFPLLPRDVRLLHGGRPDKR(R)LGSLAELDAAALPAELQLQI
lau Dog          LFPLLPRDVHLLNRARPDKR(R)LGSLSEVDAAALPPELLLQI
lau Cat          LFPLLPQDVHLLNSARPDKR(R)LGSLSEVDATALTPELFLQI
lau Horse        LFPLLPRDVHLLNSARPDKR(R)LGSLGEVDATALPPELLLQI
lau Cow          LFPLLPQDVHILNSARPDRR(R)LGSLAEVDATALTPELLLQI
lau Alpaca       LFPLLPQDVQILNSARPDKR(R)LGSLGEVDATALTPELLLQI
lau Megabat      LFPLLPQDVDLLNSARLDKK(R)MGSLGEVDSTALTPELLLQI
lau Microbat     LFPLLPQDVHLLNSARPDKR(R)LGSLGEVDATALTPELLLQI
lau Hedgehog     LFPLLTQDLHLLNSARPDKR(R)LGSLGEMDASTLTPELLLQI
lau Shrew        LFPLLSQDVELLNSARPDKK(R)LGGLGEVDASALPPELLLQI
afr Elephant     LFPLLPQDVHLLNSARPDKR(R)LGSLAEVDATALTPELLLQI
afr Rock_hyrax   LFPLLPQDVHILNSARPDKR(R)LGSLAEVDATALTPELLLQI
afr Tenrec       LFPLLPQDVHLLNSARPDKR(R)LGSLADVDGPALTPELTLQI
xen Armadillo    LFPLLPRDVHLLNSARPEKR(R)LGSLAEVDATVLPPELLLQI
xen Sloth        LFPLLPQDVHLLNSARPEKR(R)LGSLGEVDATALTPELLLQI
mar Opossum      LFPLLPRDLKFLNSARPDKR(K)LGSLAEVDASTLTPELLLQI
mar Wallaby      LFPLLPRDLKLLNSARPDKR(K)LGSLAEVDASSLTPELLLHI

NO 214
GN SCO2
IP IPI00014458
PE GLTGSTKQVAQAS
MP 196
EX Wagner
CL humans
DE SCO cytochrome oxidase deficient homolog 2 (yeast)
SQ
pri Human        AMARYVQDFHPRLLGLTGST(K)QVAQASHSYRVYYNAGPKDE
pri Chimpanzee   AMARYVQDFHPKLLGLTGST(E)QVAQASHSYRVYYNAGPKDE
pri Gorilla      AMARYVQDFHPRLLGLTGST(E)QVAQXSHSYRVYYNAGPKDE
pri Gibbon       AMARYVQDFHPRLLGLTGST(E)QVAQASHSYRVYYSAGPKDE
pri Rhesus       AMARYVQDFHPRLLGLTGST(E)QIAQATHSYRVYYSAGPKDE
pri Baboon       AMARYVKDFHPRLLGLTGST(E)QIAQATHSYRVYYSAGPKDE
pri Marmoset     AMARYVQDFHPRLLGLTGST(E)QVAQASHSYRVYYSAGPKDE
pri Tarsier      AMAQSIQNFHSQVLGLRGSP(E)QVTQASLSCRVYYQAIPKDE
pri Mouse_lemur  AMARYVQDFHPRLLGLTGST(E)QVAQASRSYRVYYSAGPKDE
eua Treeshrew    AMARYVQDFHPRLLGLTGSP(E)QVAQVSRSYRVYYSAGPKDE
eua Mouse        AMARYVQEFHPRLLGLTGST(E)QVAHASRNYRVYYSAGPKDE
eua Rat          AMARYVQEFHPRLLGLTGST(E)QVAHASRNYRVYYSAGPKDE
eua Kangaroo_rat AMARYVQDFHPRLLGLTGST(E)QVAQVSRSYRVYYSAGPZDE
eua Guinea_pig   AMARYVQDFHPRLLGLTGSA(E)QVAQASRSYRVYYSAGPKDE
eua Squirrel     AMARYVREFHPRLLGLTGSV(E)QVAQASRSYRVYYSAGPKDE
eua Rabbit       AMARYVQDFHPRLLGLTGSA(E)QVAQVSRSYRVYYSAGPKDE
eua Pika         AMARYVRDFHPRLLGLTGSA(E)QVAEVSRSYRVYYSAGPPDA
lau Dog          AMARYVQDFHPRLLGLTGSA(E)QVAQVSRSYRVYYRAGPKDE
lau Cat          AMARYVQDFHPRLLGLTGSA(E)QVAQVSRSYRVYYSAGPKDE
lau Horse        AMARYVQDFHPRLLGLTGSA(E)QIAQASHNYRVYYSAGPKDE
lau Cow          AMARYVQDFHPRLLGLTGSA(E)QIAQVSRSYRVYYSAGPKDE
lau Dolphin      AMAHYVQDFHPRLLGLTGSA(E)QVAQVSRSYRVYYSAGPKDE
lau Megabat      AMARYVQDFHPRLLGLTGSA(E)QVAQVSRSYRVYYSAGPKDE
lau Microbat     AMARYVQDFHPRLLGLTGSA(E)QVAQVSRSYRVYYSAGPKDA
lau Hedgehog     AMARYVQDFHPKLLGLTGST(E)QVSHVTRSYRVYYSAGPKDE
afr Elephant     AMARYVQDFHPRLLGLTGST(E)QVAQASRNYRVYYSPGPKDE
xen Armadillo    AMARYVQEFHPRLLGLTGSA(E)QVAQASRSYRVYYSAGPKDE
mar Wallaby      AVGRYVGEFHPRLLGLTGTS(E)QVSQAARAYRVYYSAGPPDE
pro Platypus     ALGRYVRDFHPRLLGLTGTP(E)QVRRVARSYRVYYSAGPKDE

NO 215
GN SDR42E1
IP IPI00163504.4
PE LNRNLIKEVNVRG
MP 96
EX Kim
CL humans
DE short chain dehydrogenase/reductase family 42E, member 1
SQ
pri Human        VFHIASYGMSGREQLNRNLI(K)EVNVRGTDNILQVCQRRRVP
pri Chimpanzee   VFHIASYGMSGREQLNRNLI(E)EVNVRGTDNILQVCQRRSVP
pri Gorilla      VFHIASYGMSGREQLNRNLI(E)EVNVRGTDNVLQVCQRRRVP
pri Orangutan    VFHIASYGMSGREQLNRNLI(E)EVNVRGTDNILQACQRRRVP
pri Gibbon       VFHIASYGMSGREQLNRNLI(E)EVNIRGTDNILQACQRRRVP
pri Rhesus       VFHIASYGMSGREQLNRNLI(E)EVNIGGTDNILQACQRRRVP
pri Baboon       VFHIASYGMSGREQLNRNLI(E)EVNIGGTDNILQACQRRRVP
pri Marmoset     VFHIASYGMSGWEQLSRNPI(E)EVNIGGTDNILQACQRRMVP
pri Tarsier      VFHIASYGMSGREQLEQNLI(E)EVNIGGTDNVLQACQRRGVP
pri Bushbaby     VFHVASYGMSGREQLNRKLI(H)EVNVRGTDNVLQACQRRGVP
pri Mouse_lemur  VFHIASYGMSGREQLNGNLI(E)EVNVGGTDNVLQACRRRGVP
eua Treeshrew    VFHVASYGMSGREQLNHRLI(E)DVNVRGTVNILQACQRSGVP
eua Mouse        VFHVASYGMSGREQLNKTQI(E)EVNVGGTENILRACLERGVP
eua Rat          VFHIASYGMSGREQLNKTRI(E)EVNVGGTENILQACLGRGVP
eua Guinea_pig   VFHIASYGMSGREQLNQNLI(E)EVNVGGTDNILQACWRWGVP
eua Rabbit       VFHIASYGMSGREQLNRRRI(E)EVNVGGTENILRVCRRTGVP
eua Pika         VFHIASYGMSGREQLNRSQI(E)EVNVGGTDNILQACRRNGVP
lau Dog          VFHIASYGMSGREQLNRSLI(E)EVNVGGTDNILQVCRRRGVP
lau Cat          VFHIASYGMSGREQLNRSLI(E)EVNVGGTDHILQVCRRRGVP
lau Horse        VFHVASYGMSGKEQLDRKLI(E)EVNVGGTDNVLQACRRRGVP
lau Cow          VFHIASYGMSGREQLNRSLI(E)EINVGGTDNILQACRRRGVP
lau Dolphin      VFHIASYGMSGREQLNRSLI(E)EVNVGGTDNILQACRRRGVP
lau Alpaca       VFHTASYGMSGREQLNRNLI(E)EVNVRGTDNVLQACRKRGVP
lau Megabat      VFHTASYGIAGQEQLNRNMI(K)EVIVGGTDNILQTRKRRGVP
lau Microbat     VFHIASYGMSGREQLNRKLI(E)EVNVGGTDNILQACWRRGVP
lau Hedgehog     VFHIASYGMSGREQLNRNLI(Q)EVNVGGTDNILQACRRSGVP
lau Shrew        VFHVASYGMSGREQLHPTLI(E)AVNVAGTENVLRACRRRGVA
afr Elephant     VFHIASYGMSGREQLNRNLI(E)EVNVRGTDNMLQACRRRGVP
afr Rock_hyrax   VFHIASYGMSGREQLNRSLI(E)EVNVRGTDNILQTCRRRGVP
afr Tenrec       VFHVASFGMSGREQLDSHLI(E)DINVRGTENVLQACQRLGVP
xen Armadillo    VFHIASYGMSGREQLNRNLI(E)EVNVRGTDNVLQLCRRRGVP
xen Sloth        VFHIASYGMSGQEQLNRNLI(E)EVNVGGTENVLQVCRRKGVP
mar Opossum      VFHIASFGMSGKEQLNHKLI(E)SVNVKGTENVLEACRRKGVP
mar Wallaby      VFHIASFGMSGKEQLNHKRI(E)DVNVKGTENVLQACRRKGVP
pro Platypus     VFHIASYGMSGREQLDRKLI(E)DVNVKGTENVIRACRRRAVP

NO 216
GN SDR42E1
IP IPI00163504.4
PE KKELGYKAQPFDL
MP 337
EX Kim
CL haplorhines
DE short chain dehydrogenase/reductase family 42E, member 1
SQ
pri Human        YKTGVTHYFSLEKAKKELGY(K)AQPFDLQEAVEWFKAHGHGR
pri Chimpanzee   YKTGVTHYFSLEKAKKELGY(K)AQPFDLQEAVEWFKAHGHGR
pri Gorilla      YKTGVTHYFSLEKAKKELGY(K)AQPFDLQEAVEWFKAHGHGR
pri Orangutan    YKTGVTHYFSLEKAKKELGY(K)AQPFDLQEAVEWFKAHGHGR
pri Gibbon       YKTGVTHYFSLEKAKKELGY(K)AQPFDLQEAVEWFKAHGHGR
pri Rhesus       YKTGVTHYFSLEKAKKELGY(K)AQPFDLQEAVEWFKAHGHGR
pri Baboon       YKTGVTHYFSLEKAKKELGY(K)AQPFDLQEAVEWFKAHGHGR
pri Marmoset     YKTGVTHYFSLEKAKKELGY(K)AQPFDLQEVVEWFKAHGHGR
pri Tarsier      YKTGVTHYFSLEKAKKELGY(K)AQPFDLQEVVEWFKTHGHGR
pri Bushbaby     YKTGVTHYFSLEKARKELGY(E)AQPYDLQEVVEWFEAHGHGR
eua Mouse        YKTGVTHYFSLEKAKKELGF(E)PQPFDLQEVVEWFKAHGHGR
eua Rat          YKTGVTHYFSLEKAKSELGY(A)PQPFDLQEVVEWFKAHGHGR
eua Kangaroo_rat YKTGVTHYFSLEKAKKELGY(E)AQPYDLQEMVEWFRAHGHGK
eua Guinea_pig   YKTGVTHYFSLEKAKKELGY(E)AQPFDLQEVVDWFKAHGHGR
eua Rabbit       YKTGVTHYCSLEKAKKELGY(E)AQPFDLQEAVEWFKARGHGR
eua Pika         YKTGITHYCSLEKARKELGY(E)AQPFDFQEVVEWFKARGHGR
lau Dog          YKTGVTHYFSLDKAKKELGY(E)AQPFDLQDVVDWFKAHGHGR
lau Cat          YKTGVTHYFSLEKAKKELGY(E)AQPFDLQEVVDWFKAHGHGR
lau Horse        YKTGVTHYFSLEKAKKELGY(E)AQPFDLQEVVEWFKARGHGR
lau Cow          YKTGVTHYFSLEKARKELGY(E)AQPFDLQEAVEWFKAHGHGR
lau Dolphin      YKTGVTHYFSLEKAKKELGY(E)AQPFDLQEVVEWFKAHGHGR
lau Alpaca       YKTGVTHYFSLEKAKKELGY(E)AQAFDFQEVVEWFKAHGHGR
lau Megabat      YKTDITHYFSLEKAKKELGY(E)AWPFDLQEVVEZFKAHCHSK
lau Microbat     NKTGITHYFSLEKAKKELGY(E)PQPFDLQEIVEWF-ARGHGR
lau Hedgehog     YKTGVTHYFSLEKAKKELGY(E)PQPFDLQEVVEWFKAHGHGR
afr Elephant     YKTGVTHYFSLEKAKRELGY(E)AQPFGLQEVVEWFKAQGHGQ
afr Rock_hyrax   YKTGVTHYFSLQKARRELGY(E)AQSFGLQEVVEWFKAHGHGQ
xen Armadillo    YKTGVTHYFSLEKARKELSY(E)AQPFDLQEAVEWFKAHGHGR
xen Sloth        YKTGVTHYFSLEKARKELGY(E)AQPFDLQEVVEWFRAHGHGR
mar Opossum      YKTGITHYFSMEKARKELGY(E)AQPFDFNEVIDWLKAEGHGR
mar Wallaby      YKTGVTHYFSMEKAKKELGY(E)PQSFDFKEVVDWFKAEGHGR
pro Platypus     YKTGVTHYFSLEKARRELGY(E)AQVFDFREVVEWFKAQGHGR

NO 217
GN SEC11C
IP IPI00219436.3
PE DIFGDLKKMNKRQ
MP 23
EX Kim
CL apes
DE SEC11 homolog C (S. cerevisiae)
SQ
pri Human        RAGAVGAHLPASGLDIFGDL(K)KMNKRQLYYQVLNFAMIVSS
pri Chimpanzee   RAGAVGAHLPASSLDIFGDL(K)KMNKRQLYYQVLNFAMIVSS
pri Gorilla      RAGAVGAHLPASGLDIFGDL(K)KMNKRQLYYQVLNFAMIVSS
pri Orangutan    RAGAVGAHLPASGLDIFGDL(K)KMNKRQLYYQVLNFAMIVSS
pri Gibbon       RAGAVGAHLPASGLDIFGDL(K)KMNKRQLYYQVLNFAMIVSS
pri Rhesus       RAGAVGAHLPASGLDIFGDL(R)KMNKRQLYYQVLNFAMIVSS
pri Baboon       RAGAVGAHLPASGLDIFGDL(R)KMNKRQLYYQVLNFAMIVSS
pri Marmoset     RAGAVGAHLPTSGLDIFGDL(R)KMNKRQLYYQVLNFAMIVSS
pri Tarsier      RVGAVETHLPTTGLDIFGDL(R)KMNKHZLCYQMLKFAMIVSS
pri Bushbaby     RAGAVGTHLPTSGLDIFGDL(R)KMNKRQLYZQVLNFAMIMSS
pri Mouse_lemur  RAGAVGTHLPASGLDIFGDL(R)KMNKRQLYYQVLNFAMIVSS
eua Treeshrew    RAGSVGTHLPASGLDIFGDL(R)KMNKRQL-YQVLNFAMIVSS
eua Mouse        RAGAVGTHLPTSSLDIFGDL(R)KMNKRQLYYQVLNFAMIVSS
eua Rat          RAGAVGTHLPTSSLDIFGDL(R)KMNKRQLYYQVLNFAMIVSS
eua Kangaroo_rat RAGAVGAHLPASGLDIFGDL(R)KMNKRQLYYQVLNFAMIVSS
eua Guinea_pig   RAGSMGSHLPASCLDIFGDL(R)KMNKRQLYYQVLNFAMIVSS
eua Rabbit       RAGGVAAHLPASGLDIFGDL(R)KMNKRQLYYQVLNFAMIVSS
eua Pika         RAGGVAAHLPASGLDIFGDL(R)KMNKRQLYYQVLNFAMIVSS
lau Dog          RAGAVGTHLPASGLDIFGDL(R)KMNKRQLYYQVLNFAMIVSS
lau Cat          RAGAVGTHLPASGLDIFGDL(R)KMNKRQLYYQVLNFAMIVSS
lau Horse        RAGAVGTHLPASGLDIFGDL(R)KMNKRQLYYQVLNFAMIVSS
lau Cow          RASTVGAHLPASGLDIFGDL(R)KMNKRQLYYQVLNFAMIVSS
lau Dolphin      RAGAVGSHLPASGLDIFGDL(R)KMNKRQLYYQVLNFAMIVSS
lau Alpaca       RAGAVGSHLPASGLDIFGDL(R)KMNKRQLYYQVLNFAMIVSS
lau Megabat      RAGAVGTHLPASGLDIFRDL(R)KMNKRQLYYQVLNFAMIVSS
[truncated: 118,381 more chars]
