# Supplementary figures and images for: Gains of ubiquitylation sites in highly conserved proteins in the human lineage
Source: BMC Bioinformatics. 2012 Nov 17;13:306. doi: 10.1186/1471-2105-13-306 (PMC3561281; doi:10.1186/1471-2105-13-306)

**Supplementary Fig. S1.** Phylogenetic tree of the species analyzed in this study.

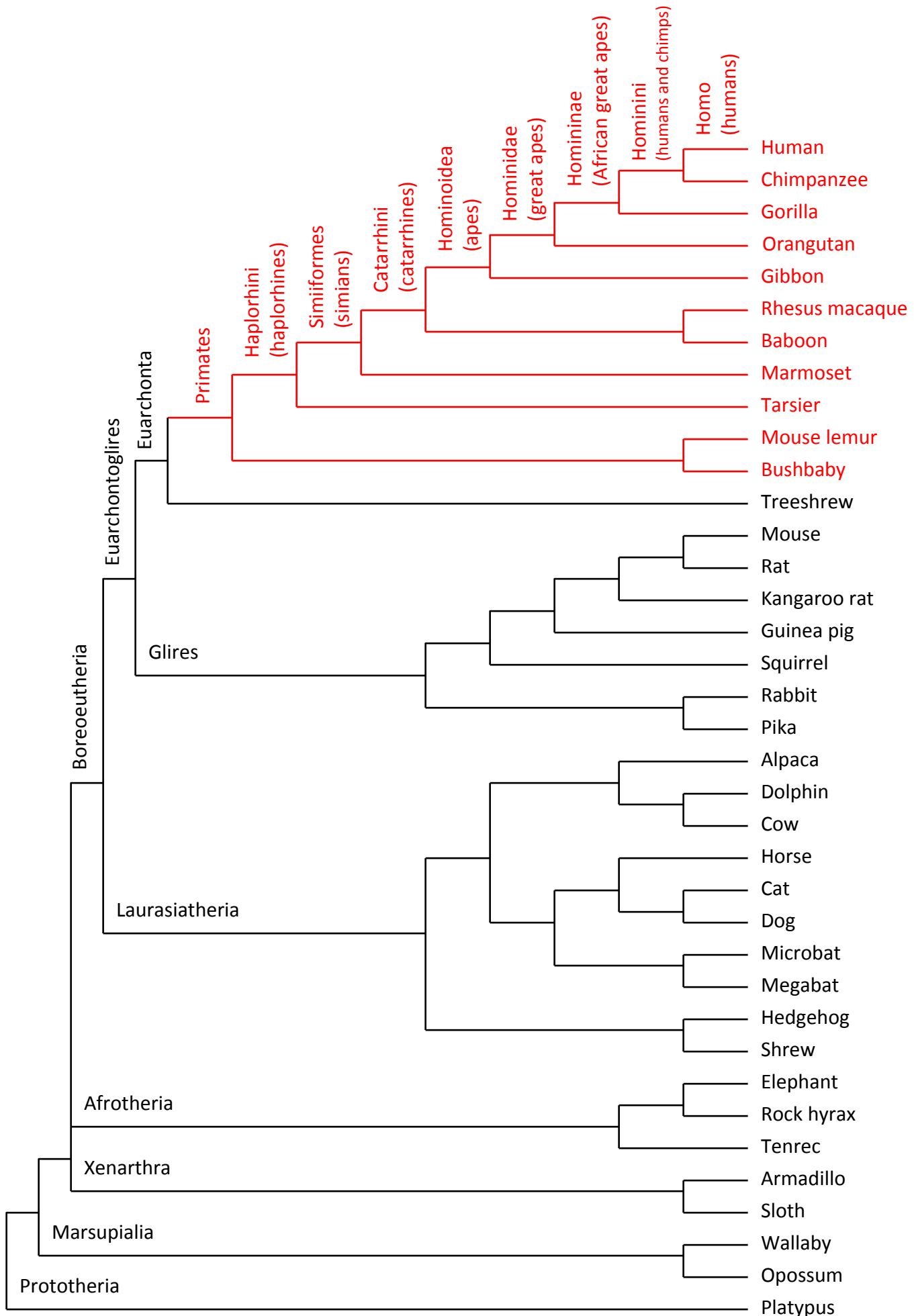

Supplement: Additional file 3 — Phylogenetic tree of the 37 mammals used in this study. [file 1471-2105-13-306-S3.pdf]
